# Systematic Study of Hard-Wall Confinement-Induced Effects on Atomic Electronic Structure

**DOI:** 10.1021/acs.jpca.4c05641

**Published:** 2025-01-29

**Authors:** Hugo Åström, Susi Lehtola

**Affiliations:** Department of Chemistry, Faculty of Science, University of Helsinki, P.O. Box 55, A.I. Virtanens Plats 1, University of Helsinki FI-00014, Finland

## Abstract

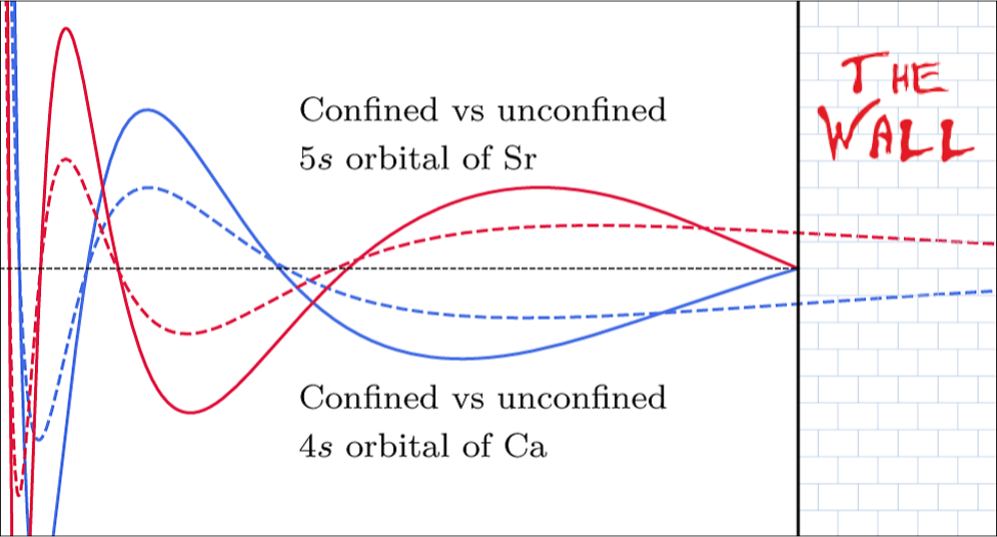

We point out that although a litany of studies have been published on atoms in hard-wall confinement, they have either not been systematic, having only looked at select atoms and/or select electron configurations, or they have not used robust numerical methods. To remedy the situation, we perform in this work a methodical study of atoms in hard-wall confinement with the HelFEM program, which employs the finite element method that trivially implements the hard-wall potential, guarantees variational results, and allows for easily finding the numerically exact solution. Our fully numerical calculations are based on nonrelativistic density functional theory and spherically averaged densities. We consider three levels of density functional approximations: the local density approximation employing the Perdew–Wang (PW92) functional, the generalized-gradient approximation (GGA) employing the Perdew–Burke–Ernzerhof (PBE) functional, and the *meta*-GGA approximation employing the r^2^SCAN functional. Importantly, the completely dissimilar density functional approximations are in excellent agreement, suggesting that the observed results are not artifacts of the employed level of theory. We systematically examine low-lying configurations of the H–Xe atoms and their monocations and investigate how the configurations—especially the ground-state configuration—behave as a function of the position of the hard-wall boundary. We perform calculations with both spin-polarized as well as spin-restricted densities and demonstrate that spin-polarization effects are significant in open-shell configurations, even though some previous studies have only considered the spin-restricted model. We demonstrate the importance of considering ground-state changes for confined atoms by computing the ionization radii for the H–Xe atoms and observe significant differences to earlier studies. Confirming previous observations, we identify electron shifts on the outermost shells for a majority of the elements: valence s electrons are highly unfavored under strong confinement, and the high-lying 3d and 4f orbitals become occupied in atoms of periods 2–3 and 3–4, respectively. We also comment on deficiencies of a commonly used density-based estimate for the van der Waals (vdW) radius of atoms and propose a better behaved variant in terms of the number of electrons outside the vdW radius that we expect will prove useful in future studies.

## 1 Introduction

The pioneering study of Michels et al.^1^ was concerned on the question of how atomic polarizabilities—which have an important role in chemical bonding—evolve as a function of pressure. Published in 1937, the study examined the hydrogen atom enclosed in an impenetrable sphere with a variable radius to simulate pressure effects. Such frugal models offer a great tool for understanding basic physical effects precisely due to their simplicity: Michels et al.^1^ found that hydrogen becomes less polarizable in increasing pressure, that is, when the radius of the sphere is decreased.

An interest in analogous studies of atoms in hard-wall confinement (i.e., atoms in a spherical box) continues to this day. A multitude of studies have been published on the very first atoms of the periodic table; see ref (2) for a review dedicated to the H and He atoms confined by a finite or infinite spherical barrier with 200 references. Yet, few studies have considered many-electron atoms or reported systematic calculations on the periodic table.

In hard-wall confinement, the wave function must vanish beyond the hard-wall boundary, and this can be accomplished only within a basis set with finite support. Many studies on confined atoms employing basis sets of an analytic form—such as Gaussian or Slater-type orbital basis sets—have been published in the literature. However, these basis sets have finite tails in the forbidden region. Truncated Gaussian or Slater-type orbital basis sets where this tail has been cut off do not have this issue, but they too suffer from basis set truncation errors (BSTEs), which are *a priori* unknown.

Our recent studies on atoms and molecules in strong magnetic fields found that changes in the electron configuration often resulted in significant increases in the BSTE of standard Gaussian-type orbital basis sets,^3,4^ as these basis sets have not been optimized to describe electronic structure in this context. As we will discuss below, confinement is likewise expected to result in electron shifts to orbitals of higher angular momentum. Since this effect has again not been considered in the parametrization of standard Gaussian-type orbital basis sets, they cannot be expected to yield reliable estimates under confinement since the orbitals of confined atoms can look extremely dissimilar to the orbitals of the unconfined atom. The qualitatively correct description of confinement-induced effects then requires a careful study of basis set convergence, ideally supplemented by comparisons to fully numerical reference values. We point out that few studies have attempted to reach the complete basis set (CBS) limit, and we will report numerically exact CBS limit energies later in this work.

Before laying out the scope of the present study, we will briefly review the literature we found that fulfills our key criteria at least in part; studies mentioned herein have examined atoms with more than 4 electrons and employed a robust numerical method.

Boeyens^5^ performed fully numerical Hartree–Fock calculations to determine the ionization radii of compressed atoms. However, Boeyens^5^ did not employ a proper hard-wall potential. To approximate a hard-wall boundary at *r* = *r*_c_, Boeyens^5^ multiplied the wave function by the smooth step function  at every self-consistent field (SCF) iteration before the normalization of the updated wave function was carried out. Boeyens^5^ employed standard electron configurations for the elements and thus did not consider changes in the occupations as a function of the confinement.

Chattaraj and Sarkar^6^ employed a similar approach to that of Boeyens^5^ in a density functional theory (DFT) study on chemical reactivity indices of the He–Ne and Br atoms, finding that the atoms become harder and less polarizable in increasing confinement, thus coming to the same conclusion as Michels et al.^1^ in the case of hydrogen 66 years earlier. Chattaraj and Sarkar^7^ also carried out a similar study for the reactivity indices for the He–Ne atoms and the C^+^, C^2+^, C^3+^, and C^4+^ ions, with the same conclusion. Yet, it appears that Chattaraj and Sarkar did not consider the possibility of the electron configuration changing as a function of confinement in refs (6 and 7).

Sarkar et al.^8^ studied the effects of confinement on the chemical reactivity of the nitrogen atom. Sarkar et al.^8^ compared the method of Boeyens^5^ to the true hard-wall potential and found the arising chemical reactivities to yield the same qualitative trends. Moreover, Sarkar et al.^8^ found that different methods to calculate the reactivity yield dissimilar results and that different electronic occupations also lead to different chemical reactivities.

Connerade et al.^9^ studied the filling of shells in the 3d and 4d elements as well as their cations in average-of-configurations Hartree–Fock calculations in a spherical cavity surrounded by a 10*E*_*h*_ potential wall. They found that orbital filling becomes more regular for successive rows with increasing pressure, that is, when the finite barrier is placed closer and closer to the nucleus. In specific, the s–d competition disappears for these elements, as the (*n* – 1)d orbitals become energetically more favorable than the *n*s orbitals under confinement. Connerade and co-workers have also reported studies on some individual atoms under confinement. For instance, Connerade and Dolmatov^10^ examined the Cr atom, while Connerade and Semaoune performed relativistic calculations on the La and Cs atoms in refs (11 and 12), respectively.

A number of studies of atoms in hard-wall confinement have been carried out at the DFT level by Garza et al. Garza et al.^13^ reported a finite differences method implementation for solving the atomic DFT equations in the presence of a hard-wall boundary and studied the Ne and Na (and He) atoms in exchange-only calculations. In a follow-up study, Garza et al.^14^ examined the electrostatic potential of the unconfined and hard-wall confined Li, Na, K, and Rb atoms with DFT in order to study changes in the atoms’ shell structure and found that the shell structure is gradually lost in increasing confinement. Similarly, Sen et al.^15^ determined atomic ionization radii for the He–Ca atoms with hard-wall confined DFT calculations. As none of the studies by Garza et al.,^13^ Garza et al.,^14^ or Sen et al.^15^ discuss occupations, we can only assume that the ground-state electron configuration of the unconfined atom was used in their calculations. In contrast, the study on the chemical reactivity indices of the compressed Li, Na, and K; Be, Mg, and Ca; N, P, and As; and Ne, Ar, and Kr atoms by Garza et al.,^16^ which was likewise carried out with hard-wall confined DFT, explicitly mentions finding the lowest electron configuration for each confinement radius.

Continuing with the Garza group, Guerra et al.^17^ modeled pressure effects on the electronic properties of Ca, Sr, and Ba with hard-wall confined DFT and found that their ground state changes from the *n*s^2^ configuration to the *n*s^1^(*n* – 1)d^1^ and *n*s^0^(*n* – 1)d^2^ configurations in increasing pressure. Confinement effects on the spin potential of first-row transition metal cations were investigated with hard-wall confined DFT by Lozano-Espinosa et al.^18^

Pašteka et al.^19^ studied the C and K (and He) atoms in hard-wall confinement with the finite element method (FEM) at the complete active space SCF^20,21^ (CASSCF) level of theory, which represents the most sophisticated level of theory in the studies we found. Also Pašteka et al.^19^ found changes in atomic ground-state configurations under confinement: the ground-state configuration of carbon changes from 1s^2^2s^2^2p^2^ in the free atom to 1s^2^2p^4^ in strong confinement.

As this literature overview demonstrates, although several fully numerical studies have been published on atoms in confinement, they have mostly focused on small groups of similar atoms. The study of Boeyens^5^ was the most complete, considering nonrelativistic calculations on the first 105 atoms of the periodic table, but the study relied on an *ad hoc* implementation of confinement, and—like in many other studies mentioned above—changes in the ground state as a function of confinement were not considered. Studies that considered changes in the ground state are likewise limited: Connerade et al.^9^ only considered 3d and 4d elements, Garza et al.^16^ only studied atoms of groups 1, 2, 15, and 18, and Guerra et al.^17^ only studied heavy atoms of group 2.

The only study that appears to have systematically investigated effects of confinement on the electronic structure of atoms so far is the work of Rahm et al.,^22^ which studied the first 93 atoms under pressure, employing the ANO-RCC Gaussian basis set^23^ with scalar relativistic corrections and the 25% hybrid^24,25^ of the Perdew–Burke–Ernzerhof (PBE) functional,^26,27^ which is commonly known as PBE0. Rahm et al.^22^ identified electron shifts, s → p, s → d, s → f, and d → f, as essential chemical and physical consequences of compression.

In contrast to the literature reviewed above on hard-wall confinement, the study of Rahm et al.^22^ employed the extreme pressure polarizable continuum model^28,29^ (XP-PCM) to describe pressure effects. In contrast to the presently employed hard-wall confinement model employing an infinite potential barrier, the XP-PCM model employs a finite step-function potential barrier at the boundary.^28,29^ When the pressure is increased, the size of the cavity is decreased, and the height of the potential barrier is increased. The XP-PCM model has also been used in combination with Gaussian basis sets to study atoms’ nonbonded radii,^30^ relations between atomic radii and electronegativity,^31^ and the pressure-dependence of atoms’ various electronic properties such as electronegativity, hardness, electrophilicity, electron density, electron affinity, and ionization potential.^22,32,33^ Yet, as already discussed above, Gaussian basis sets cannot be expected to deliver reliable results in strong confinement,^19^ and Rahm et al.^22^ also did not report carrying out a basis set convergence study of their findings.

As is clear from the above discussions, there is a hole in the literature regarding systematic, fully numerical studies covering (a significant fraction of) the periodic table. Changes in the electronic structure of atoms are an expected effect of confinement,^34,35^ and they thus need to be taken into account by considering changes in the electronic occupations as a function of confinement. Fully numerical techniques are critically important as changes in the electronic occupations cannot be expected to be adequately reproduced by standard Gaussian-type orbital basis sets, for example.

In this work, we will address this major shortcoming of the existing literature with a systematic study of atoms in hard-wall confinement within a fully numerical approach while also thoroughly considering the effect of the confinement on the ground-state electron configuration.

Our results confirm previous partial findings of the fully numerical studies of Connerade et al.,^9^ Guerra et al.,^17^ and Pašteka et al.^19^ as well as the thorough Gaussian basis study of Rahm et al.:^22^ confinement-induced electron shifts can be observed for a majority of the elements. It is important to note here that all of these studies were carried out with different methodologies. Furthermore, as we shall see, the dissimilar functionals that we employ in this work are all in good agreement. This suggests that the findings of this work indeed correspond to physical effects of confinement despite the arguably simplistic level of theory employed herein.

The layout of this work is the following. The theory behind the present work is briefly discussed in Section 2. The form of the Hamiltonian including the hard-wall potential and the employed DFT approach is discussed in Section 2.1. The employed theories for calculating ionization energies and ionization radii are discussed in Sections 2.2 and 2.3, respectively. As our study includes data on a variety of electron configurations, we discuss ways to estimate the corresponding atomic radii in Section 2.4, where we also introduce a new way to estimate atoms’ van der Waals (vdW) radii. Next, the computational details including the numerical approach employed are presented in Section 3. The results are presented in Section 4. We first demonstrate that our method works by computing ionization energies and ionization radii in Sections 4.1 and 4.2, respectively. Then, we analyze the numerical and physical behavior of the employed vdW radius estimates in Section 4.3 and find our vdW estimate to address many issues in a commonly used estimate. The highlights from our analysis of the behavior of the low-lying configurations of the H–Xe atoms in hard-wall confinement are presented in Section 4.4. The article concludes in a summary and discussion in Section 5. Atomic units are employed throughout the work. The full set of raw data as well as a detailed analysis of the results is included in the Supporting Information.

## 2 Theory

### 2.1 DFT Approach

The Hamiltonian for the confined atoms reads
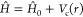
1where
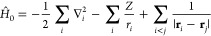
2is the standard electronic Hamiltonian for an atom with atomic number *Z*, and *V*_c_(*r*) is the potential for a hard-wall boundary at *r*_c_
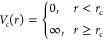
3

As already mentioned in Section 1, we carry out calculations on many-electron atoms within the context of DFT.^36,37^ We expand the one-particle states, also known as atomic orbitals (AOs), in a numerical basis set as
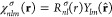
4where  are spherical harmonics; we will discuss the form of the radial expansion *R*_*nl*_^σ^(*r*) below in Section 3.

Following standard practice in numerical DFT calculations on atoms, we assume that the electron density is spherically symmetric, *n*_σ_(**r**) = *n*_σ_(*r*) for each spin σ. This simplification leads to an efficient algorithm, since the problem of solving the Kohn–Sham equations,^37^ i.e., the optimization of the s, p, d, and f orbitals, becomes a set of coupled one-dimensional problems.^38,39^

Furthermore, we will assume integer occupations on the s, p, d and f shells with the aim to extract chemical understanding from the calculations. These integer occupations are then averaged over all magnetic sublevels to produce spherically symmetric total densities; this leads to fractional occupations on the individual spatial orbitals. The electron density is thus given simply as
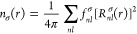
5where *f*_*nl*_^σ^ is the number of spin-σ electrons on the *nl* shell. The occupations *f*_*nl*_^σ^ are determined from the number of electrons with angular momentum *l* using Hund’s rules: the orbitals are filled starting from *n* = *l* + 1 by occupying the 2*l* + 1 spin-up orbitals for the given *n* before occupying the corresponding 2*l* + 1 spin-down orbitals. We refer to refs (38 and 39) for further discussion.

The above choice of theory has both physical and practical rationale. The fractional occupation formalism automatically guarantees spherical degeneracy,^38,39^ since all magnetic sublevels are assumed to behave identically. In contrast, DFT calculations with integer occupations of the magnetic sublevels often break spatial symmetry and fail to reproduce the degeneracy of atomic multiplets.^38^ Although several specialized approaches to reproduce atomic multiplets within a DFT style description have been proposed,^40−44^ they appear to be quite involved and have not gained wide adoption. We also wish to direct the reader to the discussion of Baerends et al.^45^ on atomic reference energies for DFT calculations.

### 2.2 Ionization Energies

Within the above level of theory, atoms’ ionization energies can be calculated in two ways. First, in the ΔSCF method, the ionization energy is determined for each value of *r*_c_ as the difference between the total energy of the cation and of the neutral atom
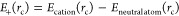
6As is discussed below in Section 3, the calculations on unconfined atoms employ the value *r*_c_ = 40*a*_0_, which is large enough to afford fully converged energies for the ground states of atoms and their cations.

Second, Boeyens^5^ used Koopmans’ theorem^46^ to determine the ionization radii. While there is no Koopmans’ theorem for DFT as in Hartree–Fock, Janak’s theorem^47^ provides a justification for an analysis based on orbital energies. The ionization energy is determined via Janak’s theorem as
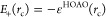
7where ε^HOAO^(*r*_c_) is the eigenvalue of the highest occupied atomic orbital of the neutral atom with the hard-wall boundary at *r* = *r*_c_.

### 2.3 Ionization Radii

Now that we have defined ways to calculate ionization energies, we can calculate ionization radii for atoms, i.e., the location of the hard-wall boundary where the atom favors casting away its outermost electron. The ionization radius *r*_+_ for atom X is by solving the nonlinear equation
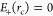
8

If the ionization energy is computed with eq 6, eq 8 is straightforward to solve to high precision by bisection once a crossing between the atomic and cationic curves has been identified. Now, the issue becomes that the energy of the atom in its neutral and cationic charge states depends on the employed electron configurations. This issue can be approached in two ways. The first is to follow the work of Boeyens^5^ and many others (see Section 1) and fix the electron configurations to that of the ground state of the unconfined neutral atom and its monocation, respectively. The second is to realize that confinement can affect the ground-state configurations of the atom and its cation and to compute the energies *E*_neutral atom_(*r*_c_) and *E*_cation_(*r*_c_) for the lowest configurations of the neutral atom and cation, respectively, at each value of *r*_c_.

Alternatively, we can use the estimate from Janak’s theorem of eq 7 to solve eq 8. Again, one can choose to use the electron configuration of the ground state of the unconfined neutral atom or to employ a relaxed configuration at every value of *r*_c_. However, the exact solution to eq 8 is sometimes problematic in the latter case. A case in point is the V atom: the value of *r*_+_ corresponds to the location of a ground-state crossing between a state with ε^HOAO^(*r*) < 0 and a state with ε^HOAO^(*r*) > 0, which means that eq 8 is not truly satisfied no matter how tightly *r*_+_ is converged.

The origin of the above problem is the present use of electron configurations with integer occupations. However, it has long been known that the use of nonintegral occupation numbers for the 4s and 3d shells can result in a lower total energy for transition metal atoms in average-of-configuration Hartree–Fock calculations.^48^ This phenomenon is also topical for the present DFT calculations^38,49^ and has also been found in DFT calculations that do not employ any exchange–correlation functional.^50^ Calculations employing variationally optimized fractional occupation numbers would allow finding an exact solution to eq 8, likely producing a slightly different radius. However, we do not consider such fractional occupations in this work since integer occupation numbers are more commonly used and allow a clearer physical interpretation of the results.

### 2.4 Atomic Radii

The location of the density maximum of the outermost orbital is a long-established estimator for the size of covalently bound atoms^51^
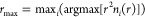
9where *n*_*i*_(*r*) is the *i*th orbital density.

Since the electron density of an atom decays in the far valence region, a density threshold can be used to estimate the atom’s vdW radius^52^
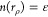
10where the electron density was defined above in eq 5, and the solution of eq 10 with a given threshold ε defines the vdW radius estimate *r*_ρ_.

Due to several issues related to eq 10 discovered and discussed in detail later in this work (for instance, eq 10 does not have a solution in some cases, leaving the vdW radius undefined), we propose another metric for the vdW radius in this work. The vdW radius can be determined by containing all the electrons of the atom except ε within the volume enclosed by that radius
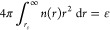
11where the electron density is again defined by eq 5. This metric is well-defined for any configuration of any element and also exhibits superior numerical stability over eq 10; see Section 4.3 for discussion. Equation 11 is also straightforward to extend to nonspherically symmetric electron densities as
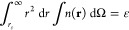
12which will be relevant for applications with the CASSCF method, for instance.

## 3 Computational Details

FEM offers a straightforward way to the numerical solution of the radial functions *R*_*nl*_^σ^;^53^ a review of the employed FEM approach can be found in refs (54 and 55). In short, the radial domain is first divided into *N*_elem_ segments *r* ∈ [*r*_*i*_^start^,*r*_*i*_^end^] called elements. An exponential radial grid  is employed, so that the size of the elements increases with the distance from the nucleus.^54,55^ A piecewise polynomial basis of shape functions *B*_*n*_(*r*) is then built up in each element.^54,55^ Finally, the numerical radial basis functions to be used in eq 4 are built from FEM shape functions *B*_*n*_(*r*) as
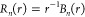
13and the same radial basis set is used for all angular momenta *l*. This approach is robust and allows maximal flexibility: as the basis functions within one element have zero overlap with basis functions in other elements, the spatial representation can be adaptively refined, if necessary.

The end point of the last element is called the practical infinity, *r*_∞_, and all basis functions are built to vanish at *r*_∞_.^54,55^ The physical interpretation of *r*_∞_ is that there is a hard-wall potential at this point; eq 3 is therefore already built-in in the FEM approach, and this feature has been previously used in many studies, such as refs (19 and 55). Thus, *r*_∞_ represents a physical parameter that expresses the location of the hard-wall boundary. In contrast, in studies of unconfined atoms (which represent the typical applications of FEM in the literature), *r*_∞_ is a discretization parameter that needs to be converged such that the obtained solution does not change if an even larger value is employed for *r*_∞_.

As our main focus is on atoms in confinement, the calculations for unconfined atoms employed the default value *r*_∞_ = 40*a*_0_, which is sufficient to reproduce the CBS limit energy of the ground states of neutral atoms and their cations. We note here that as larger values of *r*_∞_ may be necessary to capture the behavior of loosely bound states, the energies of the excited states in unconfined atoms may not be converged to the CBS limit with respect to this parameter. In contrast, the energies for the excited states in the confined atoms are converged to the CBS limit, as is explained below.

All calculations in this work were carried out with the free and open-source^56^HelFEM program; the present implementation is publicly available on GitHub.^57^ The calculations employed shape functions defined by 15-node Lagrange interpolating polynomials specified by Gauss–Lobatto quadrature nodes; this corresponds to the use of a 14th order polynomial basis set. All calculations were converged to the CBS limit with respect to the number of radial elements; radial elements were added until 1μ*E*_*h*_ precision in the total energy was achieved. We can thus be assured that we have found the numerically exact wave function.

We employ the optimal FEM implementation of the fractional occupation formalism recently described in refs (38 and 39). We perform calculations within the local density approximation^58,59^ employing the Perdew–Wang (PW92) correlation functional,^60^ within the generalized-gradient approximation (GGA) employing the Perdew–Burke–Ernzerhof (PBE) exchange–correlation functional,^26,27^ and within the *meta*-GGA approximation employing the r^2^SCAN exchange–correlation functional,^61,62^ all as implemented in Libxc(63) using the lda_x-lda_c_pw, gga_x_pbe-gga_c_pbe, and mgga_x_r2scan-mgga_c_r2scan keywords, respectively.

We initiated the work by identifying the three lowest-lying configurations of all elements with atomic number 1 ≤ *Z* ≤ 54 in the hard-wall potential for each *r*_c_ ∈ {1.0, 1.2, 1.4, ..., 10.0}*a*_0_ with both spin-restricted and spin-polarized densities, respectively, with a fixed angular momentum cutoff *l*_max_ = 3. The configurations were determined with the automated algorithm introduced in ref (38). The configurations considered for the spin-(un)restricted analysis were then chosen as the union of the low-lying configurations obtained from the spin-(un)restricted search over all of the values mentioned above of *r*_c_.

We checked whether *g* orbitals would become occupied for heavy atoms by repeating the configuration search for the Rb and Xe atoms with *l*_max_ = 4. However, no configurations with occupied *g* orbitals were obtained in this search.

The configuration search above was performed with the PBE functional only. However, as we shall see in Section 4, the results are qualitatively independent of the employed density functional approximation. For this reason, we are confident that our search yielded all relevant configurations for the PW92 and r^2^SCAN functionals as well.

Thus, being convinced to have found the low-lying configurations for the H–Xe atoms, we performed calculations on all these configurations with *r*_c_ ∈ {1.0, 1.1, ..., 10.0}*a*_0_. The wide range of studied values of *r*_c_ allows us to observe many interesting evolutions. The states with *r*_c_ = 10.0*a*_0_ are similar to those of the unconfined atom, while *r*_c_ = 1.0*a*_0_ represents extreme confinement, where even core orbitals experience confinement effects, and the valence electrons are bound only by the confinement potential.

## 4 Results

### 4.1 Ionization Energies of Unconfined Atoms

As a first step to validate our methods, we compute ionization energies for the unconfined H–Xe atoms in the spin-restricted and spin-polarized formalisms according to the two methods discussed in Section 2.2.

**Figure 1:**
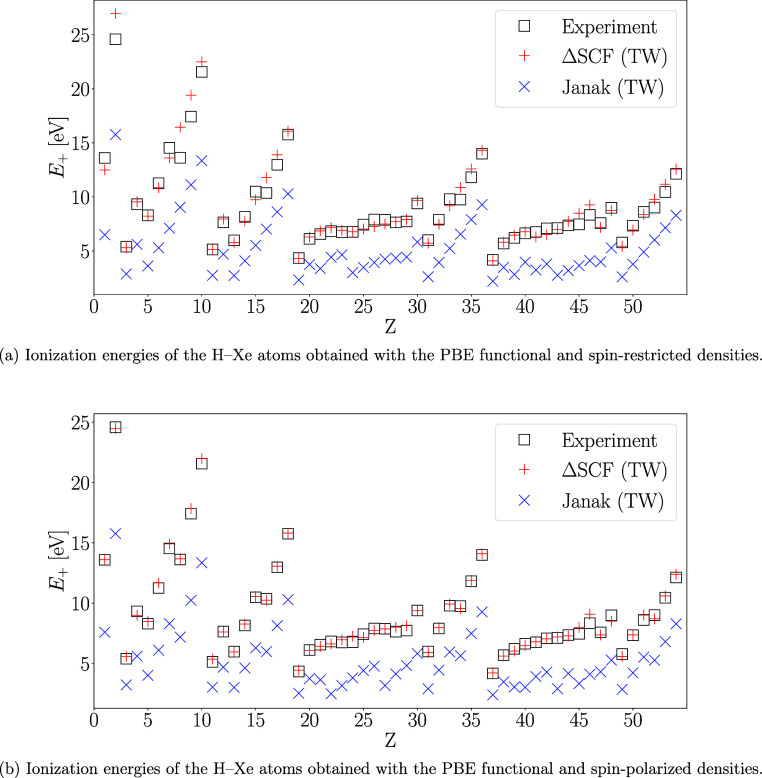
Comparison of the ionization energies of unconfined atoms computed with spin-restricted (Figure 1a) and spin-polarized (Figure 1b) densities in this work (TW) with Δ*S*CF or Janak’s theorem against experimental values of Kramida et al.^64^

As the three dissimilar functionals yield ionization energies that are all in good agreement with the experimental data, we discuss only the PBE data here. The comparison of our PBE values to the experimental values of Kramida et al.^64^ is depicted in Figure 1; analogous plots for the PW92 and r^2^SCAN functionals are included in the Supporting Information.

The values obtained from Janak’s theorem are consistently and significantly lower than the experimental values, and we will therefore not discuss them further.

The ΔSCF formalism produces ionization energies in good agreement with the experiment. As expected, the results of the spin-polarized calculations are in better agreement with the experiment than those from the spin-restricted calculations. Notable differences can already be observed for the H and He atoms. Spin-polarized calculations also faithfully reproduce the dip in the ionization energy beyond half-filling of the p shell, while spin-restricted calculations predict that the ionization energy increases monotonically with the filling of the p shell.

### 4.2 Ionization Radii

#### 4.2.1 ΔSCF

As discussed in Section 2.3, Δ*S*CF ionization radii can be computed in two ways. In the first method, we fix the configurations to those of the ground state of the neutral atoms and their cations, respectively. In the second method, we relax the configurations of the atoms and cations to the lowest configurations at each radius. The resulting ionization radii for the H–Xe atoms obtained with the PBE functional are shown in Table 1. The inset shows the predictions of the second method, when they differ from those of the fixed configuration approach.

**Table 1:**
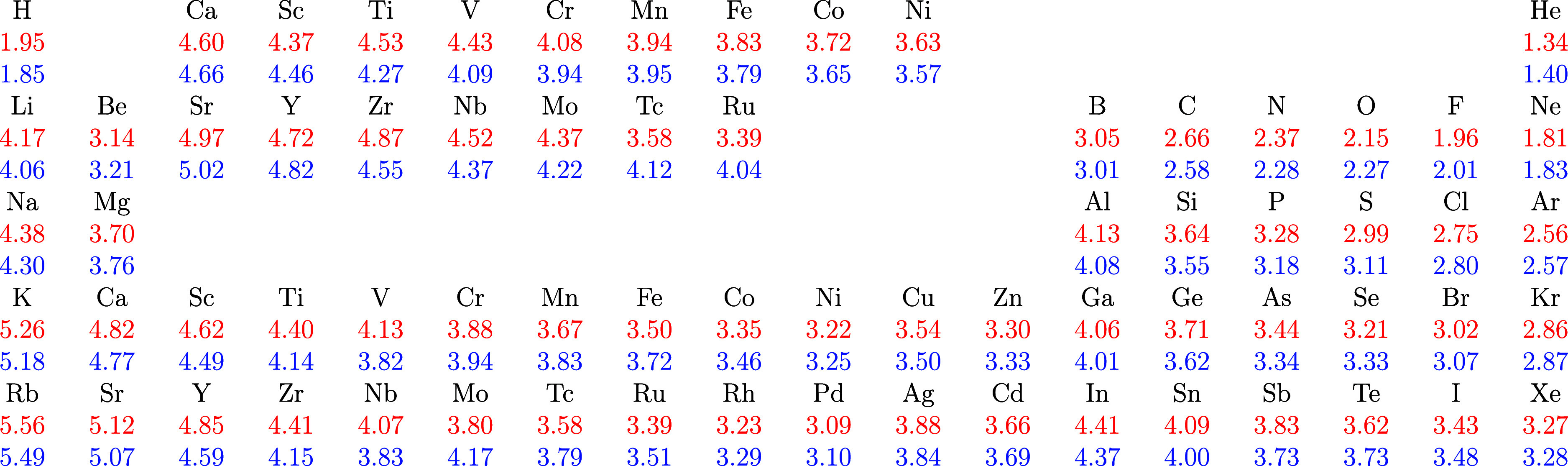
Ionization Radii *R* in *a*_0_ for the H–Xe Atoms from Spin-Restricted (in Red) and Spin-Polarized (in Blue) Nonrelativistic Δ*S*CF Calculations with Fractional Occupations and the PBE Functional with Configurations Fixed to the Ground States of the Unconfined Neutral Atom and Its Cation^a^

a Values obtained while considering the *R* dependence of the ground state of the neutral atom and its cation are highlighted in the inset for the elements where the value differs from the static configurations.

The ionization radius for the hydrogen atom is an interesting place to start the analysis. The corresponding exact model is analytically solvable: Sommerfeld and Welker^65^ obtained the ionization radius 1.8352*a*_0_ in their pioneering study. The exact ground state of hydrogen is fully spin-polarized and our spin-polarized PW92, PBE, and r^2^SCAN values of 1.90*a*_0_, 1.86*a*_0_, and 1.84*a*_0_ deviate from the exact value by only 4.5%, 1.4%, and 0.3%, respectively. The spin-restricted values of 1.99*a*_0_, 1.95*a*_0_, and 1.96*a*_0_ exhibit much larger deviations of 8.4%, 6.3%, and 6.8%, respectively, but are still remarkably accurate considering that our spin-restricted calculations are for an atom with 1/2 spin-up and 1/2 spin-down electrons.

Moving on to heavier elements, the comparison of the results from the fixed electron configuration and relaxed configuration methods proves to be interesting. Although inclusion of the relaxation effects does not appear to be important for most of the studied elements, large differences are observed in the pre-d and d blocks of the periodic table (Ca–Ni and Sr–Ru).

Since relaxing the configuration can result in a considerable decrease in the energy of the neutral atom, the second method is naively expected to yield ionization radii that are considerably smaller than the radii obtained with the first method of examining fixed electron configurations for the neutral atom and its cation. As expected, allowing the configuration to relax results in significant decreases in the ionization radii for most transition metals: for instance, the ionization radius of Mo decreases by 13% in the spin-restricted calculations when changes to the ground state of Mo and Mo^+^ are considered (the spin-polarized change is just −1.4%). Similarly, the spin-polarized calculation on Ru leads to an ionization radius that is 13% smaller when the electron configuration is allowed to relax (no change is seen in the spin-restricted calculation).

**Figure 2:**
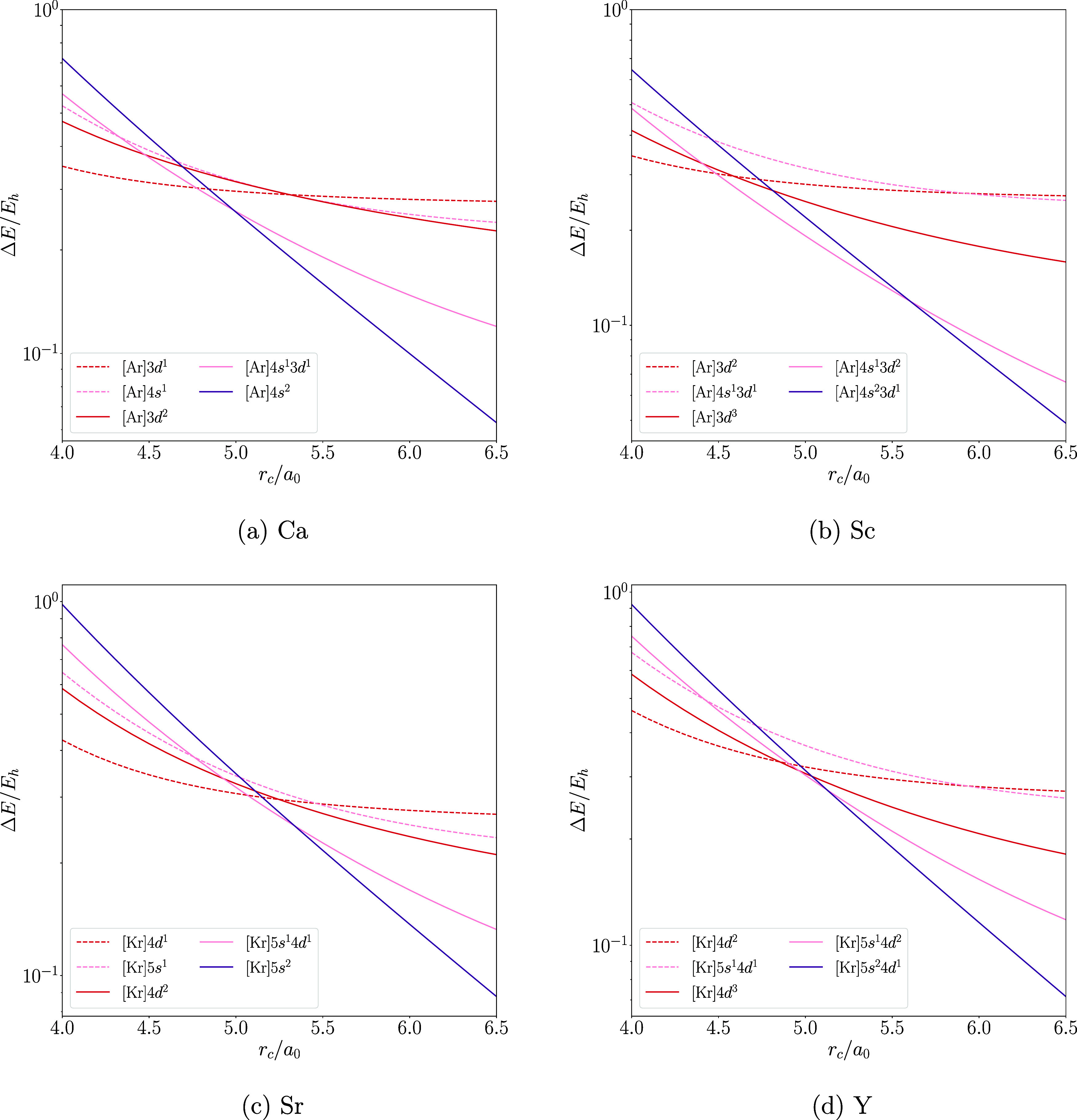
Ground-state crossings for the spin-polarized Ca, Sc, and Sr atoms and the spin-restricted Y atom in the hard-wall potential with the PBE functional. Configurations for the neutral atoms shown as solid lines and those corresponding to the monocation as dashed lines.

However, significant changes in energy upon relaxing the configuration may also occur for the cation, in which case the ionization radius can increase. Interestingly, such increases when the ground states are relaxed *are* observed for the Ca, Sc, and Sr atoms in both the spin-restricted and spin-polarized cases, as well as the Y atom in the spin-restricted case. The corresponding cations exhibit a ground-state crossing that moves the crossover point of eq 8 down in energy and thereby the ionization radius to the right; see Figure 2 for an illustration of these four exceptional cases.

Because the various configurations’ energies span several orders of magnitude under the studied range of confinement radii, we compare the energy of the confined atom with electron configuration *i* to that of the ground-state configuration of the unconfined atom
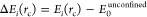
14where *i* is the state in question in all plots in this study.

The differences observed in ionization radii with and without relaxing the electron configuration underline the importance of considering all possible low-lying configurations as a function of confinement. Many transition metal atoms and their monocations exhibit ground-state crossings already in weak confinement due to the low-lying s–d excitations in these systems (see Section 4.4).

The spin-polarized and spin-restricted ionization radii differ for all atoms, which is not surprising given that either the neutral atom or its cation is going to be open-shell. Interestingly, the differences between these two approaches are the smallest for the noble gas atoms, while alkali metals show much larger differences between spin-polarized and spin-unpolarized calculations. As the spin-polarized calculations gave ionization energies in best agreement with the experiment in Section 4.1, the spin-polarized ionization radii should be the most accurate.

**Figure 3:**
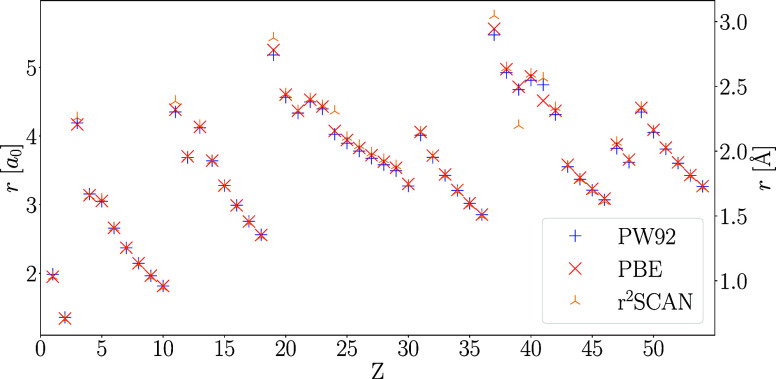
Ionization radii for the H–Xe atoms with the PW92, PBE, and r^2^SCAN functionals obtained from spin-polarized densities via ΔSCF with relaxed configurations.

**Figure 4:**
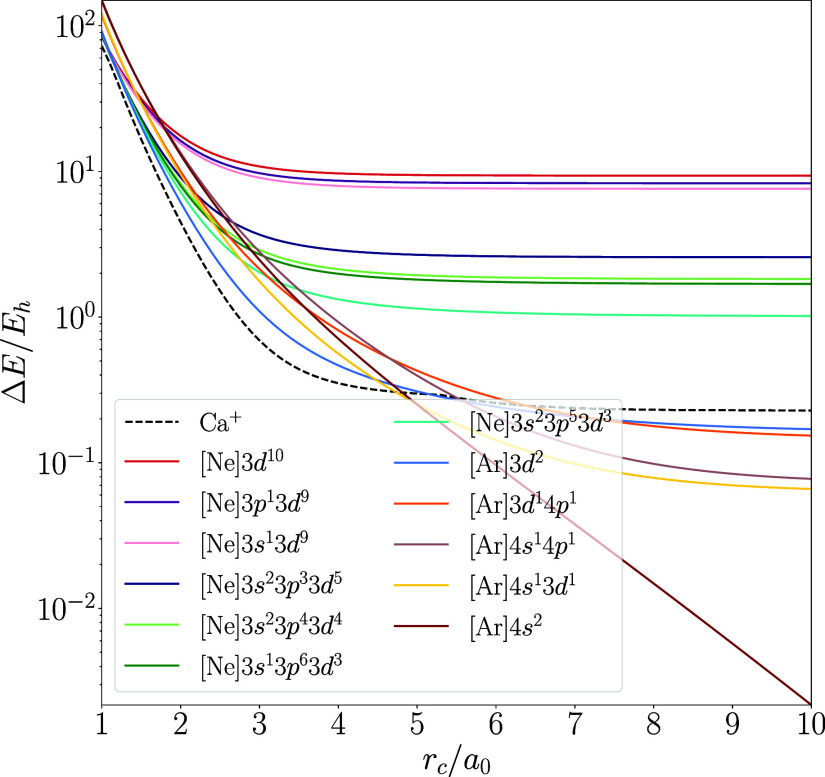
Energies of low-lying configurations of hard-wall confined spin-polarized Ca computed with PW92 shown as the energy difference from unconfined Ca as a function of the confinement radius. Note the semilogarithmic scale.

**Figure 5:**
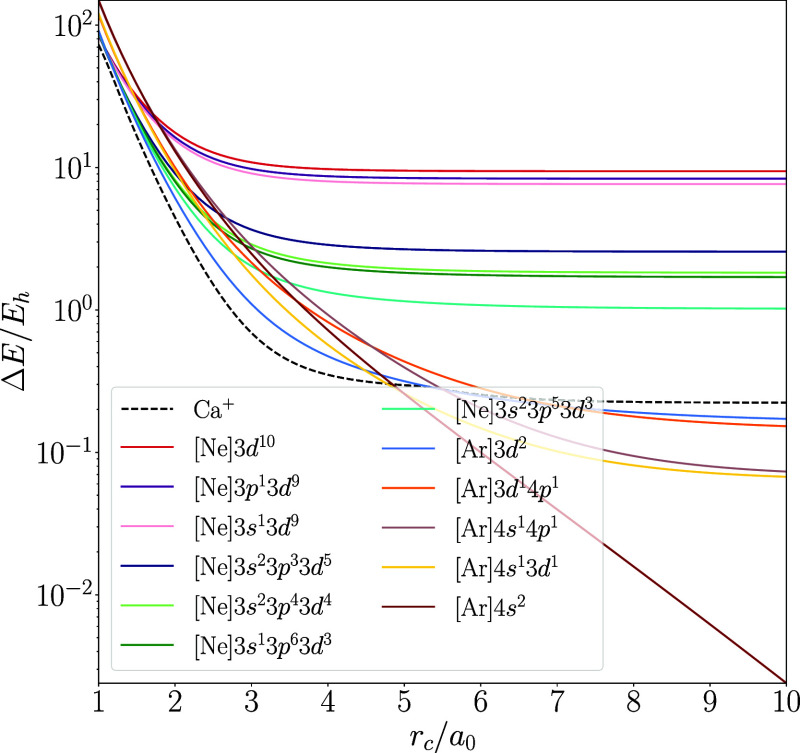
Energies of low-lying configurations of hard-wall confined spin-polarized Ca computed with PBE shown as the energy difference from unconfined Ca as a function of the confinement radius. Note the semilogarithmic scale.

**Figure 6:**
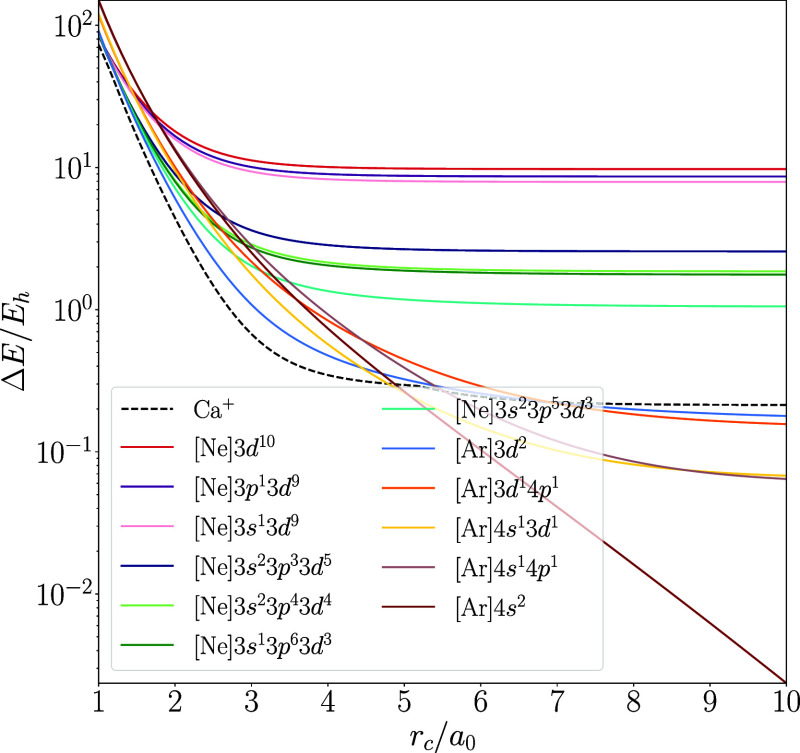
Energies of low-lying configurations of hard-wall confined spin-polarized Ca computed with r^2^SCAN shown as the energy difference from unconfined Ca as a function of the confinement radius. Note the semilogarithmic scale.

A comparison of the ionization radii obtained with the PW92, PBE, and r^2^SCAN functionals with spin-polarized densities and relaxed configurations is shown in Figure 3. Except for the alkali metals as well as the Cr, Y, and Nb atoms, the dissimilar density functional approximations are in excellent agreement. We further validate this agreement by qualitatively studying the functional dependence of the behavior of all low-lying configurations of the Ca atom in Figures 4–6, while the graphical representation of the results of the other atoms is included in the Supporting Information. We chose to feature Ca here due to its interesting chemistry, which will be discussed below in Section 4.4. The only major difference between the functionals is that r^2^SCAN predicts the 4s^1^3d^1^ configuration to be lower in energy than the 4s^1^4p^1^ configuration in the unconfined atom, while PW92 and PBE predict the opposite.

Despite such minor differences in the ordering of some states or relative energy differences between states, the qualitative behaviors obtained with the three different density functional approximations agree well with each other; the changes in the electron configurations are practically independent of the functional. This suggests that the phenomena found with the methodology employed in this work are not artifacts of the used level of theory but true physical phenomena within the studied model of confinement.

#### 4.2.2 Janak’s Theorem

We report ionization radii obtained from calculations using Janak’s theorem as tables in the Supporting Information, as we already found above in Section 4.1 that these calculations failed to reproduce ionization energies in a reliable manner.

Sen et al.^15^ computed atomic ionization radii with Janak’s theorem for the He–Ne atoms with exchange-only calculations based on Becke’s 1988 generalized-gradient functional.^66^ In contrast to our work, Sen et al.^15^ employed the transition state approximation (TSA) of Slater et al.^48^ in their calculations; the orbital energies were thus determined in calculations where half an electron was removed from the highest-lying orbital. Sen et al.^15^ compared this method to ΔSCF calculations and found that the two methods yield ionization radii in close agreement to each other. Despite the differences between our approaches, we will see below that our results are in good agreement with those of Sen et al.^15^

#### 4.2.3 Comparison to Literature Radii

**Figure 7:**
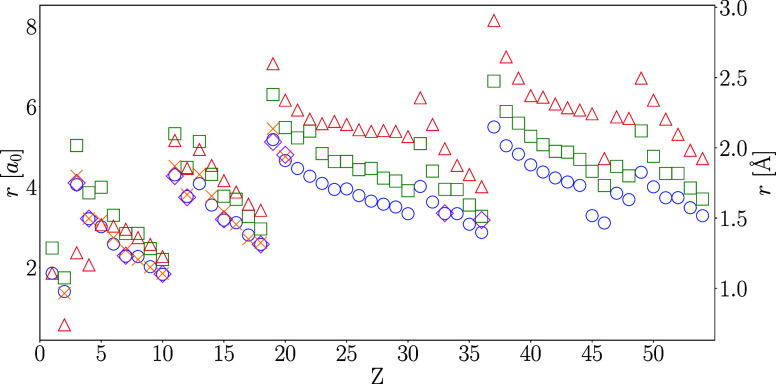
Comparison of ionization radii computed in this work with spin-polarized densities and the PBE density functional via ΔSCF (blue circles) or Janak’s theorem (green squares) against the Hartree–Fock values of Boeyens^5^ (red triangles) and DFT values of Sen et al.^15^ (magenta diamonds) and Garza et al.^16^ (orange crosses).

Numerically reliable ionization radii have been previously published by Boeyens,^5^ Sen et al.,^15^ and Garza et al.^16^ We compare our ionization radii from calculations based on the Δ*S*CF method (Section 4.2.1) and Janak’s theorem (Section 4.2.2) with relaxed configurations and the PBE functional against the earlier calculations in Figure 7. Analogous plots for the PW92 and r^2^SCAN functionals can be found in Supporting Information. We note that the dip in energy for the group 16 elements, which was found in the ionization energies in Section 4.1, is also visible in the ionization radii in Figure 7.

We first note that the analysis of Boeyens^5^ was based on nonrelativistic Hartree–Fock calculations for 1 ≤ *Z* ≤ 102, and *r*_+_ was identified via Koopmans’ theorem^46^ by finding the zero of the orbital energy of the outermost valence electron shell. However, as discussed in Section 1, Boeyens^5^ did not employ a proper hard-wall potential and did not consider the changing nature of the ground-state configuration as a function of confinement. The values of Boeyens^5^ are systematically and considerably larger than the results of any of the other calculations for *Z* ≥ 15. These surprisingly large differences are likely explained by the lack of electron correlation in Hartree–Fock theory, which is known to lead to overestimation of atoms’ size compared to high-level wave function calculations, with DFT yielding close agreement with accurate theoretical reference values.^67^ The closeness of DFT densities with multideterminantal densities has also been noted on previously by Ortiz-Henarejos and San-Fabián,^68^ for example.

Our calculations based on Janak’s theorem without the TSA systematically predict larger ionization radii than our ΔSCF calculations, but they are still much smaller than the Hartree–Fock values of Boeyens.^5^ Our Δ*S*CF calculations are in excellent agreement with the limited data reported by Sen et al.^15^ in the spin-restricted formalism, who also found good agreement between their TSA method and Δ*S*CF calculations in their work. Interestingly, our spin-polarized data appears to be in better agreement with the calculations of Sen et al.^15^ than our spin-restricted calculations, as the additional comparisons included in the Supporting Information show, even though the calculations of Sen et al.^15^ were spin-restricted. As was discussed in Section 4.2.2, Sen et al.^15^ did not include correlation in their calculations, which likely explains the differences between our data.

Our ΔSCF calculations are also in good agreement for the 9 atoms studied by Garza et al.^16^ with spin-polarized Δ*S*CF calculations with the PW92 functional.

Since our ΔSCF calculations were found to produce ionization potentials of unconfined atoms in good agreement with experimental values (Section 4.1) and also yield ionization radii in good agreement between dissimilar density functional approximations as well as with previous literature, we are confident that our calculations offer thus a reliable basis for studies of confined atoms and a reliable ground for further studies with elaborate wave function methods.

### 4.3 Estimates for Atomic Radii

Before proceeding to the in-depth analysis of the changes of atoms’ electronic structure in confinement, we return to the question of the atomic radii discussed above in Section 2.4. Because the present study involves several configurations for each atom, many not being bound with respect to ionization in the unconfined atom, it is useful to be able to estimate the resulting atoms’ size. While the covalent size estimate of Slater^51^ in eq 9 is parameter-free, the two vdW radius estimates of eqs 10 and 11 feature a threshold parameter.

Bader et al.^52^ employed the threshold 0.002 electrons/bohr^3^ in eq 10, while Boyd^69^ showed that the criterion 0.001 electrons/bohr^3^ reproduces the same relative radii of atoms as those obtained with an even smaller value for the threshold. Rahm et al. also employed the threshold 0.001 electrons/bohr^3^ in their study of atomic and ionic radii,^70,71^ and we chose to adopt this value of the threshold, ε = 10^–3^ in eq 10, as well. We note that Smith et al.^72^ have also used eq 10 recently to estimate vdW radii, opting to use a threshold of 0.0015 electrons/bohr^3^ instead.

We discovered an issue in our analysis with the *r*_ρ_ estimate of eq 10 for the vdW radius for the 3d^1^ state of the H atom. As only s-type orbitals can have a finite value at the nucleus^54^ and as the 3d orbital in question turns out to be extremely diffuse in character, the density criterion eq 10 is not satisfied with any radius. As a result, *r*_ρ_ is not defined for the employed value of ε.

In addition, *r*_ρ_ for the 1s^1^3d^1^ configuration of He turns out to be smaller (*r*_ρ_ = 2.10*a*_0_ for spin-unrestricted PBE) than that for the 1s^1^2p^1^ (*r*_ρ_ = 4.05*a*_0_) or 1s^2^ (*r*_ρ_ = 2.68*a*_0_) configurations, even though basic physical insight would say that the 1s^2^ ground-state configuration has to be more compact than those where an electron is excited from the 1s shell to the 2p shell, not to mention to the 3d shell.

**Figure 8:**
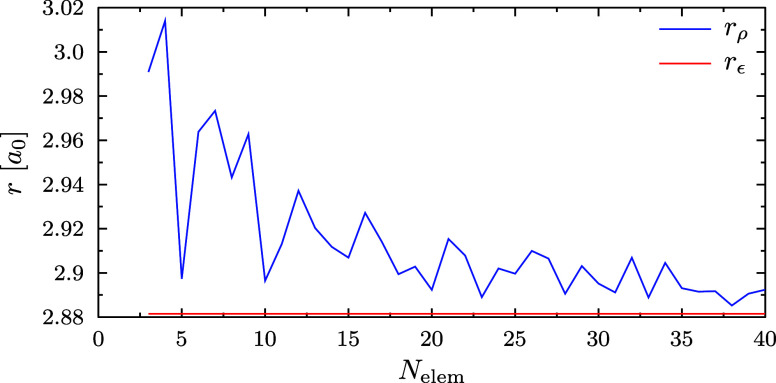
Dependence of the radial estimates *r*_ρ_ and *r*_ε_ defined by eqs 10 and 11, respectively, on the number of radial elements *N*_elem_ used in the discretization. The calculations are performed for the ground state of the hydrogen atom, using unrestricted Hartree–Fock theory, and the error in the total energy compared to the analytical value −0.5*E*_*h*_ is smaller than 10^–7^*E*_*h*_ in all calculations shown in the figure. The estimates from eq 11 agree with the analytical value *r*_ε_ ≈ 2.8815126965663684390*a*_0_ to a precision better than one part per million.

We also find that the criterion of eq 10 is overly sensitive to the numerical discretization: a finite element calculation for the exact ground state of the unconfined hydrogen atom reveals a significant sensitivity to numerical noise, as shown in Figure 8. We find it highly likely that similar issues are also present in the Gaussian-basis calculations of refs (70−72) that relied on the estimate of eq 10: small changes to the Gaussian basis likely result in considerable changes in the estimated radius, since the calculations of Figure 8 which all reproduce the exact ground-state energy −1/2*E*_*h*_ to sub-μ*E*_*h*_ accuracy exhibit considerable differences in the estimate of the vdW radius.

Note that the ANO-RCC basis set employed in refs (70 and 71) produces an analogous Hartree–Fock energy of −0.49998374*E*_*h*_ (basis set obtained from the Basis Set Exchange^73^ and calculation performed with ERKALE^74^) and thus exhibits a much larger truncation error of 16.3 μ*E*_*h*_ for the unconfined hydrogen atom. Smith et al.^72^ examined various Gaussian basis sets, and variations in the order of 0.01*a*_0_ can be observed in their data as well.

To choose the criterion ε in eq 11, we match the two estimates of the vdW radius using the well-known analytical expression for the ground state of the hydrogen atom (*R*_1s_(*r*) = 2*e*^–*r*^, *f*_1s_ = 1). Performing the matching using 20-digit precision in Maple 2024, the electron density is found to be 10^–3^ electrons/bohr^3^ at *r*_ε_ ≈ 2.8815126965663684390*a*_0_ ≈ 1.524 Å, and evaluating eq 11 yields the threshold ε ≈ 0.073416683704840394115 electrons for use in determining the radius from eq 11.

The data in Figure 8 show that the proposed *r*_ε_ estimate of eq 11 is numerically well-behaved, yielding excellent agreement with the radius determined from the analytical solution, thus successfully addressing the issues with undefined vdW radii and numerical stability in the *r*_ρ_ estimate of eq 10. This criterion also easily resolves the issue with the configurations of He, agreeing with physical insight that the 1s^2^ ground state (*r*_ε_ = 2.26*a*_0_ for spin-unrestricted PBE) is more compact than the 1s^1^2p^1^ (*r*_ε_ = 8.07*a*_0_) or 1s^1^3d^1^ (*r*_ε_ = 15.48*a*_0_) excited states, the latter of which also show a significant difference in diffuse character.

The *r*_ϵ_ estimates for the states of He are also in excellent qualitative agreement with the covalent radius estimates *r*_max_ of eq 9: both *r*_ε_ and *r*_max_ predict the size order 1s^2^ < 2p^2^ < 1s^1^2p^1^ < 1s^1^3d^1^, while the *r*_ρ_ estimate of eq 10 predicts a thoroughly dissimilar order 1s^1^3d^1^ < 1s^2^ < 1s^1^2p^1^ < 2p^2^.

We note again here that the 1s^1^3d^1^ excited state is so diffuse in the unconfined He atom that it is likely not converged to the CBS limit in our calculations, as we chose not to converge the value of the practical infinity *r*_∞_ given that excited states of the unconfined atom are not the focus of this work.

### 4.4 Observed Changes in the Electronic Structure

In the following, we will discuss the key observations from the systematic analysis of all of the atoms H–Xe. We focus the discussion in the main text on the PBE functional due to its insurmountable popularity in the literature but note that the behaviors of various atoms are qualitatively independent of the functional, as was already demonstrated for the Ca atom in Section 4.2. Thorough analyses for all functionals are available in the Supporting Information.

In addition to energies of the configurations as a function of confinement, we also report the estimated atomic radii for the configurations of the unconfined atoms with the methodology discussed in Sections 2.4 and 4.3 in the Supporting Information. We note again the limitations of the previously used *r*_ρ_ estimate of eq 10 that was discussed above in Section 4.3, in that the reported *r*_ρ_ values exhibit numerical noise. Furthermore, the excitation energies of each configuration relative to the ground state are also included in the Supporting Information for the case of the unconfined atom.

We note here that we were unable to converge the calculations of some configurations with the r^2^SCAN functional. We tentatively attribute this to the numerical ill-behavedness of the functional^75^ and to the slow convergence of *meta*-GGA functionals in general with respect to the number of radial elements.^39^ We have excluded all nonconverged calculations from our analysis.

The in-depth analysis of the data included in the Supporting Information led us to the following observations on the behavior of the H–Xe atoms, which can be grouped into the following classification based on the atom’s position in the periodic table.

#### 4.4.1 H–He

Similarly to previous studies, we do not observe ground-state crossings for the H or He atoms in the studied range of confinement radii. The 1s orbital remains occupied over the whole domain of the studied confinement radii. Interestingly, both atoms have configurations with an occupied 3d orbital that are stable with respect to ionization in the unconfined atom (the configuration has a lower energy than that of the cation’s ground state), even though the 3d orbital is extremely diffuse as *r*_ε_ ≈ 15*a*_0_ for the corresponding configuration.

#### 4.4.2 Li–O

The valence electrons of the Li–O atoms occupy the 2s orbital in the unconfined atom. Contrary to the H and He atoms, we observe ground-state crossings where the 2s electrons hop to the 2p orbital as the confinement radius is decreased; for the Li atom, this happens already at *r*_c_ = 3*a*_0_, but the location of the crossover point *r*_c_ decreases in increasing charge and occurs for the O atom at *r*_c_ = *a*_0_.

The electron shift for Li was also predicted by Rahm et al.,^22^ while the 2s^2^ → 2p^2^ transition of the C atom was reported by Pašteka et al.^19^ As far as we are aware, the analogous transitions for the Be, B, N, and O atoms have not been discussed in previous literature.

We further note that the Li and O atoms have configurations with an occupied 3d orbital that are stable with respect to ionization in the unconfined atom, although the 3d orbital is again extremely diffuse as *r*_ε_ ≈ 15*a*_0_, as in the case of H and He.

#### 4.4.3 F

The valence electrons in the F atom occupy the 2s and 2p orbitals. One of the 2s electrons hops to the 2p orbital at *r*_c_ = *a*_0_, which represents extreme confinement, thus fully filling the 2p orbital. This electron transition does not appear to have been previously reported in the literature. Similarly to Li and O, F has a configuration with an occupied 3d orbital already in the unconfined atom, but the orbital is equally diffuse.

#### 4.4.4 Ne

Confirming previous studies, the Ne atom does not exhibit ground-state crossings in the studied range of confinement radii. We note that Ne also has a configuration with an occupied 3d orbital, which is stable with respect to ionization in the unconfined atom.

#### 4.4.5 Na–Mg

The valence electrons of Na and Mg occupy the 3s orbital, which becomes unfavorable in confinement, like the 2s orbital in Li and Be. While in Li and Be the electrons hopped from 2s to 2p, the 3s electrons of Na and Mg hop to the 3d orbital around *r*_c_ ≈ 2*a*_0_, instead, even though the 3d orbital has a larger extent than the 3p orbital in the unconfined atom, and the 3d orbital is not a valence orbital even in the Mg atom without confinement.^76^ These electron transitions, which occur at relatively strong confinement, have not been previously reported in the literature, to our knowledge.

We observe that the [Ne]4f^1^ configuration of the Na atom is stable with respect to ionization in the unconfined atom and flips below the initial ground state in extreme confinement (close to *r*_c_ = *a*_0_). The 4f orbital is highly diffuse in the unconfined atom (*r*_ε_ ≈ 24*a*_0_). Also the state [Ne]3d^1^4f^1^ of Mg flips below the initial ground state close to *r*_c_ = *a*_0_.

#### 4.4.6 Al–Ar

The valence electrons of the unconfined Al–Ar atoms occupy the 3s and 3p orbitals. No electron shifts were reported in previous studies for these atoms. However, we see that the 3p orbital becomes unfavorable in confinement, gradually transferring all electrons to the 3d orbital when the confinement radius is decreased, the transition occurring between *r*_c_ = 2*a*_0_ and *r*_c_ = 1.5*a*_0_ (strong confinement). As the confinement radius is further decreased, both of the 3s electrons follow. The behavior is thus different from that observed above for Li–Ne, likely because the 3d orbital is too high to be accessible in the lighter atoms.

#### 4.4.7 K–Ca

Confirming the results of previous studies,^9,17,22^ analogously to Na and Mg, the 4s valence electrons of the K and Ca atoms hop to the 3d orbital around *r*_c_ ≈ 4.5*a*_0_. The 3d orbital is thus already easily accessible, and its relevance in chemistry has been well established in the literature:^76−82^ Ca is sometimes referred to in the literature as an “honorary transition metal”^82^ due to its covalent binding like a transition metal,^76,78,79,81^ following similar work performed earlier on the heavier analogous Cs and Ba that are not considered in this work.^83^

Further novel ground-state crossings happen close to *r*_c_ = *a*_0_ (extreme confinement), where the [Ar] core configuration is opened up and all the 3s and 3p electrons also move to the 3d orbital. We observe that both atoms have a configuration with an occupied 3d orbital that is bound relative to ionization in the unconfined atom.

#### 4.4.8 Sc–Ni

The 3d transition metals feature strong competition between the 4s and 3d orbitals, which can be observed from the small excitation energies in the unconfined atom. The states [Ar]3d^*n*^, *n* ≤ 10 are stable with respect to ionization already in the unconfined atom, which explains why ground-state crossings happen already in weak confinement.

Confirming previous findings,^9,22^ the 4s electrons shift to the 3d orbital around *r*_c_ = 4*a*_0_ in the Sc–Ni atoms, similarly to the observations made above for K and Ca. However, here we observe that the Sc and Ti atoms exhibit additional ground-state crossings in extreme confinement (between *r*_c_ = *a*_0_ and *r*_c_ = 2*a*_0_), where some 3s and 3p electrons also shift to the 3d orbital.

#### 4.4.9 Cu–Zn

The unconfined Cu and Zn atoms already have a fully filled 3d orbital in their ground states, thus the 4s → 3d shift observed earlier in the 3d transition metal series cannot happen for these atoms. The 4s electrons hop to the 4f orbital in extreme confinement (around *r*_c_ = 1.5*a*_0_), which does not appear to have been previously reported in the literature.

#### 4.4.10 Ga–Kr

The ground-state configurations of the unconfined Ga–Kr atoms have filled 4s and 3d orbitals and a variably filled 4p orbital. The 4s and 4p electrons hop to the 4f orbital as the confinement radius is decreased (between *r*_c_ = 1.5*a*_0_ and *r*_c_ = *a*_0_), leading to a [Ar]3d^10^4f^*n*^ ground state in extreme confinement. The favoring of the 4f orbital over the 4s and 4p orbitals in the confined Kr atom has been previously reported by Garza et al.^16^

#### 4.4.11 Rb–Sr

Like the alkali and earth alkali atoms in the previous period, the valence 5s electrons of the Rb and Sr atoms hop to the 4d orbital already around *r*_c_ = 5*a*_0_, confirming the previous observations of Guerra et al.^17^ and Rahm et al.^22^ However, unlike the lighter analogues K and Ca, we also see a 4d → 4f shift when the confinement radius is further decreased to *r*_c_ ≈ 1.2*a*_0_, which represents extreme confinement. The 4s and 4p electrons also shift to the 4f orbital below *r*_c_ = 1.2*a*_0_, which, to the best of our knowledge, has not been reported in the literature so far.

#### 4.4.12 Y–Rh

The valence electrons of the unconfined Y–Rh atoms occupy the 5s and 4d orbitals in their ground states. The 5s valence orbital becomes unfavorable in confinement, and the electrons hop to the 4d orbital at confinement radii varying from *r*_c_ = 6.1*a*_0_ to *r*_c_ = 4.2*a*_0_, that is, already in weak confinement, in agreement with previous literature.^9,22^ Again, like for Cu–Sr, we observe further electron shifts to the 4f orbital, and the 4d orbital is partially depopulated after the 5s orbital in confinement radii below *r*_c_ = 1.5*a*_0_, which already represents extreme confinement. Y and Zr shift electrons from the 4p core orbital to the 4f orbital close to *r*_c_ = *a*_0_. These additional electron shifts do not appear to have been reported in previous studies.

#### 4.4.13 Pd

It is well-known that the unconfined Pd atom has a particularly stable ground state ([Kr]4d^10^).^9,22^ We observe no ground-state crossings for Pd in the studied range of confinement radii.

#### 4.4.14 Ag–Cd

Ag and Cd already have a filled 4d orbital, while we observe previously unexplored electron shifts where 5s electrons hop to the 4f orbital around *r*_c_ = 2*a*_0_.

#### 4.4.15 In–Xe

The In–Xe atoms have filled 5s and 4d orbitals and a 5p orbital with variable filling in the unconfined atom. We observe that the 5p electrons start to gradually shift to the 4f orbital around *r*_c_ = 2*a*_0_, after which both of the 5s electrons follow analogously to Ga–Kr, which does not appear to have been reported in the literature before now.

## 5 Summary and Conclusions

To the best of our knowledge, a robust and systematic study of atoms in confinement has not been previously published in the literature. Such a study needs to both use a suitable numerical method and consider the changing nature of the ground state as a function of the confinement. We did not find a single publication that satisfies both of these criteria while also considering an appreciable portion of the periodic table instead of just some specific subrows or groups.

We therefore took a fully numerical approach based on the FEM to study atoms in hard-wall confinement. We chose to represent the atoms with spherically averaged, spin-restricted, or spin-polarized densities and carried out calculations at three levels of density functional approximations: the local density approximation employing the Perdew–Wang (PW92) correlation functional, the GGA employing the Perdew–Burke–Ernzerhof (PBE) exchange–correlation functional, and the *meta*-GGA approximation employing the r^2^SCAN exchange–correlation functional. We compared the ionization energies of unconfined atoms predicted by spin-restricted or spin-polarized calculations to experiment and unsurprisingly observed the latter to be more accurate, while even the spin-restricted calculations succeed in predicting periodic trends.

We demonstrated the significance of considering confinement-induced changes in the electronic structure by comparing ionization radii computed for fixed electron configurations to those obtained in calculations in which the electron configuration can relax as a function of the confinement radius. Depending on the atom, this relaxation can result in either an increase or a decrease of the ionization radius due to competing effects in the neutral atom and its cation, and the largest differences are seen for the pre-d and early d elements.

As a side effect of our study, we also examined various atomic size estimates. We found severe issues in the vdW radius estimate proposed by Bader et al.:^52^ it is not always defined, it has severe issues with numerical stability even with huge numerical basis sets, and its predictions do not agree with basic physical insights for the 1s^2^ ground and 1s^1^2p^1^ and 1s^1^3d^1^ excited configurations of the He atom. We proposed a related estimate which is always defined, does not suffer from severe numerical problems like the aforementioned estimate, and also predicts the aforementioned states of the He atom to be increasingly diffuse in character, in agreement with the covalent radius estimate of Slater.^51^ We expect our proposed vdW radius estimate to be useful in future work.

We then carried out a systematic study of the total energies of the ground and low-lying excited states of the H–Xe atoms as well as their monocations as a function of the confinement radius *r*_c_, paying special focus on how the ground state evolves as a function of confinement. We have confirmed electron shifts made in previous studies on confined atoms but also made novel observations. We summarize our analysis of the calculations as follows.

- Ground-state crossings occur for the majority of the elements.
- Valence s electrons are highly unfavored under strong confinement: already the Li and Be atoms show a 2s → 2p electron shift.
- Shifts from the ns and np to the 3d orbital are observed for the elements of the second and third periods.
- Shifts to the 4f orbital can be observed for Cu onward in strong confinement.

Importantly, we saw that the dissimilar density functional approximations were in excellent agreement in calculations of ionization radii and that the qualitative behavior of the confined atoms is similar for dissimilar density functionals. We are therefore confident that our results are not artifacts of the employed level of theory and that similar results would be obtained even with high-level *ab initio* calculations.

Like most studies of confined atoms, the present study was limited to the use of integer occupation numbers for the s, p, d, and f shells to maximize physical interpretability. However, as we discussed in Section 4.2.2, the use of nonintegral occupation numbers could be useful for transition metals, for instance,^38,48,49^ and we hope to revisit such calculations with state-of-the-art optimization algorithms such as optimal damping^84^ in future work.

Our study was limited to the H–Xe atoms because relativistic effects are well-known to be essential for heavy elements.^85,86^ Relativistic calculations of atoms in confinement should be feasible using the same approach, and such calculations might be carried out with the recent implementation of Čertík et al.,^87^ for example.

### 5.1 Connections to Basis Set Design

It is interesting to draw a parallel here to basis set design, since the application of confinement potentials in the construction of numerical atomic orbital (NAO) basis sets as originally proposed by Averill and Ellis^88^ was the original motivation of our work. While Averill and Ellis^88^ employed a finite potential barrier, Sankey and Niklewski^89^ proposed building NAO basis sets using a hard-wall potential to enforce the locality of the resulting basis set. In later works, Sankey and co-workers refer to the arising orbitals as “fireballs” due to their excited nature relative to the unconfined atom.^90,91^ The technique of Sankey and Niklewski^89^ has been later used by many other authors as well, such as Sánchez-Portal et al.,^92^ Basanta et al.,^93^ Kenny and Horsfield,^94^ and Nakata et al.^95^

The demands for various chemical elements’ AO basis sets for electronic structure calculations^96−98^ are inherently tied to the elements’ chemistry. Importantly, the relevance of distinct angular momentum *l* orbitals for atoms is expected to be a continuous function: as we move to heavier elements, we first see an increasing importance of p functions, followed by increasing importance of d functions, f functions, and so on. Unlike the occupied orbitals in a SCF calculation on an atom, the importance of polarization functions in a basis set is not a step function for which we can certainly say whether a specific element requires s, p, d, f, etc. functions or not.

This continuity is a challenge when predicting the relevance of distinct orbitals to chemical bonding. The advantage of our arguably simple model is that we are able to highlight these chemical characteristics of the studied atoms, that is, the relative importance of the electron configurations as a function of confinement. The physicality of the model is therefore obvious. For example, we saw in this work that the simple hard-wall confinement model predicts the importance of the 3d orbital in K and Ca, which is obvious from the large transition radius *r*_c_ ≈ 4.5*a*_0_.

In contrast, the 3d orbital is so high in the second-row 3p block that electron shifts to it occur only in strong confinement. Yet, it is by now well-known^99−104^ that tight d functions need to be included in the basis set for the second-row p block elements in polyatomic electronic structure calculations, as this inclusion typically results in significant increases of atomization energies; for example, the atomization energies of sulfur-containing molecules such as SO_2_ and SO_3_ typically increase by tens of kcal/mol upon the addition of 3d functions. Perchloric acid (HClO_4_) and dichlorine heptoxide (Cl_2_O_7_) represent even more extreme cases, with documented increases of the atomization energy by 50 and 100 kcal/mol, respectively.^105^

Martin^105^ found a physical interpretation for this effect: the 3d Rydberg orbital on the chlorine atom(s) can accept back-bonding contributions from the oxygen atoms. Mehta and Martin^106^ also point out that the 3d orbital comes down in energy when the atom becomes more positively charged. Indeed, AOs determined for cations are commonly used to introduce additional flexibility in NAO basis sets.^107^ Mehta and Martin^106^ also made the discovery that the fourth-row 5p block analogously exhibits a heightened importance of 4f Rydberg orbitals.

Based on the above findings, we expect that heavier elements will exhibit electron shifts to the 4f orbital in weaker and weaker confinement, since the 4f orbital will come down in energy as the nuclear charge is increased.

As hard-wall confinement leads to a discontinuous derivative at the boundary, most modern NAO methods employ soft confinement potentials to achieve orbitals that go smoothly to zero. We aim to present a thorough discussion of atoms in soft confinement in a follow-up study.

## Supporting Information Available

Raw energies for all calculations (ZIP)
Comparison of the ionization energies with the PW92 and r^2^SCAN functional; values of the ionization radii via Janak’s theorem for both the fixed and relaxed configuration approaches; detailed analysis of the evolution of the ground state of the H–Xe atoms and state crossings with the ground-state configuration of the unconfined atom; estimated atomic sizes and excitation energies relative to the ground state for the studied configurations in the unconfined atom; plots of the total energy of the low-lying states of the H–Xe atoms and their monocations as a function of the confinement radius; and all of the above sets of results both for the spin-restricted and spin-polarized formalisms for all the functionals considered in this work: PW92, PBE, and r^2^SCAN (PDF)
