# Supplementary material for: Systematic Study of Hard-Wall Confinement-Induced Effects on Atomic Electronic Structure
Source: J Phys Chem A. 2025 Jan 29;129(11):2791–805. doi: 10.1021/acs.jpca.4c05641 (PMC11931545; doi:10.1021/acs.jpca.4c05641)
Supplement: Supplementary file 2 — jp4c05641_si_002.pdf [file jp4c05641_si_002.pdf]

Supporting Information: Systematic Study of Confinement Induced Effects on Atomic Electronic Structure

Hugo Åström and Susi Lehtola*

University of Helsinki, Department of Chemistry, Faculty of Science, P.O. Box 55 (A.I. Virtanens plats 1), FI-00014 University of Helsinki, Finland

E-mail: susi.lehtola@alumni.helsinki.fi

1 Ionization energies

A comparison of experimental ionization energies to ionization energies calculated with the PW92 functional is shown in fig. S1. An analogous comparison of experimental values and values calculated with the r^{2}SCAN functional is shown in fig. S2.

2 Ionization radii

Ionization radii computed with the PW92 functional via ΔSCF are shown in table S1 and via Janak's theorem in table S2. Analogous values for the r^{2}SCAN functional are shown in table S3 and table S4 respectively. Ionization radii computed with the PBE functional via Janak's theorem are shown in table S5. A comparison to literature radii is shown for the PW92 functional in fig. S3 for spin-restricted densities and in fig. S4 for spin-polarized densities, for the r^{2}SCAN functional in fig. S5 for spin-restricted densities and in fig. S6 for spin-polarized densities, and for the PBE functional in fig. S7 for spin-restricted densities.

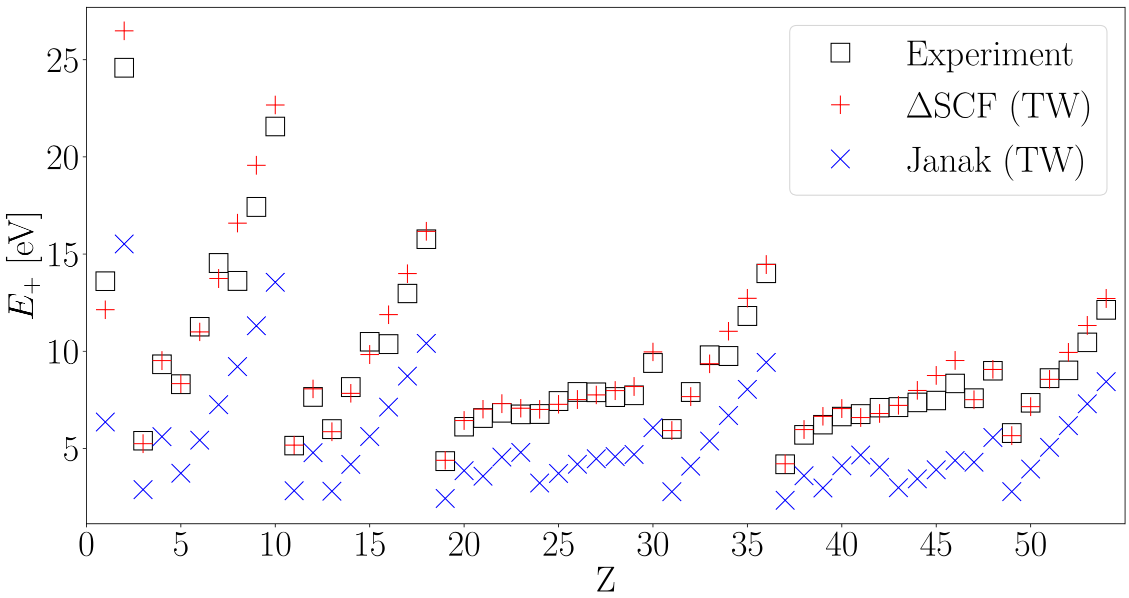

(a) Ionization energies of the H–Xe atoms obtained with the PW92 functional and spin-restricted densities.

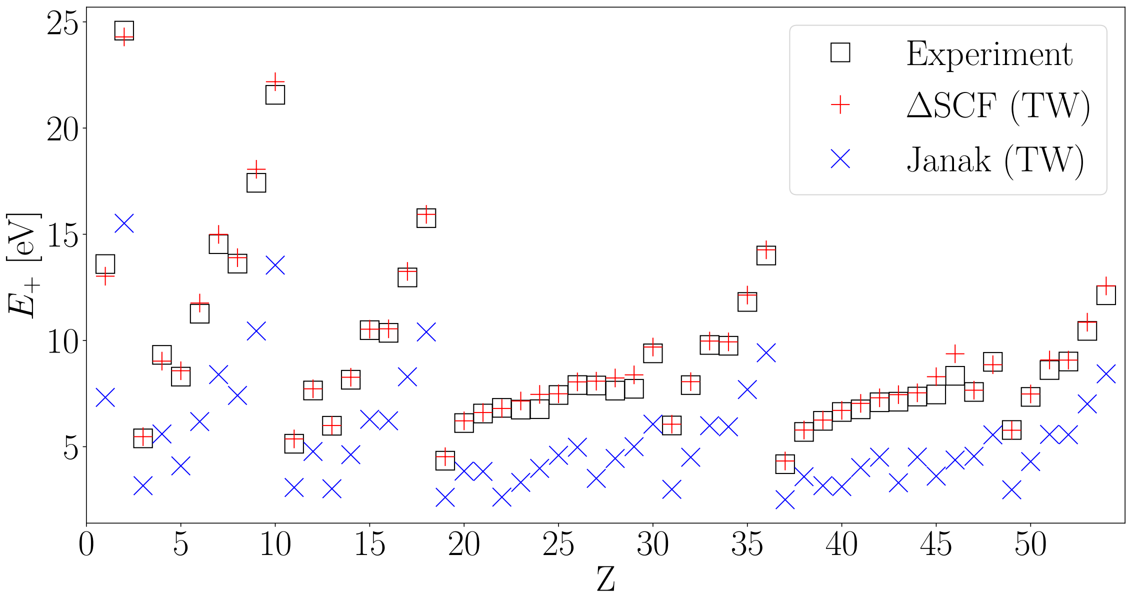

(b) Ionization energies of the H–Xe atoms obtained with the PW92 functional and spin-polarized densities.

Figure S1: Comparison of the ionization energies of unconfined atoms computed with spin-restricted (fig. S1a) and spin-polarized (fig. S1b) densities in this work (TW) with ΔSCF or Janak's theorem against experimental values.

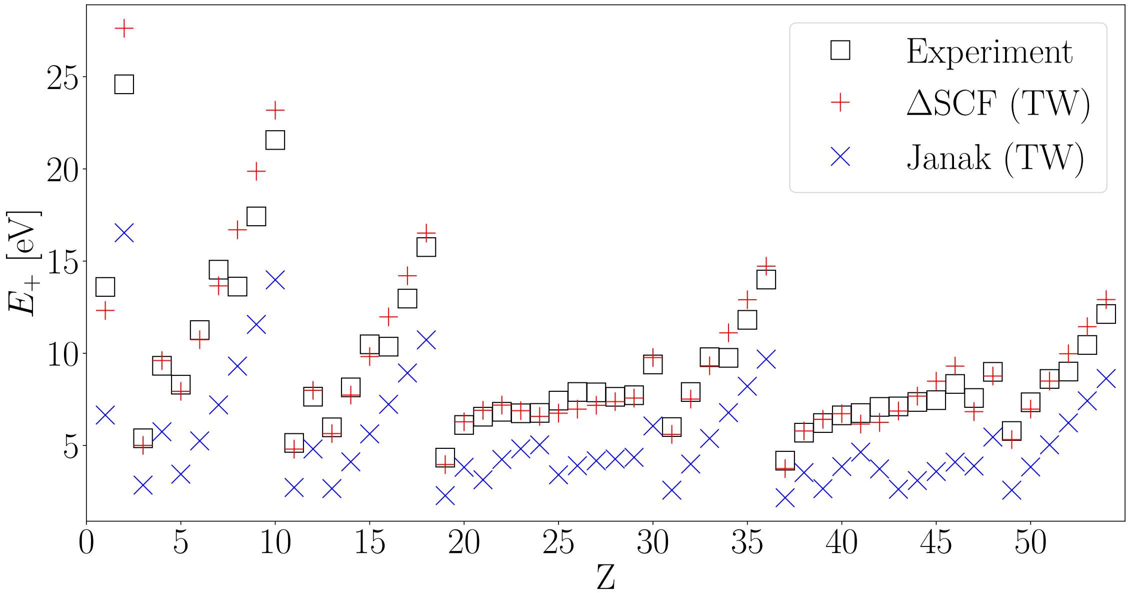

(a) Ionization energies of the H–Xe atoms obtained with the r^{2}SCAN functional and spin-restricted densities.

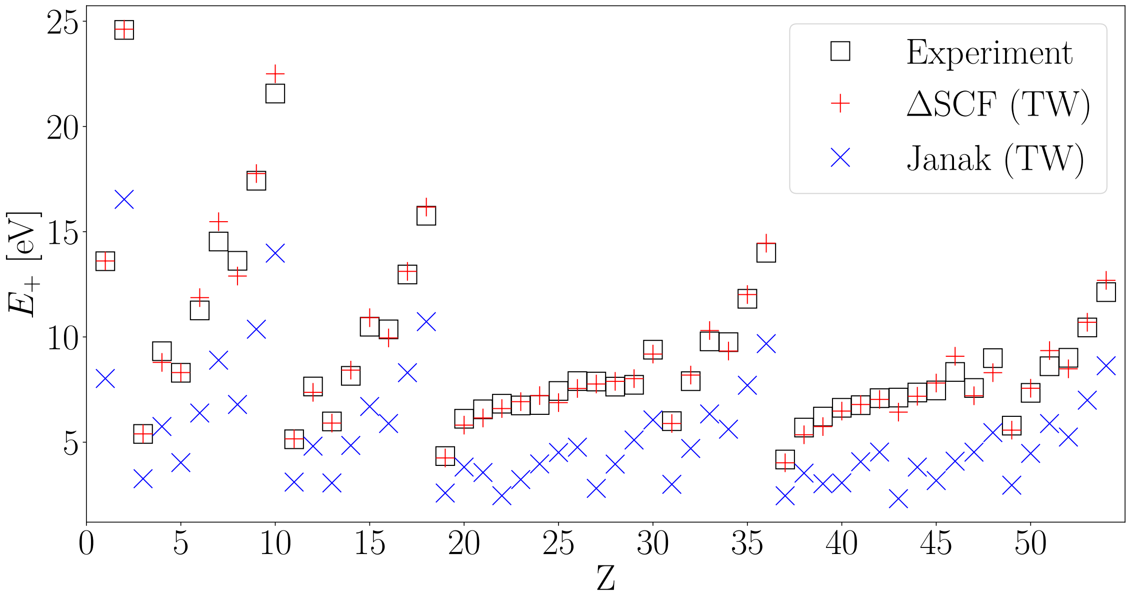

(b) Ionization energies of the H–Xe atoms obtained with the r^{2}SCAN functional and spin-polarized densities.

Figure S2: Comparison of the ionization energies of unconfined atoms computed with spin-restricted (fig. S2a) and spin-polarized (fig. S2b) densities in this work (TW) with ΔSCF or Janak's theorem against experimental values.

H		Ca	Sc	Ti	V	Cr	Mn	Fe	Co	Ni							He
1.98		4.57	4.34	4.50	4.40	4.03	3.90	3.78	3.68	3.58							1.36
1.90		4.62	4.42	4.23	4.06	3.91	3.90	3.74	3.62	3.54							1.41
Li	Be	Sr	Y	Zr	Nb	Mo	Tc	Ru				B	C	N	O	F	Ne
4.19	3.16	4.92	4.68	4.81	4.75	4.31	3.55	3.37				3.05	2.66	2.37	2.14	1.96	1.81
4.09	3.22	4.97	4.76	4.51	4.34	4.19	4.09	4.00				3.01	2.58	2.28	2.26	2.01	1.83
Na	Mg											Al	Si	P	S	Cl	Ar
4.35	3.69											4.12	3.64	3.28	2.99	2.76	2.56
4.27	3.74											4.08	3.56	3.19	3.10	2.80	2.57
K	Ca	Sc	Ti	V	Cr	Mn	Fe	Co	Ni	Cu	Zn	Ga	Ge	As	Se	Br	Kr
5.18	4.78	4.56	4.35	4.04	3.80	3.61	3.44	3.30	3.17	3.49	3.27	4.01	3.69	3.43	3.21	3.02	2.85
5.12	4.72	4.45	4.08	3.77	3.91	3.80	3.63	3.39	3.20	3.47	3.30	3.98	3.62	3.34	3.30	3.06	2.86
Rb	Sr	Y	Zr	Nb	Mo	Tc	Ru	Rh	Pd	Ag	Cd	In	Sn	Sb	Te	I	Xe
5.47	5.07	4.76	4.34	4.02	3.77	3.55	3.37	3.21	3.07	3.82	3.62	4.35	4.05	3.81	3.60	3.42	3.27
5.41	5.01	4.55	4.14	3.82	4.06	3.72	3.47	3.26	3.08	3.80	3.64	4.32	3.99	3.73	3.69	3.46	3.28

Table S1: Ionization radii R in a_0 for the H–Xe atoms from spin-restricted (in red) and spin-polarized (in blue) non-relativistic ΔSCF calculations with fractional occupations and the PW92 functional with configurations fixed to the ground states of the unconfined neutral atom and its cation. Values obtained while considering the R dependence of the ground state of the neutral atom and its cation are highlighted in the insert for the elements where the value differs from the static configurations.

H		Sc	Ti	V	Mn	Fe	Co	Ni									He
2.75		5.12	4.88	4.69	4.66	4.52	4.40	4.30									1.76
2.53		5.18	5.29	4.76	4.58	4.38	4.33	4.18									1.76
Li	Be	Y	Zr	Nb	Mo	Tc	Ru					B	C	N	O	F	Ne
5.36	3.88	5.60	5.20	4.99	5.06	4.85	4.50					4.13	3.47	3.01	2.67	2.40	2.19
5.11	3.88	5.53	5.22	5.01	4.84	4.73	4.64					3.98	3.29	2.84	2.83	2.46	2.19
Na	Mg											Al	Si	P	S	Cl	Ar
5.53	4.47											5.30	4.51	3.96	3.54	3.22	2.96
5.30	4.47											5.15	4.33	3.79	3.67	3.26	2.96
K	Ca	Sc	Ti	V	Cr	Mn	Fe	Co	Ni	Cu	Zn	Ga	Ge	As	Se	Br	Kr
6.44	5.44	5.12	4.96	5.23	4.81	4.66	4.52	4.61	4.81	4.20	3.86	5.18	4.56	4.11	3.77	3.49	3.26
6.22	5.44	5.57	5.29	4.76	4.59	4.82	4.82	4.33	4.30	4.10	3.86	5.04	4.39	3.95	3.89	3.53	3.26
Rb	Sr	Y	Zr	Nb	Mo	Tc	Ru	Rh	Pd	Ag	Cd	In	Sn	Sb	Te	I	Xe
6.73	5.82	5.60	5.46	5.32	5.28	4.85	4.50	4.21	3.97	4.53	4.21	5.46	4.90	4.49	4.17	3.91	3.69
6.53	5.82	5.93	5.22	5.30	4.84	4.75	4.71	4.29	3.97	4.44	4.21	5.33	4.75	4.34	4.28	3.95	3.69

Table S2: Ionization radii R in a_0 for the H–Xe atoms obtained via Janak's theorem from spin-restricted (in red) calculations and spin-polarized (in blue) with fractional occupations and the PW92 functional with configurations fixed to the ground states of the unconfined neutral atom and its cation. Values obtained while considering the R dependence of the ground state of the neutral atom and its cation are highlighted in the insert for the elements where the value differs from the static configurations.

H		Ca	Sc	Ti	V	Cr	Mn	Fe	Co	Ni							He
1.95		4.61	4.37	4.54	4.44	4.36	3.98	3.86	3.75	3.65							1.32
1.84		4.75	4.54	4.26	4.08	3.92	4.01	3.82	3.64	3.57							1.39
Li	Be	Sr	Y	Zr	Nb	Mo	Tc	Ru				B	C	N	O	F	Ne
4.27	3.13	5.00	4.15	4.90	4.84	4.41	3.56	3.36				3.08	2.66	2.36	2.12	1.94	1.78
4.09	3.24	5.13	4.91	4.55	4.36	4.21	3.69	4.03				3.02	2.55	2.23	2.29	2.00	1.80
Na	Mg											Al	Si	P	S	Cl	Ar
4.51	3.69											4.15	3.64	3.26	2.97	2.73	2.53
4.34	3.81											4.09	3.52	3.13	3.13	2.79	2.55
K	Ca	Sc	Ti	V	Cr	Mn	Fe	Co	Ni	Cu	Zn	Ga	Ge	As	Se	Br	Kr
5.43	4.84	4.67	4.46	4.19	3.91	3.68	3.49	3.33	3.20	3.57	3.28	4.06	3.70	3.42	3.19	2.99	2.83
5.25	4.79	4.49	4.12	3.77	3.92	3.81	3.72	3.48	3.24	3.50	3.34	3.99	3.59	3.29	3.34	3.05	2.84
Rb	Sr	Y	Zr	Nb	Mo	Tc	Ru	Rh	Pd	Ag	Cd	In	Sn	Sb	Te	I	Xe
5.75	5.16	4.95	4.45	4.08	3.79	3.56	3.36	3.19	3.05	3.91	3.65	4.42	4.08	3.81	3.59	3.41	3.25
5.57	5.09	4.59	4.11	3.77	4.21	3.85	3.52	3.27	3.07	3.85	3.70	4.36	3.97	3.69	3.75	3.47	3.26

Table S3: Ionization radii R in a_0 for the H–Xe atoms from spin-restricted (in red) and spin-polarized (in blue) non-relativistic ΔSCF calculations with fractional occupations and the r^{2}SCAN functional with configurations fixed to the ground states of the unconfined neutral atom and its cation. Values obtained while considering the R dependence of the ground state of the neutral atom and its cation are highlighted in the insert for the elements where the value differs from the static configurations.

H		Sc	V	Cr	Fe	Co	Ni										He
2.66		5.29	4.67	4.51	4.60	4.48	4.37										1.69
2.39		5.20	4.73	4.52	4.38	4.56	4.11										1.69
Li	Be	Y	Zr	Nb	Mo	Ru						B	C	N	O	F	Ne
5.36	3.82	5.79	5.21	5.00	5.16	4.57						4.19	3.47	2.98	2.63	2.36	2.14
4.98	3.82	5.58	5.17	4.96	4.79	4.59						3.96	3.22	2.75	2.86	2.43	2.14
Na	Mg											Al	Si	P	S	Cl	Ar
5.62	4.45											5.33	4.49	3.92	3.50	3.17	2.91
5.25	4.45											5.10	4.24	3.68	3.69	3.23	2.91
K	Ca	Sc	Ti	V	Cr	Mn	Fe	Co	Ni	Cu	Zn	Ga	Ge	As	Se	Br	Kr
6.63	5.47	5.29	4.87	5.05	4.97	4.73	4.60	4.80	5.12	4.27	3.84	5.24	4.55	4.08	3.73	3.45	3.22
6.22	5.47	5.91	5.34	4.73	4.52	4.61	5.01	4.56	4.21	4.04	3.84	5.00	4.31	3.85	3.91	3.51	3.22
Rb	Sr	Y	Zr	Nb	Mo	Tc	Ru	Rh	Pd	Ag	Cd	In	Sn	Sb	Te	I	Xe
6.98	5.88	5.79	5.35	5.52	5.47	4.97	4.57	4.25	3.98	4.64	4.22	5.54	4.91	4.48	4.15	3.88	3.65
6.56	5.88	6.08	5.17	5.27	4.79	5.08	4.94	4.38	3.98	4.42	4.22	5.32	4.68	4.25	4.33	3.94	3.65

Table S4: Ionization radii R in a_0 for the H–Xe atoms obtained via Janak's theorem from spin-restricted (in red) calculations and spin-polarized (in blue) with fractional occupations and the r^{2}SCAN functional with configurations fixed to the ground states of the unconfined neutral atom and its cation. Values obtained while considering the R dependence of the ground state of the neutral atom and its cation are highlighted in the insert for the elements where the value differs from the static configurations.

H		Sc	V	Mn	Fe	Co	Ni										He
2.71		5.17	4.73	4.73	4.60	4.48	4.37										1.74
2.48		5.22	4.83	4.63	4.43	4.46	4.22										1.74
Li	Be	Y	Zr	Nb	Mo	Ru						B	C	N	O	F	Ne
5.34	3.85	5.69	5.25	5.32	5.15	4.60						4.16	3.48	3.02	2.68	2.41	2.20
5.03	3.85	5.59	5.26	5.05	4.89	4.69						3.99	3.30	2.84	2.85	2.47	2.20
Na	Mg											Al	Si	P	S	Cl	Ar
5.60	4.49											5.32	4.51	3.96	3.54	3.22	2.95
5.32	4.49											5.13	4.31	3.77	3.69	3.27	2.95
K	Ca	Sc	Ti	V	Cr	Mn	Fe	Co	Ni	Cu	Zn	Ga	Ge	As	Se	Br	Kr
6.58	5.48	5.17	4.92	5.34	4.94	4.73	4.60	4.60	4.78	4.27	3.90	5.28	4.60	4.14	3.78	3.50	3.27
6.30	5.48	5.60	5.39	4.83	4.63	4.79	5.02	4.46	4.25	4.15	3.90	5.08	4.40	3.94	3.93	3.55	3.27
Rb	Sr	Y	Zr	Nb	Mo	Tc	Ru	Rh	Pd	Ag	Cd	In	Sn	Sb	Te	I	Xe
6.91	5.88	5.69	5.49	5.40	5.43	4.97	4.60	4.29	4.04	4.63	4.28	5.57	4.96	4.53	4.19	3.92	3.70
6.63	5.88	6.08	5.26	5.38	4.89	4.87	4.86	4.39	4.04	4.51	4.28	5.40	4.76	4.34	4.34	3.97	3.70

Table S5: Ionization radii R in a_0 for the H–Xe atoms obtained via Janak's theorem from spin-restricted (in red) calculations and spin-polarized (in blue) with fractional occupations and the PBE functional with configurations fixed to the ground states of the unconfined neutral atom and its cation. Values obtained while considering the R dependence of the ground state of the neutral atom and its cation are highlighted in the insert for the elements where the value differs from the static configurations.

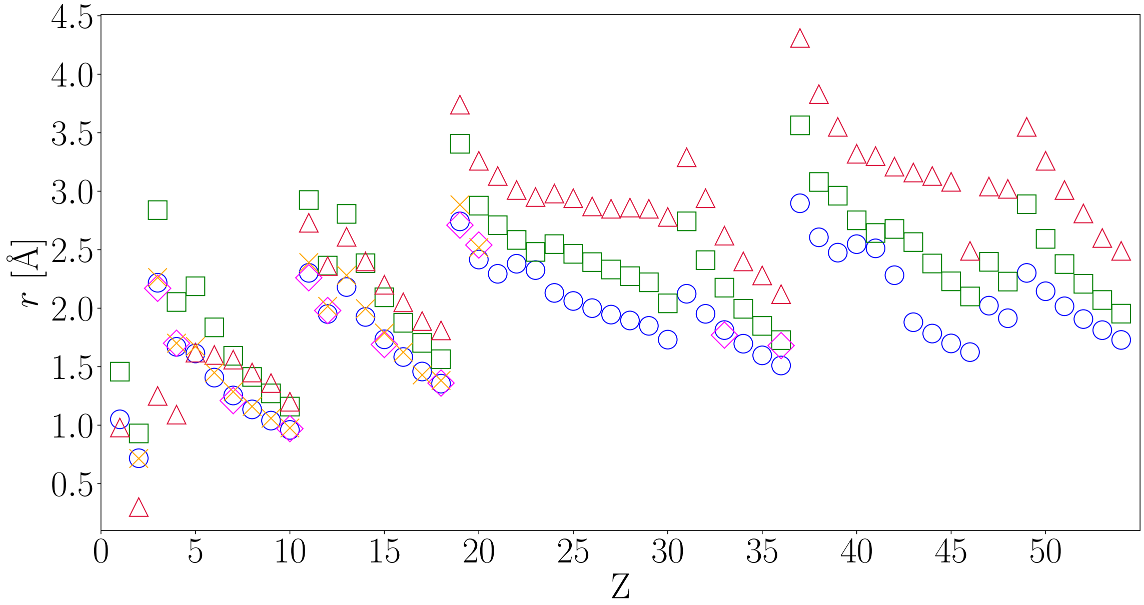

Figure S3: Comparison of ionization radii computed in this work with spin-restricted densities and the PW92 density functional via ΔSCF (blue circles) or Janak's theorem (green squares) against the Hartree–Fock values of Boeyens (red triangles) and DFT values of Sen (magenta diamonds) and Garza (orange crosses).

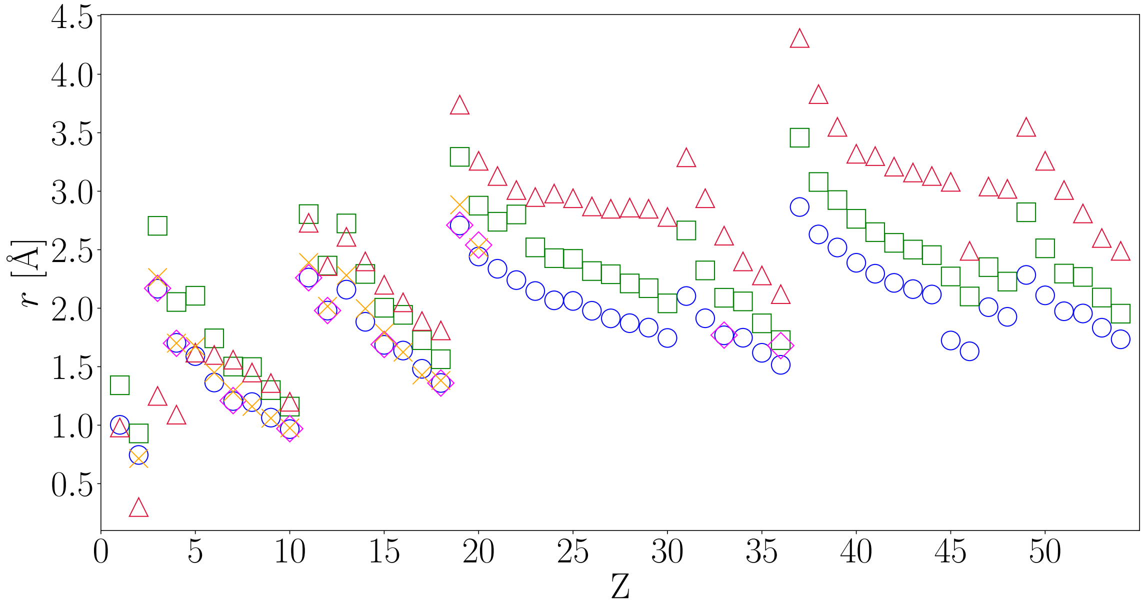

Figure S4: Comparison of ionization radii computed in this work with spin-polarized densities and the PW92 density functional via ΔSCF (blue circles) or Janak's theorem (green squares) against the Hartree–Fock values of Boeyens (red triangles) and DFT values of Sen (magenta diamonds) and Garza (orange crosses).

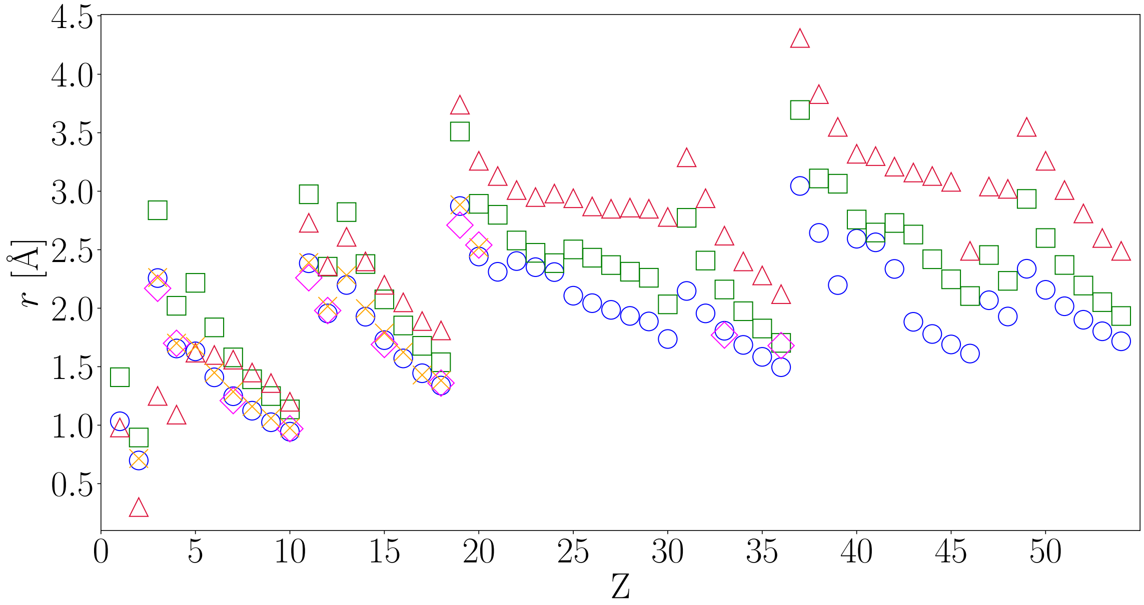

Figure S5: Comparison of ionization radii computed in this work with spin-restricted densities and the r^{2}SCAN density functional via ΔSCF (blue circles) or Janak's theorem (green squares) against the Hartree–Fock values of Boeyens (red triangles) and DFT values of Sen (magenta diamonds) and Garza (orange crosses).

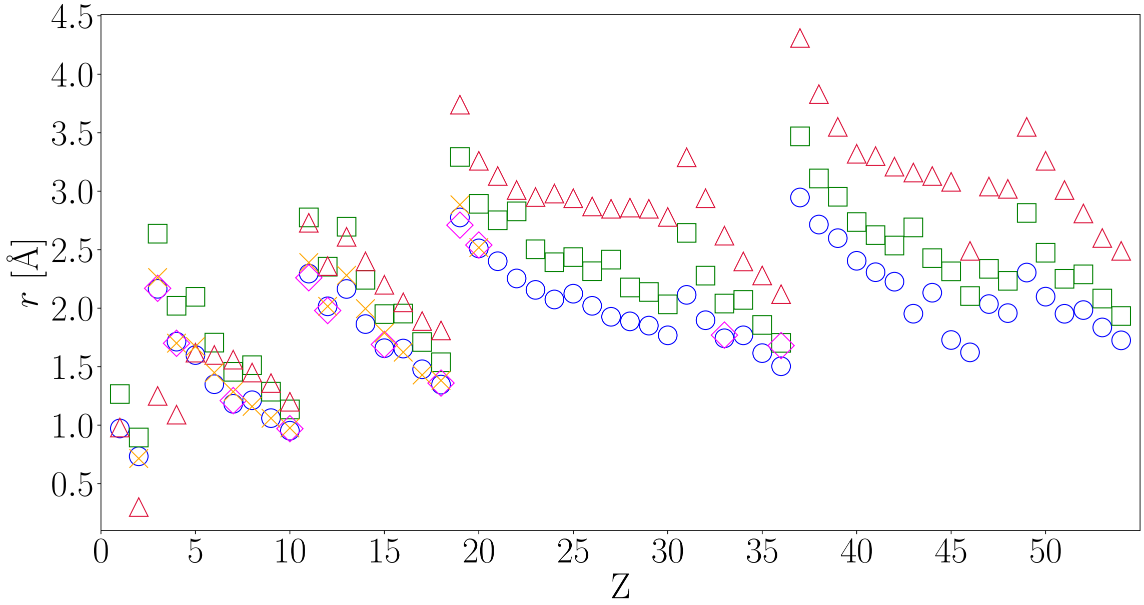

Figure S6: Comparison of ionization radii computed in this work with spin-polarized densities and the r^{2}SCAN density functional via ΔSCF (blue circles) or Janak's theorem (green squares) against the Hartree–Fock values of Boeyens (red triangles) and DFT values of Sen (magenta diamonds) and Garza (orange crosses).

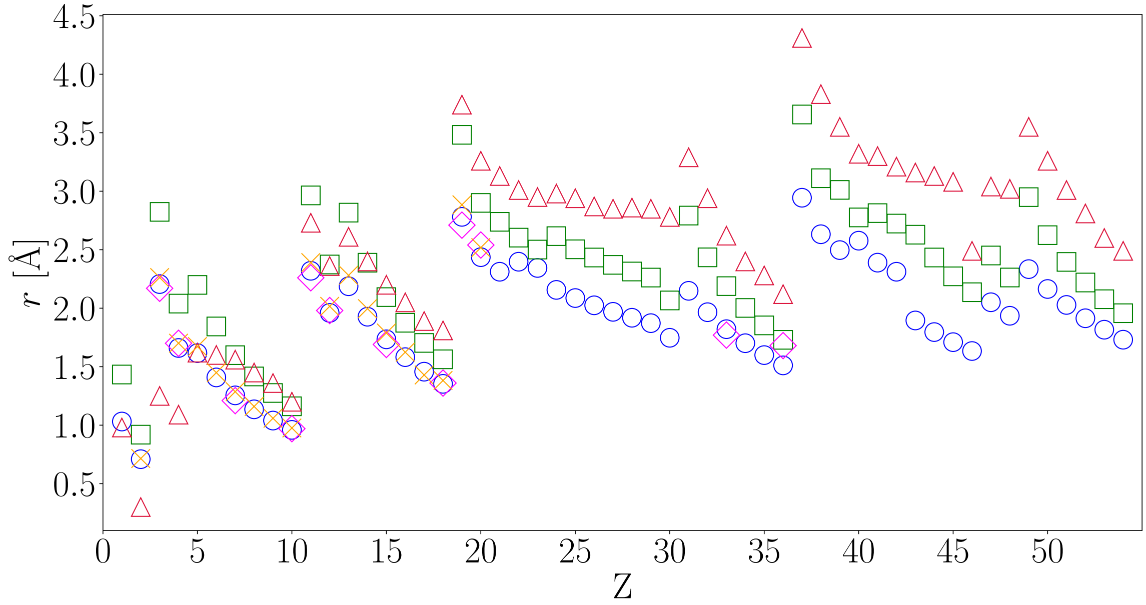

Figure S7: Comparison of ionization radii computed in this work with spin-restricted densities and the PBE density functional via ΔSCF (blue circles) or Janak's theorem (green squares) against the Hartree–Fock values of Boeyens (red triangles) and DFT values of Sen (magenta diamonds) and Garza (orange crosses).

3 Spin-restricted analysis

We present a detailed analysis of the evolution of the ground state as a function of confinement of the spin-restricted H–Xe atoms. The analysis is carried out one atom at a time. We also detail any flips between low-lying configurations and the unconfined ground state configuration. Plots are also included for all calculations, and as discussed in the main text, the energies are shown relative to the ground state of the unconfined atom

$$\Delta E_i(r_c) = E_i(r_c) - E_0^{\text{unconfined}}. \tag{1}$$

Furthermore, we provide some information on the various electronic configurations in the unconfined atom. We provide two estimates for the van der Waals radius in units of a_0: r_ϵ, and r_ρ. r_ϵ has been computed to the threshold $\epsilon = 7.341668 \times 10^{-2}$ and r_ρ to the threshold $\epsilon = 10^{-3}$ for each configuration. We also provide the location density maximum of the valence orbital in units of a_0, as a third metric for the atomic size. The excitation energy relative to the ground state, ΔE, is also given in units of eV. The ionization energy computed with the ΔSCF approach provides a point of reference for the excitation energies, as configurations with excitation energies smaller than the ionization potential are bound with respect to ionization—even if some such configurations have extremely diffuse orbitals. The ionization energy is marked in the tables by a horizontal line.

3.1 PW92

H The energies of the low lying configurations of hard-wall confined H are shown in fig. S8 for the neutral atom. The ground state of the unconfined H is $1s^1$. We do not observe any ground state crossing for H in the considered confinement radii.

The ionization energy of the unconfined atom is $\Delta E_0 = 12.13$ eV. The studied configurations, atomic radii and excitation energies in the unconfined calculations are as follows:

configuration	r_ϵ	r_ρ	$r_{\max}$	ΔE
$1s^1$	3.28	3.15	1.06	0.00
$2p^1$	9.12	3.83	4.26	8.91
$3d^1$	16.63	undefined	9.45	10.60

He The energies of the low lying configurations of hard-wall confined He are shown in fig. S9 for the neutral atom and in fig. S10 for the cation. The ground state of the unconfined He is $1s^2$. We do not observe any ground state crossing for He in the considered confinement radii.

The ionization energy of the unconfined atom is $\Delta E_0 = 26.49$ eV. The studied configurations, atomic radii and excitation energies in the unconfined calculations are as follows:

configuration	r_ϵ	r_ρ	$r_{\max}$	ΔE
$1s^2$	2.28	2.68	0.57	0.00
$1s^1 2p^1$	8.54	3.94	3.76	22.96
$1s^1 3d^1$	16.57	2.10	9.39	24.96
$2p^2$	6.56	4.59	2.37	58.18

Li The energies of the low lying configurations of hard-wall confined Li are shown in fig. S11 for the neutral atom and in fig. S12 for the cation. The ground state of the unconfined Li is [He]$2s^1$. At $r_c = 3.1a_0$ the ground state changes to [He]$2p^1$.

The ionization energy of the unconfined atom is $\Delta E_0 = 5.24$ eV. The studied configurations, atomic radii and excitation energies in the unconfined calculations are as follows:

configuration	r_ϵ	r_ρ	$r_{\max}$	ΔE
[He]$2s^1$	6.64	4.16	3.12	0.00
[He]$2p^1$	8.60	3.89	3.81	1.75
[He]$3d^1$	16.60	1.91	9.42	3.71

Be The energies of the low lying configurations of hard-wall confined Be are shown in fig. S13 for the neutral atom and in fig. S14 for the cation. The ground state of the unconfined Be is [He]$2s^2$. At $r_c = 2.3a_0$ the ground state changes to [He]$2p^2$.

At $r_c = 2.3a_0$ the state [He]$2s^1 2p^1$ flips below the initial ground state. At $r_c = 1.2a_0$ the state [He]$2p^1 3d^1$ flips below the initial ground state. The ionization energy of the unconfined atom is $\Delta E_0 = 9.51$ eV. The studied configu-

rations, atomic radii and excitation energies in the unconfined calculations are as follows:

configuration	r_ϵ	r_ρ	$r_{\max}$	ΔE
[He]$2s^2$	5.09	4.16	2.04	0.00
[He]$2s^12p^1$	5.90	4.31	2.22	3.52
[He]$2p^2$	6.32	4.45	2.17	7.06
[He]$2p^13d^1$	15.25	3.83	7.99	11.63

B The energies of the low lying configurations of hard-wall confined B are shown in fig. S15 for the neutral atom and in fig. S16 for the cation. The ground state of the unconfined B is [He]$2s^22p^1$. At $r_c = 1.8a_0$ the ground state changes to [He]$2p^3$.

At $r_c = 1.8a_0$ the state [He]$2s^12p^2$ flips below the initial ground state. The ionization energy of the unconfined atom is $\Delta E_0 = 8.32$ eV. The studied configurations, atomic radii and excitation energies in the unconfined calculations are as follows:

configuration	r_ϵ	r_ρ	$r_{\max}$	ΔE
[He]$2s^22p^1$	4.65	3.98	1.56	0.00
[He]$2s^23s^1$	10.09	3.78	5.61	5.03
[He]$2s^12p^2$	4.96	4.08	1.54	5.68
[He]$2p^3$	5.15	4.16	1.53	11.39

C The energies of the low lying configurations of hard-wall confined C are shown in fig. S17 for the neutral atom and in fig. S18 for the cation. The ground state of the unconfined C is [He]$2s^22p^2$. At $r_c = 1.5a_0$ the ground state changes to [He]$2p^4$.

At $r_c = 1.5a_0$ the state [He]$2s^12p^3$ flips below the initial ground state. The ionization energy of the unconfined atom is $\Delta E_0 = 10.99$ eV. The studied configurations, atomic radii and excitation energies in the unconfined calculations are as follows:

configuration	r_ϵ	r_ρ	$r_{\max}$	ΔE
[He]$2s^22p^2$	4.12	3.78	1.22	0.00
[He]$2s^22p^13s^1$	9.08	4.15	4.86	7.27
[He]$2s^12p^3$	4.29	3.83	1.21	8.23
[He]$2p^4$	4.40	3.89	1.18	16.49

N The energies of the low lying configurations of hard-wall confined N are shown in fig. S19 for the neutral atom and in fig. S20 for the cation. The ground state of the unconfined N is [He]$2s^22p^3$. At $r_c = 1.3a_0$ the ground state changes to [He]$2p^5$.

At $r_c = 1.3a_0$ the state [He]$2s^12p^4$ flips below the initial ground state. The ionization energy of the unconfined atom is $\Delta E_0 = 13.74$ eV. The studied configurations, atomic radii and excitation energies in the unconfined calculations are as follows:

configuration	r_ϵ	r_ρ	$r_{\max}$	ΔE
[He]$2s^22p^3$	3.69	3.57	1.02	0.00
[He]$2s^22p^23s^1$	8.38	4.31	4.35	9.67
[He]$2s^12p^4$	3.79	3.64	1.02	11.17
[He]$2p^5$	3.87	3.64	0.96	22.39

O The energies of the low lying configurations of hard-wall confined O are shown in fig. S21 for the neutral atom and in fig. S22 for the cation. The ground state of the unconfined O is [He]$2s^22p^4$. At $r_c = 1.1a_0$ the ground state changes to [He]$2p^6$.

At $r_c = 1.1a_0$ the state [He]$2s^12p^5$ flips below the initial ground state. The ionization energy of the unconfined atom is $\Delta E_0 = 16.59$ eV. The studied configurations, atomic radii and excitation energies in the unconfined calculations are as follows:

configuration	r_ϵ	r_ρ	$r_{\max}$	ΔE
[He]$2s^22p^4$	3.34	3.41	0.87	0.00
[He]$2s^22p^33s^1$	7.85	4.35	3.97	12.21
[He]$2s^12p^5$	3.42	3.41	0.87	14.53
[He]$2p^6$	3.48	3.49	0.82	29.12

F The energies of the low lying configurations of hard-wall confined F are shown in fig. S23 for the neutral atom and in fig. S24 for the cation. The ground state of the unconfined F is [He]$2s^22p^5$. At $r_c = 1.0a_0$ the ground state changes to [He]$2s^12p^6$.

The ionization energy of the unconfined atom is $\Delta E_0 = 19.57$ eV. The studied configurations, atomic radii and excitation energies in the unconfined calculations are as follows:

configuration	r_ϵ	r_ρ	$r_{\max}$	ΔE
[He]$2s^22p^5$	3.06	3.24	0.77	0.00
[He]$2s^22p^43s^1$	7.43	4.31	3.68	14.90
[He]$2s^22p^43d^1$	16.33	2.87	9.15	18.02
[He]$2s^12p^6$	3.12	3.24	0.77	18.30
[He]$2p^63d^1$	16.28	2.96	9.09	55.21

Ne The energies of the low lying configurations of hard-wall confined Ne are shown in fig. S25 for the neutral atom and in fig. S26 for the cation. The ground state of the unconfined Ne is [He]$2s^2 2p^6$. We do not observe any ground state crossing for Ne in the considered confinement radii.

The ionization energy of the unconfined atom is $\Delta E_0 = 22.67$ eV. The studied configurations, atomic radii and excitation energies in the unconfined calculations are as follows:

configuration	r_ϵ	r_ρ	$r_{\max}$	ΔE
[He]$2s^2 2p^6$	2.83	3.06	0.68	0.00
[He]$2s^2 2p^5 3s^1$	7.10	4.26	3.44	17.74
[He]$2s^2 2p^5 3d^1$	16.40	2.78	9.22	21.13
[He]$2s^1 2p^6 3d^1$	16.38	2.78	9.20	43.88

Na The energies of the low lying configurations of hard-wall confined Na are shown in fig. S27 for the neutral atom and in fig. S28 for the cation. The ground state of the unconfined Na is [Ne]$3s^1$. At $r_c = 2.0a_0$ the ground state changes to [Ne]$3d^1$.

At $r_c = 1.6a_0$ the state [Ne]$3p^1$ flips below the initial ground state. At $r_c = 1.1a_0$ the state [Ne]$4f^1$ flips below the initial ground state. The ionization energy of the unconfined atom is $\Delta E_0 = 5.16$ eV. The studied configurations, atomic radii and excitation energies in the unconfined calculations are as follows:

configuration	r_ϵ	r_ρ	$r_{\max}$	ΔE
[Ne]$3s^1$	6.81	4.21	3.24	0.00
[Ne]$3p^1$	9.74	3.49	4.78	2.11
[Ne]$3d^1$	16.45	2.68	9.27	3.62
[Ne]$4f^1$	25.25	2.59	16.46	4.26

Mg The energies of the low lying configurations of hard-wall confined Mg are shown in fig. S29 for the neutral atom and in fig. S30 for the cation. The ground state of the unconfined Mg is [Ne]$3s^2$. At $r_c = 1.8a_0$ the ground state changes to [Ne]$3d^2$.

At $r_c = 1.8a_0$ the state [Ne]$3s^1 3d^1$ flips below the initial ground state. At $r_c = 1.7a_0$ the state [Ne]$3p^1 3d^1$ flips below the initial ground state. At $r_c = 1.4a_0$ the state [Ne]$3s^1 3p^1$ flips below the initial ground state. At $r_c = 1.4a_0$ the state [Ne]$3p^2$ flips below the initial ground state. At $r_c = 1.3a_0$ the state [Ne]$3d^1 4f^1$ flips below the initial ground state. The ionization energy of the unconfined atom is $\Delta E_0 = 8.06$ eV. The studied configurations, atomic radii and excitation energies in the unconfined calculations are as follows:

configuration	r_ϵ	r_ρ	$r_{\max}$	ΔE
[Ne]$3s^2$	5.86	4.59	2.52	0.00
[Ne]$3s^1 3p^1$	7.33	4.78	3.32	3.51
[Ne]$3s^1 3d^1$	14.74	4.11	7.38	6.29
[Ne]$3p^2$	7.63	5.17	3.19	7.23
[Ne]$3p^1 3d^1$	13.27	4.78	6.11	10.43
[Ne]$3d^2$	11.94	5.17	5.17	14.26
[Ne]$3d^1 4f^1$	22.37	3.94	12.93	15.91

Al The energies of the low lying configurations of hard-wall confined Al are shown in fig. S31 for the neutral atom and in fig. S32 for the cation. The ground state of the unconfined Al is [Ne]$3s^2 3p^1$. At $r_c = 1.9a_0$ the ground state changes to [Ne]$3s^2 3d^1$. Furthermore, at $r_c = 1.6a_0$ we see a ground state crossing to [Ne]$3d^3$.

At $r_c = 1.7a_0$ the state [Ne]$3s^1 3d^2$ flips below the initial ground state. At $r_c = 1.6a_0$ the state [Ne]$3p^1 3d^2$ flips below the initial ground state. At $r_c = 1.3a_0$ the state [Ne]$3s^1 3p^2$ flips below the initial ground state. The ionization energy of the unconfined atom is $\Delta E_0 = 5.85$ eV. The studied configurations, atomic radii and excitation energies in the unconfined calculations are as follows:

configuration	r_ϵ	r_ρ	$r_{\max}$	ΔE
[Ne]$3s^2 3p^1$	6.09	4.67	2.63	0.00
[Ne]$3s^2 4s^1$	11.46	4.11	6.66	2.98
[Ne]$3s^2 3d^1$	14.36	4.19	6.81	4.02
[Ne]$3s^1 3p^2$	6.38	4.89	2.55	5.12
[Ne]$3s^1 3d^2$	11.15	5.33	4.22	15.22
[Ne]$3p^1 3d^2$	10.66	5.50	3.94	21.21
[Ne]$3d^3$	9.82	5.90	3.54	27.63

Si The energies of the low lying configurations of hard-wall confined Si are shown in fig. S33 for the neutral atom and in fig. S34 for the cation. The ground state of the unconfined Si is [Ne]$3s^2 3p^2$. At $r_c = 1.7a_0$ the ground state changes to [Ne]$3s^2 3d^2$. Furthermore, at $r_c = 1.5a_0$ we see a ground state crossing to [Ne]$3d^4$.

At $r_c = 1.7a_0$ the state [Ne]$3s^23p^13d^1$ flips below the initial ground state. At $r_c = 1.6a_0$ the state [Ne]$3p^13d^3$ flips below the initial ground state. At $r_c = 1.6a_0$ the state [Ne]$3s^13d^3$ flips below the initial ground state. At $r_c = 1.2a_0$ the state [Ne]$3s^13p^3$ flips below the initial ground state. The ionization energy of the unconfined atom is $\Delta E_0 = 7.83$ eV. The studied configurations, atomic radii and excitation energies in the unconfined calculations are as follows:

configuration	r_ϵ	r_ρ	$r_{\max}$	ΔE
[Ne]$3s^23p^2$	5.54	4.51	2.15	0.00
[Ne]$3s^23p^14s^1$	10.32	4.51	5.81	4.58
[Ne]$3s^23p^13d^1$	13.63	4.35	5.60	5.86
[Ne]$3s^13p^3$	5.64	4.67	2.10	6.75
[Ne]$3s^23d^2$	10.79	5.17	3.63	13.31
[Ne]$3s^13d^3$	9.34	5.50	2.98	29.26
[Ne]$3p^13d^3$	9.09	5.50	2.88	37.02
[Ne]$3d^4$	8.53	5.69	2.69	46.13

P The energies of the low lying configurations of hard-wall confined P are shown in fig. S35 for the neutral atom and in fig. S36 for the cation. The ground state of the unconfined P is [Ne]$3s^23p^3$. At $r_c = 1.6a_0$ the ground state changes to [Ne]$3s^23p^23d^1$. Furthermore, at $r_c = 1.5a_0$ we see a ground state crossing to [Ne]$3s^23d^3$. At $r_c = 1.4a_0$ we see a ground state crossing to [Ne]$3d^5$.

At $r_c = 1.5a_0$ the state [Ne]$3p^13d^4$ flips below the initial ground state. At $r_c = 1.5a_0$ the state [Ne]$3s^13d^4$ flips below the initial ground state. At $r_c = 1.5a_0$ the state [Ne]$3s^23p^13d^2$ flips below the initial ground state. At $r_c = 1.2a_0$ the state [Ne]$3s^13p^4$ flips below the initial ground state. The ionization energy of the unconfined atom is $\Delta E_0 = 9.83$ eV. The studied configurations, atomic radii and excitation energies in the unconfined calculations are as follows:

configuration	r_ϵ	r_ρ	$r_{\max}$	ΔE
[Ne]$3s^23p^3$	5.03	4.31	1.83	0.00
[Ne]$3s^23p^24s^1$	9.57	4.59	5.24	6.28
[Ne]$3s^23p^23d^1$	13.53	4.21	4.97	7.80
[Ne]$3s^13p^4$	5.07	4.39	1.80	8.41
[Ne]$3s^23p^13d^2$	10.34	5.02	3.00	17.54
[Ne]$3s^23d^3$	9.07	5.33	2.58	28.16
[Ne]$3s^13d^4$	8.20	5.50	2.32	48.84
[Ne]$3p^13d^4$	8.04	5.33	2.27	58.35
[Ne]$3d^5$	7.64	5.50	2.16	70.23

S The energies of the low lying configurations of hard-wall confined S are shown in fig. S37 for the neutral atom and in fig. S38 for the cation. The ground state of the unconfined S is [Ne]$3s^23p^4$. At $r_c = 1.4a_0$ the ground state changes to [Ne]$3s^23d^4$. Furthermore, at $r_c = 1.3a_0$ we see a ground state crossing to [Ne]$3d^6$.

At $r_c = 1.4a_0$ the state [Ne]$3p^13d^5$ flips below the initial ground state. At $r_c = 1.4a_0$ the state [Ne]$3s^13d^5$ flips below the initial ground state. At $r_c = 1.4a_0$ the state [Ne]$3s^23p^13d^3$ flips below the initial ground state. At $r_c = 1.4a_0$ the state [Ne]$3s^23p^23d^2$ flips below the initial ground state. At $r_c = 1.4a_0$ the state [Ne]$3s^23p^33d^1$ flips below the initial ground state. At $r_c = 1.1a_0$ the state [Ne]$3s^13p^5$ flips below the initial ground state. The ionization energy of the unconfined atom is $\Delta E_0 = 11.87$ eV. The studied configurations, atomic radii and excitation energies in the unconfined calculations are as follows:

configuration	r_ϵ	r_ρ	$r_{\max}$	ΔE
[Ne]$3s^23p^4$	4.60	4.11	1.60	0.00
[Ne]$3s^23p^34s^1$	9.02	4.59	4.83	8.07
[Ne]$3s^23p^33d^1$	13.71	4.08	4.81	9.84
[Ne]$3s^13p^5$	4.62	4.15	1.58	10.11
[Ne]$3s^23p^23d^2$	10.14	4.89	2.55	22.03
[Ne]$3s^23p^13d^3$	8.75	5.17	2.20	35.17
[Ne]$3s^23d^4$	8.00	5.33	2.03	48.93
[Ne]$3s^13d^5$	7.39	5.17	1.90	74.40
[Ne]$3p^13d^5$	7.28	5.17	1.87	85.68
[Ne]$3d^6$	6.97	5.17	1.81	100.45

Cl The energies of the low lying configurations of hard-wall confined Cl are shown in fig. S39 for the neutral atom and in fig. S40

for the cation. The ground state of the unconfined Cl is [Ne]$3s^2 3p^5$. At $r_c = 1.3a_0$ the ground state changes to [Ne]$3s^1 3d^6$. Furthermore, at $r_c = 1.2a_0$ we see a ground state crossing to [Ne]$3d^7$.

At $r_c = 1.3a_0$ the state [Ne]$3p^1 3d^6$ flips below the initial ground state. At $r_c = 1.3a_0$ the state [Ne]$3s^2 3p^4 3d^1$ flips below the initial ground state. At $r_c = 1.3a_0$ the state [Ne]$3s^2 3p^3 3d^2$ flips below the initial ground state. At $r_c = 1.0a_0$ the state [Ne]$3s^1 3p^6$ flips below the initial ground state. The ionization energy of the unconfined atom is $\Delta E_0 = 13.98$ eV. The studied configurations, atomic radii and excitation energies in the unconfined calculations are as follows:

configuration	r_ϵ	r_ρ	$r_{\max}$	ΔE
[Ne]$3s^2 3p^5$	4.24	3.98	1.43	0.00
[Ne]$3s^2 3p^4 4s^1$	8.58	4.59	4.52	9.97
[Ne]$3s^1 3p^6$	4.26	3.98	1.41	11.87
[Ne]$3s^2 3p^4 3d^1$	14.01	3.94	5.51	11.98
[Ne]$3s^2 3p^3 3d^2$	10.11	4.67	2.19	26.78
[Ne]$3s^1 3d^6$	6.78	5.02	1.61	106.40
[Ne]$3p^1 3d^6$	6.70	5.02	1.60	119.47
[Ne]$3d^7$	6.45	5.02	1.56	137.26

Ar The energies of the low lying configurations of hard-wall confined Ar are shown in fig. S41 for the neutral atom and in fig. S42 for the cation. The ground state of the unconfined Ar is [Ne]$3s^2 3p^6$. At $r_c = 1.2a_0$ the ground state changes to [Ne]$3s^2 3d^6$. Furthermore, at $r_c = 1.1a_0$ we see a ground state crossing to [Ne]$3d^8$.

At $r_c = 1.2a_0$ the state [Ne]$3p^1 3d^7$ flips below the initial ground state. At $r_c = 1.2a_0$ the state [Ne]$3s^1 3d^7$ flips below the initial ground state. At $r_c = 1.2a_0$ the state [Ne]$3s^2 3p^1 3d^5$ flips below the initial ground state. At $r_c = 1.2a_0$ the state [Ne]$3s^2 3p^5 3d^1$ flips below the initial ground state. At $r_c = 1.2a_0$ the state [Ne]$3s^2 3p^4 3d^2$ flips below the initial ground state. At $r_c = 1.1a_0$ the state [Ne]$3s^1 3p^6 3d^1$ flips below the initial ground state. The ionization energy of the unconfined atom is $\Delta E_0 = 16.17$ eV. The studied configurations, atomic radii and excitation energies in the unconfined calculations are as follows:

configuration	r_ϵ	r_ρ	$r_{\max}$	ΔE
[Ne]$3s^2 3p^6$	3.94	3.83	1.29	0.00
[Ne]$3s^2 3p^5 4s^1$	8.23	4.51	4.26	11.96
[Ne]$3s^2 3p^5 3d^1$	14.34	3.78	6.33	14.21
[Ne]$3s^1 3p^6 3d^1$	14.16	3.83	5.67	28.36
[Ne]$3s^2 3p^4 3d^2$	10.19	4.59	1.90	31.79
[Ne]$3s^2 3p^1 3d^5$	7.04	4.89	1.50	89.61
[Ne]$3s^2 3d^6$	6.65	4.89	1.45	109.85
[Ne]$3s^1 3d^7$	6.30	4.78	1.40	145.30
[Ne]$3p^1 3d^7$	6.24	4.78	1.39	160.22
[Ne]$3d^8$	6.03	4.78	1.37	181.18

K The energies of the low lying configurations of hard-wall confined K are shown in fig. S43 for the neutral atom and in fig. S44 for the cation. The ground state of the unconfined K is [Ar]$4s^1$. At $r_c = 4.6a_0$ the ground state changes to [Ar]$3d^1$. Furthermore, at $r_c = 1.2a_0$ we see a ground state crossing to [Ne]$3s^2 3p^5 3d^2$. At $r_c = 1.1a_0$ we see a ground state crossing to [Ne]$3d^9$.

At $r_c = 2.5a_0$ the state [Ne]$3s^1 3p^6 3d^2$ flips below the initial ground state. At $r_c = 2.3a_0$ the state [Ne]$3s^2 3p^4 3d^3$ flips below the initial ground state. At $r_c = 1.5a_0$ the state [Ne]$3s^1 3d^8$ flips below the initial ground state. At $r_c = 1.5a_0$ the state [Ne]$3p^1 3d^8$ flips below the initial ground state. At $r_c = 1.4a_0$ the state [Ar]$4p^1$ flips below the initial ground state. The ionization energy of the unconfined atom is $\Delta E_0 = 4.38$ eV. The studied configurations, atomic radii and excitation energies in the unconfined calculations are as follows:

configuration	r_ϵ	r_ρ	$r_{\max}$	ΔE
[Ar]$4s^1$	7.94	4.45	4.04	0.00
[Ar]$4p^1$	10.77	3.71	5.63	1.61
[Ar]$3d^1$	14.65	3.64	6.93	2.48
[Ne]$3s^2 3p^5 3d^2$	10.36	4.45	1.66	22.99
[Ne]$3s^1 3p^6 3d^2$	10.23	4.39	1.61	39.24
[Ne]$3s^2 3p^4 3d^3$	8.54	4.78	1.48	44.65
[Ne]$3s^1 3d^8$	5.91	4.67	1.24	177.55
[Ne]$3p^1 3d^8$	5.86	4.59	1.24	194.35
[Ne]$3d^9$	5.68	4.59	1.22	218.64

Ca The energies of the low lying configurations of hard-wall confined Ca are shown in fig. S45 for the neutral atom and in fig. S46 for the cation. The ground state of the un-

confined Ca is [Ar]$4s^2$. At $r_c = 4.7a_0$ the ground state changes to [Ar]$4s^1 3d^1$. Furthermore, at $r_c = 4.4a_0$ we see a ground state crossing to [Ar]$3d^2$. At $r_c = 1.1a_0$ we see a ground state crossing to [Ne]$3s^2 3p^4 3d^4$. Moreover, at $r_c = 1.0a_0$ we see a ground state crossing to [Ne]$3d^{10}$.

At $r_c = 3.5a_0$ the state [Ar]$3d^1 4p^1$ flips below the initial ground state. At $r_c = 3.1a_0$ the state [Ne]$3s^2 3p^5 3d^3$ flips below the initial ground state. At $r_c = 2.8a_0$ the state [Ne]$3s^1 3p^6 3d^3$ flips below the initial ground state. At $r_c = 1.8a_0$ the state [Ne]$3s^1 3d^9$ flips below the initial ground state. At $r_c = 1.7a_0$ the state [Ne]$3p^1 3d^9$ flips below the initial ground state. At $r_c = 1.4a_0$ the state [Ar]$4s^1 4p^1$ flips below the initial ground state. The ionization energy of the unconfined atom is $\Delta E_0 = 6.43$ eV. The studied configurations, atomic radii and excitation energies in the unconfined calculations are as follows:

configuration	r_ϵ	r_ρ	$r_{\max}$	ΔE
[Ar]$4s^2$	7.12	5.17	3.34	0.00
[Ar]$4s^1 3d^1$	7.95	4.89	3.50	2.14
[Ar]$4s^1 4p^1$	8.49	5.33	4.24	2.46
[Ar]$3d^1 4p^1$	9.46	4.78	4.69	4.28
[Ar]$3d^2$	10.57	4.31	1.46	4.72
[Ne]$3s^2 3p^5 3d^3$	8.63	4.67	1.32	29.54
[Ne]$3s^1 3p^6 3d^3$	8.52	4.59	1.30	47.88
[Ne]$3s^2 3p^4 3d^4$	7.54	4.67	1.25	55.09
[Ne]$3s^1 3d^9$	5.58	4.51	1.12	207.96
[Ne]$3p^1 3d^9$	5.54	4.45	1.11	226.71
[Ne]$3d^{10}$	5.38	4.45	1.10	254.49

Sc The energies of the low lying configurations of hard-wall confined Sc are shown in fig. S47 for the neutral atom and in fig. S48 for the cation. The ground state of the unconfined Sc is [Ar]$4s^2 3d^1$. At $r_c = 4.7a_0$ the ground state changes to [Ar]$4s^1 3d^2$. Furthermore, at $r_c = 4.3a_0$ we see a ground state crossing to [Ar]$3d^3$. At $r_c = 1.0a_0$ we see a ground state crossing to [Ne]$3s^2 3d^9$.

At $r_c = 3.3a_0$ the state [Ar]$3d^2 4p^1$ flips below the initial ground state. At $r_c = 3.0a_0$ the state [Ne]$3s^2 3p^5 3d^4$ flips below the initial ground state. At $r_c = 2.6a_0$ the state [Ne]$3s^1 3p^6 3d^4$ flips below the initial ground state. At $r_c = 2.5a_0$ the state [Ne]$3s^2 3p^4 3d^5$ flips below the initial ground state. At $r_c = 1.9a_0$ the state [Ne]$3s^2 3p^1 3d^8$ flips below the initial ground state. At $r_c = 1.7a_0$ the state [Ne]$3s^1 3d^{10}$ flips below the initial ground state. At $r_c = 1.3a_0$ the state [Ar]$4s^1 3d^1 4p^1$ flips below the initial ground state. The ionization energy of the unconfined atom is $\Delta E_0 = 7.02$ eV. The studied configurations, atomic radii and excitation energies in the unconfined calculations are as follows:

configuration	r_ϵ	r_ρ	$r_{\max}$	ΔE
[Ar]$4s^2 3d^1$	6.70	5.17	3.09	0.00
[Ar]$4s^1 3d^2$	7.08	4.89	3.24	1.49
[Ar]$4s^1 3d^1 4p^1$	8.02	5.33	3.92	2.79
[Ar]$3d^3$	8.77	4.59	1.19	3.84
[Ar]$3d^2 4p^1$	8.86	4.89	4.31	3.92
[Ne]$3s^2 3p^5 3d^4$	7.59	4.59	1.13	32.75
[Ne]$3s^1 3p^6 3d^4$	7.51	4.59	1.12	53.19
[Ne]$3s^2 3p^4 3d^5$	6.85	4.59	1.10	62.21
[Ne]$3s^2 3p^1 3d^8$	5.71	4.45	1.04	152.99
[Ne]$3s^2 3d^9$	5.50	4.39	1.03	183.87
[Ne]$3s^1 3d^{10}$	5.30	4.35	1.02	235.76

Ti The energies of the low lying configurations of hard-wall confined Ti are shown in fig. S49 for the neutral atom and in fig. S50 for the cation. The ground state of the unconfined Ti is [Ar]$4s^2 3d^2$. At $r_c = 4.9a_0$ the ground state changes to [Ar]$4s^1 3d^3$. Furthermore, at $r_c = 4.3a_0$ we see a ground state crossing to [Ar]$3d^4$. At $r_c = 1.0a_0$ we see a ground state crossing to [Ne]$3s^2 3p^4 3d^6$.

At $r_c = 3.2a_0$ the state [Ar]$3d^3 4p^1$ flips below the initial ground state. At $r_c = 2.8a_0$ the state [Ne]$3s^2 3p^5 3d^5$ flips below the initial ground state. At $r_c = 2.5a_0$ the state [Ne]$3s^1 3p^6 3d^5$ flips below the initial ground state. At $r_c = 2.1a_0$ the state [Ne]$3s^2 3p^3 3d^7$ flips below the initial ground state. At $r_c = 1.3a_0$ the state [Ar]$4s^1 3d^2 4p^1$ flips below the initial ground state. The ionization energy of the unconfined atom is $\Delta E_0 = 7.30$ eV. The studied configurations, atomic radii and excitation energies in the unconfined calculations are as follows:

configuration	r_ϵ	r_ρ	$r_{\max}$	ΔE
[Ar]$4s^2 3d^2$	6.37	5.02	2.90	0.00
[Ar]$4s^1 3d^3$	6.58	4.78	3.05	0.89
[Ar]$3d^4$	7.69	4.51	1.03	2.99
[Ar]$4s^1 3d^2 4p^1$	7.71	5.17	3.70	3.07
[Ar]$3d^3 4p^1$	8.51	4.89	4.07	3.57
[Ne]$3s^2 3p^5 3d^5$	6.88	4.51	1.01	35.98
[Ne]$3s^1 3p^6 3d^5$	6.81	4.45	1.00	58.57
[Ne]$3s^2 3p^4 3d^6$	6.33	4.45	0.99	69.43
[Ne]$3s^2 3p^3 3d^7$	5.93	4.39	0.97	103.27

V The energies of the low lying configurations of hard-wall confined V are shown in fig. S51 for the neutral atom and in fig. S52 for the cation. The ground state of the unconfined V is [Ar]$4s^2 3d^3$. At $r_c = 5.4a_0$ the ground state changes to [Ar]$4s^1 3d^4$. Furthermore, at $r_c = 4.3a_0$ we see a ground state crossing to [Ar]$3d^5$.

At $r_c = 3.2a_0$ the state [Ar]$3d^4 4p^1$ flips below the initial ground state. At $r_c = 2.7a_0$ the state [Ne]$3s^2 3p^5 3d^6$ flips below the initial ground state. At $r_c = 2.4a_0$ the state [Ne]$3s^1 3p^6 3d^6$ flips below the initial ground state. At $r_c = 2.3a_0$ the state [Ne]$3s^2 3p^4 3d^7$ flips below the initial ground state. The ionization energy of the unconfined atom is $\Delta E_0 = 7.07$ eV. The studied configurations, atomic radii and excitation energies in the unconfined calculations are as follows:

configuration	r_ϵ	r_ρ	$r_{\max}$	ΔE
[Ar]$4s^2 3d^3$	6.12	4.89	2.75	0.00
[Ar]$4s^1 3d^4$	6.22	4.67	2.89	0.34
[Ar]$3d^5$	6.95	4.45	0.93	2.18
[Ar]$3d^4 4p^1$	8.28	4.78	3.89	3.24
[Ne]$3s^2 3p^5 3d^6$	6.35	4.39	0.91	39.33
[Ne]$3s^1 3p^6 3d^6$	6.29	4.35	0.91	64.12
[Ne]$3s^2 3p^4 3d^7$	5.92	4.31	0.90	76.89

Cr The energies of the low lying configurations of hard-wall confined Cr are shown in fig. S53 for the neutral atom and in fig. S54 for the cation. The ground state of the unconfined Cr is [Ar]$4s^1 3d^5$. At $r_c = 4.3a_0$ the ground state changes to [Ar]$3d^6$.

At $r_c = 2.1a_0$ the state [Ne]$3s^2 3p^5 3d^7$ flips below the initial ground state. At $r_c = 1.9a_0$ the state [Ne]$3s^1 3p^6 3d^7$ flips below the initial ground state. At $r_c = 1.7a_0$ the state [Ne]$3s^2 3p^4 3d^8$ flips below the initial ground state. At $r_c = 1.2a_0$ the state [Ar]$3d^5 4p^1$ flips below the initial ground state. The ionization energy of the unconfined atom is $\Delta E_0 = 7.01$ eV. The studied configurations, atomic radii and excitation energies in the unconfined calculations are as follows:

configuration	r_ϵ	r_ρ	$r_{\max}$	ΔE
[Ar]$4s^1 3d^5$	5.95	4.59	2.76	0.00
[Ar]$4s^2 3d^4$	5.90	4.78	2.63	0.17
[Ar]$3d^6$	6.40	4.31	0.85	1.57
[Ar]$3d^5 4p^1$	8.11	4.67	3.74	3.10
[Ne]$3s^2 3p^5 3d^7$	5.93	4.25	0.84	43.02
[Ne]$3s^1 3p^6 3d^7$	5.89	4.22	0.83	70.06
[Ne]$3s^2 3p^4 3d^8$	5.58	4.20	0.83	84.82

Mn The energies of the low lying configurations of hard-wall confined Mn are shown in fig. S55 for the neutral atom and in fig. S56 for the cation. The ground state of the unconfined Mn is [Ar]$4s^1 3d^6$. At $r_c = 4.3a_0$ the ground state changes to [Ar]$3d^7$.

At $r_c = 2.0a_0$ the state [Ne]$3s^2 3p^5 3d^8$ flips below the initial ground state. At $r_c = 1.8a_0$ the state [Ne]$3s^1 3p^6 3d^8$ flips below the initial ground state. At $r_c = 1.1a_0$ the state [Ar]$3d^6 4p^1$ flips below the initial ground state. The ionization energy of the unconfined atom is $\Delta E_0 = 7.27$ eV. The studied configurations, atomic radii and excitation energies in the unconfined calculations are as follows:

configuration	r_ϵ	r_ρ	$r_{\max}$	ΔE
[Ar]$4s^1 3d^6$	5.72	4.45	2.65	0.00
[Ar]$4s^2 3d^5$	5.71	4.67	2.52	0.67
[Ar]$3d^7$	5.97	4.20	0.78	1.32
[Ar]$3d^6 4p^1$	7.98	4.59	3.62	3.30
[Ne]$3s^2 3p^5 3d^8$	5.59	4.16	0.77	47.18
[Ne]$3s^1 3p^6 3d^8$	5.55	4.15	0.77	76.55

Fe The energies of the low lying configurations of hard-wall confined Fe are shown in fig. S57 for the neutral atom and in fig. S58 for the cation. The ground state of the unconfined Fe is [Ar]$4s^1 3d^7$. At $r_c = 4.4a_0$ the ground state changes to [Ar]$3d^8$.

At $r_c = 2.0a_0$ the state [Ne]$3s^2 3p^5 3d^9$ flips below the initial ground state. At $r_c = 1.7a_0$ the state [Ne]$3s^1 3p^6 3d^9$ flips below the initial ground state. At $r_c = 1.1a_0$ the state [Ar]$3d^7 4p^1$

flips below the initial ground state. The ionization energy of the unconfined atom is $\Delta E_0 = 7.51$ eV. The studied configurations, atomic radii and excitation energies in the unconfined calculations are as follows:

configuration	r_ϵ	r_ρ	$r_{\max}$	ΔE
[Ar]$4s^1 3d^7$	5.53	4.35	2.55	0.00
[Ar]$3d^8$	5.63	4.13	0.72	1.08
[Ar]$4s^2 3d^6$	5.55	4.51	2.42	1.14
[Ar]$3d^7 4p^1$	7.87	4.51	3.51	3.50
[Ne]$3s^2 3p^5 3d^9$	5.31	4.08	0.72	51.51
[Ne]$3s^1 3p^6 3d^9$	5.28	4.08	0.72	83.27

Co The energies of the low lying configurations of hard-wall confined Co are shown in fig. S59 for the neutral atom and in fig. S60 for the cation. The ground state of the unconfined Co is [Ar]$4s^1 3d^8$. At $r_c = 4.6a_0$ the ground state changes to [Ar]$3d^9$.

At $r_c = 1.9a_0$ the state [Ne]$3s^2 3p^5 3d^{10}$ flips below the initial ground state. At $r_c = 1.6a_0$ the state [Ne]$3s^1 3p^6 3d^{10}$ flips below the initial ground state. At $r_c = 1.1a_0$ the state [Ar]$3d^8 4p^1$ flips below the initial ground state. The ionization energy of the unconfined atom is $\Delta E_0 = 7.75$ eV. The studied configurations, atomic radii and excitation energies in the unconfined calculations are as follows:

configuration	r_ϵ	r_ρ	$r_{\max}$	ΔE
[Ar]$4s^1 3d^8$	5.37	4.25	2.46	0.00
[Ar]$3d^9$	5.34	4.05	0.68	0.84
[Ar]$4s^2 3d^7$	5.40	4.45	2.33	1.59
[Ar]$3d^8 4p^1$	7.79	4.45	3.43	3.69
[Ne]$3s^2 3p^5 3d^{10}$	5.07	4.02	0.68	56.00
[Ne]$3s^1 3p^6 3d^{10}$	5.04	4.02	0.68	90.22

Ni The energies of the low lying configurations of hard-wall confined Ni are shown in fig. S61 for the neutral atom and in fig. S62 for the cation. The ground state of the unconfined Ni is [Ar]$4s^1 3d^9$. At $r_c = 4.8a_0$ the ground state changes to [Ar]$3d^{10}$.

At $r_c = 1.4a_0$ the state [Ar]$3d^9 4f^1$ flips below the initial ground state. At $r_c = 1.2a_0$ the state [Ne]$3s^2 3p^5 3d^{10} 4f^1$ flips below the initial ground state. At $r_c = 1.0a_0$ the state [Ar]$3d^9 4p^1$ flips below the initial ground state. The ionization energy of the unconfined atom is $\Delta E_0 = 7.97$ eV. The studied configurations, atomic radii and excitation energies in the unconfined calculations are as follows:

configuration	r_ϵ	r_ρ	$r_{\max}$	ΔE
[Ar]$4s^1 3d^9$	5.22	4.17	2.38	0.00
[Ar]$3d^{10}$	5.09	3.98	0.65	0.61
[Ar]$4s^2 3d^8$	5.27	4.35	2.25	2.02
[Ar]$3d^9 4p^1$	7.72	4.39	3.35	3.87
[Ar]$3d^9 4f^1$	25.18	3.41	16.38	7.06
[Ne]$3s^2 3p^5 3d^{10} 4f^1$	25.19	3.41	16.39	67.69

Cu The energies of the low lying configurations of hard-wall confined Cu are shown in fig. S63 for the neutral atom and in fig. S64 for the cation. The ground state of the unconfined Cu is [Ar]$4s^1 3d^{10}$. At $r_c = 1.4a_0$ the ground state changes to [Ar]$3d^{10} 4f^1$.

At $r_c = 1.1a_0$ the state [Ar]$3d^{10} 4d^1$ flips below the initial ground state. At $r_c = 1.0a_0$ the state [Ar]$3d^{10} 4p^1$ flips below the initial ground state. The ionization energy of the unconfined atom is $\Delta E_0 = 8.18$ eV. The studied configurations, atomic radii and excitation energies in the unconfined calculations are as follows:

configuration	r_ϵ	r_ρ	$r_{\max}$	ΔE
[Ar]$4s^1 3d^{10}$	5.09	4.13	2.30	0.00
[Ar]$4s^2 3d^9$	5.14	4.28	2.18	2.45
[Ar]$3d^{10} 4p^1$	7.66	4.35	3.28	4.06
[Ar]$3d^{10} 4d^1$	16.25	3.41	9.06	6.61
[Ar]$3d^{10} 4f^1$	25.19	3.41	16.39	7.28

Zn The energies of the low lying configurations of hard-wall confined Zn are shown in fig. S65 for the neutral atom and in fig. S66 for the cation. The ground state of the unconfined Zn is [Ar]$4s^2 3d^{10}$. At $r_c = 1.3a_0$ the ground state changes to [Ar]$3d^{10} 4f^2$.

At $r_c = 1.3a_0$ the state [Ar]$4s^1 3d^{10} 4f^1$ flips below the initial ground state. At $r_c = 1.2a_0$ the state [Ar]$3d^{10} 4d^1 4f^1$ flips below the initial ground state. At $r_c = 1.2a_0$ the state [Ar]$3d^{10} 4p^1 4f^1$ flips below the initial ground state. At $r_c = 1.1a_0$ the state [Ar]$4s^1 3d^{10} 4d^1$ flips below the initial ground state. At $r_c = 1.0a_0$ the state [Ar]$4s^1 3d^{10} 4p^1$ flips below the initial ground state. At $r_c = 1.0a_0$ the state [Ar]$3d^{10} 4p^2$ flips below the initial ground state. The ionization energy of the unconfined atom is $\Delta E_0 = 9.96$ eV. The studied configurations,

atomic radii and excitation energies in the unconfined calculations are as follows:

configuration	r_ϵ	r_ρ	$r_{\max}$	ΔE
[Ar]$4s^2 3d^{10}$	5.03	4.21	2.12	0.00
[Ar]$4s^1 3d^{10} 4p^1$	6.67	4.51	2.86	4.96
[Ar]$4s^1 3d^{10} 4d^1$	15.61	3.83	8.38	8.31
[Ar]$4s^1 3d^{10} 4f^1$	25.13	3.71	16.32	9.05
[Ar]$3d^{10} 4p^2$	6.90	4.89	2.73	10.28
[Ar]$3d^{10} 4p^1 4f^1$	24.89	4.02	16.05	15.18
[Ar]$3d^{10} 4d^1 4f^1$	21.86	3.89	12.29	21.30
[Ar]$3d^{10} 4f^2$	18.57	2.96	9.54	23.39

Ga The energies of the low lying configurations of hard-wall confined Ga are shown in fig. S67 for the neutral atom and in fig. S68 for the cation. The ground state of the unconfined Ga is [Ar]$4s^2 3d^{10} 4p^1$. At $r_c = 1.3a_0$ the ground state changes to [Ar]$4s^2 3d^{10} 4f^1$. Furthermore, at $r_c = 1.2a_0$ we see a ground state crossing to [Ar]$3d^{10} 4f^3$.

At $r_c = 1.3a_0$ the state [Ar]$4s^1 3d^{10} 4f^2$ flips below the initial ground state. At $r_c = 1.3a_0$ the state [Ar]$3d^{10} 4p^1 4f^2$ flips below the initial ground state. At $r_c = 1.2a_0$ the state [Ar]$3d^{10} 4d^1 4f^2$ flips below the initial ground state. At $r_c = 1.1a_0$ the state [Ar]$4s^2 3d^{10} 4d^1$ flips below the initial ground state. The ionization energy of the unconfined atom is $\Delta E_0 =$ 5.91 eV. The studied configurations, atomic radii and excitation energies in the unconfined calculations are as follows:

configuration	r_ϵ	r_ρ	$r_{\max}$	ΔE
[Ar]$4s^2 3d^{10} 4p^1$	5.92	4.51	2.53	0.00
[Ar]$4s^2 3d^{10} 5s^1$	11.14	3.98	6.41	2.96
[Ar]$4s^2 3d^{10} 4d^1$	15.24	3.98	7.96	4.22
[Ar]$4s^2 3d^{10} 4f^1$	25.13	3.83	16.32	5.00
[Ar]$4s^1 3d^{10} 4p^2$	6.20	4.78	2.44	6.32
[Ar]$4s^1 3d^{10} 4f^2$	18.49	3.49	9.42	22.03
[Ar]$3d^{10} 4p^1 4f^2$	18.39	3.83	9.24	30.06
[Ar]$3d^{10} 4d^1 4f^2$	17.57	5.17	8.00	39.64
[Ar]$3d^{10} 4f^3$	15.43	5.69	6.66	43.83

Ge The energies of the low lying configurations of hard-wall confined Ge are shown in fig. S69 for the neutral atom and in fig. S70 for the cation. The ground state of the unconfined Ge is [Ar]$4s^2 3d^{10} 4p^2$. At $r_c = 1.3a_0$ the ground state changes to [Ar]$4s^2 3d^{10} 4f^2$. Furthermore, at $r_c = 1.2a_0$ we see a ground state crossing to [Ar]$3d^{10} 4f^4$.

At $r_c = 1.3a_0$ the state [Ar]$4s^1 3d^{10} 4f^3$ flips below the initial ground state. At $r_c = 1.3a_0$ the state [Ar]$4s^2 3d^{10} 4p^1 4f^1$ flips below the initial ground state. At $r_c = 1.2a_0$ the state [Ar]$3d^{10} 4d^1 4f^3$ flips below the initial ground state. At $r_c = 1.0a_0$ the state [Ar]$4s^2 3d^{10} 4p^1 4d^1$ flips below the initial ground state. The ionization energy of the unconfined atom is $\Delta E_0 =$ 7.66 eV. The studied configurations, atomic radii and excitation energies in the unconfined calculations are as follows:

configuration	r_ϵ	r_ρ	$r_{\max}$	ΔE
[Ar]$4s^2 3d^{10} 4p^2$	5.63	4.51	2.21	0.00
[Ar]$4s^2 3d^{10} 4p^1 5s^1$	10.25	4.51	5.76	4.38
[Ar]$4s^2 3d^{10} 4p^1 4d^1$	14.37	4.23	6.86	5.83
[Ar]$4s^2 3d^{10} 4p^1 4f^1$	25.06	3.98	16.24	6.75
[Ar]$4s^1 3d^{10} 4p^3$	5.70	4.67	2.16	7.66
[Ar]$4s^2 3d^{10} 4f^2$	18.48	3.57	9.38	18.36
[Ar]$4s^1 3d^{10} 4f^3$	15.39	5.69	6.52	43.57
[Ar]$3d^{10} 4d^1 4f^3$	15.05	6.11	5.81	66.29
[Ar]$3d^{10} 4f^4$	13.59	6.84	5.07	73.01

As The energies of the low lying configurations of hard-wall confined As are shown in fig. S71 for the neutral atom and in fig. S72 for the cation. The ground state of the unconfined As is [Ar]$4s^2 3d^{10} 4p^3$. At $r_c = 1.2a_0$ the ground state changes to [Ar]$4s^1 3d^{10} 4f^4$. Furthermore, at $r_c = 1.1a_0$ we see a ground state crossing to [Ar]$3d^{10} 4f^5$.

At $r_c = 1.2a_0$ the state [Ar]$3d^{10} 4d^1 4f^4$ flips below the initial ground state. At $r_c = 1.2a_0$ the state [Ar]$4s^2 3d^{10} 4f^3$ flips below the initial ground state. At $r_c = 1.2a_0$ the state [Ar]$4s^2 3d^{10} 4p^2 4f^1$ flips below the initial ground state. At $r_c = 1.0a_0$ the state [Ar]$4s^2 3d^{10} 4p^2 4d^1$ flips below the initial ground state. The ionization energy of the unconfined atom is $\Delta E_0 =$ 9.35 eV. The studied configurations, atomic radii and excitation energies in the unconfined calculations are as follows:

configuration	r_ϵ	r_ρ	$r_{\max}$	ΔE
$[Ar]4s^2 3d^{10} 4p^3$	5.26	4.45	1.98	0.00
$[Ar]4s^2 3d^{10} 4p^2 5s^1$	9.65	4.78	5.32	5.82
$[Ar]4s^2 3d^{10} 4p^2 4d^1$	13.94	4.31	6.17	7.44
$[Ar]4s^2 3d^{10} 4p^2 4f^1$	25.07	4.02	16.26	8.43
$[Ar]4s^1 3d^{10} 4p^4$	5.28	4.51	1.95	8.99
$[Ar]4s^2 3d^{10} 4f^3$	15.39	5.69	6.46	40.96
$[Ar]4s^1 3d^{10} 4f^4$	13.59	6.84	4.94	74.39
$[Ar]3d^{10} 4d^1 4f^4$	13.45	6.58	4.53	102.02
$[Ar]3d^{10} 4f^5$	12.37	6.84	4.07	111.55

Se The energies of the low lying configurations of hard-wall confined Se are shown in fig. S73 for the neutral atom and in fig. S74 for the cation. The ground state of the unconfined Se is $[Ar]4s^2 3d^{10} 4p^4$. At $r_c = 1.2a_0$ the ground state changes to $[Ar]4s^2 3d^{10} 4f^4$. Furthermore, at $r_c = 1.1a_0$ we see a ground state crossing to $[Ar]3d^{10} 4f^6$.

At $r_c = 1.2a_0$ the state $[Ar]4s^1 3d^{10} 4f^5$ flips below the initial ground state. At $r_c = 1.2a_0$ the state $[Ar]4s^2 3d^{10} 4p^1 4f^3$ flips below the initial ground state. At $r_c = 1.2a_0$ the state $[Ar]4s^2 3d^{10} 4p^3 4f^1$ flips below the initial ground state. At $r_c = 1.1a_0$ the state $[Ar]3d^{10} 4d^1 4f^5$ flips below the initial ground state. At $r_c = 1.0a_0$ the state $[Ar]4s^2 3d^{10} 4p^3 4d^1$ flips below the initial ground state. The ionization energy of the unconfined atom is $\Delta E_0 = 11.03$ eV. The studied configurations, atomic radii and excitation energies in the unconfined calculations are as follows:

configuration	r_ϵ	r_ρ	$r_{\max}$	ΔE
$[Ar]4s^2 3d^{10} 4p^4$	4.93	4.31	1.81	0.00
$[Ar]4s^2 3d^{10} 4p^3 5s^1$	9.20	4.78	4.98	7.30
$[Ar]4s^2 3d^{10} 4p^3 4d^1$	13.76	4.25	5.70	9.08
$[Ar]4s^2 3d^{10} 4p^3 4f^1$	25.11	3.98	16.29	10.12
$[Ar]4s^1 3d^{10} 4p^5$	4.93	4.35	1.79	10.31
$[Ar]4s^2 3d^{10} 4p^1 4f^3$	15.37	5.69	6.37	48.01
$[Ar]4s^2 3d^{10} 4f^4$	13.60	6.84	4.86	73.43
$[Ar]4s^1 3d^{10} 4f^5$	12.40	6.84	3.95	115.05
$[Ar]3d^{10} 4d^1 4f^5$	12.35	6.58	3.69	147.43
$[Ar]3d^{10} 4f^6$	11.50	6.84	3.39	159.95

Br The energies of the low lying configurations of hard-wall confined Br are shown in fig. S75 for the neutral atom and in fig. S76 for the cation. The ground state of the unconfined Br is $[Ar]4s^2 3d^{10} 4p^5$. At $r_c = 1.2a_0$ the ground state changes to $[Ar]4s^2 3d^{10} 4p^4 4f^1$. Furthermore, at $r_c = 1.1a_0$ we see a ground state crossing to $[Ar]3d^{10} 4f^7$.

At $r_c = 1.2a_0$ the state $[Ar]4s^2 3d^{10} 4p^3 4f^2$ flips below the initial ground state. At $r_c = 1.1a_0$ the state $[Ar]4s^1 3d^{10} 4f^6$ flips below the initial ground state. At $r_c = 1.1a_0$ the state $[Ar]3d^{10} 4p^1 4f^6$ flips below the initial ground state. The ionization energy of the unconfined atom is $\Delta E_0 = 12.73$ eV. The studied configurations, atomic radii and excitation energies in the unconfined calculations are as follows:

configuration	r_ϵ	r_ρ	$r_{\max}$	ΔE
$[Ar]4s^2 3d^{10} 4p^5$	4.63	4.17	1.67	0.00
$[Ar]4s^2 3d^{10} 4p^4 5s^1$	8.84	4.78	4.72	8.82
$[Ar]4s^2 3d^{10} 4p^4 4d^1$	13.72	4.16	5.38	10.75
$[Ar]4s^1 3d^{10} 4p^6$	4.64	4.20	1.65	11.64
$[Ar]4s^2 3d^{10} 4p^4 4f^1$	25.14	3.89	16.33	11.82
$[Ar]4s^2 3d^{10} 4p^3 4f^2$	18.43	3.71	9.30	30.61
$[Ar]4s^1 3d^{10} 4f^6$	11.56	6.58	3.28	166.02
$[Ar]3d^{10} 4p^1 4f^6$	11.57	6.58	3.23	180.79
$[Ar]3d^{10} 4f^7$	10.87	6.58	2.90	218.67

Kr The energies of the low lying configurations of hard-wall confined Kr are shown in fig. S77 for the neutral atom and in fig. S78 for the cation. The ground state of the unconfined Kr is $[Ar]4s^2 3d^{10} 4p^6$. At $r_c = 1.1a_0$ the ground state changes to $[Ar]4s^1 3d^{10} 4f^7$. Furthermore, at $r_c = 1.0a_0$ we see a ground state crossing to $[Ar]3d^{10} 4f^8$.

At $r_c = 1.1a_0$ the state $[Ar]3d^{10} 4p^1 4f^7$ flips below the initial ground state. At $r_c = 1.1a_0$ the state $[Ar]4s^2 3d^{10} 4p^5 4f^1$ flips below the initial ground state. At $r_c = 1.1a_0$ the state $[Ar]4s^2 3d^{10} 4p^4 4f^2$ flips below the initial ground state. The ionization energy of the unconfined atom is $\Delta E_0 = 14.45$ eV. The studied configurations, atomic radii and excitation energies in the unconfined calculations are as follows:

configuration	r_ϵ	r_ρ	$r_{\max}$	ΔE
[Ar]$4s^2 3d^{10} 4p^6$	4.38	4.08	1.55	0.00
[Ar]$4s^2 3d^{10} 4p^5 5s^1$	8.55	4.67	4.50	10.40
[Ar]$4s^2 3d^{10} 4p^5 4d^1$	13.76	4.08	5.22	12.46
[Ar]$4s^2 3d^{10} 4p^5 4f^1$	25.16	3.78	16.35	13.55
[Ar]$4s^1 3d^{10} 4p^6 4d^1$	13.53	4.11	4.18	25.96
[Ar]$4s^2 3d^{10} 4p^4 4f^2$	18.45	3.64	9.33	34.74
[Ar]$4s^1 3d^{10} 4f^7$	10.95	6.58	2.80	227.70
[Ar]$3d^{10} 4p^1 4f^7$	10.97	6.58	2.76	244.06
[Ar]$3d^{10} 4f^8$	10.39	6.34	2.53	288.10

Rb The energies of the low lying configurations of hard-wall confined Rb are shown in fig. S79 for the neutral atom and in fig. S80 for the cation. The ground state of the unconfined Rb is [Kr]$5s^1$. At $r_c = 4.9a_0$ the ground state changes to [Kr]$4d^1$. Furthermore, at $r_c = 1.2a_0$ we see a ground state crossing to [Kr]$4f^1$. At $r_c = 1.1a_0$ we see a ground state crossing to [Ar]$4s^2 3d^{10} 4p^5 4f^2$. Moreover, at $r_c = 1.0a_0$ we see a ground state crossing to [Ar]$3d^{10} 4f^9$.

At $r_c = 2.9a_0$ the state [Ar]$4s^2 3d^{10} 4p^5 4d^2$ flips below the initial ground state. At $r_c = 1.3a_0$ the state [Ar]$4s^1 3d^{10} 4f^8$ flips below the initial ground state. At $r_c = 1.2a_0$ the state [Ar]$3d^{10} 4p^1 4f^8$ flips below the initial ground state. At $r_c = 1.1a_0$ the state [Kr]$5p^1$ flips below the initial ground state. The ionization energy of the unconfined atom is $\Delta E_0 = 4.20$ eV. The studied configurations, atomic radii and excitation energies in the unconfined calculations are as follows:

configuration	r_ϵ	r_ρ	$r_{\max}$	ΔE
[Kr]$5s^1$	8.30	4.59	4.32	0.00
[Kr]$5p^1$	11.29	3.98	6.05	1.54
[Kr]$4d^1$	13.86	3.98	5.28	2.21
[Kr]$4f^1$	25.18	3.71	16.38	3.29
[Ar]$4s^2 3d^{10} 4p^5 4d^2$	9.94	4.89	2.52	19.61
[Ar]$4s^2 3d^{10} 4p^5 4f^2$	18.47	3.57	9.36	26.90
[Ar]$4s^1 3d^{10} 4f^8$	10.49	6.34	2.44	288.48
[Ar]$3d^{10} 4p^1 4f^8$	10.51	6.34	2.41	306.40
[Ar]$3d^{10} 4f^9$	10.02	6.11	2.24	356.62

Sr The energies of the low lying configurations of hard-wall confined Sr are shown in fig. S81 for the neutral atom and in fig. S82 for the cation. The ground state of the unconfined Sr is [Kr]$5s^2$. At $r_c = 4.9a_0$ the ground state changes to [Kr]$5s^1 4d^1$. Furthermore, at $r_c = 4.8a_0$ we see a ground state crossing to [Kr]$4d^2$. At $r_c = 1.2a_0$ we see a ground state crossing to [Kr]$4f^2$. Moreover, at $r_c = 1.0a_0$ we see a ground state crossing to [Ar]$3d^{10} 4f^{10}$.

At $r_c = 3.4a_0$ the state [Kr]$4d^1 4f^1$ flips below the initial ground state. At $r_c = 3.4a_0$ the state [Ar]$4s^2 3d^{10} 4p^5 4d^3$ flips below the initial ground state. At $r_c = 1.5a_0$ the state [Ar]$4s^2 3d^{10} 4f^8$ flips below the initial ground state. At $r_c = 1.4a_0$ the state [Ar]$4s^1 3d^{10} 4f^9$ flips below the initial ground state. At $r_c = 1.1a_0$ the state [Kr]$5s^1 5p^1$ flips below the initial ground state. The ionization energy of the unconfined atom is $\Delta E_0 = 5.97$ eV. The studied configurations, atomic radii and excitation energies in the unconfined calculations are as follows:

configuration	r_ϵ	r_ρ	$r_{\max}$	ΔE
[Kr]$5s^2$	7.61	5.50	3.68	0.00
[Kr]$5s^1 4d^1$	8.25	5.17	3.78	2.06
[Kr]$5s^1 5p^1$	9.10	5.50	4.72	2.27
[Kr]$4d^2$	9.89	4.78	2.30	4.28
[Kr]$4d^1 4f^1$	24.73	4.02	15.87	6.48
[Kr]$4f^2$	18.49	3.49	9.40	11.95
[Ar]$4s^2 3d^{10} 4p^5 4d^3$	8.29	5.02	2.05	25.27
[Ar]$4s^2 3d^{10} 4f^8$	10.59	6.34	2.36	279.68
[Ar]$4s^1 3d^{10} 4f^9$	10.14	6.11	2.16	353.54
[Ar]$3d^{10} 4f^{10}$	9.75	6.11	2.01	429.44

Y The energies of the low lying configurations of hard-wall confined Y are shown in fig. S83 for the neutral atom and in fig. S84 for the cation. The ground state of the unconfined Y is [Kr]$5s^2 4d^1$. At $r_c = 5.1a_0$ the ground state changes to [Kr]$5s^1 4d^2$. Furthermore, at $r_c = 4.9a_0$ we see a ground state crossing to [Kr]$4d^3$. At $r_c = 1.2a_0$ we see a ground state crossing to [Kr]$4d^2 4f^1$. Moreover, at $r_c = 1.1a_0$ we see a ground state crossing to [Kr]$4d^1 4f^2$. At $r_c = 1.0a_0$ we see a ground state crossing to [Ar]$4s^2 3d^{10} 4f^9$.

At $r_c = 3.5a_0$ the state [Kr]$4d^2 5p^1$ flips below the initial ground state. At $r_c = 3.3a_0$ the state [Ar]$4s^2 3d^{10} 4p^5 4d^4$ flips below the initial ground state. At $r_c = 1.4a_0$ the state [Ar]$4s^2 3d^{10} 4p^1 4f^8$ flips below the initial ground state. At $r_c = 1.3a_0$ the state [Ar]$4s^1 3d^{10} 4f^{10}$ flips below the initial ground state. The ionization energy of

the unconfined atom is $\Delta E_0 = 6.65$ eV. The studied configurations, atomic radii and excitation energies in the unconfined calculations are as follows:

configuration	r_ϵ	r_ρ	$r_{\max}$	ΔE
$[\text{Kr}]5s^2 4d^1$	7.15	5.33	3.39	0.00
$[\text{Kr}]5s^1 4d^2$	7.39	5.17	3.50	1.46
$[\text{Kr}]5s^2 5p^1$	7.94	5.69	4.04	2.22
$[\text{Kr}]4d^3$	8.19	4.89	1.90	3.34
$[\text{Kr}]4d^2 5p^1$	9.12	5.17	4.60	3.83
$[\text{Kr}]4d^2 4f^1$	24.80	4.15	15.95	6.44
$[\text{Kr}]4d^1 4f^2$	18.39	3.71	9.22	13.65
$[\text{Ar}]4s^2 3d^{10} 4p^5 4d^4$	7.29	4.89	1.79	27.59
$[\text{Ar}]4s^2 3d^{10} 4p^1 4f^8$	10.71	6.34	2.27	280.47
$[\text{Ar}]4s^2 3d^{10} 4f^9$	10.26	6.11	2.08	339.70
$[\text{Ar}]4s^1 3d^{10} 4f^{10}$	9.88	6.11	1.94	421.56

Zr The energies of the low lying configurations of hard-wall confined Zr are shown in fig. S85 for the neutral atom and in fig. S86 for the cation. The ground state of the unconfined Zr is $[\text{Kr}]5s^2 4d^2$. At $r_c = 5.4a_0$ the ground state changes to $[\text{Kr}]5s^1 4d^3$. Furthermore, at $r_c = 5.0a_0$ we see a ground state crossing to $[\text{Kr}]4d^4$. At $r_c = 1.1a_0$ we see a ground state crossing to $[\text{Kr}]4d^2 4f^2$. Moreover, at $r_c = 1.0a_0$ we see a ground state crossing to $[\text{Ar}]4s^2 3d^{10} 4f^{10}$.

At $r_c = 3.4a_0$ the state $[\text{Kr}]4d^3 5p^1$ flips below the initial ground state. At $r_c = 3.2a_0$ the state $[\text{Kr}]4d^3 4f^1$ flips below the initial ground state. At $r_c = 3.1a_0$ the state $[\text{Ar}]4s^2 3d^{10} 4p^5 4d^5$ flips below the initial ground state. At $r_c = 1.4a_0$ the state $[\text{Ar}]4s^2 3d^{10} 4p^1 4f^9$ flips below the initial ground state. At $r_c = 1.4a_0$ the state $[\text{Ar}]4s^2 3d^{10} 4p^2 4f^8$ flips below the initial ground state. The ionization energy of the unconfined atom is $\Delta E_0 = 7.05$ eV. The studied configurations, atomic radii and excitation energies in the unconfined calculations are as follows:

configuration	r_ϵ	r_ρ	$r_{\max}$	ΔE
$[\text{Kr}]5s^2 4d^2$	6.77	5.33	3.19	0.00
$[\text{Kr}]5s^1 4d^3$	6.85	5.02	3.30	0.76
$[\text{Kr}]4d^4$	7.18	4.89	1.68	2.19
$[\text{Kr}]4d^3 5p^1$	8.72	5.17	4.33	3.36
$[\text{Kr}]4d^3 4f^1$	24.90	4.15	16.06	6.13
$[\text{Kr}]4d^2 4f^2$	18.37	3.83	9.18	14.93
$[\text{Ar}]4s^2 3d^{10} 4p^5 4d^5$	6.60	4.78	1.61	29.55
$[\text{Ar}]4s^2 3d^{10} 4p^2 4f^8$	10.82	6.34	2.20	276.02
$[\text{Ar}]4s^2 3d^{10} 4p^1 4f^9$	10.40	6.11	2.00	340.17
$[\text{Ar}]4s^2 3d^{10} 4f^{10}$	10.02	5.90	1.87	406.05

Nb The energies of the low lying configurations of hard-wall confined Nb are shown in fig. S87 for the neutral atom and in fig. S88 for the cation. The ground state of the unconfined Nb is $[\text{Kr}]5s^2 4d^3$. At $r_c = 7.7a_0$ the ground state changes to $[\text{Kr}]5s^1 4d^4$. Furthermore, at $r_c = 5.3a_0$ we see a ground state crossing to $[\text{Kr}]4d^5$. At $r_c = 1.1a_0$ we see a ground state crossing to $[\text{Kr}]4f^5$.

At $r_c = 3.4a_0$ the state $[\text{Kr}]4d^4 5p^1$ flips below the initial ground state. At $r_c = 3.1a_0$ the state $[\text{Kr}]4d^4 4f^1$ flips below the initial ground state. At $r_c = 3.1a_0$ the state $[\text{Ar}]4s^2 3d^{10} 4p^5 4d^6$ flips below the initial ground state. At $r_c = 2.5a_0$ the state $[\text{Kr}]4d^3 4f^2$ flips below the initial ground state. At $r_c = 1.7a_0$ the state $[\text{Ar}]4s^2 3d^{10} 4p^5 4f^6$ flips below the initial ground state. At $r_c = 1.5a_0$ the state $[\text{Ar}]4s^2 3d^{10} 4p^4 4f^7$ flips below the initial ground state. The ionization energy of the unconfined atom is $\Delta E_0 = 6.59$ eV. The studied configurations, atomic radii and excitation energies in the unconfined calculations are as follows:

configuration	r_ϵ	r_ρ	$r_{\max}$	ΔE
$[\text{Kr}]5s^2 4d^3$	6.49	5.17	3.04	0.00
$[\text{Kr}]5s^1 4d^4$	6.48	4.89	3.15	0.02
$[\text{Kr}]4d^5$	6.49	4.67	1.52	0.93
$[\text{Kr}]4d^4 5p^1$	8.47	5.02	4.13	2.80
$[\text{Kr}]4d^4 4f^1$	24.98	4.08	16.15	5.67
$[\text{Kr}]4d^3 4f^2$	18.38	3.83	9.20	15.98
$[\text{Ar}]4s^2 3d^{10} 4p^5 4d^6$	6.08	4.67	1.48	31.32
$[\text{Kr}]4f^5$	12.65	6.58	3.64	74.07
$[\text{Ar}]4s^2 3d^{10} 4p^5 4f^6$	11.93	6.58	2.91	135.08
$[\text{Ar}]4s^2 3d^{10} 4p^4 4f^7$	11.38	6.34	2.43	199.44

Mo The energies of the low lying configurations of hard-wall confined Mo are shown in fig. S89 for the neutral atom and in fig. S90 for the cation. The ground state of the unconfined Mo is [Kr]$5s^14d^5$. At $r_c = 6.0a_0$ the ground state changes to [Kr]$4d^6$. Furthermore, at $r_c = 1.0a_0$ we see a ground state crossing to [Kr]$4f^6$.

At $r_c = 2.5a_0$ the state [Ar]$4s^23d^{10}4p^54d^7$ flips below the initial ground state. At $r_c = 2.4a_0$ the state [Kr]$4d^54f^1$ flips below the initial ground state. At $r_c = 1.9a_0$ the state [Kr]$4d^44f^2$ flips below the initial ground state. At $r_c = 1.4a_0$ the state [Kr]$4d^14f^5$ flips below the initial ground state. At $r_c = 1.3a_0$ the state [Ar]$4s^23d^{10}4p^54f^7$ flips below the initial ground state. At $r_c = 1.0a_0$ the state [Kr]$4d^55p^1$ flips below the initial ground state. The ionization energy of the unconfined atom is $\Delta E_0 = 6.80$ eV. The studied configurations, atomic radii and excitation energies in the unconfined calculations are as follows:

configuration	r_ϵ	r_ρ	$r_{\max}$	ΔE
[Kr]$5s^14d^5$	6.20	4.78	3.02	0.00
[Kr]$4d^6$	5.97	4.59	1.40	0.36
[Kr]$5s^24d^4$	6.26	5.02	2.91	0.76
[Kr]$4d^55p^1$	8.30	4.89	3.98	2.95
[Kr]$4d^54f^1$	25.03	4.02	16.21	5.88
[Kr]$4d^44f^2$	18.40	3.78	9.23	17.64
[Ar]$4s^23d^{10}4p^54d^7$	5.66	4.51	1.37	33.73
[Kr]$4d^14f^5$	12.71	6.58	3.55	82.93
[Kr]$4f^6$	11.96	6.58	2.90	111.18
[Ar]$4s^23d^{10}4p^54f^7$	11.44	6.34	2.40	180.46

Tc The energies of the low lying configurations of hard-wall confined Tc are shown in fig. S91 for the neutral atom and in fig. S92 for the cation. The ground state of the unconfined Tc is [Kr]$4d^7$. At $r_c = 1.0a_0$ the ground state changes to [Kr]$4f^7$.

At $r_c = 1.0a_0$ the state [Kr]$4d^14f^6$ flips below the initial ground state. At $r_c = 1.0a_0$ the state [Kr]$4d^24f^5$ flips below the initial ground state. At $r_c = 1.0a_0$ the state [Kr]$4d^64f^1$ flips below the initial ground state. At $r_c = 1.0a_0$ the state [Kr]$4d^54f^2$ flips below the initial ground state. The ionization energy of the unconfined atom is $\Delta E_0 = 7.21$ eV. The studied configurations, atomic radii and excitation energies in the unconfined calculations are as follows:

configuration	r_ϵ	r_ρ	$r_{\max}$	ΔE
[Kr]$4d^7$	5.56	4.45	1.30	0.00
[Kr]$5s^14d^6$	5.98	4.67	2.92	0.23
[Kr]$5s^24d^5$	6.08	4.89	2.80	1.80
[Kr]$4d^65p^1$	8.19	4.78	3.86	3.32
[Kr]$4d^64f^1$	25.08	3.94	16.26	6.30
[Kr]$4d^54f^2$	18.42	3.71	9.28	19.47
[Ar]$4s^23d^{10}4p^54d^8$	5.32	4.39	1.28	36.34
[Kr]$4d^24f^5$	12.76	6.58	3.50	91.98
[Kr]$4d^14f^6$	12.05	6.34	2.81	123.16
[Kr]$4f^7$	11.49	6.34	2.37	156.47

Ru The energies of the low lying configurations of hard-wall confined Ru are shown in fig. S93 for the neutral atom and in fig. S94 for the cation. The ground state of the unconfined Ru is [Kr]$4d^8$. At $r_c = 1.0a_0$ the ground state changes to [Kr]$4d^24f^6$.

At $r_c = 1.0a_0$ the state [Kr]$4d^34f^5$ flips below the initial ground state. At $r_c = 1.0a_0$ the state [Kr]$4d^14f^7$ flips below the initial ground state. At $r_c = 1.0a_0$ the state [Kr]$4d^74f^1$ flips below the initial ground state. The ionization energy of the unconfined atom is $\Delta E_0 = 7.98$ eV. The studied configurations, atomic radii and excitation energies in the unconfined calculations are as follows:

configuration	r_ϵ	r_ρ	$r_{\max}$	ΔE
[Kr]$4d^8$	5.22	4.28	1.22	0.00
[Kr]$5s^14d^7$	5.80	4.59	2.83	0.85
[Kr]$5s^24d^6$	5.92	4.78	2.71	3.25
[Kr]$4d^75p^1$	8.11	4.78	3.76	4.06
[Kr]$4d^74f^1$	25.11	3.89	16.30	7.07
[Ar]$4s^23d^{10}4p^54d^9$	5.03	4.22	1.20	39.30
[Kr]$4d^34f^5$	12.80	6.58	3.47	101.35
[Kr]$4d^24f^6$	12.12	6.34	2.75	135.53
[Kr]$4d^14f^7$	11.61	6.34	2.28	171.95

Rh The energies of the low lying configurations of hard-wall confined Rh are shown in fig. S95 for the neutral atom and in fig. S96 for the cation. The ground state of the unconfined Rh is [Kr]$4d^9$. At $r_c = 1.0a_0$ the ground state changes to [Kr]$4d^84f^1$.

The ionization energy of the unconfined atom is $\Delta E_0 = 8.75$ eV. The studied configurations,

atomic radii and excitation energies in the unconfined calculations are as follows:

configuration	r_ϵ	r_ρ	$r_{\max}$	ΔE
[Kr]$4d^9$	4.94	4.16	1.15	0.00
[Kr]$5s^1 4d^8$	5.66	4.51	2.75	1.48
[Kr]$5s^2 4d^7$	5.79	4.78	2.63	4.73
[Kr]$4d^8 5p^1$	8.05	4.67	3.68	4.81
[Kr]$4d^8 4f^1$	25.14	3.78	16.33	7.84
[Kr]$4d^7 4f^2$	18.46	3.57	9.35	23.76
[Ar]$4s^2 3d^{10} 4p^5 4d^{10}$	4.78	4.13	1.14	42.26

Pd The energies of the low lying configurations of hard-wall confined Pd are shown in fig. S97 for the neutral atom and in fig. S98 for the cation. The ground state of the unconfined Pd is [Kr]$4d^{10}$. We do not observe any ground state crossing for Pd in the considered confinement radii.

The ionization energy of the unconfined atom is $\Delta E_0 = 9.52$ eV. The studied configurations, atomic radii and excitation energies in the unconfined calculations are as follows:

configuration	r_ϵ	r_ρ	$r_{\max}$	ΔE
[Kr]$4d^{10}$	4.69	4.08	1.09	0.00
[Kr]$5s^1 4d^9$	5.53	4.39	2.67	2.13
[Kr]$4d^9 5p^1$	8.01	4.59	3.62	5.57
[Kr]$5s^2 4d^8$	5.67	4.67	2.55	6.25
[Kr]$4d^9 4f^1$	25.16	3.71	16.35	8.62
[Kr]$4d^8 4f^2$	18.48	3.49	9.39	25.88
[Ar]$4s^2 3d^{10} 4p^5 4d^{10} 4f^1$	25.17	3.71	16.36	54.52

Ag The energies of the low lying configurations of hard-wall confined Ag are shown in fig. S99 for the neutral atom and in fig. S100 for the cation. The ground state of the unconfined Ag is [Kr]$5s^1 4d^{10}$. At $r_c = 2.1a_0$ the ground state changes to [Kr]$4d^{10} 4f^1$.

At $r_c = 1.6a_0$ the state [Kr]$4d^9 4f^2$ flips below the initial ground state. At $r_c = 1.5a_0$ the state [Ar]$4s^2 3d^{10} 4p^5 4d^{10} 4f^2$ flips below the initial ground state. At $r_c = 1.4a_0$ the state [Kr]$4d^8 4f^3$ flips below the initial ground state. The ionization energy of the unconfined atom is $\Delta E_0 = 7.50$ eV. The studied configurations, atomic radii and excitation energies in the unconfined calculations are as follows:

configuration	r_ϵ	r_ρ	$r_{\max}$	ΔE
[Kr]$5s^1 4d^{10}$	5.43	4.31	2.61	0.00
[Kr]$4d^{10} 5p^1$	7.98	4.51	3.56	3.53
[Kr]$5s^2 4d^9$	5.57	4.59	2.49	5.01
[Kr]$4d^{10} 4f^1$	25.18	3.64	16.37	6.59
[Kr]$4d^9 4f^2$	18.50	3.41	9.41	25.18
[Kr]$4d^8 4f^3$	15.42	5.69	6.40	52.42
[Ar]$4s^2 3d^{10} 4p^5 4d^{10} 4f^2$	18.50	3.41	9.41	74.92

Cd The energies of the low lying configurations of hard-wall confined Cd are shown in fig. S101 for the neutral atom and in fig. S102 for the cation. The ground state of the unconfined Cd is [Kr]$5s^2 4d^{10}$. At $r_c = 2.0a_0$ the ground state changes to [Kr]$4d^{10} 4f^2$.

At $r_c = 2.0a_0$ the state [Kr]$5s^1 4d^{10} 4f^1$ flips below the initial ground state. At $r_c = 1.8a_0$ the state [Kr]$4d^9 4f^3$ flips below the initial ground state. At $r_c = 1.8a_0$ the state [Kr]$4d^{10} 5p^1 4f^1$ flips below the initial ground state. At $r_c = 1.6a_0$ the state [Kr]$4d^8 4f^4$ flips below the initial ground state. At $r_c = 1.6a_0$ the state [Ar]$4s^2 3d^{10} 4p^5 4d^{10} 4f^3$ flips below the initial ground state. The ionization energy of the unconfined atom is $\Delta E_0 = 9.07$ eV. The studied configurations, atomic radii and excitation energies in the unconfined calculations are as follows:

configuration	r_ϵ	r_ρ	$r_{\max}$	ΔE
[Kr]$5s^2 4d^{10}$	5.47	4.51	2.43	0.00
[Kr]$5s^1 4d^{10} 5p^1$	6.99	4.78	3.16	4.25
[Kr]$5s^1 4d^{10} 5d^1$	15.48	4.08	8.24	7.40
[Kr]$5s^1 4d^{10} 4f^1$	25.06	3.94	16.24	8.15
[Kr]$4d^{10} 5p^2$	7.30	5.02	3.05	8.77
[Kr]$4d^{10} 5p^1 4f^1$	24.73	4.20	15.86	13.37
[Kr]$4d^{10} 4f^2$	18.51	3.33	9.44	20.69
[Kr]$4d^9 4f^3$	15.42	5.69	6.43	49.82
[Kr]$4d^8 4f^4$	13.78	6.58	4.70	86.74
[Ar]$4s^2 3d^{10} 4p^5 4d^{10} 4f^3$	15.42	5.69	6.42	103.45

In The energies of the low lying configurations of hard-wall confined In are shown in fig. S103 for the neutral atom and in fig. S104 for the cation. The ground state of the unconfined In is [Kr]$5s^2 4d^{10} 5p^1$. At $r_c = 2.2a_0$ the ground state changes to [Kr]$5s^2 4d^{10} 4f^1$. Furthermore, at $r_c = 2.0a_0$ we see a ground state crossing to [Kr]$4d^{10} 4f^3$.

At $r_c = 2.1a_0$ the state [Kr]$5s^1 4d^{10} 4f^2$ flips below the initial ground state. At $r_c = 2.0a_0$ the state [Kr]$4d^{10} 5p^1 4f^2$ flips below the initial ground state. At $r_c = 1.8a_0$ the state [Kr]$4d^9 4f^4$ flips below the initial ground state. At $r_c = 1.7a_0$ the state [Kr]$4d^8 4f^5$ flips below the initial ground state. At $r_c = 1.7a_0$ the state [Ar]$4s^2 3d^{10} 4p^5 4d^{10} 4f^4$ flips below the initial ground state. At $r_c = 1.0a_0$ the state [Kr]$5s^2 4d^{10} 5d^1$ flips below the initial ground state. The ionization energy of the unconfined atom is $\Delta E_0 = 5.65$ eV. The studied configurations, atomic radii and excitation energies in the unconfined calculations are as follows:

configuration	r_ϵ	r_ρ	$r_{\max}$	ΔE
[Kr]$5s^2 4d^{10} 5p^1$	6.31	4.78	2.86	0.00
[Kr]$5s^2 4d^{10} 6s^1$	11.87	4.19	6.97	2.89
[Kr]$5s^2 4d^{10} 5d^1$	14.94	4.26	7.62	3.91
[Kr]$5s^2 4d^{10} 4f^1$	25.04	4.08	16.22	4.74
[Kr]$5s^1 4d^{10} 5p^2$	6.63	5.02	2.78	5.25
[Kr]$5s^1 4d^{10} 4f^2$	18.40	3.78	9.23	19.36
[Kr]$4d^{10} 5p^1 4f^2$	18.25	4.13	8.96	26.00
[Kr]$4d^{10} 4f^3$	15.42	5.69	6.46	37.38
[Kr]$4d^9 4f^4$	13.76	6.58	4.73	76.75
[Kr]$4d^8 4f^5$	12.85	6.58	3.57	122.37
[Ar]$4s^2 3d^{10} 4p^5 4d^{10} 4f^4$	13.78	6.58	4.72	134.24

Sn The energies of the low lying configurations of hard-wall confined Sn are shown in fig. S105 for the neutral atom and in fig. S106 for the cation. The ground state of the unconfined Sn is [Kr]$5s^2 4d^{10} 5p^2$. At $r_c = 2.2a_0$ the ground state changes to [Kr]$5s^2 4d^{10} 4f^2$. Furthermore, at $r_c = 1.9a_0$ we see a ground state crossing to [Kr]$4d^{10} 4f^4$.

At $r_c = 2.2a_0$ the state [Kr]$5s^2 4d^{10} 5p^1 4f^1$ flips below the initial ground state. At $r_c = 2.1a_0$ the state [Kr]$5s^1 4d^{10} 4f^3$ flips below the initial ground state. At $r_c = 1.9a_0$ the state [Kr]$4d^9 4f^5$ flips below the initial ground state. At $r_c = 1.8a_0$ the state [Ar]$4s^2 3d^{10} 4p^5 4d^{10} 4f^5$ flips below the initial ground state. At $r_c = 1.7a_0$ the state [Kr]$4d^8 4f^6$ flips below the initial ground state. The ionization energy of the unconfined atom is $\Delta E_0 = 7.14$ eV. The studied configurations, atomic radii and excitation energies in the unconfined calculations are as follows:

configuration	r_ϵ	r_ρ	$r_{\max}$	ΔE
[Kr]$5s^2 4d^{10} 5p^2$	6.10	4.89	2.57	0.00
[Kr]$5s^2 4d^{10} 5p^1 6s^1$	11.00	4.67	6.32	4.10
[Kr]$5s^2 4d^{10} 5p^1 5d^1$	13.77	4.67	6.20	5.23
[Kr]$5s^1 4d^{10} 5p^3$	6.20	5.02	2.52	6.22
[Kr]$5s^2 4d^{10} 5p^1 4f^1$	24.92	4.28	16.08	6.22
[Kr]$5s^2 4d^{10} 4f^2$	18.35	3.94	9.13	16.28
[Kr]$5s^1 4d^{10} 4f^3$	15.40	5.69	6.21	36.65
[Kr]$4d^{10} 4f^4$	13.75	6.58	4.76	59.23
[Kr]$4d^9 4f^5$	12.84	6.58	3.61	107.80
[Kr]$4d^8 4f^6$	12.33	6.34	2.73	160.99
[Ar]$4s^2 3d^{10} 4p^5 4d^{10} 4f^5$	12.87	6.58	3.58	169.07

Sb The energies of the low lying configurations of hard-wall confined Sb are shown in fig. S107 for the neutral atom and in fig. S108 for the cation. The ground state of the unconfined Sb is [Kr]$5s^2 4d^{10} 5p^3$. At $r_c = 2.1a_0$ the ground state changes to [Kr]$5s^2 4d^{10} 4f^3$. Furthermore, at $r_c = 1.9a_0$ we see a ground state crossing to [Kr]$4d^{10} 4f^5$.

At $r_c = 2.1a_0$ the state [Kr]$5s^1 4d^{10} 4f^4$ flips below the initial ground state. At $r_c = 2.1a_0$ the state [Kr]$5s^2 4d^{10} 5p^1 4f^2$ flips below the initial ground state. At $r_c = 2.1a_0$ the state [Kr]$5s^2 4d^{10} 5p^2 4f^1$ flips below the initial ground state. At $r_c = 1.9a_0$ the state [Kr]$4d^9 4f^6$ flips below the initial ground state. At $r_c = 1.8a_0$ the state [Kr]$4d^8 4f^7$ flips below the initial ground state. At $r_c = 1.8a_0$ the state [Ar]$4s^2 3d^{10} 4p^5 4d^{10} 4f^6$ flips below the initial ground state. The ionization energy of the unconfined atom is $\Delta E_0 = 8.56$ eV. The studied configurations, atomic radii and excitation energies in the unconfined calculations are as follows:

configuration	r_ϵ	r_ρ	$r_{\max}$	ΔE
[Kr]$5s^2 4d^{10} 5p^3$	5.78	4.89	2.35	0.00
[Kr]$5s^2 4d^{10} 5p^2 6s^1$	10.40	4.89	5.88	5.30
[Kr]$5s^2 4d^{10} 5p^2 5d^1$	13.03	4.78	5.20	6.51
[Kr]$5s^1 4d^{10} 5p^4$	5.82	4.89	2.32	7.16
[Kr]$5s^2 4d^{10} 5p^2 4f^1$	24.92	4.35	16.08	7.64
[Kr]$5s^2 4d^{10} 5p^1 4f^2$	18.27	4.15	8.92	19.59
[Kr]$5s^2 4d^{10} 4f^3$	15.44	5.69	6.02	34.19
[Kr]$5s^1 4d^{10} 4f^4$	13.87	6.58	4.44	58.99
[Kr]$4d^{10} 4f^5$	12.82	6.58	3.64	84.89
[Kr]$4d^9 4f^6$	12.32	6.34	2.77	141.41
[Kr]$4d^8 4f^7$	12.02	6.34	2.05	201.31
[Ar]$4s^2 3d^{10} 4p^5 4d^{10} 4f^6$	12.37	6.34	2.73	206.35

Te The energies of the low lying configurations of hard-wall confined Te are shown in fig. S109 for the neutral atom and in fig. S110 for the cation. The ground state of the unconfined Te is [Kr]$5s^2 4d^{10} 5p^4$. At $r_c = 2.1a_0$ the ground state changes to [Kr]$5s^2 4d^{10} 4f^4$. Furthermore, at $r_c = 1.8a_0$ we see a ground state crossing to [Kr]$4d^{10} 4f^6$.

At $r_c = 2.1a_0$ the state [Kr]$5s^2 4d^{10} 5p^1 4f^3$ flips below the initial ground state. At $r_c = 2.1a_0$ the state [Kr]$5s^2 4d^{10} 5p^3 4f^1$ flips below the initial ground state. At $r_c = 2.1a_0$ the state [Kr]$5s^2 4d^{10} 5p^2 4f^2$ flips below the initial ground state. At $r_c = 2.0a_0$ the state [Kr]$5s^1 4d^{10} 4f^5$ flips below the initial ground state. At $r_c = 2.0a_0$ the state [Kr]$4d^{10} 5p^1 4f^5$ flips below the initial ground state. At $r_c = 1.9a_0$ the state [Kr]$4d^9 4f^7$ flips below the initial ground state. At $r_c = 1.8a_0$ the state [Kr]$4d^8 4f^8$ flips below the initial ground state. At $r_c = 1.8a_0$ the state [Ar]$4s^2 3d^{10} 4p^5 4d^{10} 4f^7$ flips below the initial ground state. The ionization energy of the unconfined atom is $\Delta E_0 = 9.94$ eV. The studied configurations, atomic radii and excitation energies in the unconfined calculations are as follows:

configuration	r_ϵ	r_ρ	$r_{\max}$	ΔE
[Kr]$5s^2 4d^{10} 5p^4$	5.48	4.78	2.18	0.00
[Kr]$5s^2 4d^{10} 5p^3 6s^1$	9.95	5.02	5.54	6.51
[Kr]$5s^2 4d^{10} 5p^3 5d^1$	12.51	4.78	4.48	7.79
[Kr]$5s^1 4d^{10} 5p^5$	5.50	4.78	2.16	8.09
[Kr]$5s^2 4d^{10} 5p^3 4f^1$	24.95	4.31	16.12	9.03
[Kr]$5s^2 4d^{10} 5p^2 4f^2$	18.26	4.20	8.81	22.82
[Kr]$5s^2 4d^{10} 5p^1 4f^3$	15.58	5.50	5.64	39.27
[Kr]$5s^2 4d^{10} 4f^4$	14.10	6.34	4.09	56.84
[Kr]$5s^1 4d^{10} 4f^5$	13.10	6.34	3.22	84.55
[Kr]$4d^{10} 5p^1 4f^5$	13.15	6.34	3.06	94.20
[Kr]$4d^{10} 4f^6$	12.31	6.34	2.81	112.72
[Kr]$4d^9 4f^7$	12.03	6.34	2.07	176.22
[Kr]$4d^8 4f^8$	11.86	6.11	1.54	242.26
[Ar]$4s^2 3d^{10} 4p^5 4d^{10} 4f^7$	12.08	6.34	2.00	244.75

I The energies of the low lying configurations of hard-wall confined I are shown in fig. S111 for the neutral atom and in fig. S112 for the cation. The ground state of the unconfined I is [Kr]$5s^2 4d^{10} 5p^5$. At $r_c = 2.1a_0$ the ground state changes to [Kr]$5s^2 4d^{10} 5p^4 4f^1$. Furthermore, at $r_c = 2.0a_0$ we see a ground state crossing to [Kr]$5s^2 4d^{10} 4f^5$. At $r_c = 1.8a_0$ we see a ground state crossing to [Kr]$4d^{10} 4f^7$.

At $r_c = 2.1a_0$ the state [Kr]$5s^2 4d^{10} 5p^3 4f^2$ flips below the initial ground state. At $r_c = 2.0a_0$ the state [Kr]$5s^1 4d^{10} 4f^6$ flips below the initial ground state. At $r_c = 2.0a_0$ the state [Kr]$5s^2 4d^{10} 5p^1 4f^4$ flips below the initial ground state. At $r_c = 2.0a_0$ the state [Kr]$5s^2 4d^{10} 5p^2 4f^3$ flips below the initial ground state. At $r_c = 1.9a_0$ the state [Kr]$4d^{10} 5p^1 4f^6$ flips below the initial ground state. At $r_c = 1.8a_0$ the state [Kr]$4d^9 4f^8$ flips below the initial ground state. At $r_c = 1.8a_0$ the state [Ar]$4s^2 3d^{10} 4p^5 4d^{10} 4f^8$ flips below the initial ground state. At $r_c = 1.7a_0$ the state [Kr]$4d^8 4f^9$ flips below the initial ground state. The ionization energy of the unconfined atom is $\Delta E_0 = 11.33$ eV. The studied configurations, atomic radii and excitation energies in the unconfined calculations are as follows:

configuration	r_ϵ	r_ρ	$r_{\max}$	ΔE
[Kr]$5s^2 4d^{10} 5p^5$	5.21	4.59	2.04	0.00
[Kr]$5s^2 4d^{10} 5p^4 6s^1$	9.58	5.02	5.27	7.75
[Kr]$5s^1 4d^{10} 5p^6$	5.22	4.67	2.03	9.01
[Kr]$5s^2 4d^{10} 5p^4 5d^1$	12.14	4.67	3.95	9.08
[Kr]$5s^2 4d^{10} 5p^4 4f^1$	24.99	4.23	16.17	10.41
[Kr]$5s^2 4d^{10} 5p^3 4f^2$	18.34	4.20	8.75	26.01
[Kr]$5s^2 4d^{10} 5p^2 4f^3$	15.93	5.33	5.18	44.02
[Kr]$5s^2 4d^{10} 5p^1 4f^4$	14.56	5.90	3.16	62.77
[Kr]$5s^2 4d^{10} 4f^5$	13.51	6.11	2.61	81.86
[Kr]$5s^1 4d^{10} 4f^6$	12.72	6.34	2.27	111.46
[Kr]$4d^{10} 5p^1 4f^6$	12.78	6.11	2.17	121.78
[Kr]$4d^{10} 4f^7$	12.04	6.34	2.10	141.30
[Kr]$4d^9 4f^8$	11.88	6.11	1.51	211.13
[Kr]$4d^8 4f^9$	11.78	6.11	1.24	282.98
[Ar]$4s^2 3d^{10} 4p^5 4d^{10} 4f^8$	11.94	6.11	1.45	283.20

Xe The energies of the low lying configurations of hard-wall confined Xe are shown in fig. S113 for the neutral atom and in fig. S114 for the cation. The ground state of the unconfined Xe is [Kr]$5s^2 4d^{10} 5p^6$. At $r_c = 2.0a_0$ the ground state changes to [Kr]$5s^2 4d^{10} 4f^6$. Furthermore, at $r_c = 1.7a_0$ we see a ground state crossing to [Kr]$4d^{10} 4f^8$.

At $r_c = 2.0a_0$ the state [Kr]$5s^1 4d^{10} 4f^7$ flips below the initial ground state. At $r_c = 2.0a_0$ the state [Kr]$5s^2 4d^{10} 5p^1 4f^5$ flips below the initial ground state. At $r_c = 2.0a_0$ the state [Kr]$5s^2 4d^{10} 5p^2 4f^4$ flips below the initial ground state. At $r_c = 2.0a_0$ the state [Kr]$5s^2 4d^{10} 5p^5 4f^1$ flips below the initial ground state. At $r_c = 2.0a_0$ the state [Kr]$5s^2 4d^{10} 5p^4 4f^2$ flips below the initial ground state. At $r_c = 1.9a_0$ the state [Kr]$4d^{10} 5p^1 4f^7$ flips below the initial ground state. At $r_c = 1.8a_0$ the state [Kr]$4d^9 4f^9$ flips below the initial ground state. At $r_c = 1.7a_0$ the state [Ar]$4s^2 3d^{10} 4p^5 4d^{10} 4f^9$ flips below the initial ground state. The ionization energy of the unconfined atom is $\Delta E_0 = 12.72$ eV. The studied configurations, atomic radii and excitation energies in the unconfined calculations are as follows:

configuration	r_ϵ	r_ρ	$r_{\max}$	ΔE
[Kr]$5s^2 4d^{10} 5p^6$	4.96	4.51	1.92	0.00
[Kr]$5s^2 4d^{10} 5p^5 6s^1$	9.28	4.89	5.04	9.00
[Kr]$5s^2 4d^{10} 5p^5 5d^1$	11.87	4.67	3.54	10.38
[Kr]$5s^2 4d^{10} 5p^5 4f^1$	25.03	4.16	16.21	11.80
[Kr]$5s^2 4d^{10} 5p^4 4f^2$	18.62	4.18	8.82	29.07
[Kr]$5s^2 4d^{10} 5p^2 4f^4$	15.29	5.50	1.85	67.62
[Kr]$5s^2 4d^{10} 5p^1 4f^5$	14.20	5.90	1.85	87.36
[Kr]$5s^2 4d^{10} 4f^6$	13.27	6.11	1.66	107.31
[Kr]$5s^1 4d^{10} 4f^7$	12.54	6.11	1.66	138.30
[Kr]$4d^{10} 5p^1 4f^7$	12.61	6.11	1.85	149.19
[Kr]$4d^{10} 4f^8$	11.89	6.11	1.49	169.47
[Kr]$4d^9 4f^9$	11.82	6.11	1.19	245.25
[Ar]$4s^2 3d^{10} 4p^5 4d^{10} 4f^9$	11.87	6.11	1.16	320.84

3.2 PBE

H The energies of the low lying configurations of hard-wall confined H are shown in fig. S8 for the neutral atom. The ground state of the unconfined H is $1s^1$. We do not observe any ground state crossing for H in the considered confinement radii.

The ionization energy of the unconfined atom is $\Delta E_0 = 12.49$ eV. The studied configurations, atomic radii and excitation energies in the unconfined calculations are as follows:

configuration	r_ϵ	r_ρ	$r_{\max}$	ΔE
$1s^1$	3.22	3.06	1.03	0.00
$2p^1$	8.95	3.89	4.24	9.24
$3d^1$	16.31	undefined	9.57	10.96

He The energies of the low lying configurations of hard-wall confined He are shown in fig. S9 for the neutral atom and in fig. S10 for the cation. The ground state of the unconfined He is $1s^2$. We do not observe any ground state crossing for He in the considered confinement radii.

The ionization energy of the unconfined atom is $\Delta E_0 = 26.96$ eV. The studied configurations, atomic radii and excitation energies in the unconfined calculations are as follows:

configuration	r_ϵ	r_ρ	$r_{\max}$	ΔE
$1s^2$	2.26	2.68	0.56	0.00
$1s^1 2p^1$	8.55	3.94	3.88	23.44
$1s^1 3d^1$	16.26	2.10	9.52	25.43
$2p^2$	6.49	4.59	2.35	59.60

Li The energies of the low lying configurations of hard-wall confined Li are shown in fig. S11 for the neutral atom and in fig. S12 for the cation. The ground state of the unconfined Li is [He]$2s^1$. At $r_c = 3.0a_0$ the ground state changes to [He]$2p^1$.

The ionization energy of the unconfined atom is $\Delta E_0 = 5.30$ eV. The studied configurations, atomic radii and excitation energies in the unconfined calculations are as follows:

configuration	r_ϵ	r_ρ	$r_{\max}$	ΔE
[He]$2s^1$	6.58	4.17	3.13	0.00
[He]$2p^1$	8.54	3.94	3.88	1.79
[He]$3d^1$	16.28	1.91	9.54	3.77

Be The energies of the low lying configurations of hard-wall confined Be are shown in fig. S13 for the neutral atom and in fig. S14 for the cation. The ground state of the unconfined Be is [He]$2s^2$. At $r_c = 2.2a_0$ the ground state changes to [He]$2p^2$.

At $r_c = 2.2a_0$ the state [He]$2s^1 2p^1$ flips below the initial ground state. At $r_c = 1.2a_0$ the state [He]$2p^1 3d^1$ flips below the initial ground state. The ionization energy of the unconfined atom is $\Delta E_0 = 9.55$ eV. The studied configurations, atomic radii and excitation energies in the unconfined calculations are as follows:

configuration	r_ϵ	r_ρ	$r_{\max}$	ΔE
[He]$2s^2$	5.06	4.15	2.04	0.00
[He]$2s^12p^1$	5.91	4.28	2.24	3.61
[He]$2p^2$	6.32	4.45	2.21	7.25
[He]$2p^13d^1$	15.31	3.78	8.52	11.80

B The energies of the low lying configurations of hard-wall confined B are shown in fig. S15 for the neutral atom and in fig. S16 for the cation. The ground state of the unconfined B is [He]$2s^22p^1$. At $r_c = 1.8a_0$ the ground state changes to [He]$2p^3$.

At $r_c = 1.8a_0$ the state [He]$2s^12p^2$ flips below the initial ground state. The ionization energy of the unconfined atom is $\Delta E_0 = 8.22$ eV. The studied configurations, atomic radii and excitation energies in the unconfined calculations are as follows:

configuration	r_ϵ	r_ρ	$r_{\max}$	ΔE
[He]$2s^22p^1$	4.67	3.94	1.58	0.00
[He]$2s^23s^1$	10.08	3.71	5.72	4.95
[He]$2s^12p^2$	4.97	4.05	1.56	5.84
[He]$2p^3$	5.16	4.15	1.55	11.71

C The energies of the low lying configurations of hard-wall confined C are shown in fig. S17 for the neutral atom and in fig. S18 for the cation. The ground state of the unconfined C is [He]$2s^22p^2$. At $r_c = 1.5a_0$ the ground state changes to [He]$2p^4$.

At $r_c = 1.5a_0$ the state [He]$2s^12p^3$ flips below the initial ground state. The ionization energy of the unconfined atom is $\Delta E_0 = 10.87$ eV. The studied configurations, atomic radii and excitation energies in the unconfined calculations are as follows:

configuration	r_ϵ	r_ρ	$r_{\max}$	ΔE
[He]$2s^22p^2$	4.14	3.78	1.21	0.00
[He]$2s^22p^13s^1$	9.11	4.08	4.98	7.18
[He]$2s^12p^3$	4.30	3.83	1.21	8.46
[He]$2p^4$	4.41	3.89	1.19	16.94

N The energies of the low lying configurations of hard-wall confined N are shown in fig. S19 for the neutral atom and in fig. S20 for the cation. The ground state of the unconfined N is [He]$2s^22p^3$. At $r_c = 1.2a_0$ the ground state changes to [He]$2p^5$.

At $r_c = 1.2a_0$ the state [He]$2s^12p^4$ flips below the initial ground state. The ionization energy of the unconfined atom is $\Delta E_0 = 13.60$ eV. The studied configurations, atomic radii and excitation energies in the unconfined calculations are as follows:

configuration	r_ϵ	r_ρ	$r_{\max}$	ΔE
[He]$2s^22p^3$	3.71	3.57	1.01	0.00
[He]$2s^22p^23s^1$	8.43	4.31	4.46	9.56
[He]$2s^12p^4$	3.81	3.64	1.01	11.48
[He]$2p^5$	3.88	3.64	0.97	22.98

O The energies of the low lying configurations of hard-wall confined O are shown in fig. S21 for the neutral atom and in fig. S22 for the cation. The ground state of the unconfined O is [He]$2s^22p^4$. At $r_c = 1.1a_0$ the ground state changes to [He]$2p^6$.

At $r_c = 1.1a_0$ the state [He]$2s^12p^5$ flips below the initial ground state. The ionization energy of the unconfined atom is $\Delta E_0 = 16.44$ eV. The studied configurations, atomic radii and excitation energies in the unconfined calculations are as follows:

configuration	r_ϵ	r_ρ	$r_{\max}$	ΔE
[He]$2s^22p^4$	3.36	3.41	0.87	0.00
[He]$2s^22p^33s^1$	7.92	4.35	4.08	12.09
[He]$2s^12p^5$	3.43	3.41	0.87	14.90
[He]$2p^6$	3.49	3.49	0.82	29.83

F The energies of the low lying configurations of hard-wall confined F are shown in fig. S23 for the neutral atom and in fig. S24 for the cation. The ground state of the unconfined F is [He]$2s^22p^5$. At $r_c = 1.0a_0$ the ground state changes to [He]$2s^12p^6$.

The ionization energy of the unconfined atom is $\Delta E_0 = 19.40$ eV. The studied configurations, atomic radii and excitation energies in the unconfined calculations are as follows:

configuration	r_ϵ	r_ρ	$r_{\max}$	ΔE
[He]$2s^22p^5$	3.08	3.24	0.77	0.00
[He]$2s^22p^43s^1$	7.50	4.35	3.77	14.77
[He]$2s^22p^43d^1$	16.02	2.87	9.28	17.84
[He]$2s^12p^6$	3.13	3.24	0.77	18.72
[He]$2p^63d^1$	15.98	2.96	9.24	55.89

Ne The energies of the low lying configurations of hard-wall confined Ne are shown in

fig. S25 for the neutral atom and in fig. S26 for the cation. The ground state of the unconfined Ne is [He]$2s^2 2p^6$. We do not observe any ground state crossing for Ne in the considered confinement radii.

The ionization energy of the unconfined atom is $\Delta E_0 = 22.49$ eV. The studied configurations, atomic radii and excitation energies in the unconfined calculations are as follows:

configuration	r_ϵ	r_ρ	$r_{\max}$	ΔE
[He]$2s^2 2p^6$	2.84	3.06	0.68	0.00
[He]$2s^2 2p^5 3s^1$	7.17	4.28	3.53	17.59
[He]$2s^2 2p^5 3d^1$	16.07	2.78	9.34	20.94
[He]$2s^1 2p^6 3d^1$	16.06	2.78	9.32	44.19

Na The energies of the low lying configurations of hard-wall confined Na are shown in fig. S27 for the neutral atom and in fig. S28 for the cation. The ground state of the unconfined Na is [Ne]$3s^1$. At $r_c = 2.0a_0$ the ground state changes to [Ne]$3d^1$.

At $r_c = 1.6a_0$ the state [Ne]$3p^1$ flips below the initial ground state. At $r_c = 1.1a_0$ the state [Ne]$4f^1$ flips below the initial ground state. The ionization energy of the unconfined atom is $\Delta E_0 = 5.14$ eV. The studied configurations, atomic radii and excitation energies in the unconfined calculations are as follows:

configuration	r_ϵ	r_ρ	$r_{\max}$	ΔE
[Ne]$3s^1$	6.88	4.23	3.33	0.00
[Ne]$3p^1$	9.69	3.41	4.88	2.07
[Ne]$3d^1$	16.12	2.68	9.38	3.59
[Ne]$4f^1$	24.87	2.59	16.83	4.23

Mg The energies of the low lying configurations of hard-wall confined Mg are shown in fig. S29 for the neutral atom and in fig. S30 for the cation. The ground state of the unconfined Mg is [Ne]$3s^2$. At $r_c = 1.8a_0$ the ground state changes to [Ne]$3d^2$.

At $r_c = 1.8a_0$ the state [Ne]$3s^1 3d^1$ flips below the initial ground state. At $r_c = 1.7a_0$ the state [Ne]$3p^1 3d^1$ flips below the initial ground state. At $r_c = 1.4a_0$ the state [Ne]$3s^1 3p^1$ flips below the initial ground state. At $r_c = 1.4a_0$ the state [Ne]$3p^2$ flips below the initial ground state. At $r_c = 1.3a_0$ the state [Ne]$3d^1 4f^1$ flips below the initial ground state. The ionization energy of the unconfined atom is $\Delta E_0 = 7.95$ eV. The studied configurations, atomic radii and excitation energies in the unconfined calculations are as follows:

configuration	r_ϵ	r_ρ	$r_{\max}$	ΔE
[Ne]$3s^2$	5.89	4.59	2.56	0.00
[Ne]$3s^1 3p^1$	7.35	4.89	3.34	3.48
[Ne]$3s^1 3d^1$	14.90	4.08	8.05	6.23
[Ne]$3p^2$	7.63	5.17	3.25	7.15
[Ne]$3p^1 3d^1$	13.21	4.78	6.01	10.35
[Ne]$3d^2$	11.80	5.33	5.24	14.10
[Ne]$3d^1 4f^1$	22.38	3.98	13.97	15.78

Al The energies of the low lying configurations of hard-wall confined Al are shown in fig. S31 for the neutral atom and in fig. S32 for the cation. The ground state of the unconfined Al is [Ne]$3s^2 3p^1$. At $r_c = 1.9a_0$ the ground state changes to [Ne]$3s^2 3d^1$. Furthermore, at $r_c = 1.6a_0$ we see a ground state crossing to [Ne]$3d^3$.

At $r_c = 1.7a_0$ the state [Ne]$3s^1 3d^2$ flips below the initial ground state. At $r_c = 1.6a_0$ the state [Ne]$3p^1 3d^2$ flips below the initial ground state. At $r_c = 1.3a_0$ the state [Ne]$3s^1 3p^2$ flips below the initial ground state. The ionization energy of the unconfined atom is $\Delta E_0 = 5.77$ eV. The studied configurations, atomic radii and excitation energies in the unconfined calculations are as follows:

configuration	r_ϵ	r_ρ	$r_{\max}$	ΔE
[Ne]$3s^2 3p^1$	6.12	4.67	2.64	0.00
[Ne]$3s^2 4s^1$	11.50	4.08	6.84	2.94
[Ne]$3s^2 3d^1$	14.60	4.16	7.66	3.98
[Ne]$3s^1 3p^2$	6.40	4.78	2.57	5.11
[Ne]$3s^1 3d^2$	11.16	5.33	4.32	15.17
[Ne]$3p^1 3d^2$	10.63	5.50	3.94	21.12
[Ne]$3d^3$	9.76	5.90	3.61	27.43

Si The energies of the low lying configurations of hard-wall confined Si are shown in fig. S33 for the neutral atom and in fig. S34 for the cation. The ground state of the unconfined Si is [Ne]$3s^2 3p^2$. At $r_c = 1.7a_0$ the ground state changes to [Ne]$3s^2 3d^2$. Furthermore, at $r_c = 1.5a_0$ we see a ground state crossing to [Ne]$3d^4$.

At $r_c = 1.7a_0$ the state [Ne]$3s^2 3p^1 3d^1$ flips below the initial ground state. At $r_c = 1.6a_0$ the state [Ne]$3s^1 3d^3$ flips below the initial ground

state. At $r_c = 1.5a_0$ the state [Ne]$3p^13d^3$ flips below the initial ground state. At $r_c = 1.2a_0$ the state [Ne]$3s^13p^3$ flips below the initial ground state. The ionization energy of the unconfined atom is $\Delta E_0 = 7.75$ eV. The studied configurations, atomic radii and excitation energies in the unconfined calculations are as follows:

configuration	r_ϵ	r_ρ	$r_{\max}$	ΔE
[Ne]$3s^23p^2$	5.56	4.51	2.16	0.00
[Ne]$3s^23p^14s^1$	10.44	4.39	5.99	4.57
[Ne]$3s^23p^13d^1$	14.02	4.26	6.67	5.84
[Ne]$3s^13p^3$	5.65	4.59	2.12	6.76
[Ne]$3s^23d^2$	10.92	5.17	3.66	13.32
[Ne]$3s^13d^3$	9.36	5.50	3.01	29.23
[Ne]$3p^13d^3$	9.09	5.50	2.90	36.97
[Ne]$3d^4$	8.51	5.69	2.74	45.96

P The energies of the low lying configurations of hard-wall confined P are shown in fig. S35 for the neutral atom and in fig. S36 for the cation. The ground state of the unconfined P is [Ne]$3s^23p^3$. At $r_c = 1.5a_0$ the ground state changes to [Ne]$3s^23d^3$. Furthermore, at $r_c = 1.4a_0$ we see a ground state crossing to [Ne]$3d^5$.

At $r_c = 1.5a_0$ the state [Ne]$3s^13d^4$ flips below the initial ground state. At $r_c = 1.5a_0$ the state [Ne]$3s^23p^23d^1$ flips below the initial ground state. At $r_c = 1.5a_0$ the state [Ne]$3s^23p^13d^2$ flips below the initial ground state. At $r_c = 1.4a_0$ the state [Ne]$3p^13d^4$ flips below the initial ground state. At $r_c = 1.1a_0$ the state [Ne]$3s^13p^4$ flips below the initial ground state. The ionization energy of the unconfined atom is $\Delta E_0 = 9.74$ eV. The studied configurations, atomic radii and excitation energies in the unconfined calculations are as follows:

configuration	r_ϵ	r_ρ	$r_{\max}$	ΔE
[Ne]$3s^23p^3$	5.04	4.28	1.84	0.00
[Ne]$3s^23p^24s^1$	9.72	4.51	5.42	6.28
[Ne]$3s^23p^23d^1$	13.93	4.17	6.33	7.78
[Ne]$3s^13p^4$	5.08	4.35	1.81	8.45
[Ne]$3s^23p^13d^2$	10.55	5.02	2.94	17.59
[Ne]$3s^23d^3$	9.16	5.33	2.57	28.23
[Ne]$3s^13d^4$	8.23	5.50	2.34	48.90
[Ne]$3p^13d^4$	8.06	5.33	2.29	58.42
[Ne]$3d^5$	7.64	5.33	2.20	70.20

S The energies of the low lying configurations of hard-wall confined S are shown in fig. S37 for the neutral atom and in fig. S38 for the cation. The ground state of the unconfined S is [Ne]$3s^23p^4$. At $r_c = 1.4a_0$ the ground state changes to [Ne]$3s^23d^4$. Furthermore, at $r_c = 1.3a_0$ we see a ground state crossing to [Ne]$3d^6$.

At $r_c = 1.4a_0$ the state [Ne]$3p^13d^5$ flips below the initial ground state. At $r_c = 1.4a_0$ the state [Ne]$3s^13d^5$ flips below the initial ground state. At $r_c = 1.4a_0$ the state [Ne]$3s^23p^13d^3$ flips below the initial ground state. At $r_c = 1.4a_0$ the state [Ne]$3s^23p^23d^2$ flips below the initial ground state. At $r_c = 1.4a_0$ the state [Ne]$3s^23p^33d^1$ flips below the initial ground state. At $r_c = 1.1a_0$ the state [Ne]$3s^13p^5$ flips below the initial ground state. The ionization energy of the unconfined atom is $\Delta E_0 = 11.78$ eV. The studied configurations, atomic radii and excitation energies in the unconfined calculations are as follows:

configuration	r_ϵ	r_ρ	$r_{\max}$	ΔE
[Ne]$3s^23p^4$	4.61	4.11	1.61	0.00
[Ne]$3s^23p^34s^1$	9.19	4.51	5.00	8.08
[Ne]$3s^23p^33d^1$	14.05	4.05	6.59	9.81
[Ne]$3s^13p^5$	4.63	4.15	1.59	10.18
[Ne]$3s^23p^23d^2$	10.43	4.78	2.47	22.11
[Ne]$3s^23p^13d^3$	8.90	5.17	2.18	35.31
[Ne]$3s^23d^4$	8.07	5.33	2.03	49.10
[Ne]$3s^13d^5$	7.43	5.17	1.92	74.60
[Ne]$3p^13d^5$	7.31	5.17	1.90	85.92
[Ne]$3d^6$	6.99	5.17	1.84	100.63

Cl The energies of the low lying configurations of hard-wall confined Cl are shown in fig. S39 for the neutral atom and in fig. S40 for the cation. The ground state of the unconfined Cl is [Ne]$3s^23p^5$. At $r_c = 1.3a_0$ the ground state changes to [Ne]$3s^13d^6$. Furthermore, at $r_c = 1.2a_0$ we see a ground state crossing to [Ne]$3d^7$.

At $r_c = 1.3a_0$ the state [Ne]$3p^13d^6$ flips below the initial ground state. At $r_c = 1.3a_0$ the state [Ne]$3s^23p^43d^1$ flips below the initial ground state. At $r_c = 1.3a_0$ the state [Ne]$3s^23p^33d^2$ flips below the initial ground state. At $r_c = 1.0a_0$ the state [Ne]$3s^13p^6$ flips below the ini-

tial ground state. The ionization energy of the unconfined atom is $\Delta E_0 = 13.89$ eV. The studied configurations, atomic radii and excitation energies in the unconfined calculations are as follows:

configuration	r_ϵ	r_ρ	$r_{\max}$	ΔE
[Ne]$3s^2 3p^5$	4.25	3.94	1.43	0.00
[Ne]$3s^2 3p^4 4s^1$	8.76	4.51	4.68	9.97
[Ne]$3s^2 3p^4 3d^1$	14.24	3.89	6.97	11.94
[Ne]$3s^1 3p^6$	4.26	3.98	1.42	11.97
[Ne]$3s^2 3p^3 3d^2$	10.45	4.67	2.12	26.88
[Ne]$3s^1 3d^6$	6.83	5.02	1.63	106.81
[Ne]$3p^1 3d^6$	6.74	5.02	1.62	119.96
[Ne]$3d^7$	6.48	4.89	1.58	137.72

Ar The energies of the low lying configurations of hard-wall confined Ar are shown in fig. S41 for the neutral atom and in fig. S42 for the cation. The ground state of the unconfined Ar is [Ne]$3s^2 3p^6$. At $r_c = 1.2a_0$ the ground state changes to [Ne]$3s^2 3d^6$. Furthermore, at $r_c = 1.1a_0$ we see a ground state crossing to [Ne]$3d^8$.

At $r_c = 1.2a_0$ the state [Ne]$3p^1 3d^7$ flips below the initial ground state. At $r_c = 1.2a_0$ the state [Ne]$3s^1 3d^7$ flips below the initial ground state. At $r_c = 1.2a_0$ the state [Ne]$3s^2 3p^1 3d^5$ flips below the initial ground state. At $r_c = 1.2a_0$ the state [Ne]$3s^2 3p^5 3d^1$ flips below the initial ground state. At $r_c = 1.2a_0$ the state [Ne]$3s^2 3p^4 3d^2$ flips below the initial ground state. At $r_c = 1.1a_0$ the state [Ne]$3s^1 3p^6 3d^1$ flips below the initial ground state. The ionization energy of the unconfined atom is $\Delta E_0 = 16.07$ eV. The studied configurations, atomic radii and excitation energies in the unconfined calculations are as follows:

configuration	r_ϵ	r_ρ	$r_{\max}$	ΔE
[Ne]$3s^2 3p^6$	3.95	3.83	1.29	0.00
[Ne]$3s^2 3p^5 4s^1$	8.41	4.45	4.41	11.96
[Ne]$3s^2 3p^5 3d^1$	14.45	3.78	7.34	14.16
[Ne]$3s^1 3p^6 3d^1$	14.31	3.78	7.06	28.46
[Ne]$3s^2 3p^4 3d^2$	10.57	4.51	1.84	31.90
[Ne]$3s^2 3p^1 3d^5$	7.15	4.89	1.50	90.08
[Ne]$3s^2 3d^6$	6.72	4.89	1.46	110.37
[Ne]$3s^1 3d^7$	6.35	4.78	1.42	145.98
[Ne]$3p^1 3d^7$	6.28	4.78	1.41	161.01
[Ne]$3d^8$	6.06	4.78	1.39	181.97

K The energies of the low lying configurations of hard-wall confined K are shown in fig. S43 for the neutral atom and in fig. S44 for the cation. The ground state of the unconfined K is [Ar]$4s^1$. At $r_c = 4.6a_0$ the ground state changes to [Ar]$3d^1$. Furthermore, at $r_c = 1.2a_0$ we see a ground state crossing to [Ne]$3s^2 3p^5 3d^2$. At $r_c = 1.1a_0$ we see a ground state crossing to [Ne]$3s^1 3d^8$. Moreover, at $r_c = 1.0a_0$ we see a ground state crossing to [Ne]$3d^9$.

At $r_c = 2.5a_0$ the state [Ne]$3s^1 3p^6 3d^2$ flips below the initial ground state. At $r_c = 2.3a_0$ the state [Ne]$3s^2 3p^4 3d^3$ flips below the initial ground state. At $r_c = 1.5a_0$ the state [Ne]$3p^1 3d^8$ flips below the initial ground state. At $r_c = 1.4a_0$ the state [Ar]$4p^1$ flips below the initial ground state. The ionization energy of the unconfined atom is $\Delta E_0 = 4.30$ eV. The studied configurations, atomic radii and excitation energies in the unconfined calculations are as follows:

configuration	r_ϵ	r_ρ	$r_{\max}$	ΔE
[Ar]$4s^1$	8.11	4.45	4.19	0.00
[Ar]$4p^1$	10.81	3.71	5.82	1.54
[Ar]$3d^1$	14.65	3.64	7.65	2.43
[Ne]$3s^2 3p^5 3d^2$	10.73	4.39	1.62	23.12
[Ne]$3s^1 3p^6 3d^2$	10.63	4.35	1.57	39.56
[Ne]$3s^2 3p^4 3d^3$	8.84	4.78	1.47	44.97
[Ne]$3s^1 3d^8$	5.95	4.67	1.26	178.57
[Ne]$3p^1 3d^8$	5.90	4.59	1.25	195.52
[Ne]$3d^9$	5.71	4.59	1.24	219.83

Ca The energies of the low lying configurations of hard-wall confined Ca are shown in fig. S45 for the neutral atom and in fig. S46 for the cation. The ground state of the unconfined Ca is [Ar]$4s^2$. At $r_c = 4.6a_0$ the ground state changes to [Ar]$4s^1 3d^1$. Furthermore, at $r_c = 4.4a_0$ we see a ground state crossing to [Ar]$3d^2$. At $r_c = 1.1a_0$ we see a ground state crossing to [Ne]$3s^2 3p^4 3d^4$. Moreover, at $r_c = 1.0a_0$ we see a ground state crossing to [Ne]$3d^{10}$.

At $r_c = 3.4a_0$ the state [Ar]$3d^1 4p^1$ flips below the initial ground state. At $r_c = 3.1a_0$ the state [Ne]$3s^2 3p^5 3d^3$ flips below the initial ground state. At $r_c = 2.8a_0$ the state [Ne]$3s^1 3p^6 3d^3$ flips below the initial ground state. At $r_c =$

$1.8a_0$ the state [Ne]$3s^1 3d^9$ flips below the initial ground state. At $r_c = 1.7a_0$ the state [Ne]$3p^1 3d^9$ flips below the initial ground state. At $r_c = 1.4a_0$ the state [Ar]$4s^1 4p^1$ flips below the initial ground state. The ionization energy of the unconfined atom is $\Delta E_0 = 6.29$ eV. The studied configurations, atomic radii and excitation energies in the unconfined calculations are as follows:

configuration	r_ϵ	r_ρ	$r_{\max}$	ΔE
[Ar]$4s^2$	7.20	5.17	3.41	0.00
[Ar]$4s^1 3d^1$	8.20	4.89	3.57	2.19
[Ar]$4s^1 4p^1$	8.53	5.33	4.30	2.39
[Ar]$3d^1 4p^1$	9.56	4.89	4.82	4.26
[Ar]$3d^2$	10.92	4.28	1.43	4.75
[Ne]$3s^2 3p^5 3d^3$	8.97	4.67	1.31	29.81
[Ne]$3s^1 3p^6 3d^3$	8.88	4.59	1.30	48.36
[Ne]$3s^2 3p^4 3d^4$	7.78	4.67	1.25	55.56
[Ne]$3s^1 3d^9$	5.63	4.45	1.13	209.26
[Ne]$3p^1 3d^9$	5.58	4.45	1.13	228.19
[Ne]$3d^{10}$	5.42	4.39	1.11	256.02

Sc The energies of the low lying configurations of hard-wall confined Sc are shown in fig. S47 for the neutral atom and in fig. S48 for the cation. The ground state of the unconfined Sc is [Ar]$4s^2 3d^1$. At $r_c = 4.7a_0$ the ground state changes to [Ar]$4s^1 3d^2$. Furthermore, at $r_c = 4.3a_0$ we see a ground state crossing to [Ar]$3d^3$. At $r_c = 1.0a_0$ we see a ground state crossing to [Ne]$3s^2 3d^9$.

At $r_c = 3.3a_0$ the state [Ar]$3d^2 4p^1$ flips below the initial ground state. At $r_c = 3.0a_0$ the state [Ne]$3s^2 3p^5 3d^4$ flips below the initial ground state. At $r_c = 2.6a_0$ the state [Ne]$3s^1 3p^6 3d^4$ flips below the initial ground state. At $r_c = 2.5a_0$ the state [Ne]$3s^2 3p^4 3d^5$ flips below the initial ground state. At $r_c = 1.9a_0$ the state [Ne]$3s^2 3p^1 3d^8$ flips below the initial ground state. At $r_c = 1.7a_0$ the state [Ne]$3s^1 3d^{10}$ flips below the initial ground state. At $r_c = 1.3a_0$ the state [Ar]$4s^1 3d^1 4p^1$ flips below the initial ground state. The ionization energy of the unconfined atom is $\Delta E_0 = 6.85$ eV. The studied configurations, atomic radii and excitation energies in the unconfined calculations are as follows:

configuration	r_ϵ	r_ρ	$r_{\max}$	ΔE
[Ar]$4s^2 3d^1$	6.76	5.17	3.14	0.00
[Ar]$4s^1 3d^2$	7.23	4.89	3.29	1.54
[Ar]$4s^1 3d^1 4p^1$	8.08	5.33	3.96	2.73
[Ar]$3d^3$	9.14	4.59	1.18	3.90
[Ar]$3d^2 4p^1$	8.98	4.89	4.42	3.91
[Ne]$3s^2 3p^5 3d^4$	7.87	4.59	1.14	33.05
[Ne]$3s^1 3p^6 3d^4$	7.80	4.59	1.13	53.74
[Ne]$3s^2 3p^4 3d^5$	7.05	4.59	1.11	62.73
[Ne]$3s^2 3p^1 3d^8$	5.80	4.45	1.05	154.02
[Ne]$3s^2 3d^9$	5.57	4.39	1.04	185.03
[Ne]$3s^1 3d^{10}$	5.35	4.31	1.03	237.24

Ti The energies of the low lying configurations of hard-wall confined Ti are shown in fig. S49 for the neutral atom and in fig. S50 for the cation. The ground state of the unconfined Ti is [Ar]$4s^2 3d^2$. At $r_c = 4.9a_0$ the ground state changes to [Ar]$4s^1 3d^3$. Furthermore, at $r_c = 4.3a_0$ we see a ground state crossing to [Ar]$3d^4$. At $r_c = 1.0a_0$ we see a ground state crossing to [Ne]$3s^2 3p^4 3d^6$.

At $r_c = 3.2a_0$ the state [Ar]$3d^3 4p^1$ flips below the initial ground state. At $r_c = 2.8a_0$ the state [Ne]$3s^2 3p^5 3d^5$ flips below the initial ground state. At $r_c = 2.5a_0$ the state [Ne]$3s^1 3p^6 3d^5$ flips below the initial ground state. At $r_c = 2.1a_0$ the state [Ne]$3s^2 3p^3 3d^7$ flips below the initial ground state. At $r_c = 1.3a_0$ the state [Ar]$4s^1 3d^2 4p^1$ flips below the initial ground state. The ionization energy of the unconfined atom is $\Delta E_0 = 7.15$ eV. The studied configurations, atomic radii and excitation energies in the unconfined calculations are as follows:

configuration	r_ϵ	r_ρ	$r_{\max}$	ΔE
[Ar]$4s^2 3d^2$	6.44	5.02	2.94	0.00
[Ar]$4s^1 3d^3$	6.70	4.78	3.09	0.95
[Ar]$4s^1 3d^2 4p^1$	7.79	5.17	3.74	3.01
[Ar]$3d^4$	8.01	4.51	1.04	3.05
[Ar]$3d^3 4p^1$	8.67	4.89	4.17	3.56
[Ne]$3s^2 3p^5 3d^5$	7.12	4.51	1.01	36.31
[Ne]$3s^1 3p^6 3d^5$	7.05	4.45	1.01	59.17
[Ne]$3s^2 3p^4 3d^6$	6.50	4.45	0.99	69.99
[Ne]$3s^2 3p^3 3d^7$	6.07	4.39	0.98	104.04

V The energies of the low lying configurations of hard-wall confined V are shown in fig. S51 for the neutral atom and in fig. S52

for the cation. The ground state of the unconfined V is [Ar]$4s^2 3d^3$. At $r_c = 5.3a_0$ the ground state changes to [Ar]$4s^1 3d^4$. Furthermore, at $r_c = 4.3a_0$ we see a ground state crossing to [Ar]$3d^5$.

At $r_c = 3.1a_0$ the state [Ar]$3d^4 4p^1$ flips below the initial ground state. At $r_c = 2.7a_0$ the state [Ne]$3s^2 3p^5 3d^6$ flips below the initial ground state. At $r_c = 2.4a_0$ the state [Ne]$3s^1 3p^6 3d^6$ flips below the initial ground state. At $r_c = 2.3a_0$ the state [Ne]$3s^2 3p^4 3d^7$ flips below the initial ground state. The ionization energy of the unconfined atom is $\Delta E_0 = 6.90$ eV. The studied configurations, atomic radii and excitation energies in the unconfined calculations are as follows:

configuration	r_ϵ	r_ρ	$r_{\max}$	ΔE
[Ar]$4s^2 3d^3$	6.18	4.89	2.79	0.00
[Ar]$4s^1 3d^4$	6.33	4.67	2.92	0.40
[Ar]$3d^5$	7.22	4.45	0.93	2.24
[Ar]$3d^4 4p^1$	8.47	4.78	3.98	3.22
[Ne]$3s^2 3p^5 3d^6$	6.55	4.39	0.92	39.68
[Ne]$3s^1 3p^6 3d^6$	6.50	4.35	0.91	64.77
[Ne]$3s^2 3p^4 3d^7$	6.07	4.31	0.91	77.49

Cr The energies of the low lying configurations of hard-wall confined Cr are shown in fig. S53 for the neutral atom and in fig. S54 for the cation. The ground state of the unconfined Cr is [Ar]$4s^1 3d^5$. At $r_c = 4.3a_0$ the ground state changes to [Ar]$3d^6$.

At $r_c = 2.1a_0$ the state [Ne]$3s^2 3p^5 3d^7$ flips below the initial ground state. At $r_c = 1.9a_0$ the state [Ne]$3s^1 3p^6 3d^7$ flips below the initial ground state. At $r_c = 1.7a_0$ the state [Ne]$3s^2 3p^4 3d^8$ flips below the initial ground state. At $r_c = 1.2a_0$ the state [Ar]$3d^5 4p^1$ flips below the initial ground state. The ionization energy of the unconfined atom is $\Delta E_0 = 6.78$ eV. The studied configurations, atomic radii and excitation energies in the unconfined calculations are as follows:

configuration	r_ϵ	r_ρ	$r_{\max}$	ΔE
[Ar]$4s^1 3d^5$	6.06	4.59	2.79	0.00
[Ar]$4s^2 3d^4$	5.97	4.78	2.65	0.12
[Ar]$3d^6$	6.64	4.31	0.85	1.59
[Ar]$3d^5 4p^1$	8.32	4.67	3.83	3.03
[Ne]$3s^2 3p^5 3d^7$	6.11	4.26	0.84	43.33
[Ne]$3s^1 3p^6 3d^7$	6.07	4.23	0.84	70.70
[Ne]$3s^2 3p^4 3d^8$	5.72	4.20	0.83	85.41

Mn The energies of the low lying configurations of hard-wall confined Mn are shown in fig. S55 for the neutral atom and in fig. S56 for the cation. The ground state of the unconfined Mn is [Ar]$4s^1 3d^6$. At $r_c = 4.3a_0$ the ground state changes to [Ar]$3d^7$.

At $r_c = 2.0a_0$ the state [Ne]$3s^2 3p^5 3d^8$ flips below the initial ground state. At $r_c = 1.8a_0$ the state [Ne]$3s^1 3p^6 3d^8$ flips below the initial ground state. At $r_c = 1.1a_0$ the state [Ar]$3d^6 4p^1$ flips below the initial ground state. The ionization energy of the unconfined atom is $\Delta E_0 = 7.03$ eV. The studied configurations, atomic radii and excitation energies in the unconfined calculations are as follows:

configuration	r_ϵ	r_ρ	$r_{\max}$	ΔE
[Ar]$4s^1 3d^6$	5.83	4.45	2.67	0.00
[Ar]$4s^2 3d^5$	5.78	4.67	2.54	0.62
[Ar]$3d^7$	6.18	4.21	0.78	1.34
[Ar]$3d^6 4p^1$	8.21	4.59	3.71	3.22
[Ne]$3s^2 3p^5 3d^8$	5.76	4.16	0.78	47.52
[Ne]$3s^1 3p^6 3d^8$	5.72	4.15	0.78	77.24

Fe The energies of the low lying configurations of hard-wall confined Fe are shown in fig. S57 for the neutral atom and in fig. S58 for the cation. The ground state of the unconfined Fe is [Ar]$4s^1 3d^7$. At $r_c = 4.4a_0$ the ground state changes to [Ar]$3d^8$.

At $r_c = 2.0a_0$ the state [Ne]$3s^2 3p^5 3d^9$ flips below the initial ground state. At $r_c = 1.7a_0$ the state [Ne]$3s^1 3p^6 3d^9$ flips below the initial ground state. At $r_c = 1.1a_0$ the state [Ar]$3d^7 4p^1$ flips below the initial ground state. The ionization energy of the unconfined atom is $\Delta E_0 = 7.26$ eV. The studied configurations, atomic radii and excitation energies in the unconfined calculations are as follows:

configuration	r_ϵ	r_ρ	$r_{\max}$	ΔE
[Ar]$4s^1 3d^7$	5.64	4.35	2.57	0.00
[Ar]$4s^2 3d^6$	5.62	4.59	2.44	1.09
[Ar]$3d^8$	5.81	4.15	0.73	1.10
[Ar]$3d^7 4p^1$	8.12	4.51	3.60	3.40
[Ne]$3s^2 3p^5 3d^9$	5.46	4.11	0.72	51.87
[Ne]$3s^1 3p^6 3d^9$	5.42	4.08	0.72	84.00

Co The energies of the low lying configurations of hard-wall confined Co are shown in fig. S59 for the neutral atom and in fig. S60 for the cation. The ground state of the unconfined Co is [Ar]$4s^1 3d^8$. At $r_c = 4.5a_0$ the ground state changes to [Ar]$3d^9$.

At $r_c = 1.9a_0$ the state [Ne]$3s^2 3p^5 3d^{10}$ flips below the initial ground state. At $r_c = 1.6a_0$ the state [Ne]$3s^1 3p^6 3d^{10}$ flips below the initial ground state. At $r_c = 1.1a_0$ the state [Ar]$3d^8 4p^1$ flips below the initial ground state. The ionization energy of the unconfined atom is $\Delta E_0 = 7.49$ eV. The studied configurations, atomic radii and excitation energies in the unconfined calculations are as follows:

configuration	r_ϵ	r_ρ	$r_{\max}$	ΔE
[Ar]$4s^1 3d^8$	5.47	4.26	2.48	0.00
[Ar]$3d^9$	5.51	4.08	0.68	0.87
[Ar]$4s^2 3d^7$	5.47	4.45	2.35	1.54
[Ar]$3d^8 4p^1$	8.05	4.45	3.52	3.59
[Ne]$3s^2 3p^5 3d^{10}$	5.20	4.02	0.68	56.38
[Ne]$3s^1 3p^6 3d^{10}$	5.17	4.02	0.68	90.99

Ni The energies of the low lying configurations of hard-wall confined Ni are shown in fig. S61 for the neutral atom and in fig. S62 for the cation. The ground state of the unconfined Ni is [Ar]$4s^1 3d^9$. At $r_c = 4.7a_0$ the ground state changes to [Ar]$3d^{10}$.

At $r_c = 1.4a_0$ the state [Ar]$3d^9 4f^1$ flips below the initial ground state. At $r_c = 1.2a_0$ the state [Ne]$3s^2 3p^5 3d^{10} 4f^1$ flips below the initial ground state. At $r_c = 1.0a_0$ the state [Ar]$3d^9 4p^1$ flips below the initial ground state. The ionization energy of the unconfined atom is $\Delta E_0 = 7.70$ eV. The studied configurations, atomic radii and excitation energies in the unconfined calculations are as follows:

configuration	r_ϵ	r_ρ	$r_{\max}$	ΔE
[Ar]$4s^1 3d^9$	5.33	4.18	2.39	0.00
[Ar]$3d^{10}$	5.25	4.02	0.65	0.65
[Ar]$4s^2 3d^8$	5.34	4.39	2.27	1.98
[Ar]$3d^9 4p^1$	7.99	4.39	3.44	3.77
[Ar]$3d^9 4f^1$	24.81	3.45	16.74	6.80
[Ne]$3s^2 3p^5 3d^{10} 4f^1$	24.82	3.41	16.77	67.79

Cu The energies of the low lying configurations of hard-wall confined Cu are shown in fig. S63 for the neutral atom and in fig. S64 for the cation. The ground state of the unconfined Cu is [Ar]$4s^1 3d^{10}$. At $r_c = 1.4a_0$ the ground state changes to [Ar]$3d^{10} 4f^1$.

At $r_c = 1.1a_0$ the state [Ar]$3d^{10} 4d^1$ flips below the initial ground state. At $r_c = 1.0a_0$ the state [Ar]$3d^{10} 4p^1$ flips below the initial ground state. The ionization energy of the unconfined atom is $\Delta E_0 = 7.91$ eV. The studied configurations, atomic radii and excitation energies in the unconfined calculations are as follows:

configuration	r_ϵ	r_ρ	$r_{\max}$	ΔE
[Ar]$4s^1 3d^{10}$	5.19	4.13	2.32	0.00
[Ar]$4s^2 3d^9$	5.22	4.31	2.20	2.40
[Ar]$3d^{10} 4p^1$	7.94	4.35	3.38	3.94
[Ar]$3d^{10} 4d^1$	16.05	3.41	9.31	6.34
[Ar]$3d^{10} 4f^1$	24.81	3.45	16.75	7.00

Zn The energies of the low lying configurations of hard-wall confined Zn are shown in fig. S65 for the neutral atom and in fig. S66 for the cation. The ground state of the unconfined Zn is [Ar]$4s^2 3d^{10}$. At $r_c = 1.3a_0$ the ground state changes to [Ar]$3d^{10} 4f^2$.

At $r_c = 1.3a_0$ the state [Ar]$4s^1 3d^{10} 4f^1$ flips below the initial ground state. At $r_c = 1.2a_0$ the state [Ar]$3d^{10} 4d^1 4f^1$ flips below the initial ground state. At $r_c = 1.2a_0$ the state [Ar]$3d^{10} 4p^1 4f^1$ flips below the initial ground state. At $r_c = 1.1a_0$ the state [Ar]$4s^1 3d^{10} 4d^1$ flips below the initial ground state. At $r_c = 1.0a_0$ the state [Ar]$4s^1 3d^{10} 4p^1$ flips below the initial ground state. At $r_c = 1.0a_0$ the state [Ar]$3d^{10} 4p^2$ flips below the initial ground state. The ionization energy of the unconfined atom is $\Delta E_0 = 9.67$ eV. The studied configurations, atomic radii and excitation energies in the unconfined calculations are as follows:

configuration	r_ϵ	r_ρ	$r_{\max}$	ΔE
[Ar]$4s^2 3d^{10}$	5.11	4.22	2.13	0.00
[Ar]$4s^1 3d^{10} 4p^1$	6.85	4.59	2.90	4.87
[Ar]$4s^1 3d^{10} 4d^1$	15.62	3.83	8.86	8.04
[Ar]$4s^1 3d^{10} 4f^1$	24.76	3.71	16.71	8.76
[Ar]$3d^{10} 4p^2$	7.03	4.89	2.80	10.10
[Ar]$3d^{10} 4p^1 4f^1$	24.56	4.05	16.49	14.80
[Ar]$3d^{10} 4d^1 4f^1$	21.58	3.78	12.94	20.75
[Ar]$3d^{10} 4f^2$	18.26	2.97	9.66	22.78

Ga The energies of the low lying configurations of hard-wall confined Ga are shown in fig. S67 for the neutral atom and in fig. S68 for the cation. The ground state of the unconfined Ga is [Ar]$4s^2 3d^{10} 4p^1$. At $r_c = 1.3a_0$ the ground state changes to [Ar]$4s^2 3d^{10} 4f^1$. Furthermore, at $r_c = 1.2a_0$ we see a ground state crossing to [Ar]$3d^{10} 4f^3$.

At $r_c = 1.3a_0$ the state [Ar]$4s^1 3d^{10} 4f^2$ flips below the initial ground state. At $r_c = 1.2a_0$ the state [Ar]$3d^{10} 4d^1 4f^2$ flips below the initial ground state. At $r_c = 1.2a_0$ the state [Ar]$3d^{10} 4p^1 4f^2$ flips below the initial ground state. At $r_c = 1.1a_0$ the state [Ar]$4s^2 3d^{10} 4d^1$ flips below the initial ground state. The ionization energy of the unconfined atom is $\Delta E_0 =$ 5.72 eV. The studied configurations, atomic radii and excitation energies in the unconfined calculations are as follows:

configuration	r_ϵ	r_ρ	$r_{\max}$	ΔE
[Ar]$4s^2 3d^{10} 4p^1$	6.04	4.51	2.55	0.00
[Ar]$4s^2 3d^{10} 5s^1$	11.19	3.98	6.59	2.80
[Ar]$4s^2 3d^{10} 4d^1$	15.31	3.94	8.52	4.05
[Ar]$4s^2 3d^{10} 4f^1$	24.75	3.83	16.70	4.81
[Ar]$4s^1 3d^{10} 4p^2$	6.28	4.78	2.47	6.25
[Ar]$4s^1 3d^{10} 4f^2$	18.21	3.45	9.58	21.48
[Ar]$3d^{10} 4p^1 4f^2$	18.14	3.78	9.48	29.45
[Ar]$3d^{10} 4d^1 4f^2$	17.26	5.17	8.12	38.87
[Ar]$3d^{10} 4f^3$	15.21	5.69	6.73	42.94

Ge The energies of the low lying configurations of hard-wall confined Ge are shown in fig. S69 for the neutral atom and in fig. S70 for the cation. The ground state of the unconfined Ge is [Ar]$4s^2 3d^{10} 4p^2$. At $r_c = 1.3a_0$ the ground state changes to [Ar]$4s^2 3d^{10} 4f^2$. Furthermore, at $r_c = 1.2a_0$ we see a ground state crossing to [Ar]$3d^{10} 4f^4$.

At $r_c = 1.3a_0$ the state [Ar]$4s^1 3d^{10} 4f^3$ flips below the initial ground state. At $r_c = 1.3a_0$ the state [Ar]$4s^2 3d^{10} 4p^1 4f^1$ flips below the initial ground state. At $r_c = 1.2a_0$ the state [Ar]$3d^{10} 4d^1 4f^3$ flips below the initial ground state. At $r_c = 1.0a_0$ the state [Ar]$4s^2 3d^{10} 4p^1 4d^1$ flips below the initial ground state. The ionization energy of the unconfined atom is $\Delta E_0 =$ 7.48 eV. The studied configurations, atomic radii and excitation energies in the unconfined calculations are as follows:

configuration	r_ϵ	r_ρ	$r_{\max}$	ΔE
[Ar]$4s^2 3d^{10} 4p^2$	5.69	4.51	2.22	0.00
[Ar]$4s^2 3d^{10} 4p^1 5s^1$	10.37	4.45	5.94	4.27
[Ar]$4s^2 3d^{10} 4p^1 4d^1$	14.64	4.19	7.73	5.70
[Ar]$4s^2 3d^{10} 4p^1 4f^1$	24.68	4.12	16.60	6.57
[Ar]$4s^1 3d^{10} 4p^3$	5.75	4.67	2.18	7.59
[Ar]$4s^2 3d^{10} 4f^2$	18.19	3.66	9.55	17.91
[Ar]$4s^1 3d^{10} 4f^3$	15.19	5.69	6.66	42.75
[Ar]$3d^{10} 4d^1 4f^3$	14.85	6.34	5.87	65.29
[Ar]$3d^{10} 4f^4$	13.44	6.84	5.12	71.83

As The energies of the low lying configurations of hard-wall confined As are shown in fig. S71 for the neutral atom and in fig. S72 for the cation. The ground state of the unconfined As is [Ar]$4s^2 3d^{10} 4p^3$. At $r_c = 1.2a_0$ the ground state changes to [Ar]$4s^1 3d^{10} 4f^4$. Furthermore, at $r_c = 1.1a_0$ we see a ground state crossing to [Ar]$3d^{10} 4f^5$.

At $r_c = 1.2a_0$ the state [Ar]$3d^{10} 4d^1 4f^4$ flips below the initial ground state. At $r_c = 1.2a_0$ the state [Ar]$4s^2 3d^{10} 4f^3$ flips below the initial ground state. At $r_c = 1.2a_0$ the state [Ar]$4s^2 3d^{10} 4p^2 4f^1$ flips below the initial ground state. At $r_c = 1.0a_0$ the state [Ar]$4s^2 3d^{10} 4p^2 4d^1$ flips below the initial ground state. The ionization energy of the unconfined atom is $\Delta E_0 =$ 9.18 eV. The studied configurations, atomic radii and excitation energies in the unconfined calculations are as follows:

configuration	r_ϵ	r_ρ	$r_{\max}$	ΔE
[Ar]$4s^2 3d^{10} 4p^3$	5.30	4.45	1.99	0.00
[Ar]$4s^2 3d^{10} 4p^2 5s^1$	9.82	4.67	5.49	5.75
[Ar]$4s^2 3d^{10} 4p^2 4d^1$	14.30	4.26	7.21	7.33
[Ar]$4s^2 3d^{10} 4p^2 4f^1$	24.69	4.12	16.61	8.27
[Ar]$4s^1 3d^{10} 4p^4$	5.32	4.51	1.97	8.93
[Ar]$4s^2 3d^{10} 4f^3$	15.19	5.57	6.62	40.28
[Ar]$4s^1 3d^{10} 4f^4$	13.44	6.84	5.04	73.37
[Ar]$3d^{10} 4d^1 4f^4$	13.33	6.84	4.56	100.81
[Ar]$3d^{10} 4f^5$	12.27	6.84	4.12	110.12

Se The energies of the low lying configurations of hard-wall confined Se are shown in fig. S73 for the neutral atom and in fig. S74 for the cation. The ground state of the unconfined Se is [Ar]$4s^2 3d^{10} 4p^4$. At $r_c = 1.2a_0$ the ground state changes to [Ar]$4s^2 3d^{10} 4f^4$. Furthermore, at $r_c = 1.1a_0$ we see a ground state crossing to [Ar]$3d^{10} 4f^6$.

At $r_c = 1.2a_0$ the state [Ar]$4s^1 3d^{10} 4f^5$ flips below the initial ground state. At $r_c = 1.2a_0$ the state [Ar]$4s^2 3d^{10} 4p^1 4f^3$ flips below the initial ground state. At $r_c = 1.2a_0$ the state [Ar]$4s^2 3d^{10} 4p^3 4f^1$ flips below the initial ground state. At $r_c = 1.1a_0$ the state [Ar]$3d^{10} 4d^1 4f^5$ flips below the initial ground state. At $r_c = 1.0a_0$ the state [Ar]$4s^2 3d^{10} 4p^3 4d^1$ flips below the initial ground state. The ionization energy of the unconfined atom is $\Delta E_0 = 10.87$ eV. The studied configurations, atomic radii and excitation energies in the unconfined calculations are as follows:

configuration	r_ϵ	r_ρ	$r_{\max}$	ΔE
[Ar]$4s^2 3d^{10} 4p^4$	4.95	4.31	1.82	0.00
[Ar]$4s^2 3d^{10} 4p^3 5s^1$	9.40	4.67	5.16	7.25
[Ar]$4s^2 3d^{10} 4p^3 4d^1$	14.12	4.20	6.91	8.98
[Ar]$4s^2 3d^{10} 4p^3 4f^1$	24.73	3.98	16.68	9.96
[Ar]$4s^1 3d^{10} 4p^5$	4.96	4.35	1.80	10.26
[Ar]$4s^2 3d^{10} 4p^1 4f^3$	15.18	5.69	6.55	47.45
[Ar]$4s^2 3d^{10} 4f^4$	13.46	6.84	4.99	72.62
[Ar]$4s^1 3d^{10} 4f^5$	12.30	6.84	4.03	113.89
[Ar]$3d^{10} 4d^1 4f^5$	12.29	6.58	3.71	146.06
[Ar]$3d^{10} 4f^6$	11.45	6.69	3.43	158.32

Br The energies of the low lying configurations of hard-wall confined Br are shown in fig. S75 for the neutral atom and in fig. S76 for the cation. The ground state of the unconfined Br is [Ar]$4s^2 3d^{10} 4p^5$. At $r_c = 1.2a_0$ the ground state changes to [Ar]$4s^2 3d^{10} 4p^4 4f^1$. Furthermore, at $r_c = 1.1a_0$ we see a ground state crossing to [Ar]$3d^{10} 4f^7$.

At $r_c = 1.1a_0$ the state [Ar]$4s^1 3d^{10} 4f^6$ flips below the initial ground state. At $r_c = 1.1a_0$ the state [Ar]$3d^{10} 4p^1 4f^6$ flips below the initial ground state. At $r_c = 1.1a_0$ the state [Ar]$4s^2 3d^{10} 4p^3 4f^2$ flips below the initial ground state. The ionization energy of the unconfined atom is $\Delta E_0 = 12.58$ eV. The studied configurations, atomic radii and excitation energies in the unconfined calculations are as follows:

configuration	r_ϵ	r_ρ	$r_{\max}$	ΔE
[Ar]$4s^2 3d^{10} 4p^5$	4.65	4.17	1.68	0.00
[Ar]$4s^2 3d^{10} 4p^4 5s^1$	9.05	4.67	4.88	8.79
[Ar]$4s^2 3d^{10} 4p^4 4d^1$	14.06	4.13	6.76	10.66
[Ar]$4s^1 3d^{10} 4p^6$	4.65	4.20	1.66	11.61
[Ar]$4s^2 3d^{10} 4p^4 4f^1$	24.75	4.01	16.68	11.67
[Ar]$4s^2 3d^{10} 4p^3 4f^2$	18.15	3.78	9.48	30.29
[Ar]$4s^1 3d^{10} 4f^6$	11.51	6.58	3.34	164.78
[Ar]$3d^{10} 4p^1 4f^6$	11.53	6.58	3.28	179.57
[Ar]$3d^{10} 4f^7$	10.85	6.39	2.94	216.92

Kr The energies of the low lying configurations of hard-wall confined Kr are shown in fig. S77 for the neutral atom and in fig. S78 for the cation. The ground state of the unconfined Kr is [Ar]$4s^2 3d^{10} 4p^6$. At $r_c = 1.1a_0$ the ground state changes to [Ar]$4s^1 3d^{10} 4f^7$. Furthermore, at $r_c = 1.0a_0$ we see a ground state crossing to [Ar]$3d^{10} 4f^8$.

At $r_c = 1.1a_0$ the state [Ar]$3d^{10} 4p^1 4f^7$ flips below the initial ground state. At $r_c = 1.1a_0$ the state [Ar]$4s^2 3d^{10} 4p^5 4f^1$ flips below the initial ground state. At $r_c = 1.1a_0$ the state [Ar]$4s^2 3d^{10} 4p^4 4f^2$ flips below the initial ground state. The ionization energy of the unconfined atom is $\Delta E_0 = 14.31$ eV. The studied configurations, atomic radii and excitation energies in the unconfined calculations are as follows:

configuration	r_ϵ	r_ρ	$r_{\max}$	ΔE
$[Ar]4s^2 3d^{10} 4p^6$	4.39	4.08	1.56	0.00
$[Ar]4s^2 3d^{10} 4p^5 5s^1$	8.76	4.59	4.66	10.37
$[Ar]4s^2 3d^{10} 4p^5 4d^1$	14.06	4.05	6.72	12.37
$[Ar]4s^2 3d^{10} 4p^5 4f^1$	24.78	3.89	16.71	13.41
$[Ar]4s^1 3d^{10} 4p^6 4d^1$	13.87	4.08	6.21	25.86
$[Ar]4s^2 3d^{10} 4p^4 4f^2$	18.16	3.66	9.49	34.44
$[Ar]4s^1 3d^{10} 4f^7$	10.94	6.58	2.84	226.47
$[Ar]3d^{10} 4p^1 4f^7$	10.97	6.34	2.80	242.83
$[Ar]3d^{10} 4f^8$	10.41	6.16	2.56	286.33

Rb The energies of the low lying configurations of hard-wall confined Rb are shown in fig. S79 for the neutral atom and in fig. S80 for the cation. The ground state of the unconfined Rb is [Kr]$5s^1$. At $r_c = 4.9a_0$ the ground state changes to [Kr]$4d^1$. Furthermore, at $r_c = 1.2a_0$ we see a ground state crossing to [Kr]$4f^1$. At $r_c = 1.1a_0$ we see a ground state crossing to [Ar]$4s^2 3d^{10} 4p^5 4f^2$. Moreover, at $r_c = 1.0a_0$ we see a ground state crossing to [Ar]$3d^{10} 4f^9$.

At $r_c = 3.0a_0$ the state [Ar]$4s^2 3d^{10} 4p^5 4d^2$ flips below the initial ground state. At $r_c = 1.3a_0$ the state [Ar]$4s^1 3d^{10} 4f^8$ flips below the initial ground state. At $r_c = 1.2a_0$ the state [Ar]$3d^{10} 4p^1 4f^8$ flips below the initial ground state. At $r_c = 1.1a_0$ the state [Kr]$5p^1$ flips below the initial ground state. The ionization energy of the unconfined atom is $\Delta E_0 = 4.08$ eV. The studied configurations, atomic radii and excitation energies in the unconfined calculations are as follows:

configuration	r_ϵ	r_ρ	$r_{\max}$	ΔE
$[Kr]5s^1$	8.51	4.59	4.46	0.00
$[Kr]5p^1$	11.36	4.01	6.28	1.45
$[Kr]4d^1$	14.10	3.94	6.78	2.14
$[Kr]4f^1$	24.80	3.78	16.73	3.18
$[Ar]4s^2 3d^{10} 4p^5 4d^2$	10.29	4.78	2.46	19.63
$[Ar]4s^2 3d^{10} 4p^5 4f^2$	18.17	3.55	9.51	26.64
$[Ar]4s^1 3d^{10} 4f^8$	10.53	6.34	2.47	287.32
$[Ar]3d^{10} 4p^1 4f^8$	10.55	6.34	2.44	305.25
$[Ar]3d^{10} 4f^9$	10.08	6.06	2.27	354.93

Sr The energies of the low lying configurations of hard-wall confined Sr are shown in fig. S81 for the neutral atom and in fig. S82 for the cation. The ground state of the unconfined Sr is [Kr]$5s^2$. At $r_c = 5.0a_0$ the ground state changes to [Kr]$5s^1 4d^1$. Furthermore, at $r_c = 4.9a_0$ we see a ground state crossing to [Kr]$4d^2$. At $r_c = 1.2a_0$ we see a ground state crossing to [Kr]$4f^2$. Moreover, at $r_c = 1.0a_0$ we see a ground state crossing to [Ar]$3d^{10} 4f^{10}$.

At $r_c = 3.4a_0$ the state [Kr]$4d^1 4f^1$ flips below the initial ground state. At $r_c = 3.4a_0$ the state [Ar]$4s^2 3d^{10} 4p^5 4d^3$ flips below the initial ground state. At $r_c = 1.5a_0$ the state [Ar]$4s^2 3d^{10} 4f^8$ flips below the initial ground state. At $r_c = 1.4a_0$ the state [Ar]$4s^1 3d^{10} 4f^9$ flips below the initial ground state. At $r_c = 1.1a_0$ the state [Kr]$5s^1 5p^1$ flips below the initial ground state. The ionization energy of the unconfined atom is $\Delta E_0 = 5.80$ eV. The studied configurations, atomic radii and excitation energies in the unconfined calculations are as follows:

configuration	r_ϵ	r_ρ	$r_{\max}$	ΔE
$[Kr]5s^2$	7.72	5.50	3.77	0.00
$[Kr]5s^1 4d^1$	8.47	5.17	3.86	2.06
$[Kr]5s^1 5p^1$	9.15	5.69	4.79	2.19
$[Kr]4d^2$	10.26	4.67	2.24	4.24
$[Kr]4d^1 4f^1$	24.46	4.02	16.39	6.29
$[Kr]4f^2$	18.18	3.55	9.53	11.62
$[Ar]4s^2 3d^{10} 4p^5 4d^3$	8.53	5.02	2.02	25.32
$[Ar]4s^2 3d^{10} 4f^8$	10.64	6.34	2.40	278.94
$[Ar]4s^1 3d^{10} 4f^9$	10.22	6.11	2.19	352.43
$[Ar]3d^{10} 4f^{10}$	9.83	6.11	2.04	427.83

Y The energies of the low lying configurations of hard-wall confined Y are shown in fig. S83 for the neutral atom and in fig. S84 for the cation. The ground state of the unconfined Y is [Kr]$5s^2 4d^1$. At $r_c = 5.1a_0$ the ground state changes to [Kr]$5s^1 4d^2$. Furthermore, at $r_c = 4.9a_0$ we see a ground state crossing to [Kr]$4d^3$. At $r_c = 1.2a_0$ we see a ground state crossing to [Kr]$4d^2 4f^1$. Moreover, at $r_c = 1.1a_0$ we see a ground state crossing to [Kr]$4d^1 4f^2$. At $r_c = 1.0a_0$ we see a ground state crossing to [Ar]$4s^2 3d^{10} 4f^9$.

At $r_c = 3.5a_0$ the state [Kr]$4d^2 5p^1$ flips below the initial ground state. At $r_c = 3.3a_0$ the state [Ar]$4s^2 3d^{10} 4p^5 4d^4$ flips below the initial ground state. At $r_c = 1.4a_0$ the state [Ar]$4s^2 3d^{10} 4p^1 4f^8$ flips below the initial ground state. At $r_c = 1.3a_0$ the state [Ar]$4s^1 3d^{10} 4f^{10}$ flips below the initial ground state. The ionization energy of

the unconfined atom is $\Delta E_0 = 6.47$ eV. The studied configurations, atomic radii and excitation energies in the unconfined calculations are as follows:

configuration	r_ϵ	r_ρ	$r_{\max}$	ΔE
$[Kr]5s^2 4d^1$	7.23	5.33	3.45	0.00
$[Kr]5s^1 4d^2$	7.53	5.17	3.55	1.45
$[Kr]5s^2 5p^1$	7.97	5.69	4.06	2.12
$[Kr]4d^3$	8.46	4.89	1.87	3.30
$[Kr]4d^2 5p^1$	9.28	5.17	4.71	3.73
$[Kr]4d^2 4f^1$	24.50	4.15	16.43	6.21
$[Kr]4d^1 4f^2$	18.12	3.78	9.44	13.23
$[Ar]4s^2 3d^{10} 4p^5 4d^4$	7.46	4.89	1.78	27.62
$[Ar]4s^2 3d^{10} 4p^1 4f^8$	10.78	6.34	2.30	280.04
$[Ar]4s^2 3d^{10} 4f^9$	10.36	6.11	2.11	339.01
$[Ar]4s^1 3d^{10} 4f^{10}$	10.00	5.90	1.96	420.53

Zr The energies of the low lying configurations of hard-wall confined Zr are shown in fig. S85 for the neutral atom and in fig. S86 for the cation. The ground state of the unconfined Zr is $[Kr]5s^2 4d^2$. At $r_c = 5.4a_0$ the ground state changes to $[Kr]5s^1 4d^3$. Furthermore, at $r_c = 5.0a_0$ we see a ground state crossing to $[Kr]4d^4$. At $r_c = 1.1a_0$ we see a ground state crossing to $[Kr]4d^2 4f^2$. Moreover, at $r_c = 1.0a_0$ we see a ground state crossing to $[Ar]4s^2 3d^{10} 4f^{10}$.

At $r_c = 3.4a_0$ the state $[Kr]4d^3 5p^1$ flips below the initial ground state. At $r_c = 3.2a_0$ the state $[Kr]4d^3 4f^1$ flips below the initial ground state. At $r_c = 3.2a_0$ the state $[Ar]4s^2 3d^{10} 4p^5 4d^5$ flips below the initial ground state. At $r_c = 1.4a_0$ the state $[Ar]4s^2 3d^{10} 4p^1 4f^9$ flips below the initial ground state. At $r_c = 1.4a_0$ the state $[Ar]4s^2 3d^{10} 4p^2 4f^8$ flips below the initial ground state. The ionization energy of the unconfined atom is $\Delta E_0 = 6.78$ eV. The studied configurations, atomic radii and excitation energies in the unconfined calculations are as follows:

configuration	r_ϵ	r_ρ	$r_{\max}$	ΔE
$[Kr]5s^2 4d^2$	6.85	5.33	3.23	0.00
$[Kr]5s^1 4d^3$	6.97	5.02	3.34	0.74
$[Kr]4d^4$	7.37	4.89	1.66	2.13
$[Kr]4d^3 5p^1$	8.93	5.17	4.42	3.25
$[Kr]4d^3 4f^1$	24.56	4.15	16.49	5.86
$[Kr]4d^2 4f^2$	18.12	3.78	9.42	14.46
$[Ar]4s^2 3d^{10} 4p^5 4d^5$	6.73	4.78	1.60	29.55
$[Ar]4s^2 3d^{10} 4p^2 4f^8$	10.90	6.34	2.23	275.80
$[Ar]4s^2 3d^{10} 4p^1 4f^9$	10.52	6.11	2.02	339.80
$[Ar]4s^2 3d^{10} 4f^{10}$	10.16	5.90	1.89	405.44

Nb The energies of the low lying configurations of hard-wall confined Nb are shown in fig. S87 for the neutral atom and in fig. S88 for the cation. The ground state of the unconfined Nb is $[Kr]5s^1 4d^4$. At $r_c = 5.3a_0$ the ground state changes to $[Kr]4d^5$. Furthermore, at $r_c = 1.1a_0$ we see a ground state crossing to $[Kr]4f^5$.

At $r_c = 2.6a_0$ the state $[Ar]4s^2 3d^{10} 4p^5 4d^6$ flips below the initial ground state. At $r_c = 2.5a_0$ the state $[Kr]4d^4 4f^1$ flips below the initial ground state. At $r_c = 2.0a_0$ the state $[Kr]4d^3 4f^2$ flips below the initial ground state. At $r_c = 1.4a_0$ the state $[Ar]4s^2 3d^{10} 4p^5 4f^6$ flips below the initial ground state. At $r_c = 1.3a_0$ the state $[Ar]4s^2 3d^{10} 4p^4 4f^7$ flips below the initial ground state. At $r_c = 1.0a_0$ the state $[Kr]4d^4 5p^1$ flips below the initial ground state. The ionization energy of the unconfined atom is $\Delta E_0 = 6.31$ eV. The studied configurations, atomic radii and excitation energies in the unconfined calculations are as follows:

configuration	r_ϵ	r_ρ	$r_{\max}$	ΔE
$[Kr]5s^1 4d^4$	6.59	4.89	3.18	0.00
$[Kr]5s^2 4d^3$	6.57	5.17	3.07	0.02
$[Kr]4d^5$	6.63	4.67	1.51	0.87
$[Kr]4d^4 5p^1$	8.72	5.02	4.22	2.70
$[Kr]4d^4 4f^1$	24.62	4.08	16.56	5.39
$[Kr]4d^3 4f^2$	18.13	3.89	9.43	15.48
$[Ar]4s^2 3d^{10} 4p^5 4d^6$	6.18	4.67	1.47	31.32
$[Kr]4f^5$	12.56	6.58	3.79	73.53
$[Ar]4s^2 3d^{10} 4p^5 4f^6$	11.90	6.58	3.03	134.77
$[Ar]4s^2 3d^{10} 4p^4 4f^7$	11.41	6.34	2.50	199.30

Mo The energies of the low lying configurations of hard-wall confined Mo are shown in

fig. S89 for the neutral atom and in fig. S90 for the cation. The ground state of the unconfined Mo is [Kr]$5s^1 4d^5$. At $r_c = 6.2a_0$ the ground state changes to [Kr]$4d^6$. Furthermore, at $r_c = 1.0a_0$ we see a ground state crossing to [Kr]$4f^6$.

At $r_c = 2.5a_0$ the state [Ar]$4s^2 3d^{10} 4p^5 4d^7$ flips below the initial ground state. At $r_c = 2.4a_0$ the state [Kr]$4d^5 4f^1$ flips below the initial ground state. At $r_c = 1.9a_0$ the state [Kr]$4d^4 4f^2$ flips below the initial ground state. At $r_c = 1.4a_0$ the state [Kr]$4d^1 4f^5$ flips below the initial ground state. At $r_c = 1.3a_0$ the state [Ar]$4s^2 3d^{10} 4p^5 4f^7$ flips below the initial ground state. At $r_c = 1.0a_0$ the state [Kr]$4d^5 5p^1$ flips below the initial ground state. The ionization energy of the unconfined atom is $\Delta E_0 = 6.52$ eV. The studied configurations, atomic radii and excitation energies in the unconfined calculations are as follows:

configuration	r_ϵ	r_ρ	$r_{\max}$	ΔE
[Kr]$5s^1 4d^5$	6.31	4.78	3.05	0.00
[Kr]$4d^6$	6.08	4.59	1.39	0.31
[Kr]$5s^2 4d^4$	6.34	5.02	2.94	0.81
[Kr]$4d^5 5p^1$	8.58	4.89	4.07	2.85
[Kr]$4d^5 4f^1$	24.67	4.02	16.61	5.60
[Kr]$4d^4 4f^2$	18.14	3.78	9.45	17.14
[Ar]$4s^2 3d^{10} 4p^5 4d^7$	5.74	4.51	1.37	33.74
[Kr]$4d^1 4f^5$	12.62	6.58	3.72	82.44
[Kr]$4f^6$	11.93	6.58	3.03	110.80
[Ar]$4s^2 3d^{10} 4p^5 4f^7$	11.48	6.34	2.47	180.32

Tc The energies of the low lying configurations of hard-wall confined Tc are shown in fig. S91 for the neutral atom and in fig. S92 for the cation. The ground state of the unconfined Tc is [Kr]$4d^7$. At $r_c = 1.0a_0$ the ground state changes to [Kr]$4f^7$.

At $r_c = 1.0a_0$ the state [Kr]$4d^1 4f^6$ flips below the initial ground state. At $r_c = 1.0a_0$ the state [Kr]$4d^2 4f^5$ flips below the initial ground state. At $r_c = 1.0a_0$ the state [Kr]$4d^6 4f^1$ flips below the initial ground state. At $r_c = 1.0a_0$ the state [Kr]$4d^5 4f^2$ flips below the initial ground state. The ionization energy of the unconfined atom is $\Delta E_0 = 6.97$ eV. The studied configurations, atomic radii and excitation energies in the unconfined calculations are as follows:

configuration	r_ϵ	r_ρ	$r_{\max}$	ΔE
[Kr]$4d^7$	5.65	4.45	1.30	0.00
[Kr]$5s^1 4d^6$	6.09	4.67	2.94	0.28
[Kr]$5s^2 4d^5$	6.16	4.89	2.83	1.91
[Kr]$4d^6 5p^1$	8.49	4.78	3.95	3.26
[Kr]$4d^6 4f^1$	24.71	3.94	16.65	6.06
[Kr]$4d^5 4f^2$	18.15	3.78	9.47	19.00
[Ar]$4s^2 3d^{10} 4p^5 4d^8$	5.39	4.35	1.28	36.41
[Kr]$4d^2 4f^5$	12.67	6.58	3.68	91.54
[Kr]$4d^1 4f^6$	12.02	6.58	2.95	122.88
[Kr]$4f^7$	11.53	6.34	2.46	156.32

Ru The energies of the low lying configurations of hard-wall confined Ru are shown in fig. S93 for the neutral atom and in fig. S94 for the cation. The ground state of the unconfined Ru is [Kr]$4d^8$. At $r_c = 1.0a_0$ the ground state changes to [Kr]$4d^2 4f^6$.

At $r_c = 1.0a_0$ the state [Kr]$4d^3 4f^5$ flips below the initial ground state. At $r_c = 1.0a_0$ the state [Kr]$4d^1 4f^7$ flips below the initial ground state. At $r_c = 1.0a_0$ the state [Kr]$4d^7 4f^1$ flips below the initial ground state. The ionization energy of the unconfined atom is $\Delta E_0 = 7.73$ eV. The studied configurations, atomic radii and excitation energies in the unconfined calculations are as follows:

configuration	r_ϵ	r_ρ	$r_{\max}$	ΔE
[Kr]$4d^8$	5.30	4.28	1.22	0.00
[Kr]$5s^1 4d^7$	5.92	4.59	2.85	0.89
[Kr]$5s^2 4d^6$	6.01	4.78	2.73	3.37
[Kr]$4d^7 5p^1$	8.43	4.67	3.86	3.99
[Kr]$4d^7 4f^1$	24.74	3.89	16.69	6.82
[Ar]$4s^2 3d^{10} 4p^5 4d^9$	5.09	4.22	1.20	39.37
[Kr]$4d^3 4f^5$	12.71	6.58	3.66	100.92
[Kr]$4d^2 4f^6$	12.10	6.58	2.89	135.28
[Kr]$4d^1 4f^7$	11.64	6.34	2.36	171.86

Rh The energies of the low lying configurations of hard-wall confined Rh are shown in fig. S95 for the neutral atom and in fig. S96 for the cation. The ground state of the unconfined Rh is [Kr]$4d^9$. We do not observe any ground state crossing for Rh in the considered confinement radii.

The ionization energy of the unconfined atom is $\Delta E_0 = 8.49$ eV. The studied configurations, atomic radii and excitation energies in the un-

confined calculations are as follows:

configuration	r_ϵ	r_ρ	$r_{\max}$	ΔE
[Kr]$4d^9$	5.00	4.16	1.15	0.00
[Kr]$5s^1 4d^8$	5.78	4.51	2.77	1.53
[Kr]$4d^8 5p^1$	8.39	4.59	3.79	4.73
[Kr]$5s^2 4d^7$	5.88	4.78	2.65	4.86
[Kr]$4d^8 4f^1$	24.76	3.89	16.69	7.58
[Kr]$4d^7 4f^2$	18.17	3.55	9.52	23.24
[Ar]$4s^2 3d^{10} 4p^5 4d^{10}$	4.83	4.13	1.14	42.35

Pd The energies of the low lying configurations of hard-wall confined Pd are shown in fig. S97 for the neutral atom and in fig. S98 for the cation. The ground state of the unconfined Pd is [Kr]$4d^{10}$. We do not observe any ground state crossing for Pd in the considered confinement radii.

The ionization energy of the unconfined atom is $\Delta E_0 = 9.25$ eV. The studied configurations, atomic radii and excitation energies in the unconfined calculations are as follows:

configuration	r_ϵ	r_ρ	$r_{\max}$	ΔE
[Kr]$4d^{10}$	4.75	4.08	1.09	0.00
[Kr]$5s^1 4d^9$	5.67	4.39	2.69	2.17
[Kr]$4d^9 5p^1$	8.36	4.51	3.72	5.47
[Kr]$5s^2 4d^8$	5.77	4.67	2.57	6.38
[Kr]$4d^9 4f^1$	24.78	3.71	16.73	8.35
[Kr]$4d^8 4f^2$	18.19	3.55	9.54	25.34
[Ar]$4s^2 3d^{10} 4p^5 4d^{10} 4f^1$	24.79	3.78	16.72	54.36

Ag The energies of the low lying configurations of hard-wall confined Ag are shown in fig. S99 for the neutral atom and in fig. S100 for the cation. The ground state of the unconfined Ag is [Kr]$5s^1 4d^{10}$. At $r_c = 2.1a_0$ the ground state changes to [Kr]$4d^{10}4f^1$.

At $r_c = 1.6a_0$ the state [Kr]$4d^9 4f^2$ flips below the initial ground state. At $r_c = 1.5a_0$ the state [Ar]$4s^2 3d^{10} 4p^5 4d^{10} 4f^2$ flips below the initial ground state. At $r_c = 1.4a_0$ the state [Kr]$4d^8 4f^3$ flips below the initial ground state. The ionization energy of the unconfined atom is $\Delta E_0 = 7.18$ eV. The studied configurations, atomic radii and excitation energies in the unconfined calculations are as follows:

configuration	r_ϵ	r_ρ	$r_{\max}$	ΔE
[Kr]$5s^1 4d^{10}$	5.57	4.31	2.63	0.00
[Kr]$4d^{10} 5p^1$	8.33	4.45	3.67	3.39
[Kr]$5s^2 4d^9$	5.67	4.59	2.50	5.11
[Kr]$4d^{10} 4f^1$	24.79	3.66	16.73	6.28
[Kr]$4d^9 4f^2$	18.20	3.45	9.56	24.60
[Kr]$4d^8 4f^3$	15.21	5.55	6.55	51.65
[Ar]$4s^2 3d^{10} 4p^5 4d^{10} 4f^2$	18.20	3.45	9.55	74.47

Cd The energies of the low lying configurations of hard-wall confined Cd are shown in fig. S101 for the neutral atom and in fig. S102 for the cation. The ground state of the unconfined Cd is [Kr]$5s^2 4d^{10}$. At $r_c = 2.0a_0$ the ground state changes to [Kr]$4d^{10}4f^2$.

At $r_c = 2.0a_0$ the state [Kr]$5s^1 4d^{10} 4f^1$ flips below the initial ground state. At $r_c = 1.8a_0$ the state [Kr]$4d^9 4f^3$ flips below the initial ground state. At $r_c = 1.8a_0$ the state [Kr]$4d^{10} 5p^1 4f^1$ flips below the initial ground state. At $r_c = 1.6a_0$ the state [Kr]$4d^8 4f^4$ flips below the initial ground state. At $r_c = 1.6a_0$ the state [Ar]$4s^2 3d^{10} 4p^5 4d^{10} 4f^3$ flips below the initial ground state. The ionization energy of the unconfined atom is $\Delta E_0 = 8.73$ eV. The studied configurations, atomic radii and excitation energies in the unconfined calculations are as follows:

configuration	r_ϵ	r_ρ	$r_{\max}$	ΔE
[Kr]$5s^2 4d^{10}$	5.58	4.51	2.44	0.00
[Kr]$5s^1 4d^{10} 5p^1$	7.22	4.78	3.22	4.14
[Kr]$5s^1 4d^{10} 5d^1$	15.56	4.05	8.79	7.09
[Kr]$5s^1 4d^{10} 4f^1$	24.68	4.01	16.60	7.82
[Kr]$4d^{10} 5p^2$	7.47	5.17	3.13	8.55
[Kr]$4d^{10} 5p^1 4f^1$	24.44	4.22	16.36	12.93
[Kr]$4d^{10} 4f^2$	18.21	3.34	9.57	19.99
[Kr]$4d^9 4f^3$	15.21	5.55	6.57	48.91
[Kr]$4d^8 4f^4$	13.61	6.84	4.86	85.83
[Ar]$4s^2 3d^{10} 4p^5 4d^{10} 4f^3$	15.21	5.55	6.56	102.70

In The energies of the low lying configurations of hard-wall confined In are shown in fig. S103 for the neutral atom and in fig. S104 for the cation. The ground state of the unconfined In is [Kr]$5s^2 4d^{10} 5p^1$. At $r_c = 2.2a_0$ the ground state changes to [Kr]$5s^2 4d^{10} 4f^1$. Furthermore, at $r_c = 2.0a_0$ we see a ground state crossing to [Kr]$4d^{10}4f^3$.

At $r_c = 2.1a_0$ the state [Kr]$5s^1 4d^{10} 4f^2$ flips below the initial ground state. At $r_c = 2.0a_0$ the state [Kr]$4d^{10} 5p^1 4f^2$ flips below the initial ground state. At $r_c = 1.8a_0$ the state [Kr]$4d^9 4f^4$ flips below the initial ground state. At $r_c = 1.7a_0$ the state [Kr]$4d^8 4f^5$ flips below the initial ground state. At $r_c = 1.7a_0$ the state [Ar]$4s^2 3d^{10} 4p^5 4d^{10} 4f^4$ flips below the initial ground state. At $r_c = 1.0a_0$ the state [Kr]$5s^2 4d^{10} 5d^1$ flips below the initial ground state. The ionization energy of the unconfined atom is $\Delta E_0 = 5.43$ eV. The studied configurations, atomic radii and excitation energies in the unconfined calculations are as follows:

configuration	r_ϵ	r_ρ	$r_{\max}$	ΔE
[Kr]$5s^2 4d^{10} 5p^1$	6.45	4.78	2.89	0.00
[Kr]$5s^2 4d^{10} 6s^1$	11.93	4.18	7.18	2.71
[Kr]$5s^2 4d^{10} 5d^1$	15.15	4.25	8.35	3.73
[Kr]$5s^2 4d^{10} 4f^1$	24.66	4.24	16.57	4.52
[Kr]$5s^1 4d^{10} 5p^2$	6.75	5.02	2.82	5.16
[Kr]$5s^1 4d^{10} 4f^2$	18.14	3.78	9.46	18.75
[Kr]$4d^{10} 5p^1 4f^2$	18.03	4.11	9.28	25.32
[Kr]$4d^{10} 4f^3$	15.21	5.55	6.59	36.40
[Kr]$4d^9 4f^4$	13.59	6.84	4.88	75.74
[Kr]$4d^8 4f^5$	12.74	6.58	3.75	121.56
[Ar]$4s^2 3d^{10} 4p^5 4d^{10} 4f^4$	13.61	6.84	4.87	133.43

Sn The energies of the low lying configurations of hard-wall confined Sn are shown in fig. S105 for the neutral atom and in fig. S106 for the cation. The ground state of the unconfined Sn is [Kr]$5s^2 4d^{10} 5p^2$. At $r_c = 2.2a_0$ the ground state changes to [Kr]$5s^2 4d^{10} 4f^2$. Furthermore, at $r_c = 1.9a_0$ we see a ground state crossing to [Kr]$4d^{10} 4f^4$.

At $r_c = 2.2a_0$ the state [Kr]$5s^2 4d^{10} 5p^1 4f^1$ flips below the initial ground state. At $r_c = 2.1a_0$ the state [Kr]$5s^1 4d^{10} 4f^3$ flips below the initial ground state. At $r_c = 1.9a_0$ the state [Kr]$4d^9 4f^5$ flips below the initial ground state. At $r_c = 1.8a_0$ the state [Ar]$4s^2 3d^{10} 4p^5 4d^{10} 4f^5$ flips below the initial ground state. At $r_c = 1.7a_0$ the state [Kr]$4d^8 4f^6$ flips below the initial ground state. The ionization energy of the unconfined atom is $\Delta E_0 = 6.94$ eV. The studied configurations, atomic radii and excitation energies in the unconfined calculations are as follows:

configuration	r_ϵ	r_ρ	$r_{\max}$	ΔE
[Kr]$5s^2 4d^{10} 5p^2$	6.18	4.89	2.59	0.00
[Kr]$5s^2 4d^{10} 5p^1 6s^1$	11.13	4.67	6.53	3.97
[Kr]$5s^2 4d^{10} 5p^1 5d^1$	14.20	4.67	7.09	5.09
[Kr]$5s^2 4d^{10} 5p^1 4f^1$	24.55	4.35	16.44	6.02
[Kr]$5s^1 4d^{10} 5p^3$	6.27	5.02	2.55	6.13
[Kr]$5s^2 4d^{10} 4f^2$	18.10	4.01	9.39	15.79
[Kr]$5s^1 4d^{10} 4f^3$	15.20	5.69	6.43	35.83
[Kr]$4d^{10} 4f^4$	13.58	6.84	4.91	58.07
[Kr]$4d^9 4f^5$	12.72	6.58	3.78	106.84
[Kr]$4d^8 4f^6$	12.27	6.58	2.91	160.34
[Ar]$4s^2 3d^{10} 4p^5 4d^{10} 4f^5$	12.75	6.58	3.76	168.33

Sb The energies of the low lying configurations of hard-wall confined Sb are shown in fig. S107 for the neutral atom and in fig. S108 for the cation. The ground state of the unconfined Sb is [Kr]$5s^2 4d^{10} 5p^3$. At $r_c = 2.1a_0$ the ground state changes to [Kr]$5s^2 4d^{10} 4f^3$. Furthermore, at $r_c = 1.9a_0$ we see a ground state crossing to [Kr]$4d^{10} 4f^5$.

At $r_c = 2.1a_0$ the state [Kr]$5s^1 4d^{10} 4f^4$ flips below the initial ground state. At $r_c = 2.1a_0$ the state [Kr]$5s^2 4d^{10} 5p^1 4f^2$ flips below the initial ground state. At $r_c = 2.1a_0$ the state [Kr]$5s^2 4d^{10} 5p^2 4f^1$ flips below the initial ground state. At $r_c = 1.9a_0$ the state [Kr]$4d^9 4f^6$ flips below the initial ground state. At $r_c = 1.8a_0$ the state [Kr]$4d^8 4f^7$ flips below the initial ground state. At $r_c = 1.8a_0$ the state [Ar]$4s^2 3d^{10} 4p^5 4d^{10} 4f^6$ flips below the initial ground state. The ionization energy of the unconfined atom is $\Delta E_0 = 8.37$ eV. The studied configurations, atomic radii and excitation energies in the unconfined calculations are as follows:

configuration	r_ϵ	r_ρ	$r_{\max}$	ΔE
[Kr]$5s^2 4d^{10} 5p^3$	5.83	4.87	2.37	0.00
[Kr]$5s^2 4d^{10} 5p^2 6s^1$	10.59	4.87	6.09	5.21
[Kr]$5s^2 4d^{10} 5p^2 5d^1$	13.56	4.77	5.41	6.40
[Kr]$5s^1 4d^{10} 5p^4$	5.87	4.87	2.34	7.08
[Kr]$5s^2 4d^{10} 5p^2 4f^1$	24.54	4.46	16.44	7.45
[Kr]$5s^2 4d^{10} 5p^1 4f^2$	18.04	4.24	9.25	19.17
[Kr]$5s^2 4d^{10} 4f^3$	15.25	5.49	6.31	33.57
[Kr]$5s^1 4d^{10} 4f^4$	13.72	6.53	4.66	58.14
[Kr]$4d^{10} 4f^5$	12.70	6.58	3.80	83.74
[Kr]$4d^9 4f^6$	12.26	6.58	2.95	140.59
[Kr]$4d^8 4f^7$	12.03	6.34	2.13	200.83
[Ar]$4s^2 3d^{10} 4p^5 4d^{10} 4f^6$	12.31	6.58	2.91	205.77

Te The energies of the low lying configurations of hard-wall confined Te are shown in fig. S109 for the neutral atom and in fig. S110 for the cation. The ground state of the unconfined Te is [Kr]$5s^2 4d^{10} 5p^4$. At $r_c = 2.1a_0$ the ground state changes to [Kr]$5s^2 4d^{10} 4f^4$. Furthermore, at $r_c = 1.8a_0$ we see a ground state crossing to [Kr]$4d^{10} 4f^6$.

At $r_c = 2.1a_0$ the state [Kr]$5s^2 4d^{10} 5p^1 4f^3$ flips below the initial ground state. At $r_c = 2.1a_0$ the state [Kr]$5s^2 4d^{10} 5p^3 4f^1$ flips below the initial ground state. At $r_c = 2.1a_0$ the state [Kr]$5s^2 4d^{10} 5p^2 4f^2$ flips below the initial ground state. At $r_c = 2.0a_0$ the state [Kr]$5s^1 4d^{10} 4f^5$ flips below the initial ground state. At $r_c = 2.0a_0$ the state [Kr]$4d^{10} 5p^1 4f^5$ flips below the initial ground state. At $r_c = 1.9a_0$ the state [Kr]$4d^9 4f^7$ flips below the initial ground state. At $r_c = 1.8a_0$ the state [Kr]$4d^8 4f^8$ flips below the initial ground state. At $r_c = 1.8a_0$ the state [Ar]$4s^2 3d^{10} 4p^5 4d^{10} 4f^7$ flips below the initial ground state. The ionization energy of the unconfined atom is $\Delta E_0 = 9.77$ eV. The studied configurations, atomic radii and excitation energies in the unconfined calculations are as follows:

configuration	r_ϵ	r_ρ	$r_{\max}$	ΔE
[Kr]$5s^2 4d^{10} 5p^4$	5.51	4.77	2.20	0.00
[Kr]$5s^2 4d^{10} 5p^3 6s^1$	10.17	4.87	5.74	6.45
[Kr]$5s^2 4d^{10} 5p^3 5d^1$	13.10	4.77	4.52	7.70
[Kr]$5s^1 4d^{10} 5p^5$	5.53	4.77	2.18	8.01
[Kr]$5s^2 4d^{10} 5p^3 4f^1$	24.57	4.46	16.48	8.86
[Kr]$5s^2 4d^{10} 5p^2 4f^2$	18.03	4.24	9.16	22.47
[Kr]$5s^2 4d^{10} 5p^1 4f^3$	15.39	5.37	6.06	38.82
[Kr]$5s^2 4d^{10} 4f^4$	13.96	6.27	4.40	56.28
[Kr]$5s^1 4d^{10} 4f^5$	13.01	6.39	3.41	83.80
[Kr]$4d^{10} 5p^1 4f^5$	13.08	6.27	3.19	93.42
[Kr]$4d^{10} 4f^6$	12.24	6.39	2.98	111.72
[Kr]$4d^9 4f^7$	12.03	6.16	2.18	175.58
[Kr]$4d^8 4f^8$	11.93	6.06	1.50	241.98
[Ar]$4s^2 3d^{10} 4p^5 4d^{10} 4f^7$	12.09	6.16	2.08	244.37

I The energies of the low lying configurations of hard-wall confined I are shown in fig. S111 for the neutral atom and in fig. S112 for the cation. The ground state of the unconfined I is [Kr]$5s^2 4d^{10} 5p^5$. At $r_c = 2.1a_0$ the ground state changes to [Kr]$5s^2 4d^{10} 5p^4 4f^1$. Furthermore, at $r_c = 2.0a_0$ we see a ground state crossing to [Kr]$5s^2 4d^{10} 4f^5$. At $r_c = 1.8a_0$ we see a ground state crossing to [Kr]$4d^{10} 4f^7$.

At $r_c = 2.1a_0$ the state [Kr]$5s^2 4d^{10} 5p^3 4f^2$ flips below the initial ground state. At $r_c = 2.0a_0$ the state [Kr]$5s^1 4d^{10} 4f^6$ flips below the initial ground state. At $r_c = 2.0a_0$ the state [Kr]$5s^2 4d^{10} 5p^1 4f^4$ flips below the initial ground state. At $r_c = 2.0a_0$ the state [Kr]$5s^2 4d^{10} 5p^2 4f^3$ flips below the initial ground state. At $r_c = 1.9a_0$ the state [Kr]$4d^{10} 5p^1 4f^6$ flips below the initial ground state. At $r_c = 1.8a_0$ the state [Kr]$4d^9 4f^8$ flips below the initial ground state. At $r_c = 1.8a_0$ the state [Ar]$4s^2 3d^{10} 4p^5 4d^{10} 4f^8$ flips below the initial ground state. At $r_c = 1.7a_0$ the state [Kr]$4d^8 4f^9$ flips below the initial ground state. The ionization energy of the unconfined atom is $\Delta E_0 = 11.17$ eV. The studied configurations, atomic radii and excitation energies in the unconfined calculations are as follows:

configuration	r_ϵ	r_ρ	$r_{\max}$	ΔE
[Kr]$5s^2 4d^{10} 5p^5$	5.23	4.67	2.05	0.00
[Kr]$5s^2 4d^{10} 5p^4 6s^1$	9.82	4.87	5.46	7.70
[Kr]$5s^1 4d^{10} 5p^6$	5.24	4.67	2.04	8.94
[Kr]$5s^2 4d^{10} 5p^4 5d^1$	12.77	4.67	3.94	9.00
[Kr]$5s^2 4d^{10} 5p^4 4f^1$	24.61	4.35	16.52	10.26
[Kr]$5s^2 4d^{10} 5p^3 4f^2$	18.07	4.24	9.12	25.70
[Kr]$5s^2 4d^{10} 5p^2 4f^3$	15.74	5.12	5.86	43.71
[Kr]$5s^2 4d^{10} 5p^1 4f^4$	14.45	5.85	3.17	62.44
[Kr]$5s^2 4d^{10} 4f^5$	13.46	6.16	2.66	81.45
[Kr]$5s^1 4d^{10} 4f^6$	12.70	6.16	2.38	110.88
[Kr]$4d^{10} 5p^1 4f^6$	12.79	6.16	2.29	121.16
[Kr]$4d^{10} 4f^7$	12.03	6.16	2.24	140.48
[Kr]$4d^9 4f^8$	11.94	6.06	1.46	210.69
[Kr]$4d^8 4f^9$	11.91	5.91	1.20	282.89
[Ar]$4s^2 3d^{10} 4p^5 4d^{10} 4f^8$	12.00	6.06	1.40	283.04

Xe The energies of the low lying configurations of hard-wall confined Xe are shown in fig. S113 for the neutral atom and in fig. S114 for the cation. The ground state of the unconfined Xe is [Kr]$5s^2 4d^{10} 5p^6$. At $r_c = 2.0a_0$ the ground state changes to [Kr]$5s^2 4d^{10} 4f^6$. Furthermore, at $r_c = 1.7a_0$ we see a ground state crossing to [Kr]$4d^{10} 4f^8$.

At $r_c = 2.0a_0$ the state [Kr]$5s^1 4d^{10} 4f^7$ flips below the initial ground state. At $r_c = 2.0a_0$ the state [Kr]$5s^2 4d^{10} 5p^1 4f^5$ flips below the initial ground state. At $r_c = 2.0a_0$ the state [Kr]$5s^2 4d^{10} 5p^2 4f^4$ flips below the initial ground state. At $r_c = 2.0a_0$ the state [Kr]$5s^2 4d^{10} 5p^5 4f^1$ flips below the initial ground state. At $r_c = 2.0a_0$ the state [Kr]$5s^2 4d^{10} 5p^4 4f^2$ flips below the initial ground state. At $r_c = 1.9a_0$ the state [Kr]$4d^{10} 5p^1 4f^7$ flips below the initial ground state. At $r_c = 1.8a_0$ the state [Kr]$4d^9 4f^9$ flips below the initial ground state. At $r_c = 1.7a_0$ the state [Ar]$4s^2 3d^{10} 4p^5 4d^{10} 4f^9$ flips below the initial ground state. The ionization energy of the unconfined atom is $\Delta E_0 = 12.57$ eV. The studied configurations, atomic radii and excitation energies in the unconfined calculations are as follows:

configuration	r_ϵ	r_ρ	$r_{\max}$	ΔE
[Kr]$5s^2 4d^{10} 5p^6$	4.98	4.57	1.93	0.00
[Kr]$5s^2 4d^{10} 5p^5 6s^1$	9.53	4.87	5.22	8.97
[Kr]$5s^2 4d^{10} 5p^5 5d^1$	12.52	4.67	3.49	10.32
[Kr]$5s^2 4d^{10} 5p^5 4f^1$	24.64	4.24	16.56	11.65
[Kr]$5s^2 4d^{10} 5p^4 4f^2$	18.30	4.24	9.21	28.82
[Kr]$5s^2 4d^{10} 5p^2 4f^4$	15.21	5.46	1.86	67.46
[Kr]$5s^2 4d^{10} 5p^1 4f^5$	14.20	5.76	1.85	87.20
[Kr]$5s^2 4d^{10} 4f^6$	13.30	5.98	1.66	107.07
[Kr]$5s^1 4d^{10} 4f^7$	12.60	6.06	1.66	137.92
[Kr]$4d^{10} 5p^1 4f^7$	12.70	5.98	1.85	148.77
[Kr]$4d^{10} 4f^8$	11.94	6.06	1.44	168.87
[Kr]$4d^9 4f^9$	11.94	5.91	1.15	245.03
[Ar]$4s^2 3d^{10} 4p^5 4d^{10} 4f^9$	12.00	5.91	1.13	320.91

3.3 r²SCAN

H The energies of the low lying configurations of hard-wall confined H are shown in fig. S8 for the neutral atom. The ground state of the unconfined H is $1s^1$. We do not observe any ground state crossing for H in the considered confinement radii.

The ionization energy of the unconfined atom is $\Delta E_0 = 12.32$ eV. The studied configurations, atomic radii and excitation energies in the unconfined calculations are as follows:

configuration	r_ϵ	r_ρ	$r_{\max}$	ΔE
$1s^1$	3.19	3.06	1.04	0.00
$2p^1$	9.41	3.66	4.50	9.32
$3d^1$	17.57	undefined	10.07	10.92

He The energies of the low lying configurations of hard-wall confined He are shown in fig. S9 for the neutral atom and in fig. S10 for the cation. The ground state of the unconfined He is $1s^2$. We do not observe any ground state crossing for He in the considered confinement radii.

The ionization energy of the unconfined atom is $\Delta E_0 = 27.63$ eV. The studied configurations, atomic radii and excitation energies in the unconfined calculations are as follows:

configuration	r_ϵ	r_ρ	$r_{\max}$	ΔE
$1s^2$	2.22	2.55	0.56	0.00
$1s^1 2p^1$	9.02	3.89	4.15	24.49
$1s^1 3d^1$	17.57	2.06	10.08	26.22
$2p^2$	6.58	4.67	2.44	60.76

Li The energies of the low lying configurations of hard-wall confined Li are shown in fig. S11 for the neutral atom and in fig. S12 for the cation. The ground state of the unconfined Li is [He]$2s^1$. At $r_c = 2.9a_0$ the ground state changes to [He]$2p^1$.

The ionization energy of the unconfined atom is $\Delta E_0 = 5.01$ eV. The studied configurations, atomic radii and excitation energies in the unconfined calculations are as follows:

configuration	r_ϵ	r_ρ	$r_{\max}$	ΔE
[He]$2s^1$	6.69	4.28	3.21	0.00
[He]$2p^1$	9.11	3.78	4.23	1.91
[He]$3d^1$	17.58	1.86	10.10	3.60

Be The energies of the low lying configurations of hard-wall confined Be are shown in fig. S13 for the neutral atom and in fig. S14 for the cation. The ground state of the unconfined Be is [He]$2s^2$. At $r_c = 2.2a_0$ the ground state changes to [He]$2p^2$.

At $r_c = 2.2a_0$ the state [He]$2s^1 2p^1$ flips below the initial ground state. At $r_c = 1.1a_0$ the state [He]$2p^1 3d^1$ flips below the initial ground state. The ionization energy of the unconfined atom is $\Delta E_0 = 9.61$ eV. The studied configurations, atomic radii and excitation energies in the unconfined calculations are as follows:

configuration	r_ϵ	r_ρ	$r_{\max}$	ΔE
[He]$2s^2$	5.04	4.16	2.05	0.00
[He]$2s^12p^1$	5.93	4.46	2.31	4.00
[He]$2p^2$	6.41	4.57	2.30	7.96
[He]$2p^13d^1$	15.97	3.89	8.45	12.37

B The energies of the low lying configurations of hard-wall confined B are shown in fig. S15 for the neutral atom and in fig. S16 for the cation. The ground state of the unconfined B is [He]$2s^22p^1$. At $r_c = 1.7a_0$ the ground state changes to [He]$2p^3$.

At $r_c = 1.7a_0$ the state [He]$2s^12p^2$ flips below the initial ground state. The ionization energy of the unconfined atom is $\Delta E_0 = 7.94$ eV. The studied configurations, atomic radii and excitation energies in the unconfined calculations are as follows:

configuration	r_ϵ	r_ρ	$r_{\max}$	ΔE
[He]$2s^22p^1$	4.61	3.96	1.61	0.00
[He]$2s^23s^1$	10.32	3.61	5.85	4.86
[He]$2s^12p^2$	4.94	4.12	1.60	6.43
[He]$2p^3$	5.14	4.24	1.59	12.81

C The energies of the low lying configurations of hard-wall confined C are shown in fig. S17 for the neutral atom and in fig. S18 for the cation. The ground state of the unconfined C is [He]$2s^22p^2$. At $r_c = 1.4a_0$ the ground state changes to [He]$2p^4$.

At $r_c = 1.4a_0$ the state [He]$2s^12p^3$ flips below the initial ground state. The ionization energy of the unconfined atom is $\Delta E_0 = 10.74$ eV. The studied configurations, atomic radii and excitation energies in the unconfined calculations are as follows:

configuration	r_ϵ	r_ρ	$r_{\max}$	ΔE
[He]$2s^22p^2$	4.08	3.78	1.22	0.00
[He]$2s^22p^13s^1$	9.29	4.07	5.09	7.27
[He]$2s^12p^3$	4.24	3.89	1.22	9.22
[He]$2p^4$	4.36	4.01	1.21	18.40

N The energies of the low lying configurations of hard-wall confined N are shown in fig. S19 for the neutral atom and in fig. S20 for the cation. The ground state of the unconfined N is [He]$2s^22p^3$. At $r_c = 1.2a_0$ the ground state changes to [He]$2p^5$.

At $r_c = 1.2a_0$ the state [He]$2s^12p^4$ flips below the initial ground state. The ionization energy of the unconfined atom is $\Delta E_0 = 13.66$ eV. The studied configurations, atomic radii and excitation energies in the unconfined calculations are as follows:

configuration	r_ϵ	r_ρ	$r_{\max}$	ΔE
[He]$2s^22p^3$	3.64	3.49	1.01	0.00
[He]$2s^22p^23s^1$	8.58	4.28	4.56	9.85
[He]$2s^12p^4$	3.74	3.59	1.01	12.40
[He]$2p^5$	3.82	3.66	0.98	24.75

O The energies of the low lying configurations of hard-wall confined O are shown in fig. S21 for the neutral atom and in fig. S22 for the cation. The ground state of the unconfined O is [He]$2s^22p^4$. At $r_c = 1.1a_0$ the ground state changes to [He]$2p^6$.

At $r_c = 1.1a_0$ the state [He]$2s^12p^5$ flips below the initial ground state. The ionization energy of the unconfined atom is $\Delta E_0 = 16.70$ eV. The studied configurations, atomic radii and excitation energies in the unconfined calculations are as follows:

configuration	r_ϵ	r_ρ	$r_{\max}$	ΔE
[He]$2s^22p^4$	3.29	3.26	0.87	0.00
[He]$2s^22p^33s^1$	8.06	4.39	4.18	12.60
[He]$2s^12p^5$	3.36	3.33	0.87	15.96
[He]$2p^6$	3.42	3.45	0.83	31.88

F The energies of the low lying configurations of hard-wall confined F are shown in fig. S23 for the neutral atom and in fig. S24 for the cation. The ground state of the unconfined F is [He]$2s^22p^5$. We do not observe any ground state crossing for F in the considered confinement radii.

The ionization energy of the unconfined atom is $\Delta E_0 = 19.87$ eV. The studied configurations, atomic radii and excitation energies in the unconfined calculations are as follows:

configuration	r_ϵ	r_ρ	$r_{\max}$	ΔE
[He]$2s^22p^5$	3.00	3.12	0.76	0.00
[He]$2s^22p^43s^1$	7.64	4.39	3.88	15.52
[He]$2s^22p^43d^1$	17.37	2.83	9.89	18.45
[He]$2s^12p^6$	3.06	3.14	0.77	19.91
[He]$2p^63d^1$	17.32	2.90	9.84	58.93

Ne The energies of the low lying configurations of hard-wall confined Ne are shown in fig. S25 for the neutral atom and in fig. S26 for the cation. The ground state of the unconfined Ne is [He]$2s^22p^6$. We do not observe any ground state crossing for Ne in the considered confinement radii.

The ionization energy of the unconfined atom is $\Delta E_0 = 23.18$ eV. The studied configurations, atomic radii and excitation energies in the unconfined calculations are as follows:

configuration	r_ϵ	r_ρ	$r_{\max}$	ΔE
[He]$2s^22p^6$	2.77	3.03	0.68	0.00
[He]$2s^22p^53s^1$	7.30	4.39	3.64	18.59
[He]$2s^22p^53d^1$	17.41	2.70	9.93	21.76
[He]$2s^12p^63d^1$	17.39	2.69	9.91	46.39

Na The energies of the low lying configurations of hard-wall confined Na are shown in fig. S27 for the neutral atom and in fig. S28 for the cation. The ground state of the unconfined Na is [Ne]$3s^1$. At $r_c = 2.0a_0$ the ground state changes to [Ne]$3d^1$.

At $r_c = 1.5a_0$ the state [Ne]$3p^1$ flips below the initial ground state. At $r_c = 1.1a_0$ the state [Ne]$4f^1$ flips below the initial ground state. The ionization energy of the unconfined atom is $\Delta E_0 = 4.80$ eV. The studied configurations, atomic radii and excitation energies in the unconfined calculations are as follows:

configuration	r_ϵ	r_ρ	$r_{\max}$	ΔE
[Ne]$3s^1$	7.02	4.31	3.44	0.00
[Ne]$3p^1$	10.19	3.03	5.17	2.03
[Ne]$3d^1$	17.44	2.58	9.96	3.39
[Ne]$4f^1$	26.63	2.53	17.61	3.97

Mg The energies of the low lying configurations of hard-wall confined Mg are shown in fig. S29 for the neutral atom and in fig. S30 for the cation. The ground state of the unconfined Mg is [Ne]$3s^2$. At $r_c = 1.8a_0$ the ground state changes to [Ne]$3d^2$.

At $r_c = 1.8a_0$ the state [Ne]$3s^13d^1$ flips below the initial ground state. At $r_c = 1.7a_0$ the state [Ne]$3p^13d^1$ flips below the initial ground state. At $r_c = 1.4a_0$ the state [Ne]$3s^13p^1$ flips below the initial ground state. At $r_c = 1.4a_0$ the state [Ne]$3p^2$ flips below the initial ground state. At $r_c = 1.3a_0$ the state [Ne]$3d^14f^1$ flips below the initial ground state. The ionization energy of the unconfined atom is $\Delta E_0 = 7.99$ eV. The studied configurations, atomic radii and excitation energies in the unconfined calculations are as follows:

configuration	r_ϵ	r_ρ	$r_{\max}$	ΔE
[Ne]$3s^2$	5.88	4.64	2.58	0.00
[Ne]$3s^13p^1$	7.30	4.90	3.36	3.67
[Ne]$3s^13d^1$	15.97	4.06	8.43	6.46
[Ne]$3p^2$	7.72	5.22	3.34	7.38
[Ne]$3p^13d^1$	13.95	4.72	6.50	10.55
[Ne]$3d^2$	12.49	5.00	5.44	14.14
[Ne]$3d^14f^1$	22.98	3.59	13.35	15.65

Al The energies of the low lying configurations of hard-wall confined Al are shown in fig. S31 for the neutral atom and in fig. S32 for the cation. The ground state of the unconfined Al is [Ne]$3s^23p^1$. At $r_c = 1.8a_0$ the ground state changes to [Ne]$3s^23d^1$. Furthermore, at $r_c = 1.6a_0$ we see a ground state crossing to [Ne]$3d^3$.

At $r_c = 1.7a_0$ the state [Ne]$3s^13d^2$ flips below the initial ground state. At $r_c = 1.6a_0$ the state [Ne]$3p^13d^2$ flips below the initial ground state. At $r_c = 1.3a_0$ the state [Ne]$3s^13p^2$ flips below the initial ground state. The ionization energy of the unconfined atom is $\Delta E_0 = 5.65$ eV. The studied configurations, atomic radii and excitation energies in the unconfined calculations are as follows:

configuration	r_ϵ	r_ρ	$r_{\max}$	ΔE
[Ne]$3s^23p^1$	6.01	4.64	2.63	0.00
[Ne]$3s^24s^1$	11.81	4.03	7.00	2.99
[Ne]$3s^23d^1$	15.89	4.09	8.30	4.10
[Ne]$3s^13p^2$	6.34	4.80	2.59	5.34
[Ne]$3s^13d^2$	11.66	5.22	4.47	15.72
[Ne]$3p^13d^2$	11.10	5.47	4.10	21.67
[Ne]$3d^3$	10.19	5.89	3.70	27.89

Si The energies of the low lying configurations of hard-wall confined Si are shown in fig. S33 for the neutral atom and in fig. S34 for the cation. The ground state of the unconfined Si is [Ne]$3s^23p^2$. At $r_c = 1.7a_0$ the ground state changes to [Ne]$3s^23d^2$. Furthermore, at $r_c = 1.5a_0$ we see a ground state crossing to [Ne]$3d^4$.

At $r_c = 1.7a_0$ the state [Ne]$3s^2 3p^1 3d^1$ flips below the initial ground state. At $r_c = 1.6a_0$ the state [Ne]$3s^1 3d^3$ flips below the initial ground state. At $r_c = 1.5a_0$ the state [Ne]$3p^1 3d^3$ flips below the initial ground state. At $r_c = 1.2a_0$ the state [Ne]$3s^1 3p^3$ flips below the initial ground state. The ionization energy of the unconfined atom is $\Delta E_0 = 7.73$ eV. The studied configurations, atomic radii and excitation energies in the unconfined calculations are as follows:

configuration	r_ϵ	r_ρ	$r_{\max}$	ΔE
[Ne]$3s^2 3p^2$	5.47	4.54	2.16	0.00
[Ne]$3s^2 3p^1 4s^1$	10.68	4.28	6.17	4.75
[Ne]$3s^2 3p^1 3d^1$	15.20	4.23	7.42	6.07
[Ne]$3s^1 3p^3$	5.59	4.61	2.13	7.07
[Ne]$3s^2 3d^2$	11.44	5.00	3.98	13.93
[Ne]$3s^1 3d^3$	9.68	5.50	3.07	30.39
[Ne]$3p^1 3d^3$	9.40	5.44	2.97	38.20
[Ne]$3d^4$	8.79	5.64	2.79	47.17

P The energies of the low lying configurations of hard-wall confined P are shown in fig. S35 for the neutral atom and in fig. S36 for the cation. The ground state of the unconfined P is [Ne]$3s^2 3p^3$. At $r_c = 1.5a_0$ the ground state changes to [Ne]$3s^2 3d^3$. Furthermore, at $r_c = 1.4a_0$ we see a ground state crossing to [Ne]$3d^5$.

At $r_c = 1.5a_0$ the state [Ne]$3s^1 3d^4$ flips below the initial ground state. At $r_c = 1.5a_0$ the state [Ne]$3s^2 3p^2 3d^1$ flips below the initial ground state. At $r_c = 1.5a_0$ the state [Ne]$3s^2 3p^1 3d^2$ flips below the initial ground state. At $r_c = 1.4a_0$ the state [Ne]$3p^1 3d^4$ flips below the initial ground state. At $r_c = 1.1a_0$ the state [Ne]$3s^1 3p^4$ flips below the initial ground state. The ionization energy of the unconfined atom is $\Delta E_0 = 9.82$ eV. The studied configurations, atomic radii and excitation energies in the unconfined calculations are as follows:

configuration	r_ϵ	r_ρ	$r_{\max}$	ΔE
[Ne]$3s^2 3p^3$	4.95	4.31	1.84	0.00
[Ne]$3s^2 3p^2 4s^1$	9.95	4.38	5.60	6.59
[Ne]$3s^2 3p^2 3d^1$	15.13	4.15	7.23	8.13
[Ne]$3s^1 3p^4$	5.01	4.39	1.82	8.85
[Ne]$3s^2 3p^1 3d^2$	11.03	4.89	3.18	18.47
[Ne]$3s^2 3d^3$	9.47	5.28	2.63	29.62
[Ne]$3s^1 3d^4$	8.44	5.34	2.36	50.93
[Ne]$3p^1 3d^4$	8.26	5.31	2.33	60.59
[Ne]$3d^5$	7.82	5.34	2.23	72.44

S The energies of the low lying configurations of hard-wall confined S are shown in fig. S37 for the neutral atom and in fig. S38 for the cation. The ground state of the unconfined S is [Ne]$3s^2 3p^4$. At $r_c = 1.4a_0$ the ground state changes to [Ne]$3s^2 3d^4$. Furthermore, at $r_c = 1.3a_0$ we see a ground state crossing to [Ne]$3d^6$.

At $r_c = 1.4a_0$ the state [Ne]$3s^1 3d^5$ flips below the initial ground state. At $r_c = 1.4a_0$ the state [Ne]$3s^2 3p^1 3d^3$ flips below the initial ground state. At $r_c = 1.4a_0$ the state [Ne]$3s^2 3p^2 3d^2$ flips below the initial ground state. At $r_c = 1.4a_0$ the state [Ne]$3s^2 3p^3 3d^1$ flips below the initial ground state. At $r_c = 1.3a_0$ the state [Ne]$3p^1 3d^5$ flips below the initial ground state. At $r_c = 1.1a_0$ the state [Ne]$3s^1 3p^5$ flips below the initial ground state. The ionization energy of the unconfined atom is $\Delta E_0 = 11.97$ eV. The studied configurations, atomic radii and excitation energies in the unconfined calculations are as follows:

configuration	r_ϵ	r_ρ	$r_{\max}$	ΔE
[Ne]$3s^2 3p^4$	4.53	4.13	1.61	0.00
[Ne]$3s^2 3p^3 4s^1$	9.41	4.45	5.18	8.52
[Ne]$3s^2 3p^3 3d^1$	15.30	3.99	7.41	10.28
[Ne]$3s^1 3p^5$	4.56	4.20	1.60	10.67
[Ne]$3s^2 3p^2 3d^2$	10.89	4.79	2.47	23.27
[Ne]$3s^2 3p^1 3d^3$	9.18	5.08	2.19	37.13
[Ne]$3s^2 3d^4$	8.26	5.15	2.05	51.50
[Ne]$3s^1 3d^5$	7.56	5.13	1.94	77.77
[Ne]$3p^1 3d^5$	7.43	5.10	1.92	89.31
[Ne]$3d^6$	7.10	5.10	1.86	104.16

Cl The energies of the low lying configurations of hard-wall confined Cl are shown in fig. S39 for the neutral atom and in fig. S40

for the cation. The ground state of the unconfined Cl is [Ne]$3s^2 3p^5$. At $r_c = 1.3a_0$ the ground state changes to [Ne]$3s^1 3d^6$. Furthermore, at $r_c = 1.2a_0$ we see a ground state crossing to [Ne]$3d^7$.

At $r_c = 1.3a_0$ the state [Ne]$3p^1 3d^6$ flips below the initial ground state. At $r_c = 1.3a_0$ the state [Ne]$3s^2 3p^4 3d^1$ flips below the initial ground state. At $r_c = 1.3a_0$ the state [Ne]$3s^2 3p^3 3d^2$ flips below the initial ground state. At $r_c = 1.0a_0$ the state [Ne]$3s^1 3p^6$ flips below the initial ground state. The ionization energy of the unconfined atom is $\Delta E_0 = 14.20$ eV. The studied configurations, atomic radii and excitation energies in the unconfined calculations are as follows:

configuration	r_ϵ	r_ρ	$r_{\max}$	ΔE
[Ne]$3s^2 3p^5$	4.17	3.93	1.43	0.00
[Ne]$3s^2 3p^4 4s^1$	8.98	4.45	4.85	10.57
[Ne]$3s^2 3p^4 3d^1$	15.54	3.81	7.74	12.53
[Ne]$3s^1 3p^6$	4.19	3.99	1.42	12.55
[Ne]$3s^2 3p^3 3d^2$	10.92	4.63	2.07	28.32
[Ne]$3s^1 3d^6$	6.90	4.97	1.65	111.37
[Ne]$3p^1 3d^6$	6.80	4.93	1.63	124.82
[Ne]$3d^7$	6.54	4.93	1.59	142.82

Ar The energies of the low lying configurations of hard-wall confined Ar are shown in fig. S41 for the neutral atom and in fig. S42 for the cation. The ground state of the unconfined Ar is [Ne]$3s^2 3p^6$. At $r_c = 1.2a_0$ the ground state changes to [Ne]$3s^2 3d^6$. Furthermore, at $r_c = 1.1a_0$ we see a ground state crossing to [Ne]$3d^8$.

At $r_c = 1.2a_0$ the state [Ne]$3p^1 3d^7$ flips below the initial ground state. At $r_c = 1.2a_0$ the state [Ne]$3s^1 3d^7$ flips below the initial ground state. At $r_c = 1.2a_0$ the state [Ne]$3s^2 3p^1 3d^5$ flips below the initial ground state. At $r_c = 1.2a_0$ the state [Ne]$3s^2 3p^5 3d^1$ flips below the initial ground state. At $r_c = 1.2a_0$ the state [Ne]$3s^2 3p^4 3d^2$ flips below the initial ground state. At $r_c = 1.1a_0$ the state [Ne]$3s^1 3p^6 3d^1$ flips below the initial ground state. The ionization energy of the unconfined atom is $\Delta E_0 = 16.52$ eV. The studied configurations, atomic radii and excitation energies in the unconfined calculations are as follows:

configuration	r_ϵ	r_ρ	$r_{\max}$	ΔE
[Ne]$3s^2 3p^6$	3.87	3.76	1.29	0.00
[Ne]$3s^2 3p^5 4s^1$	8.63	4.45	4.59	12.71
[Ne]$3s^2 3p^5 3d^1$	15.80	3.66	8.09	14.88
[Ne]$3s^1 3p^6 3d^1$	15.63	3.71	7.83	29.89
[Ne]$3s^2 3p^4 3d^2$	11.04	4.54	1.79	33.63
[Ne]$3s^2 3p^1 3d^5$	7.24	4.89	1.51	94.49
[Ne]$3s^2 3d^6$	6.78	4.87	1.47	115.52
[Ne]$3s^1 3d^7$	6.38	4.87	1.43	152.20
[Ne]$3p^1 3d^7$	6.30	4.77	1.42	167.60
[Ne]$3d^8$	6.09	4.73	1.39	188.89

K The energies of the low lying configurations of hard-wall confined K are shown in fig. S43 for the neutral atom and in fig. S44 for the cation. The ground state of the unconfined K is [Ar]$4s^1$. At $r_c = 4.6a_0$ the ground state changes to [Ar]$3d^1$. Furthermore, at $r_c = 1.1a_0$ we see a ground state crossing to [Ne]$3s^1 3d^8$. At $r_c = 1.0a_0$ we see a ground state crossing to [Ne]$3d^9$.

At $r_c = 2.8a_0$ the state [Ne]$3s^2 3p^5 3d^2$ flips below the initial ground state. At $r_c = 2.4a_0$ the state [Ne]$3s^1 3p^6 3d^2$ flips below the initial ground state. At $r_c = 2.3a_0$ the state [Ne]$3s^2 3p^4 3d^3$ flips below the initial ground state. At $r_c = 1.5a_0$ the state [Ne]$3p^1 3d^8$ flips below the initial ground state. At $r_c = 1.4a_0$ the state [Ar]$4p^1$ flips below the initial ground state. The ionization energy of the unconfined atom is $\Delta E_0 = 3.97$ eV. The studied configurations, atomic radii and excitation energies in the unconfined calculations are as follows:

configuration	r_ϵ	r_ρ	$r_{\max}$	ΔE
[Ar]$4s^1$	8.33	4.38	4.38	0.00
[Ar]$4p^1$	11.31	3.55	6.13	1.47
[Ar]$3d^1$	16.05	3.49	8.41	2.36
[Ne]$3s^2 3p^5 3d^2$	11.23	4.35	1.58	24.24
[Ne]$3s^1 3p^6 3d^2$	11.13	4.32	1.55	41.48
[Ne]$3s^2 3p^4 3d^3$	9.11	4.74	1.46	47.19
[Ne]$3s^1 3d^8$	5.96	4.67	1.27	185.78
[Ne]$3p^1 3d^8$	5.90	4.67	1.26	203.19
[Ne]$3d^9$	5.71	4.67	1.24	227.93

Ca The energies of the low lying configurations of hard-wall confined Ca are shown in fig. S45 for the neutral atom and in fig. S46 for the cation. The ground state of the un-

confined Ca is [Ar]$4s^2$. At $r_c = 4.6a_0$ the ground state changes to [Ar]$4s^13d^1$. Furthermore, at $r_c = 4.5a_0$ we see a ground state crossing to [Ar]$3d^2$. At $r_c = 1.1a_0$ we see a ground state crossing to [Ne]$3s^23p^43d^4$. Moreover, at $r_c = 1.0a_0$ we see a ground state crossing to [Ne]$3d^{10}$.

At $r_c = 3.4a_0$ the state [Ar]$3d^14p^1$ flips below the initial ground state. At $r_c = 3.1a_0$ the state [Ne]$3s^23p^53d^3$ flips below the initial ground state. At $r_c = 2.8a_0$ the state [Ne]$3s^13p^63d^3$ flips below the initial ground state. At $r_c = 1.8a_0$ the state [Ne]$3s^13d^9$ flips below the initial ground state. At $r_c = 1.7a_0$ the state [Ne]$3p^13d^9$ flips below the initial ground state. At $r_c = 1.3a_0$ the state [Ar]$4s^14p^1$ flips below the initial ground state. The ionization energy of the unconfined atom is $\Delta E_0 = 6.28$ eV. The studied configurations, atomic radii and excitation energies in the unconfined calculations are as follows:

configuration	r_ϵ	r_ρ	$r_{\max}$	ΔE
[Ar]$4s^2$	7.22	5.25	3.46	0.00
[Ar]$4s^14p^1$	8.50	5.36	4.32	2.49
[Ar]$4s^13d^1$	8.43	4.98	3.68	2.50
[Ar]$3d^14p^1$	9.82	4.74	4.92	4.53
[Ar]$3d^2$	11.45	4.24	1.40	5.06
[Ne]$3s^23p^53d^3$	9.25	4.61	1.31	31.37
[Ne]$3s^13p^63d^3$	9.15	4.55	1.29	50.80
[Ne]$3s^23p^43d^4$	7.93	4.67	1.26	58.32
[Ne]$3s^13d^9$	5.61	4.54	1.14	217.53
[Ne]$3p^13d^9$	5.56	4.57	1.13	237.01
[Ne]$3d^{10}$	5.40	4.46	1.12	265.36

Sc The energies of the low lying configurations of hard-wall confined Sc are shown in fig. S47 for the neutral atom and in fig. S48 for the cation. The ground state of the unconfined Sc is [Ar]$4s^23d^1$. At $r_c = 4.6a_0$ the ground state changes to [Ar]$4s^13d^2$. Furthermore, at $r_c = 4.4a_0$ we see a ground state crossing to [Ar]$3d^3$. At $r_c = 1.0a_0$ we see a ground state crossing to [Ne]$3s^23d^9$.

At $r_c = 3.3a_0$ the state [Ar]$3d^24p^1$ flips below the initial ground state. At $r_c = 3.0a_0$ the state [Ne]$3s^23p^53d^4$ flips below the initial ground state. At $r_c = 2.6a_0$ the state [Ne]$3s^13p^63d^4$ flips below the initial ground state. At $r_c = 2.5a_0$ the state [Ne]$3s^23p^43d^5$ flips below the initial ground state. At $r_c = 1.9a_0$ the state [Ne]$3s^23p^13d^8$ flips below the initial ground state. At $r_c = 1.7a_0$ the state [Ne]$3s^13d^{10}$ flips below the initial ground state. At $r_c = 1.3a_0$ the state [Ar]$4s^13d^14p^1$ flips below the initial ground state. The ionization energy of the unconfined atom is $\Delta E_0 = 6.91$ eV. The studied configurations, atomic radii and excitation energies in the unconfined calculations are as follows:

configuration	r_ϵ	r_ρ	$r_{\max}$	ΔE
[Ar]$4s^23d^1$	6.76	5.05	3.17	0.00
[Ar]$4s^13d^2$	7.29	4.86	3.37	1.84
[Ar]$4s^13d^14p^1$	8.01	5.24	3.97	2.88
[Ar]$3d^24p^1$	9.18	4.84	4.53	4.18
[Ar]$3d^3$	9.44	4.54	1.18	4.20
[Ne]$3s^23p^53d^4$	8.04	4.67	1.14	34.69
[Ne]$3s^13p^63d^4$	7.94	4.57	1.13	56.33
[Ne]$3s^23p^43d^5$	7.12	4.67	1.11	65.67
[Ne]$3s^23p^13d^8$	5.77	4.45	1.06	160.46
[Ne]$3s^23d^9$	5.53	4.46	1.04	192.45
[Ne]$3s^13d^{10}$	5.31	4.46	1.03	246.22

Ti The energies of the low lying configurations of hard-wall confined Ti are shown in fig. S49 for the neutral atom and in fig. S50 for the cation. The ground state of the unconfined Ti is [Ar]$4s^23d^2$. At $r_c = 4.7a_0$ the ground state changes to [Ar]$4s^13d^3$. Furthermore, at $r_c = 4.3a_0$ we see a ground state crossing to [Ar]$3d^4$.

At $r_c = 3.2a_0$ the state [Ar]$3d^34p^1$ flips below the initial ground state. At $r_c = 2.8a_0$ the state [Ne]$3s^23p^53d^5$ flips below the initial ground state. At $r_c = 2.5a_0$ the state [Ne]$3s^13p^63d^5$ flips below the initial ground state. At $r_c = 2.4a_0$ the state [Ne]$3s^23p^43d^6$ flips below the initial ground state. At $r_c = 2.1a_0$ the state [Ne]$3s^23p^33d^7$ flips below the initial ground state. At $r_c = 1.2a_0$ the state [Ar]$4s^13d^24p^1$ flips below the initial ground state. The ionization energy of the unconfined atom is $\Delta E_0 = 7.19$ eV. The studied configurations, atomic radii and excitation energies in the unconfined calculations are as follows:

configuration	r_ϵ	r_ρ	$r_{\max}$	ΔE
[Ar]$4s^2 3d^2$	6.41	4.93	2.97	0.00
[Ar]$4s^1 3d^3$	6.72	4.79	3.15	1.22
[Ar]$4s^1 3d^2 4p^1$	7.70	5.10	3.73	3.19
[Ar]$3d^4$	8.19	4.54	1.04	3.31
[Ar]$3d^3 4p^1$	8.86	4.79	4.28	3.80
[Ne]$3s^2 3p^5 3d^5$	7.20	4.54	1.02	38.01
[Ne]$3s^1 3p^6 3d^5$	7.12	4.47	1.01	61.89
[Ne]$3s^2 3p^4 3d^6$	6.53	4.47	1.00	73.09
[Ne]$3s^2 3p^3 3d^7$	6.05	4.47	0.98	108.47

V The energies of the low lying configurations of hard-wall confined V are shown in fig. S51 for the neutral atom and in fig. S52 for the cation. The ground state of the unconfined V is [Ar]$4s^2 3d^3$. At $r_c = 5.0a_0$ the ground state changes to [Ar]$4s^1 3d^4$. Furthermore, at $r_c = 4.3a_0$ we see a ground state crossing to [Ar]$3d^5$.

At $r_c = 3.1a_0$ the state [Ar]$3d^4 4p^1$ flips below the initial ground state. At $r_c = 2.7a_0$ the state [Ne]$3s^2 3p^5 3d^6$ flips below the initial ground state. At $r_c = 2.4a_0$ the state [Ne]$3s^1 3p^6 3d^6$ flips below the initial ground state. At $r_c = 2.2a_0$ the state [Ne]$3s^2 3p^4 3d^7$ flips below the initial ground state. The ionization energy of the unconfined atom is $\Delta E_0 = 6.89$ eV. The studied configurations, atomic radii and excitation energies in the unconfined calculations are as follows:

configuration	r_ϵ	r_ρ	$r_{\max}$	ΔE
[Ar]$4s^2 3d^3$	6.14	4.80	2.81	0.00
[Ar]$4s^1 3d^4$	6.33	4.67	2.98	0.63
[Ar]$3d^5$	7.32	4.43	0.93	2.44
[Ar]$3d^4 4p^1$	8.66	4.71	4.10	3.42
[Ne]$3s^2 3p^5 3d^6$	6.58	4.38	0.92	41.42
[Ne]$3s^1 3p^6 3d^6$	6.51	4.38	0.92	67.60
[Ne]$3s^2 3p^4 3d^7$	6.06	4.37	0.91	80.72

Cr The energies of the low lying configurations of hard-wall confined Cr are shown in fig. S53 for the neutral atom and in fig. S54 for the cation. The ground state of the unconfined Cr is [Ar]$4s^2 3d^4$. At $r_c = 6.1a_0$ the ground state changes to [Ar]$4s^1 3d^5$. Furthermore, at $r_c = 4.4a_0$ we see a ground state crossing to [Ar]$3d^6$.

At $r_c = 3.0a_0$ the state [Ar]$3d^5 4p^1$ flips below the initial ground state. At $r_c = 2.6a_0$ the state [Ne]$3s^2 3p^5 3d^7$ flips below the initial ground state. At $r_c = 2.3a_0$ the state [Ne]$3s^1 3p^6 3d^7$ flips below the initial ground state. At $r_c = 2.1a_0$ the state [Ne]$3s^2 3p^4 3d^8$ flips below the initial ground state. The ionization energy of the unconfined atom is $\Delta E_0 = 6.58$ eV. The studied configurations, atomic radii and excitation energies in the unconfined calculations are as follows:

configuration	r_ϵ	r_ρ	$r_{\max}$	ΔE
[Ar]$4s^2 3d^4$	5.92	4.71	2.68	0.00
[Ar]$4s^1 3d^5$	6.05	4.59	2.84	0.06
[Ar]$3d^6$	6.67	4.38	0.85	1.59
[Ar]$3d^5 4p^1$	8.52	4.63	3.96	3.06
[Ne]$3s^2 3p^5 3d^7$	6.10	4.32	0.84	44.97
[Ne]$3s^1 3p^6 3d^7$	6.04	4.28	0.84	73.51
[Ne]$3s^2 3p^4 3d^8$	5.68	4.28	0.84	88.63

Mn The energies of the low lying configurations of hard-wall confined Mn are shown in fig. S55 for the neutral atom and in fig. S56 for the cation. The ground state of the unconfined Mn is [Ar]$4s^1 3d^6$. At $r_c = 4.4a_0$ the ground state changes to [Ar]$3d^7$.

At $r_c = 2.0a_0$ the state [Ne]$3s^2 3p^5 3d^8$ flips below the initial ground state. At $r_c = 1.8a_0$ the state [Ne]$3s^1 3p^6 3d^8$ flips below the initial ground state. At $r_c = 1.1a_0$ the state [Ar]$3d^6 4p^1$ flips below the initial ground state. The ionization energy of the unconfined atom is $\Delta E_0 = 6.75$ eV. The studied configurations, atomic radii and excitation energies in the unconfined calculations are as follows:

configuration	r_ϵ	r_ρ	$r_{\max}$	ΔE
[Ar]$4s^1 3d^6$	5.81	4.54	2.72	0.00
[Ar]$4s^2 3d^5$	5.73	4.63	2.56	0.48
[Ar]$3d^7$	6.17	4.28	0.79	1.25
[Ar]$3d^6 4p^1$	8.42	4.63	3.84	3.19
[Ne]$3s^2 3p^5 3d^8$	5.71	4.20	0.78	49.17
[Ne]$3s^1 3p^6 3d^8$	5.66	4.20	0.78	80.12

Fe The energies of the low lying configurations of hard-wall confined Fe are shown in fig. S57 for the neutral atom and in fig. S58 for the cation. The ground state of the unconfined Fe is [Ar]$4s^1 3d^7$. At $r_c = 4.6a_0$ the ground state changes to [Ar]$3d^8$.

At $r_c = 2.0a_0$ the state [Ne]$3s^2 3p^5 3d^9$ flips below the initial ground state. At $r_c = 1.7a_0$

the state [Ne]$3s^1 3p^6 3d^9$ flips below the initial ground state. At $r_c = 1.1a_0$ the state [Ar]$3d^7 4p^1$ flips below the initial ground state. The ionization energy of the unconfined atom is $\Delta E_0 =$ 6.97 eV. The studied configurations, atomic radii and excitation energies in the unconfined calculations are as follows:

configuration	r_ϵ	r_ρ	$r_{\max}$	ΔE
[Ar]$4s^1 3d^7$	5.62	4.45	2.61	0.00
[Ar]$3d^8$	5.77	4.20	0.73	0.98
[Ar]$4s^2 3d^6$	5.56	4.54	2.46	1.01
[Ar]$3d^7 4p^1$	8.34	4.54	3.75	3.38
[Ne]$3s^2 3p^5 3d^9$	5.39	4.13	0.73	53.58
[Ne]$3s^1 3p^6 3d^9$	5.35	4.13	0.73	87.01

Co The energies of the low lying configurations of hard-wall confined Co are shown in fig. S59 for the neutral atom and in fig. S60 for the cation. The ground state of the unconfined Co is [Ar]$4s^1 3d^8$. At $r_c = 4.7a_0$ the ground state changes to [Ar]$3d^9$.

At $r_c = 1.9a_0$ the state [Ne]$3s^2 3p^5 3d^{10}$ flips below the initial ground state. At $r_c = 1.6a_0$ the state [Ne]$3s^1 3p^6 3d^{10}$ flips below the initial ground state. At $r_c = 1.0a_0$ the state [Ar]$3d^8 4p^1$ flips below the initial ground state. The ionization energy of the unconfined atom is $\Delta E_0 = 7.18$ eV. The studied configurations, atomic radii and excitation energies in the unconfined calculations are as follows:

configuration	r_ϵ	r_ρ	$r_{\max}$	ΔE
[Ar]$4s^1 3d^8$	5.45	4.40	2.51	0.00
[Ar]$3d^9$	5.44	4.07	0.69	0.71
[Ar]$4s^2 3d^7$	5.41	4.52	2.37	1.52
[Ar]$3d^8 4p^1$	8.28	4.52	3.67	3.56
[Ne]$3s^2 3p^5 3d^{10}$	5.12	4.07	0.68	58.15
[Ne]$3s^1 3p^6 3d^{10}$	5.09	4.07	0.68	94.13

Ni The energies of the low lying configurations of hard-wall confined Ni are shown in fig. S61 for the neutral atom and in fig. S62 for the cation. The ground state of the unconfined Ni is [Ar]$4s^1 3d^9$. At $r_c = 5.1a_0$ the ground state changes to [Ar]$3d^{10}$.

At $r_c = 1.4a_0$ the state [Ar]$3d^9 4f^1$ flips below the initial ground state. At $r_c = 1.2a_0$ the state [Ne]$3s^2 3p^5 3d^{10} 4f^1$ flips below the initial ground state. At $r_c = 1.0a_0$ the state [Ar]$3d^9 4p^1$ flips below the initial ground state. The ionization energy of the unconfined atom is $\Delta E_0 = 7.38$ eV. The studied configurations, atomic radii and excitation energies in the unconfined calculations are as follows:

configuration	r_ϵ	r_ρ	$r_{\max}$	ΔE
[Ar]$4s^1 3d^9$	5.30	4.29	2.43	0.00
[Ar]$3d^{10}$	5.16	4.07	0.65	0.45
[Ar]$4s^2 3d^8$	5.28	4.52	2.29	2.01
[Ar]$3d^9 4p^1$	8.23	4.40	3.60	3.74
[Ar]$3d^9 4f^1$	26.54	3.37	17.50	6.54
[Ne]$3s^2 3p^5 3d^{10} 4f^1$	26.56	3.33	17.51	69.60

Cu The energies of the low lying configurations of hard-wall confined Cu are shown in fig. S63 for the neutral atom and in fig. S64 for the cation. The ground state of the unconfined Cu is [Ar]$4s^1 3d^{10}$. At $r_c = 1.4a_0$ the ground state changes to [Ar]$3d^{10} 4f^1$.

At $r_c = 1.1a_0$ the state [Ar]$3d^{10} 4d^1$ flips below the initial ground state. At $r_c = 1.0a_0$ the state [Ar]$3d^{10} 4p^1$ flips below the initial ground state. The ionization energy of the unconfined atom is $\Delta E_0 = 7.57$ eV. The studied configurations, atomic radii and excitation energies in the unconfined calculations are as follows:

configuration	r_ϵ	r_ρ	$r_{\max}$	ΔE
[Ar]$4s^1 3d^{10}$	5.17	4.13	2.35	0.00
[Ar]$4s^2 3d^9$	5.16	4.29	2.22	2.49
[Ar]$3d^{10} 4p^1$	8.19	4.32	3.54	3.91
[Ar]$3d^{10} 4d^1$	17.18	3.37	9.69	6.13
[Ar]$3d^{10} 4f^1$	26.56	3.32	17.51	6.73

Zn The energies of the low lying configurations of hard-wall confined Zn are shown in fig. S65 for the neutral atom and in fig. S66 for the cation. The ground state of the unconfined Zn is [Ar]$4s^2 3d^{10}$. At $r_c = 1.3a_0$ the ground state changes to [Ar]$3d^{10} 4f^2$.

At $r_c = 1.3a_0$ the state [Ar]$4s^1 3d^{10} 4f^1$ flips below the initial ground state. At $r_c = 1.2a_0$ the state [Ar]$3d^{10} 4d^1 4f^1$ flips below the initial ground state. At $r_c = 1.2a_0$ the state [Ar]$3d^{10} 4p^1 4f^1$ flips below the initial ground state. At $r_c = 1.0a_0$ the state [Ar]$4s^1 3d^{10} 4d^1$ flips below the initial ground state. The ionization energy of the unconfined atom is $\Delta E_0 =$ 9.77 eV. The studied configurations, atomic radii and excitation energies in the unconfined calculations are as follows:

configuration	r_ϵ	r_ρ	$r_{\max}$	ΔE
$[Ar]4s^2 3d^{10}$	5.04	4.23	2.15	0.00
$[Ar]4s^1 3d^{10} 4p^1$	6.77	4.55	2.90	5.16
$[Ar]4s^1 3d^{10} 4d^1$	16.60	3.81	9.10	8.29
$[Ar]4s^1 3d^{10} 4f^1$	26.54	3.71	17.47	8.93
$[Ar]3d^{10} 4p^2$	7.07	4.89	2.86	10.40
$[Ar]3d^{10} 4p^1 4f^1$	26.18	4.07	17.07	15.01
$[Ar]3d^{10} 4d^1 4f^1$	22.59	3.66	12.93	20.69
$[Ar]3d^{10} 4f^2$	19.48	2.94	9.90	22.67

Ga The energies of the low lying configurations of hard-wall confined Ga are shown in fig. S67 for the neutral atom and in fig. S68 for the cation. The ground state of the unconfined Ga is [Ar]$4s^2 3d^{10} 4p^1$. At $r_c = 1.3a_0$ the ground state changes to [Ar]$4s^2 3d^{10} 4f^1$. Furthermore, at $r_c = 1.2a_0$ we see a ground state crossing to [Ar]$3d^{10} 4f^3$.

At $r_c = 1.3a_0$ the state [Ar]$4s^1 3d^{10} 4f^2$ flips below the initial ground state. At $r_c = 1.2a_0$ the state [Ar]$3d^{10} 4d^1 4f^2$ flips below the initial ground state. At $r_c = 1.2a_0$ the state [Ar]$3d^{10} 4p^1 4f^2$ flips below the initial ground state. At $r_c = 1.1a_0$ the state [Ar]$4s^2 3d^{10} 4d^1$ flips below the initial ground state. The ionization energy of the unconfined atom is $\Delta E_0 = 5.60$ eV. The studied configurations, atomic radii and excitation energies in the unconfined calculations are as follows:

configuration	r_ϵ	r_ρ	$r_{\max}$	ΔE
$[Ar]4s^2 3d^{10} 4p^1$	5.87	4.50	2.51	0.00
$[Ar]4s^2 3d^{10} 5s^1$	11.43	3.94	6.70	2.86
$[Ar]4s^2 3d^{10} 4d^1$	16.72	3.90	9.22	4.14
$[Ar]4s^2 3d^{10} 4f^1$	26.63	3.81	17.60	4.77
$[Ar]4s^1 3d^{10} 4p^2$	6.18	4.74	2.47	6.50
$[Ar]4s^1 3d^{10} 4f^2$	19.42	3.43	9.81	21.75
$[Ar]3d^{10} 4p^1 4f^2$	19.26	3.81	9.54	29.76
$[Ar]3d^{10} 4d^1 4f^2$	18.23	4.98	8.25	38.87
$[Ar]3d^{10} 4f^3$	16.11	4.82	6.81	42.92

Ge The energies of the low lying configurations of hard-wall confined Ge are shown in fig. S69 for the neutral atom and in fig. S70 for the cation. The ground state of the unconfined Ge is [Ar]$4s^2 3d^{10} 4p^2$. At $r_c = 1.3a_0$ the ground state changes to [Ar]$4s^2 3d^{10} 4f^2$. Furthermore, at $r_c = 1.2a_0$ we see a ground state crossing to [Ar]$3d^{10} 4f^4$.

At $r_c = 1.3a_0$ the state [Ar]$4s^1 3d^{10} 4f^3$ flips below the initial ground state. At $r_c = 1.3a_0$ the state [Ar]$4s^2 3d^{10} 4p^1 4f^1$ flips below the initial ground state. At $r_c = 1.2a_0$ the state [Ar]$3d^{10} 4d^1 4f^3$ flips below the initial ground state. At $r_c = 1.0a_0$ the state [Ar]$4s^2 3d^{10} 4p^1 4d^1$ flips below the initial ground state. The ionization energy of the unconfined atom is $\Delta E_0 = 7.52$ eV. The studied configurations, atomic radii and excitation energies in the unconfined calculations are as follows:

configuration	r_ϵ	r_ρ	$r_{\max}$	ΔE
$[Ar]4s^2 3d^{10} 4p^2$	5.56	4.51	2.21	0.00
$[Ar]4s^2 3d^{10} 4p^1 5s^1$	10.59	4.34	6.09	4.50
$[Ar]4s^2 3d^{10} 4p^1 4d^1$	15.81	4.13	8.23	5.95
$[Ar]4s^2 3d^{10} 4p^1 4f^1$	26.47	4.01	17.44	6.68
$[Ar]4s^1 3d^{10} 4p^3$	5.66	4.64	2.18	7.88
$[Ar]4s^2 3d^{10} 4f^2$	19.46	3.55	9.88	18.17
$[Ar]4s^1 3d^{10} 4f^3$	16.07	5.24	6.68	43.40
$[Ar]3d^{10} 4d^1 4f^3$	15.64	6.04	5.95	65.67
$[Ar]3d^{10} 4f^4$	14.16	6.72	5.14	72.21

As The energies of the low lying configurations of hard-wall confined As are shown in fig. S71 for the neutral atom and in fig. S72 for the cation. The ground state of the unconfined As is [Ar]$4s^2 3d^{10} 4p^3$. At $r_c = 1.2a_0$ the ground state changes to [Ar]$3d^{10} 4f^5$.

At $r_c = 1.2a_0$ the state [Ar]$3d^{10} 4d^1 4f^4$ flips below the initial ground state. At $r_c = 1.2a_0$ the state [Ar]$4s^1 3d^{10} 4f^4$ flips below the initial ground state. At $r_c = 1.2a_0$ the state [Ar]$4s^2 3d^{10} 4f^3$ flips below the initial ground state. At $r_c = 1.2a_0$ the state [Ar]$4s^2 3d^{10} 4p^2 4f^1$ flips below the initial ground state. At $r_c = 1.0a_0$ the state [Ar]$4s^2 3d^{10} 4p^2 4d^1$ flips below the initial ground state. The ionization energy of the unconfined atom is $\Delta E_0 = 9.32$ eV. The studied configurations, atomic radii and excitation energies in the unconfined calculations are as follows:

configuration	r_ϵ	r_ρ	$r_{\max}$	ΔE
$[Ar]4s^2 3d^{10} 4p^3$	5.18	4.46	1.99	0.00
$[Ar]4s^2 3d^{10} 4p^2 5s^1$	10.05	4.51	5.66	6.12
$[Ar]4s^2 3d^{10} 4p^2 4d^1$	15.48	4.16	7.80	7.71
$[Ar]4s^2 3d^{10} 4p^2 4f^1$	26.49	4.00	17.46	8.48
$[Ar]4s^1 3d^{10} 4p^4$	5.23	4.51	1.97	9.26
$[Ar]4s^2 3d^{10} 4f^3$	16.07	5.35	6.69	41.18
$[Ar]4s^1 3d^{10} 4f^4$	14.18	6.63	5.03	74.62
$[Ar]3d^{10} 4d^1 4f^4$	13.99	6.63	4.63	101.87
$[Ar]3d^{10} 4f^5$	12.87	6.79	4.11	111.19

Se The energies of the low lying configurations of hard-wall confined Se are shown in fig. S73 for the neutral atom and in fig. S74 for the cation. The ground state of the unconfined Se is $[Ar]4s^2 3d^{10} 4p^4$. At $r_c = 1.2a_0$ the ground state changes to $[Ar]4s^2 3d^{10} 4f^4$. Furthermore, at $r_c = 1.1a_0$ we see a ground state crossing to $[Ar]3d^{10} 4f^6$.

At $r_c = 1.2a_0$ the state $[Ar]4s^1 3d^{10} 4f^5$ flips below the initial ground state. At $r_c = 1.2a_0$ the state $[Ar]4s^2 3d^{10} 4p^1 4f^3$ flips below the initial ground state. At $r_c = 1.2a_0$ the state $[Ar]4s^2 3d^{10} 4p^3 4f^1$ flips below the initial ground state. At $r_c = 1.1a_0$ the state $[Ar]3d^{10} 4d^1 4f^5$ flips below the initial ground state. At $r_c = 1.0a_0$ the state $[Ar]4s^2 3d^{10} 4p^3 4d^1$ flips below the initial ground state. The ionization energy of the unconfined atom is $\Delta E_0 = 11.11$ eV. The studied configurations, atomic radii and excitation energies in the unconfined calculations are as follows:

configuration	r_ϵ	r_ρ	$r_{\max}$	ΔE
$[Ar]4s^2 3d^{10} 4p^4$	4.85	4.28	1.81	0.00
$[Ar]4s^2 3d^{10} 4p^3 5s^1$	9.64	4.57	5.35	7.75
$[Ar]4s^2 3d^{10} 4p^3 4d^1$	15.36	4.13	7.61	9.47
$[Ar]4s^2 3d^{10} 4p^3 4f^1$	26.52	3.96	17.48	10.27
$[Ar]4s^1 3d^{10} 4p^5$	4.87	4.34	1.80	10.65
$[Ar]4s^2 3d^{10} 4p^1 4f^3$	16.07	5.35	6.63	48.75
$[Ar]4s^2 3d^{10} 4f^4$	14.18	6.63	5.00	74.41
$[Ar]4s^1 3d^{10} 4f^5$	12.93	6.77	4.00	116.02
$[Ar]3d^{10} 4d^1 4f^5$	12.85	6.63	3.77	148.12
$[Ar]3d^{10} 4f^6$	11.97	6.57	3.41	160.40

Br The energies of the low lying configurations of hard-wall confined Br are shown in fig. S75 for the neutral atom and in fig. S76 for the cation. The ground state of the unconfined Br is $[Ar]4s^2 3d^{10} 4p^5$. At $r_c = 1.1a_0$ the ground state changes to $[Ar]3d^{10} 4f^7$.

At $r_c = 1.1a_0$ the state $[Ar]4s^1 3d^{10} 4f^6$ flips below the initial ground state. At $r_c = 1.1a_0$ the state $[Ar]3d^{10} 4p^1 4f^6$ flips below the initial ground state. At $r_c = 1.1a_0$ the state $[Ar]4s^2 3d^{10} 4p^4 4f^1$ flips below the initial ground state. At $r_c = 1.1a_0$ the state $[Ar]4s^2 3d^{10} 4p^3 4f^2$ flips below the initial ground state. The ionization energy of the unconfined atom is $\Delta E_0 = 12.90$ eV. The studied configurations, atomic radii and excitation energies in the unconfined calculations are as follows:

configuration	r_ϵ	r_ρ	$r_{\max}$	ΔE
$[Ar]4s^2 3d^{10} 4p^5$	4.56	4.16	1.68	0.00
$[Ar]4s^2 3d^{10} 4p^4 5s^1$	9.31	4.57	5.09	9.40
$[Ar]4s^2 3d^{10} 4p^4 4d^1$	15.35	4.03	7.55	11.25
$[Ar]4s^1 3d^{10} 4p^6$	4.57	4.18	1.66	12.04
$[Ar]4s^2 3d^{10} 4p^4 4f^1$	26.55	3.88	17.50	12.07
$[Ar]4s^2 3d^{10} 4p^3 4f^2$	19.41	3.66	9.79	31.24
$[Ar]4s^1 3d^{10} 4f^6$	12.05	6.63	3.32	168.10
$[Ar]3d^{10} 4p^1 4f^6$	12.06	6.63	3.28	182.96
$[Ar]3d^{10} 4f^7$	11.32	6.47	2.91	220.33

Kr The energies of the low lying configurations of hard-wall confined Kr are shown in fig. S77 for the neutral atom and in fig. S78 for the cation. The ground state of the unconfined Kr is $[Ar]4s^2 3d^{10} 4p^6$. At $r_c = 1.1a_0$ the ground state changes to $[Ar]4s^1 3d^{10} 4f^7$. Furthermore, at $r_c = 1.0a_0$ we see a ground state crossing to $[Ar]3d^{10} 4f^8$.

At $r_c = 1.1a_0$ the state $[Ar]3d^{10} 4p^1 4f^7$ flips below the initial ground state. At $r_c = 1.1a_0$ the state $[Ar]4s^2 3d^{10} 4p^5 4f^1$ flips below the initial ground state. At $r_c = 1.1a_0$ the state $[Ar]4s^2 3d^{10} 4p^4 4f^2$ flips below the initial ground state. The ionization energy of the unconfined atom is $\Delta E_0 = 14.72$ eV. The studied configurations, atomic radii and excitation energies in the unconfined calculations are as follows:

configuration	r_ϵ	r_ρ	$r_{\max}$	ΔE
[Ar]$4s^2 3d^{10} 4p^6$	4.31	4.03	1.56	0.00
[Ar]$4s^2 3d^{10} 4p^5 5s^1$	9.02	4.51	4.88	11.09
[Ar]$4s^2 3d^{10} 4p^5 4d^1$	15.40	3.96	7.59	13.06
[Ar]$4s^2 3d^{10} 4p^5 4f^1$	26.57	3.77	17.52	13.89
[Ar]$4s^1 3d^{10} 4p^6 4d^1$	15.17	4.00	7.22	27.05
[Ar]$4s^2 3d^{10} 4p^4 4f^2$	19.43	3.55	9.81	35.58
[Ar]$4s^1 3d^{10} 4f^7$	11.42	6.48	2.83	231.30
[Ar]$3d^{10} 4p^1 4f^7$	11.43	6.48	2.80	247.79
[Ar]$3d^{10} 4f^8$	10.83	6.25	2.54	291.40

Rb The energies of the low lying configurations of hard-wall confined Rb are shown in fig. S79 for the neutral atom and in fig. S80 for the cation. The ground state of the unconfined Rb is [Kr]$5s^1$. At $r_c = 5.0a_0$ the ground state changes to [Kr]$4d^1$. Furthermore, at $r_c = 1.2a_0$ we see a ground state crossing to [Kr]$4f^1$. At $r_c = 1.1a_0$ we see a ground state crossing to [Ar]$4s^2 3d^{10} 4p^5 4f^2$. Moreover, at $r_c = 1.0a_0$ we see a ground state crossing to [Ar]$3d^{10} 4f^9$.

At $r_c = 3.0a_0$ the state [Ar]$4s^2 3d^{10} 4p^5 4d^2$ flips below the initial ground state. At $r_c = 1.3a_0$ the state [Ar]$4s^1 3d^{10} 4f^8$ flips below the initial ground state. At $r_c = 1.2a_0$ the state [Ar]$3d^{10} 4p^1 4f^8$ flips below the initial ground state. At $r_c = 1.1a_0$ the state [Kr]$5p^1$ flips below the initial ground state. The ionization energy of the unconfined atom is $\Delta E_0 = 3.75$ eV. The studied configurations, atomic radii and excitation energies in the unconfined calculations are as follows:

configuration	r_ϵ	r_ρ	$r_{\max}$	ΔE
[Kr]$5s^1$	8.77	4.46	4.69	0.00
[Kr]$5p^1$	11.86	3.83	6.59	1.37
[Kr]$4d^1$	15.48	3.88	7.68	2.09
[Kr]$4f^1$	26.58	3.72	17.54	2.91
[Ar]$4s^2 3d^{10} 4p^5 4d^2$	10.72	4.80	2.36	20.61
[Ar]$4s^2 3d^{10} 4p^5 4f^2$	19.44	3.47	9.83	27.13
[Ar]$4s^1 3d^{10} 4f^8$	10.96	6.33	2.46	293.19
[Ar]$3d^{10} 4p^1 4f^8$	10.97	6.33	2.44	311.29
[Ar]$3d^{10} 4f^9$	10.47	6.15	2.25	361.16

Sr The energies of the low lying configurations of hard-wall confined Sr are shown in fig. S81 for the neutral atom and in fig. S82 for the cation. The ground state of the unconfined Sr is [Kr]$5s^2$. At $r_c = 4.9a_0$ the ground state changes to [Kr]$4d^2$. Furthermore, at $r_c = 1.2a_0$ we see a ground state crossing to [Kr]$4f^2$. At $r_c = 1.0a_0$ we see a ground state crossing to [Ar]$3d^{10} 4f^{10}$.

At $r_c = 4.9a_0$ the state [Kr]$5s^1 4d^1$ flips below the initial ground state. At $r_c = 3.4a_0$ the state [Kr]$4d^1 4f^1$ flips below the initial ground state. At $r_c = 3.4a_0$ the state [Ar]$4s^2 3d^{10} 4p^5 4d^3$ flips below the initial ground state. At $r_c = 1.5a_0$ the state [Ar]$4s^2 3d^{10} 4f^8$ flips below the initial ground state. At $r_c = 1.4a_0$ the state [Ar]$4s^1 3d^{10} 4f^9$ flips below the initial ground state. At $r_c = 1.1a_0$ the state [Kr]$5s^1 5p^1$ flips below the initial ground state. The ionization energy of the unconfined atom is $\Delta E_0 = 5.79$ eV. The studied configurations, atomic radii and excitation energies in the unconfined calculations are as follows:

configuration	r_ϵ	r_ρ	$r_{\max}$	ΔE
[Kr]$5s^2$	7.76	5.39	3.83	0.00
[Kr]$5s^1 5p^1$	9.12	5.56	4.81	2.25
[Kr]$5s^1 4d^1$	8.64	5.10	3.99	2.31
[Kr]$4d^2$	10.70	4.67	2.14	4.48
[Kr]$4d^1 4f^1$	25.93	4.07	16.78	6.38
[Kr]$4f^2$	19.45	3.44	9.85	11.35
[Ar]$4s^2 3d^{10} 4p^5 4d^3$	8.73	4.93	1.98	26.60
[Ar]$4s^2 3d^{10} 4f^8$	11.06	6.27	2.39	285.45
[Ar]$4s^1 3d^{10} 4f^9$	10.61	6.14	2.18	359.57
[Ar]$3d^{10} 4f^{10}$	10.20	5.94	2.02	435.44

Y The energies of the low lying configurations of hard-wall confined Y are shown in fig. S83 for the neutral atom and in fig. S84 for the cation. The ground state of the unconfined Y is [Kr]$5s^2 4d^1$. At $r_c = 5.0a_0$ the ground state changes to [Kr]$4d^3$. Furthermore, at $r_c = 1.2a_0$ we see a ground state crossing to [Kr]$4d^2 4f^1$. At $r_c = 1.1a_0$ we see a ground state crossing to [Kr]$4d^1 4f^2$. Moreover, at $r_c = 1.0a_0$ we see a ground state crossing to [Ar]$4s^2 3d^{10} 4f^9$.

At $r_c = 5.0a_0$ the state [Kr]$5s^1 4d^2$ flips below the initial ground state. At $r_c = 3.5a_0$ the state [Kr]$4d^2 5p^1$ flips below the initial ground state. At $r_c = 3.3a_0$ the state [Ar]$4s^2 3d^{10} 4p^5 4d^4$ flips below the initial ground state. At $r_c = 1.4a_0$ the state [Ar]$4s^2 3d^{10} 4p^1 4f^8$ flips below the initial ground state. At $r_c = 1.3a_0$ the state [Ar]$4s^1 3d^{10} 4f^{10}$ flips below the initial ground

state. The ionization energy of the unconfined atom is $\Delta E_0 = 6.42$ eV. The studied configurations, atomic radii and excitation energies in the unconfined calculations are as follows:

configuration	r_ϵ	r_ρ	$r_{\max}$	ΔE
[Kr]$5s^24d^1$	7.23	5.31	3.49	0.00
[Kr]$5s^14d^2$	7.58	5.12	3.64	1.67
[Kr]$5s^25p^1$	7.88	5.64	4.05	1.98
[Kr]$4d^3$	8.65	4.84	1.84	3.51
[Kr]$4d^25p^1$	9.46	5.08	4.83	3.91
[Kr]$4d^24f^1$	26.09	4.23	16.98	6.28
[Kr]$4d^14f^2$	19.29	3.74	9.59	13.12
[Ar]$4s^23d^{10}4p^54d^4$	7.54	4.89	1.76	28.89
[Ar]$4s^23d^{10}4p^14f^8$	11.19	6.27	2.31	286.85
[Ar]$4s^23d^{10}4f^9$	10.75	6.14	2.11	346.61
[Ar]$4s^13d^{10}4f^{10}$	10.37	5.92	1.96	428.87

Zr The energies of the low lying configurations of hard-wall confined Zr are shown in fig. S85 for the neutral atom and in fig. S86 for the cation. The ground state of the unconfined Zr is [Kr]$5s^24d^2$. At $r_c = 5.3a_0$ the ground state changes to [Kr]$5s^14d^3$. Furthermore, at $r_c = 5.1a_0$ we see a ground state crossing to [Kr]$4d^4$. At $r_c = 1.1a_0$ we see a ground state crossing to [Kr]$4d^24f^2$. Moreover, at $r_c = 1.0a_0$ we see a ground state crossing to [Ar]$4s^23d^{10}4f^{10}$.

At $r_c = 3.4a_0$ the state [Kr]$4d^35p^1$ flips below the initial ground state. At $r_c = 3.2a_0$ the state [Kr]$4d^34f^1$ flips below the initial ground state. At $r_c = 3.2a_0$ the state [Ar]$4s^23d^{10}4p^54d^5$ flips below the initial ground state. At $r_c = 1.4a_0$ the state [Ar]$4s^23d^{10}4p^14f^9$ flips below the initial ground state. At $r_c = 1.4a_0$ the state [Ar]$4s^23d^{10}4p^24f^8$ flips below the initial ground state. The ionization energy of the unconfined atom is $\Delta E_0 = 6.72$ eV. The studied configurations, atomic radii and excitation energies in the unconfined calculations are as follows:

configuration	r_ϵ	r_ρ	$r_{\max}$	ΔE
[Kr]$5s^24d^2$	6.83	5.16	3.27	0.00
[Kr]$5s^14d^3$	6.98	5.00	3.41	0.92
[Kr]$4d^4$	7.43	4.79	1.64	2.28
[Kr]$4d^35p^1$	9.11	5.03	4.55	3.40
[Kr]$4d^34f^1$	26.22	4.23	17.12	5.88
[Kr]$4d^24f^2$	19.27	3.82	9.55	14.38
[Ar]$4s^23d^{10}4p^54d^5$	6.73	4.79	1.60	30.78
[Ar]$4s^23d^{10}4p^24f^8$	11.32	6.14	2.24	282.61
[Ar]$4s^23d^{10}4p^14f^9$	10.91	6.02	2.03	347.83
[Ar]$4s^23d^{10}4f^{10}$	10.53	5.92	1.89	414.33

Nb The energies of the low lying configurations of hard-wall confined Nb are shown in fig. S87 for the neutral atom and in fig. S88 for the cation. The ground state of the unconfined Nb is [Kr]$5s^24d^3$. At $r_c = 6.6a_0$ the ground state changes to [Kr]$5s^14d^4$. Furthermore, at $r_c = 5.5a_0$ we see a ground state crossing to [Kr]$4d^5$. At $r_c = 1.1a_0$ we see a ground state crossing to [Kr]$4f^5$.

At $r_c = 3.4a_0$ the state [Kr]$4d^45p^1$ flips below the initial ground state. At $r_c = 3.1a_0$ the state [Kr]$4d^44f^1$ flips below the initial ground state. At $r_c = 3.1a_0$ the state [Ar]$4s^23d^{10}4p^54d^6$ flips below the initial ground state. At $r_c = 2.5a_0$ the state [Kr]$4d^34f^2$ flips below the initial ground state. At $r_c = 1.6a_0$ the state [Ar]$4s^23d^{10}4p^54f^6$ flips below the initial ground state. At $r_c = 1.5a_0$ the state [Ar]$4s^23d^{10}4p^44f^7$ flips below the initial ground state. The ionization energy of the unconfined atom is $\Delta E_0 = 6.16$ eV. The studied configurations, atomic radii and excitation energies in the unconfined calculations are as follows:

configuration	r_ϵ	r_ρ	$r_{\max}$	ΔE
[Kr]$5s^24d^3$	6.53	5.06	3.10	0.00
[Kr]$5s^14d^4$	6.58	4.89	3.24	0.11
[Kr]$4d^5$	6.62	4.67	1.50	0.90
[Kr]$4d^45p^1$	8.92	4.93	4.36	2.78
[Kr]$4d^44f^1$	26.31	4.15	17.20	5.32
[Kr]$4d^34f^2$	19.29	3.82	9.56	15.39
[Ar]$4s^23d^{10}4p^54d^6$	6.13	4.61	1.47	32.48
[Kr]$4f^5$	13.13	6.54	3.78	74.33
[Ar]$4s^23d^{10}4p^54f^6$	12.39	6.40	3.02	137.49
[Ar]$4s^23d^{10}4p^44f^7$	11.85	6.27	2.52	203.96

Mo The energies of the low lying configurations of hard-wall confined Mo are shown in fig. S89 for the neutral atom and in fig. S90 for the cation. The ground state of the unconfined Mo is [Kr]$5s^1 4d^5$. At $r_c = 6.7a_0$ the ground state changes to [Kr]$4d^6$. Furthermore, at $r_c = 1.0a_0$ we see a ground state crossing to [Kr]$4f^6$.

At $r_c = 2.5a_0$ the state [Ar]$4s^2 3d^{10} 4p^5 4d^7$ flips below the initial ground state. At $r_c = 2.4a_0$ the state [Kr]$4d^5 4f^1$ flips below the initial ground state. At $r_c = 1.9a_0$ the state [Kr]$4d^4 4f^2$ flips below the initial ground state. At $r_c = 1.4a_0$ the state [Kr]$4d^1 4f^5$ flips below the initial ground state. At $r_c = 1.3a_0$ the state [Ar]$4s^2 3d^{10} 4p^5 4f^7$ flips below the initial ground state. At $r_c = 1.0a_0$ the state [Kr]$4d^5 5p^1$ flips below the initial ground state. The ionization energy of the unconfined atom is $\Delta E_0 = 6.25$ eV. The studied configurations, atomic radii and excitation energies in the unconfined calculations are as follows:

configuration	r_ϵ	r_ρ	$r_{\max}$	ΔE
[Kr]$5s^1 4d^5$	6.29	4.79	3.10	0.00
[Kr]$4d^6$	6.02	4.54	1.39	0.19
[Kr]$5s^2 4d^4$	6.30	4.97	2.97	0.75
[Kr]$4d^5 5p^1$	8.80	4.84	4.23	2.83
[Kr]$4d^5 4f^1$	26.38	4.07	17.25	5.41
[Kr]$4d^4 4f^2$	19.31	3.74	9.62	17.00
[Ar]$4s^2 3d^{10} 4p^5 4d^7$	5.67	4.54	1.37	34.79
[Kr]$4d^1 4f^5$	13.18	6.54	3.69	83.47
[Kr]$4f^6$	12.42	6.40	3.02	112.55
[Ar]$4s^2 3d^{10} 4p^5 4f^7$	11.92	6.27	2.49	184.22

Tc The energies of the low lying configurations of hard-wall confined Tc are shown in fig. S91 for the neutral atom and in fig. S92 for the cation. The ground state of the unconfined Tc is [Kr]$4d^7$. At $r_c = 1.0a_0$ the ground state changes to [Kr]$4f^7$.

At $r_c = 1.0a_0$ the state [Kr]$4d^1 4f^6$ flips below the initial ground state. At $r_c = 1.0a_0$ the state [Kr]$4d^2 4f^5$ flips below the initial ground state. At $r_c = 1.0a_0$ the state [Kr]$4d^6 4f^1$ flips below the initial ground state. At $r_c = 1.0a_0$ the state [Kr]$4d^5 4f^2$ flips below the initial ground state. The ionization energy of the unconfined atom is $\Delta E_0 = 6.87$ eV. The studied configurations, atomic radii and excitation energies in the unconfined calculations are as follows:

configuration	r_ϵ	r_ρ	$r_{\max}$	ΔE
[Kr]$4d^7$	5.57	4.44	1.30	0.00
[Kr]$5s^1 4d^6$	6.07	4.72	2.98	0.46
[Kr]$5s^2 4d^5$	6.11	4.89	2.86	2.10
[Kr]$4d^6 5p^1$	8.73	4.79	4.12	3.43
[Kr]$4d^6 4f^1$	26.44	3.92	17.37	6.03
[Kr]$4d^5 4f^2$	19.34	3.59	9.66	19.09
[Ar]$4s^2 3d^{10} 4p^5 4d^8$	5.30	4.37	1.28	37.63
[Kr]$4d^2 4f^5$	13.23	6.54	3.64	93.10
[Kr]$4d^1 4f^6$	12.51	6.40	2.94	125.26
[Kr]$4f^7$	11.98	6.27	2.47	159.60

Ru The energies of the low lying configurations of hard-wall confined Ru are shown in fig. S93 for the neutral atom and in fig. S94 for the cation. The ground state of the unconfined Ru is [Kr]$4d^8$. At $r_c = 1.0a_0$ the ground state changes to [Kr]$4d^3 4f^5$.

At $r_c = 1.0a_0$ the state [Kr]$4d^2 4f^6$ flips below the initial ground state. At $r_c = 1.0a_0$ the state [Kr]$4d^1 4f^7$ flips below the initial ground state. At $r_c = 1.0a_0$ the state [Kr]$4d^7 4f^1$ flips below the initial ground state. The ionization energy of the unconfined atom is $\Delta E_0 = 7.68$ eV. The studied configurations, atomic radii and excitation energies in the unconfined calculations are as follows:

configuration	r_ϵ	r_ρ	$r_{\max}$	ΔE
[Kr]$4d^8$	5.20	4.30	1.22	0.00
[Kr]$5s^1 4d^7$	5.90	4.57	2.88	1.14
[Kr]$5s^2 4d^6$	5.96	4.79	2.76	3.69
[Kr]$4d^7 5p^1$	8.68	4.65	4.04	4.22
[Kr]$4d^7 4f^1$	26.47	3.80	17.42	6.84
[Ar]$4s^2 3d^{10} 4p^5 4d^9$	4.99	4.24	1.20	40.65
[Kr]$4d^3 4f^5$	13.27	6.54	3.62	102.90
[Kr]$4d^2 4f^6$	12.60	6.40	2.88	138.19
[Kr]$4d^1 4f^7$	12.10	6.27	2.38	175.78

Rh The energies of the low lying configurations of hard-wall confined Rh are shown in fig. S95 for the neutral atom and in fig. S96 for the cation. The ground state of the unconfined Rh is [Kr]$4d^9$. At $r_c = 5.0a_0$ the ground state changes to [Kr]$5s^1 4d^8$. Furthermore, at $r_c = 4.9a_0$ we see a ground state crossing to [Kr]$4d^9$. We do not observe any ground state

crossing for Rh in the considered confinement radii.

The ionization energy of the unconfined atom is $\Delta E_0 = 8.49$ eV. The studied configurations, atomic radii and excitation energies in the unconfined calculations are as follows:

configuration	r_ϵ	r_ρ	$r_{\max}$	ΔE
[Kr]$4d^9$	4.90	4.15	1.15	0.00
[Kr]$5s^1 4d^8$	5.76	4.50	2.80	1.84
[Kr]$4d^8 5p^1$	8.65	4.55	3.97	5.02
[Kr]$5s^2 4d^7$	5.82	4.74	2.67	5.32
[Kr]$4d^8 4f^1$	26.50	3.70	17.45	7.65
[Kr]$4d^7 4f^2$	19.39	3.51	9.73	23.59
[Ar]$4s^2 3d^{10} 4p^5 4d^{10}$	4.73	4.11	1.14	43.68

Pd The energies of the low lying configurations of hard-wall confined Pd are shown in fig. S97 for the neutral atom and in fig. S98 for the cation. The ground state of the unconfined Pd is [Kr]$4d^{10}$. We do not observe any ground state crossing for Pd in the considered confinement radii.

The ionization energy of the unconfined atom is $\Delta E_0 = 9.30$ eV. The studied configurations, atomic radii and excitation energies in the unconfined calculations are as follows:

configuration	r_ϵ	r_ρ	$r_{\max}$	ΔE
[Kr]$4d^{10}$	4.64	4.04	1.09	0.00
[Kr]$5s^1 4d^9$	5.66	4.39	2.72	2.56
[Kr]$4d^9 5p^1$	8.63	4.47	3.92	5.84
[Kr]$5s^2 4d^8$	5.71	4.67	2.60	6.99
[Kr]$4d^9 4f^1$	26.53	3.65	17.48	8.47
[Kr]$4d^8 4f^2$	19.41	3.41	9.77	25.82
[Ar]$4s^2 3d^{10} 4p^5 4d^{10} 4f^1$	26.54	3.65	17.49	55.91

Ag The energies of the low lying configurations of hard-wall confined Ag are shown in fig. S99 for the neutral atom and in fig. S100 for the cation. The ground state of the unconfined Ag is [Kr]$5s^1 4d^{10}$. At $r_c = 2.1a_0$ the ground state changes to [Kr]$4d^{10} 4f^1$.

At $r_c = 1.6a_0$ the state [Kr]$4d^9 4f^2$ flips below the initial ground state. At $r_c = 1.5a_0$ the state [Ar]$4s^2 3d^{10} 4p^5 4d^{10} 4f^2$ flips below the initial ground state. At $r_c = 1.4a_0$ the state [Kr]$4d^8 4f^3$ flips below the initial ground state. The ionization energy of the unconfined atom is $\Delta E_0 = 6.83$ eV. The studied configurations, atomic radii and excitation energies in the unconfined calculations are as follows:

configuration	r_ϵ	r_ρ	$r_{\max}$	ΔE
[Kr]$5s^1 4d^{10}$	5.57	4.31	2.66	0.00
[Kr]$4d^{10} 5p^1$	8.62	4.39	3.88	3.37
[Kr]$5s^2 4d^9$	5.61	4.61	2.53	5.41
[Kr]$4d^{10} 4f^1$	26.55	3.56	17.50	6.00
[Kr]$4d^9 4f^2$	19.42	3.37	9.79	24.75
[Kr]$4d^8 4f^3$	16.09	5.21	6.60	52.47
[Ar]$4s^2 3d^{10} 4p^5 4d^{10} 4f^2$	19.42	3.37	9.79	76.15

Cd The energies of the low lying configurations of hard-wall confined Cd are shown in fig. S101 for the neutral atom and in fig. S102 for the cation. The ground state of the unconfined Cd is [Kr]$5s^2 4d^{10}$. At $r_c = 2.0a_0$ the ground state changes to [Kr]$4d^{10} 4f^2$.

At $r_c = 2.0a_0$ the state [Kr]$5s^1 4d^{10} 4f^1$ flips below the initial ground state. At $r_c = 1.8a_0$ the state [Kr]$4d^9 4f^3$ flips below the initial ground state. At $r_c = 1.8a_0$ the state [Kr]$4d^{10} 5p^1 4f^1$ flips below the initial ground state. At $r_c = 1.6a_0$ the state [Kr]$4d^8 4f^4$ flips below the initial ground state. At $r_c = 1.6a_0$ the state [Ar]$4s^2 3d^{10} 4p^5 4d^{10} 4f^3$ flips below the initial ground state. The ionization energy of the unconfined atom is $\Delta E_0 = 8.77$ eV. The studied configurations, atomic radii and excitation energies in the unconfined calculations are as follows:

configuration	r_ϵ	r_ρ	$r_{\max}$	ΔE
[Kr]$5s^2 4d^{10}$	5.52	4.54	2.47	0.00
[Kr]$5s^1 4d^{10} 5p^1$	7.16	4.79	3.25	4.37
[Kr]$5s^1 4d^{10} 5d^1$	16.42	4.07	8.91	7.26
[Kr]$5s^1 4d^{10} 4f^1$	26.46	3.99	17.32	7.93
[Kr]$4d^{10} 5p^2$	7.53	5.06	3.21	8.79
[Kr]$4d^{10} 5p^1 4f^1$	26.01	4.31	16.88	13.07
[Kr]$4d^{10} 4f^2$	19.43	3.31	9.81	19.83
[Kr]$4d^9 4f^3$	16.09	5.19	6.62	49.51
[Kr]$4d^8 4f^4$	14.33	6.47	4.85	87.42
[Ar]$4s^2 3d^{10} 4p^5 4d^{10} 4f^3$	16.10	5.27	6.62	104.92

In The energies of the low lying configurations of hard-wall confined In are shown in fig. S103 for the neutral atom and in fig. S104 for the cation. The ground state of the unconfined In is [Kr]$5s^2 4d^{10} 5p^1$. At $r_c = 4.5a_0$ the ground state changes to [Kr]$5s^1 4d^{10} 5p^2$. Fur-

thermore, at $r_c = 4.4a_0$ we see a ground state crossing to [Kr]$5s^2 4d^{10} 5p^1$. At $r_c = 2.2a_0$ we see a ground state crossing to [Kr]$5s^2 4d^{10} 4f^1$. Moreover, at $r_c = 2.0a_0$ we see a ground state crossing to [Kr]$4d^{10} 4f^3$.

At $r_c = 2.1a_0$ the state [Kr]$5s^1 4d^{10} 4f^2$ flips below the initial ground state. At $r_c = 2.0a_0$ the state [Kr]$4d^{10} 5p^1 4f^2$ flips below the initial ground state. At $r_c = 1.8a_0$ the state [Kr]$4d^9 4f^4$ flips below the initial ground state. At $r_c = 1.7a_0$ the state [Kr]$4d^8 4f^5$ flips below the initial ground state. At $r_c = 1.7a_0$ the state [Ar]$4s^2 3d^{10} 4p^5 4d^{10} 4f^4$ flips below the initial ground state. At $r_c = 1.0a_0$ the state [Kr]$5s^2 4d^{10} 5d^1$ flips below the initial ground state. The ionization energy of the unconfined atom is $\Delta E_0 = 5.32$ eV. The studied configurations, atomic radii and excitation energies in the unconfined calculations are as follows:

configuration	r_ϵ	r_ρ	$r_{\max}$	ΔE
[Kr]$5s^2 4d^{10} 5p^1$	6.30	4.74	2.87	0.00
[Kr]$5s^2 4d^{10} 6s^1$	12.17	4.23	7.28	2.76
[Kr]$5s^2 4d^{10} 5d^1$	16.37	4.26	8.85	3.83
[Kr]$5s^2 4d^{10} 4f^1$	26.60	4.15	17.50	4.49
[Kr]$5s^1 4d^{10} 5p^2$	6.66	4.93	2.84	5.36
[Kr]$5s^1 4d^{10} 4f^2$	19.30	3.75	9.61	18.92
[Kr]$4d^{10} 5p^1 4f^2$	19.08	4.20	9.24	25.52
[Kr]$4d^{10} 4f^3$	16.10	5.19	6.65	36.33
[Kr]$4d^9 4f^4$	14.33	6.47	4.88	76.78
[Kr]$4d^8 4f^5$	13.33	6.47	3.68	123.92
[Ar]$4s^2 3d^{10} 4p^5 4d^{10} 4f^4$	14.34	6.47	4.86	136.18

Sn The energies of the low lying configurations of hard-wall confined Sn are shown in fig. S105 for the neutral atom and in fig. S106 for the cation. The ground state of the unconfined Sn is [Kr]$5s^2 4d^{10} 5p^2$. At $r_c = 4.3a_0$ the ground state changes to [Kr]$5s^2 4d^{10} 5p^1 4f^1$. Furthermore, at $r_c = 4.2a_0$ we see a ground state crossing to [Kr]$5s^2 4d^{10} 5p^2$. At $r_c = 2.2a_0$ we see a ground state crossing to [Kr]$5s^2 4d^{10} 4f^2$. Moreover, at $r_c = 1.9a_0$ we see a ground state crossing to [Kr]$4d^{10} 4f^4$.

At $r_c = 2.1a_0$ the state [Kr]$5s^1 4d^{10} 4f^3$ flips below the initial ground state. At $r_c = 1.9a_0$ the state [Kr]$4d^9 4f^5$ flips below the initial ground state. At $r_c = 1.8a_0$ the state [Ar]$4s^2 3d^{10} 4p^5 4d^{10} 4f^5$ flips below the initial ground state. At $r_c = 1.7a_0$ the state [Kr]$4d^8 4f^6$ flips below the initial ground state. The ionization energy of the unconfined atom is $\Delta E_0 = 6.97$ eV. The studied configurations, atomic radii and excitation energies in the unconfined calculations are as follows:

configuration	r_ϵ	r_ρ	$r_{\max}$	ΔE
[Kr]$5s^2 4d^{10} 5p^2$	6.05	4.80	2.58	0.00
[Kr]$5s^2 4d^{10} 5p^1 6s^1$	11.36	4.59	6.69	4.18
[Kr]$5s^2 4d^{10} 5p^1 5d^1$	15.25	4.54	7.62	5.34
[Kr]$5s^2 4d^{10} 5p^1 4f^1$	26.39	4.32	17.34	6.13
[Kr]$5s^1 4d^{10} 5p^3$	6.18	4.91	2.56	6.35
[Kr]$5s^2 4d^{10} 4f^2$	19.34	3.88	9.66	16.07
[Kr]$5s^1 4d^{10} 4f^3$	16.06	5.36	6.39	36.31
[Kr]$4d^{10} 4f^4$	14.32	6.47	4.91	58.43
[Kr]$4d^9 4f^5$	13.32	6.47	3.71	108.63
[Kr]$4d^8 4f^6$	12.81	6.36	2.85	163.73
[Ar]$4s^2 3d^{10} 4p^5 4d^{10} 4f^5$	13.36	6.47	3.69	171.92

Sb The energies of the low lying configurations of hard-wall confined Sb are shown in fig. S107 for the neutral atom and in fig. S108 for the cation. The ground state of the unconfined Sb is [Kr]$5s^2 4d^{10} 5p^3$. At $r_c = 2.1a_0$ the ground state changes to [Kr]$5s^2 4d^{10} 4f^3$. Furthermore, at $r_c = 1.9a_0$ we see a ground state crossing to [Kr]$4d^{10} 4f^5$.

At $r_c = 2.1a_0$ the state [Kr]$5s^1 4d^{10} 4f^4$ flips below the initial ground state. At $r_c = 2.1a_0$ the state [Kr]$5s^2 4d^{10} 5p^1 4f^2$ flips below the initial ground state. At $r_c = 2.1a_0$ the state [Kr]$5s^2 4d^{10} 5p^2 4f^1$ flips below the initial ground state. At $r_c = 1.9a_0$ the state [Kr]$4d^9 4f^6$ flips below the initial ground state. At $r_c = 1.8a_0$ the state [Kr]$4d^8 4f^7$ flips below the initial ground state. At $r_c = 1.8a_0$ the state [Ar]$4s^2 3d^{10} 4p^5 4d^{10} 4f^6$ flips below the initial ground state. The ionization energy of the unconfined atom is $\Delta E_0 = 8.49$ eV. The studied configurations, atomic radii and excitation energies in the unconfined calculations are as follows:

configuration	r_ϵ	r_ρ	$r_{\max}$	ΔE
[Kr]$5s^2 4d^{10} 5p^3$	5.72	4.74	2.37	0.00
[Kr]$5s^2 4d^{10} 5p^2 6s^1$	10.86	4.70	6.29	5.55
[Kr]$5s^2 4d^{10} 5p^2 5d^1$	14.64	4.60	6.77	6.78
[Kr]$5s^1 4d^{10} 5p^4$	5.78	4.81	2.35	7.32
[Kr]$5s^2 4d^{10} 5p^2 4f^1$	26.41	4.38	17.34	7.65
[Kr]$5s^2 4d^{10} 5p^1 4f^2$	19.25	4.13	9.49	19.70
[Kr]$5s^2 4d^{10} 4f^3$	16.07	5.33	6.27	34.40
[Kr]$5s^1 4d^{10} 4f^4$	14.38	6.32	4.57	59.19
[Kr]$4d^{10} 4f^5$	13.31	6.46	3.74	84.81
[Kr]$4d^9 4f^6$	12.81	6.32	2.88	143.36
[Kr]$4d^8 4f^7$	12.55	6.17	2.16	205.39
[Ar]$4s^2 3d^{10} 4p^5 4d^{10} 4f^6$	12.86	6.32	2.84	210.42

Te The energies of the low lying configurations of hard-wall confined Te are shown in fig. S109 for the neutral atom and in fig. S110 for the cation. The ground state of the unconfined Te is [Kr]$5s^2 4d^{10} 5p^4$. At $r_c = 2.1a_0$ the ground state changes to [Kr]$5s^2 4d^{10} 4f^4$. Furthermore, at $r_c = 1.8a_0$ we see a ground state crossing to [Kr]$4d^{10} 4f^6$.

At $r_c = 2.1a_0$ the state [Kr]$5s^2 4d^{10} 5p^1 4f^3$ flips below the initial ground state. At $r_c = 2.1a_0$ the state [Kr]$5s^2 4d^{10} 5p^3 4f^1$ flips below the initial ground state. At $r_c = 2.1a_0$ the state [Kr]$5s^2 4d^{10} 5p^2 4f^2$ flips below the initial ground state. At $r_c = 2.0a_0$ the state [Kr]$5s^1 4d^{10} 4f^5$ flips below the initial ground state. At $r_c = 2.0a_0$ the state [Kr]$4d^{10} 5p^1 4f^5$ flips below the initial ground state. At $r_c = 1.9a_0$ the state [Kr]$4d^9 4f^7$ flips below the initial ground state. At $r_c = 1.8a_0$ the state [Kr]$4d^8 4f^8$ flips below the initial ground state. At $r_c = 1.8a_0$ the state [Ar]$4s^2 3d^{10} 4p^5 4d^{10} 4f^7$ flips below the initial ground state. The ionization energy of the unconfined atom is $\Delta E_0 = 9.98$ eV. The studied configurations, atomic radii and excitation energies in the unconfined calculations are as follows:

configuration	r_ϵ	r_ρ	$r_{\max}$	ΔE
[Kr]$5s^2 4d^{10} 5p^4$	5.42	4.65	2.19	0.00
[Kr]$5s^2 4d^{10} 5p^3 6s^1$	10.46	4.71	5.96	6.90
[Kr]$5s^2 4d^{10} 5p^3 5d^1$	14.24	4.60	5.89	8.18
[Kr]$5s^1 4d^{10} 5p^5$	5.45	4.70	2.18	8.28
[Kr]$5s^2 4d^{10} 5p^3 4f^1$	26.44	4.33	17.38	9.13
[Kr]$5s^2 4d^{10} 5p^2 4f^2$	19.25	4.13	9.43	23.20
[Kr]$5s^2 4d^{10} 5p^1 4f^3$	16.16	5.33	5.98	40.10
[Kr]$5s^2 4d^{10} 4f^4$	14.61	6.17	4.31	57.92
[Kr]$5s^1 4d^{10} 4f^5$	13.60	6.32	3.36	85.71
[Kr]$4d^{10} 5p^1 4f^5$	13.65	6.25	3.20	95.42
[Kr]$4d^{10} 4f^6$	12.80	6.32	2.91	113.71
[Kr]$4d^9 4f^7$	12.56	6.17	2.18	179.46
[Kr]$4d^8 4f^8$	12.44	6.09	1.53	247.77
[Ar]$4s^2 3d^{10} 4p^5 4d^{10} 4f^7$	12.62	6.17	2.12	250.19

I The energies of the low lying configurations of hard-wall confined I are shown in fig. S111 for the neutral atom and in fig. S112 for the cation. The ground state of the unconfined I is [Kr]$5s^2 4d^{10} 5p^5$. At $r_c = 3.7a_0$ the ground state changes to [Kr]$5s^2 4d^{10} 5p^4 6s^1$. Furthermore, at $r_c = 3.6a_0$ we see a ground state crossing to [Kr]$5s^2 4d^{10} 5p^5$. At $r_c = 2.0a_0$ we see a ground state crossing to [Kr]$5s^2 4d^{10} 4f^5$. Moreover, at $r_c = 1.8a_0$ we see a ground state crossing to [Kr]$4d^{10} 4f^7$.

At $r_c = 2.0a_0$ the state [Kr]$5s^1 4d^{10} 4f^6$ flips below the initial ground state. At $r_c = 2.0a_0$ the state [Kr]$5s^2 4d^{10} 5p^1 4f^4$ flips below the initial ground state. At $r_c = 2.0a_0$ the state [Kr]$5s^2 4d^{10} 5p^2 4f^3$ flips below the initial ground state. At $r_c = 2.0a_0$ the state [Kr]$5s^2 4d^{10} 5p^4 4f^1$ flips below the initial ground state. At $r_c = 2.0a_0$ the state [Kr]$5s^2 4d^{10} 5p^3 4f^2$ flips below the initial ground state. At $r_c = 1.9a_0$ the state [Kr]$4d^{10} 5p^1 4f^6$ flips below the initial ground state. At $r_c = 1.8a_0$ the state [Kr]$4d^9 4f^8$ flips below the initial ground state. At $r_c = 1.8a_0$ the state [Ar]$4s^2 3d^{10} 4p^5 4d^{10} 4f^8$ flips below the initial ground state. At $r_c = 1.7a_0$ the state [Kr]$4d^8 4f^9$ flips below the initial ground state. The ionization energy of the unconfined atom is $\Delta E_0 = 11.44$ eV. The studied configurations, atomic radii and excitation energies in the unconfined calculations are as follows:

configuration	r_ϵ	r_ρ	$r_{\max}$	ΔE
[Kr]$5s^2 4d^{10} 5p^5$	5.14	4.61	2.05	0.00
[Kr]$5s^2 4d^{10} 5p^4 6s^1$	10.13	4.70	5.70	8.25
[Kr]$5s^1 4d^{10} 5p^6$	5.16	4.61	2.04	9.23
[Kr]$5s^2 4d^{10} 5p^4 5d^1$	13.96	4.55	4.77	9.58
[Kr]$5s^2 4d^{10} 5p^4 4f^1$	26.47	4.27	17.42	10.60
[Kr]$5s^2 4d^{10} 5p^3 4f^2$	19.29	4.12	9.40	26.63
[Kr]$5s^2 4d^{10} 5p^2 4f^3$	16.48	5.10	5.74	45.41
[Kr]$5s^2 4d^{10} 5p^1 4f^4$	15.09	5.81	3.62	64.75
[Kr]$5s^2 4d^{10} 4f^5$	14.05	6.08	2.80	84.12
[Kr]$5s^1 4d^{10} 4f^6$	13.25	6.16	2.39	113.85
[Kr]$4d^{10} 5p^1 4f^6$	13.33	6.08	2.28	124.24
[Kr]$4d^{10} 4f^7$	12.56	6.16	2.21	143.55
[Kr]$4d^9 4f^8$	12.46	6.09	1.50	215.78
[Kr]$4d^8 4f^9$	12.43	5.93	1.17	289.99
[Ar]$4s^2 3d^{10} 4p^5 4d^{10} 4f^8$	12.52	6.09	1.39	290.13

Xe The energies of the low lying configurations of hard-wall confined Xe are shown in fig. S113 for the neutral atom and in fig. S114 for the cation. The ground state of the unconfined Xe is [Kr]$5s^2 4d^{10} 5p^6$. At $r_c = 9.9a_0$ the ground state changes to [Kr]$5s^2 4d^{10} 5p^5 6s^1$. Furthermore, at $r_c = 9.8a_0$ we see a ground state crossing to [Kr]$5s^2 4d^{10} 5p^5 5d^1$. At $r_c = 9.6a_0$ we see a ground state crossing to [Kr]$5s^2 4d^{10} 5p^5 6s^1$. Moreover, at $r_c = 9.5a_0$ we see a ground state crossing to [Kr]$5s^2 4d^{10} 5p^6$. At $r_c = 3.6a_0$ we see a ground state crossing to [Kr]$5s^2 4d^{10} 5p^5 6s^1$. At $r_c = 3.5a_0$ we see a ground state crossing to [Kr]$5s^2 4d^{10} 5p^6$. At $r_c = 2.0a_0$ we see a ground state crossing to [Kr]$5s^2 4d^{10} 4f^6$. Finally, at $r_c = 1.7a_0$ the ground state becomes [Kr]$4d^{10} 4f^8$.

At $r_c = 2.0a_0$ the state [Kr]$5s^1 4d^{10} 4f^7$ flips below the initial ground state. At $r_c = 2.0a_0$ the state [Kr]$5s^2 4d^{10} 5p^1 4f^5$ flips below the initial ground state. At $r_c = 2.0a_0$ the state [Kr]$5s^2 4d^{10} 5p^2 4f^4$ flips below the initial ground state. At $r_c = 2.0a_0$ the state [Kr]$5s^2 4d^{10} 5p^5 4f^1$ flips below the initial ground state. At $r_c = 2.0a_0$ the state [Kr]$5s^2 4d^{10} 5p^4 4f^2$ flips below the initial ground state. At $r_c = 1.9a_0$ the state [Kr]$4d^{10} 5p^1 4f^7$ flips below the initial ground state. At $r_c = 1.8a_0$ the state [Kr]$4d^9 4f^9$ flips below the initial ground state. At $r_c = 1.7a_0$ the state [Ar]$4s^2 3d^{10} 4p^5 4d^{10} 4f^9$ flips below the initial ground state. The ionization energy of the unconfined atom is $\Delta E_0 = 12.91$ eV. The studied configurations, atomic radii and excitation energies in the unconfined calculations are as follows:

configuration	r_ϵ	r_ρ	$r_{\max}$	ΔE
[Kr]$5s^2 4d^{10} 5p^6$	4.90	4.50	1.94	0.00
[Kr]$5s^2 4d^{10} 5p^5 6s^1$	9.84	4.72	5.48	9.62
[Kr]$5s^2 4d^{10} 5p^5 5d^1$	13.77	4.50	3.22	10.99
[Kr]$5s^2 4d^{10} 5p^5 4f^1$	26.50	4.17	17.45	12.07
[Kr]$5s^2 4d^{10} 5p^4 4f^2$	19.46	4.12	9.42	29.96
[Kr]$5s^2 4d^{10} 5p^2 4f^4$	15.84	5.45	1.86	70.36
[Kr]$5s^2 4d^{10} 5p^1 4f^5$	14.79	5.75	1.86	90.67
[Kr]$5s^2 4d^{10} 4f^6$	13.86	5.93	1.67	110.89
[Kr]$5s^1 4d^{10} 4f^7$	13.14	6.04	1.67	142.04
[Kr]$4d^{10} 5p^1 4f^7$	13.23	5.93	1.86	153.03
[Kr]$4d^{10} 4f^8$	12.47	6.04	1.47	173.10
[Kr]$4d^9 4f^9$	12.46	5.93	1.12	251.38
[Ar]$4s^2 3d^{10} 4p^5 4d^{10} 4f^9$	12.53	5.93	1.09	329.32

4 Spin-polarized analysis

We present a detailed analysis of the evolution of the ground state as a function of confinement of the spin-polarized H–Xe atoms. The analysis is carried out one atom at a time. We also detail any flips between low-lying configurations and the unconfined ground state configuration. Plots are also included for all calculations, and as discussed in the main text, the energies are shown relative to the ground state of the unconfined atom

$$\Delta E_i(r_c) = E_i(r_c) - E_0^{\text{unconfined}}. \tag{2}$$

Furthermore, we provide some information on the various electronic configurations in the unconfined atom. We provide two estimates for the van der Waals radius in units of a_0: r_ϵ, and r_ρ. r_ϵ has been computed to the threshold $\epsilon = 7.341668 \times 10^{-2}$ and r_ρ to the threshold $\epsilon = 10^{-3}$ for each configuration. We also provide the location density maximum of the valence orbital in units of a_0, as a third metric for the atomic size. The excitation energy relative to the ground state, ΔE, is also given in units of eV. The ionization energy computed with the ΔSCF approach provides a point of reference for the excitation energies, as config-

urations with excitation energies smaller than the ionization potential are bound with respect to ionization—even if some such configurations have extremely diffuse orbitals. The ionization energy is marked in the tables by a horizontal line.

4.1 PW92

H The energies of the low lying configurations of hard-wall confined H are shown in fig. S115 for the neutral atom. The ground state of the unconfined H is $1s^1$. We do not observe any ground state crossing for H in the considered confinement radii.

The ionization energy of the unconfined atom is $\Delta E_0 = 13.03$ eV. The studied configurations, atomic radii and excitation energies in the unconfined calculations are as follows:

configuration	r_ϵ	r_ρ	$r_{\max}$	ΔE
$1s^1$	3.08	3.06	1.02	0.00
$2p^1$	8.67	3.94	4.11	9.65
$3d^1$	16.01	undefined	9.19	11.45

He The energies of the low lying configurations of hard-wall confined He are shown in fig. S116 for the neutral atom and in fig. S117 for the cation. The ground state of the unconfined He is $1s^2$. We do not observe any ground state crossing for He in the considered confinement radii.

The ionization energy of the unconfined atom is $\Delta E_0 = 24.29$ eV. The studied configurations, atomic radii and excitation energies in the unconfined calculations are as follows:

configuration	r_ϵ	r_ρ	$r_{\max}$	ΔE
$1s^2$	2.28	2.68	0.57	0.00
$1s^1 2p^1$	8.02	4.02	3.56	20.53
$1s^1 3d^1$	15.95	2.10	9.13	22.71
$2p^2$	6.20	4.51	2.30	57.22

Li The energies of the low lying configurations of hard-wall confined Li are shown in fig. S118 for the neutral atom and in fig. S119 for the cation. The ground state of the unconfined Li is [He]$2s^1$. At $r_c = 3.1a_0$ the ground state changes to [He]$2p^1$.

The ionization energy of the unconfined atom is $\Delta E_0 = 5.47$ eV. The studied configurations, atomic radii and excitation energies in the unconfined calculations are as follows:

configuration	r_ϵ	r_ρ	$r_{\max}$	ΔE
[He]$2s^1$	6.40	4.17	3.07	0.00
[He]$2p^1$	8.17	3.98	3.69	1.82
[He]$3d^1$	15.98	1.91	9.16	3.89
$1s^1 2p^2$	5.96	4.39	2.11	58.74

Be The energies of the low lying configurations of hard-wall confined Be are shown in fig. S120 for the neutral atom and in fig. S121 for the cation. The ground state of the unconfined Be is [He]$2s^2$. At $r_c = 2.6a_0$ the ground state changes to [He]$2s^1 2p^1$. Furthermore, at $r_c = 2.3a_0$ we see a ground state crossing to [He]$2p^2$.

At $r_c = 1.2a_0$ the state [He]$2p^1 3d^1$ flips below the initial ground state. The ionization energy of the unconfined atom is $\Delta E_0 = 9.03$ eV. The studied configurations, atomic radii and excitation energies in the unconfined calculations are as follows:

configuration	r_ϵ	r_ρ	$r_{\max}$	ΔE
[He]$2s^2$	5.09	4.16	2.04	0.00
[He]$2s^1 2p^1$	5.57	4.22	2.16	2.47
[He]$2p^2$	5.98	4.39	2.13	6.10
[He]$2p^1 3d^1$	14.46	3.83	7.58	11.11

B The energies of the low lying configurations of hard-wall confined B are shown in fig. S122 for the neutral atom and in fig. S123 for the cation. The ground state of the unconfined B is [He]$2s^2 2p^1$. At $r_c = 2.2a_0$ the ground state changes to [He]$2s^1 2p^2$. Furthermore, at $r_c = 1.8a_0$ we see a ground state crossing to [He]$2p^3$.

The ionization energy of the unconfined atom is $\Delta E_0 = 8.58$ eV. The studied configurations, atomic radii and excitation energies in the unconfined calculations are as follows:

configuration	r_ϵ	r_ρ	$r_{\max}$	ΔE
[He]$2s^2 2p^1$	4.58	3.94	1.54	0.00
[He]$2s^1 2p^2$	4.67	3.98	1.52	3.22
[He]$2s^2 3s^1$	9.84	3.83	5.56	5.17
[He]$2p^3$	4.86	4.08	1.51	9.02

C The energies of the low lying configurations of hard-wall confined C are shown in fig. S124 for the neutral atom and in fig. S125

for the cation. The ground state of the unconfined C is [He]$2s^22p^2$. At $r_c = 1.8a_0$ the ground state changes to [He]$2s^12p^3$. Furthermore, at $r_c = 1.3a_0$ we see a ground state crossing to [He]$2p^4$.

The ionization energy of the unconfined atom is $\Delta E_0 = 11.76$ eV. The studied configurations, atomic radii and excitation energies in the unconfined calculations are as follows:

configuration	r_ϵ	r_ρ	$r_{\max}$	ΔE
[He]$2s^22p^2$	4.00	3.71	1.23	0.00
[He]$2s^12p^3$	4.04	3.78	1.21	4.03
[He]$2s^22p^13s^1$	8.77	4.25	4.76	7.88
[He]$2p^4$	4.41	3.89	1.24	16.37

N The energies of the low lying configurations of hard-wall confined N are shown in fig. S126 for the neutral atom and in fig. S127 for the cation. The ground state of the unconfined N is [He]$2s^22p^3$. At $r_c = 1.3a_0$ the ground state changes to [He]$2s^12p^4$. Furthermore, at $r_c = 1.2a_0$ we see a ground state crossing to [He]$2p^5$.

The ionization energy of the unconfined atom is $\Delta E_0 = 14.99$ eV. The studied configurations, atomic radii and excitation energies in the unconfined calculations are as follows:

configuration	r_ϵ	r_ρ	$r_{\max}$	ΔE
[He]$2s^22p^3$	3.55	3.49	1.04	0.00
[He]$2s^22p^23s^1$	8.05	4.39	4.24	10.71
[He]$2s^12p^4$	3.85	3.64	1.02	10.86
[He]$2p^5$	3.88	3.64	0.98	25.06

O The energies of the low lying configurations of hard-wall confined O are shown in fig. S128 for the neutral atom and in fig. S129 for the cation. The ground state of the unconfined O is [He]$2s^22p^4$. At $r_c = 1.1a_0$ the ground state changes to [He]$2p^6$.

At $r_c = 1.1a_0$ the state [He]$2s^12p^5$ flips below the initial ground state. The ionization energy of the unconfined atom is $\Delta E_0 = 13.90$ eV. The studied configurations, atomic radii and excitation energies in the unconfined calculations are as follows:

configuration	r_ϵ	r_ρ	$r_{\max}$	ΔE
[He]$2s^22p^4$	3.33	3.33	0.88	0.00
[He]$2s^22p^33s^1$	7.52	4.39	3.86	9.26
[He]$2s^22p^33d^1$	15.58	2.96	8.76	12.28
[He]$2s^12p^5$	3.45	3.41	0.87	14.38
[He]$2p^6$	3.48	3.49	0.82	30.60

F The energies of the low lying configurations of hard-wall confined F are shown in fig. S130 for the neutral atom and in fig. S131 for the cation. The ground state of the unconfined F is [He]$2s^22p^5$. At $r_c = 1.0a_0$ the ground state changes to [He]$2s^12p^6$.

The ionization energy of the unconfined atom is $\Delta E_0 = 18.06$ eV. The studied configurations, atomic radii and excitation energies in the unconfined calculations are as follows:

configuration	r_ϵ	r_ρ	$r_{\max}$	ΔE
[He]$2s^22p^5$	3.06	3.24	0.77	0.00
[He]$2s^22p^43s^1$	7.14	4.35	3.59	13.15
[He]$2s^22p^43d^1$	15.69	2.87	8.87	16.45
[He]$2s^12p^6$	3.13	3.24	0.77	18.22
[He]$2s^12p^53d^1$	15.67	2.96	8.84	34.98

Ne The energies of the low lying configurations of hard-wall confined Ne are shown in fig. S132 for the neutral atom and in fig. S133 for the cation. The ground state of the unconfined Ne is [He]$2s^22p^6$. We do not observe any ground state crossing for Ne in the considered confinement radii.

The ionization energy of the unconfined atom is $\Delta E_0 = 22.18$ eV. The studied configurations, atomic radii and excitation energies in the unconfined calculations are as follows:

configuration	r_ϵ	r_ρ	$r_{\max}$	ΔE
[He]$2s^22p^6$	2.83	3.06	0.68	0.00
[He]$2s^22p^53s^1$	6.85	4.28	3.38	17.03
[He]$2s^22p^53d^1$	15.77	2.78	8.95	20.58
[He]$2s^12p^63d^1$	15.76	2.78	8.93	43.30

Na The energies of the low lying configurations of hard-wall confined Na are shown in fig. S134 for the neutral atom and in fig. S135 for the cation. The ground state of the unconfined Na is [Ne]$3s^1$. At $r_c = 2.0a_0$ the ground state changes to [Ne]$3d^1$.

At $r_c = 1.6a_0$ the state [Ne]$3p^1$ flips below the initial ground state. At $r_c = 1.1a_0$ the state

[Ne]$4f^1$ flips below the initial ground state. The ionization energy of the unconfined atom is $\Delta E_0 = 5.37$ eV. The studied configurations, atomic radii and excitation energies in the unconfined calculations are as follows:

configuration	r_ϵ	r_ρ	$r_{\max}$	ΔE
[Ne]$3s^1$	6.60	4.23	3.22	0.00
[Ne]$3p^1$	9.36	3.64	4.68	2.18
[Ne]$3d^1$	15.83	2.68	9.01	3.77
[Ne]$4f^1$	24.63	2.59	16.10	4.44

Mg The energies of the low lying configurations of hard-wall confined Mg are shown in fig. S136 for the neutral atom and in fig. S137 for the cation. The ground state of the unconfined Mg is [Ne]$3s^2$. At $r_c = 1.8a_0$ the ground state changes to [Ne]$3d^2$.

At $r_c = 1.8a_0$ the state [Ne]$3s^1 3d^1$ flips below the initial ground state. At $r_c = 1.7a_0$ the state [Ne]$3p^1 3d^1$ flips below the initial ground state. At $r_c = 1.5a_0$ the state [Ne]$3s^1 3p^1$ flips below the initial ground state. At $r_c = 1.5a_0$ the state [Ne]$3p^2$ flips below the initial ground state. At $r_c = 1.3a_0$ the state [Ne]$3d^1 4f^1$ flips below the initial ground state. The ionization energy of the unconfined atom is $\Delta E_0 = 7.73$ eV. The studied configurations, atomic radii and excitation energies in the unconfined calculations are as follows:

configuration	r_ϵ	r_ρ	$r_{\max}$	ΔE
[Ne]$3s^2$	5.86	4.59	2.52	0.00
[Ne]$3s^1 3p^1$	6.95	4.78	3.23	2.80
[Ne]$3s^1 3d^1$	13.80	4.16	6.81	5.85
[Ne]$3p^2$	7.31	5.17	3.15	6.60
[Ne]$3p^1 3d^1$	12.28	4.89	5.67	10.01
[Ne]$3d^2$	11.36	5.33	5.05	13.92
[Ne]$3d^1 4f^1$	21.38	4.02	12.26	15.71

Al The energies of the low lying configurations of hard-wall confined Al are shown in fig. S138 for the neutral atom and in fig. S139 for the cation. The ground state of the unconfined Al is [Ne]$3s^2 3p^1$. At $r_c = 1.9a_0$ the ground state changes to [Ne]$3s^2 3d^1$. Furthermore, at $r_c = 1.7a_0$ we see a ground state crossing to [Ne]$3s^1 3d^2$. At $r_c = 1.6a_0$ we see a ground state crossing to [Ne]$3d^3$.

At $r_c = 1.7a_0$ the state [Ne]$3p^1 3d^2$ flips below the initial ground state. At $r_c = 1.4a_0$ the state [Ne]$3s^1 3p^2$ flips below the initial ground state. The ionization energy of the unconfined atom is $\Delta E_0 = 6.00$ eV. The studied configurations, atomic radii and excitation energies in the unconfined calculations are as follows:

configuration	r_ϵ	r_ρ	$r_{\max}$	ΔE
[Ne]$3s^2 3p^1$	6.00	4.67	2.63	0.00
[Ne]$3s^2 4s^1$	11.22	4.11	6.62	3.04
[Ne]$3s^1 3p^2$	6.08	4.78	2.51	3.61
[Ne]$3s^2 3d^1$	13.74	4.21	6.65	4.10
[Ne]$3s^1 3d^2$	10.41	5.33	4.00	14.32
[Ne]$3p^1 3d^2$	9.91	5.50	3.77	20.31
[Ne]$3d^3$	9.28	5.90	3.48	26.80

Si The energies of the low lying configurations of hard-wall confined Si are shown in fig. S140 for the neutral atom and in fig. S141 for the cation. The ground state of the unconfined Si is [Ne]$3s^2 3p^2$. At $r_c = 1.7a_0$ the ground state changes to [Ne]$3s^2 3d^2$. Furthermore, at $r_c = 1.6a_0$ we see a ground state crossing to [Ne]$3s^1 3d^3$. At $r_c = 1.5a_0$ we see a ground state crossing to [Ne]$3d^4$.

At $r_c = 1.7a_0$ the state [Ne]$3s^2 3p^1 3d^1$ flips below the initial ground state. At $r_c = 1.6a_0$ the state [Ne]$3p^1 3d^3$ flips below the initial ground state. At $r_c = 1.4a_0$ the state [Ne]$3s^1 3p^3$ flips below the initial ground state. The ionization energy of the unconfined atom is $\Delta E_0 = 8.27$ eV. The studied configurations, atomic radii and excitation energies in the unconfined calculations are as follows:

configuration	r_ϵ	r_ρ	$r_{\max}$	ΔE
[Ne]$3s^2 3p^2$	5.41	4.51	2.14	0.00
[Ne]$3s^1 3p^3$	5.38	4.51	2.08	4.29
[Ne]$3s^2 3p^1 4s^1$	10.00	4.51	5.71	4.88
[Ne]$3s^2 3p^1 3d^1$	12.63	4.39	5.01	6.15
[Ne]$3s^2 3d^2$	10.22	5.17	3.64	13.61
[Ne]$3s^1 3d^3$	8.71	5.50	2.89	27.81
[Ne]$3p^1 3d^3$	8.45	5.50	2.81	35.57
[Ne]$3d^4$	8.03	5.50	2.66	44.75

P The energies of the low lying configurations of hard-wall confined P are shown in fig. S142 for the neutral atom and in fig. S143 for the cation. The ground state of the unconfined P is [Ne]$3s^2 3p^3$. At $r_c = 1.5a_0$ the ground state changes to [Ne]$3s^1 3d^4$. Furthermore, at $r_c = 1.4a_0$ we see a ground state crossing to

[Ne]$3d^5$.

At $r_c = 1.5a_0$ the state [Ne]$3p^1 3d^4$ flips below the initial ground state. At $r_c = 1.5a_0$ the state [Ne]$3s^2 3p^2 3d^1$ flips below the initial ground state. At $r_c = 1.5a_0$ the state [Ne]$3s^2 3p^1 3d^2$ flips below the initial ground state. At $r_c = 1.2a_0$ the state [Ne]$3s^1 3p^4$ flips below the initial ground state. The ionization energy of the unconfined atom is $\Delta E_0 = 10.53$ eV. The studied configurations, atomic radii and excitation energies in the unconfined calculations are as follows:

configuration	r_ϵ	r_ρ	$r_{\max}$	ΔE
[Ne]$3s^2 3p^3$	4.88	4.25	1.82	0.00
[Ne]$3s^2 3p^2 4s^1$	9.23	4.67	5.13	6.80
[Ne]$3s^1 3p^4$	5.07	4.35	1.85	8.14
[Ne]$3s^2 3p^2 3d^1$	12.30	4.28	3.98	8.30
[Ne]$3s^2 3p^1 3d^2$	9.58	5.02	2.92	18.19
[Ne]$3s^1 3d^4$	7.63	5.33	2.27	46.80
[Ne]$3p^1 3d^4$	7.47	5.33	2.23	56.32
[Ne]$3d^5$	7.16	5.33	2.14	68.28

S The energies of the low lying configurations of hard-wall confined S are shown in fig. S144 for the neutral atom and in fig. S145 for the cation. The ground state of the unconfined S is [Ne]$3s^2 3p^4$. At $r_c = 1.5a_0$ the ground state changes to [Ne]$3s^2 3p^3 3d^1$. Furthermore, at $r_c = 1.4a_0$ we see a ground state crossing to [Ne]$3s^1 3d^5$. At $r_c = 1.2a_0$ we see a ground state crossing to [Ne]$3d^6$.

At $r_c = 1.5a_0$ the state [Ne]$3s^2 3p^2 3d^2$ flips below the initial ground state. At $r_c = 1.4a_0$ the state [Ne]$3p^1 3d^5$ flips below the initial ground state. At $r_c = 1.4a_0$ the state [Ne]$3s^2 3d^4$ flips below the initial ground state. At $r_c = 1.4a_0$ the state [Ne]$3s^1 3p^1 3d^4$ flips below the initial ground state. At $r_c = 1.1a_0$ the state [Ne]$3s^1 3p^5$ flips below the initial ground state. The ionization energy of the unconfined atom is $\Delta E_0 = 10.55$ eV. The studied configurations, atomic radii and excitation energies in the unconfined calculations are as follows:

configuration	r_ϵ	r_ρ	$r_{\max}$	ΔE
[Ne]$3s^2 3p^4$	4.57	4.11	1.61	0.00
[Ne]$3s^2 3p^3 4s^1$	8.66	4.67	4.71	6.54
[Ne]$3s^2 3p^3 3d^1$	12.36	4.13	3.27	8.27
[Ne]$3s^1 3p^5$	4.64	4.15	1.60	9.95
[Ne]$3s^2 3p^2 3d^2$	9.23	4.78	2.44	20.73
[Ne]$3s^2 3d^4$	7.49	5.17	2.04	47.77
[Ne]$3s^1 3p^1 3d^4$	7.22	5.02	1.95	54.99
[Ne]$3s^1 3d^5$	6.87	5.02	1.87	69.47
[Ne]$3p^1 3d^5$	6.76	5.02	1.85	80.75
[Ne]$3d^6$	7.57	5.17	2.02	98.64

Cl The energies of the low lying configurations of hard-wall confined Cl are shown in fig. S146 for the neutral atom and in fig. S147 for the cation. The ground state of the unconfined Cl is [Ne]$3s^2 3p^5$. At $r_c = 1.4a_0$ the ground state changes to [Ne]$3s^2 3p^3 3d^2$. Furthermore, at $r_c = 1.3a_0$ we see a ground state crossing to [Ne]$3s^1 3d^6$. At $r_c = 1.2a_0$ we see a ground state crossing to [Ne]$3d^7$.

At $r_c = 1.4a_0$ the state [Ne]$3s^2 3p^2 3d^3$ flips below the initial ground state. At $r_c = 1.4a_0$ the state [Ne]$3s^2 3p^4 3d^1$ flips below the initial ground state. At $r_c = 1.3a_0$ the state [Ne]$3p^1 3d^6$ flips below the initial ground state. At $r_c = 1.0a_0$ the state [Ne]$3s^1 3p^6$ flips below the initial ground state. The ionization energy of the unconfined atom is $\Delta E_0 = 13.25$ eV. The studied configurations, atomic radii and excitation energies in the unconfined calculations are as follows:

configuration	r_ϵ	r_ρ	$r_{\max}$	ΔE
[Ne]$3s^2 3p^5$	4.24	3.98	1.43	0.00
[Ne]$3s^2 3p^4 4s^1$	8.28	4.59	4.43	9.04
[Ne]$3s^2 3p^4 3d^1$	12.91	3.98	3.20	11.06
[Ne]$3s^1 3p^6$	4.27	3.98	1.42	11.79
[Ne]$3s^2 3p^3 3d^2$	9.07	4.67	2.08	23.29
[Ne]$3s^2 3p^2 3d^3$	7.82	4.89	1.88	39.30
[Ne]$3s^1 3d^6$	8.89	4.89	1.98	101.90
[Ne]$3p^1 3d^6$	8.76	4.89	1.91	115.05
[Ne]$3d^7$	6.72	4.89	1.63	135.91

Ar The energies of the low lying configurations of hard-wall confined Ar are shown in fig. S148 for the neutral atom and in fig. S149 for the cation. The ground state of the unconfined Ar is [Ne]$3s^2 3p^6$. At $r_c = 1.3a_0$ the ground state changes to [Ne]$3s^2 3p^4 3d^2$. Furthermore,

at $r_c = 1.2a_0$ we see a ground state crossing to [Ne]$3s^1 3p^2 3d^5$. At $r_c = 1.1a_0$ we see a ground state crossing to [Ne]$3d^8$.

At $r_c = 1.3a_0$ the state [Ne]$3s^2 3p^1 3d^5$ flips below the initial ground state. At $r_c = 1.3a_0$ the state [Ne]$3s^2 3p^5 3d^1$ flips below the initial ground state. At $r_c = 1.2a_0$ the state [Ne]$3p^1 3d^7$ flips below the initial ground state. At $r_c = 1.2a_0$ the state [Ne]$3s^1 3d^7$ flips below the initial ground state. At $r_c = 1.2a_0$ the state [Ne]$3s^2 3d^6$ flips below the initial ground state. At $r_c = 1.2a_0$ the state [Ne]$3s^1 3p^6 3d^1$ flips below the initial ground state. The ionization energy of the unconfined atom is $\Delta E_0 = 15.94$ eV. The studied configurations, atomic radii and excitation energies in the unconfined calculations are as follows:

configuration	r_ϵ	r_ρ	$r_{\max}$	ΔE
[Ne]$3s^2 3p^6$	3.94	3.83	1.29	0.00
[Ne]$3s^2 3p^5 4s^1$	7.99	4.59	4.21	11.56
[Ne]$3s^2 3p^5 3d^1$	13.48	3.83	5.64	13.86
[Ne]$3s^1 3p^6 3d^1$	13.32	3.83	2.75	27.94
[Ne]$3s^2 3p^4 3d^2$	9.26	4.51	1.84	29.72
[Ne]$3s^2 3p^1 3d^5$	6.50	4.78	1.50	84.31
[Ne]$3s^1 3p^2 3d^5$	6.32	4.67	1.46	92.58
[Ne]$3s^2 3d^6$	7.16	4.78	1.52	107.44
[Ne]$3s^1 3d^7$	7.14	4.89	1.54	141.90
[Ne]$3p^1 3d^7$	7.04	4.78	1.51	156.91
[Ne]$3d^8$	6.12	4.78	1.40	180.45

K The energies of the low lying configurations of hard-wall confined K are shown in fig. S150 for the neutral atom and in fig. S151 for the cation. The ground state of the unconfined K is [Ar]$4s^1$. At $r_c = 4.5a_0$ the ground state changes to [Ar]$3d^1$. Furthermore, at $r_c = 1.2a_0$ we see a ground state crossing to [Ne]$3s^2 3p^3 3d^4$. At $r_c = 1.1a_0$ we see a ground state crossing to [Ne]$3s^1 3d^8$. Moreover, at $r_c = 1.0a_0$ we see a ground state crossing to [Ne]$3d^9$.

At $r_c = 2.9a_0$ the state [Ne]$3s^2 3p^5 3d^2$ flips below the initial ground state. At $r_c = 2.5a_0$ the state [Ne]$3s^1 3p^6 3d^2$ flips below the initial ground state. At $r_c = 2.4a_0$ the state [Ne]$3s^2 3p^4 3d^3$ flips below the initial ground state. At $r_c = 1.9a_0$ the state [Ne]$3s^2 3p^2 3d^5$ flips below the initial ground state. At $r_c = 1.9a_0$ the state [Ne]$3s^1 3p^3 3d^5$ flips below the initial ground state. At $r_c = 1.5a_0$ the state [Ne]$3p^1 3d^8$ flips below the initial ground state. At $r_c = 1.4a_0$ the state [Ar]$4p^1$ flips below the initial ground state. The ionization energy of the unconfined atom is $\Delta E_0 = 4.53$ eV. The studied configurations, atomic radii and excitation energies in the unconfined calculations are as follows:

configuration	r_ϵ	r_ρ	$r_{\max}$	ΔE
[Ar]$4s^1$	7.75	4.51	4.03	0.00
[Ar]$4p^1$	10.43	3.78	5.55	1.65
[Ar]$3d^1$	13.96	3.64	6.68	2.57
[Ne]$3s^2 3p^5 3d^2$	9.55	4.45	1.65	22.18
[Ne]$3s^1 3p^6 3d^2$	9.42	4.39	1.58	38.37
[Ne]$3s^2 3p^4 3d^3$	7.73	4.67	1.46	41.29
[Ne]$3s^2 3p^3 3d^4$	6.79	4.67	1.38	59.08
[Ne]$3s^2 3p^2 3d^5$	6.31	4.59	1.34	82.14
[Ne]$3s^1 3p^3 3d^5$	6.15	4.59	1.32	90.84
[Ne]$3s^1 3d^8$	6.28	4.67	1.31	175.52
[Ne]$3p^1 3d^8$	6.21	4.67	1.29	192.42
[Ne]$3d^9$	5.70	4.59	1.23	218.59

Ca The energies of the low lying configurations of hard-wall confined Ca are shown in fig. S152 for the neutral atom and in fig. S153 for the cation. The ground state of the unconfined Ca is [Ar]$4s^2$. At $r_c = 4.9a_0$ the ground state changes to [Ar]$4s^1 3d^1$. Furthermore, at $r_c = 4.4a_0$ we see a ground state crossing to [Ar]$3d^2$. At $r_c = 1.1a_0$ we see a ground state crossing to [Ne]$3s^2 3p^3 3d^5$. Moreover, at $r_c = 1.0a_0$ we see a ground state crossing to [Ne]$3d^{10}$.

At $r_c = 3.5a_0$ the state [Ar]$3d^1 4p^1$ flips below the initial ground state. At $r_c = 3.2a_0$ the state [Ne]$3s^2 3p^5 3d^3$ flips below the initial ground state. At $r_c = 2.8a_0$ the state [Ne]$3s^2 3p^4 3d^4$ flips below the initial ground state. At $r_c = 2.8a_0$ the state [Ne]$3s^1 3p^6 3d^3$ flips below the initial ground state. At $r_c = 1.8a_0$ the state [Ne]$3s^1 3d^9$ flips below the initial ground state. At $r_c = 1.7a_0$ the state [Ne]$3p^1 3d^9$ flips below the initial ground state. At $r_c = 1.4a_0$ the state [Ar]$4s^1 4p^1$ flips below the initial ground state. The ionization energy of the unconfined atom is $\Delta E_0 = 6.22$ eV. The studied configurations, atomic radii and excitation energies in the un-

confined calculations are as follows:

configuration	r_ϵ	r_ρ	$r_{\max}$	ΔE
[Ar]$4s^2$	7.12	5.17	3.34	0.00
[Ar]$4s^1 3d^1$	7.61	4.89	3.47	1.70
[Ar]$4s^1 4p^1$	8.15	5.33	4.17	1.96
[Ar]$3d^1 4p^1$	9.06	4.89	4.58	3.93
[Ar]$3d^2$	9.86	4.39	1.48	4.36
[Ne]$3s^2 3p^5 3d^3$	7.89	4.59	1.32	27.58
[Ne]$3s^1 3p^6 3d^3$	7.79	4.51	1.29	45.84
[Ne]$3s^2 3p^4 3d^4$	6.82	4.59	1.25	49.65
[Ne]$3s^2 3p^3 3d^5$	6.18	4.51	1.21	70.07
[Ne]$3s^1 3d^9$	5.73	4.51	1.15	206.80
[Ne]$3p^1 3d^9$	5.68	4.51	1.14	225.64
[Ne]$3d^{10}$	5.38	4.45	1.10	254.49

Sc The energies of the low lying configurations of hard-wall confined Sc are shown in fig. S154 for the neutral atom and in fig. S155 for the cation. The ground state of the unconfined Sc is [Ar]$4s^2 3d^1$. At $r_c = 5.5a_0$ the ground state changes to [Ar]$4s^1 3d^2$. Furthermore, at $r_c = 4.4a_0$ we see a ground state crossing to [Ar]$3d^3$. At $r_c = 1.1a_0$ we see a ground state crossing to [Ne]$3s^2 3p^4 3d^5$. Moreover, at $r_c = 1.0a_0$ we see a ground state crossing to [Ne]$3s^2 3d^9$.

At $r_c = 3.5a_0$ the state [Ar]$3d^2 4p^1$ flips below the initial ground state. At $r_c = 3.1a_0$ the state [Ne]$3s^2 3p^5 3d^4$ flips below the initial ground state. At $r_c = 2.7a_0$ the state [Ne]$3s^1 3p^6 3d^4$ flips below the initial ground state. At $r_c = 1.9a_0$ the state [Ne]$3s^2 3p^1 3d^8$ flips below the initial ground state. At $r_c = 1.7a_0$ the state [Ne]$3s^1 3d^{10}$ flips below the initial ground state. At $r_c = 1.4a_0$ the state [Ar]$4s^1 3d^1 4p^1$ flips below the initial ground state. The ionization energy of the unconfined atom is $\Delta E_0 = 6.61$ eV. The studied configurations, atomic radii and excitation energies in the unconfined calculations are as follows:

configuration	r_ϵ	r_ρ	$r_{\max}$	ΔE
[Ar]$4s^2 3d^1$	6.70	5.17	3.12	0.00
[Ar]$4s^1 3d^2$	6.78	4.89	3.20	0.66
[Ar]$4s^1 3d^1 4p^1$	7.65	5.33	3.82	2.05
[Ar]$3d^3$	8.09	4.59	1.20	3.04
[Ar]$3d^2 4p^1$	8.43	4.89	4.19	3.22
[Ne]$3s^2 3p^5 3d^4$	6.91	4.51	1.13	29.50
[Ne]$3s^1 3p^6 3d^4$	6.83	4.45	1.12	49.84
[Ne]$3s^2 3p^4 3d^5$	6.19	4.45	1.10	54.46
[Ne]$3s^2 3p^1 3d^8$	6.07	4.45	1.07	151.15
[Ne]$3s^2 3d^9$	5.52	4.39	1.04	183.85
[Ne]$3s^1 3d^{10}$	5.34	4.35	1.03	235.47

Ti The energies of the low lying configurations of hard-wall confined Ti are shown in fig. S156 for the neutral atom and in fig. S157 for the cation. The ground state of the unconfined Ti is [Ar]$4s^1 3d^3$. At $r_c = 4.3a_0$ the ground state changes to [Ar]$3d^4$. Furthermore, at $r_c = 1.0a_0$ we see a ground state crossing to [Ne]$3s^2 3p^4 3d^6$.

At $r_c = 2.5a_0$ the state [Ne]$3s^2 3p^5 3d^5$ flips below the initial ground state. At $r_c = 2.1a_0$ the state [Ne]$3s^1 3p^6 3d^5$ flips below the initial ground state. At $r_c = 1.7a_0$ the state [Ne]$3s^2 3p^3 3d^7$ flips below the initial ground state. At $r_c = 1.3a_0$ the state [Ar]$3d^3 4p^1$ flips below the initial ground state. The ionization energy of the unconfined atom is $\Delta E_0 = 6.80$ eV. The studied configurations, atomic radii and excitation energies in the unconfined calculations are as follows:

configuration	r_ϵ	r_ρ	$r_{\max}$	ΔE
[Ar]$4s^1 3d^3$	6.29	4.78	3.00	0.00
[Ar]$4s^2 3d^2$	6.40	5.02	2.97	0.30
[Ar]$3d^4$	7.05	4.45	1.04	2.03
[Ar]$3d^3 4p^1$	8.08	4.89	3.94	2.83
[Ne]$3s^2 3p^5 3d^5$	6.24	4.39	1.01	31.63
[Ne]$3s^1 3p^6 3d^5$	6.18	4.35	1.00	54.09
[Ne]$3s^2 3p^4 3d^6$	11.93	4.18	1.06	63.29
[Ne]$3s^2 3p^3 3d^7$	8.75	4.39	1.05	95.47

V The energies of the low lying configurations of hard-wall confined V are shown in fig. S158 for the neutral atom and in fig. S159 for the cation. The ground state of the unconfined V is [Ar]$4s^1 3d^4$. At $r_c = 4.4a_0$ the ground state changes to [Ar]$3d^5$.

At $r_c = 2.3a_0$ the state [Ne]$3s^2 3p^5 3d^6$ flips

below the initial ground state. At $r_c = 2.0a_0$ the state [Ne]$3s^13p^63d^6$ flips below the initial ground state. At $r_c = 1.8a_0$ the state [Ne]$3s^23p^43d^7$ flips below the initial ground state. At $r_c = 1.2a_0$ the state [Ar]$3d^44p^1$ flips below the initial ground state. The ionization energy of the unconfined atom is $\Delta E_0 = 7.15$ eV. The studied configurations, atomic radii and excitation energies in the unconfined calculations are as follows:

configuration	r_ϵ	r_ρ	$r_{\max}$	ΔE
[Ar]$4s^13d^4$	5.94	4.59	2.85	0.00
[Ar]$4s^23d^3$	6.17	4.89	2.84	1.20
[Ar]$3d^5$	6.34	4.35	0.93	1.67
[Ar]$3d^44p^1$	7.84	4.78	3.75	3.07
[Ne]$3s^23p^53d^6$	10.97	4.17	0.95	37.75
[Ne]$3s^13p^63d^6$	10.99	4.16	0.94	62.42
[Ne]$3s^23p^43d^7$	8.13	4.31	0.94	74.19

Cr The energies of the low lying configurations of hard-wall confined Cr are shown in fig. S160 for the neutral atom and in fig. S161 for the cation. The ground state of the unconfined Cr is [Ar]$4s^13d^5$. At $r_c = 3.5a_0$ the ground state changes to [Ar]$3d^6$.

At $r_c = 2.1a_0$ the state [Ne]$3s^23p^53d^7$ flips below the initial ground state. At $r_c = 1.8a_0$ the state [Ne]$3s^13p^63d^7$ flips below the initial ground state. At $r_c = 1.7a_0$ the state [Ne]$3s^23p^43d^8$ flips below the initial ground state. At $r_c = 1.2a_0$ the state [Ar]$3d^54p^1$ flips below the initial ground state. The ionization energy of the unconfined atom is $\Delta E_0 = 7.46$ eV. The studied configurations, atomic radii and excitation energies in the unconfined calculations are as follows:

configuration	r_ϵ	r_ρ	$r_{\max}$	ΔE
[Ar]$4s^13d^5$	5.68	4.45	2.71	0.00
[Ar]$4s^23d^4$	5.97	4.78	2.74	2.05
[Ar]$3d^54p^1$	7.67	4.67	3.61	3.29
[Ar]$3d^6$	9.20	4.18	0.86	4.13
[Ne]$3s^23p^53d^7$	7.40	4.21	0.85	44.90
[Ne]$3s^13p^63d^7$	7.36	4.20	0.85	71.83
[Ne]$3s^23p^43d^8$	6.59	4.23	0.85	85.85

Mn The energies of the low lying configurations of hard-wall confined Mn are shown in fig. S162 for the neutral atom and in fig. S163 for the cation. The ground state of the unconfined Mn is [Ar]$4s^23d^5$. At $r_c = 4.8a_0$ the ground state changes to [Ar]$4s^13d^6$. Furthermore, at $r_c = 3.6a_0$ we see a ground state crossing to [Ar]$3d^7$.

At $r_c = 2.9a_0$ the state [Ar]$3d^64p^1$ flips below the initial ground state. At $r_c = 2.4a_0$ the state [Ne]$3s^23p^53d^8$ flips below the initial ground state. At $r_c = 2.2a_0$ the state [Ne]$3s^13p^63d^8$ flips below the initial ground state. At $r_c = 1.2a_0$ the state [Ar]$4s^13d^54p^1$ flips below the initial ground state. The ionization energy of the unconfined atom is $\Delta E_0 = 7.49$ eV. The studied configurations, atomic radii and excitation energies in the unconfined calculations are as follows:

configuration	r_ϵ	r_ρ	$r_{\max}$	ΔE
[Ar]$4s^23d^5$	5.81	4.67	2.65	0.00
[Ar]$4s^13d^6$	5.60	4.39	2.60	1.04
[Ar]$4s^13d^54p^1$	6.73	4.78	3.15	2.34
[Ar]$3d^7$	6.65	4.18	0.79	4.39
[Ar]$3d^64p^1$	7.57	4.59	3.50	4.54
[Ne]$3s^23p^53d^8$	6.16	4.17	0.78	49.77
[Ne]$3s^13p^63d^8$	6.12	4.16	0.78	79.05

Fe The energies of the low lying configurations of hard-wall confined Fe are shown in fig. S164 for the neutral atom and in fig. S165 for the cation. The ground state of the unconfined Fe is [Ar]$4s^23d^6$. At $r_c = 6.1a_0$ the ground state changes to [Ar]$4s^13d^7$. Furthermore, at $r_c = 3.7a_0$ we see a ground state crossing to [Ar]$3d^8$.

At $r_c = 2.9a_0$ the state [Ar]$3d^74p^1$ flips below the initial ground state. At $r_c = 2.3a_0$ the state [Ne]$3s^23p^53d^9$ flips below the initial ground state. At $r_c = 2.1a_0$ the state [Ne]$3s^13p^63d^9$ flips below the initial ground state. At $r_c = 1.2a_0$ the state [Ar]$4s^13d^64p^1$ flips below the initial ground state. The ionization energy of the unconfined atom is $\Delta E_0 = 8.05$ eV. The studied configurations, atomic radii and excitation energies in the unconfined calculations are as follows:

configuration	r_ϵ	r_ρ	$r_{\max}$	ΔE
[Ar]$4s^2 3d^6$	5.60	4.51	2.50	0.00
[Ar]$4s^1 3d^7$	5.42	4.31	2.51	0.16
[Ar]$3d^8$	5.83	4.13	0.73	2.74
[Ar]$4s^1 3d^6 4p^1$	6.61	4.78	3.06	2.82
[Ar]$3d^7 4p^1$	7.50	4.59	3.42	3.82
[Ne]$3s^2 3p^5 3d^9$	5.52	4.11	0.73	52.85
[Ne]$3s^1 3p^6 3d^9$	5.49	4.08	0.73	84.54

Co The energies of the low lying configurations of hard-wall confined Co are shown in fig. S166 for the neutral atom and in fig. S167 for the cation. The ground state of the unconfined Co is [Ar]$4s^1 3d^8$. At $r_c = 3.9a_0$ the ground state changes to [Ar]$3d^9$.

At $r_c = 1.9a_0$ the state [Ne]$3s^2 3p^5 3d^{10}$ flips below the initial ground state. At $r_c = 1.6a_0$ the state [Ne]$3s^1 3p^6 3d^{10}$ flips below the initial ground state. At $r_c = 1.1a_0$ the state [Ar]$3d^8 4p^1$ flips below the initial ground state. The ionization energy of the unconfined atom is $\Delta E_0 = 8.08$ eV. The studied configurations, atomic radii and excitation energies in the unconfined calculations are as follows:

configuration	r_ϵ	r_ρ	$r_{\max}$	ΔE
[Ar]$4s^1 3d^8$	5.27	4.22	2.44	0.00
[Ar]$4s^2 3d^7$	5.42	4.45	2.38	0.71
[Ar]$3d^9$	5.38	4.05	0.68	1.83
[Ar]$3d^8 4p^1$	7.46	4.51	3.36	3.82
[Ne]$3s^2 3p^5 3d^{10}$	5.13	4.02	0.68	56.80
[Ne]$3s^1 3p^6 3d^{10}$	5.10	4.02	0.68	90.98

Ni The energies of the low lying configurations of hard-wall confined Ni are shown in fig. S168 for the neutral atom and in fig. S169 for the cation. The ground state of the unconfined Ni is [Ar]$4s^1 3d^9$. At $r_c = 4.3a_0$ the ground state changes to [Ar]$3d^{10}$.

At $r_c = 1.4a_0$ the state [Ar]$3d^9 4f^1$ flips below the initial ground state. At $r_c = 1.2a_0$ the state [Ne]$3s^2 3p^5 3d^{10} 4f^1$ flips below the initial ground state. At $r_c = 1.0a_0$ the state [Ar]$3d^9 4p^1$ flips below the initial ground state. The ionization energy of the unconfined atom is $\Delta E_0 = 8.24$ eV. The studied configurations, atomic radii and excitation energies in the unconfined calculations are as follows:

configuration	r_ϵ	r_ρ	$r_{\max}$	ΔE
[Ar]$4s^1 3d^9$	5.14	4.16	2.37	0.00
[Ar]$3d^{10}$	5.09	3.98	0.65	1.10
[Ar]$4s^2 3d^8$	5.27	4.35	2.28	1.56
[Ar]$3d^9 4p^1$	7.43	4.45	3.32	3.97
[Ar]$3d^9 4f^1$	24.56	3.41	16.03	7.30
[Ne]$3s^2 3p^5 3d^{10} 4f^1$	24.57	3.41	16.04	67.80

Cu The energies of the low lying configurations of hard-wall confined Cu are shown in fig. S170 for the neutral atom and in fig. S171 for the cation. The ground state of the unconfined Cu is [Ar]$4s^1 3d^{10}$. At $r_c = 1.4a_0$ the ground state changes to [Ar]$3d^{10} 4f^1$.

At $r_c = 1.1a_0$ the state [Ar]$3d^{10} 4d^1$ flips below the initial ground state. At $r_c = 1.0a_0$ the state [Ar]$3d^{10} 4p^1$ flips below the initial ground state. The ionization energy of the unconfined atom is $\Delta E_0 = 8.38$ eV. The studied configurations, atomic radii and excitation energies in the unconfined calculations are as follows:

configuration	r_ϵ	r_ρ	$r_{\max}$	ΔE
[Ar]$4s^1 3d^{10}$	5.03	4.11	2.31	0.00
[Ar]$4s^2 3d^9$	5.15	4.28	2.19	2.40
[Ar]$3d^{10} 4p^1$	7.41	4.39	3.28	4.11
[Ar]$3d^{10} 4d^1$	15.64	3.41	8.82	6.76
[Ar]$3d^{10} 4f^1$	24.57	3.41	16.04	7.45

Zn The energies of the low lying configurations of hard-wall confined Zn are shown in fig. S172 for the neutral atom and in fig. S173 for the cation. The ground state of the unconfined Zn is [Ar]$4s^2 3d^{10}$. At $r_c = 1.3a_0$ the ground state changes to [Ar]$3d^{10} 4f^2$.

At $r_c = 1.3a_0$ the state [Ar]$4s^1 3d^{10} 4f^1$ flips below the initial ground state. At $r_c = 1.2a_0$ the state [Ar]$3d^{10} 4d^1 4f^1$ flips below the initial ground state. At $r_c = 1.2a_0$ the state [Ar]$3d^{10} 4p^1 4f^1$ flips below the initial ground state. At $r_c = 1.1a_0$ the state [Ar]$4s^1 3d^{10} 4d^1$ flips below the initial ground state. At $r_c = 1.0a_0$ the state [Ar]$4s^1 3d^{10} 4p^1$ flips below the initial ground state. At $r_c = 1.0a_0$ the state [Ar]$3d^{10} 4p^2$ flips below the initial ground state. The ionization energy of the unconfined atom is $\Delta E_0 = 9.69$ eV. The studied configurations, atomic radii and excitation energies in the unconfined calculations are as follows:

configuration	r_ϵ	r_ρ	$r_{\max}$	ΔE
[Ar]$4s^2 3d^{10}$	5.03	4.21	2.12	0.00
[Ar]$4s^1 3d^{10} 4p^1$	6.34	4.51	2.81	4.31
[Ar]$4s^1 3d^{10} 4d^1$	14.83	3.83	7.98	7.97
[Ar]$4s^1 3d^{10} 4f^1$	24.50	3.71	15.96	8.76
[Ar]$3d^{10} 4p^2$	6.65	4.89	2.72	9.67
[Ar]$3d^{10} 4p^1 4f^1$	24.23	4.02	15.66	14.92
[Ar]$3d^{10} 4d^1 4f^1$	20.75	3.98	11.50	21.12
[Ar]$3d^{10} 4f^2$	17.82	2.96	9.34	23.23

Ga The energies of the low lying configurations of hard-wall confined Ga are shown in fig. S174 for the neutral atom and in fig. S175 for the cation. The ground state of the unconfined Ga is [Ar]$4s^2 3d^{10} 4p^1$. At $r_c = 1.3a_0$ the ground state changes to [Ar]$4s^1 3d^{10} 4f^2$. Furthermore, at $r_c = 1.2a_0$ we see a ground state crossing to [Ar]$3d^{10} 4f^3$.

At $r_c = 1.3a_0$ the state [Ar]$3d^{10} 4p^1 4f^2$ flips below the initial ground state. At $r_c = 1.3a_0$ the state [Ar]$4s^2 3d^{10} 4f^1$ flips below the initial ground state. At $r_c = 1.2a_0$ the state [Ar]$3d^{10} 4d^1 4f^2$ flips below the initial ground state. At $r_c = 1.1a_0$ the state [Ar]$4s^2 3d^{10} 4d^1$ flips below the initial ground state. At $r_c = 1.0a_0$ the state [Ar]$4s^1 3d^{10} 4p^2$ flips below the initial ground state. The ionization energy of the unconfined atom is $\Delta E_0 = 6.06$ eV. The studied configurations, atomic radii and excitation energies in the unconfined calculations are as follows:

configuration	r_ϵ	r_ρ	$r_{\max}$	ΔE
[Ar]$4s^2 3d^{10} 4p^1$	5.83	4.51	2.53	0.00
[Ar]$4s^2 3d^{10} 5s^1$	10.90	4.02	6.36	3.01
[Ar]$4s^2 3d^{10} 4d^1$	14.63	3.98	7.74	4.31
[Ar]$4s^1 3d^{10} 4p^2$	5.92	4.67	2.42	4.98
[Ar]$4s^2 3d^{10} 4f^1$	24.51	3.83	15.97	5.12
[Ar]$3d^{10} 4p^3$	6.00	4.78	2.37	11.62
[Ar]$4s^1 3d^{10} 4f^2$	17.72	3.49	9.18	21.68
[Ar]$3d^{10} 4p^1 4f^2$	17.59	3.83	8.98	29.72
[Ar]$3d^{10} 4d^1 4f^2$	16.62	5.17	7.63	39.32
[Ar]$3d^{10} 4f^3$	14.67	6.11	6.54	43.52

Ge The energies of the low lying configurations of hard-wall confined Ge are shown in fig. S176 for the neutral atom and in fig. S177 for the cation. The ground state of the unconfined Ge is [Ar]$4s^2 3d^{10} 4p^2$. At $r_c = 1.3a_0$ the ground state changes to [Ar]$4s^2 3d^{10} 4p^1 4f^1$. Furthermore, at $r_c = 1.2a_0$ we see a ground state crossing to [Ar]$3d^{10} 4f^4$.

At $r_c = 1.3a_0$ the state [Ar]$4s^1 3d^{10} 4f^3$ flips below the initial ground state. At $r_c = 1.2a_0$ the state [Ar]$3d^{10} 4d^1 4f^3$ flips below the initial ground state. At $r_c = 1.2a_0$ the state [Ar]$3d^{10} 4p^1 4f^3$ flips below the initial ground state. At $r_c = 1.0a_0$ the state [Ar]$4s^2 3d^{10} 4p^1 4d^1$ flips below the initial ground state. The ionization energy of the unconfined atom is $\Delta E_0 = 8.06$ eV. The studied configurations, atomic radii and excitation energies in the unconfined calculations are as follows:

configuration	r_ϵ	r_ρ	$r_{\max}$	ΔE
[Ar]$4s^2 3d^{10} 4p^2$	5.50	4.51	2.21	0.00
[Ar]$4s^2 3d^{10} 4p^1 5s^1$	9.94	4.59	5.66	4.64
[Ar]$4s^1 3d^{10} 4p^3$	5.46	4.59	2.15	5.56
[Ar]$4s^2 3d^{10} 4p^1 4d^1$	13.47	4.31	6.34	6.12
[Ar]$4s^2 3d^{10} 4p^1 4f^1$	24.42	3.98	15.87	7.12
[Ar]$4s^1 3d^{10} 4f^3$	14.62	6.11	6.38	43.28
[Ar]$3d^{10} 4p^1 4f^3$	14.56	6.11	6.24	53.06
[Ar]$3d^{10} 4d^1 4f^3$	14.17	6.34	5.62	65.93
[Ar]$3d^{10} 4f^4$	12.83	6.84	5.00	72.64

As The energies of the low lying configurations of hard-wall confined As are shown in fig. S178 for the neutral atom and in fig. S179 for the cation. The ground state of the unconfined As is [Ar]$4s^2 3d^{10} 4p^3$. At $r_c = 1.2a_0$ the ground state changes to [Ar]$3d^{10} 4f^5$.

At $r_c = 1.2a_0$ the state [Ar]$3d^{10} 4d^1 4f^4$ flips below the initial ground state. At $r_c = 1.2a_0$ the state [Ar]$4s^1 3d^{10} 4f^4$ flips below the initial ground state. At $r_c = 1.2a_0$ the state [Ar]$4s^2 3d^{10} 4f^3$ flips below the initial ground state. At $r_c = 1.2a_0$ the state [Ar]$4s^2 3d^{10} 4p^2 4f^1$ flips below the initial ground state. At $r_c = 1.0a_0$ the state [Ar]$4s^2 3d^{10} 4p^2 4d^1$ flips below the initial ground state. The ionization energy of the unconfined atom is $\Delta E_0 = 9.97$ eV. The studied configurations, atomic radii and excitation energies in the unconfined calculations are as follows:

configuration	r_ϵ	r_ρ	$r_{\max}$	ΔE
$[Ar]4s^2 3d^{10} 4p^3$	5.12	4.39	1.98	0.00
$[Ar]4s^2 3d^{10} 4p^2 5s^1$	9.32	4.78	5.20	6.27
$[Ar]4s^2 3d^{10} 4p^2 4d^1$	12.86	4.39	5.37	7.92
$[Ar]4s^1 3d^{10} 4p^4$	5.27	4.51	1.98	8.82
$[Ar]4s^2 3d^{10} 4p^2 4f^1$	24.44	3.98	15.89	9.04
$[Ar]4s^2 3d^{10} 4f^3$	14.62	6.11	6.36	41.91
$[Ar]4s^1 3d^{10} 4f^4$	12.81	6.84	4.84	74.23
$[Ar]3d^{10} 4d^1 4f^4$	12.60	6.58	4.41	101.71
$[Ar]3d^{10} 4f^5$	11.60	6.84	4.03	111.21

Se The energies of the low lying configurations of hard-wall confined Se are shown in fig. S180 for the neutral atom and in fig. S181 for the cation. The ground state of the unconfined Se is $[Ar]4s^2 3d^{10} 4p^4$. At $r_c = 1.2a_0$ the ground state changes to $[Ar]4s^2 3d^{10} 4f^4$. Furthermore, at $r_c = 1.1a_0$ we see a ground state crossing to $[Ar]3d^{10} 4f^6$.

At $r_c = 1.2a_0$ the state $[Ar]4s^1 3d^{10} 4f^5$ flips below the initial ground state. At $r_c = 1.2a_0$ the state $[Ar]4s^2 3d^{10} 4p^1 4f^3$ flips below the initial ground state. At $r_c = 1.2a_0$ the state $[Ar]4s^2 3d^{10} 4p^3 4f^1$ flips below the initial ground state. At $r_c = 1.1a_0$ the state $[Ar]3d^{10} 4d^1 4f^5$ flips below the initial ground state. At $r_c = 1.0a_0$ the state $[Ar]4s^2 3d^{10} 4p^3 4d^1$ flips below the initial ground state. The ionization energy of the unconfined atom is $\Delta E_0 = 9.93$ eV. The studied configurations, atomic radii and excitation energies in the unconfined calculations are as follows:

configuration	r_ϵ	r_ρ	$r_{\max}$	ΔE
$[Ar]4s^2 3d^{10} 4p^4$	4.90	4.31	1.81	0.00
$[Ar]4s^2 3d^{10} 4p^3 5s^1$	8.86	4.78	4.86	6.00
$[Ar]4s^2 3d^{10} 4p^3 4d^1$	12.52	4.31	4.56	7.79
$[Ar]4s^2 3d^{10} 4p^3 4f^1$	24.47	3.94	15.93	9.00
$[Ar]4s^1 3d^{10} 4p^5$	4.94	4.35	1.80	10.20
$[Ar]4s^2 3d^{10} 4p^1 4f^3$	14.59	6.11	6.23	47.85
$[Ar]4s^2 3d^{10} 4f^4$	12.82	6.84	4.82	73.11
$[Ar]4s^1 3d^{10} 4f^5$	11.62	6.84	3.88	113.15
$[Ar]3d^{10} 4d^1 4f^5$	11.51	6.58	3.62	145.33
$[Ar]3d^{10} 4f^6$	10.72	6.58	3.37	157.80

Br The energies of the low lying configurations of hard-wall confined Br are shown in fig. S182 for the neutral atom and in fig. S183 for the cation. The ground state of the unconfined Br is $[Ar]4s^2 3d^{10} 4p^5$. At $r_c = 1.2a_0$ the ground state changes to $[Ar]4s^2 3d^{10} 4p^3 4f^2$. Furthermore, at $r_c = 1.1a_0$ we see a ground state crossing to $[Ar]3d^{10} 4f^7$.

At $r_c = 1.2a_0$ the state $[Ar]4s^2 3d^{10} 4p^2 4f^3$ flips below the initial ground state. At $r_c = 1.2a_0$ the state $[Ar]4s^2 3d^{10} 4p^4 4f^1$ flips below the initial ground state. At $r_c = 1.1a_0$ the state $[Ar]4s^1 3d^{10} 4f^6$ flips below the initial ground state. At $r_c = 1.1a_0$ the state $[Ar]3d^{10} 4p^1 4f^6$ flips below the initial ground state. At $r_c = 1.0a_0$ the state $[Ar]4s^2 3d^{10} 4p^4 4d^1$ flips below the initial ground state. The ionization energy of the unconfined atom is $\Delta E_0 = 12.14$ eV. The studied configurations, atomic radii and excitation energies in the unconfined calculations are as follows:

configuration	r_ϵ	r_ρ	$r_{\max}$	ΔE
$[Ar]4s^2 3d^{10} 4p^5$	4.63	4.17	1.67	0.00
$[Ar]4s^2 3d^{10} 4p^4 5s^1$	8.55	4.78	4.63	8.05
$[Ar]4s^2 3d^{10} 4p^4 4d^1$	12.65	4.21	4.42	10.00
$[Ar]4s^2 3d^{10} 4p^4 4f^1$	24.51	3.89	15.96	11.20
$[Ar]4s^1 3d^{10} 4p^6$	4.64	4.20	1.66	11.58
$[Ar]4s^2 3d^{10} 4p^3 4f^2$	17.65	3.71	9.05	28.60
$[Ar]4s^2 3d^{10} 4p^2 4f^3$	14.58	5.90	6.17	53.66
$[Ar]4s^1 3d^{10} 4f^6$	10.77	6.58	3.23	162.35
$[Ar]3d^{10} 4p^1 4f^6$	10.77	6.58	3.19	177.05
$[Ar]3d^{10} 4f^7$	10.07	6.34	2.89	214.70

Kr The energies of the low lying configurations of hard-wall confined Kr are shown in fig. S184 for the neutral atom and in fig. S185 for the cation. The ground state of the unconfined Kr is $[Ar]4s^2 3d^{10} 4p^6$. At $r_c = 1.1a_0$ the ground state changes to $[Ar]4s^1 3d^{10} 4f^7$. Furthermore, at $r_c = 1.0a_0$ we see a ground state crossing to $[Ar]3d^{10} 4f^8$.

At $r_c = 1.1a_0$ the state $[Ar]3d^{10} 4p^1 4f^7$ flips below the initial ground state. At $r_c = 1.1a_0$ the state $[Ar]4s^2 3d^{10} 4p^5 4f^1$ flips below the initial ground state. At $r_c = 1.1a_0$ the state $[Ar]4s^2 3d^{10} 4p^4 4f^2$ flips below the initial ground state. The ionization energy of the unconfined atom is $\Delta E_0 = 14.27$ eV. The studied configurations, atomic radii and excitation energies in the unconfined calculations are as follows:

configuration	r_ϵ	r_ρ	$r_{\max}$	ΔE
$[Ar]4s^2 3d^{10} 4p^6$	4.38	4.08	1.55	0.00
$[Ar]4s^2 3d^{10} 4p^5 5s^1$	8.31	4.67	4.46	10.05
$[Ar]4s^2 3d^{10} 4p^5 4d^1$	12.90	4.11	4.63	12.16
$[Ar]4s^2 3d^{10} 4p^5 4f^1$	24.53	3.78	16.00	13.34
$[Ar]4s^1 3d^{10} 4p^6 4d^1$	12.72	4.15	4.01	25.62
$[Ar]4s^2 3d^{10} 4p^4 4f^2$	17.68	3.64	9.10	33.72
$[Ar]4s^1 3d^{10} 4f^7$	10.14	6.34	2.77	222.26
$[Ar]3d^{10} 4p^1 4f^7$	10.14	6.34	2.73	238.53
$[Ar]3d^{10} 4f^8$	21.21	5.80	3.94	284.37

Rb The energies of the low lying configurations of hard-wall confined Rb are shown in fig. S186 for the neutral atom and in fig. S187 for the cation. The ground state of the unconfined Rb is [Kr]$5s^1$. At $r_c = 4.9a_0$ the ground state changes to [Kr]$4d^1$. Furthermore, at $r_c = 1.2a_0$ we see a ground state crossing to [Kr]$4f^1$. At $r_c = 1.1a_0$ we see a ground state crossing to [Ar]$4s^1 3d^{10} 4f^8$. Moreover, at $r_c = 1.0a_0$ we see a ground state crossing to [Ar]$3d^{10} 4f^9$.

At $r_c = 3.0a_0$ the state [Ar]$4s^2 3d^{10} 4p^5 4d^2$ flips below the initial ground state. At $r_c = 2.0a_0$ the state [Ar]$4s^2 3d^{10} 4p^5 4f^2$ flips below the initial ground state. At $r_c = 1.3a_0$ the state [Ar]$3d^{10} 4p^1 4f^8$ flips below the initial ground state. At $r_c = 1.1a_0$ the state [Kr]$5p^1$ flips below the initial ground state. The ionization energy of the unconfined atom is $\Delta E_0 = 4.33$ eV. The studied configurations, atomic radii and excitation energies in the unconfined calculations are as follows:

configuration	r_ϵ	r_ρ	$r_{\max}$	ΔE
$[Kr]5s^1$	8.11	4.67	4.31	0.00
$[Kr]5p^1$	10.95	3.98	5.98	1.58
$[Kr]4d^1$	13.18	4.02	5.28	2.27
$[Kr]4f^1$	24.56	3.71	16.02	3.39
$[Ar]4s^2 3d^{10} 4p^5 4d^2$	9.24	4.89	2.51	18.95
$[Ar]4s^2 3d^{10} 4p^5 4f^2$	17.71	3.57	9.15	26.66
$[Ar]4s^1 3d^{10} 4f^8$	21.66	5.90	11.85	283.22
$[Ar]3d^{10} 4p^1 4f^8$	21.74	5.90	12.07	301.05
$[Ar]3d^{10} 4f^9$	15.95	5.90	2.58	353.87

Sr The energies of the low lying configurations of hard-wall confined Sr are shown in fig. S188 for the neutral atom and in fig. S189 for the cation. The ground state of the unconfined Sr is [Kr]$5s^2$. At $r_c = 5.2a_0$ the ground state changes to [Kr]$5s^1 4d^1$. Furthermore, at $r_c = 4.8a_0$ we see a ground state crossing to [Kr]$4d^2$. At $r_c = 1.2a_0$ we see a ground state crossing to [Kr]$4f^2$. Moreover, at $r_c = 1.0a_0$ we see a ground state crossing to [Ar]$3d^{10} 4f^{10}$.

At $r_c = 3.6a_0$ the state [Kr]$4d^1 5p^1$ flips below the initial ground state. At $r_c = 3.5a_0$ the state [Kr]$4d^1 4f^1$ flips below the initial ground state. At $r_c = 3.5a_0$ the state [Ar]$4s^2 3d^{10} 4p^5 4d^3$ flips below the initial ground state. At $r_c = 1.5a_0$ the state [Ar]$4s^2 3d^{10} 4f^8$ flips below the initial ground state. At $r_c = 1.4a_0$ the state [Ar]$4s^1 3d^{10} 4f^9$ flips below the initial ground state. At $r_c = 1.1a_0$ the state [Kr]$5s^1 5p^1$ flips below the initial ground state. The ionization energy of the unconfined atom is $\Delta E_0 = 5.78$ eV. The studied configurations, atomic radii and excitation energies in the unconfined calculations are as follows:

configuration	r_ϵ	r_ρ	$r_{\max}$	ΔE
$[Kr]5s^2$	7.61	5.50	3.68	0.00
$[Kr]5s^1 4d^1$	7.94	5.17	3.76	1.65
$[Kr]5s^1 5p^1$	8.77	5.50	4.65	1.85
$[Kr]4d^1 5p^1$	9.44	5.17	4.93	3.80
$[Kr]4d^2$	9.30	4.78	2.35	3.93
$[Kr]4d^1 4f^1$	24.05	4.02	15.46	6.31
$[Kr]4f^2$	17.73	3.49	9.19	11.80
$[Ar]4s^2 3d^{10} 4p^5 4d^3$	7.71	5.02	2.05	23.68
$[Ar]4s^2 3d^{10} 4f^8$	21.46	5.82	3.09	276.15
$[Ar]4s^1 3d^{10} 4f^9$	16.52	5.90	2.82	348.91
$[Ar]3d^{10} 4f^{10}$	13.40	5.90	2.17	427.45

Y The energies of the low lying configurations of hard-wall confined Y are shown in fig. S190 for the neutral atom and in fig. S191 for the cation. The ground state of the unconfined Y is [Kr]$5s^2 4d^1$. At $r_c = 5.9a_0$ the ground state changes to [Kr]$5s^1 4d^2$. Furthermore, at $r_c = 4.9a_0$ we see a ground state crossing to [Kr]$4d^3$. At $r_c = 1.2a_0$ we see a ground state crossing to [Kr]$4d^2 4f^1$. Moreover, at $r_c = 1.1a_0$ we see a ground state crossing to [Kr]$4d^1 4f^2$. At $r_c = 1.0a_0$ we see a ground state crossing to [Ar]$4s^2 3d^{10} 4f^9$.

At $r_c = 3.6a_0$ the state [Kr]$4d^2 5p^1$ flips below the initial ground state. At $r_c = 3.4a_0$ the state [Ar]$4s^2 3d^{10} 4p^5 4d^4$ flips below the ini-

tial ground state. At $r_c = 1.5a_0$ the state [Ar]$4s^2 3d^{10} 4p^1 4f^8$ flips below the initial ground state. At $r_c = 1.3a_0$ the state [Ar]$4s^1 3d^{10} 4f^{10}$ flips below the initial ground state. At $r_c = 1.1a_0$ the state [Kr]$5s^1 4d^1 5p^1$ flips below the initial ground state. The ionization energy of the unconfined atom is $\Delta E_0 = 6.25$ eV. The studied configurations, atomic radii and excitation energies in the unconfined calculations are as follows:

configuration	r_ϵ	r_ρ	$r_{\max}$	ΔE
[Kr]$5s^2 4d^1$	7.15	5.33	3.42	0.00
[Kr]$5s^1 4d^2$	7.12	5.17	3.47	0.68
[Kr]$5s^1 4d^1 5p^1$	8.12	5.50	4.22	1.93
[Kr]$4d^3$	7.68	4.89	1.93	2.56
[Kr]$4d^2 5p^1$	8.70	5.17	4.49	3.17
[Kr]$4d^2 4f^1$	24.14	4.13	15.56	5.99
[Kr]$4d^1 4f^2$	17.59	3.78	8.96	13.43
[Ar]$4s^2 3d^{10} 4p^5 4d^4$	6.79	4.89	1.80	24.99
[Ar]$4s^2 3d^{10} 4p^1 4f^8$	21.79	5.90	12.03	275.60
[Ar]$4s^2 3d^{10} 4f^9$	16.44	5.90	2.31	337.12
[Ar]$4s^1 3d^{10} 4f^{10}$	14.25	5.90	2.25	417.98

Zr The energies of the low lying configurations of hard-wall confined Zr are shown in fig. S192 for the neutral atom and in fig. S193 for the cation. The ground state of the unconfined Zr is [Kr]$5s^1 4d^3$. At $r_c = 5.0a_0$ the ground state changes to [Kr]$4d^4$. Furthermore, at $r_c = 1.1a_0$ we see a ground state crossing to [Kr]$4d^2 4f^2$. At $r_c = 1.0a_0$ we see a ground state crossing to [Ar]$4s^2 3d^{10} 4p^3 4f^7$.

At $r_c = 2.7a_0$ the state [Ar]$4s^2 3d^{10} 4p^5 4d^5$ flips below the initial ground state. At $r_c = 2.6a_0$ the state [Kr]$4d^3 4f^1$ flips below the initial ground state. At $r_c = 1.2a_0$ the state [Ar]$4s^2 3d^{10} 4p^2 4f^8$ flips below the initial ground state. At $r_c = 1.2a_0$ the state [Ar]$4s^2 3d^{10} 4p^1 4f^9$ flips below the initial ground state. At $r_c = 1.0a_0$ the state [Kr]$4d^3 5p^1$ flips below the initial ground state. The ionization energy of the unconfined atom is $\Delta E_0 = 6.71$ eV. The studied configurations, atomic radii and excitation energies in the unconfined calculations are as follows:

configuration	r_ϵ	r_ρ	$r_{\max}$	ΔE
[Kr]$5s^1 4d^3$	6.59	5.02	3.26	0.00
[Kr]$5s^2 4d^2$	6.79	5.33	3.26	0.36
[Kr]$4d^4$	6.74	4.78	1.69	1.36
[Kr]$4d^3 5p^1$	8.28	5.17	4.20	2.76
[Kr]$4d^3 4f^1$	24.25	4.11	15.69	5.76
[Kr]$4d^2 4f^2$	17.57	3.83	8.91	14.98
[Ar]$4s^2 3d^{10} 4p^5 4d^5$	6.15	4.67	1.62	26.25
[Ar]$4s^2 3d^{10} 4p^3 4f^7$	10.44	6.34	2.44	205.99
[Ar]$4s^2 3d^{10} 4p^2 4f^8$	22.00	5.69	12.66	269.93
[Ar]$4s^2 3d^{10} 4p^1 4f^9$	16.87	5.90	2.48	336.79

Nb The energies of the low lying configurations of hard-wall confined Nb are shown in fig. S194 for the neutral atom and in fig. S195 for the cation. The ground state of the unconfined Nb is [Kr]$5s^1 4d^4$. At $r_c = 5.4a_0$ the ground state changes to [Kr]$4d^5$. Furthermore, at $r_c = 1.1a_0$ we see a ground state crossing to [Kr]$4f^5$.

At $r_c = 2.6a_0$ the state [Ar]$4s^2 3d^{10} 4p^5 4d^6$ flips below the initial ground state. At $r_c = 2.5a_0$ the state [Kr]$4d^4 4f^1$ flips below the initial ground state. At $r_c = 2.0a_0$ the state [Kr]$4d^3 4f^2$ flips below the initial ground state. At $r_c = 1.4a_0$ the state [Ar]$4s^2 3d^{10} 4p^5 4f^6$ flips below the initial ground state. At $r_c = 1.3a_0$ the state [Ar]$4s^2 3d^{10} 4p^4 4f^7$ flips below the initial ground state. At $r_c = 1.0a_0$ the state [Kr]$4d^4 5p^1$ flips below the initial ground state. The ionization energy of the unconfined atom is $\Delta E_0 = 7.04$ eV. The studied configurations, atomic radii and excitation energies in the unconfined calculations are as follows:

configuration	r_ϵ	r_ρ	$r_{\max}$	ΔE
[Kr]$5s^1 4d^4$	6.22	4.89	3.10	0.00
[Kr]$4d^5$	6.09	4.67	1.53	0.75
[Kr]$5s^2 4d^3$	6.53	5.17	3.13	1.43
[Kr]$4d^4 5p^1$	8.02	5.02	4.00	2.97
[Kr]$4d^4 4f^1$	24.34	4.05	15.78	6.10
[Kr]$4d^3 4f^2$	17.58	3.83	8.93	17.02
[Ar]$4s^2 3d^{10} 4p^5 4d^6$	6.21	4.59	1.50	30.83
[Kr]$4f^5$	11.80	6.58	3.64	75.09
[Ar]$4s^2 3d^{10} 4p^5 4f^6$	11.05	6.34	2.91	134.49
[Ar]$4s^2 3d^{10} 4p^4 4f^7$	10.49	6.34	2.42	195.78

Mo The energies of the low lying configurations of hard-wall confined Mo are shown in

fig. S196 for the neutral atom and in fig. S197 for the cation. The ground state of the unconfined Mo is [Kr]$5s^1 4d^5$. At $r_c = 4.3a_0$ the ground state changes to [Kr]$4d^6$. Furthermore, at $r_c = 1.1a_0$ we see a ground state crossing to [Kr]$4d^5 4f^1$. At $r_c = 1.0a_0$ we see a ground state crossing to [Kr]$4f^6$.

At $r_c = 2.4a_0$ the state [Ar]$4s^2 3d^{10} 4p^5 4d^7$ flips below the initial ground state. At $r_c = 1.9a_0$ the state [Kr]$4d^4 4f^2$ flips below the initial ground state. At $r_c = 1.4a_0$ the state [Kr]$4d^1 4f^5$ flips below the initial ground state. At $r_c = 1.3a_0$ the state [Ar]$4s^2 3d^{10} 4p^5 4f^7$ flips below the initial ground state. At $r_c = 1.0a_0$ the state [Kr]$4d^5 5p^1$ flips below the initial ground state. The ionization energy of the unconfined atom is $\Delta E_0 = 7.30$ eV. The studied configurations, atomic radii and excitation energies in the unconfined calculations are as follows:

configuration	r_ϵ	r_ρ	$r_{\max}$	ΔE
[Kr]$5s^1 4d^5$	5.94	4.78	2.97	0.00
[Kr]$4d^6$	5.95	4.51	1.40	2.48
[Kr]$5s^2 4d^4$	6.34	5.02	3.02	2.51
[Kr]$4d^5 5p^1$	7.84	4.89	3.84	3.16
[Kr]$4d^5 4f^1$	24.41	3.98	15.86	6.36
[Kr]$4d^4 4f^2$	17.61	3.78	8.97	18.92
[Ar]$4s^2 3d^{10} 4p^5 4d^7$	5.75	4.51	1.38	35.59
[Kr]$4d^1 4f^5$	11.86	6.58	3.52	84.78
[Kr]$4f^6$	11.07	6.34	2.93	112.66
[Ar]$4s^2 3d^{10} 4p^5 4f^7$	10.53	6.34	2.41	179.85

Tc The energies of the low lying configurations of hard-wall confined Tc are shown in fig. S198 for the neutral atom and in fig. S199 for the cation. The ground state of the unconfined Tc is [Kr]$5s^1 4d^6$. At $r_c = 4.7a_0$ the ground state changes to [Kr]$4d^7$. Furthermore, at $r_c = 1.0a_0$ we see a ground state crossing to [Kr]$4f^7$.

At $r_c = 2.3a_0$ the state [Kr]$4d^6 4f^1$ flips below the initial ground state. At $r_c = 2.3a_0$ the state [Ar]$4s^2 3d^{10} 4p^5 4d^8$ flips below the initial ground state. At $r_c = 1.9a_0$ the state [Kr]$4d^5 4f^2$ flips below the initial ground state. At $r_c = 1.4a_0$ the state [Kr]$4d^2 4f^5$ flips below the initial ground state. At $r_c = 1.3a_0$ the state [Kr]$4d^1 4f^6$ flips below the initial ground state. At $r_c = 1.0a_0$ the state [Kr]$4d^6 5p^1$ flips below the initial ground state. The ionization energy of the unconfined atom is $\Delta E_0 = 7.45$ eV. The studied configurations, atomic radii and excitation energies in the unconfined calculations are as follows:

configuration	r_ϵ	r_ρ	$r_{\max}$	ΔE
[Kr]$5s^1 4d^6$	5.80	4.67	2.87	0.00
[Kr]$5s^2 4d^5$	6.18	4.89	2.93	0.63
[Kr]$4d^7$	5.56	4.39	1.30	1.40
[Kr]$4d^6 5p^1$	7.76	4.78	3.74	3.28
[Kr]$4d^6 4f^1$	24.45	3.94	15.90	6.51
[Kr]$4d^5 4f^2$	17.64	3.64	9.02	17.75
[Ar]$4s^2 3d^{10} 4p^5 4d^8$	5.37	4.39	1.29	37.53
[Kr]$4d^2 4f^5$	11.91	6.58	3.44	91.40
[Kr]$4d^1 4f^6$	11.15	6.34	2.81	122.37
[Kr]$4f^7$	10.55	6.34	2.42	155.29

Ru The energies of the low lying configurations of hard-wall confined Ru are shown in fig. S200 for the neutral atom and in fig. S201 for the cation. The ground state of the unconfined Ru is [Kr]$5s^1 4d^7$. At $r_c = 5.9a_0$ the ground state changes to [Kr]$4d^8$. Furthermore, at $r_c = 1.0a_0$ we see a ground state crossing to [Kr]$4d^2 4f^6$.

At $r_c = 2.3a_0$ the state [Kr]$4d^7 4f^1$ flips below the initial ground state. At $r_c = 2.3a_0$ the state [Ar]$4s^2 3d^{10} 4p^5 4d^9$ flips below the initial ground state. At $r_c = 1.4a_0$ the state [Kr]$4d^3 4f^5$ flips below the initial ground state. At $r_c = 1.3a_0$ the state [Kr]$4d^1 4f^7$ flips below the initial ground state. The ionization energy of the unconfined atom is $\Delta E_0 = 7.54$ eV. The studied configurations, atomic radii and excitation energies in the unconfined calculations are as follows:

configuration	r_ϵ	r_ρ	$r_{\max}$	ΔE
[Kr]$5s^1 4d^7$	5.66	4.59	2.79	0.00
[Kr]$4d^8$	5.22	4.28	1.22	0.34
[Kr]$5s^2 4d^6$	5.97	4.78	2.79	1.68
[Kr]$4d^7 5p^1$	7.73	4.78	3.67	3.37
[Kr]$4d^7 4f^1$	24.48	3.83	15.94	6.60
[Ar]$4s^2 3d^{10} 4p^5 4d^9$	5.05	4.22	1.21	39.48
[Kr]$4d^3 4f^5$	11.97	6.58	3.39	97.85
[Kr]$4d^2 4f^6$	11.24	6.34	2.72	131.96
[Kr]$4d^1 4f^7$	10.66	6.34	2.30	168.12

Rh The energies of the low lying configurations of hard-wall confined Rh are shown in fig. S202 for the neutral atom and in fig. S203 for the cation. The ground state of the unconfined Rh is [Kr]$4d^9$. At $r_c = 1.0a_0$ the ground state changes to [Kr]$4d^5 4f^4$.

At $r_c = 1.0a_0$ the state [Kr]$4d^6 4f^3$ flips below the initial ground state. At $r_c = 1.0a_0$ the state [Kr]$4d^4 4f^5$ flips below the initial ground state. At $r_c = 1.0a_0$ the state [Kr]$4d^8 4f^1$ flips below the initial ground state. The ionization energy of the unconfined atom is $\Delta E_0 = 8.29$ eV. The studied configurations, atomic radii and excitation energies in the unconfined calculations are as follows:

configuration	r_ϵ	r_ρ	$r_{\max}$	ΔE
[Kr]$4d^9$	4.94	4.16	1.15	0.00
[Kr]$5s^1 4d^8$	5.54	4.45	2.72	0.70
[Kr]$5s^2 4d^7$	5.81	4.78	2.68	3.47
[Kr]$4d^8 5p^1$	7.72	4.67	3.62	4.15
[Kr]$4d^8 4f^1$	24.51	3.78	15.97	7.36
[Ar]$4s^2 3d^{10} 4p^5 4d^{10}$	4.79	4.13	1.14	42.15
[Kr]$4d^6 4f^3$	14.63	5.90	6.18	43.86
[Kr]$4d^5 4f^4$	13.00	6.58	4.47	70.46
[Kr]$4d^4 4f^5$	12.01	6.58	3.37	104.85

Pd The energies of the low lying configurations of hard-wall confined Pd are shown in fig. S204 for the neutral atom and in fig. S205 for the cation. The ground state of the unconfined Pd is [Kr]$4d^{10}$. We do not observe any ground state crossing for Pd in the considered confinement radii.

The ionization energy of the unconfined atom is $\Delta E_0 = 9.37$ eV. The studied configurations, atomic radii and excitation energies in the unconfined calculations are as follows:

configuration	r_ϵ	r_ρ	$r_{\max}$	ΔE
[Kr]$4d^{10}$	4.69	4.08	1.09	0.00
[Kr]$5s^1 4d^9$	5.45	4.39	2.67	1.74
[Kr]$4d^9 5p^1$	7.73	4.59	3.59	5.25
[Kr]$5s^2 4d^8$	5.68	4.67	2.58	5.62
[Kr]$4d^9 4f^1$	24.54	3.71	16.00	8.44
[Kr]$4d^8 4f^2$	17.72	3.49	9.17	25.06
[Ar]$4s^2 3d^{10} 4p^5 4d^{10} 4f^1$	24.54	3.71	16.01	54.26

Ag The energies of the low lying configurations of hard-wall confined Ag are shown in fig. S206 for the neutral atom and in fig. S207 for the cation. The ground state of the unconfined Ag is [Kr]$5s^1 4d^{10}$. At $r_c = 2.1a_0$ the ground state changes to [Kr]$4d^{10} 4f^1$.

At $r_c = 1.6a_0$ the state [Kr]$4d^9 4f^2$ flips below the initial ground state. At $r_c = 1.5a_0$ the state [Kr]$4d^8 4f^3$ flips below the initial ground state. The ionization energy of the unconfined atom is $\Delta E_0 = 7.66$ eV. The studied configurations, atomic radii and excitation energies in the unconfined calculations are as follows:

configuration	r_ϵ	r_ρ	$r_{\max}$	ΔE
[Kr]$5s^1 4d^{10}$	5.37	4.31	2.61	0.00
[Kr]$4d^{10} 5p^1$	7.75	4.51	3.56	3.56
[Kr]$5s^2 4d^9$	5.57	4.59	2.50	5.01
[Kr]$4d^{10} 4f^1$	24.55	3.64	16.02	6.73
[Kr]$4d^9 4f^2$	17.74	3.41	9.20	25.02
[Kr]$4d^8 4f^3$	14.64	5.90	6.28	51.37

Cd The energies of the low lying configurations of hard-wall confined Cd are shown in fig. S208 for the neutral atom and in fig. S209 for the cation. The ground state of the unconfined Cd is [Kr]$5s^2 4d^{10}$. At $r_c = 2.0a_0$ the ground state changes to [Kr]$4d^{10} 4f^2$.

At $r_c = 2.0a_0$ the state [Kr]$5s^1 4d^{10} 4f^1$ flips below the initial ground state. At $r_c = 1.8a_0$ the state [Kr]$4d^9 4f^3$ flips below the initial ground state. At $r_c = 1.8a_0$ the state [Kr]$4d^{10} 5p^1 4f^1$ flips below the initial ground state. At $r_c = 1.6a_0$ the state [Kr]$4d^8 4f^4$ flips below the initial ground state. At $r_c = 1.6a_0$ the state [Ar]$4s^2 3d^{10} 4p^5 4d^{10} 4f^3$ flips below the initial ground state. The ionization energy of the unconfined atom is $\Delta E_0 = 8.86$ eV. The studied configurations, atomic radii and excitation energies in the unconfined calculations are as follows:

configuration	r_ϵ	r_ρ	$r_{\max}$	ΔE
[Kr]$5s^2 4d^{10}$	5.47	4.51	2.43	0.00
[Kr]$5s^1 4d^{10} 5p^1$	6.71	4.78	3.13	3.71
[Kr]$5s^1 4d^{10} 5d^1$	14.69	4.08	7.83	7.11
[Kr]$5s^1 4d^{10} 4f^1$	24.41	3.94	15.86	7.92
[Kr]$4d^{10} 5p^2$	7.06	5.02	3.05	8.25
[Kr]$4d^{10} 5p^1 4f^1$	24.03	4.20	15.44	13.15
[Kr]$4d^{10} 4f^2$	17.76	3.33	9.23	20.54
[Kr]$4d^9 4f^3$	14.64	5.90	6.32	49.17
[Kr]$4d^8 4f^4$	12.97	6.58	4.63	84.87
[Ar]$4s^2 3d^{10} 4p^5 4d^{10} 4f^3$	14.64	5.90	6.32	102.74

In The energies of the low lying configurations of hard-wall confined In are shown in fig. S210 for the neutral atom and in fig. S211 for the cation. The ground state of the unconfined In is [Kr]$5s^2 4d^{10} 5p^1$. At $r_c = 2.2a_0$ the ground state changes to [Kr]$5s^2 4d^{10} 4f^1$. Furthermore, at $r_c = 2.0a_0$ we see a ground state crossing to [Kr]$4d^{10} 4f^3$.

At $r_c = 2.1a_0$ the state [Kr]$5s^1 4d^{10} 4f^2$ flips below the initial ground state. At $r_c = 2.0a_0$ the state [Kr]$4d^{10} 5p^1 4f^2$ flips below the initial ground state. At $r_c = 1.8a_0$ the state [Kr]$4d^9 4f^4$ flips below the initial ground state. At $r_c = 1.7a_0$ the state [Kr]$4d^8 4f^5$ flips below the initial ground state. At $r_c = 1.7a_0$ the state [Ar]$4s^2 3d^{10} 4p^5 4d^{10} 4f^4$ flips below the initial ground state. At $r_c = 1.0a_0$ the state [Kr]$5s^2 4d^{10} 5d^1$ flips below the initial ground state. The ionization energy of the unconfined atom is $\Delta E_0 = 5.77$ eV. The studied configurations, atomic radii and excitation energies in the unconfined calculations are as follows:

configuration	r_ϵ	r_ρ	$r_{\max}$	ΔE
[Kr]$5s^2 4d^{10} 5p^1$	6.24	4.78	2.87	0.00
[Kr]$5s^2 4d^{10} 6s^1$	11.62	4.19	6.92	2.93
[Kr]$5s^2 4d^{10} 5d^1$	14.35	4.28	7.44	3.98
[Kr]$5s^1 4d^{10} 5p^2$	6.37	5.02	2.77	4.16
[Kr]$5s^2 4d^{10} 4f^1$	24.41	4.08	15.86	4.84
[Kr]$4d^{10} 5p^3$	6.48	5.17	2.72	9.65
[Kr]$5s^1 4d^{10} 4f^2$	17.60	3.78	8.97	19.07
[Kr]$4d^{10} 5p^1 4f^2$	17.41	4.16	8.65	25.69
[Kr]$4d^{10} 4f^3$	14.65	5.90	6.35	37.05
[Kr]$4d^9 4f^4$	12.95	6.58	4.68	75.67
[Kr]$4d^8 4f^5$	12.01	6.58	3.54	119.68
[Ar]$4s^2 3d^{10} 4p^5 4d^{10} 4f^4$	12.96	6.58	4.67	133.14

Sn The energies of the low lying configurations of hard-wall confined Sn are shown in fig. S212 for the neutral atom and in fig. S213 for the cation. The ground state of the unconfined Sn is [Kr]$5s^2 4d^{10} 5p^2$. At $r_c = 2.2a_0$ the ground state changes to [Kr]$5s^2 4d^{10} 4f^2$. Furthermore, at $r_c = 1.9a_0$ we see a ground state crossing to [Kr]$4d^{10} 4f^4$.

At $r_c = 2.2a_0$ the state [Kr]$5s^2 4d^{10} 5p^1 4f^1$ flips below the initial ground state. At $r_c = 2.1a_0$ the state [Kr]$5s^1 4d^{10} 4f^3$ flips below the initial ground state. At $r_c = 1.9a_0$ the state [Kr]$4d^9 4f^5$ flips below the initial ground state. At $r_c = 1.8a_0$ the state [Kr]$4d^8 4f^6$ flips below the initial ground state. At $r_c = 1.8a_0$ the state [Ar]$4s^2 3d^{10} 4p^5 4d^{10} 4f^5$ flips below the initial ground state. The ionization energy of the unconfined atom is $\Delta E_0 = 7.48$ eV. The studied configurations, atomic radii and excitation energies in the unconfined calculations are as follows:

configuration	r_ϵ	r_ρ	$r_{\max}$	ΔE
[Kr]$5s^2 4d^{10} 5p^2$	5.98	4.89	2.57	0.00
[Kr]$5s^2 4d^{10} 5p^1 6s^1$	10.69	4.78	6.23	4.32
[Kr]$5s^1 4d^{10} 5p^3$	5.97	4.89	2.51	4.55
[Kr]$5s^2 4d^{10} 5p^1 5d^1$	12.86	4.78	5.78	5.45
[Kr]$5s^2 4d^{10} 5p^1 4f^1$	24.25	4.28	15.69	6.54
[Kr]$5s^2 4d^{10} 4f^2$	17.59	3.94	8.93	16.63
[Kr]$5s^1 4d^{10} 4f^3$	14.59	5.90	6.04	36.31
[Kr]$4d^{10} 4f^4$	12.94	6.84	4.72	58.79
[Kr]$4d^9 4f^5$	11.98	6.58	3.59	106.31
[Kr]$4d^8 4f^6$	11.43	6.34	2.73	157.44
[Ar]$4s^2 3d^{10} 4p^5 4d^{10} 4f^5$	12.01	6.58	3.58	167.59

Sb The energies of the low lying configurations of hard-wall confined Sb are shown in fig. S214 for the neutral atom and in fig. S215 for the cation. The ground state of the unconfined Sb is [Kr]$5s^2 4d^{10} 5p^3$. At $r_c = 2.1a_0$ the ground state changes to [Kr]$5s^2 4d^{10} 4f^3$. Furthermore, at $r_c = 1.9a_0$ we see a ground state crossing to [Kr]$4d^{10} 4f^5$.

At $r_c = 2.1a_0$ the state [Kr]$5s^1 4d^{10} 4f^4$ flips below the initial ground state. At $r_c = 2.1a_0$ the state [Kr]$5s^2 4d^{10} 5p^2 4f^1$ flips below the initial ground state. At $r_c = 2.1a_0$ the state [Kr]$5s^2 4d^{10} 5p^1 4f^2$ flips below the initial ground state. At $r_c = 1.9a_0$ the state [Kr]$4d^9 4f^6$ flips below the initial ground state. At $r_c = 1.8a_0$ the state [Kr]$4d^8 4f^7$ flips below the initial ground state. The ionization energy of the unconfined atom is $\Delta E_0 = 9.08$ eV. The studied configurations, atomic radii and excitation energies in the unconfined calculations are as follows:

configuration	r_ϵ	r_ρ	$r_{\max}$	ΔE
[Kr]$5s^2 4d^{10} 5p^3$	5.65	4.78	2.35	0.00
[Kr]$5s^2 4d^{10} 5p^2 6s^1$	10.06	5.02	5.77	5.68
[Kr]$5s^2 4d^{10} 5p^2 5d^1$	11.89	4.89	4.73	6.85
[Kr]$5s^1 4d^{10} 5p^4$	5.79	4.89	2.34	7.08
[Kr]$5s^2 4d^{10} 5p^2 4f^1$	24.25	4.35	15.69	8.14
[Kr]$5s^2 4d^{10} 5p^1 4f^2$	17.45	4.16	8.64	20.36
[Kr]$5s^2 4d^{10} 4f^3$	14.62	5.90	5.95	34.90
[Kr]$5s^1 4d^{10} 4f^4$	13.03	6.58	4.34	58.58
[Kr]$4d^{10} 4f^5$	11.96	6.58	3.64	84.42
[Kr]$4d^9 4f^6$	11.41	6.34	2.79	139.54
[Kr]$4d^8 4f^7$	11.08	6.11	2.08	196.89

Te The energies of the low lying configurations of hard-wall confined Te are shown in fig. S216 for the neutral atom and in fig. S217 for the cation. The ground state of the unconfined Te is [Kr]$5s^2 4d^{10} 5p^4$. At $r_c = 2.1a_0$ the ground state changes to [Kr]$5s^2 4d^{10} 4f^4$. Furthermore, at $r_c = 1.9a_0$ we see a ground state crossing to [Kr]$5s^1 4d^{10} 4f^5$. At $r_c = 1.8a_0$ we see a ground state crossing to [Kr]$4d^{10} 4f^6$.

At $r_c = 2.1a_0$ the state [Kr]$5s^2 4d^{10} 5p^1 4f^3$ flips below the initial ground state. At $r_c = 2.1a_0$ the state [Kr]$5s^2 4d^{10} 5p^3 4f^1$ flips below the initial ground state. At $r_c = 2.1a_0$ the state [Kr]$5s^2 4d^{10} 5p^2 4f^2$ flips below the initial ground state. At $r_c = 1.9a_0$ the state [Kr]$4d^9 4f^7$ flips below the initial ground state. At $r_c = 1.8a_0$ the state [Kr]$4d^8 4f^8$ flips below the initial ground state. At $r_c = 1.8a_0$ the state [Ar]$4s^2 3d^{10} 4p^5 4d^{10} 4f^7$ flips below the initial ground state. The ionization energy of the unconfined atom is $\Delta E_0 = 9.08$ eV. The studied configurations, atomic radii and excitation energies in the unconfined calculations are as follows:

configuration	r_ϵ	r_ρ	$r_{\max}$	ΔE
[Kr]$5s^2 4d^{10} 5p^4$	5.45	4.78	2.18	0.00
[Kr]$5s^2 4d^{10} 5p^3 6s^1$	9.59	5.02	5.42	5.47
[Kr]$5s^2 4d^{10} 5p^3 5d^1$	11.16	4.89	4.06	6.68
[Kr]$5s^1 4d^{10} 5p^5$	5.50	4.78	2.17	8.03
[Kr]$5s^2 4d^{10} 5p^3 4f^1$	24.29	4.28	15.73	8.14
[Kr]$5s^2 4d^{10} 5p^2 4f^2$	17.43	4.23	8.48	22.40
[Kr]$5s^2 4d^{10} 5p^1 4f^3$	14.72	5.69	5.48	38.96
[Kr]$5s^2 4d^{10} 4f^4$	13.20	6.34	4.13	56.48
[Kr]$5s^1 4d^{10} 4f^5$	12.19	6.34	3.19	82.56
[Kr]$4d^{10} 4f^6$	11.39	6.34	2.85	110.77
[Kr]$4d^9 4f^7$	11.06	6.34	2.14	172.45
[Kr]$4d^8 4f^8$	22.00	5.69	12.77	236.90
[Ar]$4s^2 3d^{10} 4p^5 4d^{10} 4f^7$	11.11	6.11	2.09	241.06

I The energies of the low lying configurations of hard-wall confined I are shown in fig. S218 for the neutral atom and in fig. S219 for the cation. The ground state of the unconfined I is [Kr]$5s^2 4d^{10} 5p^5$. At $r_c = 2.1a_0$ the ground state changes to [Kr]$5s^2 4d^{10} 5p^3 4f^2$. Furthermore, at $r_c = 2.0a_0$ we see a ground state crossing to [Kr]$5s^2 4d^{10} 4f^5$. At $r_c = 1.8a_0$ we see a ground state crossing to [Kr]$4d^{10} 4f^7$.

At $r_c = 2.1a_0$ the state [Kr]$5s^2 4d^{10} 5p^1 4f^4$ flips below the initial ground state. At $r_c = 2.1a_0$ the state [Kr]$5s^2 4d^{10} 5p^4 4f^1$ flips below the initial ground state. At $r_c = 2.0a_0$ the state [Kr]$5s^1 4d^{10} 4f^6$ flips below the initial ground state. At $r_c = 2.0a_0$ the state [Kr]$4d^{10} 5p^1 4f^6$ flips below the initial ground state. At $r_c = 1.9a_0$ the state [Kr]$4d^9 4f^8$ flips below the initial ground state. At $r_c = 1.8a_0$ the state [Kr]$4d^8 4f^9$ flips below the initial ground state. At $r_c = 1.8a_0$ the state [Ar]$4s^2 3d^{10} 4p^5 4d^{10} 4f^8$ flips below the initial ground state. The ionization energy of the unconfined atom is $\Delta E_0 = 10.86$ eV. The studied configurations, atomic radii and excitation energies in the unconfined calculations are as follows:

configuration	r_ϵ	r_ρ	$r_{\max}$	ΔE
[Kr]$5s^2 4d^{10} 5p^5$	5.20	4.59	2.04	0.00
[Kr]$5s^2 4d^{10} 5p^4 6s^1$	9.29	5.02	5.19	7.12
[Kr]$5s^2 4d^{10} 5p^4 5d^1$	10.99	4.78	3.74	8.41
[Kr]$5s^1 4d^{10} 5p^6$	5.22	4.67	2.03	8.97
[Kr]$5s^2 4d^{10} 5p^4 4f^1$	24.34	4.22	15.79	9.92
[Kr]$5s^2 4d^{10} 5p^3 4f^2$	17.52	4.25	8.39	24.32
[Kr]$5s^2 4d^{10} 5p^1 4f^4$	13.60	6.11	3.20	61.43
[Kr]$5s^2 4d^{10} 4f^5$	12.53	6.34	2.76	80.56
[Kr]$5s^1 4d^{10} 4f^6$	11.73	6.34	2.33	107.99
[Kr]$4d^{10} 5p^1 4f^6$	11.75	6.11	2.26	118.27
[Kr]$4d^{10} 4f^7$	11.05	6.34	2.20	137.95
[Kr]$4d^9 4f^8$	21.89	5.69	12.51	206.97
[Kr]$4d^8 4f^9$	17.29	5.69	5.39	277.63
[Ar]$4s^2 3d^{10} 4p^5 4d^{10} 4f^8$	21.88	5.69	12.49	279.11

Xe The energies of the low lying configurations of hard-wall confined Xe are shown in fig. S220 for the neutral atom and in fig. S221 for the cation. The ground state of the unconfined Xe is [Kr]$5s^2 4d^{10} 5p^6$. At $r_c = 2.0a_0$ the ground state changes to [Kr]$5s^2 4d^{10} 4f^6$. Furthermore, at $r_c = 1.8a_0$ we see a ground state crossing to [Kr]$5s^1 4d^{10} 4f^7$. At $r_c = 1.7a_0$ we see a ground state crossing to [Kr]$4d^{10} 4f^8$.

At $r_c = 2.0a_0$ the state [Kr]$5s^2 4d^{10} 5p^1 4f^5$ flips below the initial ground state. At $r_c = 2.0a_0$ the state [Kr]$5s^2 4d^{10} 5p^2 4f^4$ flips below the initial ground state. At $r_c = 2.0a_0$ the state [Kr]$5s^2 4d^{10} 5p^5 4f^1$ flips below the initial ground state. At $r_c = 2.0a_0$ the state [Kr]$5s^2 4d^{10} 5p^4 4f^2$ flips below the initial ground state. At $r_c = 1.9a_0$ the state [Kr]$4d^{10} 5p^1 4f^7$ flips below the initial ground state. At $r_c = 1.8a_0$ the state [Kr]$4d^9 4f^9$ flips below the initial ground state. At $r_c = 1.8a_0$ the state [Ar]$4s^2 3d^{10} 4p^5 4d^{10} 4f^9$ flips below the initial ground state. The ionization energy of the unconfined atom is $\Delta E_0 = 12.57$ eV. The studied configurations, atomic radii and excitation energies in the unconfined calculations are as follows:

configuration	r_ϵ	r_ρ	$r_{\max}$	ΔE
[Kr]$5s^2 4d^{10} 5p^6$	4.96	4.51	1.92	0.00
[Kr]$5s^2 4d^{10} 5p^5 6s^1$	9.04	4.89	5.00	8.72
[Kr]$5s^2 4d^{10} 5p^5 5d^1$	10.98	4.67	3.48	10.10
[Kr]$5s^2 4d^{10} 5p^5 4f^1$	24.39	4.16	15.84	11.63
[Kr]$5s^2 4d^{10} 5p^4 4f^2$	17.83	4.21	8.52	28.16
[Kr]$5s^2 4d^{10} 5p^2 4f^4$	14.30	5.69	1.85	65.17
[Kr]$5s^2 4d^{10} 5p^1 4f^5$	13.15	5.90	1.85	85.09
[Kr]$5s^2 4d^{10} 4f^6$	12.22	6.11	1.66	105.14
[Kr]$5s^1 4d^{10} 4f^7$	11.49	6.11	1.67	133.41
[Kr]$4d^{10} 5p^1 4f^7$	11.51	6.11	1.85	144.29
[Kr]$4d^{10} 4f^8$	21.79	5.69	12.17	166.25
[Kr]$4d^9 4f^9$	17.39	5.69	1.33	241.54
[Ar]$4s^2 3d^{10} 4p^5 4d^{10} 4f^9$	17.42	5.69	1.28	317.17

4.2 PBE

H The energies of the low lying configurations of hard-wall confined H are shown in fig. S115 for the neutral atom. The ground state of the unconfined H is $1s^1$. We do not observe any ground state crossing for H in the considered confinement radii.

The ionization energy of the unconfined atom is $\Delta E_0 = 13.61$ eV. The studied configurations, atomic radii and excitation energies in the unconfined calculations are as follows:

configuration	r_ϵ	r_ρ	$r_{\max}$	ΔE
$1s^1$	3.02	2.96	0.98	0.00
$2p^1$	8.46	4.05	4.03	10.12
$3d^1$	15.53	undefined	9.16	11.98

He The energies of the low lying configurations of hard-wall confined He are shown in fig. S116 for the neutral atom and in fig. S117 for the cation. The ground state of the unconfined He is $1s^2$. We do not observe any ground state crossing for He in the considered confinement radii.

The ionization energy of the unconfined atom is $\Delta E_0 = 24.47$ eV. The studied configurations, atomic radii and excitation energies in the unconfined calculations are as follows:

configuration	r_ϵ	r_ρ	$r_{\max}$	ΔE
$1s^2$	2.26	2.68	0.56	0.00
$1s^1 2p^1$	8.07	4.05	3.68	20.66
$1s^1 3d^1$	15.48	2.10	9.12	22.83
$2p^2$	6.13	4.45	2.28	58.43

Li The energies of the low lying configurations of hard-wall confined Li are shown in fig. S118 for the neutral atom and in fig. S119 for the cation. The ground state of the unconfined Li is [He]$2s^1$. At $r_c = 3.0a_0$ the ground state changes to [He]$2p^1$.

The ionization energy of the unconfined atom is $\Delta E_0 = 5.59$ eV. The studied configurations, atomic radii and excitation energies in the unconfined calculations are as follows:

configuration	r_ϵ	r_ρ	$r_{\max}$	ΔE
[He]$2s^1$	6.34	4.17	3.07	0.00
[He]$2p^1$	8.14	4.05	3.75	1.87
[He]$3d^1$	15.51	1.91	9.14	3.96
$1s^1 2p^2$	5.99	4.39	2.15	59.57

Be The energies of the low lying configurations of hard-wall confined Be are shown in fig. S120 for the neutral atom and in fig. S121 for the cation. The ground state of the unconfined Be is [He]$2s^2$. At $r_c = 2.5a_0$ the ground state changes to [He]$2s^1 2p^1$. Furthermore, at $r_c = 2.2a_0$ we see a ground state crossing to [He]$2p^2$.

At $r_c = 1.2a_0$ the state [He]$2p^1 3d^1$ flips below the initial ground state. The ionization energy of the unconfined atom is $\Delta E_0 = 9.00$ eV. The studied configurations, atomic radii and excitation energies in the unconfined calculations are as follows:

configuration	r_ϵ	r_ρ	$r_{\max}$	ΔE
[He]$2s^2$	5.06	4.15	2.04	0.00
[He]$2s^1 2p^1$	5.58	4.19	2.17	2.44
[He]$2p^2$	5.99	4.39	2.16	6.20
[He]$2p^1 3d^1$	14.70	3.78	8.26	11.22

B The energies of the low lying configurations of hard-wall confined B are shown in fig. S122 for the neutral atom and in fig. S123 for the cation. The ground state of the unconfined B is [He]$2s^2 2p^1$. At $r_c = 2.1a_0$ the ground state changes to [He]$2s^1 2p^2$. Furthermore, at $r_c = 1.8a_0$ we see a ground state crossing to [He]$2p^3$.

The ionization energy of the unconfined atom is $\Delta E_0 = 8.49$ eV. The studied configurations, atomic radii and excitation energies in the unconfined calculations are as follows:

configuration	r_ϵ	r_ρ	$r_{\max}$	ΔE
[He]$2s^2 2p^1$	4.59	3.94	1.57	0.00
[He]$2s^1 2p^2$	4.68	3.98	1.53	3.19
[He]$2s^2 3s^1$	9.77	3.78	5.61	5.06
[He]$2p^3$	4.87	4.05	1.52	9.20

C The energies of the low lying configurations of hard-wall confined C are shown in fig. S124 for the neutral atom and in fig. S125 for the cation. The ground state of the unconfined C is [He]$2s^2 2p^2$. At $r_c = 1.8a_0$ the ground state changes to [He]$2s^1 2p^3$. Furthermore, at $r_c = 1.3a_0$ we see a ground state crossing to [He]$2p^4$.

The ionization energy of the unconfined atom is $\Delta E_0 = 11.68$ eV. The studied configurations, atomic radii and excitation energies in the unconfined calculations are as follows:

configuration	r_ϵ	r_ρ	$r_{\max}$	ΔE
[He]$2s^2 2p^2$	4.02	3.71	1.22	0.00
[He]$2s^1 2p^3$	4.05	3.71	1.20	3.98
[He]$2s^2 2p^1 3s^1$	8.80	4.23	4.86	7.79
[He]$2p^4$	4.42	3.89	1.23	16.82

N The energies of the low lying configurations of hard-wall confined N are shown in fig. S126 for the neutral atom and in fig. S127 for the cation. The ground state of the unconfined N is [He]$2s^2 2p^3$. At $r_c = 1.2a_0$ the ground state changes to [He]$2p^5$.

At $r_c = 1.2a_0$ the state [He]$2s^1 2p^4$ flips below the initial ground state. The ionization energy of the unconfined atom is $\Delta E_0 = 14.91$ eV. The studied configurations, atomic radii and excitation energies in the unconfined calculations are as follows:

configuration	r_ϵ	r_ρ	$r_{\max}$	ΔE
[He]$2s^2 2p^3$	3.57	3.49	1.03	0.00
[He]$2s^2 2p^2 3s^1$	8.13	4.45	4.34	10.62
[He]$2s^1 2p^4$	3.87	3.64	1.01	11.07
[He]$2p^5$	3.89	3.64	0.98	25.73

O The energies of the low lying configurations of hard-wall confined O are shown in fig. S128 for the neutral atom and in fig. S129 for the cation. The ground state of the unconfined O is [He]$2s^2 2p^4$. At $r_c = 1.1a_0$ the ground state changes to [He]$2s^1 2p^5$. Furthermore, at

$r_c = 1.0a_0$ we see a ground state crossing to [He]$2p^6$.

The ionization energy of the unconfined atom is $\Delta E_0 = 13.68$ eV. The studied configurations, atomic radii and excitation energies in the unconfined calculations are as follows:

configuration	r_ϵ	r_ρ	$r_{\max}$	ΔE
[He]$2s^2 2p^4$	3.34	3.33	0.88	0.00
[He]$2s^2 2p^3 3s^1$	7.61	4.45	3.96	9.04
[He]$2s^2 2p^3 3d^1$	15.19	2.97	8.82	12.01
[He]$2s^1 2p^5$	3.47	3.41	0.87	14.68
[He]$2p^6$	3.49	3.49	0.82	31.35

F The energies of the low lying configurations of hard-wall confined F are shown in fig. S130 for the neutral atom and in fig. S131 for the cation. The ground state of the unconfined F is [He]$2s^2 2p^5$. At $r_c = 1.0a_0$ the ground state changes to [He]$2s^1 2p^6$.

The ionization energy of the unconfined atom is $\Delta E_0 = 17.85$ eV. The studied configurations, atomic radii and excitation energies in the unconfined calculations are as follows:

configuration	r_ϵ	r_ρ	$r_{\max}$	ΔE
[He]$2s^2 2p^5$	3.08	3.24	0.77	0.00
[He]$2s^2 2p^4 3s^1$	7.23	4.39	3.69	12.93
[He]$2s^2 2p^4 3d^1$	15.27	2.82	8.90	16.19
[He]$2s^1 2p^6$	3.14	3.24	0.76	18.61
[He]$2s^1 2p^5 3d^1$	15.25	2.90	8.88	35.09

Ne The energies of the low lying configurations of hard-wall confined Ne are shown in fig. S132 for the neutral atom and in fig. S133 for the cation. The ground state of the unconfined Ne is [He]$2s^2 2p^6$. We do not observe any ground state crossing for Ne in the considered confinement radii.

The ionization energy of the unconfined atom is $\Delta E_0 = 21.98$ eV. The studied configurations, atomic radii and excitation energies in the unconfined calculations are as follows:

configuration	r_ϵ	r_ρ	$r_{\max}$	ΔE
[He]$2s^2 2p^6$	2.84	3.06	0.68	0.00
[He]$2s^2 2p^5 3s^1$	6.93	4.31	3.48	16.84
[He]$2s^2 2p^5 3d^1$	15.33	2.70	8.96	20.33
[He]$2s^1 2p^6 3d^1$	15.32	2.76	8.95	43.52

Na The energies of the low lying configurations of hard-wall confined Na are shown in fig. S134 for the neutral atom and in fig. S135 for the cation. The ground state of the unconfined Na is [Ne]$3s^1$. At $r_c = 2.0a_0$ the ground state changes to [Ne]$3d^1$.

At $r_c = 1.6a_0$ the state [Ne]$3p^1$ flips below the initial ground state. At $r_c = 1.1a_0$ the state [Ne]$4f^1$ flips below the initial ground state. The ionization energy of the unconfined atom is $\Delta E_0 = 5.36$ eV. The studied configurations, atomic radii and excitation energies in the unconfined calculations are as follows:

configuration	r_ϵ	r_ρ	$r_{\max}$	ΔE
[Ne]$3s^1$	6.69	4.26	3.31	0.00
[Ne]$3p^1$	9.33	3.64	4.77	2.13
[Ne]$3d^1$	15.38	2.60	9.01	3.72
[Ne]$4f^1$	23.80	2.58	16.17	4.40

Mg The energies of the low lying configurations of hard-wall confined Mg are shown in fig. S136 for the neutral atom and in fig. S137 for the cation. The ground state of the unconfined Mg is [Ne]$3s^2$. At $r_c = 1.8a_0$ the ground state changes to [Ne]$3d^2$.

At $r_c = 1.8a_0$ the state [Ne]$3s^1 3d^1$ flips below the initial ground state. At $r_c = 1.7a_0$ the state [Ne]$3p^1 3d^1$ flips below the initial ground state. At $r_c = 1.5a_0$ the state [Ne]$3s^1 3p^1$ flips below the initial ground state. At $r_c = 1.5a_0$ the state [Ne]$3p^2$ flips below the initial ground state. At $r_c = 1.3a_0$ the state [Ne]$3d^1 4f^1$ flips below the initial ground state. The ionization energy of the unconfined atom is $\Delta E_0 = 7.61$ eV. The studied configurations, atomic radii and excitation energies in the unconfined calculations are as follows:

configuration	r_ϵ	r_ρ	$r_{\max}$	ΔE
[Ne]$3s^2$	5.89	4.59	2.56	0.00
[Ne]$3s^1 3p^1$	6.97	4.78	3.22	2.71
[Ne]$3s^1 3d^1$	14.20	4.13	7.62	5.76
[Ne]$3p^2$	7.32	5.17	3.21	6.45
[Ne]$3p^1 3d^1$	12.36	4.89	5.39	9.88
[Ne]$3d^2$	11.24	5.50	5.12	13.68
[Ne]$3d^1 4f^1$	22.01	4.17	14.12	15.52

Al The energies of the low lying configurations of hard-wall confined Al are shown in fig. S138 for the neutral atom and in fig. S139 for the cation. The ground state of the unconfined Al is [Ne]$3s^2 3p^1$. At $r_c = 1.9a_0$ the ground

state changes to [Ne]$3s^23d^1$. Furthermore, at $r_c = 1.7a_0$ we see a ground state crossing to [Ne]$3s^13d^2$. At $r_c = 1.6a_0$ we see a ground state crossing to [Ne]$3d^3$.

At $r_c = 1.7a_0$ the state [Ne]$3p^13d^2$ flips below the initial ground state. At $r_c = 1.4a_0$ the state [Ne]$3s^13p^2$ flips below the initial ground state. The ionization energy of the unconfined atom is ΔE_0 = 5.96 eV. The studied configurations, atomic radii and excitation energies in the unconfined calculations are as follows:

configuration	r_ϵ	r_ρ	$r_{\max}$	ΔE
[Ne]$3s^23p^1$	6.02	4.59	2.64	0.00
[Ne]$3s^24s^1$	11.17	4.12	6.72	3.01
[Ne]$3s^13p^2$	6.08	4.78	2.52	3.48
[Ne]$3s^23d^1$	14.05	4.16	7.51	4.10
[Ne]$3s^13d^2$	10.52	5.33	4.03	14.27
[Ne]$3p^13d^2$	9.94	5.50	3.72	20.19
[Ne]$3d^3$	9.26	5.90	3.55	26.54

Si The energies of the low lying configurations of hard-wall confined Si are shown in fig. S140 for the neutral atom and in fig. S141 for the cation. The ground state of the unconfined Si is [Ne]$3s^23p^2$. At $r_c = 1.7a_0$ the ground state changes to [Ne]$3s^23d^2$. Furthermore, at $r_c = 1.6a_0$ we see a ground state crossing to [Ne]$3s^13d^3$. At $r_c = 1.5a_0$ we see a ground state crossing to [Ne]$3d^4$.

At $r_c = 1.7a_0$ the state [Ne]$3s^23p^13d^1$ flips below the initial ground state. At $r_c = 1.6a_0$ the state [Ne]$3p^13d^3$ flips below the initial ground state. At $r_c = 1.4a_0$ the state [Ne]$3s^13p^3$ flips below the initial ground state. The ionization energy of the unconfined atom is ΔE_0 = 8.27 eV. The studied configurations, atomic radii and excitation energies in the unconfined calculations are as follows:

configuration	r_ϵ	r_ρ	$r_{\max}$	ΔE
[Ne]$3s^23p^2$	5.41	4.45	2.16	0.00
[Ne]$3s^13p^3$	5.38	4.51	2.09	4.14
[Ne]$3s^23p^14s^1$	10.14	4.45	5.87	4.94
[Ne]$3s^23p^13d^1$	13.19	4.35	4.95	6.19
[Ne]$3s^23d^2$	10.31	5.17	3.67	13.69
[Ne]$3s^13d^3$	8.78	5.50	2.88	27.83
[Ne]$3p^13d^3$	8.50	5.50	2.81	35.54
[Ne]$3d^4$	8.03	5.50	2.71	44.61

P The energies of the low lying configurations of hard-wall confined P are shown in fig. S142 for the neutral atom and in fig. S143 for the cation. The ground state of the unconfined P is [Ne]$3s^23p^3$. At $r_c = 1.5a_0$ the ground state changes to [Ne]$3s^13d^4$. Furthermore, at $r_c = 1.4a_0$ we see a ground state crossing to [Ne]$3d^5$.

At $r_c = 1.5a_0$ the state [Ne]$3p^13d^4$ flips below the initial ground state. At $r_c = 1.5a_0$ the state [Ne]$3s^23p^23d^1$ flips below the initial ground state. At $r_c = 1.5a_0$ the state [Ne]$3s^23p^13d^2$ flips below the initial ground state. At $r_c = 1.1a_0$ the state [Ne]$3s^13p^4$ flips below the initial ground state. The ionization energy of the unconfined atom is ΔE_0 = 10.55 eV. The studied configurations, atomic radii and excitation energies in the unconfined calculations are as follows:

configuration	r_ϵ	r_ρ	$r_{\max}$	ΔE
[Ne]$3s^23p^3$	4.88	4.22	1.83	0.00
[Ne]$3s^23p^24s^1$	9.43	4.59	5.30	6.89
[Ne]$3s^13p^4$	5.08	4.35	1.85	8.27
[Ne]$3s^23p^23d^1$	12.95	4.23	3.79	8.35
[Ne]$3s^23p^13d^2$	9.80	5.02	2.84	18.33
[Ne]$3s^13d^4$	7.69	5.33	2.27	46.94
[Ne]$3p^13d^4$	7.52	5.17	2.25	56.45
[Ne]$3d^5$	7.19	5.33	2.18	68.36

S The energies of the low lying configurations of hard-wall confined S are shown in fig. S144 for the neutral atom and in fig. S145 for the cation. The ground state of the unconfined S is [Ne]$3s^23p^4$. At $r_c = 1.5a_0$ the ground state changes to [Ne]$3s^23p^33d^1$. Furthermore, at $r_c = 1.4a_0$ we see a ground state crossing to [Ne]$3s^13d^5$. At $r_c = 1.2a_0$ we see a ground state crossing to [Ne]$3d^6$.

At $r_c = 1.5a_0$ the state [Ne]$3s^23p^23d^2$ flips below the initial ground state. At $r_c = 1.4a_0$ the state [Ne]$3p^13d^5$ flips below the initial ground state. At $r_c = 1.4a_0$ the state [Ne]$3s^23d^4$ flips below the initial ground state. At $r_c = 1.4a_0$ the state [Ne]$3s^13p^13d^4$ flips below the initial ground state. At $r_c = 1.1a_0$ the state [Ne]$3s^13p^5$ flips below the initial ground state. The ionization energy of the unconfined atom is ΔE_0 = 10.25 eV. The studied configurations, atomic radii and excitation energies in the unconfined

calculations are as follows:

configuration	r_ϵ	r_ρ	$r_{\max}$	ΔE
[Ne]$3s^2 3p^4$	4.58	4.11	1.61	0.00
[Ne]$3s^2 3p^3 4s^1$	8.89	4.59	4.87	6.31
[Ne]$3s^2 3p^3 3d^1$	13.02	4.08	3.08	7.99
[Ne]$3s^1 3p^5$	4.65	4.15	1.61	10.04
[Ne]$3s^2 3p^2 3d^2$	9.57	4.78	2.36	20.59
[Ne]$3s^2 3d^4$	7.54	5.17	2.06	47.72
[Ne]$3s^1 3p^1 3d^4$	7.33	5.02	1.95	54.93
[Ne]$3s^1 3d^5$	6.93	5.02	1.88	69.46
[Ne]$3p^1 3d^5$	6.81	5.02	1.87	80.79
[Ne]$3d^6$	7.69	5.02	1.94	98.86

Cl The energies of the low lying configurations of hard-wall confined Cl are shown in fig. S146 for the neutral atom and in fig. S147 for the cation. The ground state of the unconfined Cl is [Ne]$3s^2 3p^5$. At $r_c = 1.4a_0$ the ground state changes to [Ne]$3s^2 3p^3 3d^2$. Furthermore, at $r_c = 1.3a_0$ we see a ground state crossing to [Ne]$3s^1 3d^6$. At $r_c = 1.2a_0$ we see a ground state crossing to [Ne]$3d^7$.

At $r_c = 1.4a_0$ the state [Ne]$3s^2 3p^2 3d^3$ flips below the initial ground state. At $r_c = 1.4a_0$ the state [Ne]$3s^2 3p^4 3d^1$ flips below the initial ground state. At $r_c = 1.3a_0$ the state [Ne]$3p^1 3d^6$ flips below the initial ground state. At $r_c = 1.0a_0$ the state [Ne]$3s^1 3p^6$ flips below the initial ground state. The ionization energy of the unconfined atom is $\Delta E_0 = 13.06$ eV. The studied configurations, atomic radii and excitation energies in the unconfined calculations are as follows:

configuration	r_ϵ	r_ρ	$r_{\max}$	ΔE
[Ne]$3s^2 3p^5$	4.25	3.94	1.43	0.00
[Ne]$3s^2 3p^4 4s^1$	8.48	4.59	4.58	8.92
[Ne]$3s^2 3p^4 3d^1$	13.42	3.94	3.11	10.90
[Ne]$3s^1 3p^6$	4.27	3.98	1.43	11.89
[Ne]$3s^2 3p^3 3d^2$	9.51	4.59	2.02	22.93
[Ne]$3s^2 3p^2 3d^3$	8.03	4.89	1.86	39.08
[Ne]$3s^1 3d^6$	10.19	4.89	1.84	102.13
[Ne]$3p^1 3d^6$	10.04	4.78	1.78	115.37
[Ne]$3d^7$	6.79	4.89	1.64	136.33

Ar The energies of the low lying configurations of hard-wall confined Ar are shown in fig. S148 for the neutral atom and in fig. S149 for the cation. The ground state of the unconfined Ar is [Ne]$3s^2 3p^6$. At $r_c = 1.3a_0$ the ground state changes to [Ne]$3s^2 3p^4 3d^2$. Furthermore, at $r_c = 1.2a_0$ we see a ground state crossing to [Ne]$3s^1 3p^2 3d^5$. At $r_c = 1.1a_0$ we see a ground state crossing to [Ne]$3d^8$.

At $r_c = 1.3a_0$ the state [Ne]$3s^2 3p^1 3d^5$ flips below the initial ground state. At $r_c = 1.3a_0$ the state [Ne]$3s^2 3p^5 3d^1$ flips below the initial ground state. At $r_c = 1.2a_0$ the state [Ne]$3p^1 3d^7$ flips below the initial ground state. At $r_c = 1.2a_0$ the state [Ne]$3s^1 3d^7$ flips below the initial ground state. At $r_c = 1.2a_0$ the state [Ne]$3s^2 3d^6$ flips below the initial ground state. At $r_c = 1.1a_0$ the state [Ne]$3s^1 3p^6 3d^1$ flips below the initial ground state. The ionization energy of the unconfined atom is $\Delta E_0 = 15.81$ eV. The studied configurations, atomic radii and excitation energies in the unconfined calculations are as follows:

configuration	r_ϵ	r_ρ	$r_{\max}$	ΔE
[Ne]$3s^2 3p^6$	3.95	3.83	1.29	0.00
[Ne]$3s^2 3p^5 4s^1$	8.18	4.59	4.36	11.51
[Ne]$3s^2 3p^5 3d^1$	13.80	3.78	6.95	13.76
[Ne]$3s^1 3p^6 3d^1$	13.71	3.83	6.70	27.99
[Ne]$3s^2 3p^4 3d^2$	9.72	4.51	1.80	29.60
[Ne]$3s^2 3p^1 3d^5$	6.59	4.78	1.51	84.19
[Ne]$3s^1 3p^2 3d^5$	6.45	4.67	1.46	92.38
[Ne]$3s^2 3d^6$	7.87	4.78	1.51	107.71
[Ne]$3s^1 3d^7$	7.46	4.78	1.55	142.38
[Ne]$3p^1 3d^7$	7.35	4.78	1.52	157.52
[Ne]$3d^8$	6.17	4.78	1.41	181.20

K The energies of the low lying configurations of hard-wall confined K are shown in fig. S150 for the neutral atom and in fig. S151 for the cation. The ground state of the unconfined K is [Ar]$4s^1$. At $r_c = 4.6a_0$ the ground state changes to [Ar]$3d^1$. Furthermore, at $r_c = 1.2a_0$ we see a ground state crossing to [Ne]$3s^2 3p^3 3d^4$. At $r_c = 1.1a_0$ we see a ground state crossing to [Ne]$3s^1 3d^8$. Moreover, at $r_c = 1.0a_0$ we see a ground state crossing to [Ne]$3d^9$.

At $r_c = 2.9a_0$ the state [Ne]$3s^2 3p^5 3d^2$ flips below the initial ground state. At $r_c = 2.5a_0$ the state [Ne]$3s^1 3p^6 3d^2$ flips below the initial ground state. At $r_c = 2.4a_0$ the state [Ne]$3s^2 3p^4 3d^3$ flips below the initial ground state. At $r_c = 1.9a_0$ the state [Ne]$3s^2 3p^2 3d^5$

flips below the initial ground state. At $r_c = 1.9a_0$ the state [Ne]$3s^13p^33d^5$ flips below the initial ground state. At $r_c = 1.5a_0$ the state [Ne]$3p^13d^8$ flips below the initial ground state. At $r_c = 1.4a_0$ the state [Ar]$4p^1$ flips below the initial ground state. The ionization energy of the unconfined atom is $\Delta E_0 = 4.45$ eV. The studied configurations, atomic radii and excitation energies in the unconfined calculations are as follows:

configuration	r_ϵ	r_ρ	$r_{\max}$	ΔE
[Ar]$4s^1$	7.93	4.51	4.19	0.00
[Ar]$4p^1$	10.48	3.66	5.75	1.56
[Ar]$3d^1$	14.10	3.64	7.47	2.50
[Ne]$3s^23p^53d^2$	10.04	4.45	1.62	22.23
[Ne]$3s^13p^63d^2$	9.93	4.39	1.56	38.61
[Ne]$3s^23p^43d^3$	8.03	4.59	1.46	41.26
[Ne]$3s^23p^33d^4$	6.99	4.59	1.38	58.77
[Ne]$3s^23p^23d^5$	6.44	4.59	1.34	82.00
[Ne]$3s^13p^33d^5$	6.31	4.51	1.32	90.54
[Ne]$3s^13d^8$	6.44	4.67	1.33	176.38
[Ne]$3p^13d^8$	6.36	4.67	1.31	193.45
[Ne]$3d^9$	5.74	4.59	1.24	219.78

Ca The energies of the low lying configurations of hard-wall confined Ca are shown in fig. S152 for the neutral atom and in fig. S153 for the cation. The ground state of the unconfined Ca is [Ar]$4s^2$. At $r_c = 4.9a_0$ the ground state changes to [Ar]$4s^13d^1$. Furthermore, at $r_c = 4.4a_0$ we see a ground state crossing to [Ar]$3d^2$. At $r_c = 1.1a_0$ we see a ground state crossing to [Ne]$3s^23p^33d^5$. Moreover, at $r_c = 1.0a_0$ we see a ground state crossing to [Ne]$3d^{10}$.

At $r_c = 3.5a_0$ the state [Ar]$3d^14p^1$ flips below the initial ground state. At $r_c = 3.2a_0$ the state [Ne]$3s^23p^53d^3$ flips below the initial ground state. At $r_c = 2.8a_0$ the state [Ne]$3s^23p^43d^4$ flips below the initial ground state. At $r_c = 2.8a_0$ the state [Ne]$3s^13p^63d^3$ flips below the initial ground state. At $r_c = 1.8a_0$ the state [Ne]$3s^13d^9$ flips below the initial ground state. At $r_c = 1.7a_0$ the state [Ne]$3p^13d^9$ flips below the initial ground state. At $r_c = 1.4a_0$ the state [Ar]$4s^14p^1$ flips below the initial ground state. The ionization energy of the unconfined atom is $\Delta E_0 = 6.07$ eV. The studied configurations, atomic radii and excitation energies in the unconfined calculations are as follows:

configuration	r_ϵ	r_ρ	$r_{\max}$	ΔE
[Ar]$4s^2$	7.20	5.17	3.41	0.00
[Ar]$4s^13d^1$	7.85	4.89	3.54	1.72
[Ar]$4s^14p^1$	8.17	5.33	4.16	1.84
[Ar]$3d^14p^1$	9.19	5.02	4.68	3.89
[Ar]$3d^2$	10.34	4.35	1.46	4.37
[Ne]$3s^23p^53d^3$	8.23	4.59	1.32	27.66
[Ne]$3s^13p^63d^3$	8.13	4.51	1.29	46.14
[Ne]$3s^23p^43d^4$	7.03	4.51	1.25	49.62
[Ne]$3s^23p^33d^5$	6.34	4.45	1.21	69.73
[Ne]$3s^13d^9$	5.82	4.51	1.16	207.98
[Ne]$3p^13d^9$	5.77	4.45	1.15	227.03
[Ne]$3d^{10}$	5.42	4.39	1.11	256.02

Sc The energies of the low lying configurations of hard-wall confined Sc are shown in fig. S154 for the neutral atom and in fig. S155 for the cation. The ground state of the unconfined Sc is [Ar]$4s^23d^1$. At $r_c = 5.6a_0$ the ground state changes to [Ar]$4s^13d^2$. Furthermore, at $r_c = 4.4a_0$ we see a ground state crossing to [Ar]$3d^3$. At $r_c = 1.1a_0$ we see a ground state crossing to [Ne]$3s^23p^43d^5$. Moreover, at $r_c = 1.0a_0$ we see a ground state crossing to [Ne]$3s^23d^9$.

At $r_c = 3.5a_0$ the state [Ar]$3d^24p^1$ flips below the initial ground state. At $r_c = 3.1a_0$ the state [Ne]$3s^23p^53d^4$ flips below the initial ground state. At $r_c = 2.7a_0$ the state [Ne]$3s^13p^63d^4$ flips below the initial ground state. At $r_c = 1.9a_0$ the state [Ne]$3s^23p^13d^8$ flips below the initial ground state. At $r_c = 1.7a_0$ the state [Ne]$3s^13d^{10}$ flips below the initial ground state. At $r_c = 1.4a_0$ the state [Ar]$4s^13d^14p^1$ flips below the initial ground state. The ionization energy of the unconfined atom is $\Delta E_0 = 6.42$ eV. The studied configurations, atomic radii and excitation energies in the unconfined calculations are as follows:

configuration	r_ϵ	r_ρ	$r_{\max}$	ΔE
[Ar]$4s^2 3d^1$	6.77	5.17	3.18	0.00
[Ar]$4s^1 3d^2$	6.91	4.89	3.23	0.65
[Ar]$4s^1 3d^1 4p^1$	7.71	5.33	3.82	1.93
[Ar]$3d^3$	8.48	4.51	1.20	3.02
[Ar]$3d^2 4p^1$	8.61	5.02	4.26	3.16
[Ne]$3s^2 3p^5 3d^4$	7.16	4.51	1.14	29.51
[Ne]$3s^1 3p^6 3d^4$	7.08	4.45	1.13	50.10
[Ne]$3s^2 3p^4 3d^5$	6.36	4.39	1.11	54.34
[Ne]$3s^2 3p^1 3d^8$	6.32	4.45	1.08	152.04
[Ne]$3s^2 3d^9$	5.59	4.39	1.04	185.00
[Ne]$3s^1 3d^{10}$	5.41	4.35	1.04	236.89

Ti The energies of the low lying configurations of hard-wall confined Ti are shown in fig. S156 for the neutral atom and in fig. S157 for the cation. The ground state of the unconfined Ti is [Ar]$4s^1 3d^3$. At $r_c = 4.3a_0$ the ground state changes to [Ar]$3d^4$. Furthermore, at $r_c = 1.0a_0$ we see a ground state crossing to [Ne]$3s^2 3p^5 3d^5$.

At $r_c = 2.1a_0$ the state [Ne]$3s^1 3p^6 3d^5$ flips below the initial ground state. At $r_c = 2.0a_0$ the state [Ne]$3s^2 3p^4 3d^6$ flips below the initial ground state. At $r_c = 1.7a_0$ the state [Ne]$3s^2 3p^3 3d^7$ flips below the initial ground state. At $r_c = 1.3a_0$ the state [Ar]$3d^3 4p^1$ flips below the initial ground state. The ionization energy of the unconfined atom is $\Delta E_0 = 6.63$ eV. The studied configurations, atomic radii and excitation energies in the unconfined calculations are as follows:

configuration	r_ϵ	r_ρ	$r_{\max}$	ΔE
[Ar]$4s^1 3d^3$	6.39	4.78	3.02	0.00
[Ar]$4s^2 3d^2$	6.47	5.02	3.02	0.35
[Ar]$3d^4$	7.34	4.45	1.04	2.02
[Ar]$3d^3 4p^1$	8.31	4.89	4.00	2.78
[Ne]$3s^2 3p^5 3d^5$	6.44	4.35	1.01	31.63
[Ne]$3s^1 3p^6 3d^5$	6.37	4.31	1.01	54.37
[Ne]$3s^2 3p^4 3d^6$	13.77	4.11	1.05	63.31
[Ne]$3s^2 3p^3 3d^7$	9.73	4.45	1.05	95.60

V The energies of the low lying configurations of hard-wall confined V are shown in fig. S158 for the neutral atom and in fig. S159 for the cation. The ground state of the unconfined V is [Ar]$4s^1 3d^4$. At $r_c = 4.4a_0$ the ground state changes to [Ar]$3d^5$.

At $r_c = 2.3a_0$ the state [Ne]$3s^2 3p^5 3d^6$ flips below the initial ground state. At $r_c = 2.0a_0$ the state [Ne]$3s^1 3p^6 3d^6$ flips below the initial ground state. At $r_c = 1.8a_0$ the state [Ne]$3s^2 3p^4 3d^7$ flips below the initial ground state. At $r_c = 1.2a_0$ the state [Ar]$3d^4 4p^1$ flips below the initial ground state. The ionization energy of the unconfined atom is $\Delta E_0 = 6.96$ eV. The studied configurations, atomic radii and excitation energies in the unconfined calculations are as follows:

configuration	r_ϵ	r_ρ	$r_{\max}$	ΔE
[Ar]$4s^1 3d^4$	6.04	4.59	2.85	0.00
[Ar]$4s^2 3d^3$	6.24	4.89	2.89	1.28
[Ar]$3d^5$	6.57	4.31	0.94	1.65
[Ar]$3d^4 4p^1$	8.11	4.78	3.81	3.02
[Ne]$3s^2 3p^4 3d^7$	9.27	4.28	0.94	74.53
[Ne]$3s^2 3p^5 3d^6$	Not converged			
[Ne]$3s^1 3p^6 3d^6$	Not converged			

Cr The energies of the low lying configurations of hard-wall confined Cr are shown in fig. S160 for the neutral atom and in fig. S161 for the cation. The ground state of the unconfined Cr is [Ar]$4s^1 3d^5$. At $r_c = 3.5a_0$ the ground state changes to [Ar]$3d^6$.

At $r_c = 2.1a_0$ the state [Ne]$3s^2 3p^5 3d^7$ flips below the initial ground state. At $r_c = 1.8a_0$ the state [Ne]$3s^1 3p^6 3d^7$ flips below the initial ground state. At $r_c = 1.7a_0$ the state [Ne]$3s^2 3p^4 3d^8$ flips below the initial ground state. At $r_c = 1.2a_0$ the state [Ar]$3d^5 4p^1$ flips below the initial ground state. The ionization energy of the unconfined atom is $\Delta E_0 = 7.26$ eV. The studied configurations, atomic radii and excitation energies in the unconfined calculations are as follows:

configuration	r_ϵ	r_ρ	$r_{\max}$	ΔE
[Ar]$4s^1 3d^5$	5.78	4.45	2.72	0.00
[Ar]$4s^2 3d^4$	6.05	4.78	2.78	2.17
[Ar]$3d^5 4p^1$	7.96	4.67	3.67	3.24
[Ne]$3s^2 3p^5 3d^7$	8.55	4.20	0.86	45.31
[Ne]$3s^1 3p^6 3d^7$	8.51	4.18	0.86	72.57
[Ne]$3s^2 3p^4 3d^8$	7.18	4.25	0.85	86.48
[Ar]$3d^6$	Not converged			

Mn The energies of the low lying configurations of hard-wall confined Mn are shown in fig. S162 for the neutral atom and in fig. S163 for the cation. The ground state of the un-

confined Mn is [Ar]$4s^2 3d^5$. At $r_c = 4.7a_0$ the ground state changes to [Ar]$4s^1 3d^6$. Furthermore, at $r_c = 3.6a_0$ we see a ground state crossing to [Ar]$3d^7$.

At $r_c = 2.9a_0$ the state [Ar]$3d^6 4p^1$ flips below the initial ground state. At $r_c = 2.4a_0$ the state [Ne]$3s^2 3p^5 3d^8$ flips below the initial ground state. At $r_c = 2.1a_0$ the state [Ne]$3s^1 3p^6 3d^8$ flips below the initial ground state. At $r_c = 1.2a_0$ the state [Ar]$4s^1 3d^5 4p^1$ flips below the initial ground state. The ionization energy of the unconfined atom is $\Delta E_0 = 7.16$ eV. The studied configurations, atomic radii and excitation energies in the unconfined calculations are as follows:

configuration	r_ϵ	r_ρ	$r_{\max}$	ΔE
[Ar]$4s^2 3d^5$	5.89	4.67	2.69	0.00
[Ar]$4s^1 3d^6$	5.69	4.39	2.61	1.12
[Ar]$4s^1 3d^5 4p^1$	6.88	4.78	3.16	2.16
[Ar]$3d^6 4p^1$	7.80	4.59	3.57	4.56
[Ar]$3d^7$	7.50	4.17	0.79	4.61
[Ne]$3s^2 3p^5 3d^8$	6.66	4.17	0.79	50.29
[Ne]$3s^1 3p^6 3d^8$	6.62	4.16	0.79	79.92

Fe The energies of the low lying configurations of hard-wall confined Fe are shown in fig. S164 for the neutral atom and in fig. S165 for the cation. The ground state of the unconfined Fe is [Ar]$4s^2 3d^6$. At $r_c = 5.8a_0$ the ground state changes to [Ar]$4s^1 3d^7$. Furthermore, at $r_c = 3.7a_0$ we see a ground state crossing to [Ar]$3d^8$.

At $r_c = 2.9a_0$ the state [Ar]$3d^7 4p^1$ flips below the initial ground state. At $r_c = 2.3a_0$ the state [Ne]$3s^2 3p^5 3d^9$ flips below the initial ground state. At $r_c = 2.1a_0$ the state [Ne]$3s^1 3p^6 3d^9$ flips below the initial ground state. At $r_c = 1.2a_0$ the state [Ar]$4s^1 3d^6 4p^1$ flips below the initial ground state. The ionization energy of the unconfined atom is $\Delta E_0 = 7.73$ eV. The studied configurations, atomic radii and excitation energies in the unconfined calculations are as follows:

configuration	r_ϵ	r_ρ	$r_{\max}$	ΔE
[Ar]$4s^2 3d^6$	5.67	4.59	2.53	0.00
[Ar]$4s^1 3d^7$	5.51	4.31	2.53	0.23
[Ar]$4s^1 3d^6 4p^1$	6.75	4.78	3.07	2.64
[Ar]$3d^8$	6.16	4.13	0.73	2.94
[Ar]$3d^7 4p^1$	7.73	4.59	3.50	3.82
[Ne]$3s^2 3p^5 3d^9$	5.79	4.11	0.73	53.37
[Ne]$3s^1 3p^6 3d^9$	5.75	4.08	0.73	85.42

Co The energies of the low lying configurations of hard-wall confined Co are shown in fig. S166 for the neutral atom and in fig. S167 for the cation. The ground state of the unconfined Co is [Ar]$4s^1 3d^8$. At $r_c = 3.9a_0$ the ground state changes to [Ar]$3d^9$.

At $r_c = 1.9a_0$ the state [Ne]$3s^2 3p^5 3d^{10}$ flips below the initial ground state. At $r_c = 1.6a_0$ the state [Ne]$3s^1 3p^6 3d^{10}$ flips below the initial ground state. At $r_c = 1.1a_0$ the state [Ar]$3d^8 4p^1$ flips below the initial ground state. The ionization energy of the unconfined atom is $\Delta E_0 = 7.87$ eV. The studied configurations, atomic radii and excitation energies in the unconfined calculations are as follows:

configuration	r_ϵ	r_ρ	$r_{\max}$	ΔE
[Ar]$4s^1 3d^8$	5.35	4.23	2.45	0.00
[Ar]$4s^2 3d^7$	5.50	4.45	2.41	0.65
[Ar]$3d^9$	5.57	4.08	0.68	1.94
[Ar]$3d^8 4p^1$	7.69	4.51	3.45	3.74
[Ne]$3s^2 3p^5 3d^{10}$	5.29	4.02	0.68	57.24
[Ne]$3s^1 3p^6 3d^{10}$	5.26	4.02	0.68	91.79

Ni The energies of the low lying configurations of hard-wall confined Ni are shown in fig. S168 for the neutral atom and in fig. S169 for the cation. The ground state of the unconfined Ni is [Ar]$4s^1 3d^9$. At $r_c = 4.2a_0$ the ground state changes to [Ar]$3d^{10}$.

At $r_c = 1.4a_0$ the state [Ar]$3d^9 4f^1$ flips below the initial ground state. At $r_c = 1.2a_0$ the state [Ne]$3s^2 3p^5 3d^{10} 4f^1$ flips below the initial ground state. At $r_c = 1.0a_0$ the state [Ar]$3d^9 4p^1$ flips below the initial ground state. The ionization energy of the unconfined atom is $\Delta E_0 = 8.01$ eV. The studied configurations, atomic radii and excitation energies in the unconfined calculations are as follows:

configuration	r_ϵ	r_ρ	$r_{\max}$	ΔE
[Ar]$4s^1 3d^9$	5.22	4.17	2.39	0.00
[Ar]$3d^{10}$	5.25	4.02	0.65	1.18
[Ar]$4s^2 3d^8$	5.35	4.39	2.30	1.52
[Ar]$3d^9 4p^1$	7.67	4.45	3.41	3.87
[Ar]$3d^9 4f^1$	23.76	3.46	16.12	7.05
[Ne]$3s^2 3p^5 3d^{10} 4f^1$	23.76	3.39	16.12	67.89

Cu The energies of the low lying configurations of hard-wall confined Cu are shown in fig. S170 for the neutral atom and in fig. S171 for the cation. The ground state of the unconfined Cu is [Ar]$4s^1 3d^{10}$. At $r_c = 1.4a_0$ the ground state changes to [Ar]$3d^{10}4f^1$.

At $r_c = 1.1a_0$ the state [Ar]$3d^{10}4d^1$ flips below the initial ground state. At $r_c = 1.0a_0$ the state [Ar]$3d^{10}4p^1$ flips below the initial ground state. The ionization energy of the unconfined atom is $\Delta E_0 = 8.14$ eV. The studied configurations, atomic radii and excitation energies in the unconfined calculations are as follows:

configuration	r_ϵ	r_ρ	$r_{\max}$	ΔE
[Ar]$4s^1 3d^{10}$	5.12	4.13	2.32	0.00
[Ar]$4s^2 3d^9$	5.22	4.31	2.21	2.38
[Ar]$3d^{10} 4p^1$	7.66	4.39	3.38	4.01
[Ar]$3d^{10} 4d^1$	15.37	3.45	9.01	6.48
[Ar]$3d^{10} 4f^1$	23.77	3.39	16.12	7.18

Zn The energies of the low lying configurations of hard-wall confined Zn are shown in fig. S172 for the neutral atom and in fig. S173 for the cation. The ground state of the unconfined Zn is [Ar]$4s^2 3d^{10}$. At $r_c = 1.3a_0$ the ground state changes to [Ar]$3d^{10}4f^2$.

At $r_c = 1.3a_0$ the state [Ar]$4s^1 3d^{10} 4f^1$ flips below the initial ground state. At $r_c = 1.2a_0$ the state [Ar]$3d^{10}4d^1 4f^1$ flips below the initial ground state. At $r_c = 1.2a_0$ the state [Ar]$3d^{10}4p^1 4f^1$ flips below the initial ground state. At $r_c = 1.1a_0$ the state [Ar]$4s^1 3d^{10} 4d^1$ flips below the initial ground state. At $r_c = 1.0a_0$ the state [Ar]$4s^1 3d^{10} 4p^1$ flips below the initial ground state. At $r_c = 1.0a_0$ the state [Ar]$3d^{10}4p^2$ flips below the initial ground state. The ionization energy of the unconfined atom is $\Delta E_0 = 9.38$ eV. The studied configurations, atomic radii and excitation energies in the unconfined calculations are as follows:

configuration	r_ϵ	r_ρ	$r_{\max}$	ΔE
[Ar]$4s^2 3d^{10}$	5.11	4.22	2.13	0.00
[Ar]$4s^1 3d^{10} 4p^1$	6.47	4.51	2.83	4.12
[Ar]$4s^1 3d^{10} 4d^1$	14.96	3.83	8.56	7.64
[Ar]$4s^1 3d^{10} 4f^1$	23.71	3.72	16.06	8.41
[Ar]$3d^{10} 4p^2$	6.74	4.89	2.79	9.40
[Ar]$3d^{10} 4p^1 4f^1$	23.58	4.03	15.93	14.49
[Ar]$3d^{10} 4d^1 4f^1$	20.99	3.89	12.59	20.52
[Ar]$3d^{10} 4f^2$	17.46	2.97	9.46	22.55

Ga The energies of the low lying configurations of hard-wall confined Ga are shown in fig. S174 for the neutral atom and in fig. S175 for the cation. The ground state of the unconfined Ga is [Ar]$4s^2 3d^{10} 4p^1$. At $r_c = 1.3a_0$ the ground state changes to [Ar]$4s^1 3d^{10} 4f^2$. Furthermore, at $r_c = 1.2a_0$ we see a ground state crossing to [Ar]$3d^{10}4f^3$.

At $r_c = 1.3a_0$ the state [Ar]$3d^{10}4p^1 4f^2$ flips below the initial ground state. At $r_c = 1.3a_0$ the state [Ar]$4s^2 3d^{10} 4f^1$ flips below the initial ground state. At $r_c = 1.2a_0$ the state [Ar]$3d^{10}4d^1 4f^2$ flips below the initial ground state. At $r_c = 1.1a_0$ the state [Ar]$4s^2 3d^{10} 4d^1$ flips below the initial ground state. At $r_c = 1.0a_0$ the state [Ar]$4s^1 3d^{10} 4p^2$ flips below the initial ground state. The ionization energy of the unconfined atom is $\Delta E_0 = 5.91$ eV. The studied configurations, atomic radii and excitation energies in the unconfined calculations are as follows:

configuration	r_ϵ	r_ρ	$r_{\max}$	ΔE
[Ar]$4s^2 3d^{10} 4p^1$	5.93	4.51	2.56	0.00
[Ar]$4s^2 3d^{10} 5s^1$	10.86	4.01	6.47	2.86
[Ar]$4s^2 3d^{10} 4d^1$	14.74	4.01	8.34	4.16
[Ar]$4s^1 3d^{10} 4p^2$	5.97	4.67	2.44	4.75
[Ar]$4s^2 3d^{10} 4f^1$	23.72	3.83	16.07	4.94
[Ar]$3d^{10} 4p^3$	6.04	4.78	2.41	11.32
[Ar]$4s^1 3d^{10} 4f^2$	17.44	3.45	9.40	21.09
[Ar]$3d^{10} 4p^1 4f^2$	17.38	3.89	9.32	29.10
[Ar]$3d^{10} 4d^1 4f^2$	16.46	5.33	7.70	38.54
[Ar]$3d^{10} 4f^3$	14.50	6.11	6.64	42.56

Ge The energies of the low lying configurations of hard-wall confined Ge are shown in fig. S176 for the neutral atom and in fig. S177 for the cation. The ground state of the unconfined Ge is [Ar]$4s^2 3d^{10} 4p^2$. At $r_c = 1.3a_0$ the ground state changes to [Ar]$4s^2 3d^{10} 4p^1 4f^1$.

Furthermore, at $r_c = 1.2a_0$ we see a ground state crossing to [Ar]$3d^{10}4f^4$.

At $r_c = 1.3a_0$ the state [Ar]$4s^13d^{10}4f^3$ flips below the initial ground state. At $r_c = 1.2a_0$ the state [Ar]$3d^{10}4d^14f^3$ flips below the initial ground state. At $r_c = 1.2a_0$ the state [Ar]$3d^{10}4p^14f^3$ flips below the initial ground state. At $r_c = 1.0a_0$ the state [Ar]$4s^23d^{10}4p^14d^1$ flips below the initial ground state. The ionization energy of the unconfined atom is ΔE_0 = 7.98 eV. The studied configurations, atomic radii and excitation energies in the unconfined calculations are as follows:

configuration	r_ϵ	r_ρ	$r_{\max}$	ΔE
[Ar]$4s^23d^{10}4p^2$	5.54	4.51	2.23	0.00
[Ar]$4s^23d^{10}4p^15s^1$	10.09	4.51	5.82	4.61
[Ar]$4s^13d^{10}4p^3$	5.49	4.59	2.16	5.30
[Ar]$4s^23d^{10}4p^14d^1$	13.94	4.25	7.28	6.07
[Ar]$4s^23d^{10}4p^14f^1$	23.66	4.00	16.02	7.01
[Ar]$4s^13d^{10}4f^3$	14.49	6.06	6.57	42.53
[Ar]$3d^{10}4p^14f^3$	14.46	6.11	6.47	52.35
[Ar]$3d^{10}4d^14f^3$	14.11	6.34	5.63	65.01
[Ar]$3d^{10}4f^4$	12.75	7.10	5.08	71.46

As The energies of the low lying configurations of hard-wall confined As are shown in fig. S178 for the neutral atom and in fig. S179 for the cation. The ground state of the unconfined As is [Ar]$4s^23d^{10}4p^3$. At $r_c = 1.2a_0$ the ground state changes to [Ar]$3d^{10}4f^5$.

At $r_c = 1.2a_0$ the state [Ar]$3d^{10}4d^14f^4$ flips below the initial ground state. At $r_c = 1.2a_0$ the state [Ar]$4s^13d^{10}4f^4$ flips below the initial ground state. At $r_c = 1.2a_0$ the state [Ar]$4s^23d^{10}4f^3$ flips below the initial ground state. At $r_c = 1.2a_0$ the state [Ar]$4s^23d^{10}4p^24f^1$ flips below the initial ground state. At $r_c = 1.0a_0$ the state [Ar]$4s^23d^{10}4p^24d^1$ flips below the initial ground state. The ionization energy of the unconfined atom is ΔE_0 = 9.93 eV. The studied configurations, atomic radii and excitation energies in the unconfined calculations are as follows:

configuration	r_ϵ	r_ρ	$r_{\max}$	ΔE
[Ar]$4s^23d^{10}4p^3$	5.14	4.39	2.00	0.00
[Ar]$4s^23d^{10}4p^25s^1$	9.55	4.67	5.37	6.31
[Ar]$4s^23d^{10}4p^24d^1$	13.45	4.31	6.19	7.90
[Ar]$4s^13d^{10}4p^4$	5.30	4.51	1.98	8.84
[Ar]$4s^23d^{10}4p^24f^1$	23.66	4.07	16.02	8.96
[Ar]$4s^23d^{10}4f^3$	14.46	6.06	6.53	41.44
[Ar]$4s^13d^{10}4f^4$	12.76	6.84	4.98	73.46
[Ar]$3d^{10}4d^14f^4$	12.62	6.84	4.43	100.71
[Ar]$3d^{10}4f^5$	11.58	6.84	4.09	109.90

Se The energies of the low lying configurations of hard-wall confined Se are shown in fig. S180 for the neutral atom and in fig. S181 for the cation. The ground state of the unconfined Se is [Ar]$4s^23d^{10}4p^4$. At $r_c = 1.2a_0$ the ground state changes to [Ar]$4s^23d^{10}4f^4$. Furthermore, at $r_c = 1.1a_0$ we see a ground state crossing to [Ar]$3d^{10}4f^6$.

At $r_c = 1.2a_0$ the state [Ar]$4s^13d^{10}4f^5$ flips below the initial ground state. At $r_c = 1.2a_0$ the state [Ar]$4s^23d^{10}4p^14f^3$ flips below the initial ground state. At $r_c = 1.2a_0$ the state [Ar]$4s^23d^{10}4p^34f^1$ flips below the initial ground state. At $r_c = 1.1a_0$ the state [Ar]$3d^{10}4d^14f^5$ flips below the initial ground state. At $r_c = 1.0a_0$ the state [Ar]$4s^23d^{10}4p^34d^1$ flips below the initial ground state. The ionization energy of the unconfined atom is ΔE_0 = 9.55 eV. The studied configurations, atomic radii and excitation energies in the unconfined calculations are as follows:

configuration	r_ϵ	r_ρ	$r_{\max}$	ΔE
[Ar]$4s^23d^{10}4p^4$	4.92	4.31	1.82	0.00
[Ar]$4s^23d^{10}4p^35s^1$	9.12	4.78	5.02	5.72
[Ar]$4s^23d^{10}4p^34d^1$	13.16	4.26	4.53	7.44
[Ar]$4s^23d^{10}4p^34f^1$	23.68	3.97	16.02	8.59
[Ar]$4s^13d^{10}4p^5$	4.97	4.35	1.82	10.18
[Ar]$4s^23d^{10}4p^14f^3$	14.46	5.98	6.46	47.28
[Ar]$4s^23d^{10}4f^4$	12.75	6.84	4.93	72.30
[Ar]$4s^13d^{10}4f^5$	11.63	6.84	3.98	112.08
[Ar]$3d^{10}4d^14f^5$	11.58	6.58	3.63	143.96
[Ar]$3d^{10}4f^6$	10.75	6.53	3.41	156.09

Br The energies of the low lying configurations of hard-wall confined Br are shown in fig. S182 for the neutral atom and in fig. S183 for the cation. The ground state of the un-

confined Br is [Ar]$4s^2 3d^{10} 4p^5$. At $r_c = 1.2a_0$ the ground state changes to [Ar]$4s^2 3d^{10} 4p^3 4f^2$. Furthermore, at $r_c = 1.1a_0$ we see a ground state crossing to [Ar]$3d^{10} 4f^7$.

At $r_c = 1.2a_0$ the state [Ar]$4s^2 3d^{10} 4p^2 4f^3$ flips below the initial ground state. At $r_c = 1.2a_0$ the state [Ar]$4s^2 3d^{10} 4p^4 4f^1$ flips below the initial ground state. At $r_c = 1.1a_0$ the state [Ar]$4s^1 3d^{10} 4f^6$ flips below the initial ground state. At $r_c = 1.1a_0$ the state [Ar]$3d^{10} 4p^1 4f^6$ flips below the initial ground state. At $r_c = 1.0a_0$ the state [Ar]$4s^2 3d^{10} 4p^4 4d^1$ flips below the initial ground state. The ionization energy of the unconfined atom is $\Delta E_0 = 11.88$ eV. The studied configurations, atomic radii and excitation energies in the unconfined calculations are as follows:

configuration	r_ϵ	r_ρ	$r_{\max}$	ΔE
[Ar]$4s^2 3d^{10} 4p^5$	4.65	4.17	1.68	0.00
[Ar]$4s^2 3d^{10} 4p^4 5s^1$	8.78	4.78	4.78	7.88
[Ar]$4s^2 3d^{10} 4p^4 4d^1$	13.22	4.17	4.39	9.77
[Ar]$4s^2 3d^{10} 4p^4 4f^1$	23.71	3.89	16.07	10.92
[Ar]$4s^1 3d^{10} 4p^6$	4.66	4.20	1.67	11.54
[Ar]$4s^2 3d^{10} 4p^3 4f^2$	17.37	3.78	9.29	27.85
[Ar]$4s^2 3d^{10} 4p^2 4f^3$	14.47	5.98	6.43	52.93
[Ar]$4s^1 3d^{10} 4f^6$	10.83	6.58	3.30	161.10
[Ar]$3d^{10} 4p^1 4f^6$	10.85	6.58	3.24	175.79
[Ar]$3d^{10} 4f^7$	10.14	6.27	2.93	212.77

Kr The energies of the low lying configurations of hard-wall confined Kr are shown in fig. S184 for the neutral atom and in fig. S185 for the cation. The ground state of the unconfined Kr is [Ar]$4s^2 3d^{10} 4p^6$. At $r_c = 1.1a_0$ the ground state changes to [Ar]$4s^1 3d^{10} 4f^7$. Furthermore, at $r_c = 1.0a_0$ we see a ground state crossing to [Ar]$3d^{10} 4f^8$.

At $r_c = 1.1a_0$ the state [Ar]$3d^{10} 4p^1 4f^7$ flips below the initial ground state. At $r_c = 1.1a_0$ the state [Ar]$4s^2 3d^{10} 4p^5 4f^1$ flips below the initial ground state. At $r_c = 1.1a_0$ the state [Ar]$4s^2 3d^{10} 4p^4 4f^2$ flips below the initial ground state. The ionization energy of the unconfined atom is $\Delta E_0 = 14.10$ eV. The studied configurations, atomic radii and excitation energies in the unconfined calculations are as follows:

configuration	r_ϵ	r_ρ	$r_{\max}$	ΔE
[Ar]$4s^2 3d^{10} 4p^6$	4.39	4.08	1.56	0.00
[Ar]$4s^2 3d^{10} 4p^5 5s^1$	8.54	4.67	4.61	9.98
[Ar]$4s^2 3d^{10} 4p^5 4d^1$	13.42	4.08	6.31	12.02
[Ar]$4s^2 3d^{10} 4p^5 4f^1$	23.73	3.80	16.08	13.13
[Ar]$4s^1 3d^{10} 4p^6 4d^1$	13.30	4.11	4.00	25.47
[Ar]$4s^2 3d^{10} 4p^4 4f^2$	17.37	3.66	9.30	33.20
[Ar]$4s^1 3d^{10} 4f^7$	10.24	6.34	2.81	220.91
[Ar]$3d^{10} 4p^1 4f^7$	10.26	6.34	2.77	237.16
[Ar]$3d^{10} 4f^8$	Not converged			

Rb The energies of the low lying configurations of hard-wall confined Rb are shown in fig. S186 for the neutral atom and in fig. S187 for the cation. The ground state of the unconfined Rb is [Kr]$5s^1$. At $r_c = 4.9a_0$ the ground state changes to [Kr]$4d^1$. Furthermore, at $r_c = 1.2a_0$ we see a ground state crossing to [Kr]$4f^1$. At $r_c = 1.1a_0$ we see a ground state crossing to [Ar]$4s^1 3d^{10} 4f^8$. Moreover, at $r_c = 1.0a_0$ we see a ground state crossing to [Ar]$3d^{10} 4f^9$.

At $r_c = 3.0a_0$ the state [Ar]$4s^2 3d^{10} 4p^5 4d^2$ flips below the initial ground state. At $r_c = 2.0a_0$ the state [Ar]$4s^2 3d^{10} 4p^5 4f^2$ flips below the initial ground state. At $r_c = 1.3a_0$ the state [Ar]$3d^{10} 4p^1 4f^8$ flips below the initial ground state. At $r_c = 1.1a_0$ the state [Kr]$5p^1$ flips below the initial ground state. The ionization energy of the unconfined atom is $\Delta E_0 = 4.22$ eV. The studied configurations, atomic radii and excitation energies in the unconfined calculations are as follows:

configuration	r_ϵ	r_ρ	$r_{\max}$	ΔE
[Kr]$5s^1$	8.35	4.67	4.47	0.00
[Kr]$5p^1$	11.04	4.01	6.22	1.47
[Kr]$4d^1$	13.63	3.98	6.76	2.20
[Kr]$4f^1$	23.75	3.72	16.10	3.26
[Ar]$4s^2 3d^{10} 4p^5 4d^2$	9.61	4.78	2.44	18.87
[Ar]$4s^2 3d^{10} 4p^5 4f^2$	17.39	3.55	9.33	26.31
[Ar]$4s^1 3d^{10} 4f^8$	22.83	5.69	15.03	281.91
[Ar]$3d^{10} 4p^1 4f^8$	22.84	5.69	15.04	299.69
[Ar]$3d^{10} 4f^9$	Not converged			

Sr The energies of the low lying configurations of hard-wall confined Sr are shown in fig. S188 for the neutral atom and in fig. S189 for the cation. The ground state of the uncon-

fined Sr is [Kr]$5s^2$. At $r_c = 5.3a_0$ the ground state changes to [Kr]$5s^1 4d^1$. Furthermore, at $r_c = 4.9a_0$ we see a ground state crossing to [Kr]$4d^2$. At $r_c = 1.2a_0$ we see a ground state crossing to [Kr]$4f^2$. Moreover, at $r_c = 1.0a_0$ we see a ground state crossing to [Ar]$3d^{10}4f^{10}$.

At $r_c = 3.6a_0$ the state [Kr]$4d^1 5p^1$ flips below the initial ground state. At $r_c = 3.5a_0$ the state [Kr]$4d^1 4f^1$ flips below the initial ground state. At $r_c = 3.5a_0$ the state [Ar]$4s^2 3d^{10} 4p^5 4d^3$ flips below the initial ground state. At $r_c = 1.5a_0$ the state [Ar]$4s^2 3d^{10} 4f^8$ flips below the initial ground state. At $r_c = 1.4a_0$ the state [Ar]$4s^1 3d^{10} 4f^9$ flips below the initial ground state. At $r_c = 1.1a_0$ the state [Kr]$5s^1 5p^1$ flips below the initial ground state. The ionization energy of the unconfined atom is $\Delta E_0 = 5.62$ eV. The studied configurations, atomic radii and excitation energies in the unconfined calculations are as follows:

configuration	r_ϵ	r_ρ	$r_{\max}$	ΔE
[Kr]$5s^2$	7.72	5.50	3.77	0.00
[Kr]$5s^1 4d^1$	8.14	5.17	3.83	1.61
[Kr]$5s^1 5p^1$	8.79	5.69	4.64	1.71
[Kr]$4d^1 5p^1$	9.58	5.17	5.03	3.68
[Kr]$4d^2$	9.70	4.78	2.28	3.85
[Kr]$4d^1 4f^1$	23.56	4.07	15.89	6.07
[Kr]$4f^2$	17.40	3.55	9.36	11.40
[Ar]$4s^2 3d^{10} 4p^5 4d^3$	7.90	4.89	2.03	23.48
[Ar]$4s^2 3d^{10} 4f^8$	22.46	5.69	14.69	274.87
[Ar]$4s^1 3d^{10} 4f^9$	17.46	5.90	2.63	347.81
[Ar]$3d^{10} 4f^{10}$	14.70	5.73	2.17	425.81

Y The energies of the low lying configurations of hard-wall confined Y are shown in fig. S190 for the neutral atom and in fig. S191 for the cation. The ground state of the unconfined Y is [Kr]$5s^2 4d^1$. At $r_c = 6.1a_0$ the ground state changes to [Kr]$5s^1 4d^2$. Furthermore, at $r_c = 4.9a_0$ we see a ground state crossing to [Kr]$4d^3$. At $r_c = 1.2a_0$ we see a ground state crossing to [Kr]$4d^2 4f^1$. Moreover, at $r_c = 1.1a_0$ we see a ground state crossing to [Kr]$4d^1 4f^2$. At $r_c = 1.0a_0$ we see a ground state crossing to [Ar]$4s^2 3d^{10} 4f^9$.

At $r_c = 3.6a_0$ the state [Kr]$4d^2 5p^1$ flips below the initial ground state. At $r_c = 3.4a_0$ the state [Ar]$4s^2 3d^{10} 4p^5 4d^4$ flips below the initial ground state. At $r_c = 1.5a_0$ the state [Ar]$4s^2 3d^{10} 4p^1 4f^8$ flips below the initial ground state. At $r_c = 1.3a_0$ the state [Ar]$4s^1 3d^{10} 4f^{10}$ flips below the initial ground state. At $r_c = 1.1a_0$ the state [Kr]$5s^1 4d^1 5p^1$ flips below the initial ground state. The ionization energy of the unconfined atom is $\Delta E_0 = 6.04$ eV. The studied configurations, atomic radii and excitation energies in the unconfined calculations are as follows:

configuration	r_ϵ	r_ρ	$r_{\max}$	ΔE
[Kr]$5s^2 4d^1$	7.23	5.33	3.48	0.00
[Kr]$5s^1 4d^2$	7.22	5.17	3.50	0.57
[Kr]$5s^1 4d^1 5p^1$	8.18	5.50	4.21	1.78
[Kr]$4d^3$	7.91	4.89	1.90	2.40
[Kr]$4d^2 5p^1$	8.90	5.33	4.54	3.01
[Kr]$4d^2 4f^1$	23.54	4.11	15.89	5.66
[Kr]$4d^1 4f^2$	17.36	3.78	9.28	12.95
[Ar]$4s^2 3d^{10} 4p^5 4d^4$	6.91	4.89	1.78	24.62
[Ar]$4s^2 3d^{10} 4p^1 4f^8$	22.68	5.69	14.88	274.50
[Ar]$4s^1 3d^{10} 4f^{10}$	15.07	5.90	2.25	416.89
[Ar]$4s^2 3d^{10} 4f^9$		Not converged		

Zr The energies of the low lying configurations of hard-wall confined Zr are shown in fig. S192 for the neutral atom and in fig. S193 for the cation. The ground state of the unconfined Zr is [Kr]$5s^1 4d^3$. At $r_c = 5.1a_0$ the ground state changes to [Kr]$4d^4$. Furthermore, at $r_c = 1.1a_0$ we see a ground state crossing to [Kr]$4d^2 4f^2$. At $r_c = 1.0a_0$ we see a ground state crossing to [Ar]$4s^2 3d^{10} 4p^3 4f^7$.

At $r_c = 2.8a_0$ the state [Ar]$4s^2 3d^{10} 4p^5 4d^5$ flips below the initial ground state. At $r_c = 2.6a_0$ the state [Kr]$4d^3 4f^1$ flips below the initial ground state. At $r_c = 1.2a_0$ the state [Ar]$4s^2 3d^{10} 4p^2 4f^8$ flips below the initial ground state. At $r_c = 1.2a_0$ the state [Ar]$4s^2 3d^{10} 4p^1 4f^9$ flips below the initial ground state. At $r_c = 1.0a_0$ the state [Kr]$4d^3 5p^1$ flips below the initial ground state. The ionization energy of the unconfined atom is $\Delta E_0 = 6.49$ eV. The studied configurations, atomic radii and excitation energies in the unconfined calculations are as follows:

configuration	r_ϵ	r_ρ	$r_{\max}$	ΔE
$[Kr]5s^14d^3$	6.68	5.02	3.27	0.00
$[Kr]5s^24d^2$	6.88	5.33	3.30	0.54
$[Kr]4d^4$	6.87	4.78	1.68	1.28
$[Kr]4d^35p^1$	8.55	5.17	4.25	2.70
$[Kr]4d^34f^1$	23.57	4.11	15.92	5.52
$[Kr]4d^24f^2$	17.35	3.89	9.26	14.61
$[Ar]4s^23d^{10}4p^54d^5$	6.24	4.67	1.61	25.90
$[Ar]4s^23d^{10}4p^34f^7$	10.64	6.34	2.48	205.13
$[Ar]4s^23d^{10}4p^24f^8$	22.74	5.50	15.01	269.06
$[Ar]4s^23d^{10}4p^14f^9$	17.40	5.69	2.50	336.16

Nb The energies of the low lying configurations of hard-wall confined Nb are shown in fig. S194 for the neutral atom and in fig. S195 for the cation. The ground state of the unconfined Nb is $[Kr]5s^14d^4$. At $r_c = 5.6a_0$ the ground state changes to $[Kr]4d^5$. Furthermore, at $r_c = 1.1a_0$ we see a ground state crossing to $[Kr]4f^5$.

At $r_c = 2.6a_0$ the state $[Ar]4s^23d^{10}4p^54d^6$ flips below the initial ground state. At $r_c = 2.5a_0$ the state $[Kr]4d^44f^1$ flips below the initial ground state. At $r_c = 2.0a_0$ the state $[Kr]4d^34f^2$ flips below the initial ground state. At $r_c = 1.4a_0$ the state $[Ar]4s^23d^{10}4p^54f^6$ flips below the initial ground state. At $r_c = 1.3a_0$ the state $[Ar]4s^23d^{10}4p^44f^7$ flips below the initial ground state. At $r_c = 1.0a_0$ the state $[Kr]4d^45p^1$ flips below the initial ground state. The ionization energy of the unconfined atom is $\Delta E_0 = 6.80$ eV. The studied configurations, atomic radii and excitation energies in the unconfined calculations are as follows:

configuration	r_ϵ	r_ρ	$r_{\max}$	ΔE
$[Kr]5s^14d^4$	6.31	4.89	3.11	0.00
$[Kr]4d^5$	6.18	4.59	1.53	0.65
$[Kr]5s^24d^3$	6.63	5.17	3.17	1.67
$[Kr]4d^45p^1$	8.33	5.02	4.05	2.91
$[Kr]4d^44f^1$	23.61	4.07	15.95	5.83
$[Kr]4d^34f^2$	17.35	3.78	9.26	16.63
$[Ar]4s^23d^{10}4p^54d^6$	6.62	4.59	1.49	30.79
$[Kr]4f^5$	11.82	6.58	3.79	74.84
$[Ar]4s^23d^{10}4p^54f^6$	11.17	6.34	3.02	134.31
$[Ar]4s^23d^{10}4p^44f^7$	10.68	6.34	2.47	195.42

Mo The energies of the low lying configurations of hard-wall confined Mo are shown in fig. S196 for the neutral atom and in fig. S197 for the cation. The ground state of the unconfined Mo is $[Kr]5s^14d^5$. At $r_c = 4.2a_0$ the ground state changes to $[Kr]4d^6$. Furthermore, at $r_c = 1.1a_0$ we see a ground state crossing to $[Kr]4d^54f^1$. At $r_c = 1.0a_0$ we see a ground state crossing to $[Kr]4f^6$.

At $r_c = 2.4a_0$ the state $[Ar]4s^23d^{10}4p^54d^7$ flips below the initial ground state. At $r_c = 1.9a_0$ the state $[Kr]4d^44f^2$ flips below the initial ground state. At $r_c = 1.4a_0$ the state $[Kr]4d^14f^5$ flips below the initial ground state. At $r_c = 1.3a_0$ the state $[Ar]4s^23d^{10}4p^54f^7$ flips below the initial ground state. At $r_c = 1.0a_0$ the state $[Kr]4d^55p^1$ flips below the initial ground state. The ionization energy of the unconfined atom is $\Delta E_0 = 7.05$ eV. The studied configurations, atomic radii and excitation energies in the unconfined calculations are as follows:

configuration	r_ϵ	r_ρ	$r_{\max}$	ΔE
$[Kr]5s^14d^5$	6.04	4.78	2.98	0.00
$[Kr]4d^6$	6.13	4.51	1.40	2.68
$[Kr]5s^24d^4$	6.44	5.02	3.07	2.80
$[Kr]4d^55p^1$	8.19	4.89	3.90	3.08
$[Kr]4d^54f^1$	23.65	3.97	16.00	6.08
$[Kr]4d^44f^2$	17.36	3.78	9.27	18.51
$[Ar]4s^23d^{10}4p^54d^7$	5.93	4.51	1.38	35.88
$[Kr]4d^14f^5$	11.91	6.58	3.68	84.70
$[Kr]4f^6$	11.17	6.34	3.04	112.65
$[Ar]4s^23d^{10}4p^54f^7$	10.71	6.34	2.48	179.85

Tc The energies of the low lying configurations of hard-wall confined Tc are shown in fig. S198 for the neutral atom and in fig. S199 for the cation. The ground state of the unconfined Tc is $[Kr]5s^14d^6$. At $r_c = 4.6a_0$ the ground state changes to $[Kr]4d^7$. Furthermore, at $r_c = 1.0a_0$ we see a ground state crossing to $[Kr]4f^7$.

At $r_c = 2.3a_0$ the state $[Kr]4d^64f^1$ flips below the initial ground state. At $r_c = 2.3a_0$ the state $[Ar]4s^23d^{10}4p^54d^8$ flips below the initial ground state. At $r_c = 1.9a_0$ the state $[Kr]4d^54f^2$ flips below the initial ground state. At $r_c = 1.4a_0$ the state $[Kr]4d^24f^5$ flips below the initial ground state. At $r_c = 1.3a_0$ the state $[Kr]4d^14f^6$ flips below the initial ground state.

At $r_c = 1.0a_0$ the state [Kr]$4d^6 5p^1$ flips below the initial ground state. The ionization energy of the unconfined atom is $\Delta E_0 = 7.21$ eV. The studied configurations, atomic radii and excitation energies in the unconfined calculations are as follows:

configuration	r_ϵ	r_ρ	$r_{\max}$	ΔE
[Kr]$5s^1 4d^6$	5.89	4.67	2.88	0.00
[Kr]$5s^2 4d^5$	6.29	4.89	2.98	0.61
[Kr]$4d^7$	5.68	4.39	1.30	1.56
[Kr]$4d^6 5p^1$	8.07	4.78	3.81	3.20
[Kr]$4d^6 4f^1$	23.67	3.92	16.03	6.24
[Kr]$4d^5 4f^2$	17.37	3.66	9.29	16.98
[Ar]$4s^2 3d^{10} 4p^5 4d^8$	5.47	4.35	1.29	37.76
[Kr]$4d^2 4f^5$	11.98	6.58	3.62	91.08
[Kr]$4d^1 4f^6$	11.29	6.34	2.93	122.19
[Kr]$4f^7$	10.73	6.34	2.49	155.17

Ru The energies of the low lying configurations of hard-wall confined Ru are shown in fig. S200 for the neutral atom and in fig. S201 for the cation. The ground state of the unconfined Ru is [Kr]$5s^1 4d^7$. At $r_c = 5.7a_0$ the ground state changes to [Kr]$4d^8$. Furthermore, at $r_c = 1.0a_0$ we see a ground state crossing to [Kr]$4d^2 4f^6$.

At $r_c = 2.3a_0$ the state [Kr]$4d^7 4f^1$ flips below the initial ground state. At $r_c = 2.3a_0$ the state [Ar]$4s^2 3d^{10} 4p^5 4d^9$ flips below the initial ground state. At $r_c = 1.4a_0$ the state [Kr]$4d^3 4f^5$ flips below the initial ground state. At $r_c = 1.3a_0$ the state [Kr]$4d^1 4f^7$ flips below the initial ground state. The ionization energy of the unconfined atom is $\Delta E_0 = 7.29$ eV. The studied configurations, atomic radii and excitation energies in the unconfined calculations are as follows:

configuration	r_ϵ	r_ρ	$r_{\max}$	ΔE
[Kr]$5s^1 4d^7$	5.76	4.59	2.81	0.00
[Kr]$4d^8$	5.31	4.28	1.22	0.45
[Kr]$5s^2 4d^6$	6.08	4.89	2.83	1.70
[Kr]$4d^7 5p^1$	8.04	4.78	3.75	3.28
[Kr]$4d^7 4f^1$	23.70	3.83	16.06	6.32
[Ar]$4s^2 3d^{10} 4p^5 4d^9$	5.12	4.22	1.21	39.65
[Kr]$4d^3 4f^5$	12.04	6.58	3.59	97.25
[Kr]$4d^2 4f^6$	11.39	6.34	2.84	131.56
[Kr]$4d^1 4f^7$	10.87	6.11	2.37	167.85

Rh The energies of the low lying configurations of hard-wall confined Rh are shown in fig. S202 for the neutral atom and in fig. S203 for the cation. The ground state of the unconfined Rh is [Kr]$4d^9$. At $r_c = 1.0a_0$ the ground state changes to [Kr]$4d^5 4f^4$.

At $r_c = 1.0a_0$ the state [Kr]$4d^6 4f^3$ flips below the initial ground state. At $r_c = 1.0a_0$ the state [Kr]$4d^4 4f^5$ flips below the initial ground state. At $r_c = 1.0a_0$ the state [Kr]$4d^8 4f^1$ flips below the initial ground state. The ionization energy of the unconfined atom is $\Delta E_0 = 7.98$ eV. The studied configurations, atomic radii and excitation energies in the unconfined calculations are as follows:

configuration	r_ϵ	r_ρ	$r_{\max}$	ΔE
[Kr]$4d^9$	5.01	4.16	1.15	0.00
[Kr]$5s^1 4d^8$	5.65	4.45	2.74	0.65
[Kr]$5s^2 4d^7$	5.91	4.78	2.71	3.47
[Kr]$4d^8 5p^1$	8.04	4.67	3.71	3.99
[Kr]$4d^8 4f^1$	23.72	3.77	16.08	7.02
[Ar]$4s^2 3d^{10} 4p^5 4d^{10}$	4.84	4.13	1.14	42.22
[Kr]$4d^6 4f^3$	14.49	5.91	6.41	42.81
[Kr]$4d^5 4f^4$	12.97	6.58	4.73	69.23
[Kr]$4d^4 4f^5$	12.08	6.58	3.59	103.92

Pd The energies of the low lying configurations of hard-wall confined Pd are shown in fig. S204 for the neutral atom and in fig. S205 for the cation. The ground state of the unconfined Pd is [Kr]$4d^{10}$. We do not observe any ground state crossing for Pd in the considered confinement radii.

The ionization energy of the unconfined atom is $\Delta E_0 = 9.09$ eV. The studied configurations, atomic radii and excitation energies in the unconfined calculations are as follows:

configuration	r_ϵ	r_ρ	$r_{\max}$	ΔE
[Kr]$4d^{10}$	4.75	4.08	1.09	0.00
[Kr]$5s^1 4d^9$	5.57	4.39	2.68	1.73
[Kr]$4d^9 5p^1$	8.07	4.59	3.69	5.13
[Kr]$5s^2 4d^8$	5.78	4.67	2.61	5.69
[Kr]$4d^9 4f^1$	23.74	3.72	16.10	8.12
[Kr]$4d^8 4f^2$	17.40	3.55	9.35	24.39
[Ar]$4s^2 3d^{10} 4p^5 4d^{10} 4f^1$	23.74	3.72	16.10	54.04

Ag The energies of the low lying configurations of hard-wall confined Ag are shown in

fig. S206 for the neutral atom and in fig. S207 for the cation. The ground state of the unconfined Ag is [Kr]$5s^1 4d^{10}$. At $r_c = 2.1a_0$ the ground state changes to [Kr]$4d^{10}4f^1$.

At $r_c = 1.7a_0$ the state [Kr]$4d^9 4f^2$ flips below the initial ground state. At $r_c = 1.5a_0$ the state [Kr]$4d^8 4f^3$ flips below the initial ground state. The ionization energy of the unconfined atom is ΔE_0 = 7.37 eV. The studied configurations, atomic radii and excitation energies in the unconfined calculations are as follows:

configuration	r_ϵ	r_ρ	$r_{\max}$	ΔE
[Kr]$5s^1 4d^{10}$	5.50	4.31	2.64	0.00
[Kr]$4d^{10}5p^1$	8.10	4.51	3.68	3.44
[Kr]$5s^2 4d^9$	5.67	4.59	2.52	5.12
[Kr]$4d^{10}4f^1$	23.75	3.66	16.11	6.41
[Kr]$4d^9 4f^2$	17.41	3.45	9.37	24.38
[Kr]$4d^8 4f^3$	14.48	5.98	6.46	50.47

Cd The energies of the low lying configurations of hard-wall confined Cd are shown in fig. S208 for the neutral atom and in fig. S209 for the cation. The ground state of the unconfined Cd is [Kr]$5s^2 4d^{10}$. At $r_c = 2.0a_0$ the ground state changes to [Kr]$4d^{10}4f^2$.

At $r_c = 2.0a_0$ the state [Kr]$5s^1 4d^{10}4f^1$ flips below the initial ground state. At $r_c = 1.8a_0$ the state [Kr]$4d^9 4f^3$ flips below the initial ground state. At $r_c = 1.8a_0$ the state [Kr]$4d^{10}5p^1 4f^1$ flips below the initial ground state. At $r_c = 1.6a_0$ the state [Kr]$4d^8 4f^4$ flips below the initial ground state. At $r_c = 1.6a_0$ the state [Ar]$4s^2 3d^{10}4p^5 4d^{10}4f^3$ flips below the initial ground state. The ionization energy of the unconfined atom is ΔE_0 = 8.51 eV. The studied configurations, atomic radii and excitation energies in the unconfined calculations are as follows:

configuration	r_ϵ	r_ρ	$r_{\max}$	ΔE
[Kr]$5s^2 4d^{10}$	5.58	4.51	2.44	0.00
[Kr]$5s^1 4d^{10}5p^1$	6.88	4.78	3.16	3.52
[Kr]$5s^1 4d^{10}5d^1$	14.95	4.08	8.55	6.76
[Kr]$5s^1 4d^{10}4f^1$	23.65	3.97	16.00	7.54
[Kr]$4d^{10}5p^2$	7.20	5.02	3.13	7.97
[Kr]$4d^{10}5p^1 4f^1$	23.48	4.21	15.83	12.67
[Kr]$4d^{10}4f^2$	17.42	3.34	9.39	19.77
[Kr]$4d^9 4f^3$	14.48	5.98	6.49	48.16
[Kr]$4d^8 4f^4$	12.90	6.84	4.82	83.82
[Ar]$4s^2 3d^{10}4p^5 4d^{10}4f^3$	14.48	5.98	6.49	101.89

In The energies of the low lying configurations of hard-wall confined In are shown in fig. S210 for the neutral atom and in fig. S211 for the cation. The ground state of the unconfined In is [Kr]$5s^2 4d^{10}5p^1$. At $r_c = 2.2a_0$ the ground state changes to [Kr]$5s^2 4d^{10}4f^1$. Furthermore, at $r_c = 2.0a_0$ we see a ground state crossing to [Kr]$4d^{10}4f^3$.

At $r_c = 2.1a_0$ the state [Kr]$5s^1 4d^{10}4f^2$ flips below the initial ground state. At $r_c = 2.0a_0$ the state [Kr]$4d^{10}5p^1 4f^2$ flips below the initial ground state. At $r_c = 1.9a_0$ the state [Kr]$4d^9 4f^4$ flips below the initial ground state. At $r_c = 1.7a_0$ the state [Kr]$4d^8 4f^5$ flips below the initial ground state. At $r_c = 1.7a_0$ the state [Ar]$4s^2 3d^{10}4p^5 4d^{10}4f^4$ flips below the initial ground state. At $r_c = 1.0a_0$ the state [Kr]$5s^2 4d^{10}5d^1$ flips below the initial ground state. The ionization energy of the unconfined atom is ΔE_0 = 5.59 eV. The studied configurations, atomic radii and excitation energies in the unconfined calculations are as follows:

configuration	r_ϵ	r_ρ	$r_{\max}$	ΔE
[Kr]$5s^2 4d^{10}5p^1$	6.36	4.78	2.90	0.00
[Kr]$5s^2 4d^{10}6s^1$	11.60	4.29	7.06	2.75
[Kr]$5s^2 4d^{10}5d^1$	14.62	4.25	8.22	3.82
[Kr]$5s^1 4d^{10}5p^2$	6.45	5.02	2.80	3.94
[Kr]$5s^2 4d^{10}4f^1$	23.65	4.09	16.01	4.62
[Kr]$4d^{10}5p^3$	6.55	5.17	2.77	9.33
[Kr]$5s^1 4d^{10}4f^2$	17.37	3.78	9.30	18.42
[Kr]$4d^{10}5p^1 4f^2$	17.28	4.24	9.14	25.01
[Kr]$4d^{10}4f^3$	14.48	5.98	6.51	36.02
[Kr]$4d^9 4f^4$	12.88	6.84	4.85	74.61
[Kr]$4d^8 4f^5$	12.03	6.58	3.72	118.75
[Ar]$4s^2 3d^{10}4p^5 4d^{10}4f^4$	12.88	6.84	4.84	132.26

Sn The energies of the low lying configurations of hard-wall confined Sn are shown in fig. S212 for the neutral atom and in fig. S213 for the cation. The ground state of the unconfined Sn is [Kr]$5s^2 4d^{10}5p^2$. At $r_c = 2.2a_0$ the ground state changes to [Kr]$5s^2 4d^{10}4f^2$. Furthermore, at $r_c = 1.9a_0$ we see a ground state crossing to [Kr]$4d^{10}4f^4$.

At $r_c = 2.2a_0$ the state [Kr]$5s^2 4d^{10}5p^1 4f^1$ flips below the initial ground state. At $r_c = 2.1a_0$ the state [Kr]$5s^1 4d^{10}4f^3$ flips below the initial ground state. At $r_c = 1.9a_0$ the state

[Kr]$4d^9 4f^5$ flips below the initial ground state. At $r_c = 1.8a_0$ the state [Kr]$4d^8 4f^6$ flips below the initial ground state. At $r_c = 1.8a_0$ the state [Ar]$4s^2 3d^{10} 4p^5 4d^{10} 4f^5$ flips below the initial ground state. The ionization energy of the unconfined atom is $\Delta E_0 = 7.37$ eV. The studied configurations, atomic radii and excitation energies in the unconfined calculations are as follows:

configuration	r_ϵ	r_ρ	$r_{\max}$	ΔE
[Kr]$5s^2 4d^{10} 5p^2$	6.04	4.89	2.60	0.00
[Kr]$5s^2 4d^{10} 5p^1 6s^1$	10.85	4.67	6.41	4.26
[Kr]$5s^1 4d^{10} 5p^3$	6.01	4.89	2.53	4.27
[Kr]$5s^2 4d^{10} 5p^1 5d^1$	13.44	4.67	6.15	5.38
[Kr]$5s^2 4d^{10} 5p^1 4f^1$	23.55	4.28	15.89	6.39
[Kr]$5s^2 4d^{10} 4f^2$	17.32	3.97	9.22	16.22
[Kr]$5s^1 4d^{10} 4f^3$	14.49	6.11	6.35	35.58
[Kr]$4d^{10} 4f^4$	12.86	6.84	4.88	57.68
[Kr]$4d^9 4f^5$	11.99	6.58	3.76	105.39
[Kr]$4d^8 4f^6$	11.54	6.34	2.91	156.71
[Ar]$4s^2 3d^{10} 4p^5 4d^{10} 4f^5$	12.01	6.58	3.75	166.89

Sb The energies of the low lying configurations of hard-wall confined Sb are shown in fig. S214 for the neutral atom and in fig. S215 for the cation. The ground state of the unconfined Sb is [Kr]$5s^2 4d^{10} 5p^3$. At $r_c = 2.1a_0$ the ground state changes to [Kr]$5s^2 4d^{10} 4f^3$. Furthermore, at $r_c = 1.9a_0$ we see a ground state crossing to [Kr]$4d^{10} 4f^5$.

At $r_c = 2.1a_0$ the state [Kr]$5s^1 4d^{10} 4f^4$ flips below the initial ground state. At $r_c = 2.1a_0$ the state [Kr]$5s^2 4d^{10} 5p^2 4f^1$ flips below the initial ground state. At $r_c = 2.1a_0$ the state [Kr]$5s^2 4d^{10} 5p^1 4f^2$ flips below the initial ground state. At $r_c = 1.9a_0$ the state [Kr]$4d^9 4f^6$ flips below the initial ground state. At $r_c = 1.8a_0$ the state [Kr]$4d^8 4f^7$ flips below the initial ground state. The ionization energy of the unconfined atom is $\Delta E_0 = 9.02$ eV. The studied configurations, atomic radii and excitation energies in the unconfined calculations are as follows:

configuration	r_ϵ	r_ρ	$r_{\max}$	ΔE
[Kr]$5s^2 4d^{10} 5p^3$	5.68	4.77	2.38	0.00
[Kr]$5s^2 4d^{10} 5p^2 6s^1$	10.32	4.87	5.96	5.69
[Kr]$5s^2 4d^{10} 5p^2 5d^1$	12.53	4.87	4.62	6.83
[Kr]$5s^1 4d^{10} 5p^4$	5.83	4.87	2.35	7.06
[Kr]$5s^2 4d^{10} 5p^2 4f^1$	23.52	4.34	15.86	8.04
[Kr]$5s^2 4d^{10} 5p^1 4f^2$	17.26	4.24	9.08	20.13
[Kr]$5s^2 4d^{10} 4f^3$	14.48	5.85	6.23	34.49
[Kr]$5s^1 4d^{10} 4f^4$	13.02	6.53	4.59	57.98
[Kr]$4d^{10} 4f^5$	11.95	6.58	3.80	83.48
[Kr]$4d^9 4f^6$	11.51	6.34	2.96	138.87
[Kr]$4d^8 4f^7$	11.28	6.11	2.19	196.38

Te The energies of the low lying configurations of hard-wall confined Te are shown in fig. S216 for the neutral atom and in fig. S217 for the cation. The ground state of the unconfined Te is [Kr]$5s^2 4d^{10} 5p^4$. At $r_c = 2.1a_0$ the ground state changes to [Kr]$5s^2 4d^{10} 4f^4$. Furthermore, at $r_c = 1.9a_0$ we see a ground state crossing to [Kr]$5s^1 4d^{10} 4f^5$. At $r_c = 1.8a_0$ we see a ground state crossing to [Kr]$4d^{10} 4f^6$.

At $r_c = 2.1a_0$ the state [Kr]$5s^2 4d^{10} 5p^1 4f^3$ flips below the initial ground state. At $r_c = 2.1a_0$ the state [Kr]$5s^2 4d^{10} 5p^3 4f^1$ flips below the initial ground state. At $r_c = 2.1a_0$ the state [Kr]$5s^2 4d^{10} 5p^2 4f^2$ flips below the initial ground state. At $r_c = 1.9a_0$ the state [Kr]$4d^9 4f^7$ flips below the initial ground state. At $r_c = 1.8a_0$ the state [Kr]$4d^8 4f^8$ flips below the initial ground state. At $r_c = 1.8a_0$ the state [Ar]$4s^2 3d^{10} 4p^5 4d^{10} 4f^7$ flips below the initial ground state. The ionization energy of the unconfined atom is $\Delta E_0 = 8.70$ eV. The studied configurations, atomic radii and excitation energies in the unconfined calculations are as follows:

configuration	r_ϵ	r_ρ	$r_{\max}$	ΔE
[Kr]$5s^2 4d^{10} 5p^4$	5.48	4.77	2.20	0.00
[Kr]$5s^2 4d^{10} 5p^3 6s^1$	9.90	4.87	5.60	5.19
[Kr]$5s^2 4d^{10} 5p^3 5d^1$	11.83	4.77	3.92	6.35
[Kr]$5s^2 4d^{10} 5p^3 4f^1$	23.53	4.28	15.88	7.73
[Kr]$5s^1 4d^{10} 5p^5$	5.54	4.77	2.19	7.97
[Kr]$5s^2 4d^{10} 5p^2 4f^2$	17.26	4.24	8.98	21.94
[Kr]$5s^2 4d^{10} 5p^1 4f^3$	14.67	5.57	5.95	38.53
[Kr]$5s^2 4d^{10} 4f^4$	13.18	6.39	4.41	55.93
[Kr]$5s^1 4d^{10} 4f^5$	12.30	6.39	3.33	81.85
[Kr]$4d^{10} 4f^6$	11.47	6.27	3.01	109.79
[Kr]$4d^9 4f^7$	11.25	6.06	2.27	171.72
[Kr]$4d^8 4f^8$	22.51	5.50	14.71	236.28
[Ar]$4s^2 3d^{10} 4p^5 4d^{10} 4f^7$	11.30	6.06	2.22	240.59

I The energies of the low lying configurations of hard-wall confined I are shown in fig. S218 for the neutral atom and in fig. S219 for the cation. The ground state of the unconfined I is [Kr]$5s^2 4d^{10} 5p^5$. At $r_c = 2.1a_0$ the ground state changes to [Kr]$5s^2 4d^{10} 5p^3 4f^2$. Furthermore, at $r_c = 2.0a_0$ we see a ground state crossing to [Kr]$5s^2 4d^{10} 4f^5$. At $r_c = 1.8a_0$ we see a ground state crossing to [Kr]$4d^{10} 4f^7$.

At $r_c = 2.1a_0$ the state [Kr]$5s^2 4d^{10} 5p^1 4f^4$ flips below the initial ground state. At $r_c = 2.1a_0$ the state [Kr]$5s^2 4d^{10} 5p^4 4f^1$ flips below the initial ground state. At $r_c = 2.0a_0$ the state [Kr]$5s^1 4d^{10} 4f^6$ flips below the initial ground state. At $r_c = 2.0a_0$ the state [Kr]$4d^{10} 5p^1 4f^6$ flips below the initial ground state. At $r_c = 1.9a_0$ the state [Kr]$4d^9 4f^8$ flips below the initial ground state. At $r_c = 1.8a_0$ the state [Kr]$4d^8 4f^9$ flips below the initial ground state. At $r_c = 1.8a_0$ the state [Ar]$4s^2 3d^{10} 4p^5 4d^{10} 4f^8$ flips below the initial ground state. The ionization energy of the unconfined atom is $\Delta E_0 = 10.60$ eV. The studied configurations, atomic radii and excitation energies in the unconfined calculations are as follows:

configuration	r_ϵ	r_ρ	$r_{\max}$	ΔE
[Kr]$5s^2 4d^{10} 5p^5$	5.23	4.67	2.05	0.00
[Kr]$5s^2 4d^{10} 5p^4 6s^1$	9.55	4.96	5.35	6.95
[Kr]$5s^2 4d^{10} 5p^4 5d^1$	11.62	4.77	3.61	8.19
[Kr]$5s^1 4d^{10} 5p^6$	5.25	4.67	2.05	8.90
[Kr]$5s^2 4d^{10} 5p^4 4f^1$	23.58	4.22	15.93	9.63
[Kr]$5s^2 4d^{10} 5p^3 4f^2$	17.32	4.24	8.94	23.64
[Kr]$5s^2 4d^{10} 5p^1 4f^4$	13.75	5.98	3.15	60.94
[Kr]$5s^2 4d^{10} 4f^5$	12.65	6.16	2.89	80.00
[Kr]$5s^1 4d^{10} 4f^6$	11.96	6.16	2.40	107.25
[Kr]$4d^{10} 5p^1 4f^6$	12.03	6.06	2.35	117.48
[Kr]$4d^{10} 4f^7$	11.22	6.06	2.34	137.01
[Kr]$4d^9 4f^8$	22.46	5.50	14.67	206.25
[Kr]$4d^8 4f^9$	16.96	5.42	7.73	277.15
[Ar]$4s^2 3d^{10} 4p^5 4d^{10} 4f^8$	22.44	5.50	14.65	278.68

Xe The energies of the low lying configurations of hard-wall confined Xe are shown in fig. S220 for the neutral atom and in fig. S221 for the cation. The ground state of the unconfined Xe is [Kr]$5s^2 4d^{10} 5p^6$. At $r_c = 2.0a_0$ the ground state changes to [Kr]$5s^2 4d^{10} 4f^6$. Furthermore, at $r_c = 1.8a_0$ we see a ground state crossing to [Kr]$5s^1 4d^{10} 4f^7$. At $r_c = 1.7a_0$ we see a ground state crossing to [Kr]$4d^{10} 4f^8$.

At $r_c = 2.0a_0$ the state [Kr]$5s^2 4d^{10} 5p^1 4f^5$ flips below the initial ground state. At $r_c = 2.0a_0$ the state [Kr]$5s^2 4d^{10} 5p^2 4f^4$ flips below the initial ground state. At $r_c = 2.0a_0$ the state [Kr]$5s^2 4d^{10} 5p^5 4f^1$ flips below the initial ground state. At $r_c = 2.0a_0$ the state [Kr]$5s^2 4d^{10} 5p^4 4f^2$ flips below the initial ground state. At $r_c = 1.9a_0$ the state [Kr]$4d^{10} 5p^1 4f^7$ flips below the initial ground state. At $r_c = 1.8a_0$ the state [Kr]$4d^9 4f^9$ flips below the initial ground state. At $r_c = 1.8a_0$ the state [Ar]$4s^2 3d^{10} 4p^5 4d^{10} 4f^9$ flips below the initial ground state. The ionization energy of the unconfined atom is $\Delta E_0 = 12.39$ eV. The studied configurations, atomic radii and excitation energies in the unconfined calculations are as follows:

configuration	r_ϵ	r_ρ	$r_{\max}$	ΔE
[Kr]$5s^2 4d^{10} 5p^6$	4.98	4.57	1.93	0.00
[Kr]$5s^2 4d^{10} 5p^5 6s^1$	9.30	4.87	5.18	8.64
[Kr]$5s^2 4d^{10} 5p^5 5d^1$	11.70	4.67	3.37	9.97
[Kr]$5s^2 4d^{10} 5p^5 4f^1$	23.62	4.16	15.97	11.42
[Kr]$5s^2 4d^{10} 5p^4 4f^2$	17.55	4.24	9.06	27.72
[Kr]$5s^2 4d^{10} 5p^2 4f^4$	14.63	5.49	1.87	64.65
[Kr]$5s^2 4d^{10} 5p^1 4f^5$	13.49	5.80	1.86	84.63
[Kr]$5s^2 4d^{10} 4f^6$	12.48	5.98	1.73	104.64
[Kr]$5s^1 4d^{10} 4f^7$	11.83	5.91	1.70	132.72
[Kr]$4d^{10} 5p^1 4f^7$	11.90	5.85	1.86	143.56
[Kr]$4d^{10} 4f^8$	22.38	5.50	14.59	165.44
[Kr]$4d^9 4f^9$	17.22	5.46	1.34	241.05
[Ar]$4s^2 3d^{10} 4p^5 4d^{10} 4f^9$	17.24	5.42	1.27	316.97

4.3 r^{2}SCAN

H The energies of the low lying configurations of hard-wall confined H are shown in fig. S115 for the neutral atom. The ground state of the unconfined H is $1s^1$. We do not observe any ground state crossing for H in the considered confinement radii.

The ionization energy of the unconfined atom is $\Delta E_0 = 13.61$ eV. The studied configurations, atomic radii and excitation energies in the unconfined calculations are as follows:

configuration	r_ϵ	r_ρ	$r_{\max}$	ΔE
$1s^1$	2.94	2.97	0.99	0.00
$2p^1$	8.75	4.01	4.26	10.37
$3d^1$	16.93	undefined	9.62	12.13

He The energies of the low lying configurations of hard-wall confined He are shown in fig. S116 for the neutral atom and in fig. S117 for the cation. The ground state of the unconfined He is $1s^2$. We do not observe any ground state crossing for He in the considered confinement radii.

The ionization energy of the unconfined atom is $\Delta E_0 = 24.62$ eV. The studied configurations, atomic radii and excitation energies in the unconfined calculations are as follows:

configuration	r_ϵ	r_ρ	$r_{\max}$	ΔE
$1s^2$	2.22	2.55	0.56	0.00
$1s^1 2p^1$	8.29	4.01	3.87	21.16
$1s^1 3d^1$	16.93	2.04	9.57	23.14
$2p^2$	6.12	4.57	2.33	59.42

Li The energies of the low lying configurations of hard-wall confined Li are shown in fig. S118 for the neutral atom and in fig. S119 for the cation. The ground state of the unconfined Li is [He]$2s^1$. At $r_c = 2.9a_0$ the ground state changes to [He]$2p^1$.

The ionization energy of the unconfined atom is $\Delta E_0 = 5.39$ eV. The studied configurations, atomic radii and excitation energies in the unconfined calculations are as follows:

configuration	r_ϵ	r_ρ	$r_{\max}$	ΔE
[He]$2s^1$	6.31	4.26	3.08	0.00
[He]$2p^1$	8.45	4.01	4.01	2.03
[He]$3d^1$	16.94	1.91	9.62	3.92
$1s^1 2p^2$	5.94	4.46	2.19	60.47

Be The energies of the low lying configurations of hard-wall confined Be are shown in fig. S120 for the neutral atom and in fig. S121 for the cation. The ground state of the unconfined Be is [He]$2s^2$. At $r_c = 2.6a_0$ the ground state changes to [He]$2s^1 2p^1$. Furthermore, at $r_c = 2.2a_0$ we see a ground state crossing to [He]$2p^2$.

At $r_c = 1.2a_0$ the state [He]$2p^1 3d^1$ flips below the initial ground state. The ionization energy of the unconfined atom is $\Delta E_0 = 8.79$ eV. The studied configurations, atomic radii and excitation energies in the unconfined calculations are as follows:

configuration	r_ϵ	r_ρ	$r_{\max}$	ΔE
[He]$2s^2$	5.04	4.16	2.05	0.00
[He]$2s^1 2p^1$	5.50	4.31	2.21	2.38
[He]$2p^2$	5.96	4.46	2.20	6.53
[He]$2p^1 3d^1$	15.51	3.89	8.11	11.61

B The energies of the low lying configurations of hard-wall confined B are shown in fig. S122 for the neutral atom and in fig. S123 for the cation. The ground state of the unconfined B is [He]$2s^2 2p^1$. At $r_c = 2.3a_0$ the ground state changes to [He]$2s^1 2p^2$. Furthermore, at $r_c = 1.8a_0$ we see a ground state crossing to [He]$2p^3$.

The ionization energy of the unconfined atom is $\Delta E_0 = 8.31$ eV. The studied configurations, atomic radii and excitation energies in the unconfined calculations are as follows:

configuration	r_ϵ	r_ρ	$r_{\max}$	ΔE
[He]$2s^22p^1$	4.53	3.99	1.59	0.00
[He]$2s^12p^2$	4.58	3.96	1.54	2.78
[He]$2s^23s^1$	9.80	3.71	5.60	5.02
[He]$2p^3$	4.78	4.12	1.53	9.36

C The energies of the low lying configurations of hard-wall confined C are shown in fig. S124 for the neutral atom and in fig. S125 for the cation. The ground state of the unconfined C is [He]$2s^22p^2$. At $r_c = 2.0a_0$ the ground state changes to [He]$2s^12p^3$. Furthermore, at $r_c = 1.2a_0$ we see a ground state crossing to [He]$2p^4$.

The ionization energy of the unconfined atom is $\Delta E_0 = 11.87$ eV. The studied configurations, atomic radii and excitation energies in the unconfined calculations are as follows:

configuration	r_ϵ	r_ρ	$r_{\max}$	ΔE
[He]$2s^22p^2$	3.93	3.72	1.24	0.00
[He]$2s^12p^3$	3.93	3.72	1.20	3.12
[He]$2s^22p^13s^1$	8.79	4.28	4.86	8.13
[He]$2p^4$	4.44	4.01	1.29	18.19

N The energies of the low lying configurations of hard-wall confined N are shown in fig. S126 for the neutral atom and in fig. S127 for the cation. The ground state of the unconfined N is [He]$2s^22p^3$. At $r_c = 1.2a_0$ the ground state changes to [He]$2s^12p^4$. Furthermore, at $r_c = 1.1a_0$ we see a ground state crossing to [He]$2p^5$.

The ionization energy of the unconfined atom is $\Delta E_0 = 15.48$ eV. The studied configurations, atomic radii and excitation energies in the unconfined calculations are as follows:

configuration	r_ϵ	r_ρ	$r_{\max}$	ΔE
[He]$2s^22p^3$	3.47	3.46	1.04	0.00
[He]$2s^22p^23s^1$	8.10	4.47	4.35	11.37
[He]$2s^12p^4$	3.97	3.59	1.06	11.77
[He]$2p^5$	3.83	3.66	1.00	28.55

O The energies of the low lying configurations of hard-wall confined O are shown in fig. S128 for the neutral atom and in fig. S129 for the cation. The ground state of the unconfined O is [He]$2s^22p^4$. At $r_c = 1.1a_0$ the ground state changes to [He]$2s^12p^5$. Furthermore, at $r_c = 1.0a_0$ we see a ground state crossing to [He]$2p^6$.

The ionization energy of the unconfined atom is $\Delta E_0 = 12.89$ eV. The studied configurations, atomic radii and excitation energies in the unconfined calculations are as follows:

configuration	r_ϵ	r_ρ	$r_{\max}$	ΔE
[He]$2s^22p^4$	3.28	3.25	0.88	0.00
[He]$2s^22p^33s^1$	7.59	4.47	3.98	8.47
[He]$2s^22p^33d^1$	16.69	2.86	9.34	11.39
[He]$2s^12p^5$	3.43	3.35	0.86	15.69
[He]$2p^6$	3.42	3.45	0.83	33.96

F The energies of the low lying configurations of hard-wall confined F are shown in fig. S130 for the neutral atom and in fig. S131 for the cation. The ground state of the unconfined F is [He]$2s^22p^5$. At $r_c = 1.0a_0$ the ground state changes to [He]$2s^12p^6$.

The ionization energy of the unconfined atom is $\Delta E_0 = 17.76$ eV. The studied configurations, atomic radii and excitation energies in the unconfined calculations are as follows:

configuration	r_ϵ	r_ρ	$r_{\max}$	ΔE
[He]$2s^22p^5$	3.00	3.12	0.77	0.00
[He]$2s^22p^43s^1$	7.18	4.38	3.71	13.05
[He]$2s^22p^43d^1$	16.75	2.81	9.37	16.27
[He]$2s^12p^6$	3.08	3.16	0.76	19.78
[He]$2s^12p^53d^1$	16.73	2.86	9.36	36.47

Ne The energies of the low lying configurations of hard-wall confined Ne are shown in fig. S132 for the neutral atom and in fig. S133 for the cation. The ground state of the unconfined Ne is [He]$2s^22p^6$. We do not observe any ground state crossing for Ne in the considered confinement radii.

The ionization energy of the unconfined atom is $\Delta E_0 = 22.50$ eV. The studied configurations, atomic radii and excitation energies in the unconfined calculations are as follows:

configuration	r_ϵ	r_ρ	$r_{\max}$	ΔE
[He]$2s^22p^6$	2.77	3.03	0.68	0.00
[He]$2s^22p^53s^1$	6.88	4.35	3.50	17.55
[He]$2s^22p^53d^1$	16.79	2.66	9.41	21.01
[He]$2s^12p^63d^1$	16.78	2.70	9.42	45.57

Na The energies of the low lying configurations of hard-wall confined Na are shown in

fig. S134 for the neutral atom and in fig. S135 for the cation. The ground state of the unconfined Na is [Ne]$3s^1$. At $r_c = 2.0a_0$ the ground state changes to [Ne]$3d^1$.

At $r_c = 1.5a_0$ the state [Ne]$3p^1$ flips below the initial ground state. At $r_c = 1.1a_0$ the state [Ne]$4f^1$ flips below the initial ground state. The ionization energy of the unconfined atom is $\Delta E_0 = 5.16$ eV. The studied configurations, atomic radii and excitation energies in the unconfined calculations are as follows:

configuration	r_ϵ	r_ρ	$r_{\max}$	ΔE
[Ne]$3s^1$	6.64	4.28	3.33	0.00
[Ne]$3p^1$	9.59	3.14	4.96	2.18
[Ne]$3d^1$	16.82	2.54	9.50	3.67
[Ne]$4f^1$	25.92	2.53	16.75	4.28

Mg The energies of the low lying configurations of hard-wall confined Mg are shown in fig. S136 for the neutral atom and in fig. S137 for the cation. The ground state of the unconfined Mg is [Ne]$3s^2$. At $r_c = 1.8a_0$ the ground state changes to [Ne]$3d^2$.

At $r_c = 1.8a_0$ the state [Ne]$3s^1 3d^1$ flips below the initial ground state. At $r_c = 1.7a_0$ the state [Ne]$3p^1 3d^1$ flips below the initial ground state. At $r_c = 1.5a_0$ the state [Ne]$3s^1 3p^1$ flips below the initial ground state. At $r_c = 1.5a_0$ the state [Ne]$3p^2$ flips below the initial ground state. At $r_c = 1.3a_0$ the state [Ne]$3d^1 4f^1$ flips below the initial ground state. The ionization energy of the unconfined atom is $\Delta E_0 = 7.37$ eV. The studied configurations, atomic radii and excitation energies in the unconfined calculations are as follows:

configuration	r_ϵ	r_ρ	$r_{\max}$	ΔE
[Ne]$3s^2$	5.88	4.64	2.58	0.00
[Ne]$3s^1 3p^1$	6.86	4.74	3.24	2.52
[Ne]$3s^1 3d^1$	15.14	4.13	7.68	5.69
[Ne]$3p^2$	7.31	5.09	3.23	6.43
[Ne]$3p^1 3d^1$	13.23	4.80	6.04	10.00
[Ne]$3d^2$	11.87	5.22	5.23	13.77
[Ne]$3d^1 4f^1$	22.53	3.88	12.79	15.41

Al The energies of the low lying configurations of hard-wall confined Al are shown in fig. S138 for the neutral atom and in fig. S139 for the cation. The ground state of the unconfined Al is [Ne]$3s^2 3p^1$. At $r_c = 1.8a_0$ the ground state changes to [Ne]$3s^2 3d^1$. Furthermore, at $r_c = 1.7a_0$ we see a ground state crossing to [Ne]$3s^1 3d^2$. At $r_c = 1.6a_0$ we see a ground state crossing to [Ne]$3d^3$.

At $r_c = 1.7a_0$ the state [Ne]$3p^1 3d^2$ flips below the initial ground state. At $r_c = 1.5a_0$ the state [Ne]$3s^1 3p^2$ flips below the initial ground state. The ionization energy of the unconfined atom is $\Delta E_0 = 5.91$ eV. The studied configurations, atomic radii and excitation energies in the unconfined calculations are as follows:

configuration	r_ϵ	r_ρ	$r_{\max}$	ΔE
[Ne]$3s^2 3p^1$	5.92	4.61	2.65	0.00
[Ne]$3s^1 3p^2$	5.96	4.74	2.52	3.02
[Ne]$3s^2 4s^1$	11.22	4.07	6.72	3.07
[Ne]$3s^2 3d^1$	15.15	4.23	7.64	4.25
[Ne]$3s^1 3d^2$	10.97	5.14	4.17	14.49
[Ne]$3p^1 3d^2$	10.26	5.47	3.81	20.63
[Ne]$3d^3$	9.49	5.75	3.59	27.05

Si The energies of the low lying configurations of hard-wall confined Si are shown in fig. S140 for the neutral atom and in fig. S141 for the cation. The ground state of the unconfined Si is [Ne]$3s^2 3p^2$. At $r_c = 1.7a_0$ the ground state changes to [Ne]$3s^2 3d^2$. Furthermore, at $r_c = 1.6a_0$ we see a ground state crossing to [Ne]$3s^1 3d^3$. At $r_c = 1.5a_0$ we see a ground state crossing to [Ne]$3d^4$.

At $r_c = 1.7a_0$ the state [Ne]$3s^2 3p^1 3d^1$ flips below the initial ground state. At $r_c = 1.6a_0$ the state [Ne]$3p^1 3d^3$ flips below the initial ground state. At $r_c = 1.5a_0$ the state [Ne]$3s^1 3p^3$ flips below the initial ground state. The ionization energy of the unconfined atom is $\Delta E_0 = 8.42$ eV. The studied configurations, atomic radii and excitation energies in the unconfined calculations are as follows:

configuration	r_ϵ	r_ρ	$r_{\max}$	ΔE
[Ne]$3s^2 3p^2$	5.30	4.50	2.16	0.00
[Ne]$3s^1 3p^3$	5.26	4.50	2.08	3.36
[Ne]$3s^2 3p^1 4s^1$	10.17	4.39	5.92	5.24
[Ne]$3s^2 3p^1 3d^1$	14.05	4.28	6.08	6.57
[Ne]$3s^2 3d^2$	10.73	5.03	3.75	14.43
[Ne]$3s^1 3d^3$	8.91	5.39	2.93	28.61
[Ne]$3p^1 3d^3$	8.56	5.34	2.85	36.58
[Ne]$3d^4$	8.09	5.50	2.73	45.83

P The energies of the low lying configurations of hard-wall confined P are shown in fig. S142 for the neutral atom and in fig. S143 for the cation. The ground state of the unconfined P is [Ne]$3s^2 3p^3$. At $r_c = 1.5a_0$ the ground state changes to [Ne]$3s^1 3d^4$. Furthermore, at $r_c = 1.4a_0$ we see a ground state crossing to [Ne]$3d^5$.

At $r_c = 1.5a_0$ the state [Ne]$3p^1 3d^4$ flips below the initial ground state. At $r_c = 1.5a_0$ the state [Ne]$3s^2 3p^2 3d^1$ flips below the initial ground state. At $r_c = 1.5a_0$ the state [Ne]$3s^2 3p^1 3d^2$ flips below the initial ground state. At $r_c = 1.1a_0$ the state [Ne]$3s^1 3p^4$ flips below the initial ground state. The ionization energy of the unconfined atom is $\Delta E_0 = 10.92$ eV. The studied configurations, atomic radii and excitation energies in the unconfined calculations are as follows:

configuration	r_ϵ	r_ρ	$r_{\max}$	ΔE
[Ne]$3s^2 3p^3$	4.77	4.26	1.83	0.00
[Ne]$3s^2 3p^2 4s^1$	9.45	4.55	5.35	7.45
[Ne]$3s^1 3p^4$	5.10	4.38	1.91	8.39
[Ne]$3s^2 3p^2 3d^1$	13.78	4.17	5.08	8.97
[Ne]$3s^2 3p^1 3d^2$	10.09	5.00	2.87	19.58
[Ne]$3s^1 3d^4$	7.68	5.17	2.30	48.54
[Ne]$3p^1 3d^4$	7.48	5.13	2.27	58.37
[Ne]$3d^5$	7.16	5.17	2.20	70.58

S The energies of the low lying configurations of hard-wall confined S are shown in fig. S144 for the neutral atom and in fig. S145 for the cation. The ground state of the unconfined S is [Ne]$3s^2 3p^4$. At $r_c = 1.5a_0$ the ground state changes to [Ne]$3s^2 3p^3 3d^1$. Furthermore, at $r_c = 1.4a_0$ we see a ground state crossing to [Ne]$3s^1 3d^5$. At $r_c = 1.2a_0$ we see a ground state crossing to [Ne]$3d^6$.

At $r_c = 1.5a_0$ the state [Ne]$3s^2 3p^2 3d^2$ flips below the initial ground state. At $r_c = 1.4a_0$ the state [Ne]$3p^1 3d^5$ flips below the initial ground state. At $r_c = 1.4a_0$ the state [Ne]$3s^2 3d^4$ flips below the initial ground state. At $r_c = 1.4a_0$ the state [Ne]$3s^1 3p^1 3d^4$ flips below the initial ground state. At $r_c = 1.1a_0$ the state [Ne]$3s^1 3p^5$ flips below the initial ground state. The ionization energy of the unconfined atom is $\Delta E_0 = 9.95$ eV. The studied configurations, atomic radii and excitation energies in the unconfined calculations are as follows:

configuration	r_ϵ	r_ρ	$r_{\max}$	ΔE
[Ne]$3s^2 3p^4$	4.50	4.13	1.62	0.00
[Ne]$3s^2 3p^3 4s^1$	8.91	4.54	4.93	6.24
[Ne]$3s^2 3p^3 3d^1$	13.88	4.07	2.68	7.97
[Ne]$3s^1 3p^5$	4.61	4.20	1.64	10.37
[Ne]$3s^2 3p^2 3d^2$	9.76	4.71	2.32	21.42
[Ne]$3s^2 3d^4$	7.55	5.06	2.08	49.88
[Ne]$3s^1 3p^1 3d^4$	7.24	5.00	1.96	56.03
[Ne]$3s^1 3d^5$	6.85	4.97	1.90	71.26
[Ne]$3p^1 3d^5$	6.71	5.00	1.88	83.00
[Ne]$3d^6$	9.37	5.00	2.14	102.09

Cl The energies of the low lying configurations of hard-wall confined Cl are shown in fig. S146 for the neutral atom and in fig. S147 for the cation. The ground state of the unconfined Cl is [Ne]$3s^2 3p^5$. At $r_c = 1.4a_0$ the ground state changes to [Ne]$3s^2 3p^3 3d^2$. Furthermore, at $r_c = 1.3a_0$ we see a ground state crossing to [Ne]$3s^2 3p^2 3d^3$. At $r_c = 1.2a_0$ we see a ground state crossing to [Ne]$3s^1 3d^6$. Moreover, at $r_c = 1.1a_0$ we see a ground state crossing to [Ne]$3d^7$.

At $r_c = 1.4a_0$ the state [Ne]$3s^2 3p^4 3d^1$ flips below the initial ground state. At $r_c = 1.3a_0$ the state [Ne]$3p^1 3d^6$ flips below the initial ground state. At $r_c = 1.0a_0$ the state [Ne]$3s^1 3p^6$ flips below the initial ground state. The ionization energy of the unconfined atom is $\Delta E_0 = 13.12$ eV. The studied configurations, atomic radii and excitation energies in the unconfined calculations are as follows:

configuration	r_ϵ	r_ρ	$r_{\max}$	ΔE
[Ne]$3s^2 3p^5$	4.17	3.93	1.44	0.00
[Ne]$3s^2 3p^4 4s^1$	8.46	4.63	4.62	9.18
[Ne]$3s^2 3p^4 3d^1$	14.44	3.87	6.09	11.22
[Ne]$3s^1 3p^6$	4.21	3.99	1.44	12.39
[Ne]$3s^2 3p^3 3d^2$	9.65	4.57	1.97	23.25
[Ne]$3s^2 3p^2 3d^3$	8.01	4.81	1.85	40.35
[Ne]$3s^1 3d^6$	13.00	4.72	2.66	104.83
[Ne]$3p^1 3d^6$	12.91	4.67	2.35	118.63
[Ne]$3d^7$	7.21	4.89	1.69	141.11

Ar The energies of the low lying configurations of hard-wall confined Ar are shown in fig. S148 for the neutral atom and in fig. S149 for the cation. The ground state of the uncon-

fined Ar is [Ne]$3s^2 3p^6$. At $r_c = 1.3a_0$ the ground state changes to [Ne]$3s^2 3p^4 3d^2$. Furthermore, at $r_c = 1.2a_0$ we see a ground state crossing to [Ne]$3s^1 3p^2 3d^5$. At $r_c = 1.1a_0$ we see a ground state crossing to [Ne]$3d^8$.

At $r_c = 1.3a_0$ the state [Ne]$3s^2 3p^1 3d^5$ flips below the initial ground state. At $r_c = 1.2a_0$ the state [Ne]$3p^1 3d^7$ flips below the initial ground state. At $r_c = 1.2a_0$ the state [Ne]$3s^1 3d^7$ flips below the initial ground state. At $r_c = 1.2a_0$ the state [Ne]$3s^2 3d^6$ flips below the initial ground state. At $r_c = 1.2a_0$ the state [Ne]$3s^2 3p^5 3d^1$ flips below the initial ground state. At $r_c = 1.1a_0$ the state [Ne]$3s^1 3p^6 3d^1$ flips below the initial ground state. The ionization energy of the unconfined atom is $\Delta E_0 = 16.17$ eV. The studied configurations, atomic radii and excitation energies in the unconfined calculations are as follows:

configuration	r_ϵ	r_ρ	$r_{\max}$	ΔE
[Ne]$3s^2 3p^6$	3.87	3.76	1.29	0.00
[Ne]$3s^2 3p^5 4s^1$	8.14	4.54	4.40	12.07
[Ne]$3s^2 3p^5 3d^1$	14.97	3.71	7.17	14.38
[Ne]$3s^1 3p^6 3d^1$	14.84	3.70	6.88	29.27
[Ne]$3s^2 3p^4 3d^2$	9.93	4.50	1.76	30.67
[Ne]$3s^2 3p^1 3d^5$	6.46	4.72	1.51	87.07
[Ne]$3s^1 3p^2 3d^5$	6.27	4.61	1.47	93.19
[Ne]$3s^2 3d^6$	10.64	4.72	1.55	112.17
[Ne]$3s^1 3d^7$	8.62	4.79	1.65	146.96
[Ne]$3p^1 3d^7$	8.47	4.79	1.60	162.77
[Ne]$3d^8$	6.29	4.73	1.44	187.93

K The energies of the low lying configurations of hard-wall confined K are shown in fig. S150 for the neutral atom and in fig. S151 for the cation. The ground state of the unconfined K is [Ar]$4s^1$. At $r_c = 4.5a_0$ the ground state changes to [Ar]$3d^1$. Furthermore, at $r_c = 1.2a_0$ we see a ground state crossing to [Ne]$3s^1 3p^3 3d^5$. At $r_c = 1.0a_0$ we see a ground state crossing to [Ne]$3d^9$.

At $r_c = 2.9a_0$ the state [Ne]$3s^2 3p^5 3d^2$ flips below the initial ground state. At $r_c = 2.5a_0$ the state [Ne]$3s^1 3p^6 3d^2$ flips below the initial ground state. At $r_c = 2.4a_0$ the state [Ne]$3s^2 3p^4 3d^3$ flips below the initial ground state. At $r_c = 2.2a_0$ the state [Ne]$3s^2 3p^3 3d^4$ flips below the initial ground state. At $r_c = 1.9a_0$ the state [Ne]$3s^2 3p^2 3d^5$ flips below the initial ground state. At $r_c = 1.5a_0$ the state [Ne]$3s^1 3d^8$ flips below the initial ground state. At $r_c = 1.5a_0$ the state [Ne]$3p^1 3d^8$ flips below the initial ground state. At $r_c = 1.4a_0$ the state [Ar]$4p^1$ flips below the initial ground state. The ionization energy of the unconfined atom is $\Delta E_0 = 4.25$ eV. The studied configurations, atomic radii and excitation energies in the unconfined calculations are as follows:

configuration	r_ϵ	r_ρ	$r_{\max}$	ΔE
[Ar]$4s^1$	7.89	4.49	4.22	0.00
[Ar]$4p^1$	10.76	3.55	5.90	1.58
[Ar]$3d^1$	15.43	3.49	7.86	2.56
[Ne]$3s^2 3p^5 3d^2$	10.31	4.38	1.58	23.17
[Ne]$3s^1 3p^6 3d^2$	10.19	4.32	1.53	40.31
[Ne]$3s^2 3p^4 3d^3$	8.00	4.55	1.45	42.47
[Ne]$3s^2 3p^3 3d^4$	6.83	4.55	1.38	59.48
[Ne]$3s^2 3p^2 3d^5$	6.27	4.55	1.34	83.89
[Ne]$3s^1 3p^3 3d^5$	6.10	4.45	1.32	89.81
[Ne]$3s^1 3d^8$	6.92	4.67	1.37	182.47
[Ne]$3p^1 3d^8$	6.80	4.67	1.34	200.28
[Ne]$3d^9$	5.76	4.67	1.25	227.96

Ca The energies of the low lying configurations of hard-wall confined Ca are shown in fig. S152 for the neutral atom and in fig. S153 for the cation. The ground state of the unconfined Ca is [Ar]$4s^2$. At $r_c = 5.0a_0$ the ground state changes to [Ar]$4s^1 3d^1$. Furthermore, at $r_c = 4.4a_0$ we see a ground state crossing to [Ar]$3d^2$. At $r_c = 1.1a_0$ we see a ground state crossing to [Ne]$3s^2 3p^3 3d^5$. Moreover, at $r_c = 1.0a_0$ we see a ground state crossing to [Ne]$3d^{10}$.

At $r_c = 3.5a_0$ the state [Ar]$3d^1 4p^1$ flips below the initial ground state. At $r_c = 3.2a_0$ the state [Ne]$3s^2 3p^5 3d^3$ flips below the initial ground state. At $r_c = 2.9a_0$ the state [Ne]$3s^1 3p^6 3d^3$ flips below the initial ground state. At $r_c = 2.8a_0$ the state [Ne]$3s^2 3p^4 3d^4$ flips below the initial ground state. At $r_c = 1.8a_0$ the state [Ne]$3s^1 3d^9$ flips below the initial ground state. At $r_c = 1.7a_0$ the state [Ne]$3p^1 3d^9$ flips below the initial ground state. At $r_c = 1.5a_0$ the state [Ar]$4s^1 4p^1$ flips below the initial ground state. The ionization energy of the unconfined atom is $\Delta E_0 = 5.81$ eV. The studied configurations,

atomic radii and excitation energies in the unconfined calculations are as follows:

configuration	r_ϵ	r_ρ	$r_{\max}$	ΔE
[Ar]$4s^2$	7.22	5.25	3.46	0.00
[Ar]$4s^1 4p^1$	8.10	5.36	4.20	1.61
[Ar]$4s^1 3d^1$	7.84	4.98	3.55	1.74
[Ar]$3d^1 4p^1$	9.24	4.89	4.73	3.99
[Ar]$3d^2$	10.68	4.32	1.43	4.54
[Ne]$3s^2 3p^5 3d^3$	8.25	4.55	1.31	28.56
[Ne]$3s^1 3p^6 3d^3$	8.14	4.50	1.29	47.90
[Ne]$3s^2 3p^4 3d^4$	6.90	4.50	1.25	50.57
[Ne]$3s^2 3p^3 3d^5$	6.14	4.41	1.21	69.90
[Ne]$3s^1 3d^9$	6.01	4.54	1.18	215.37
[Ne]$3p^1 3d^9$	5.92	4.54	1.17	235.21
[Ne]$3d^{10}$	5.40	4.46	1.12	265.36

Sc The energies of the low lying configurations of hard-wall confined Sc are shown in fig. S154 for the neutral atom and in fig. S155 for the cation. The ground state of the unconfined Sc is [Ar]$4s^2 3d^1$. At $r_c = 5.9a_0$ the ground state changes to [Ar]$4s^1 3d^2$. Furthermore, at $r_c = 4.3a_0$ we see a ground state crossing to [Ar]$3d^3$. At $r_c = 1.1a_0$ we see a ground state crossing to [Ne]$3s^2 3p^4 3d^5$. Moreover, at $r_c = 1.0a_0$ we see a ground state crossing to [Ne]$3s^2 3d^9$.

At $r_c = 3.5a_0$ the state [Ar]$3d^2 4p^1$ flips below the initial ground state. At $r_c = 3.1a_0$ the state [Ne]$3s^2 3p^5 3d^4$ flips below the initial ground state. At $r_c = 2.7a_0$ the state [Ne]$3s^1 3p^6 3d^4$ flips below the initial ground state. At $r_c = 1.9a_0$ the state [Ne]$3s^2 3p^1 3d^8$ flips below the initial ground state. At $r_c = 1.7a_0$ the state [Ne]$3s^1 3d^{10}$ flips below the initial ground state. At $r_c = 1.4a_0$ the state [Ar]$4s^1 3d^1 4p^1$ flips below the initial ground state. The ionization energy of the unconfined atom is $\Delta E_0 = 6.15$ eV. The studied configurations, atomic radii and excitation energies in the unconfined calculations are as follows:

configuration	r_ϵ	r_ρ	$r_{\max}$	ΔE
[Ar]$4s^2 3d^1$	6.77	5.05	3.25	0.00
[Ar]$4s^1 3d^2$	6.81	4.87	3.24	0.52
[Ar]$4s^1 3d^1 4p^1$	7.56	5.19	3.82	1.65
[Ar]$3d^3$	8.55	4.57	1.20	3.03
[Ar]$3d^2 4p^1$	8.58	4.96	4.33	3.15
[Ne]$3s^2 3p^5 3d^4$	7.06	4.43	1.14	30.06
[Ne]$3s^1 3p^6 3d^4$	6.96	4.43	1.13	51.60
[Ne]$3s^2 3p^4 3d^5$	6.17	4.39	1.11	54.69
[Ne]$3s^2 3p^1 3d^8$	6.75	4.57	1.10	157.67
[Ne]$3s^2 3d^9$	5.59	4.46	1.05	192.43
[Ne]$3s^1 3d^{10}$	5.45	4.46	1.05	245.40

Ti The energies of the low lying configurations of hard-wall confined Ti are shown in fig. S156 for the neutral atom and in fig. S157 for the cation. The ground state of the unconfined Ti is [Ar]$4s^1 3d^3$. At $r_c = 4.3a_0$ the ground state changes to [Ar]$3d^4$. Furthermore, at $r_c = 1.0a_0$ we see a ground state crossing to [Ne]$3s^2 3p^5 3d^5$.

At $r_c = 2.1a_0$ the state [Ne]$3s^1 3p^6 3d^5$ flips below the initial ground state. At $r_c = 2.0a_0$ the state [Ne]$3s^2 3p^4 3d^6$ flips below the initial ground state. At $r_c = 1.7a_0$ the state [Ne]$3s^2 3p^3 3d^7$ flips below the initial ground state. At $r_c = 1.2a_0$ the state [Ar]$3d^3 4p^1$ flips below the initial ground state. The ionization energy of the unconfined atom is $\Delta E_0 = 6.60$ eV. The studied configurations, atomic radii and excitation energies in the unconfined calculations are as follows:

configuration	r_ϵ	r_ρ	$r_{\max}$	ΔE
[Ar]$4s^1 3d^3$	6.27	4.73	3.03	0.00
[Ar]$4s^2 3d^2$	6.47	4.93	3.10	0.60
[Ar]$3d^4$	7.27	4.47	1.05	2.11
[Ar]$3d^3 4p^1$	8.27	4.79	4.09	2.93
[Ne]$3s^2 3p^5 3d^5$	6.27	4.32	1.02	32.04
[Ne]$3s^1 3p^6 3d^5$	6.19	4.32	1.01	55.82
[Ne]$3s^2 3p^4 3d^6$	15.18	3.94	7.38	63.86
[Ne]$3s^2 3p^3 3d^7$	10.67	4.45	1.09	96.63

V The energies of the low lying configurations of hard-wall confined V are shown in fig. S158 for the neutral atom and in fig. S159 for the cation. The ground state of the unconfined V is [Ar]$4s^1 3d^4$. At $r_c = 4.4a_0$ the ground state changes to [Ar]$3d^5$.

At $r_c = 2.2a_0$ the state [Ne]$3s^2 3p^5 3d^6$ flips

below the initial ground state. At $r_c = 2.0a_0$ the state [Ne]$3s^13p^63d^6$ flips below the initial ground state. At $r_c = 1.8a_0$ the state [Ne]$3s^23p^43d^7$ flips below the initial ground state. At $r_c = 1.2a_0$ the state [Ar]$3d^44p^1$ flips below the initial ground state. The ionization energy of the unconfined atom is $\Delta E_0 = 6.93$ eV. The studied configurations, atomic radii and excitation energies in the unconfined calculations are as follows:

configuration	r_ϵ	r_ρ	$r_{\max}$	ΔE
[Ar]$4s^13d^4$	5.91	4.59	2.86	0.00
[Ar]$4s^23d^3$	6.24	4.80	2.98	1.66
[Ar]$3d^5$	6.42	4.32	0.94	1.68
[Ar]$3d^44p^1$	8.09	4.67	3.91	3.18
[Ne]$3s^23p^53d^6$	15.03	4.01	0.97	38.69
[Ne]$3s^13p^63d^6$	14.98	4.01	0.97	64.75
[Ne]$3s^23p^43d^7$	10.50	4.32	0.96	76.35

Cr The energies of the low lying configurations of hard-wall confined Cr are shown in fig. S160 for the neutral atom and in fig. S161 for the cation. The ground state of the unconfined Cr is [Ar]$4s^13d^5$. At $r_c = 3.4a_0$ the ground state changes to [Ar]$3d^6$.

At $r_c = 2.1a_0$ the state [Ne]$3s^23p^53d^7$ flips below the initial ground state. At $r_c = 1.8a_0$ the state [Ne]$3s^13p^63d^7$ flips below the initial ground state. At $r_c = 1.7a_0$ the state [Ne]$3s^23p^43d^8$ flips below the initial ground state. At $r_c = 1.2a_0$ the state [Ar]$3d^54p^1$ flips below the initial ground state. The ionization energy of the unconfined atom is $\Delta E_0 = 7.21$ eV. The studied configurations, atomic radii and excitation energies in the unconfined calculations are as follows:

configuration	r_ϵ	r_ρ	$r_{\max}$	ΔE
[Ar]$4s^13d^5$	5.63	4.43	2.72	0.00
[Ar]$4s^23d^4$	6.06	4.79	2.87	2.67
[Ar]$3d^54p^1$	7.97	4.59	3.78	3.41
[Ne]$3s^23p^53d^7$	10.18	4.20	0.87	47.42
[Ne]$3s^13p^63d^7$	10.10	4.20	0.87	75.81
[Ne]$3s^23p^43d^8$	8.04	4.37	0.87	89.76
[Ar]$3d^6$	Not converged			

Mn The energies of the low lying configurations of hard-wall confined Mn are shown in fig. S162 for the neutral atom and in fig. S163 for the cation. The ground state of the unconfined Mn is [Ar]$4s^23d^5$. At $r_c = 4.4a_0$ the ground state changes to [Ar]$4s^13d^6$. Furthermore, at $r_c = 3.5a_0$ we see a ground state crossing to [Ar]$3d^7$.

At $r_c = 2.9a_0$ the state [Ar]$3d^64p^1$ flips below the initial ground state. At $r_c = 2.4a_0$ the state [Ne]$3s^23p^53d^8$ flips below the initial ground state. At $r_c = 2.1a_0$ the state [Ne]$3s^13p^63d^8$ flips below the initial ground state. At $r_c = 1.3a_0$ the state [Ar]$4s^13d^54p^1$ flips below the initial ground state. The ionization energy of the unconfined atom is $\Delta E_0 = 6.88$ eV. The studied configurations, atomic radii and excitation energies in the unconfined calculations are as follows:

configuration	r_ϵ	r_ρ	$r_{\max}$	ΔE
[Ar]$4s^23d^5$	5.90	4.65	2.79	0.00
[Ar]$4s^13d^54p^1$	6.66	4.79	3.14	1.87
[Ar]$4s^13d^6$	5.79	4.43	2.62	1.92
[Ar]$3d^64p^1$	7.86	4.59	3.66	5.54
[Ar]$3d^7$	9.39	4.20	0.80	5.96
[Ne]$3s^23p^53d^8$	7.47	4.20	0.80	53.19
[Ne]$3s^13p^63d^8$	7.39	4.20	0.80	83.98

Fe The energies of the low lying configurations of hard-wall confined Fe are shown in fig. S164 for the neutral atom and in fig. S165 for the cation. The ground state of the unconfined Fe is [Ar]$4s^23d^6$. At $r_c = 5.0a_0$ the ground state changes to [Ar]$4s^13d^7$. Furthermore, at $r_c = 3.6a_0$ we see a ground state crossing to [Ar]$3d^8$.

At $r_c = 2.8a_0$ the state [Ar]$3d^74p^1$ flips below the initial ground state. At $r_c = 2.3a_0$ the state [Ne]$3s^23p^53d^9$ flips below the initial ground state. At $r_c = 2.1a_0$ the state [Ne]$3s^13p^63d^9$ flips below the initial ground state. At $r_c = 1.2a_0$ the state [Ar]$4s^13d^64p^1$ flips below the initial ground state. The ionization energy of the unconfined atom is $\Delta E_0 = 7.55$ eV. The studied configurations, atomic radii and excitation energies in the unconfined calculations are as follows:

configuration	r_ϵ	r_ρ	$r_{\max}$	ΔE
[Ar]$4s^23d^6$	5.64	4.63	2.58	0.00
[Ar]$4s^13d^7$	5.45	4.37	2.55	0.76
[Ar]$4s^13d^64p^1$	6.51	4.72	3.03	2.45
[Ar]$3d^8$	6.60	4.20	0.74	3.88
[Ar]$3d^74p^1$	7.80	4.54	3.60	4.50
[Ne]$3s^23p^53d^9$	6.01	4.13	0.74	55.99
[Ne]$3s^13p^63d^9$	5.96	4.13	0.74	89.28

Co The energies of the low lying configurations of hard-wall confined Co are shown in fig. S166 for the neutral atom and in fig. S167 for the cation. The ground state of the unconfined Co is [Ar]$4s^13d^8$. At $r_c = 3.8a_0$ the ground state changes to [Ar]$3d^9$.

At $r_c = 1.8a_0$ the state [Ne]$3s^23p^53d^{10}$ flips below the initial ground state. At $r_c = 1.6a_0$ the state [Ne]$3s^13p^63d^{10}$ flips below the initial ground state. At $r_c = 1.0a_0$ the state [Ar]$3d^84p^1$ flips below the initial ground state. The ionization energy of the unconfined atom is $\Delta E_0 = 7.76$ eV. The studied configurations, atomic radii and excitation energies in the unconfined calculations are as follows:

configuration	r_ϵ	r_ρ	$r_{\max}$	ΔE
[Ar]$4s^13d^8$	5.27	4.40	2.49	0.00
[Ar]$4s^23d^7$	5.44	4.52	2.44	0.38
[Ar]$3d^9$	5.58	4.18	0.69	2.20
[Ar]$3d^84p^1$	7.78	4.52	3.56	3.88
[Ne]$3s^23p^53d^{10}$	5.28	4.07	0.69	59.33
[Ne]$3s^13p^63d^{10}$	5.25	4.07	0.69	95.18

Ni The energies of the low lying configurations of hard-wall confined Ni are shown in fig. S168 for the neutral atom and in fig. S169 for the cation. The ground state of the unconfined Ni is [Ar]$4s^13d^9$. At $r_c = 4.2a_0$ the ground state changes to [Ar]$3d^{10}$.

At $r_c = 1.4a_0$ the state [Ar]$3d^94f^1$ flips below the initial ground state. At $r_c = 1.2a_0$ the state [Ne]$3s^23p^53d^{10}4f^1$ flips below the initial ground state. At $r_c = 1.0a_0$ the state [Ar]$3d^94p^1$ flips below the initial ground state. The ionization energy of the unconfined atom is $\Delta E_0 = 7.89$ eV. The studied configurations, atomic radii and excitation energies in the unconfined calculations are as follows:

configuration	r_ϵ	r_ρ	$r_{\max}$	ΔE
[Ar]$4s^13d^9$	5.14	4.29	2.42	0.00
[Ar]$3d^{10}$	5.16	4.07	0.65	1.27
[Ar]$4s^23d^8$	5.29	4.52	2.33	1.50
[Ar]$3d^94p^1$	7.76	4.52	3.53	4.00
[Ar]$3d^94f^1$	25.85	3.35	16.62	7.01
[Ne]$3s^23p^53d^{10}4f^1$	25.86	3.37	16.61	69.84

Cu The energies of the low lying configurations of hard-wall confined Cu are shown in fig. S170 for the neutral atom and in fig. S171 for the cation. The ground state of the unconfined Cu is [Ar]$4s^13d^{10}$. At $r_c = 1.4a_0$ the ground state changes to [Ar]$3d^{10}4f^1$.

At $r_c = 1.1a_0$ the state [Ar]$3d^{10}4d^1$ flips below the initial ground state. At $r_c = 1.0a_0$ the state [Ar]$3d^{10}4p^1$ flips below the initial ground state. The ionization energy of the unconfined atom is $\Delta E_0 = 8.02$ eV. The studied configurations, atomic radii and excitation energies in the unconfined calculations are as follows:

configuration	r_ϵ	r_ρ	$r_{\max}$	ΔE
[Ar]$4s^13d^{10}$	5.04	4.13	2.37	0.00
[Ar]$4s^23d^9$	5.16	4.29	2.23	2.59
[Ar]$3d^{10}4p^1$	7.74	4.38	3.50	4.13
[Ar]$3d^{10}4d^1$	16.62	3.37	9.27	6.51
[Ar]$3d^{10}4f^1$	25.86	3.33	16.56	7.14

Zn The energies of the low lying configurations of hard-wall confined Zn are shown in fig. S172 for the neutral atom and in fig. S173 for the cation. The ground state of the unconfined Zn is [Ar]$4s^23d^{10}$. At $r_c = 1.3a_0$ the ground state changes to [Ar]$3d^{10}4f^2$.

At $r_c = 1.3a_0$ the state [Ar]$4s^13d^{10}4f^1$ flips below the initial ground state. At $r_c = 1.2a_0$ the state [Ar]$3d^{10}4d^14f^1$ flips below the initial ground state. At $r_c = 1.2a_0$ the state [Ar]$3d^{10}4p^14f^1$ flips below the initial ground state. At $r_c = 1.1a_0$ the state [Ar]$4s^13d^{10}4d^1$ flips below the initial ground state. At $r_c = 1.0a_0$ the state [Ar]$4s^13d^{10}4p^1$ flips below the initial ground state. At $r_c = 1.0a_0$ the state [Ar]$3d^{10}4p^2$ flips below the initial ground state. The ionization energy of the unconfined atom is $\Delta E_0 = 9.19$ eV. The studied configurations, atomic radii and excitation energies in the unconfined calculations are as follows:

configuration	r_ϵ	r_ρ	$r_{\max}$	ΔE
[Ar]$4s^2 3d^{10}$	5.04	4.23	2.15	0.00
[Ar]$4s^1 3d^{10} 4p^1$	6.21	4.50	2.78	3.93
[Ar]$4s^1 3d^{10} 4d^1$	16.22	3.76	8.85	7.65
[Ar]$4s^1 3d^{10} 4f^1$	25.91	3.64	16.69	8.31
[Ar]$3d^{10} 4p^2$	6.66	4.82	2.81	9.40
[Ar]$3d^{10} 4p^1 4f^1$	25.55	4.04	16.29	14.60
[Ar]$3d^{10} 4d^1 4f^1$	22.09	3.76	12.32	20.48
[Ar]$3d^{10} 4f^2$	18.87	2.89	9.40	22.50

Ga The energies of the low lying configurations of hard-wall confined Ga are shown in fig. S174 for the neutral atom and in fig. S175 for the cation. The ground state of the unconfined Ga is [Ar]$4s^2 3d^{10} 4p^1$. At $r_c = 1.3a_0$ the ground state changes to [Ar]$4s^1 3d^{10} 4f^2$. Furthermore, at $r_c = 1.2a_0$ we see a ground state crossing to [Ar]$3d^{10} 4f^3$.

At $r_c = 1.3a_0$ the state [Ar]$3d^{10} 4p^1 4f^2$ flips below the initial ground state. At $r_c = 1.3a_0$ the state [Ar]$4s^2 3d^{10} 4f^1$ flips below the initial ground state. At $r_c = 1.2a_0$ the state [Ar]$3d^{10} 4d^1 4f^2$ flips below the initial ground state. At $r_c = 1.1a_0$ the state [Ar]$4s^2 3d^{10} 4d^1$ flips below the initial ground state. At $r_c = 1.0a_0$ the state [Ar]$4s^1 3d^{10} 4p^2$ flips below the initial ground state. The ionization energy of the unconfined atom is $\Delta E_0 = 5.89$ eV. The studied configurations, atomic radii and excitation energies in the unconfined calculations are as follows:

configuration	r_ϵ	r_ρ	$r_{\max}$	ΔE
[Ar]$4s^2 3d^{10} 4p^1$	5.76	4.50	2.54	0.00
[Ar]$4s^2 3d^{10} 5s^1$	10.88	3.95	6.43	2.96
[Ar]$4s^1 3d^{10} 4p^2$	5.79	4.61	2.42	4.22
[Ar]$4s^2 3d^{10} 4d^1$	16.01	3.94	8.62	4.33
[Ar]$4s^2 3d^{10} 4f^1$	25.90	3.83	16.72	5.01
[Ar]$3d^{10} 4p^3$	5.93	4.82	2.41	11.08
[Ar]$4s^1 3d^{10} 4f^2$	18.86	3.43	9.38	21.18
[Ar]$3d^{10} 4p^1 4f^2$	18.67	3.81	9.11	29.39
[Ar]$3d^{10} 4d^1 4f^2$	17.74	5.06	7.98	38.70
[Ar]$3d^{10} 4f^3$	15.50	5.54	6.50	42.77

Ge The energies of the low lying configurations of hard-wall confined Ge are shown in fig. S176 for the neutral atom and in fig. S177 for the cation. The ground state of the unconfined Ge is [Ar]$4s^2 3d^{10} 4p^2$. At $r_c = 1.3a_0$ the ground state changes to [Ar]$4s^2 3d^{10} 4p^1 4f^1$. Furthermore, at $r_c = 1.2a_0$ we see a ground state crossing to [Ar]$3d^{10} 4f^4$.

At $r_c = 1.3a_0$ the state [Ar]$4s^1 3d^{10} 4f^3$ flips below the initial ground state. At $r_c = 1.2a_0$ the state [Ar]$3d^{10} 4d^1 4f^3$ flips below the initial ground state. At $r_c = 1.2a_0$ the state [Ar]$3d^{10} 4p^1 4f^3$ flips below the initial ground state. At $r_c = 1.0a_0$ the state [Ar]$4s^1 3d^{10} 4p^3$ flips below the initial ground state. At $r_c = 1.0a_0$ the state [Ar]$4s^2 3d^{10} 4p^1 4d^1$ flips below the initial ground state. The ionization energy of the unconfined atom is $\Delta E_0 = 8.19$ eV. The studied configurations, atomic radii and excitation energies in the unconfined calculations are as follows:

configuration	r_ϵ	r_ρ	$r_{\max}$	ΔE
[Ar]$4s^2 3d^{10} 4p^2$	5.39	4.51	2.22	0.00
[Ar]$4s^1 3d^{10} 4p^3$	5.34	4.51	2.15	4.49
[Ar]$4s^2 3d^{10} 4p^1 5s^1$	10.11	4.45	5.86	4.98
[Ar]$4s^2 3d^{10} 4p^1 4d^1$	14.88	4.18	7.34	6.48
[Ar]$4s^2 3d^{10} 4p^1 4f^1$	25.78	4.00	16.60	7.31
[Ar]$4s^1 3d^{10} 4f^3$	15.47	5.66	6.39	43.24
[Ar]$3d^{10} 4p^1 4f^3$	15.46	5.74	6.26	53.22
[Ar]$3d^{10} 4d^1 4f^3$	15.10	6.18	5.81	65.82
[Ar]$3d^{10} 4f^4$	13.50	6.70	4.96	72.34

As The energies of the low lying configurations of hard-wall confined As are shown in fig. S178 for the neutral atom and in fig. S179 for the cation. The ground state of the unconfined As is [Ar]$4s^2 3d^{10} 4p^3$. At $r_c = 1.2a_0$ the ground state changes to [Ar]$3d^{10} 4f^5$.

At $r_c = 1.2a_0$ the state [Ar]$3d^{10} 4d^1 4f^4$ flips below the initial ground state. At $r_c = 1.2a_0$ the state [Ar]$4s^1 3d^{10} 4f^4$ flips below the initial ground state. At $r_c = 1.2a_0$ the state [Ar]$4s^2 3d^{10} 4f^3$ flips below the initial ground state. At $r_c = 1.2a_0$ the state [Ar]$4s^2 3d^{10} 4p^2 4f^1$ flips below the initial ground state. At $r_c = 1.0a_0$ the state [Ar]$4s^2 3d^{10} 4p^2 4d^1$ flips below the initial ground state. The ionization energy of the unconfined atom is $\Delta E_0 = 10.31$ eV. The studied configurations, atomic radii and excitation energies in the unconfined calculations are as follows:

configuration	r_ϵ	r_ρ	$r_{\max}$	ΔE
[Ar]$4s^2 3d^{10} 4p^3$	5.01	4.37	1.99	0.00
[Ar]$4s^2 3d^{10} 4p^2 5s^1$	9.58	4.64	5.44	6.88
[Ar]$4s^2 3d^{10} 4p^2 4d^1$	14.34	4.22	6.54	8.51
[Ar]$4s^1 3d^{10} 4p^4$	5.28	4.51	2.04	8.89
[Ar]$4s^2 3d^{10} 4p^2 4f^1$	25.81	4.00	16.63	9.42
[Ar]$4s^2 3d^{10} 4f^3$	15.46	5.75	6.36	42.94
[Ar]$4s^1 3d^{10} 4f^4$	13.52	6.77	4.84	75.13
[Ar]$3d^{10} 4d^1 4f^4$	13.38	6.63	4.51	102.60
[Ar]$3d^{10} 4f^5$	12.16	6.66	4.02	111.83

Se The energies of the low lying configurations of hard-wall confined Se are shown in fig. S180 for the neutral atom and in fig. S181 for the cation. The ground state of the unconfined Se is [Ar]$4s^2 3d^{10} 4p^4$. At $r_c = 1.2 a_0$ the ground state changes to [Ar]$4s^2 3d^{10} 4f^4$. Furthermore, at $r_c = 1.1 a_0$ we see a ground state crossing to [Ar]$3d^{10} 4f^6$.

At $r_c = 1.2 a_0$ the state [Ar]$4s^1 3d^{10} 4f^5$ flips below the initial ground state. At $r_c = 1.2 a_0$ the state [Ar]$4s^2 3d^{10} 4p^1 4f^3$ flips below the initial ground state. At $r_c = 1.2 a_0$ the state [Ar]$4s^2 3d^{10} 4p^3 4f^1$ flips below the initial ground state. At $r_c = 1.1 a_0$ the state [Ar]$3d^{10} 4d^1 4f^5$ flips below the initial ground state. At $r_c = 1.0 a_0$ the state [Ar]$4s^2 3d^{10} 4p^3 4d^1$ flips below the initial ground state. The ionization energy of the unconfined atom is $\Delta E_0 = 9.33$ eV. The studied configurations, atomic radii and excitation energies in the unconfined calculations are as follows:

configuration	r_ϵ	r_ρ	$r_{\max}$	ΔE
[Ar]$4s^2 3d^{10} 4p^4$	4.82	4.28	1.81	0.00
[Ar]$4s^2 3d^{10} 4p^3 5s^1$	9.14	4.64	5.09	5.72
[Ar]$4s^2 3d^{10} 4p^3 4d^1$	14.04	4.17	5.90	7.47
[Ar]$4s^2 3d^{10} 4p^3 4f^1$	25.84	3.92	16.65	8.45
[Ar]$4s^1 3d^{10} 4p^5$	4.92	4.34	1.84	10.36
[Ar]$4s^2 3d^{10} 4p^1 4f^3$	15.46	5.69	6.28	48.72
[Ar]$4s^2 3d^{10} 4f^4$	13.52	6.61	4.80	74.35
[Ar]$4s^1 3d^{10} 4f^5$	12.24	6.77	3.91	114.36
[Ar]$3d^{10} 4d^1 4f^5$	12.16	6.63	3.65	146.57
[Ar]$3d^{10} 4f^6$	11.19	6.46	3.38	158.73

Br The energies of the low lying configurations of hard-wall confined Br are shown in fig. S182 for the neutral atom and in fig. S183 for the cation. The ground state of the unconfined Br is [Ar]$4s^2 3d^{10} 4p^5$. At $r_c = 1.2 a_0$ the ground state changes to [Ar]$4s^2 3d^{10} 4p^3 4f^2$. Furthermore, at $r_c = 1.1 a_0$ we see a ground state crossing to [Ar]$3d^{10} 4f^7$.

At $r_c = 1.2 a_0$ the state [Ar]$4s^2 3d^{10} 4p^2 4f^3$ flips below the initial ground state. At $r_c = 1.2 a_0$ the state [Ar]$4s^2 3d^{10} 4p^4 4f^1$ flips below the initial ground state. At $r_c = 1.1 a_0$ the state [Ar]$4s^1 3d^{10} 4f^6$ flips below the initial ground state. At $r_c = 1.1 a_0$ the state [Ar]$3d^{10} 4p^1 4f^6$ flips below the initial ground state. At $r_c = 1.0 a_0$ the state [Ar]$4s^2 3d^{10} 4p^4 4d^1$ flips below the initial ground state. The ionization energy of the unconfined atom is $\Delta E_0 = 12.01$ eV. The studied configurations, atomic radii and excitation energies in the unconfined calculations are as follows:

configuration	r_ϵ	r_ρ	$r_{\max}$	ΔE
[Ar]$4s^2 3d^{10} 4p^5$	4.56	4.16	1.68	0.00
[Ar]$4s^2 3d^{10} 4p^4 5s^1$	8.77	4.72	4.82	8.23
[Ar]$4s^2 3d^{10} 4p^4 4d^1$	14.21	4.11	6.06	10.17
[Ar]$4s^2 3d^{10} 4p^4 4f^1$	25.85	3.88	16.67	11.13
[Ar]$4s^1 3d^{10} 4p^6$	4.59	4.18	1.68	11.87
[Ar]$4s^2 3d^{10} 4p^3 4f^2$	18.80	3.66	9.28	28.15
[Ar]$4s^2 3d^{10} 4p^2 4f^3$	15.47	5.75	6.24	54.30
[Ar]$4s^1 3d^{10} 4f^6$	11.32	6.48	3.27	164.50
[Ar]$3d^{10} 4p^1 4f^6$	11.35	6.48	3.22	179.42
[Ar]$3d^{10} 4f^7$	10.46	6.29	2.92	216.57

Kr The energies of the low lying configurations of hard-wall confined Kr are shown in fig. S184 for the neutral atom and in fig. S185 for the cation. The ground state of the unconfined Kr is [Ar]$4s^2 3d^{10} 4p^6$. At $r_c = 1.1 a_0$ the ground state changes to [Ar]$4s^1 3d^{10} 4f^7$. Furthermore, at $r_c = 1.0 a_0$ we see a ground state crossing to [Ar]$3d^{10} 4f^8$.

At $r_c = 1.1 a_0$ the state [Ar]$3d^{10} 4p^1 4f^7$ flips below the initial ground state. At $r_c = 1.1 a_0$ the state [Ar]$4s^2 3d^{10} 4p^5 4f^1$ flips below the initial ground state. At $r_c = 1.1 a_0$ the state [Ar]$4s^2 3d^{10} 4p^4 4f^2$ flips below the initial ground state. The ionization energy of the unconfined atom is $\Delta E_0 = 14.46$ eV. The studied configurations, atomic radii and excitation energies in the unconfined calculations are as follows:

configuration	r_ϵ	r_ρ	$r_{\max}$	ΔE
[Ar]$4s^2 3d^{10} 4p^6$	4.31	4.03	1.56	0.00
[Ar]$4s^2 3d^{10} 4p^5 5s^1$	8.51	4.65	4.64	10.54
[Ar]$4s^2 3d^{10} 4p^5 4d^1$	14.48	4.01	6.51	12.65
[Ar]$4s^2 3d^{10} 4p^5 4f^1$	25.86	3.74	16.62	13.57
[Ar]$4s^1 3d^{10} 4p^6 4d^1$	14.34	4.07	6.18	26.52
[Ar]$4s^2 3d^{10} 4p^4 4f^2$	18.81	3.56	9.27	34.12
[Ar]$4s^1 3d^{10} 4f^7$	10.62	6.33	2.81	225.83
[Ar]$3d^{10} 4p^1 4f^7$	10.64	6.33	2.77	242.35
[Ar]$3d^{10} 4f^8$	Not converged			

Rb The energies of the low lying configurations of hard-wall confined Rb are shown in fig. S186 for the neutral atom and in fig. S187 for the cation. The ground state of the unconfined Rb is [Kr]$5s^1$. At $r_c = 4.9a_0$ the ground state changes to [Kr]$4d^1$. Furthermore, at $r_c = 1.2a_0$ we see a ground state crossing to [Kr]$4f^1$. At $r_c = 1.1a_0$ we see a ground state crossing to [Ar]$4s^1 3d^{10} 4f^8$. Moreover, at $r_c = 1.0a_0$ we see a ground state crossing to [Ar]$3d^{10} 4f^9$.

At $r_c = 3.0a_0$ the state [Ar]$4s^2 3d^{10} 4p^5 4d^2$ flips below the initial ground state. At $r_c = 2.0a_0$ the state [Ar]$4s^2 3d^{10} 4p^5 4f^2$ flips below the initial ground state. At $r_c = 1.3a_0$ the state [Ar]$3d^{10} 4p^1 4f^8$ flips below the initial ground state. At $r_c = 1.1a_0$ the state [Kr]$5p^1$ flips below the initial ground state. The ionization energy of the unconfined atom is $\Delta E_0 = 4.02$ eV. The studied configurations, atomic radii and excitation energies in the unconfined calculations are as follows:

configuration	r_ϵ	r_ρ	$r_{\max}$	ΔE
[Kr]$5s^1$	8.31	4.65	4.50	0.00
[Kr]$5p^1$	11.32	3.88	6.36	1.48
[Kr]$4d^1$	14.78	3.88	6.99	2.26
[Kr]$4f^1$	25.88	3.72	16.70	3.14
[Ar]$4s^2 3d^{10} 4p^5 4d^2$	9.81	4.80	2.38	19.75
[Ar]$4s^2 3d^{10} 4p^5 4f^2$	18.83	3.45	9.33	26.92
[Ar]$4s^1 3d^{10} 4f^8$	24.52	5.75	15.16	287.82
[Ar]$3d^{10} 4p^1 4f^8$	24.57	5.66	15.08	305.91
[Ar]$3d^{10} 4f^9$	Not converged			

Sr The energies of the low lying configurations of hard-wall confined Sr are shown in fig. S188 for the neutral atom and in fig. S189 for the cation. The ground state of the unconfined Sr is [Kr]$5s^2$. At $r_c = 5.4a_0$ the ground state changes to [Kr]$5s^1 4d^1$. Furthermore, at $r_c = 4.9a_0$ we see a ground state crossing to [Kr]$4d^2$. At $r_c = 1.2a_0$ we see a ground state crossing to [Kr]$4f^2$. Moreover, at $r_c = 1.0a_0$ we see a ground state crossing to [Ar]$4s^1 3d^{10} 4f^9$.

At $r_c = 3.6a_0$ the state [Kr]$4d^1 5p^1$ flips below the initial ground state. At $r_c = 3.5a_0$ the state [Kr]$4d^1 4f^1$ flips below the initial ground state. At $r_c = 3.5a_0$ the state [Ar]$4s^2 3d^{10} 4p^5 4d^3$ flips below the initial ground state. At $r_c = 1.5a_0$ the state [Ar]$4s^2 3d^{10} 4f^8$ flips below the initial ground state. At $r_c = 1.3a_0$ the state [Ar]$3d^{10} 4f^{10}$ flips below the initial ground state. At $r_c = 1.1a_0$ the state [Kr]$5s^1 5p^1$ flips below the initial ground state. The ionization energy of the unconfined atom is $\Delta E_0 = 5.35$ eV. The studied configurations, atomic radii and excitation energies in the unconfined calculations are as follows:

configuration	r_ϵ	r_ρ	$r_{\max}$	ΔE
[Kr]$5s^2$	7.76	5.39	3.83	0.00
[Kr]$5s^1 5p^1$	8.72	5.50	4.68	1.47
[Kr]$5s^1 4d^1$	8.11	5.10	3.86	1.56
[Kr]$4d^1 5p^1$	9.61	5.03	5.09	3.70
[Kr]$4d^2$	9.93	4.67	2.23	3.93
[Kr]$4d^1 4f^1$	25.27	4.07	16.05	6.06
[Kr]$4f^2$	18.84	3.39	9.36	11.18
[Ar]$4s^2 3d^{10} 4p^5 4d^3$	7.86	4.89	2.00	24.25
[Ar]$4s^2 3d^{10} 4f^8$	24.75	5.75	15.41	281.66
[Ar]$4s^1 3d^{10} 4f^9$	18.40	5.72	2.84	354.60
[Ar]$3d^{10} 4f^{10}$	15.58	5.81	2.16	433.35

Y The energies of the low lying configurations of hard-wall confined Y are shown in fig. S190 for the neutral atom and in fig. S191 for the cation. The ground state of the unconfined Y is [Kr]$5s^2 4d^1$. At $r_c = 6.7a_0$ the ground state changes to [Kr]$5s^1 4d^2$. Furthermore, at $r_c = 4.9a_0$ we see a ground state crossing to [Kr]$4d^3$. At $r_c = 1.1a_0$ we see a ground state crossing to [Kr]$4d^1 4f^2$. Moreover, at $r_c = 1.0a_0$ we see a ground state crossing to [Ar]$4s^2 3d^{10} 4f^9$.

At $r_c = 3.7a_0$ the state [Kr]$4d^2 5p^1$ flips below the initial ground state. At $r_c = 3.4a_0$ the state [Kr]$4d^2 4f^1$ flips below the initial ground state. At $r_c = 3.4a_0$ the state [Ar]$4s^2 3d^{10} 4p^5 4d^4$ flips

below the initial ground state. At $r_c = 1.5a_0$ the state [Ar]$4s^2 3d^{10} 4p^1 4f^8$ flips below the initial ground state. At $r_c = 1.3a_0$ the state [Ar]$4s^1 3d^{10} 4f^{10}$ flips below the initial ground state. At $r_c = 1.1a_0$ the state [Kr]$5s^1 4d^1 5p^1$ flips below the initial ground state. The ionization energy of the unconfined atom is $\Delta E_0 = 5.74$ eV. The studied configurations, atomic radii and excitation energies in the unconfined calculations are as follows:

configuration	r_ϵ	r_ρ	$r_{\max}$	ΔE
[Kr]$5s^2 4d^1$	7.25	5.31	3.56	0.00
[Kr]$5s^1 4d^2$	7.13	5.06	3.52	0.37
[Kr]$5s^1 4d^1 5p^1$	8.03	5.44	4.22	1.47
[Kr]$4d^3$	7.88	4.84	1.88	2.31
[Kr]$4d^2 5p^1$	8.85	5.12	4.62	2.92
[Kr]$4d^2 4f^1$	25.44	4.15	16.28	5.53
[Kr]$4d^1 4f^2$	18.69	3.65	9.12	12.81
[Ar]$4s^2 3d^{10} 4p^5 4d^4$	6.77	4.79	1.77	25.04
[Ar]$4s^2 3d^{10} 4p^1 4f^8$	24.79	5.59	15.40	281.24
[Ar]$4s^1 3d^{10} 4f^{10}$	15.96	5.81	2.28	424.81
[Ar]$4s^2 3d^{10} 4f^9$	Not converged			

Zr The energies of the low lying configurations of hard-wall confined Zr are shown in fig. S192 for the neutral atom and in fig. S193 for the cation. The ground state of the unconfined Zr is [Kr]$5s^1 4d^3$. At $r_c = 5.1a_0$ the ground state changes to [Kr]$4d^4$. Furthermore, at $r_c = 1.1a_0$ we see a ground state crossing to [Kr]$4d^2 4f^2$. At $r_c = 1.0a_0$ we see a ground state crossing to [Ar]$4s^2 3d^{10} 4p^3 4f^7$.

At $r_c = 2.8a_0$ the state [Ar]$4s^2 3d^{10} 4p^5 4d^5$ flips below the initial ground state. At $r_c = 2.6a_0$ the state [Kr]$4d^3 4f^1$ flips below the initial ground state. At $r_c = 1.2a_0$ the state [Ar]$4s^2 3d^{10} 4p^2 4f^8$ flips below the initial ground state. At $r_c = 1.2a_0$ the state [Ar]$4s^2 3d^{10} 4p^1 4f^9$ flips below the initial ground state. At $r_c = 1.0a_0$ the state [Kr]$4d^3 5p^1$ flips below the initial ground state. The ionization energy of the unconfined atom is $\Delta E_0 = 6.48$ eV. The studied configurations, atomic radii and excitation energies in the unconfined calculations are as follows:

configuration	r_ϵ	r_ρ	$r_{\max}$	ΔE
[Kr]$5s^1 4d^3$	6.55	4.93	3.28	0.00
[Kr]$5s^2 4d^2$	6.90	5.15	3.40	0.88
[Kr]$4d^4$	6.74	4.73	1.67	1.33
[Kr]$4d^3 5p^1$	8.49	5.03	4.34	2.86
[Kr]$4d^3 4f^1$	25.56	4.09	16.35	5.59
[Kr]$4d^2 4f^2$	18.67	3.76	9.08	14.82
[Ar]$4s^2 3d^{10} 4p^5 4d^5$	6.05	4.61	1.61	26.25
[Ar]$4s^2 3d^{10} 4p^3 4f^7$	11.05	6.14	2.46	209.77
[Ar]$4s^2 3d^{10} 4p^2 4f^8$	24.83	5.53	15.44	275.68
[Ar]$4s^2 3d^{10} 4p^1 4f^9$	18.56	5.64	2.82	344.59

Nb The energies of the low lying configurations of hard-wall confined Nb are shown in fig. S194 for the neutral atom and in fig. S195 for the cation. The ground state of the unconfined Nb is [Kr]$5s^1 4d^4$. At $r_c = 5.7a_0$ the ground state changes to [Kr]$4d^5$. Furthermore, at $r_c = 1.1a_0$ we see a ground state crossing to [Kr]$4f^5$. At $r_c = 1.0a_0$ we see a ground state crossing to [Ar]$4s^2 3d^{10} 4p^4 4f^7$.

At $r_c = 2.6a_0$ the state [Ar]$4s^2 3d^{10} 4p^5 4d^6$ flips below the initial ground state. At $r_c = 2.5a_0$ the state [Kr]$4d^4 4f^1$ flips below the initial ground state. At $r_c = 2.0a_0$ the state [Kr]$4d^3 4f^2$ flips below the initial ground state. At $r_c = 1.4a_0$ the state [Ar]$4s^2 3d^{10} 4p^5 4f^6$ flips below the initial ground state. At $r_c = 1.0a_0$ the state [Kr]$4d^4 5p^1$ flips below the initial ground state. The ionization energy of the unconfined atom is $\Delta E_0 = 6.79$ eV. The studied configurations, atomic radii and excitation energies in the unconfined calculations are as follows:

configuration	r_ϵ	r_ρ	$r_{\max}$	ΔE
[Kr]$5s^1 4d^4$	6.16	4.84	3.11	0.00
[Kr]$4d^5$	6.00	4.61	1.52	0.60
[Kr]$5s^2 4d^3$	6.65	5.06	3.27	2.14
[Kr]$4d^4 5p^1$	8.30	4.93	4.15	3.10
[Kr]$4d^4 4f^1$	25.65	4.07	16.46	5.90
[Kr]$4d^3 4f^2$	18.69	3.75	9.05	16.99
[Ar]$4s^2 3d^{10} 4p^5 4d^6$	8.37	4.54	1.49	31.82
[Kr]$4f^5$	12.49	6.54	3.70	76.72
[Ar]$4s^2 3d^{10} 4p^5 4f^6$	11.70	6.40	2.95	137.96
[Ar]$4s^2 3d^{10} 4p^4 4f^7$	11.11	6.14	2.44	200.58

Mo The energies of the low lying configurations of hard-wall confined Mo are shown in

fig. S196 for the neutral atom and in fig. S197 for the cation. The ground state of the unconfined Mo is [Kr]$5s^1 4d^5$. At $r_c = 4.0a_0$ the ground state changes to [Kr]$4d^6$. Furthermore, at $r_c = 1.1a_0$ we see a ground state crossing to [Kr]$4d^5 4f^1$. At $r_c = 1.0a_0$ we see a ground state crossing to [Kr]$4f^6$.

At $r_c = 2.4a_0$ the state [Ar]$4s^2 3d^{10} 4p^5 4d^7$ flips below the initial ground state. At $r_c = 1.9a_0$ the state [Kr]$4d^4 4f^2$ flips below the initial ground state. At $r_c = 1.4a_0$ the state [Kr]$4d^1 4f^5$ flips below the initial ground state. At $r_c = 1.3a_0$ the state [Ar]$4s^2 3d^{10} 4p^5 4f^7$ flips below the initial ground state. At $r_c = 1.0a_0$ the state [Kr]$4d^5 5p^1$ flips below the initial ground state. The ionization energy of the unconfined atom is $\Delta E_0 = 7.03$ eV. The studied configurations, atomic radii and excitation energies in the unconfined calculations are as follows:

configuration	r_ϵ	r_ρ	$r_{\max}$	ΔE
[Kr]$5s^1 4d^5$	5.88	4.72	2.97	0.00
[Kr]$4d^5 5p^1$	8.19	4.85	4.01	3.30
[Kr]$5s^2 4d^4$	6.46	4.99	3.18	3.40
[Kr]$4d^6$	6.64	4.50	1.39	3.49
[Kr]$4d^5 4f^1$	25.72	3.92	16.44	6.15
[Kr]$4d^4 4f^2$	18.71	3.65	9.09	19.02
[Ar]$4s^2 3d^{10} 4p^5 4d^7$	6.13	4.50	1.38	37.75
[Kr]$4d^1 4f^5$	12.58	6.54	3.58	87.26
[Kr]$4f^6$	11.71	6.40	2.97	116.01
[Ar]$4s^2 3d^{10} 4p^5 4f^7$	11.15	6.27	2.44	185.12

Tc The energies of the low lying configurations of hard-wall confined Tc are shown in fig. S198 for the neutral atom and in fig. S199 for the cation. The ground state of the unconfined Tc is [Kr]$5s^1 4d^6$. At $r_c = 4.4a_0$ the ground state changes to [Kr]$4d^7$. Furthermore, at $r_c = 1.0a_0$ we see a ground state crossing to [Kr]$4f^7$.

At $r_c = 2.4a_0$ the state [Kr]$4d^6 4f^1$ flips below the initial ground state. At $r_c = 2.3a_0$ the state [Ar]$4s^2 3d^{10} 4p^5 4d^8$ flips below the initial ground state. At $r_c = 1.9a_0$ the state [Kr]$4d^5 4f^2$ flips below the initial ground state. At $r_c = 1.4a_0$ the state [Kr]$4d^2 4f^5$ flips below the initial ground state. At $r_c = 1.3a_0$ the state [Kr]$4d^1 4f^6$ flips below the initial ground state. At $r_c = 1.0a_0$ the state [Kr]$4d^6 5p^1$ flips below the initial ground state. The ionization energy of the unconfined atom is $\Delta E_0 = 6.43$ eV. The studied configurations, atomic radii and excitation energies in the unconfined calculations are as follows:

configuration	r_ϵ	r_ρ	$r_{\max}$	ΔE
[Kr]$5s^1 4d^6$	5.77	4.65	2.88	0.00
[Kr]$5s^2 4d^5$	6.31	4.92	3.10	0.10
[Kr]$4d^7$	5.70	4.44	1.30	2.01
[Kr]$4d^6 5p^1$	8.12	4.79	3.92	3.38
[Kr]$4d^6 4f^1$	25.75	3.87	16.51	6.26
[Kr]$4d^5 4f^2$	18.74	3.61	9.14	16.38
[Ar]$4s^2 3d^{10} 4p^5 4d^8$	5.47	4.37	1.29	39.33
[Kr]$4d^2 4f^5$	12.65	6.40	3.50	93.09
[Kr]$4d^1 4f^6$	11.83	6.40	2.85	125.20
[Kr]$4f^7$	11.17	6.27	2.44	159.14

Ru The energies of the low lying configurations of hard-wall confined Ru are shown in fig. S200 for the neutral atom and in fig. S201 for the cation. The ground state of the unconfined Ru is [Kr]$5s^1 4d^7$. At $r_c = 5.3a_0$ the ground state changes to [Kr]$4d^8$. Furthermore, at $r_c = 1.0a_0$ we see a ground state crossing to [Kr]$4d^2 4f^6$.

At $r_c = 2.3a_0$ the state [Kr]$4d^7 4f^1$ flips below the initial ground state. At $r_c = 2.3a_0$ the state [Ar]$4s^2 3d^{10} 4p^5 4d^9$ flips below the initial ground state. At $r_c = 1.4a_0$ the state [Kr]$4d^3 4f^5$ flips below the initial ground state. At $r_c = 1.3a_0$ the state [Kr]$4d^1 4f^7$ flips below the initial ground state. The ionization energy of the unconfined atom is $\Delta E_0 = 7.18$ eV. The studied configurations, atomic radii and excitation energies in the unconfined calculations are as follows:

configuration	r_ϵ	r_ρ	$r_{\max}$	ΔE
[Kr]$5s^1 4d^7$	5.64	4.55	2.82	0.00
[Kr]$4d^8$	5.24	4.30	1.22	0.68
[Kr]$5s^2 4d^6$	6.04	4.82	2.89	1.46
[Kr]$4d^7 5p^1$	8.12	4.67	3.88	3.43
[Kr]$4d^7 4f^1$	25.78	3.82	16.55	6.30
[Ar]$4s^2 3d^{10} 4p^5 4d^9$	5.05	4.20	1.21	41.07
[Kr]$4d^3 4f^5$	12.72	6.43	3.45	98.80
[Kr]$4d^2 4f^6$	11.94	6.27	2.76	134.29
[Kr]$4d^1 4f^7$	11.33	6.14	2.33	171.73

Rh The energies of the low lying configurations of hard-wall confined Rh are shown in fig. S202 for the neutral atom and in fig. S203 for the cation. The ground state of the unconfined Rh is [Kr]$4d^9$. At $r_c = 1.0a_0$ the ground state changes to [Kr]$4d^5 4f^4$.

At $r_c = 1.0a_0$ the state [Kr]$4d^6 4f^3$ flips below the initial ground state. At $r_c = 1.0a_0$ the state [Kr]$4d^4 4f^5$ flips below the initial ground state. At $r_c = 1.0a_0$ the state [Kr]$4d^8 4f^1$ flips below the initial ground state. The ionization energy of the unconfined atom is $\Delta E_0 = 7.81$ eV. The studied configurations, atomic radii and excitation energies in the unconfined calculations are as follows:

configuration	r_ϵ	r_ρ	$r_{\max}$	ΔE
[Kr]$4d^9$	4.90	4.15	1.15	0.00
[Kr]$5s^1 4d^8$	5.55	4.47	2.76	0.61
[Kr]$5s^2 4d^7$	5.86	4.72	2.76	3.42
[Kr]$4d^8 5p^1$	8.14	4.61	3.86	4.08
[Kr]$4d^8 4f^1$	25.81	3.72	16.58	6.93
[Kr]$4d^6 4f^3$	15.50	5.61	6.20	42.72
[Ar]$4s^2 3d^{10} 4p^5 4d^{10}$	4.75	4.11	1.15	43.49
[Kr]$4d^5 4f^4$	13.85	6.33	4.54	68.91
[Kr]$4d^4 4f^5$	12.77	6.30	3.42	104.94

Pd The energies of the low lying configurations of hard-wall confined Pd are shown in fig. S204 for the neutral atom and in fig. S205 for the cation. The ground state of the unconfined Pd is [Kr]$4d^{10}$. We do not observe any ground state crossing for Pd in the considered confinement radii.

The ionization energy of the unconfined atom is $\Delta E_0 = 9.08$ eV. The studied configurations, atomic radii and excitation energies in the unconfined calculations are as follows:

configuration	r_ϵ	r_ρ	$r_{\max}$	ΔE
[Kr]$4d^{10}$	4.64	4.04	1.09	0.00
[Kr]$5s^1 4d^9$	5.49	4.39	2.72	1.88
[Kr]$4d^9 5p^1$	8.16	4.51	3.85	5.38
[Kr]$5s^2 4d^8$	5.72	4.67	2.64	6.05
[Kr]$4d^9 4f^1$	25.83	3.67	16.60	8.20
[Kr]$4d^8 4f^2$	18.80	3.41	9.25	24.67
[Ar]$4s^2 3d^{10} 4p^5 4d^{10} 4f^1$	25.84	3.65	16.54	55.50

Ag The energies of the low lying configurations of hard-wall confined Ag are shown in fig. S206 for the neutral atom and in fig. S207 for the cation. The ground state of the unconfined Ag is [Kr]$5s^1 4d^{10}$. At $r_c = 2.1a_0$ the ground state changes to [Kr]$4d^{10} 4f^1$.

At $r_c = 1.7a_0$ the state [Kr]$4d^9 4f^2$ flips below the initial ground state. At $r_c = 1.5a_0$ the state [Kr]$4d^8 4f^3$ flips below the initial ground state. The ionization energy of the unconfined atom is $\Delta E_0 = 7.21$ eV. The studied configurations, atomic radii and excitation energies in the unconfined calculations are as follows:

configuration	r_ϵ	r_ρ	$r_{\max}$	ΔE
[Kr]$5s^1 4d^{10}$	5.44	4.31	2.68	0.00
[Kr]$4d^{10} 5p^1$	8.19	4.47	3.85	3.53
[Kr]$5s^2 4d^9$	5.61	4.56	2.55	5.54
[Kr]$4d^{10} 4f^1$	25.85	3.59	16.67	6.33
[Kr]$4d^9 4f^2$	18.81	3.36	9.27	24.71
[Kr]$4d^8 4f^3$	15.49	5.62	6.27	51.32

Cd The energies of the low lying configurations of hard-wall confined Cd are shown in fig. S208 for the neutral atom and in fig. S209 for the cation. The ground state of the unconfined Cd is [Kr]$5s^2 4d^{10}$. At $r_c = 2.0a_0$ the ground state changes to [Kr]$4d^{10} 4f^2$.

At $r_c = 2.0a_0$ the state [Kr]$5s^1 4d^{10} 4f^1$ flips below the initial ground state. At $r_c = 1.8a_0$ the state [Kr]$4d^9 4f^3$ flips below the initial ground state. At $r_c = 1.8a_0$ the state [Kr]$4d^{10} 5p^1 4f^1$ flips below the initial ground state. At $r_c = 1.6a_0$ the state [Kr]$4d^8 4f^4$ flips below the initial ground state. At $r_c = 1.6a_0$ the state [Ar]$4s^2 3d^{10} 4p^5 4d^{10} 4f^3$ flips below the initial ground state. The ionization energy of the unconfined atom is $\Delta E_0 = 8.30$ eV. The studied configurations, atomic radii and excitation energies in the unconfined calculations are as follows:

configuration	r_ϵ	r_ρ	$r_{\max}$	ΔE
[Kr]$5s^2 4d^{10}$	5.52	4.54	2.47	0.00
[Kr]$5s^1 4d^{10} 5p^1$	6.63	4.73	3.12	3.32
[Kr]$5s^1 4d^{10} 5d^1$	15.94	4.07	8.57	6.75
[Kr]$5s^1 4d^{10} 4f^1$	25.88	3.95	16.59	7.42
[Kr]$4d^{10} 5p^2$	7.12	5.03	3.16	7.89
[Kr]$4d^{10} 5p^1 4f^1$	25.39	4.31	16.19	12.71
[Kr]$4d^{10} 4f^2$	18.83	3.24	9.32	19.65
[Kr]$4d^9 4f^3$	15.49	5.64	6.31	48.80
[Kr]$4d^8 4f^4$	13.73	6.54	4.65	85.28
[Ar]$4s^2 3d^{10} 4p^5 4d^{10} 4f^3$	15.49	5.64	6.31	104.11

In The energies of the low lying configurations of hard-wall confined In are shown in fig. S210 for the neutral atom and in fig. S211 for the cation. The ground state of the unconfined In is [Kr]$5s^2 4d^{10} 5p^1$. At $r_c = 2.2a_0$ the ground state changes to [Kr]$5s^2 4d^{10} 4f^1$. Furthermore, at $r_c = 2.0a_0$ we see a ground state crossing to [Kr]$4d^{10} 4f^3$.

At $r_c = 2.1a_0$ the state [Kr]$5s^1 4d^{10} 4f^2$ flips below the initial ground state. At $r_c = 2.0a_0$ the state [Kr]$4d^{10} 5p^1 4f^2$ flips below the initial ground state. At $r_c = 1.9a_0$ the state [Kr]$4d^9 4f^4$ flips below the initial ground state. At $r_c = 1.7a_0$ the state [Kr]$4d^8 4f^5$ flips below the initial ground state. At $r_c = 1.7a_0$ the state [Ar]$4s^2 3d^{10} 4p^5 4d^{10} 4f^4$ flips below the initial ground state. At $r_c = 1.0a_0$ the state [Kr]$5s^2 4d^{10} 5d^1$ flips below the initial ground state. The ionization energy of the unconfined atom is $\Delta E_0 = 5.57$ eV. The studied configurations, atomic radii and excitation energies in the unconfined calculations are as follows:

configuration	r_ϵ	r_ρ	$r_{\max}$	ΔE
[Kr]$5s^2 4d^{10} 5p^1$	6.20	4.74	2.89	0.00
[Kr]$5s^1 4d^{10} 5p^2$	6.27	4.86	2.77	3.42
[Kr]$5s^2 4d^{10} 5d^1$	15.66	4.26	8.25	3.98
[Kr]$5s^2 4d^{10} 4f^1$	25.85	4.15	16.68	4.69
[Kr]$4d^{10} 5p^3$	6.44	5.02	2.77	9.03
[Kr]$5s^1 4d^{10} 4f^2$	18.73	3.75	9.15	18.49
[Kr]$4d^{10} 5p^1 4f^2$	18.58	4.23	8.85	25.19
[Kr]$4d^{10} 4f^3$	15.49	5.64	6.33	36.15
[Kr]$4d^9 4f^4$	13.69	6.54	4.69	75.83
[Kr]$4d^8 4f^5$	12.76	6.47	3.58	121.01
[Ar]$4s^2 3d^{10} 4p^5 4d^{10} 4f^4$	13.70	6.54	4.68	135.17
[Kr]$5s^2 4d^{10} 6s^1$	Not converged			

Sn The energies of the low lying configurations of hard-wall confined Sn are shown in fig. S212 for the neutral atom and in fig. S213 for the cation. The ground state of the unconfined Sn is [Kr]$5s^2 4d^{10} 5p^2$. At $r_c = 2.2a_0$ the ground state changes to [Kr]$5s^2 4d^{10} 4f^2$. Furthermore, at $r_c = 2.0a_0$ we see a ground state crossing to [Kr]$5s^1 4d^{10} 4f^3$. At $r_c = 1.9a_0$ we see a ground state crossing to [Kr]$4d^{10} 4f^4$.

At $r_c = 2.2a_0$ the state [Kr]$5s^2 4d^{10} 5p^1 4f^1$ flips below the initial ground state. At $r_c = 1.9a_0$ the state [Kr]$4d^9 4f^5$ flips below the initial ground state. At $r_c = 1.8a_0$ the state [Kr]$4d^8 4f^6$ flips below the initial ground state. At $r_c = 1.8a_0$ the state [Ar]$4s^2 3d^{10} 4p^5 4d^{10} 4f^5$ flips below the initial ground state. The ionization energy of the unconfined atom is $\Delta E_0 = 7.56$ eV. The studied configurations, atomic radii and excitation energies in the unconfined calculations are as follows:

configuration	r_ϵ	r_ρ	$r_{\max}$	ΔE
[Kr]$5s^2 4d^{10} 5p^2$	5.90	4.80	2.59	0.00
[Kr]$5s^1 4d^{10} 5p^3$	5.86	4.82	2.52	3.51
[Kr]$5s^2 4d^{10} 5p^1 6s^1$	10.90	4.63	6.47	4.61
[Kr]$5s^2 4d^{10} 5p^1 5d^1$	14.23	4.63	6.65	5.78
[Kr]$5s^2 4d^{10} 5p^1 4f^1$	25.70	4.32	16.51	6.68
[Kr]$5s^2 4d^{10} 4f^2$	18.71	3.91	9.11	16.77
[Kr]$5s^1 4d^{10} 4f^3$	15.56	5.58	6.14	36.17
[Kr]$4d^{10} 4f^4$	13.66	6.57	4.72	58.47
[Kr]$4d^9 4f^5$	12.72	6.47	3.64	107.52
[Kr]$4d^8 4f^6$	12.16	6.25	2.76	160.01
[Ar]$4s^2 3d^{10} 4p^5 4d^{10} 4f^5$	12.75	6.47	3.62	170.80

Sb The energies of the low lying configurations of hard-wall confined Sb are shown in fig. S214 for the neutral atom and in fig. S215 for the cation. The ground state of the unconfined Sb is [Kr]$5s^2 4d^{10} 5p^3$. At $r_c = 2.1a_0$ the ground state changes to [Kr]$5s^2 4d^{10} 4f^3$. Furthermore, at $r_c = 1.9a_0$ we see a ground state crossing to [Kr]$4d^{10} 4f^5$.

At $r_c = 2.1a_0$ the state [Kr]$5s^1 4d^{10} 4f^4$ flips below the initial ground state. At $r_c = 2.1a_0$ the state [Kr]$5s^2 4d^{10} 5p^2 4f^1$ flips below the initial ground state. At $r_c = 2.1a_0$ the state [Kr]$5s^2 4d^{10} 5p^1 4f^2$ flips below the initial ground state. At $r_c = 1.9a_0$ the state [Kr]$4d^9 4f^6$ flips below the initial ground state. At $r_c = 1.8a_0$ the state [Kr]$4d^8 4f^7$ flips below the initial ground state. The ionization energy of the unconfined atom is $\Delta E_0 = 9.35$ eV. The studied configurations, atomic radii and excitation energies in the unconfined calculations are as follows:

configuration	r_ϵ	r_ρ	$r_{\max}$	ΔE
[Kr]$5s^2 4d^{10} 5p^3$	5.56	4.71	2.37	0.00
[Kr]$5s^1 4d^{10} 5p^4$	5.81	4.78	2.42	7.10
[Kr]$5s^2 4d^{10} 5p^2 5d^1$	13.25	4.68	5.29	7.41
[Kr]$5s^2 4d^{10} 5p^2 4f^1$	25.73	4.33	16.48	8.47
[Kr]$5s^2 4d^{10} 5p^1 4f^2$	18.63	4.13	8.95	21.09
[Kr]$5s^2 4d^{10} 4f^3$	15.61	5.54	6.07	35.85
[Kr]$5s^1 4d^{10} 4f^4$	13.85	6.32	4.42	59.39
[Kr]$4d^{10} 4f^5$	12.67	6.46	3.68	85.24
[Kr]$4d^9 4f^6$	12.13	6.25	2.82	142.13
[Kr]$4d^8 4f^7$	11.83	6.09	2.11	200.83
[Kr]$5s^2 4d^{10} 5p^2 6s^1$	Not converged			

Te The energies of the low lying configurations of hard-wall confined Te are shown in fig. S216 for the neutral atom and in fig. S217 for the cation. The ground state of the unconfined Te is [Kr]$5s^2 4d^{10} 5p^4$. At $r_c = 2.1a_0$ the ground state changes to [Kr]$5s^2 4d^{10} 4f^4$. Furthermore, at $r_c = 1.9a_0$ we see a ground state crossing to [Kr]$5s^1 4d^{10} 4f^5$. At $r_c = 1.8a_0$ we see a ground state crossing to [Kr]$4d^{10} 4f^6$.

At $r_c = 2.1a_0$ the state [Kr]$5s^2 4d^{10} 5p^1 4f^3$ flips below the initial ground state. At $r_c = 2.1a_0$ the state [Kr]$5s^2 4d^{10} 5p^3 4f^1$ flips below the initial ground state. At $r_c = 2.1a_0$ the state [Kr]$5s^2 4d^{10} 5p^2 4f^2$ flips below the initial ground state. At $r_c = 1.9a_0$ the state [Kr]$4d^9 4f^7$ flips below the initial ground state. At $r_c = 1.8a_0$ the state [Kr]$4d^8 4f^8$ flips below the initial ground state. At $r_c = 1.8a_0$ the state [Ar]$4s^2 3d^{10} 4p^5 4d^{10} 4f^7$ flips below the initial ground state. The ionization energy of the unconfined atom is $\Delta E_0 = 8.49$ eV. The studied configurations, atomic radii and excitation energies in the unconfined calculations are as follows:

configuration	r_ϵ	r_ρ	$r_{\max}$	ΔE
[Kr]$5s^2 4d^{10} 5p^4$	5.38	4.65	2.20	0.00
[Kr]$5s^2 4d^{10} 5p^3 6s^1$	9.96	4.80	5.70	5.20
[Kr]$5s^2 4d^{10} 5p^3 5d^1$	12.47	4.68	3.82	6.40
[Kr]$5s^2 4d^{10} 5p^3 4f^1$	25.76	4.28	16.52	7.60
[Kr]$5s^1 4d^{10} 5p^5$	5.48	4.71	2.22	8.09
[Kr]$5s^2 4d^{10} 5p^2 4f^2$	18.64	4.18	8.87	22.55
[Kr]$5s^2 4d^{10} 5p^1 4f^3$	15.68	5.47	5.69	39.89
[Kr]$5s^2 4d^{10} 4f^4$	14.03	6.17	4.22	57.77
[Kr]$5s^1 4d^{10} 4f^5$	12.96	6.25	3.26	83.74
[Kr]$4d^{10} 4f^6$	12.09	6.32	2.88	112.09
[Kr]$4d^9 4f^7$	11.80	6.09	2.17	175.58
[Kr]$4d^8 4f^8$	24.51	5.45	15.08	241.17
[Ar]$4s^2 3d^{10} 4p^5 4d^{10} 4f^7$	11.86	6.09	2.13	246.40

I The energies of the low lying configurations of hard-wall confined I are shown in fig. S218 for the neutral atom and in fig. S219 for the cation. The ground state of the unconfined I is [Kr]$5s^2 4d^{10} 5p^5$. At $r_c = 2.1a_0$ the ground state changes to [Kr]$5s^2 4d^{10} 5p^3 4f^2$. Furthermore, at $r_c = 2.0a_0$ we see a ground state crossing to [Kr]$5s^2 4d^{10} 4f^5$. At $r_c = 1.8a_0$ we see a ground state crossing to [Kr]$4d^{10} 4f^7$.

At $r_c = 2.1a_0$ the state [Kr]$5s^2 4d^{10} 5p^1 4f^4$ flips below the initial ground state. At $r_c = 2.1a_0$ the state [Kr]$5s^2 4d^{10} 5p^4 4f^1$ flips below the initial ground state. At $r_c = 2.0a_0$ the state [Kr]$5s^1 4d^{10} 4f^6$ flips below the initial ground state. At $r_c = 2.0a_0$ the state [Kr]$4d^{10} 5p^1 4f^6$ flips below the initial ground state. At $r_c = 1.9a_0$ the state [Kr]$4d^9 4f^8$ flips below the initial ground state. At $r_c = 1.8a_0$ the state [Kr]$4d^8 4f^9$ flips below the initial ground state. At $r_c = 1.8a_0$ the state [Ar]$4s^2 3d^{10} 4p^5 4d^{10} 4f^8$ flips below the initial ground state. The ionization energy of the unconfined atom is $\Delta E_0 = 10.70$ eV. The studied configurations, atomic radii and excitation energies in the unconfined calculations are as follows:

configuration	r_ϵ	r_ρ	r_{max}	ΔE
[Kr]$5s^2 4d^{10} 5p^5$	5.14	4.61	2.05	0.00
[Kr]$5s^2 4d^{10} 5p^4 6s^1$	9.57	4.85	5.42	7.26
[Kr]$5s^2 4d^{10} 5p^4 5d^1$	12.35	4.66	3.46	8.57
[Kr]$5s^1 4d^{10} 5p^6$	5.18	4.61	2.06	9.11
[Kr]$5s^2 4d^{10} 5p^4 4f^1$	25.78	4.27	16.61	9.82
[Kr]$5s^2 4d^{10} 5p^3 4f^2$	18.72	4.17	8.83	23.97
[Kr]$5s^2 4d^{10} 5p^1 4f^4$	14.52	5.87	3.35	63.15
[Kr]$5s^2 4d^{10} 4f^5$	13.36	6.08	2.84	82.71
[Kr]$5s^1 4d^{10} 4f^6$	12.55	6.08	2.35	109.92
[Kr]$4d^{10} 5p^1 4f^6$	12.58	6.01	2.29	120.36
[Kr]$4d^{10} 4f^7$	11.77	6.16	2.23	140.12
[Kr]$4d^9 4f^8$	24.45	5.45	14.99	210.79
[Kr]$4d^8 4f^9$	18.49	5.37	7.56	282.77
[Ar]$4s^2 3d^{10} 4p^5 4d^{10} 4f^8$	24.44	5.48	14.99	285.24

configuration	r_ϵ	r_ρ	r_{max}	ΔE
[Kr]$5s^2 4d^{10} 5p^6$	4.90	4.50	1.94	0.00
[Kr]$5s^2 4d^{10} 5p^5 6s^1$	9.30	4.81	5.21	9.14
[Kr]$5s^2 4d^{10} 5p^5 5d^1$	12.45	4.57	3.23	10.56
[Kr]$5s^2 4d^{10} 5p^5 4f^1$	25.80	4.17	16.65	11.81
[Kr]$5s^2 4d^{10} 5p^4 4f^2$	19.01	4.18	8.96	28.63
[Kr]$5s^2 4d^{10} 5p^2 4f^4$	15.37	5.41	1.86	67.03
[Kr]$5s^2 4d^{10} 5p^1 4f^5$	14.17	5.71	1.86	87.85
[Kr]$5s^2 4d^{10} 4f^6$	13.13	5.93	1.67	108.37
[Kr]$5s^1 4d^{10} 4f^7$	12.37	5.93	1.67	136.27
[Kr]$4d^{10} 5p^1 4f^7$	12.41	5.84	1.86	147.34
[Kr]$4d^{10} 4f^8$	24.40	5.48	14.91	169.39
[Kr]$4d^9 4f^9$	18.56	5.41	6.96	246.53
[Ar]$4s^2 3d^{10} 4p^5 4d^{10} 4f^9$	18.59	5.41	6.88	324.55

Xe The energies of the low lying configurations of hard-wall confined Xe are shown in fig. S220 for the neutral atom and in fig. S221 for the cation. The ground state of the unconfined Xe is [Kr]$5s^2 4d^{10} 5p^6$. At $r_c = 2.0a_0$ the ground state changes to [Kr]$5s^2 4d^{10} 4f^6$. Furthermore, at $r_c = 1.8a_0$ we see a ground state crossing to [Kr]$5s^1 4d^{10} 4f^7$. At $r_c = 1.7a_0$ we see a ground state crossing to [Kr]$4d^{10} 4f^8$.

At $r_c = 2.0a_0$ the state [Kr]$5s^2 4d^{10} 5p^1 4f^5$ flips below the initial ground state. At $r_c = 2.0a_0$ the state [Kr]$5s^2 4d^{10} 5p^2 4f^4$ flips below the initial ground state. At $r_c = 2.0a_0$ the state [Kr]$5s^2 4d^{10} 5p^5 4f^1$ flips below the initial ground state. At $r_c = 2.0a_0$ the state [Kr]$5s^2 4d^{10} 5p^4 4f^2$ flips below the initial ground state. At $r_c = 1.9a_0$ the state [Kr]$4d^{10} 5p^1 4f^7$ flips below the initial ground state. At $r_c = 1.8a_0$ the state [Kr]$4d^9 4f^9$ flips below the initial ground state. At $r_c = 1.8a_0$ the state [Ar]$4s^2 3d^{10} 4p^5 4d^{10} 4f^9$ flips below the initial ground state. The ionization energy of the unconfined atom is $\Delta E_0 = 12.69$ eV. The studied configurations, atomic radii and excitation energies in the unconfined calculations are as follows:

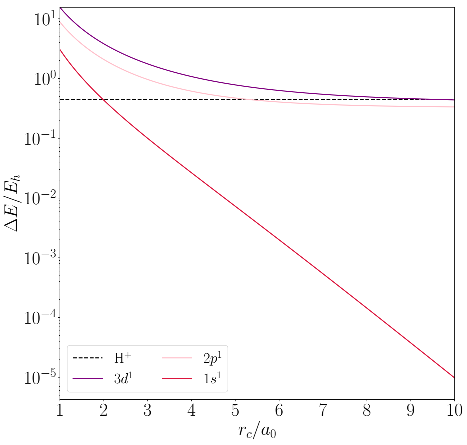

(a) PW92

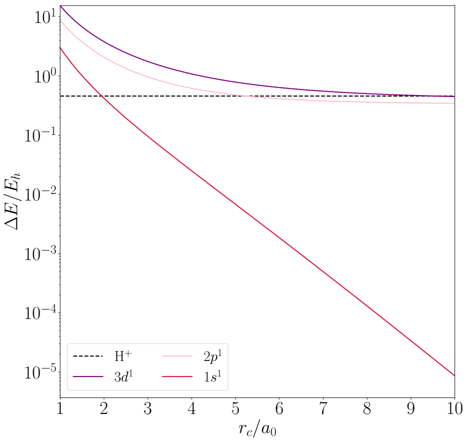

(b) PBE

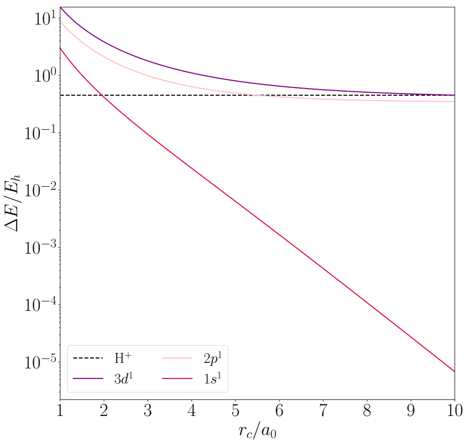

(c) r^{2}SCAN

Figure S8: Energies of various low lying configurations of hard-wall confined spin-restricted H shown as the energy difference from unconfined H as a function of the confinement radius $r_\infty = 1.0, 1.1, \ldots, 10.0a_0$. Note semilogarithmic scale.

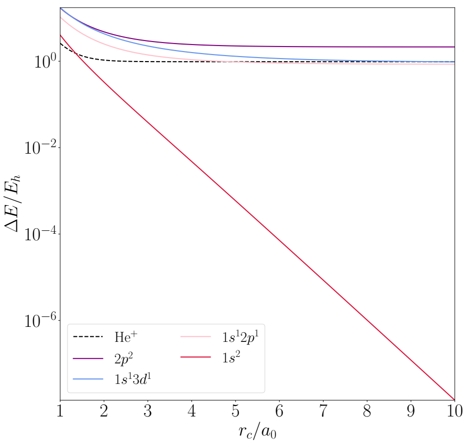

(a) PW92

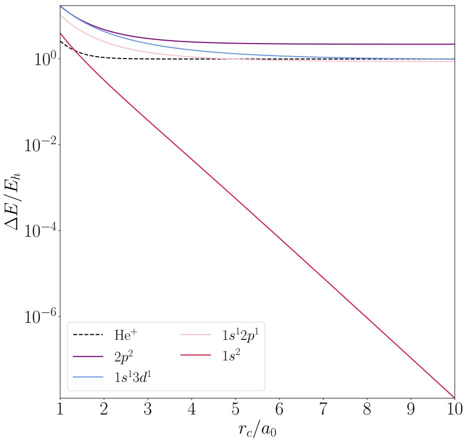

(b) PBE

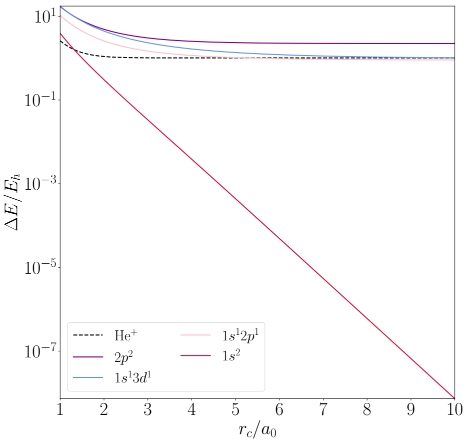

(c) r^{2}SCAN

Figure S9: Energies of various low lying configurations of hard-wall confined spin-restricted He shown as the energy difference from unconfined He as a function of the confinement radius $r_\infty = 1.0, 1.1, \ldots, 10.0a_0$. Note semilogarithmic scale.

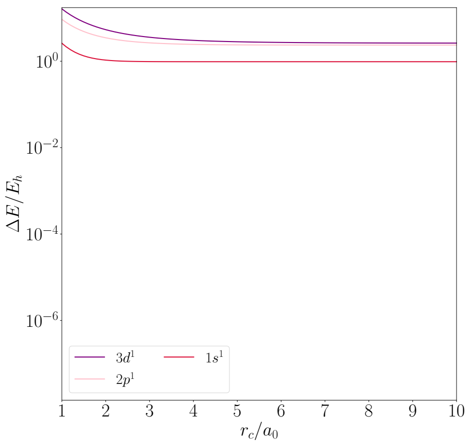

(a) PW92

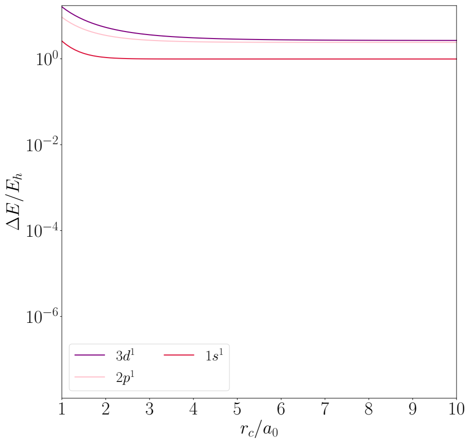

(b) PBE

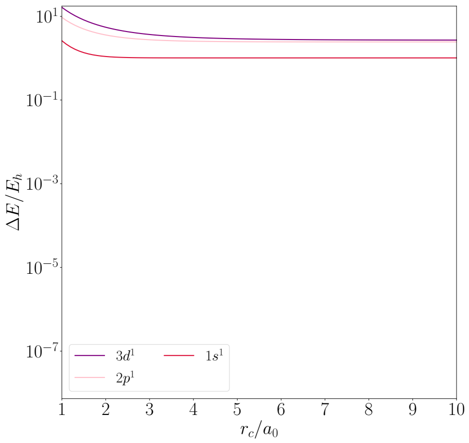

(c) r^{2}SCAN

Figure S10: Energies of various low lying configurations of the hard-wall confined spin-restricted monocation of He shown as the energy difference from unconfined He as a function of the confinement radius $r_\infty = 1.0, 1.1, \ldots, 10.0a_0$. Note semilogarithmic scale.

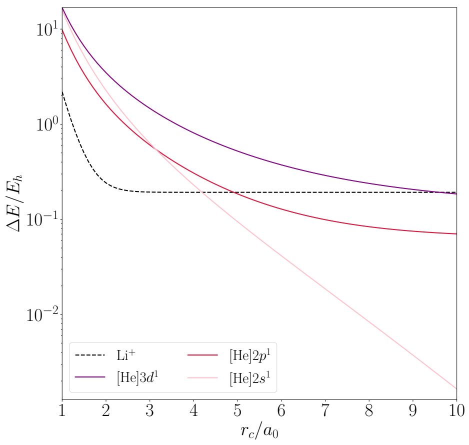

(a) PW92

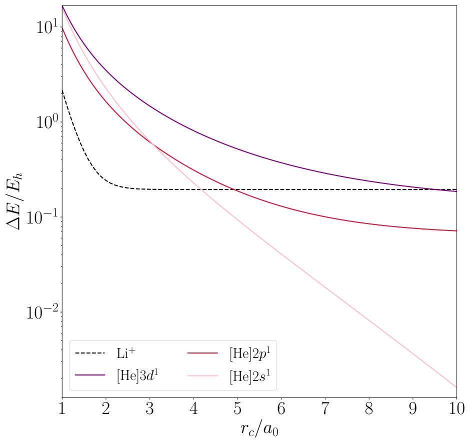

(b) PBE

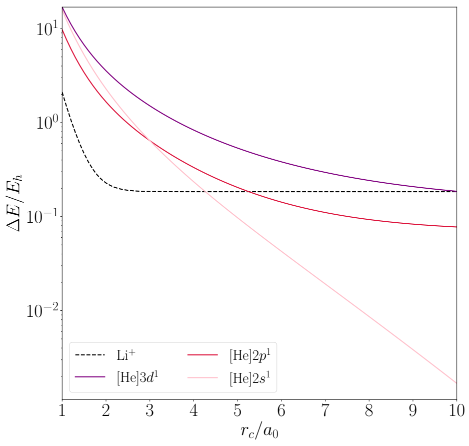

(c) r^{2}SCAN

Figure S11: Energies of various low lying configurations of hard-wall confined spin-restricted Li shown as the energy difference from unconfined Li as a function of the confinement radius $r_\infty = 1.0, 1.1, \ldots, 10.0a_0$. Note semilogarithmic scale.

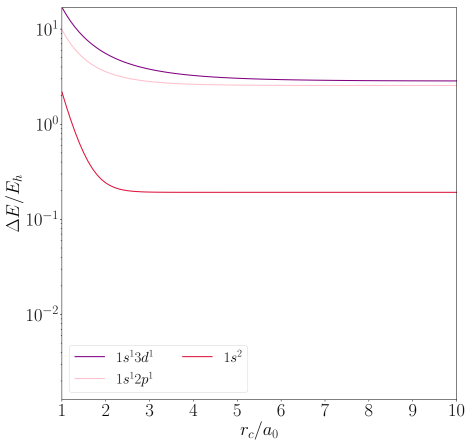

(a) PW92

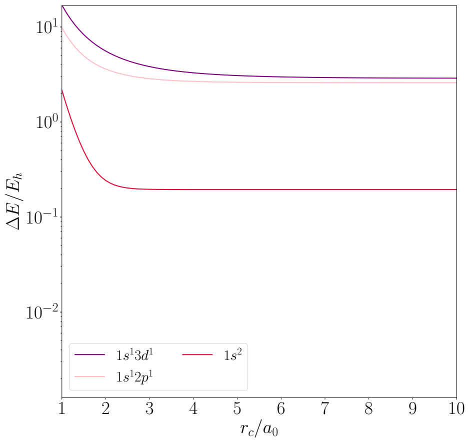

(b) PBE

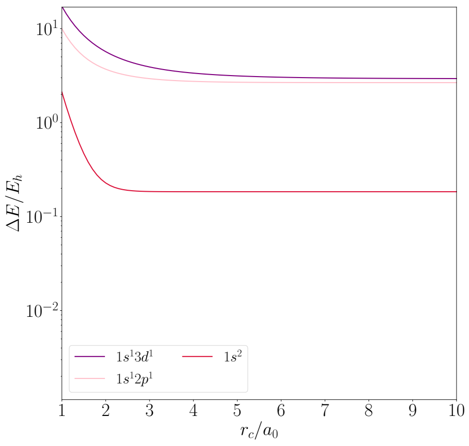

(c) r^{2}SCAN

Figure S12: Energies of various low lying configurations of the hard-wall confined spin-restricted monocation of Li shown as the energy difference from unconfined Li as a function of the confinement radius $r_\infty = 1.0, 1.1, \ldots, 10.0a_0$. Note semilogarithmic scale.

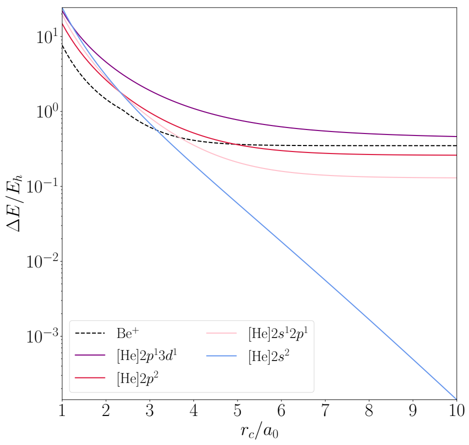

(a) PW92

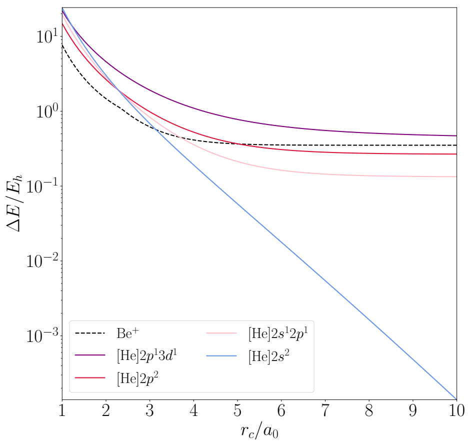

(b) PBE

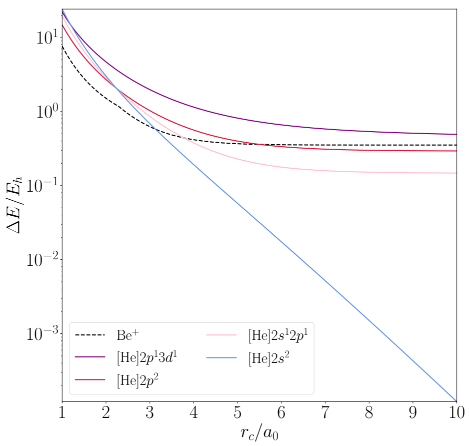

(c) r^{2}SCAN

Figure S13: Energies of various low lying configurations of hard-wall confined spin-restricted Be shown as the energy difference from unconfined Be as a function of the confinement radius $r_\infty = 1.0, 1.1, \ldots, 10.0 a_0$. Note semilogarithmic scale.

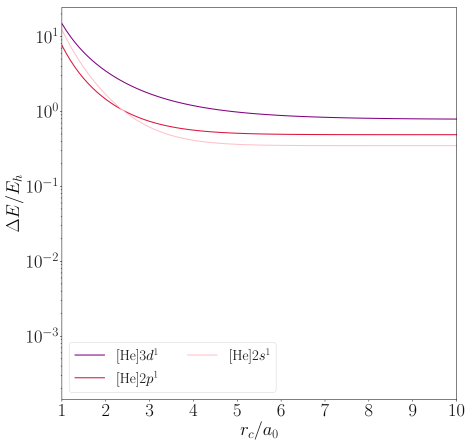

(a) PW92

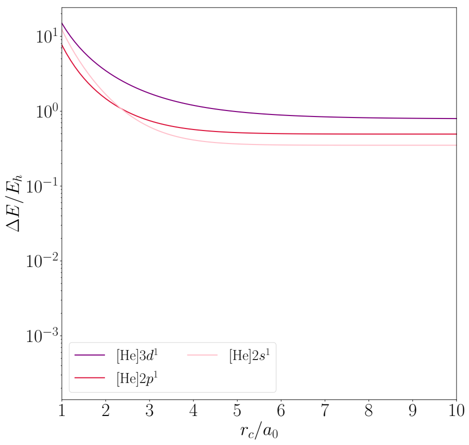

(b) PBE

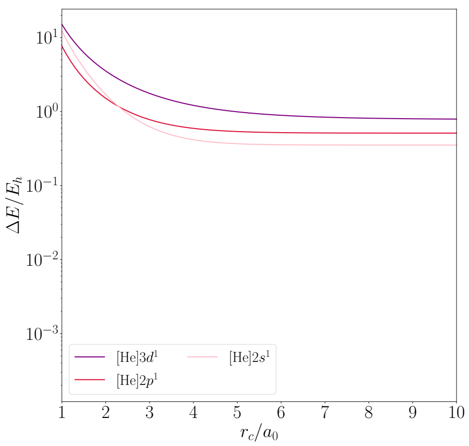

(c) r^{2}SCAN

Figure S14: Energies of various low lying configurations of the hard-wall confined spin-restricted monocation of Be shown as the energy difference from unconfined Be as a function of the confinement radius $r_\infty = 1.0, 1.1, \ldots, 10.0 a_0$. Note semilogarithmic scale.

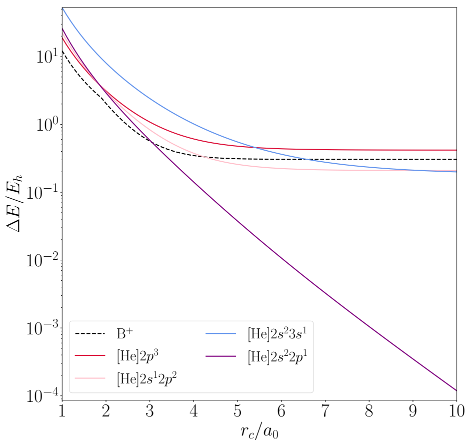

(a) PW92

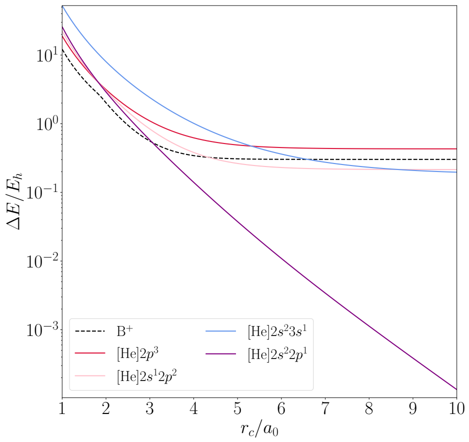

(b) PBE

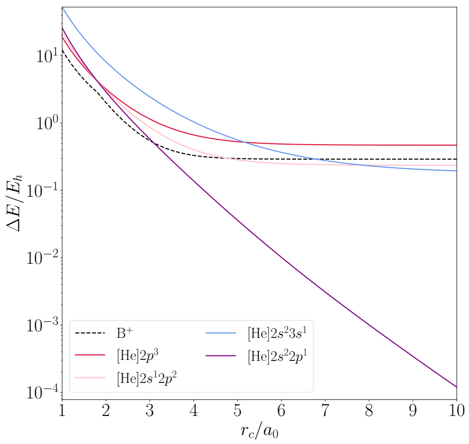

(c) r^{2}SCAN

Figure S15: Energies of various low lying configurations of hard-wall confined spin-restricted B shown as the energy difference from unconfined B as a function of the confinement radius $r_\infty = 1.0, 1.1, \ldots, 10.0a_0$. Note semilogarithmic scale.

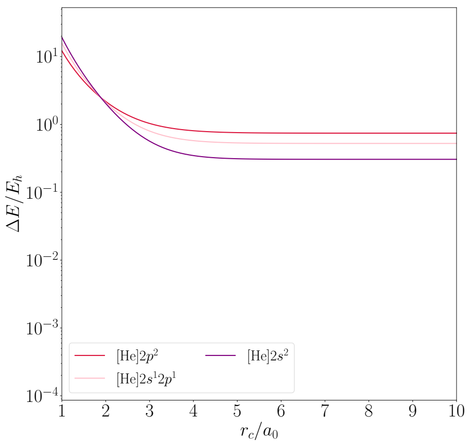

(a) PW92

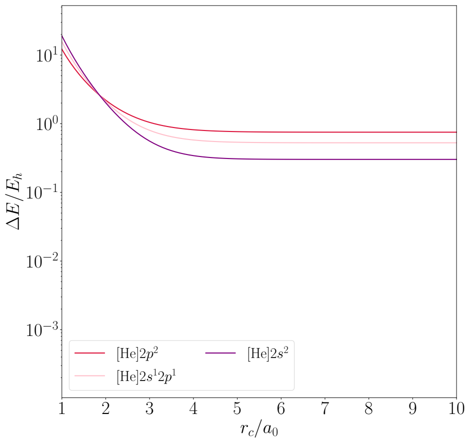

(b) PBE

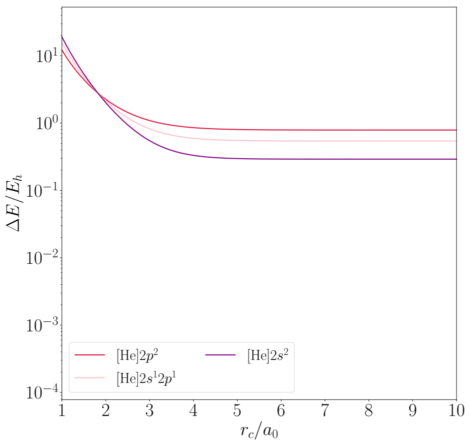

(c) r^{2}SCAN

Figure S16: Energies of various low lying configurations of the hard-wall confined spin-restricted monocation of B shown as the energy difference from unconfined B as a function of the confinement radius $r_\infty = 1.0, 1.1, \ldots, 10.0a_0$. Note semilogarithmic scale.

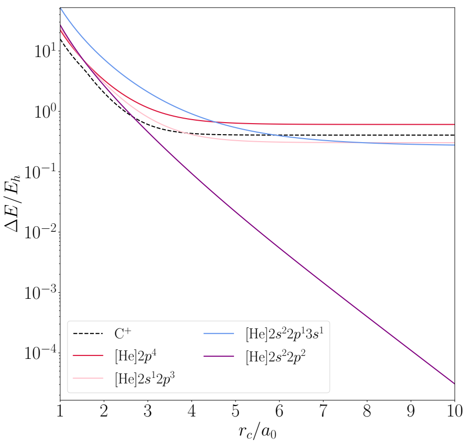

(a) PW92

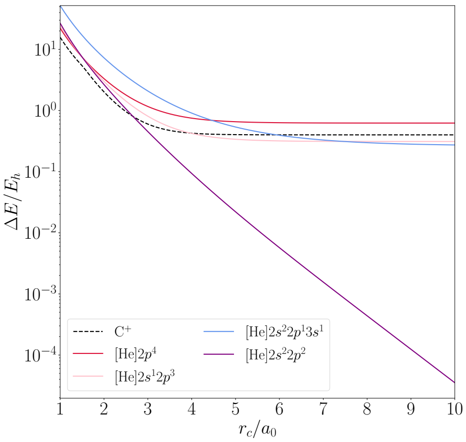

(b) PBE

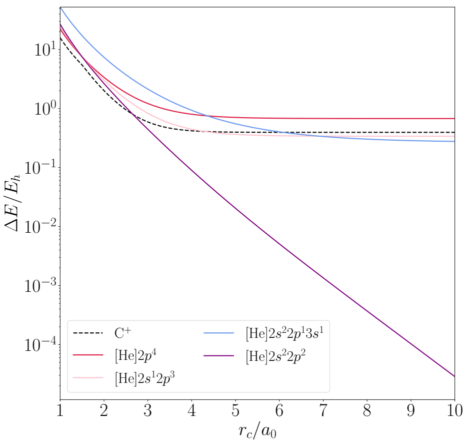

(c) r^{2}SCAN

Figure S17: Energies of various low lying configurations of hard-wall confined spin-restricted C shown as the energy difference from unconfined C as a function of the confinement radius $r_\infty = 1.0, 1.1, \ldots, 10.0a_0$. Note semilogarithmic scale.

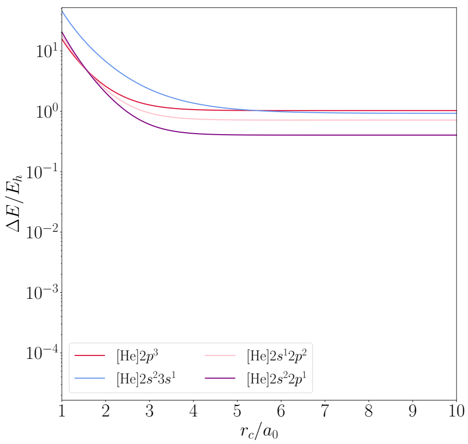

(a) PW92

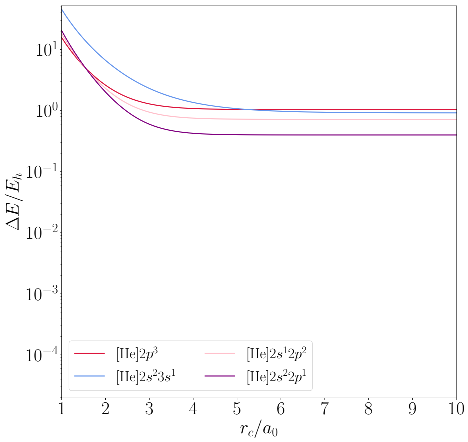

(b) PBE

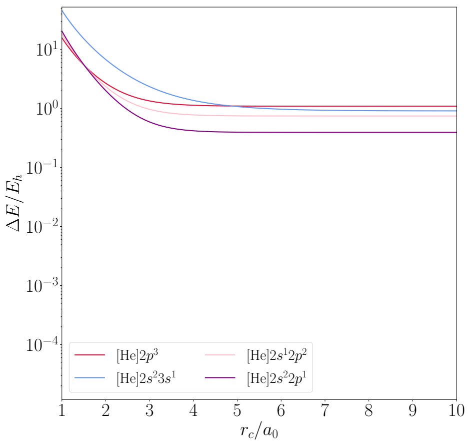

(c) r^{2}SCAN

Figure S18: Energies of various low lying configurations of the hard-wall confined spin-restricted monocation of C shown as the energy difference from unconfined C as a function of the confinement radius $r_\infty = 1.0, 1.1, \ldots, 10.0a_0$. Note semilogarithmic scale.

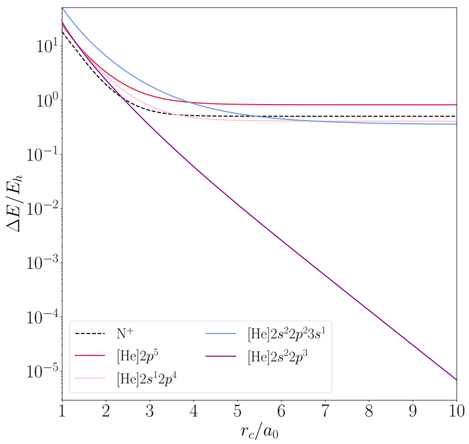

(a) PW92

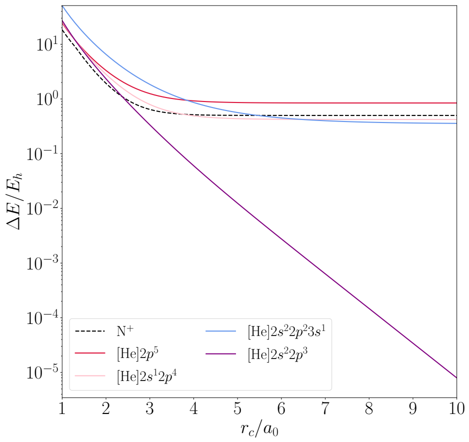

(b) PBE

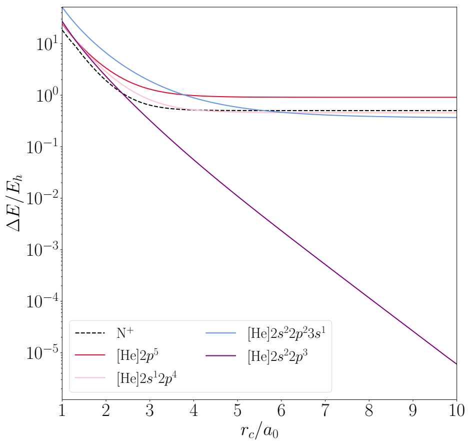

(c) r^{2}SCAN

Figure S19: Energies of various low lying configurations of hard-wall confined spin-restricted N shown as the energy difference from unconfined N as a function of the confinement radius $r_\infty = 1.0, 1.1, \ldots, 10.0a_0$. Note semilogarithmic scale.

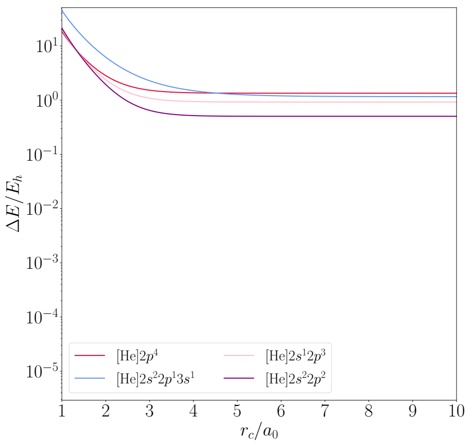

(a) PW92

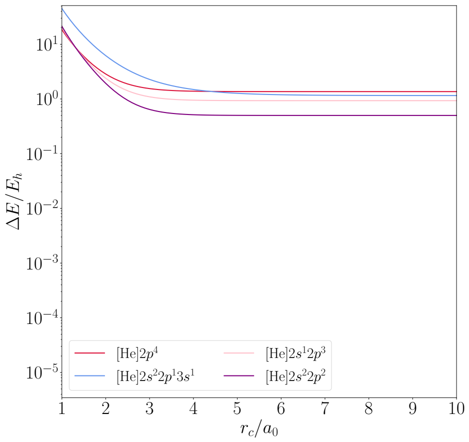

(b) PBE

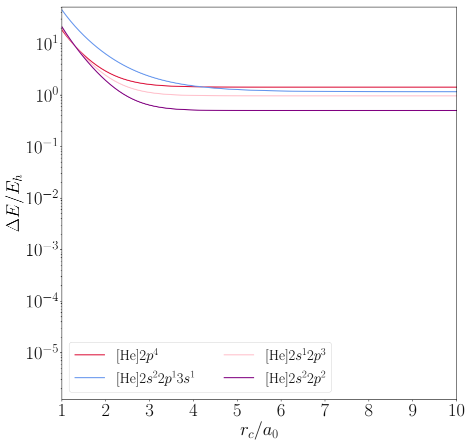

(c) r^{2}SCAN

Figure S20: Energies of various low lying configurations of the hard-wall confined spin-restricted monocation of N shown as the energy difference from unconfined N as a function of the confinement radius $r_\infty = 1.0, 1.1, \ldots, 10.0a_0$. Note semilogarithmic scale.

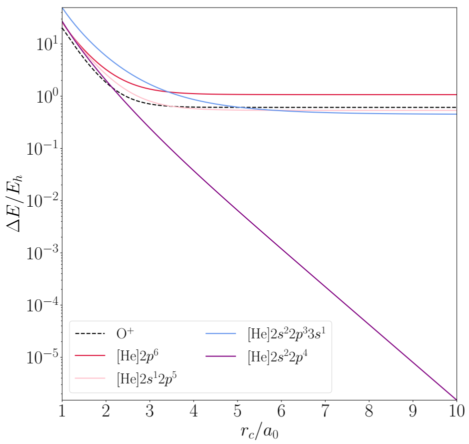

(a) PW92

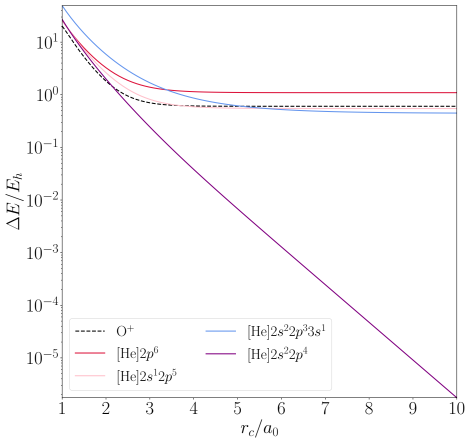

(b) PBE

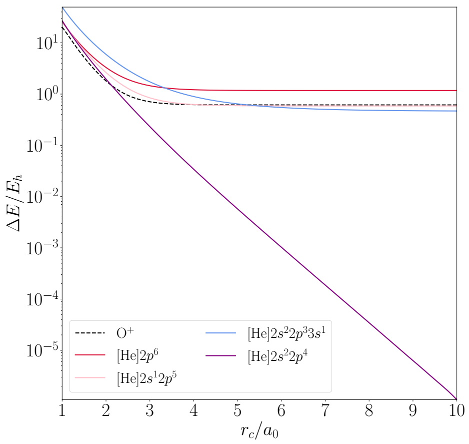

(c) r^{2}SCAN

Figure S21: Energies of various low lying configurations of hard-wall confined spin-restricted O shown as the energy difference from unconfined O as a function of the confinement radius $r_\infty = 1.0, 1.1, \ldots, 10.0a_0$. Note semilogarithmic scale.

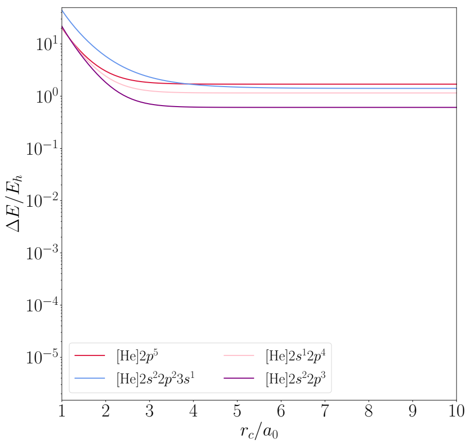

(a) PW92

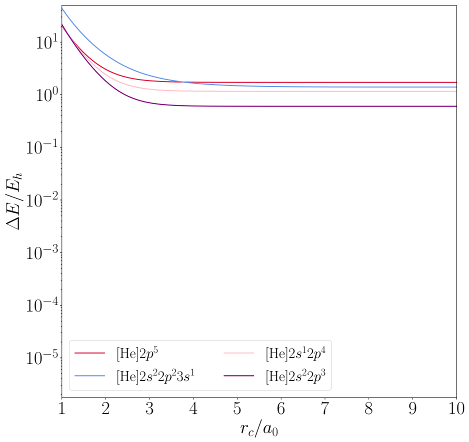

(b) PBE

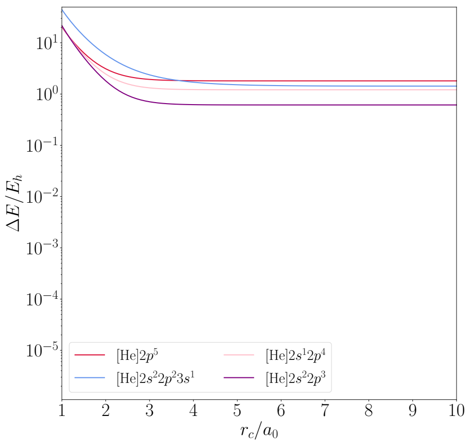

(c) r^{2}SCAN

Figure S22: Energies of various low lying configurations of the hard-wall confined spin-restricted monocation of O shown as the energy difference from unconfined O as a function of the confinement radius $r_\infty = 1.0, 1.1, \ldots, 10.0a_0$. Note semilogarithmic scale.

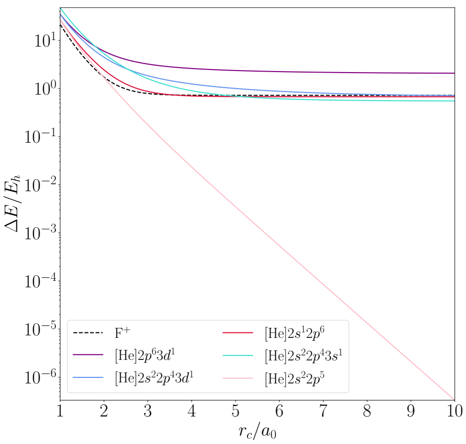

(a) PW92

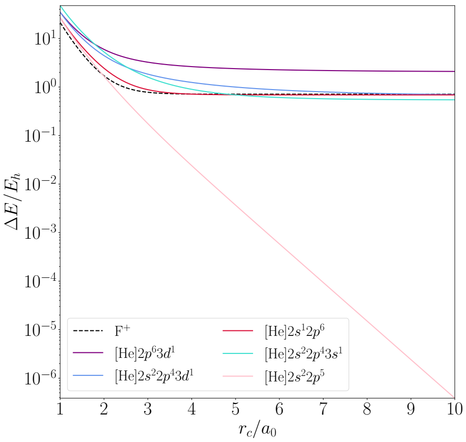

(b) PBE

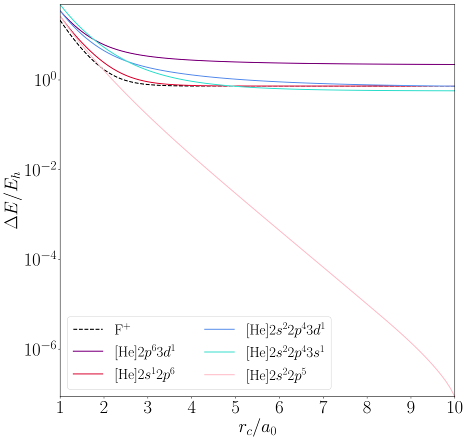

(c) r^{2}SCAN

Figure S23: Energies of various low lying configurations of hard-wall confined spin-restricted F shown as the energy difference from unconfined F as a function of the confinement radius $r_\infty = 1.0, 1.1, \ldots, 10.0 a_0$. Note semilogarithmic scale.

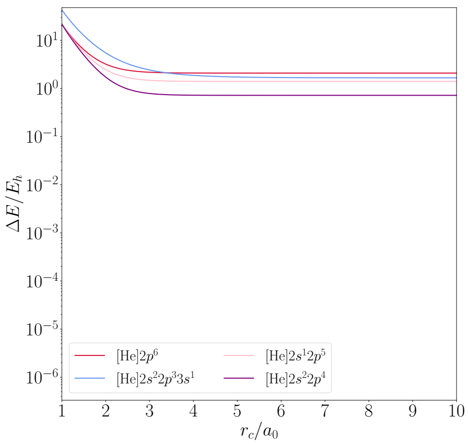

(a) PW92

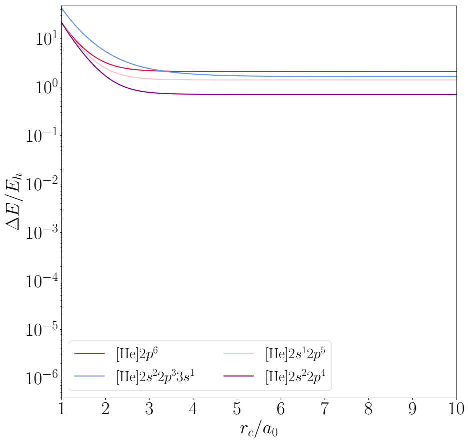

(b) PBE

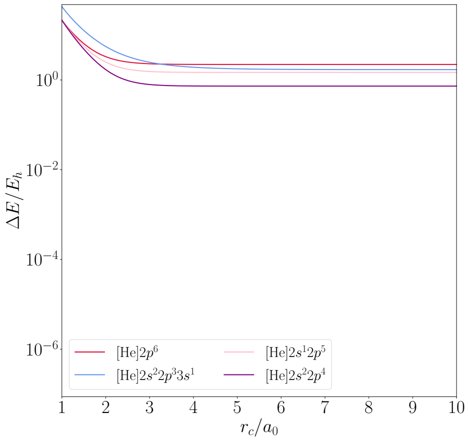

(c) r^{2}SCAN

Figure S24: Energies of various low lying configurations of the hard-wall confined spin-restricted monocation of F shown as the energy difference from unconfined F as a function of the confinement radius $r_\infty = 1.0, 1.1, \ldots, 10.0 a_0$. Note semilogarithmic scale.

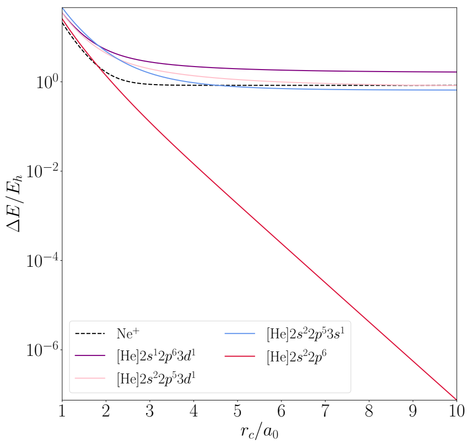

(a) PW92

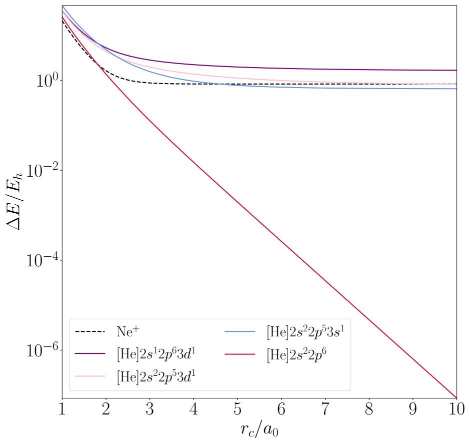

(b) PBE

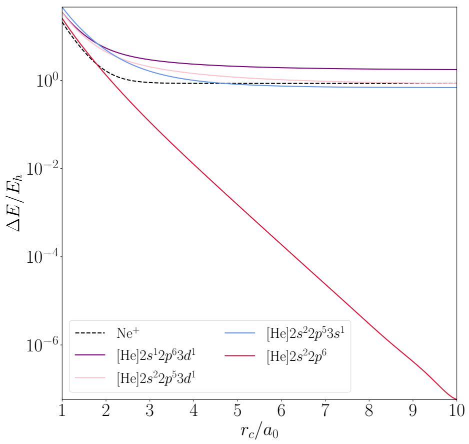

(c) r^{2}SCAN

Figure S25: Energies of various low lying configurations of hard-wall confined spin-restricted Ne shown as the energy difference from unconfined Ne as a function of the confinement radius $r_\infty = 1.0, 1.1, \ldots, 10.0 a_0$. Note semilogarithmic scale.

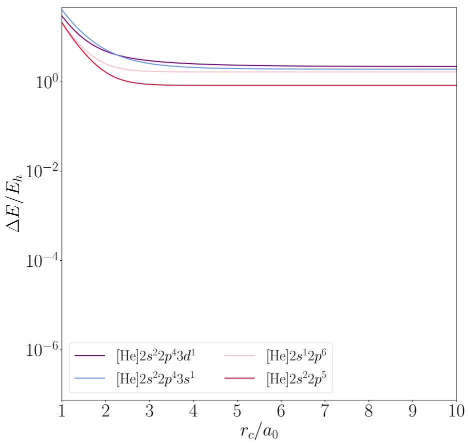

(a) PW92

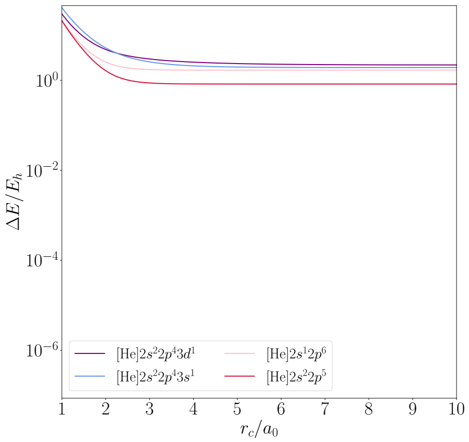

(b) PBE

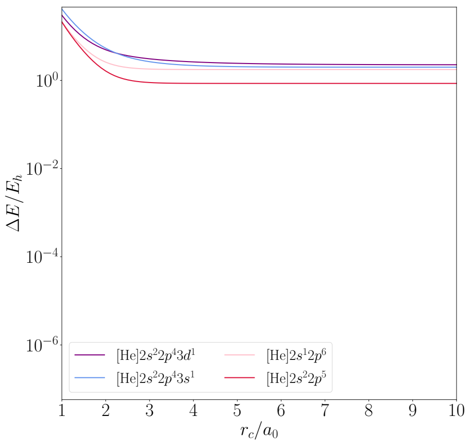

(c) r^{2}SCAN

Figure S26: Energies of various low lying configurations of the hard-wall confined spin-restricted monocation of Ne shown as the energy difference from unconfined Ne as a function of the confinement radius $r_\infty = 1.0, 1.1, \ldots, 10.0 a_0$. Note semilogarithmic scale.

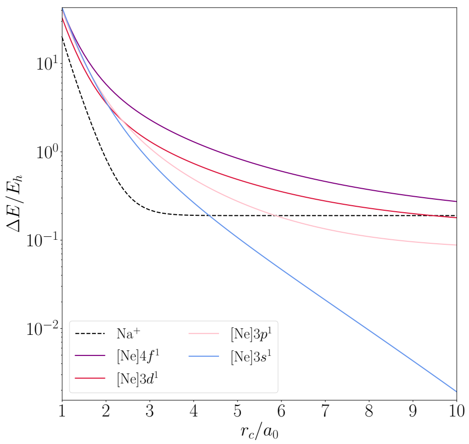

(a) PW92

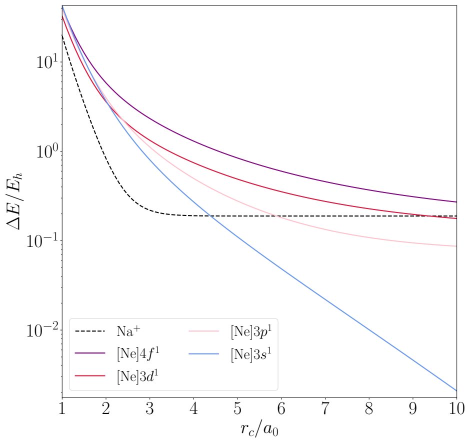

(b) PBE

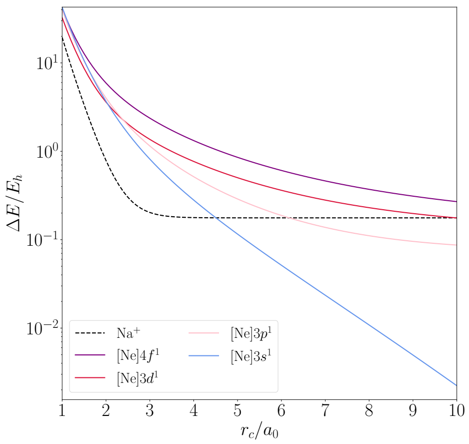

(c) r^{2}SCAN

Figure S27: Energies of various low lying configurations of hard-wall confined spin-restricted Na shown as the energy difference from unconfined Na as a function of the confinement radius $r_\infty = 1.0, 1.1, \ldots, 10.0a_0$. Note semilogarithmic scale.

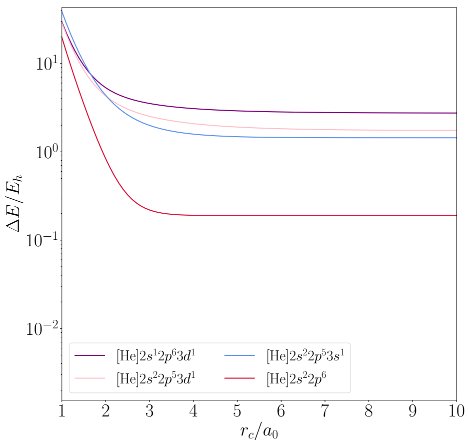

(a) PW92

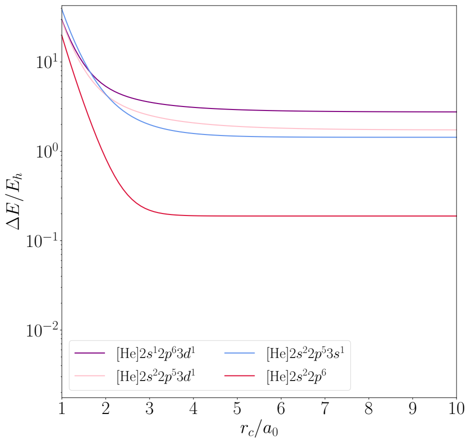

(b) PBE

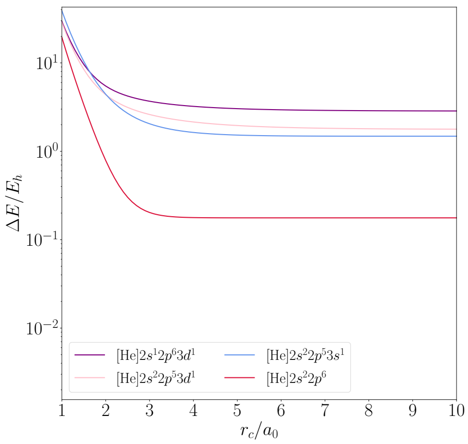

(c) r^{2}SCAN

Figure S28: Energies of various low lying configurations of the hard-wall confined spin-restricted monocation of Na shown as the energy difference from unconfined Na as a function of the confinement radius $r_\infty = 1.0, 1.1, \ldots, 10.0a_0$. Note semilogarithmic scale.

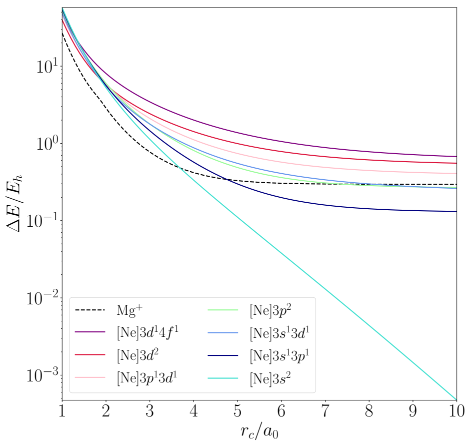

(a) PW92

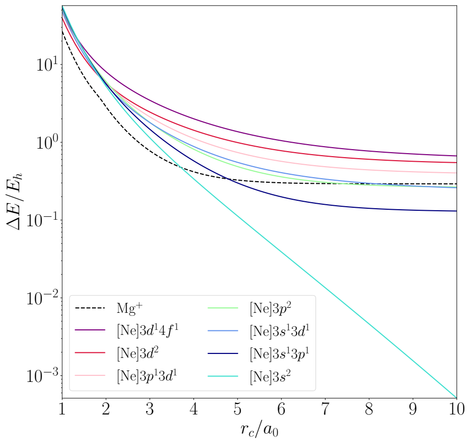

(b) PBE

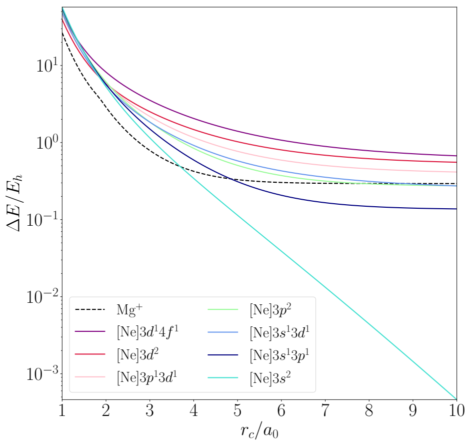

(c) r^{2}SCAN

Figure S29: Energies of various low lying configurations of hard-wall confined spin-restricted Mg shown as the energy difference from unconfined Mg as a function of the confinement radius $r_\infty = 1.0, 1.1, \ldots, 10.0a_0$. Note semilogarithmic scale.

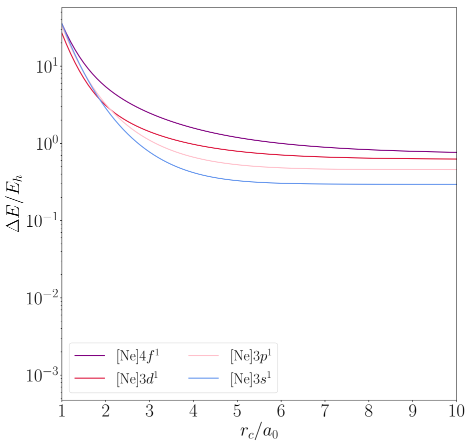

(a) PW92

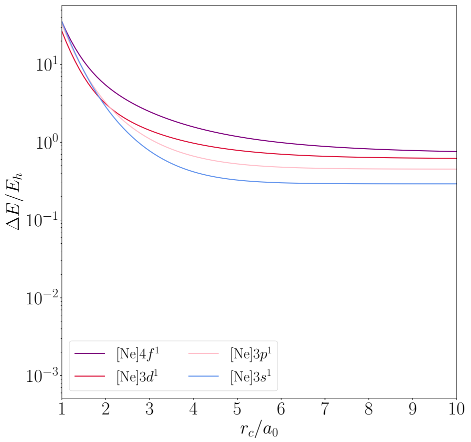

(b) PBE

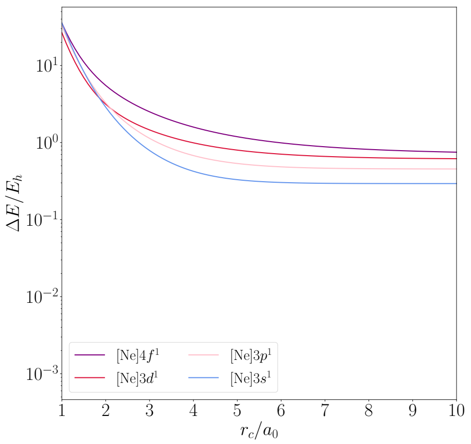

(c) r^{2}SCAN

Figure S30: Energies of various low lying configurations of the hard-wall confined spin-restricted monocation of Mg shown as the energy difference from unconfined Mg as a function of the confinement radius $r_\infty = 1.0, 1.1, \ldots, 10.0a_0$. Note semilogarithmic scale.

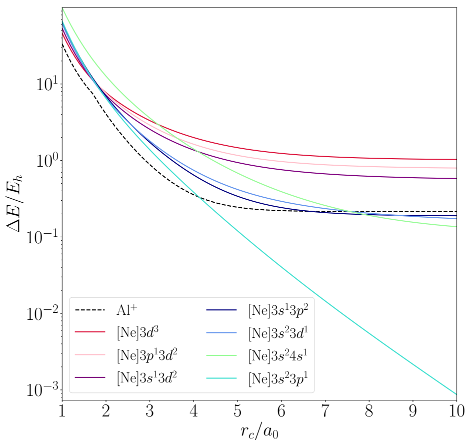

(a) PW92

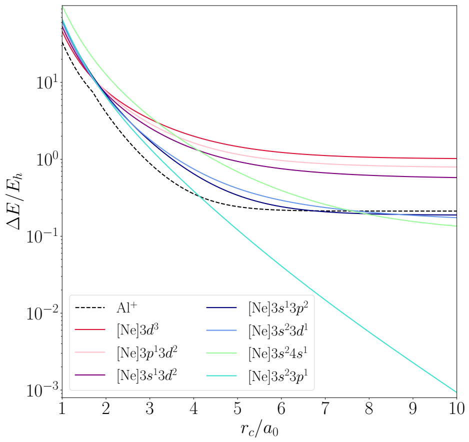

(b) PBE

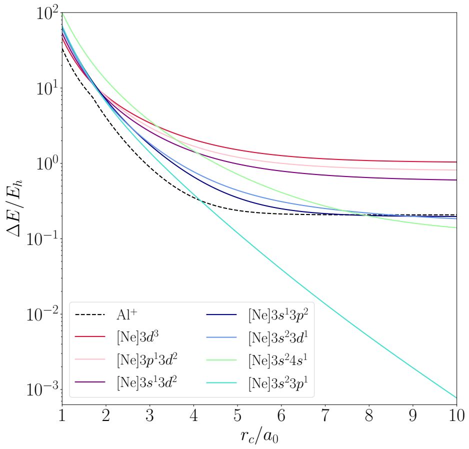

(c) r^{2}SCAN

Figure S31: Energies of various low lying configurations of hard-wall confined spin-restricted Al shown as the energy difference from unconfined Al as a function of the confinement radius $r_\infty = 1.0, 1.1, \ldots, 10.0 a_0$. Note semilogarithmic scale.

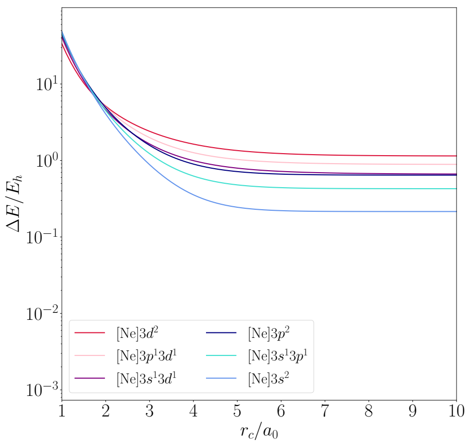

(a) PW92

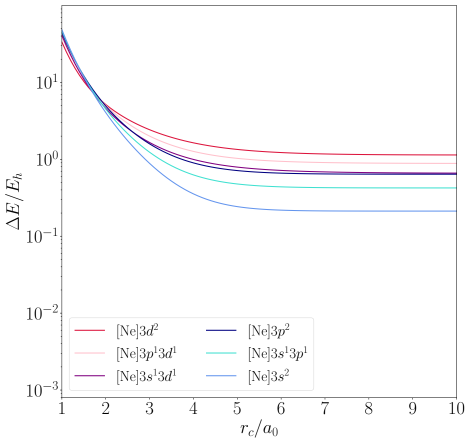

(b) PBE

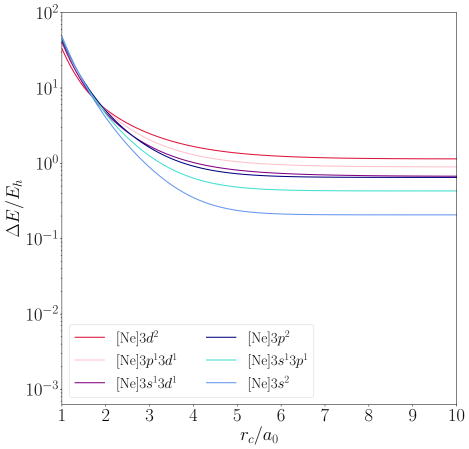

(c) r^{2}SCAN

Figure S32: Energies of various low lying configurations of the hard-wall confined spin-restricted monocation of Al shown as the energy difference from unconfined Al as a function of the confinement radius $r_\infty = 1.0, 1.1, \ldots, 10.0 a_0$. Note semilogarithmic scale.

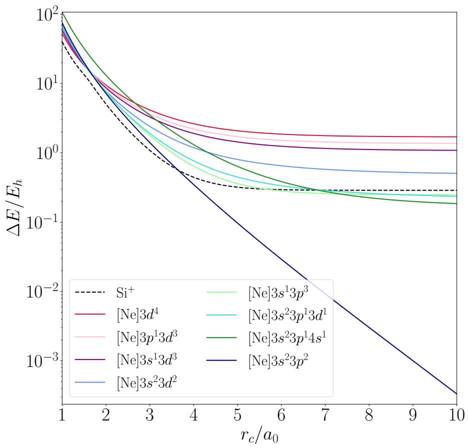

(a) PW92

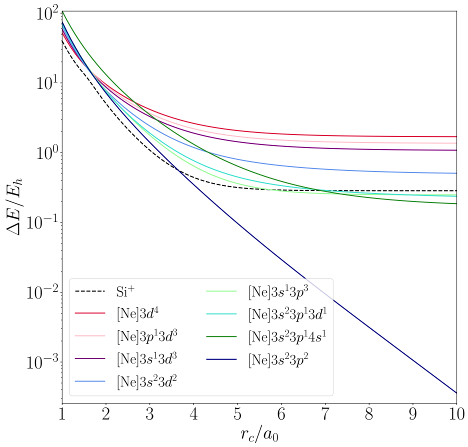

(b) PBE

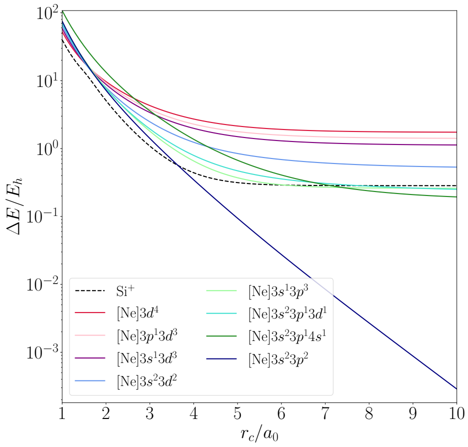

(c) r^{2}SCAN

Figure S33: Energies of various low lying configurations of hard-wall confined spin-restricted Si shown as the energy difference from unconfined Si as a function of the confinement radius $r_\infty = 1.0, 1.1, \ldots, 10.0a_0$. Note semilogarithmic scale.

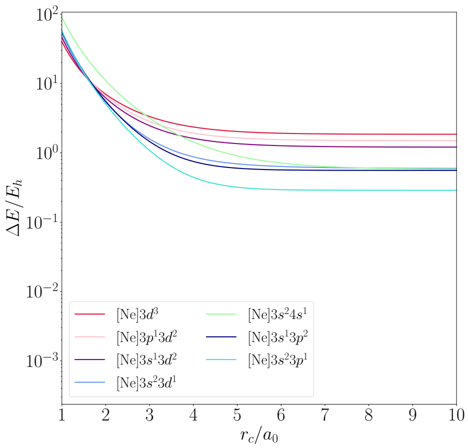

(a) PW92

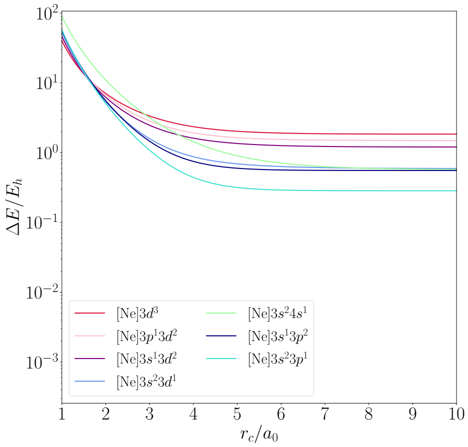

(b) PBE

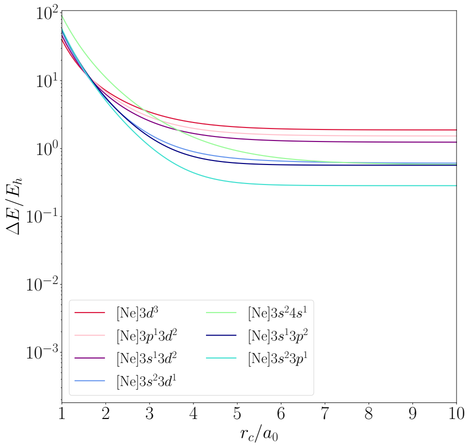

(c) r^{2}SCAN

Figure S34: Energies of various low lying configurations of the hard-wall confined spin-restricted monocation of Si shown as the energy difference from unconfined Si as a function of the confinement radius $r_\infty = 1.0, 1.1, \ldots, 10.0a_0$. Note semilogarithmic scale.

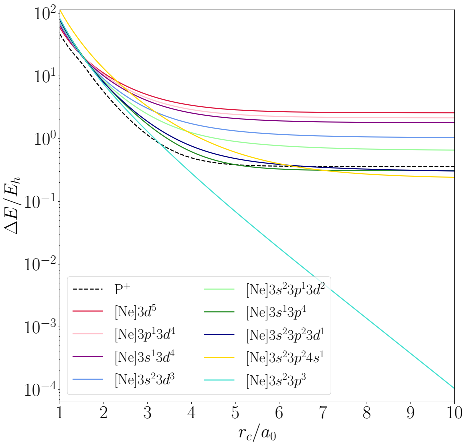

(a) PW92

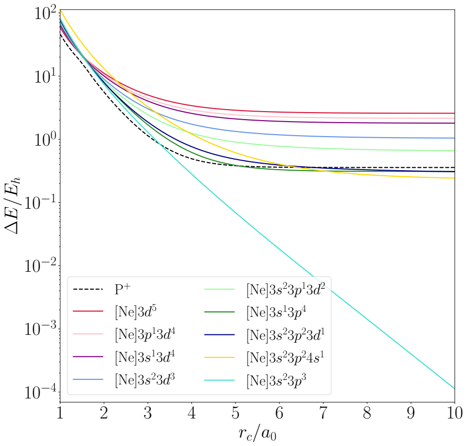

(b) PBE

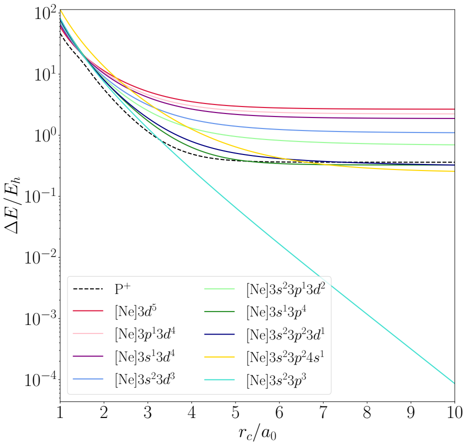

(c) r^{2}SCAN

Figure S35: Energies of various low lying configurations of hard-wall confined spin-restricted P shown as the energy difference from unconfined P as a function of the confinement radius $r_\infty = 1.0, 1.1, \ldots, 10.0 a_0$. Note semilogarithmic scale.

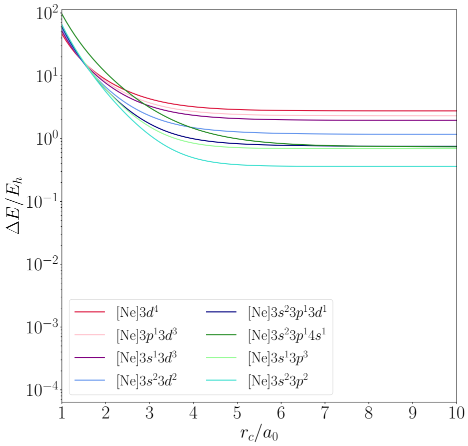

(a) PW92

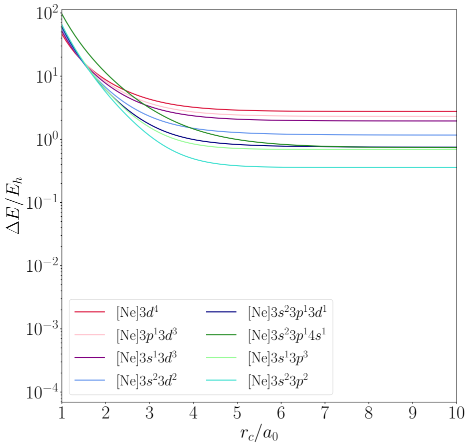

(b) PBE

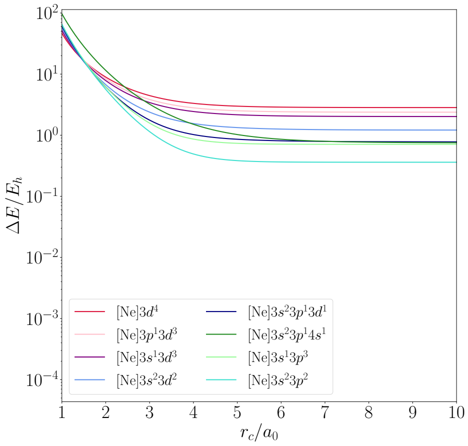

(c) r^{2}SCAN

Figure S36: Energies of various low lying configurations of the hard-wall confined spin-restricted monocation of P shown as the energy difference from unconfined P as a function of the confinement radius $r_\infty = 1.0, 1.1, \ldots, 10.0 a_0$. Note semilogarithmic scale.

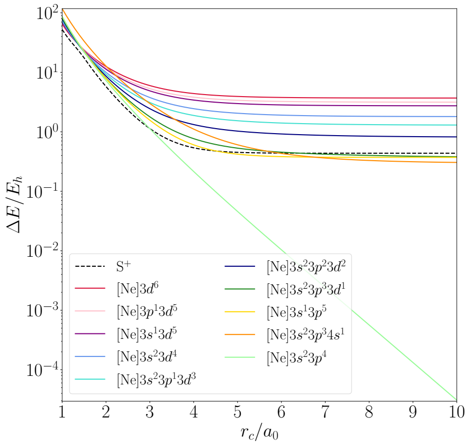

(a) PW92

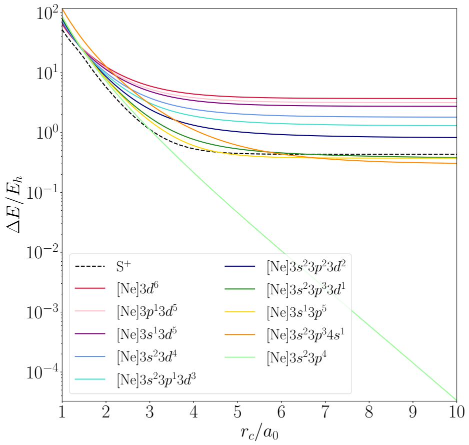

(b) PBE

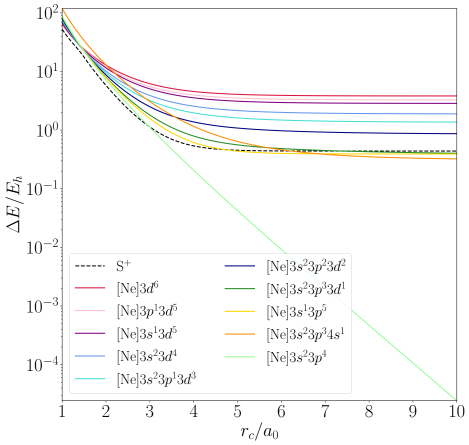

(c) r^{2}SCAN

Figure S37: Energies of various low lying configurations of hard-wall confined spin-restricted S shown as the energy difference from unconfined S as a function of the confinement radius $r_\infty = 1.0, 1.1, \ldots, 10.0 a_0$. Note semilogarithmic scale.

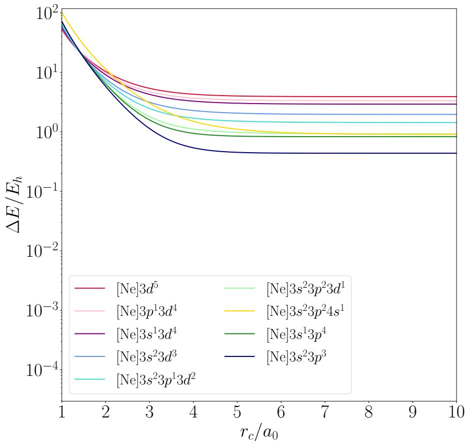

(a) PW92

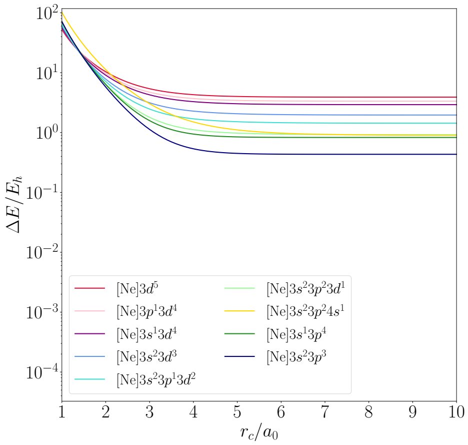

(b) PBE

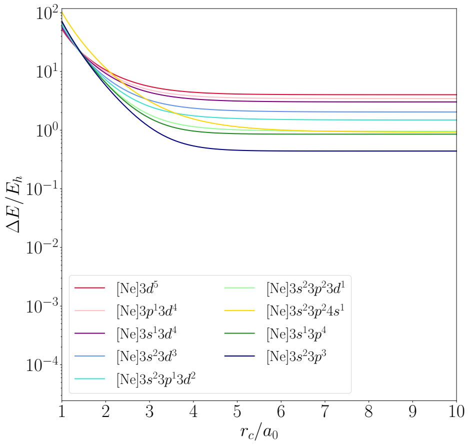

(c) r^{2}SCAN

Figure S38: Energies of various low lying configurations of the hard-wall confined spin-restricted monocation of S shown as the energy difference from unconfined S as a function of the confinement radius $r_\infty = 1.0, 1.1, \ldots, 10.0 a_0$. Note semilogarithmic scale.

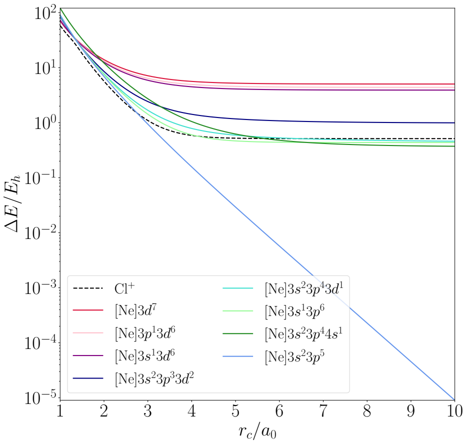

(a) PW92

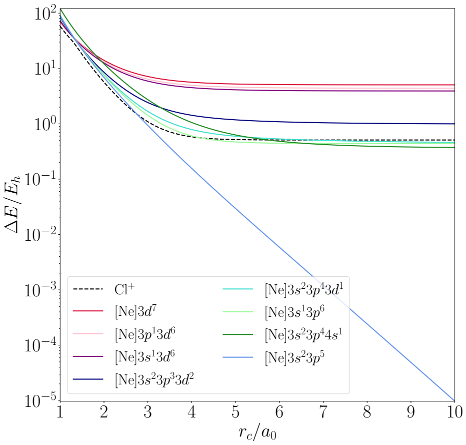

(b) PBE

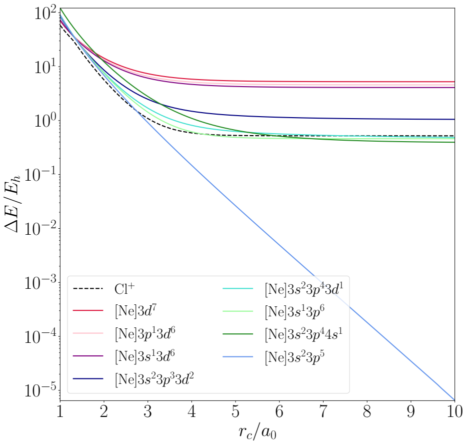

(c) r^{2}SCAN

Figure S39: Energies of various low lying configurations of hard-wall confined spin-restricted Cl shown as the energy difference from unconfined Cl as a function of the confinement radius $r_\infty = 1.0, 1.1, \ldots, 10.0 a_0$. Note semilogarithmic scale.

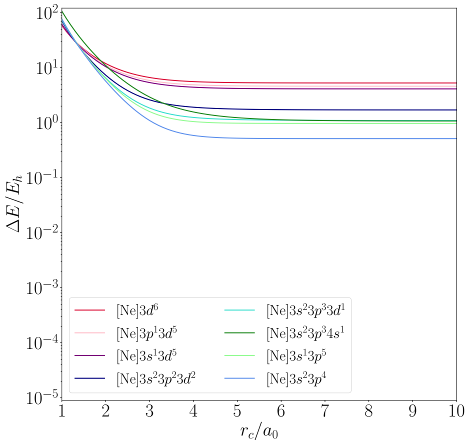

(a) PW92

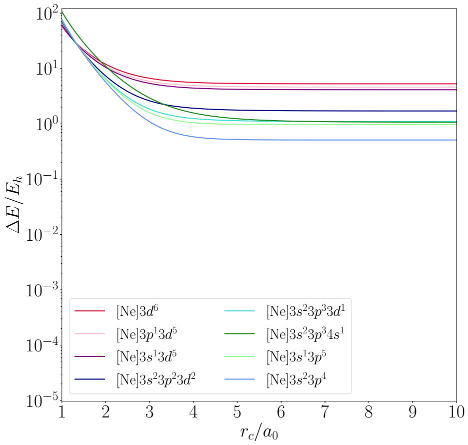

(b) PBE

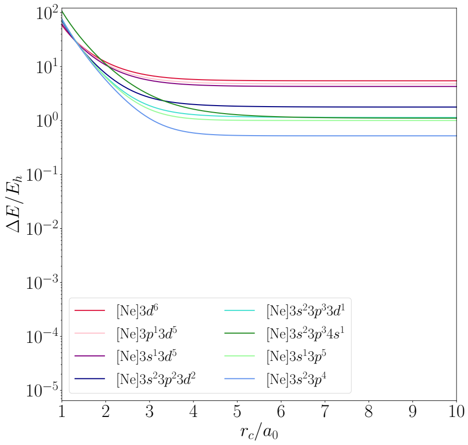

(c) r^{2}SCAN

Figure S40: Energies of various low lying configurations of the hard-wall confined spin-restricted monocation of Cl shown as the energy difference from unconfined Cl as a function of the confinement radius $r_\infty = 1.0, 1.1, \ldots, 10.0 a_0$. Note semilogarithmic scale.

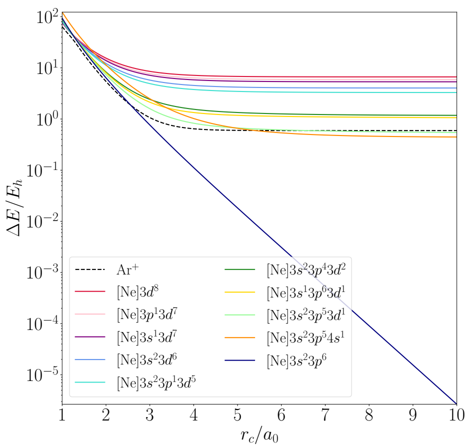

(a) PW92

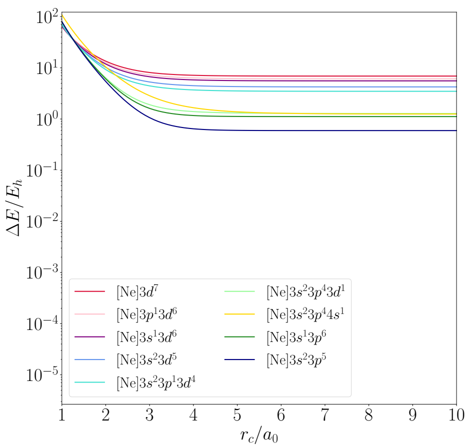

(a) PW92

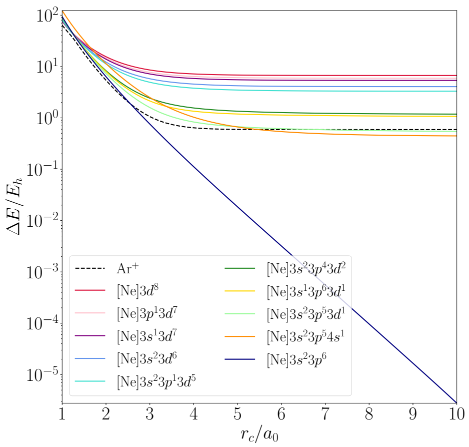

(b) PBE

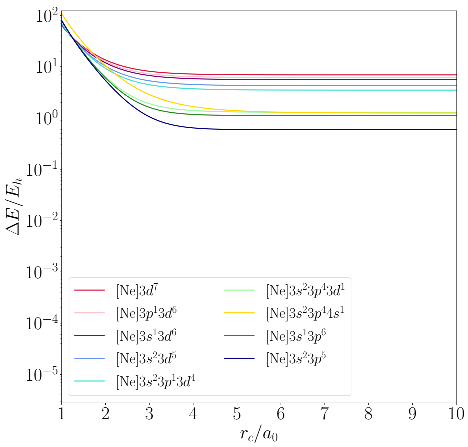

(b) PBE

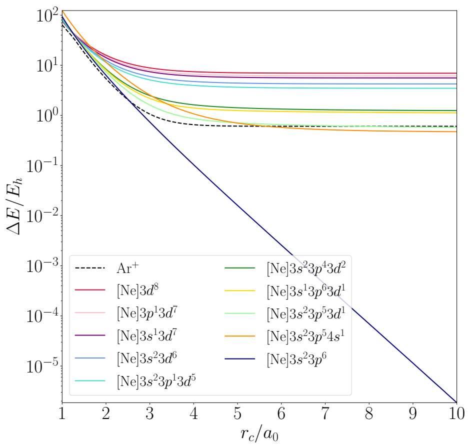

(c) r^{2}SCAN

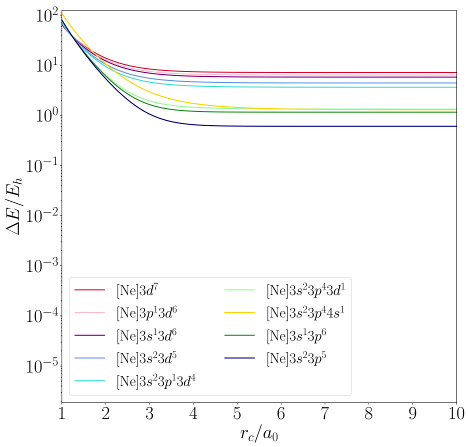

(c) r^{2}SCAN

Figure S41: Energies of various low lying configurations of hard-wall confined spin-restricted Ar shown as the energy difference from unconfined Ar as a function of the confinement radius $r_\infty = 1.0, 1.1, \ldots, 10.0 a_0$. Note semilogarithmic scale.

Figure S42: Energies of various low lying configurations of the hard-wall confined spin-restricted monocation of Ar shown as the energy difference from unconfined Ar as a function of the confinement radius $r_\infty = 1.0, 1.1, \ldots, 10.0 a_0$. Note semilogarithmic scale.

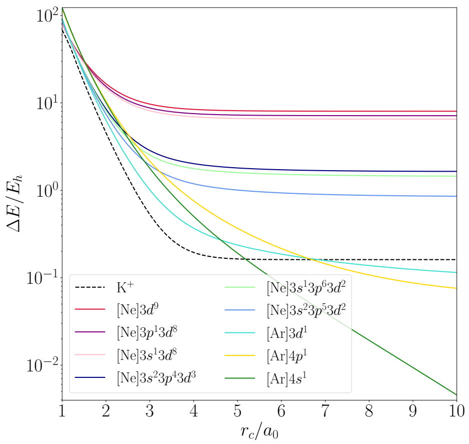

(a) PW92

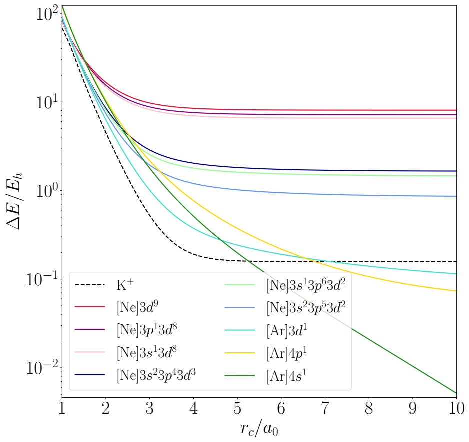

(b) PBE

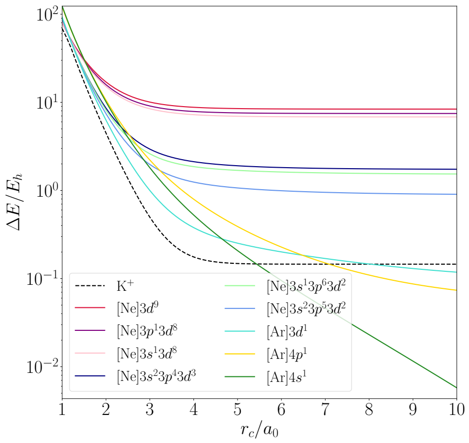

(c) r^{2}SCAN

Figure S43: Energies of various low lying configurations of hard-wall confined spin-restricted K shown as the energy difference from unconfined K as a function of the confinement radius $r_\infty = 1.0, 1.1, \ldots, 10.0a_0$. Note semilogarithmic scale.

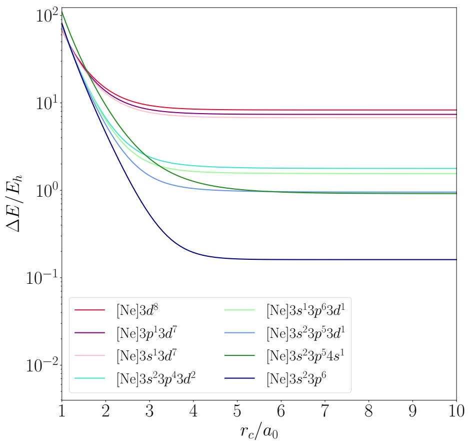

(a) PW92

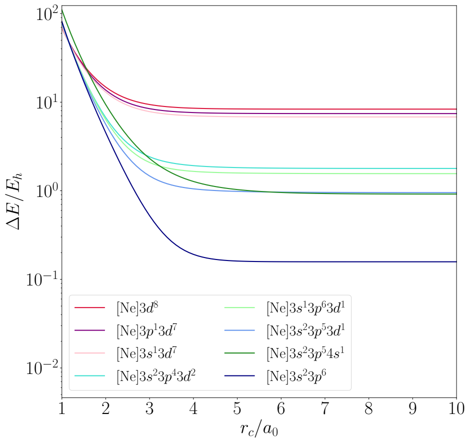

(b) PBE

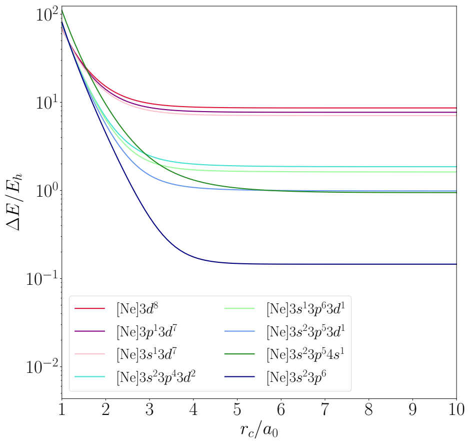

(c) r^{2}SCAN

Figure S44: Energies of various low lying configurations of the hard-wall confined spin-restricted monocation of K shown as the energy difference from unconfined K as a function of the confinement radius $r_\infty = 1.0, 1.1, \ldots, 10.0a_0$. Note semilogarithmic scale.

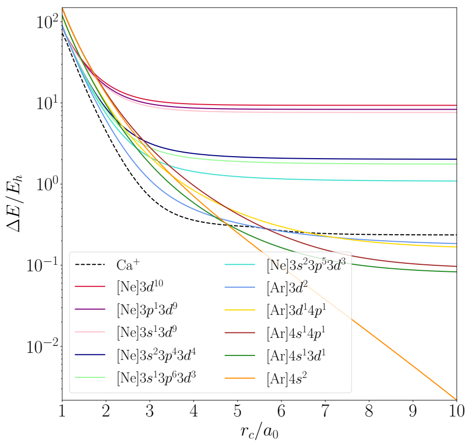

(a) PW92

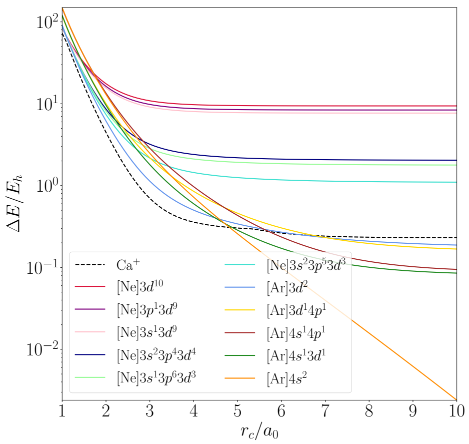

(b) PBE

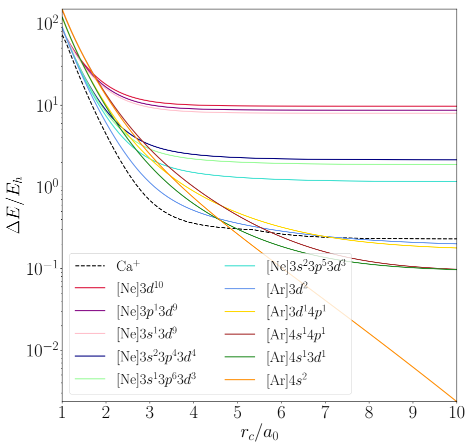

(c) r^{2}SCAN

Figure S45: Energies of various low lying configurations of hard-wall confined spin-restricted Ca shown as the energy difference from unconfined Ca as a function of the confinement radius $r_\infty = 1.0, 1.1, \ldots, 10.0a_0$. Note semilogarithmic scale.

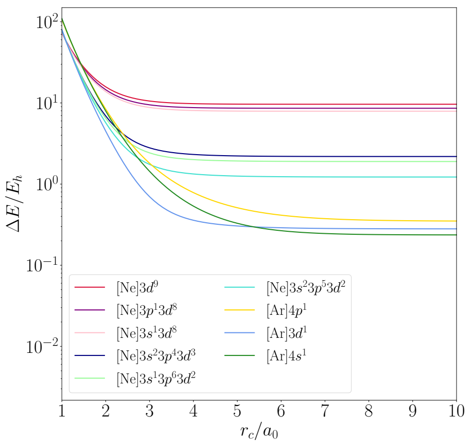

(a) PW92

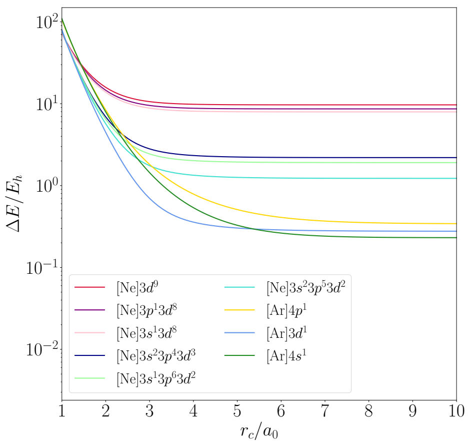

(b) PBE

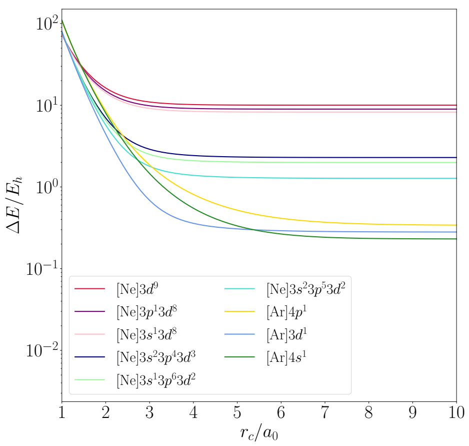

(c) r^{2}SCAN

Figure S46: Energies of various low lying configurations of the hard-wall confined spin-restricted monocation of Ca shown as the energy difference from unconfined Ca as a function of the confinement radius $r_\infty = 1.0, 1.1, \ldots, 10.0a_0$. Note semilogarithmic scale.

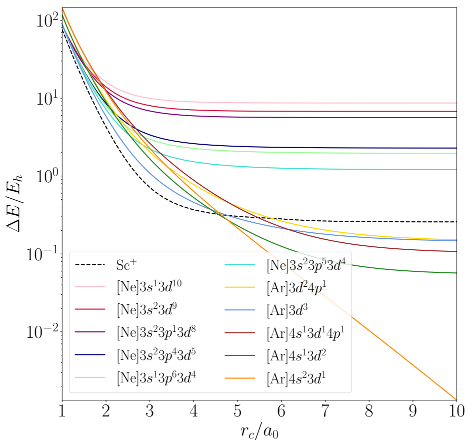

(a) PW92

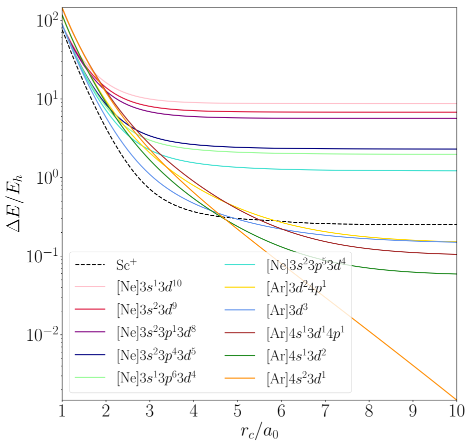

(b) PBE

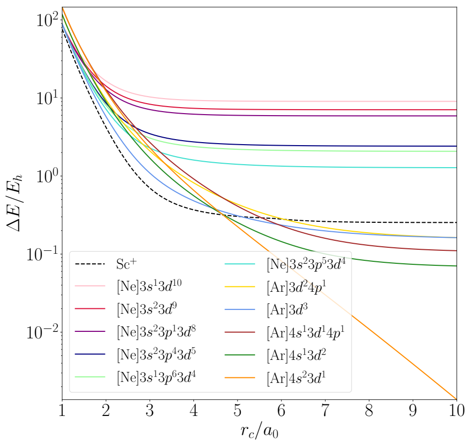

(c) r^{2}SCAN

Figure S47: Energies of various low lying configurations of hard-wall confined spin-restricted Sc shown as the energy difference from unconfined Sc as a function of the confinement radius $r_\infty = 1.0, 1.1, \ldots, 10.0 a_0$. Note semilogarithmic scale.

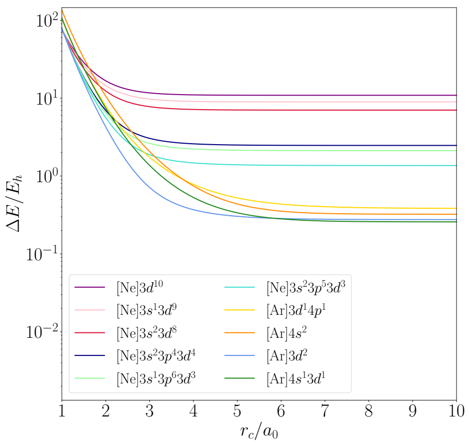

(a) PW92

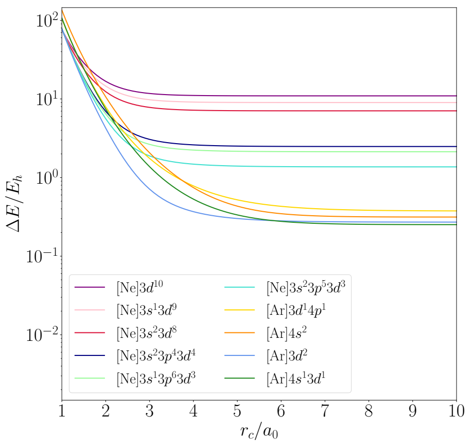

(b) PBE

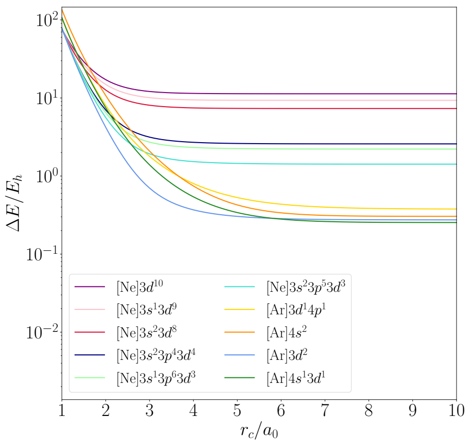

(c) r^{2}SCAN

Figure S48: Energies of various low lying configurations of the hard-wall confined spin-restricted monocation of Sc shown as the energy difference from unconfined Sc as a function of the confinement radius $r_\infty = 1.0, 1.1, \ldots, 10.0 a_0$. Note semilogarithmic scale.

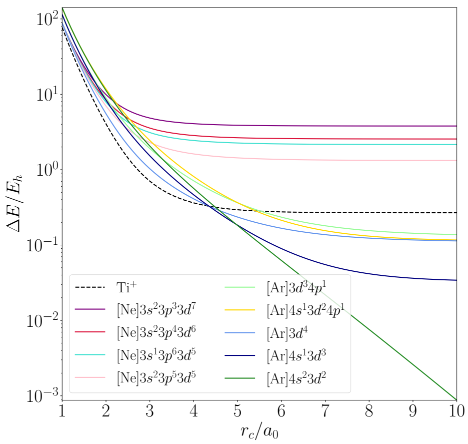

(a) PW92

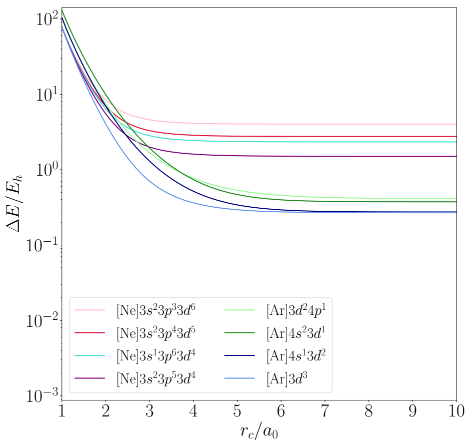

(a) PW92

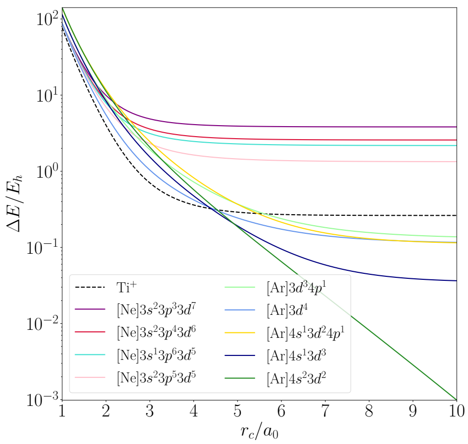

(b) PBE

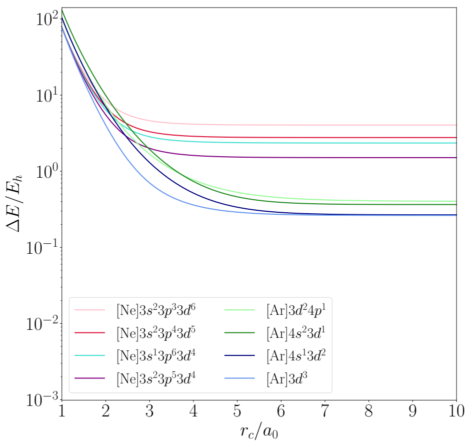

(b) PBE

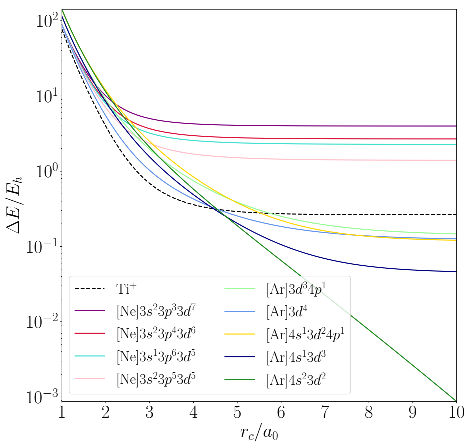

(c) r^{2}SCAN

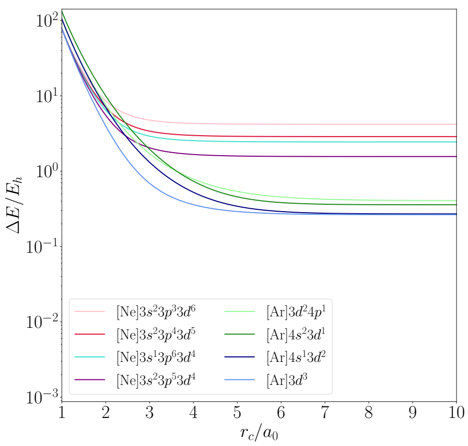

(c) r^{2}SCAN

Figure S49: Energies of various low lying configurations of hard-wall confined spin-restricted Ti shown as the energy difference from unconfined Ti as a function of the confinement radius $r_\infty = 1.0, 1.1, \ldots, 10.0a_0$. Note semilogarithmic scale.

Figure S50: Energies of various low lying configurations of the hard-wall confined spin-restricted monocation of Ti shown as the energy difference from unconfined Ti as a function of the confinement radius $r_\infty = 1.0, 1.1, \ldots, 10.0a_0$. Note semilogarithmic scale.

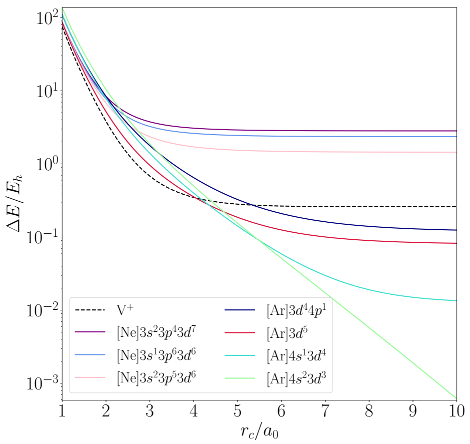

(a) PW92

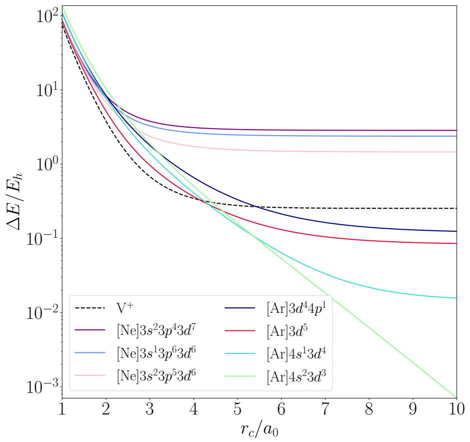

(b) PBE

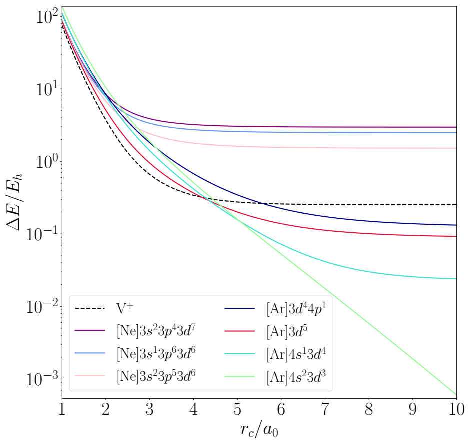

(c) r^{2}SCAN

Figure S51: Energies of various low lying configurations of hard-wall confined spin-restricted V shown as the energy difference from unconfined V as a function of the confinement radius $r_\infty = 1.0, 1.1, \ldots, 10.0 a_0$. Note semilogarithmic scale.

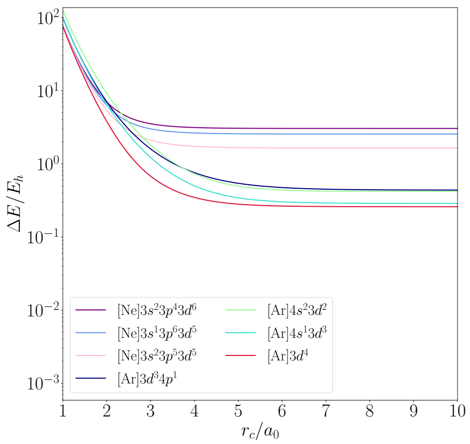

(a) PW92

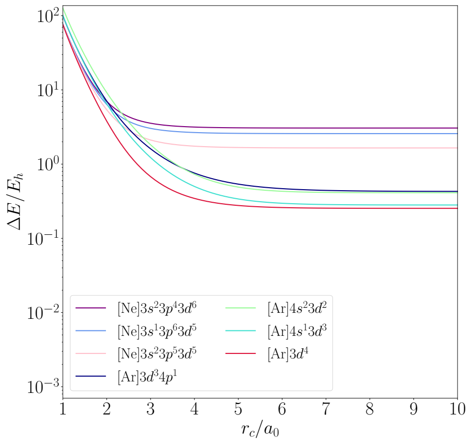

(b) PBE

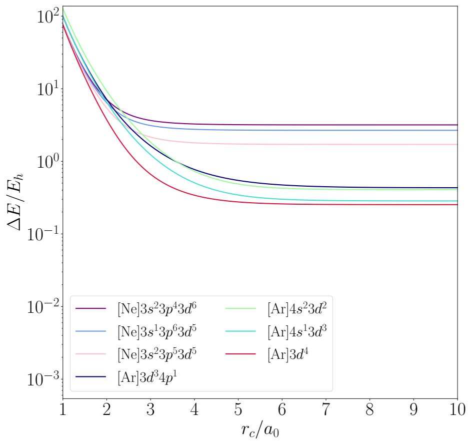

(c) r^{2}SCAN

Figure S52: Energies of various low lying configurations of the hard-wall confined spin-restricted monocation of V shown as the energy difference from unconfined V as a function of the confinement radius $r_\infty = 1.0, 1.1, \ldots, 10.0 a_0$. Note semilogarithmic scale.

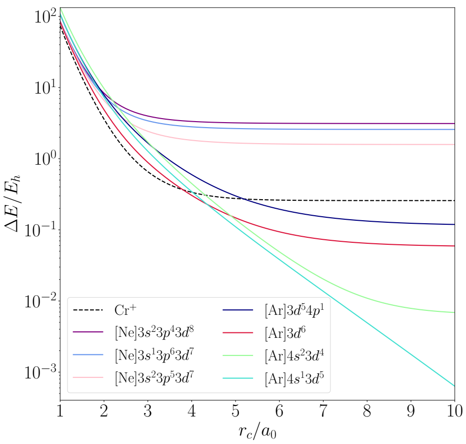

(a) PW92

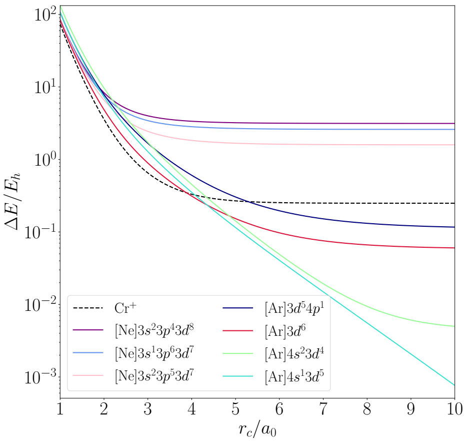

(b) PBE

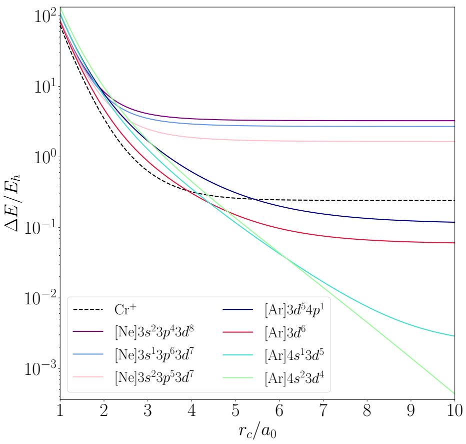

(c) r^{2}SCAN

Figure S53: Energies of various low lying configurations of hard-wall confined spin-restricted Cr shown as the energy difference from unconfined Cr as a function of the confinement radius $r_\infty = 1.0, 1.1, \ldots, 10.0 a_0$. Note semilogarithmic scale.

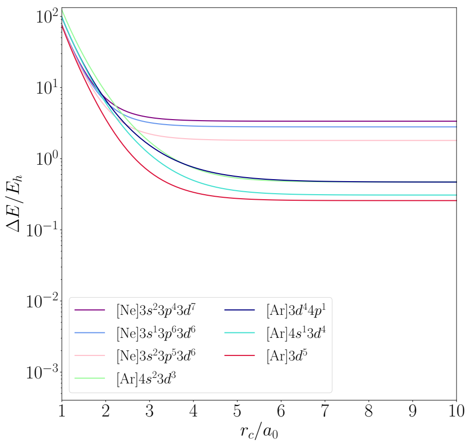

(a) PW92

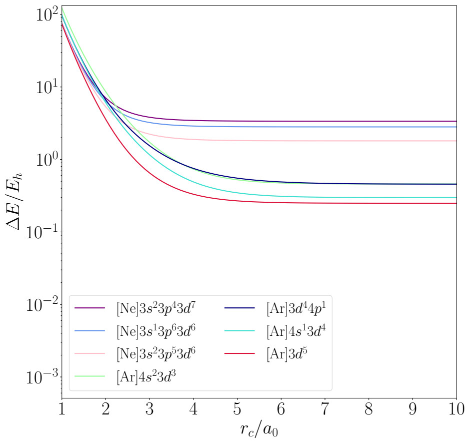

(b) PBE

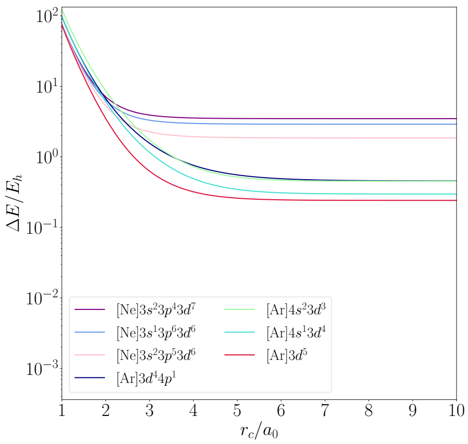

(c) r^{2}SCAN

Figure S54: Energies of various low lying configurations of the hard-wall confined spin-restricted monocation of Cr shown as the energy difference from unconfined Cr as a function of the confinement radius $r_\infty = 1.0, 1.1, \ldots, 10.0 a_0$. Note semilogarithmic scale.

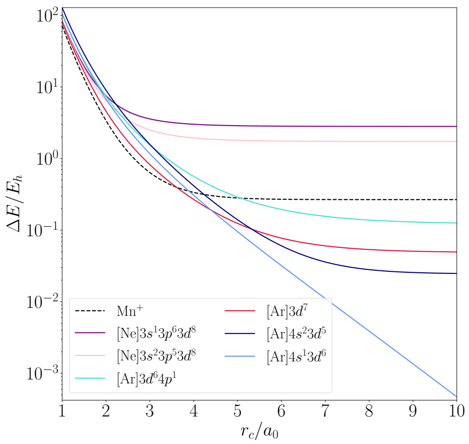

(a) PW92

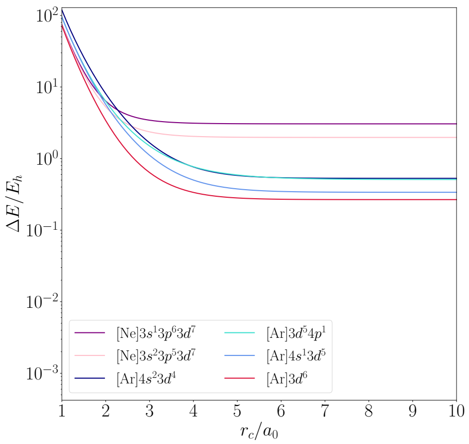

(a) PW92

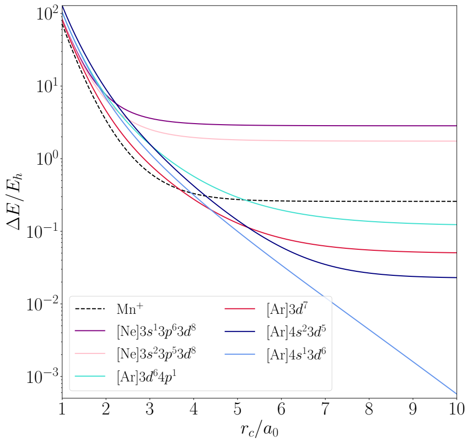

(b) PBE

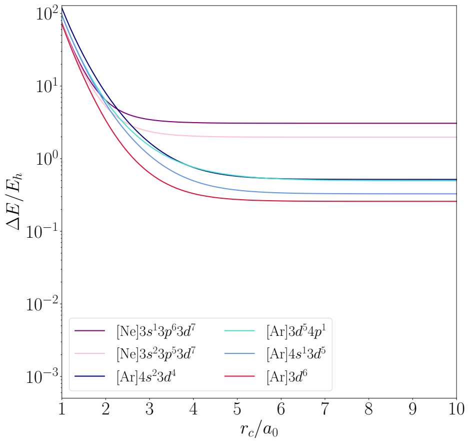

(b) PBE

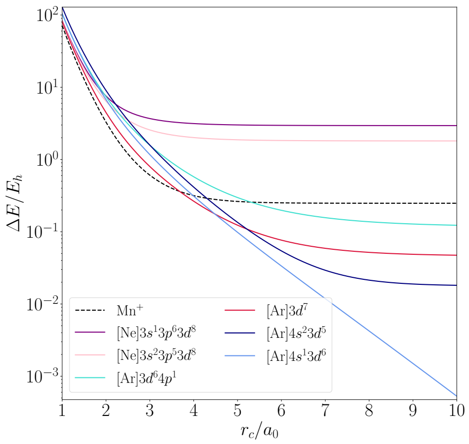

(c) r^{2}SCAN

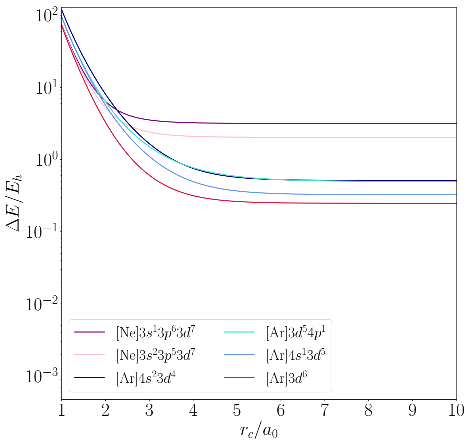

(c) r^{2}SCAN

Figure S55: Energies of various low lying configurations of hard-wall confined spin-restricted Mn shown as the energy difference from unconfined Mn as a function of the confinement radius $r_\infty = 1.0, 1.1, \ldots, 10.0 a_0$. Note semilogarithmic scale.

Figure S56: Energies of various low lying configurations of the hard-wall confined spin-restricted monocation of Mn shown as the energy difference from unconfined Mn as a function of the confinement radius $r_\infty = 1.0, 1.1, \ldots, 10.0 a_0$. Note semilogarithmic scale.

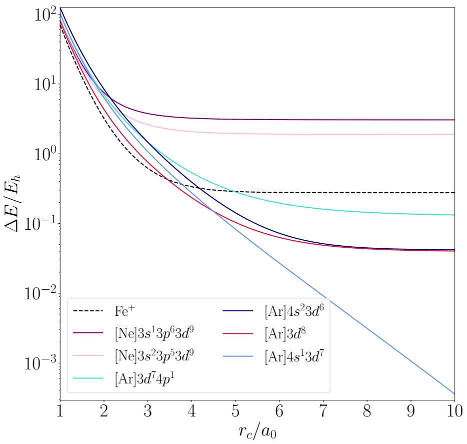

(a) PW92

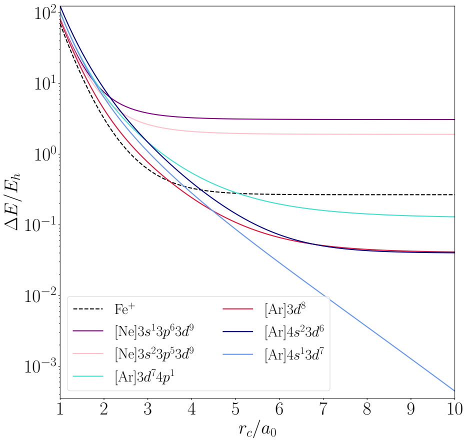

(b) PBE

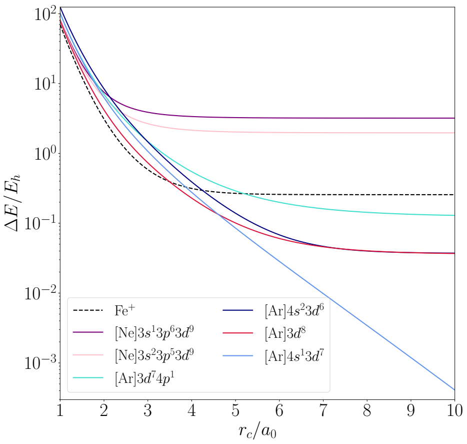

(c) r^{2}SCAN

Figure S57: Energies of various low lying configurations of hard-wall confined spin-restricted Fe shown as the energy difference from unconfined Fe as a function of the confinement radius $r_\infty = 1.0, 1.1, \ldots, 10.0 a_0$. Note semilogarithmic scale.

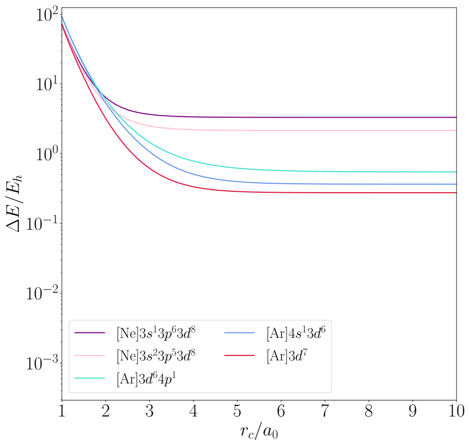

(a) PW92

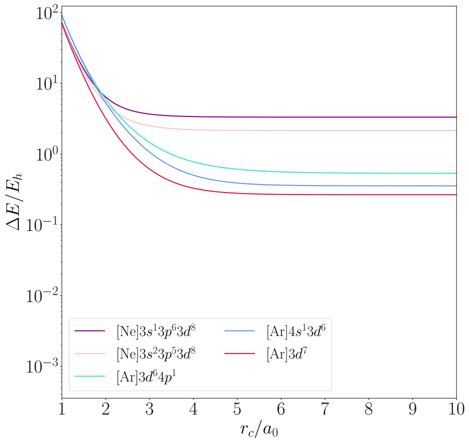

(b) PBE

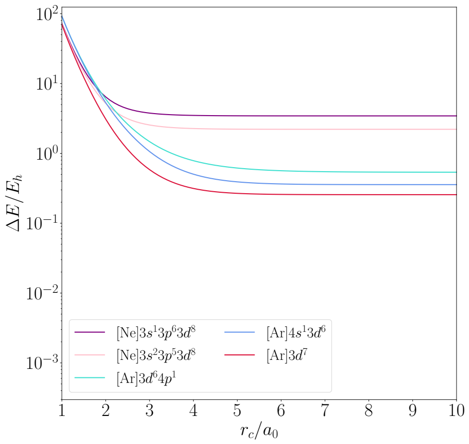

(c) r^{2}SCAN

Figure S58: Energies of various low lying configurations of the hard-wall confined spin-restricted monocation of Fe shown as the energy difference from unconfined Fe as a function of the confinement radius $r_\infty = 1.0, 1.1, \ldots, 10.0 a_0$. Note semilogarithmic scale.

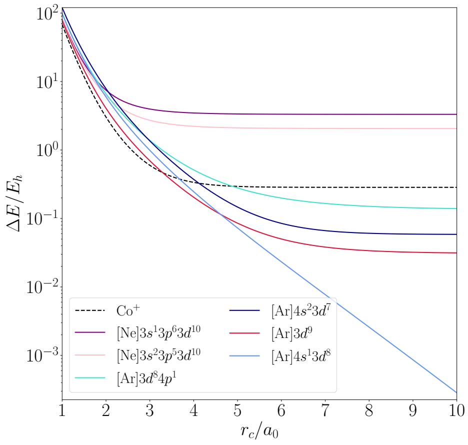

(a) PW92

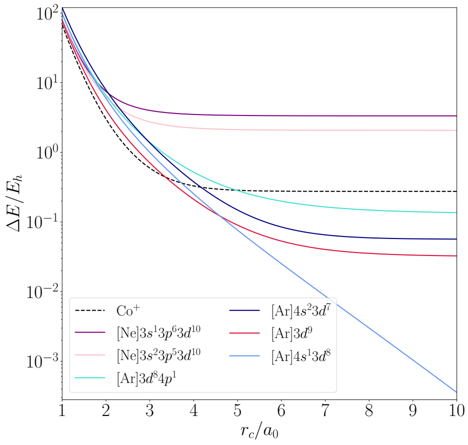

(b) PBE

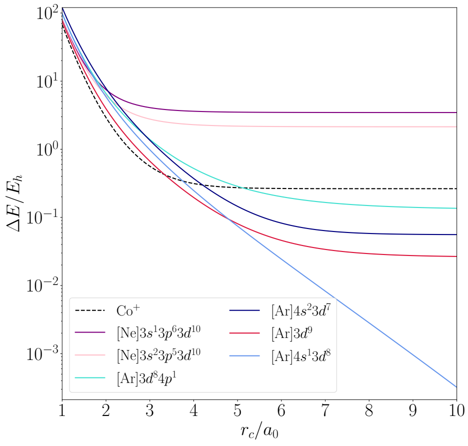

(c) r^{2}SCAN

Figure S59: Energies of various low lying configurations of hard-wall confined spin-restricted Co shown as the energy difference from unconfined Co as a function of the confinement radius $r_\infty = 1.0, 1.1, \ldots, 10.0 a_0$. Note semilogarithmic scale.

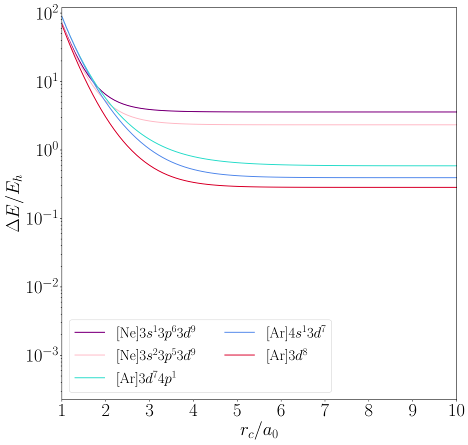

(a) PW92

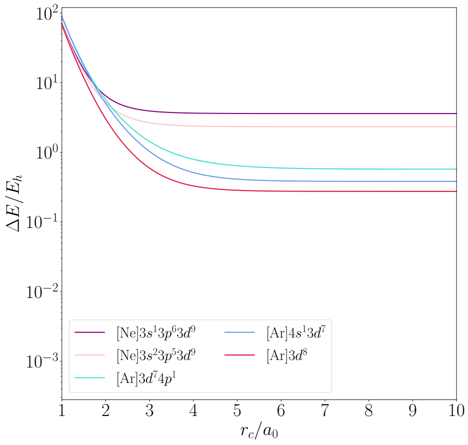

(b) PBE

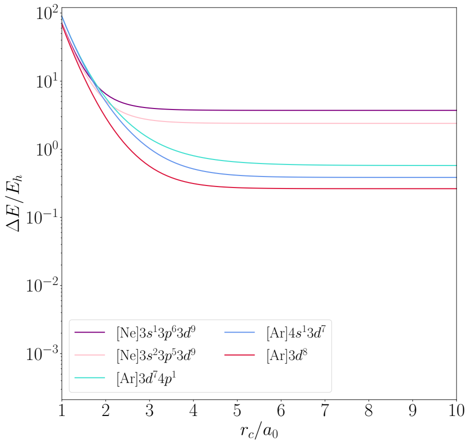

(c) r^{2}SCAN

Figure S60: Energies of various low lying configurations of the hard-wall confined spin-restricted monocation of Co shown as the energy difference from unconfined Co as a function of the confinement radius $r_\infty = 1.0, 1.1, \ldots, 10.0 a_0$. Note semilogarithmic scale.

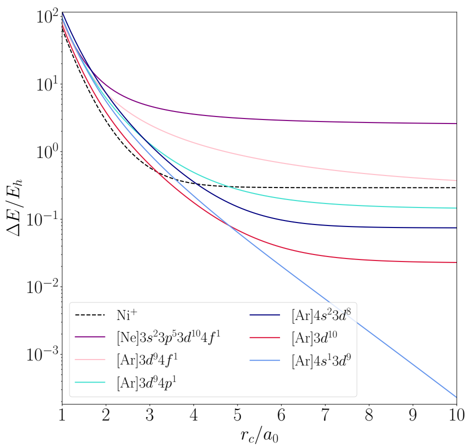

(a) PW92

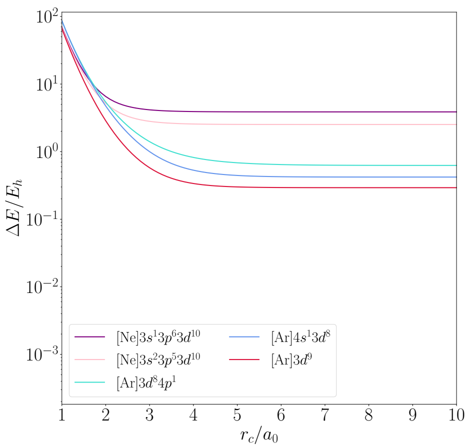

(a) PW92

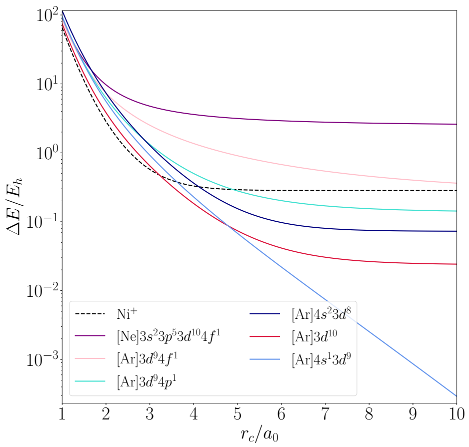

(b) PBE

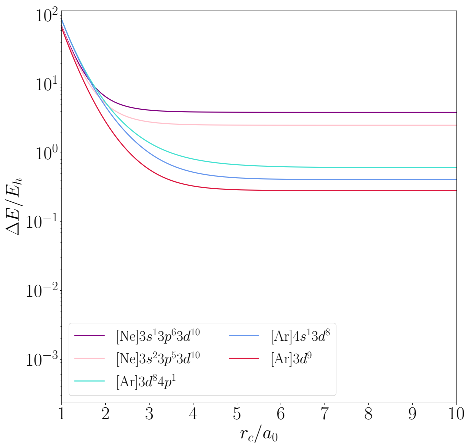

(b) PBE

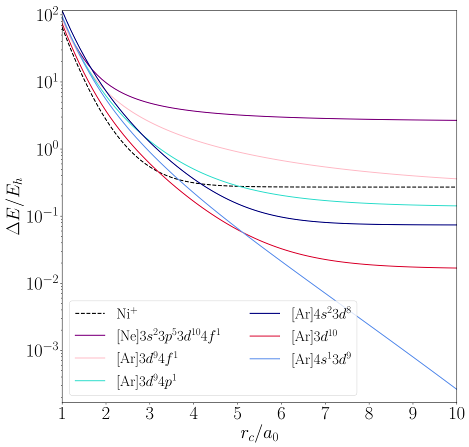

(c) r^{2}SCAN

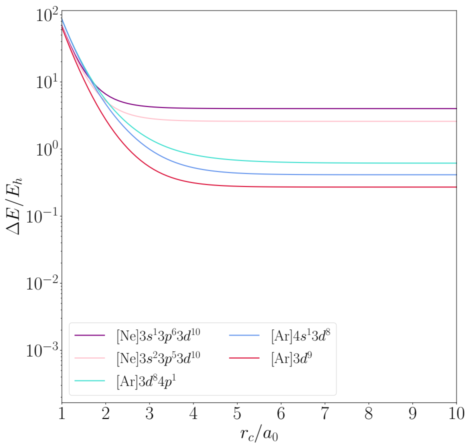

(c) r^{2}SCAN

Figure S61: Energies of various low lying configurations of hard-wall confined spin-restricted Ni shown as the energy difference from unconfined Ni as a function of the confinement radius $r_\infty = 1.0, 1.1, \ldots, 10.0 a_0$. Note semilogarithmic scale.

Figure S62: Energies of various low lying configurations of the hard-wall confined spin-restricted monocation of Ni shown as the energy difference from unconfined Ni as a function of the confinement radius $r_\infty = 1.0, 1.1, \ldots, 10.0 a_0$. Note semilogarithmic scale.

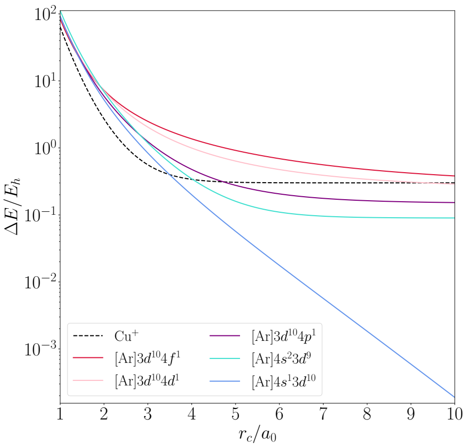

(a) PW92

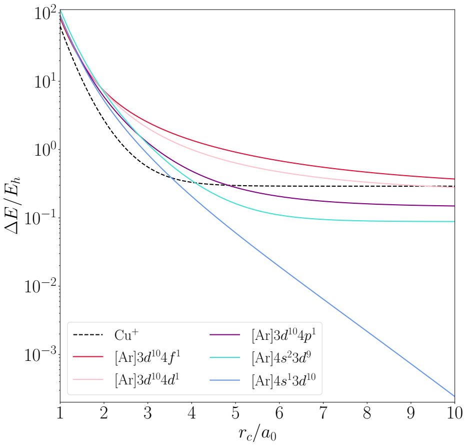

(b) PBE

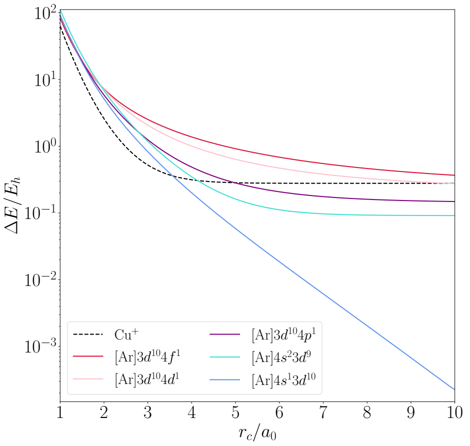

(c) r^{2}SCAN

Figure S63: Energies of various low lying configurations of hard-wall confined spin-restricted Cu shown as the energy difference from unconfined Cu as a function of the confinement radius $r_\infty = 1.0, 1.1, \ldots, 10.0a_0$. Note semilogarithmic scale.

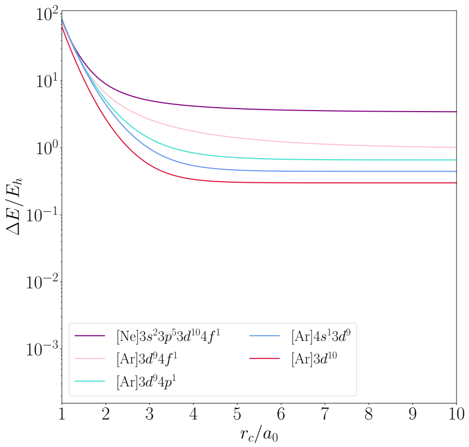

(a) PW92

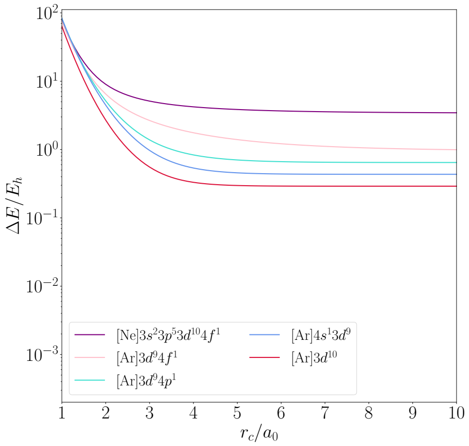

(b) PBE

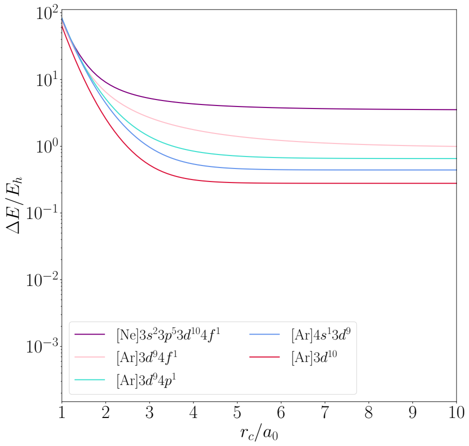

(c) r^{2}SCAN

Figure S64: Energies of various low lying configurations of the hard-wall confined spin-restricted monocation of Cu shown as the energy difference from unconfined Cu as a function of the confinement radius $r_\infty = 1.0, 1.1, \ldots, 10.0a_0$. Note semilogarithmic scale.

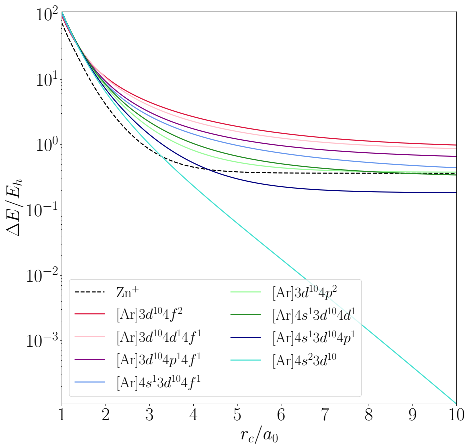

(a) PW92

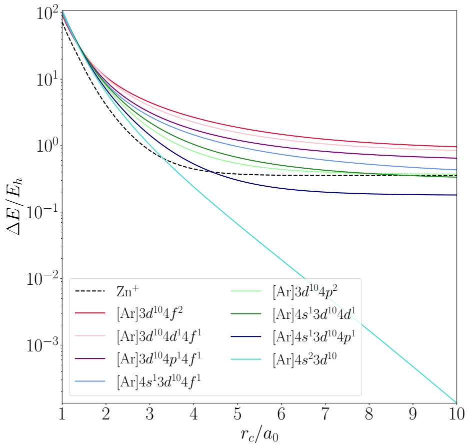

(b) PBE

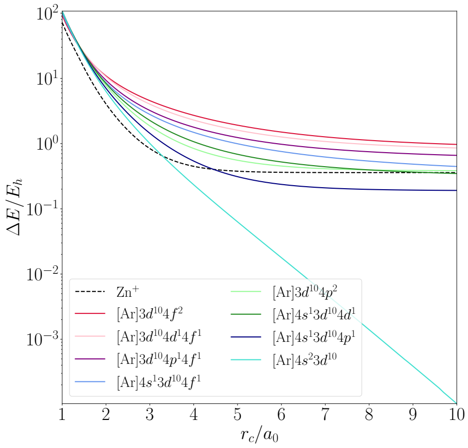

(c) r^{2}SCAN

Figure S65: Energies of various low lying configurations of hard-wall confined spin-restricted Zn shown as the energy difference from unconfined Zn as a function of the confinement radius $r_\infty = 1.0, 1.1, \ldots, 10.0a_0$. Note semilogarithmic scale.

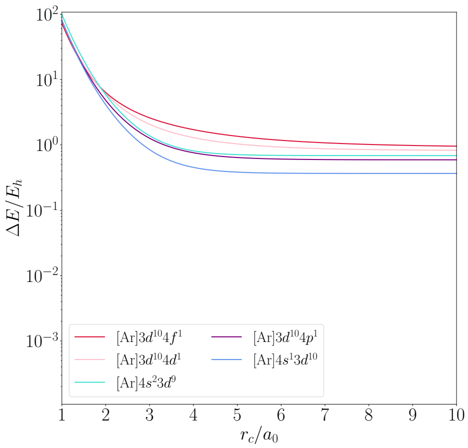

(a) PW92

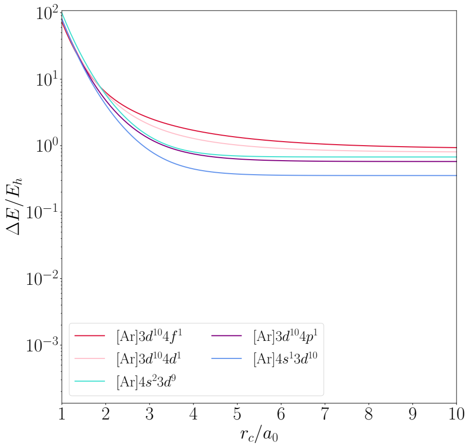

(b) PBE

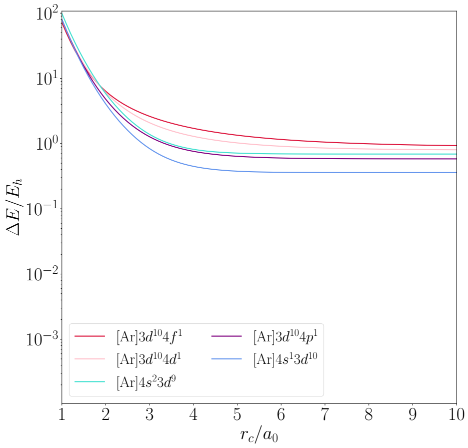

(c) r^{2}SCAN

Figure S66: Energies of various low lying configurations of the hard-wall confined spin-restricted monocation of Zn shown as the energy difference from unconfined Zn as a function of the confinement radius $r_\infty = 1.0, 1.1, \ldots, 10.0a_0$. Note semilogarithmic scale.

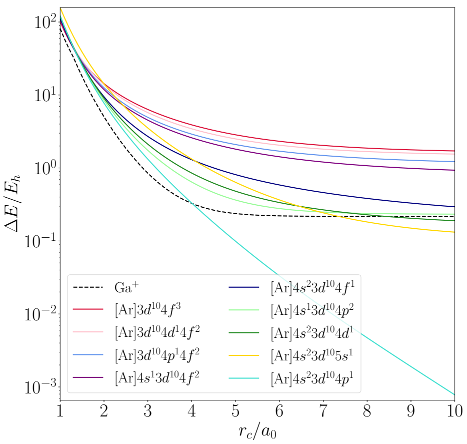

(a) PW92

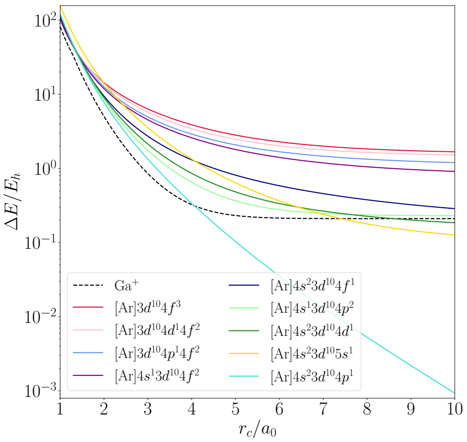

(b) PBE

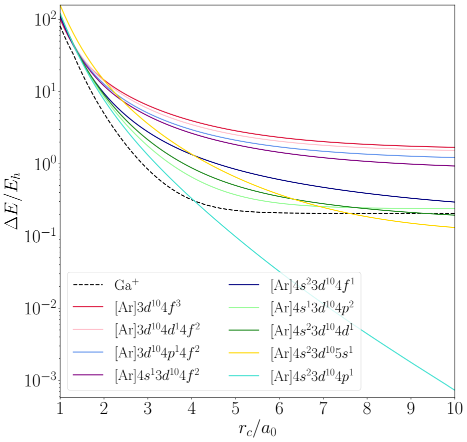

(c) r^{2}SCAN

Figure S67: Energies of various low lying configurations of hard-wall confined spin-restricted Ga shown as the energy difference from unconfined Ga as a function of the confinement radius $r_\infty = 1.0, 1.1, \ldots, 10.0 a_0$. Note semilogarithmic scale.

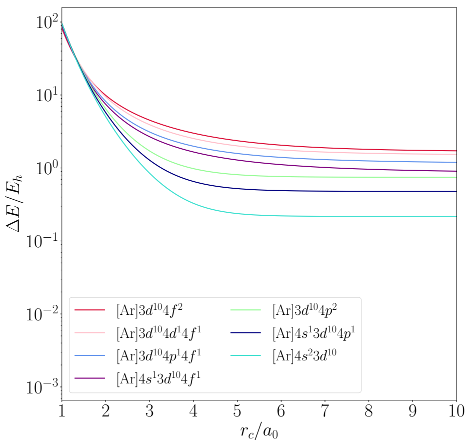

(a) PW92

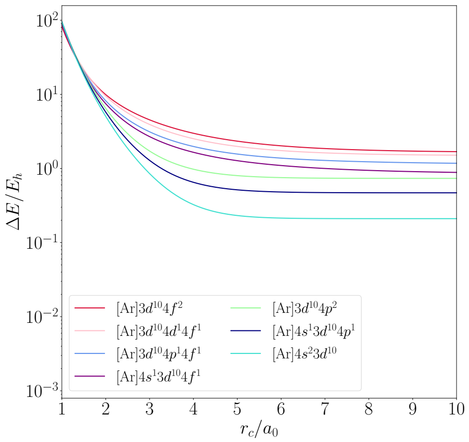

(b) PBE

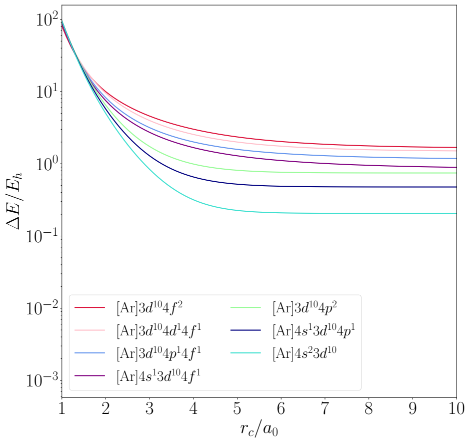

(c) r^{2}SCAN

Figure S68: Energies of various low lying configurations of the hard-wall confined spin-restricted monocation of Ga shown as the energy difference from unconfined Ga as a function of the confinement radius $r_\infty = 1.0, 1.1, \ldots, 10.0 a_0$. Note semilogarithmic scale.

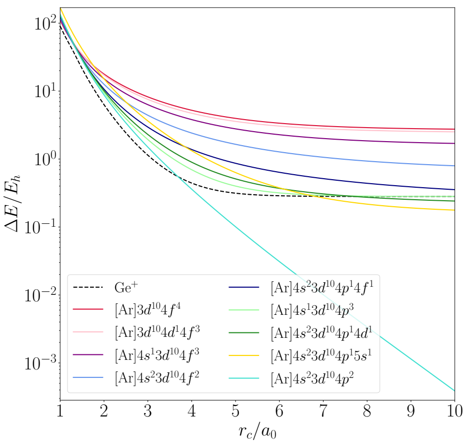

(a) PW92

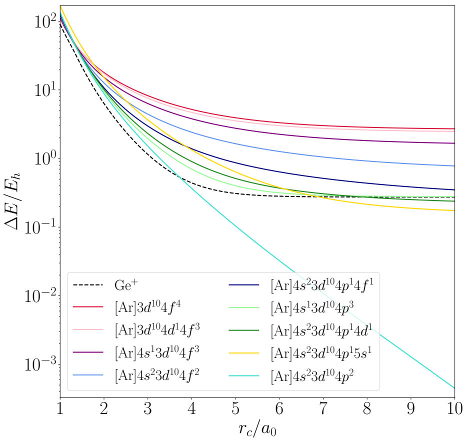

(b) PBE

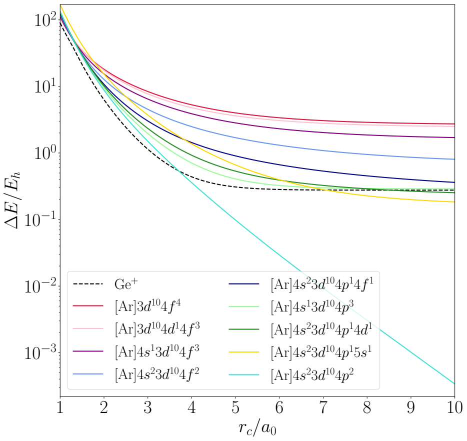

(c) r^{2}SCAN

Figure S69: Energies of various low lying configurations of hard-wall confined spin-restricted Ge shown as the energy difference from unconfined Ge as a function of the confinement radius $r_\infty = 1.0, 1.1, \ldots, 10.0a_0$. Note semilogarithmic scale.

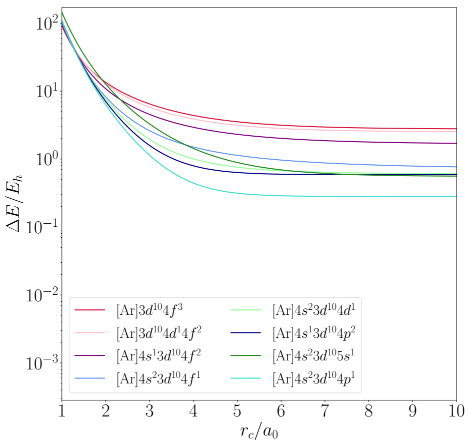

(a) PW92

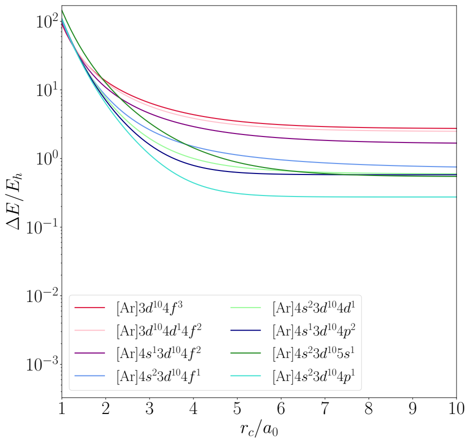

(b) PBE

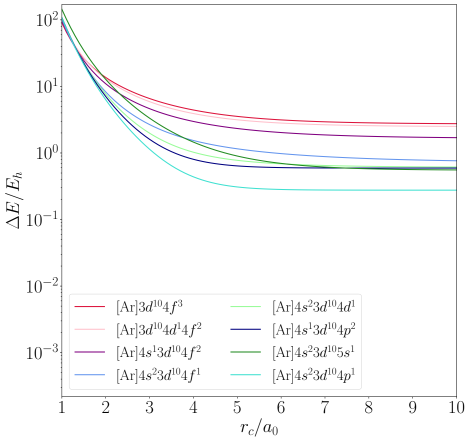

(c) r^{2}SCAN

Figure S70: Energies of various low lying configurations of the hard-wall confined spin-restricted monocation of Ge shown as the energy difference from unconfined Ge as a function of the confinement radius $r_\infty = 1.0, 1.1, \ldots, 10.0a_0$. Note semilogarithmic scale.

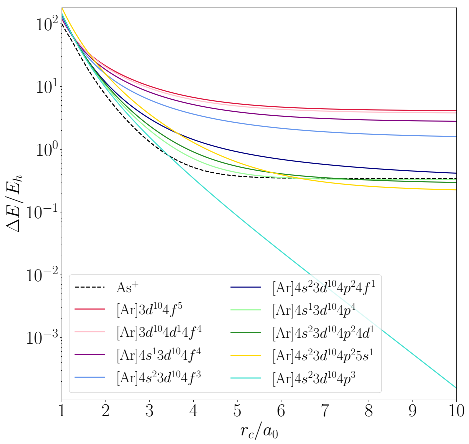

(a) PW92

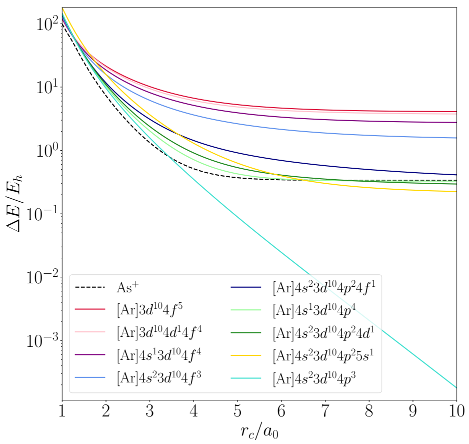

(b) PBE

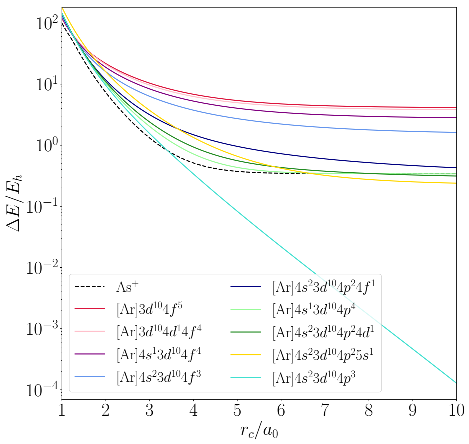

(c) r^{2}SCAN

Figure S71: Energies of various low lying configurations of hard-wall confined spin-restricted As shown as the energy difference from unconfined As as a function of the confinement radius $r_\infty = 1.0, 1.1, \ldots, 10.0 a_0$. Note semilogarithmic scale.

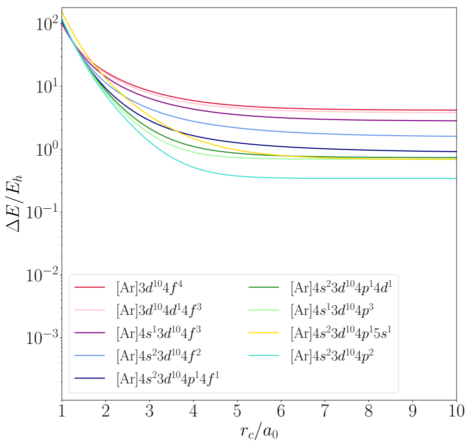

(a) PW92

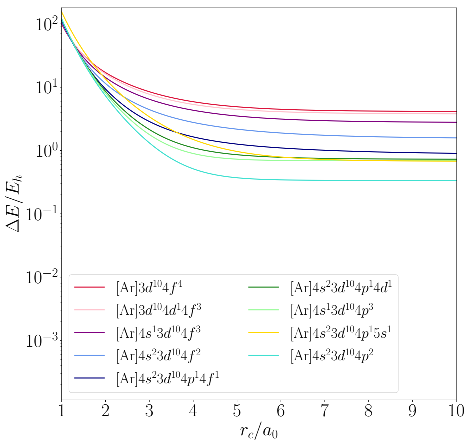

(b) PBE

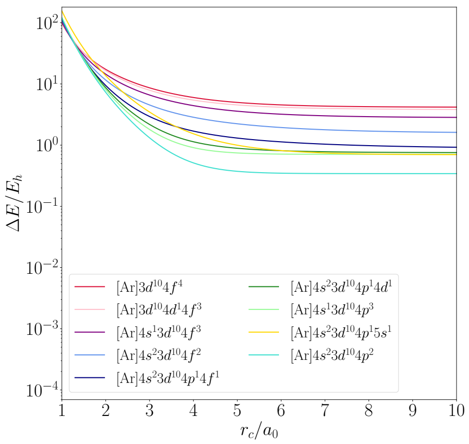

(c) r^{2}SCAN

Figure S72: Energies of various low lying configurations of the hard-wall confined spin-restricted monocation of As shown as the energy difference from unconfined As as a function of the confinement radius $r_\infty = 1.0, 1.1, \ldots, 10.0 a_0$. Note semilogarithmic scale.

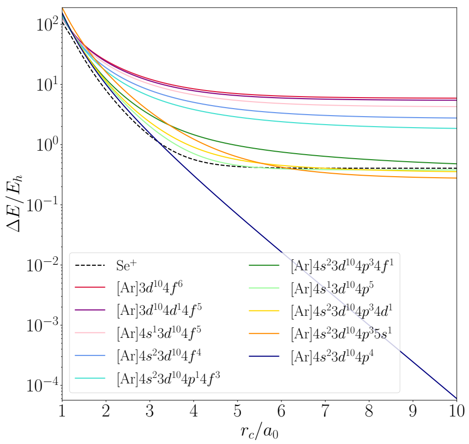

(a) PW92

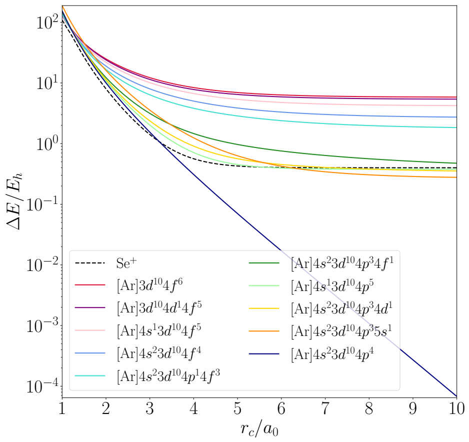

(b) PBE

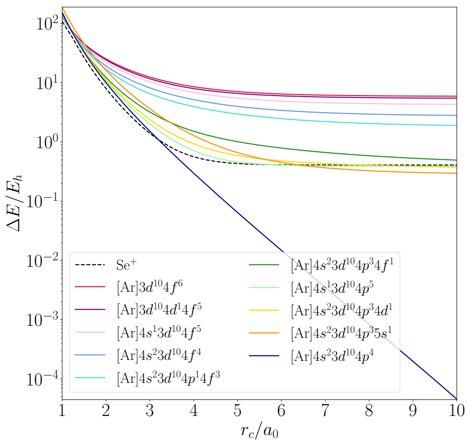

(c) r^{2}SCAN

Figure S73: Energies of various low lying configurations of hard-wall confined spin-restricted Se shown as the energy difference from unconfined Se as a function of the confinement radius $r_\infty = 1.0, 1.1, \ldots, 10.0a_0$. Note semilogarithmic scale.

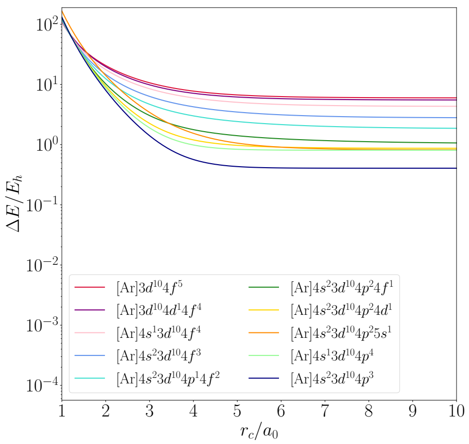

(a) PW92

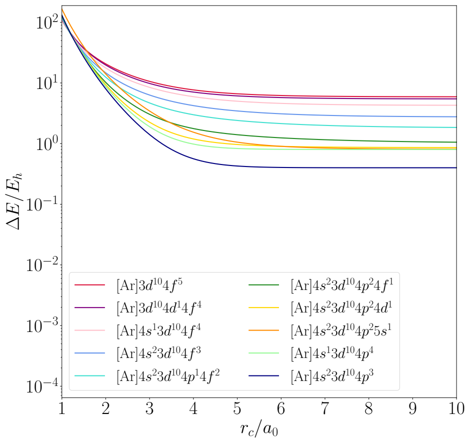

(b) PBE

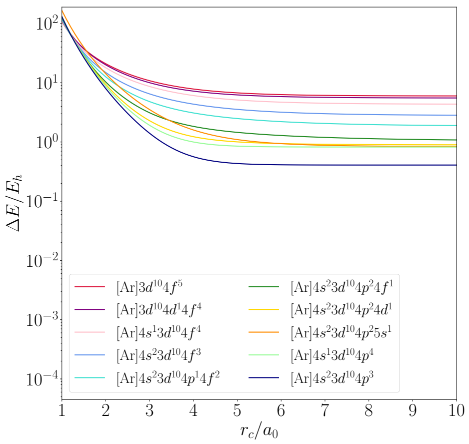

(c) r^{2}SCAN

Figure S74: Energies of various low lying configurations of the hard-wall confined spin-restricted monocation of Se shown as the energy difference from unconfined Se as a function of the confinement radius $r_\infty = 1.0, 1.1, \ldots, 10.0a_0$. Note semilogarithmic scale.

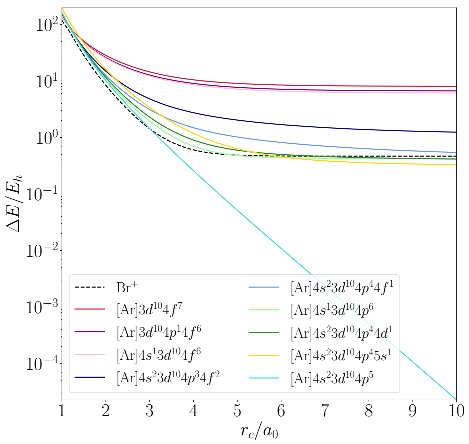

(a) PW92

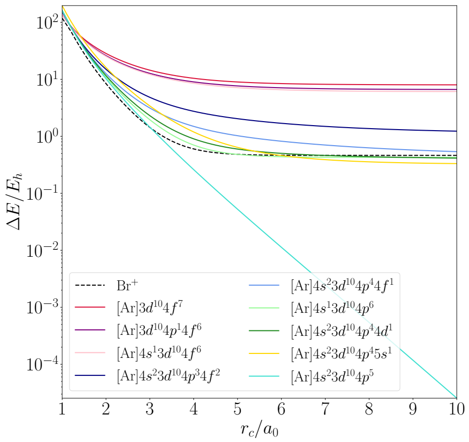

(b) PBE

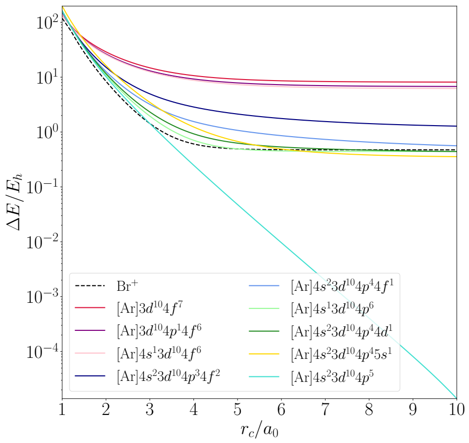

(c) r^{2}SCAN

Figure S75: Energies of various low lying configurations of hard-wall confined spin-restricted Br shown as the energy difference from unconfined Br as a function of the confinement radius $r_\infty = 1.0, 1.1, \ldots, 10.0a_0$. Note semilogarithmic scale.

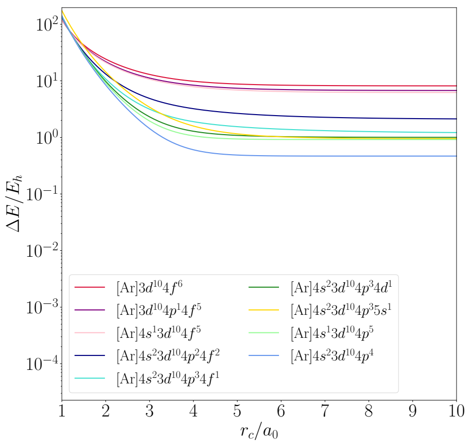

(a) PW92

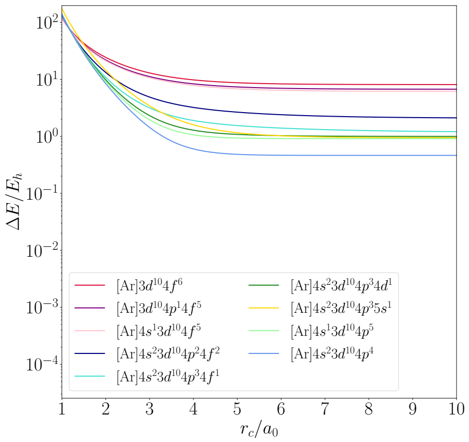

(b) PBE

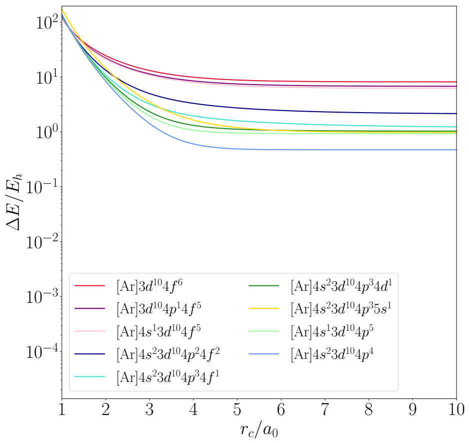

(c) r^{2}SCAN

Figure S76: Energies of various low lying configurations of the hard-wall confined spin-restricted monocation of Br shown as the energy difference from unconfined Br as a function of the confinement radius $r_\infty = 1.0, 1.1, \ldots, 10.0a_0$. Note semilogarithmic scale.

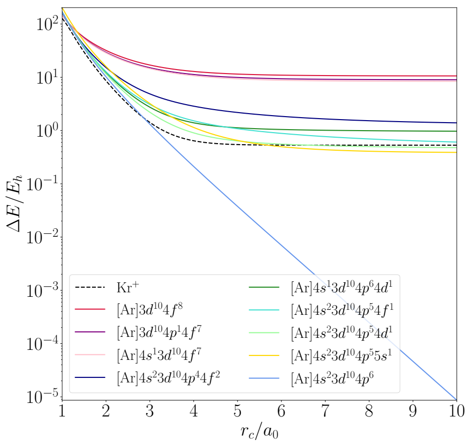

(a) PW92

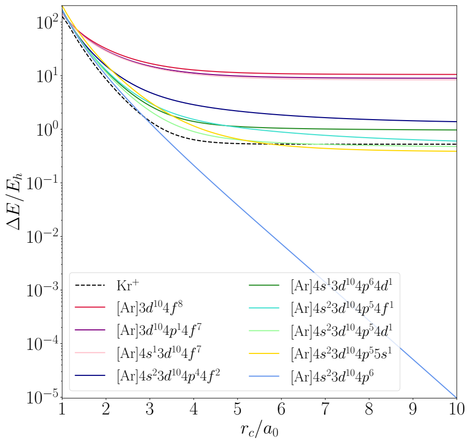

(b) PBE

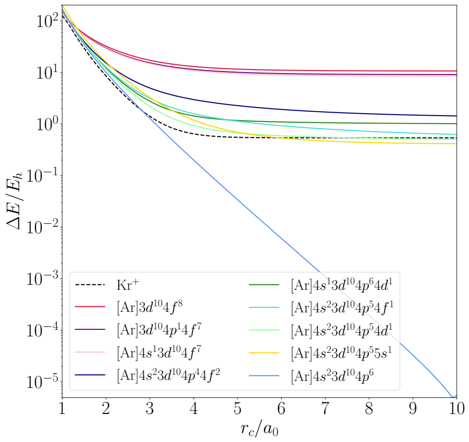

(c) r^{2}SCAN

Figure S77: Energies of various low lying configurations of hard-wall confined spin-restricted Kr shown as the energy difference from unconfined Kr as a function of the confinement radius $r_\infty = 1.0, 1.1, \ldots, 10.0a_0$. Note semilogarithmic scale.

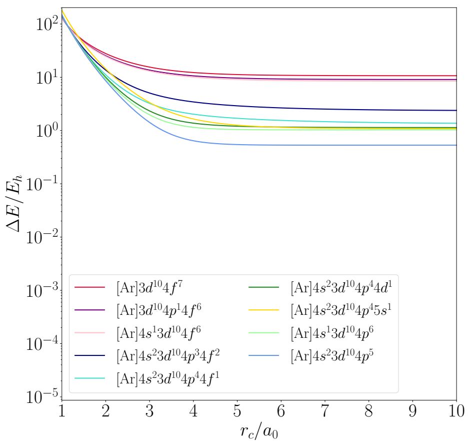

(a) PW92

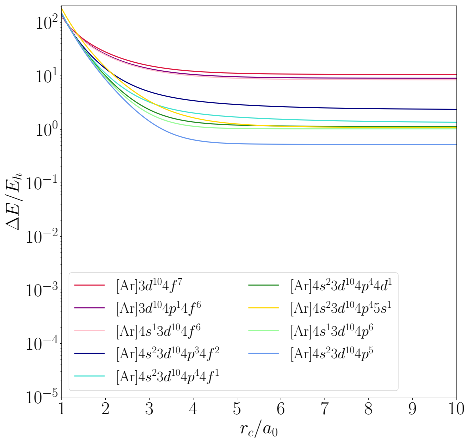

(b) PBE

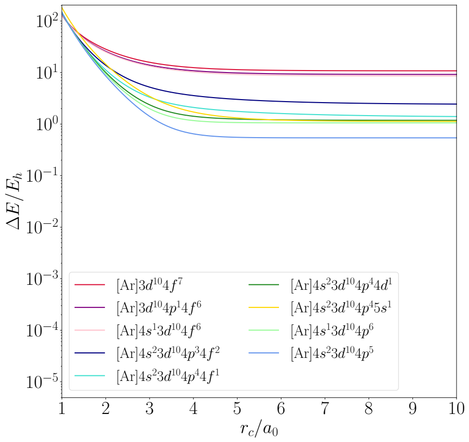

(c) r^{2}SCAN

Figure S78: Energies of various low lying configurations of the hard-wall confined spin-restricted monocation of Kr shown as the energy difference from unconfined Kr as a function of the confinement radius $r_\infty = 1.0, 1.1, \ldots, 10.0a_0$. Note semilogarithmic scale.

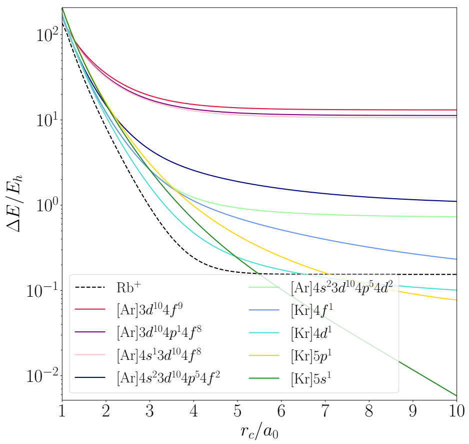

(a) PW92

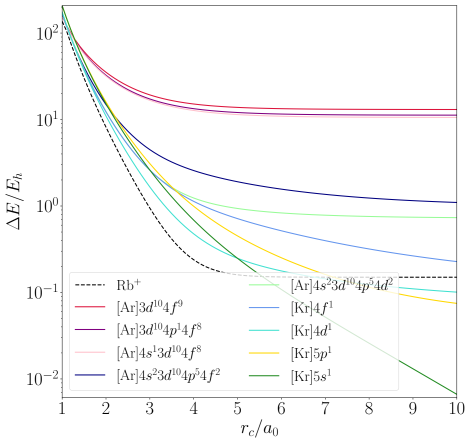

(b) PBE

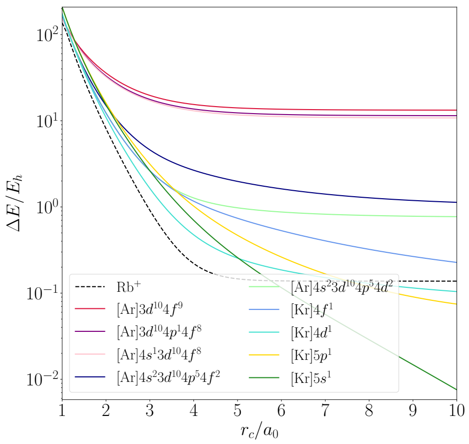

(c) r^{2}SCAN

Figure S79: Energies of various low lying configurations of hard-wall confined spin-restricted Rb shown as the energy difference from unconfined Rb as a function of the confinement radius $r_\infty = 1.0, 1.1, \ldots, 10.0 a_0$. Note semilogarithmic scale.

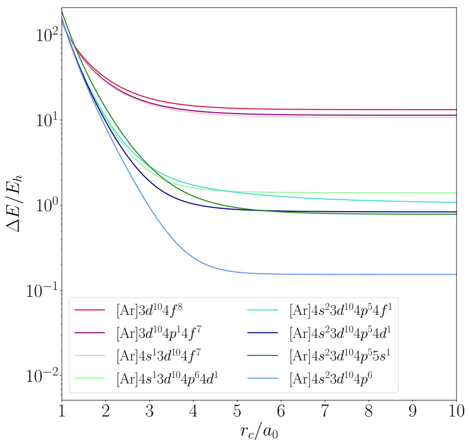

(a) PW92

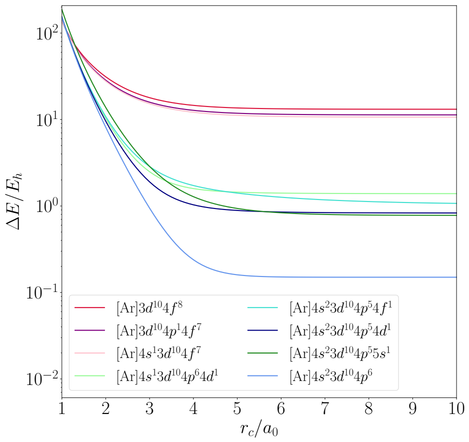

(b) PBE

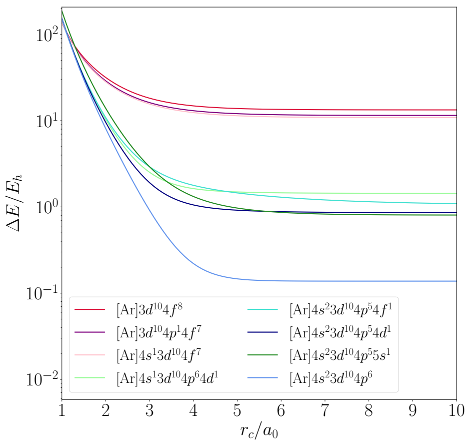

(c) r^{2}SCAN

Figure S80: Energies of various low lying configurations of the hard-wall confined spin-restricted monocation of Rb shown as the energy difference from unconfined Rb as a function of the confinement radius $r_\infty = 1.0, 1.1, \ldots, 10.0 a_0$. Note semilogarithmic scale.

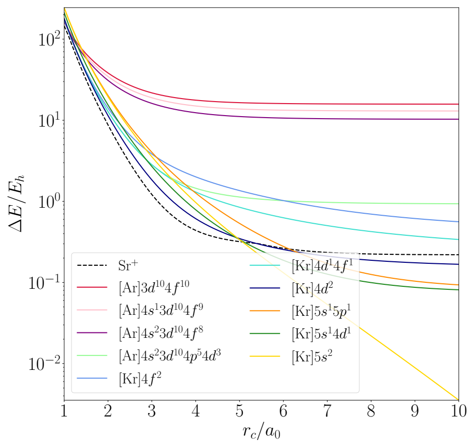

(a) PW92

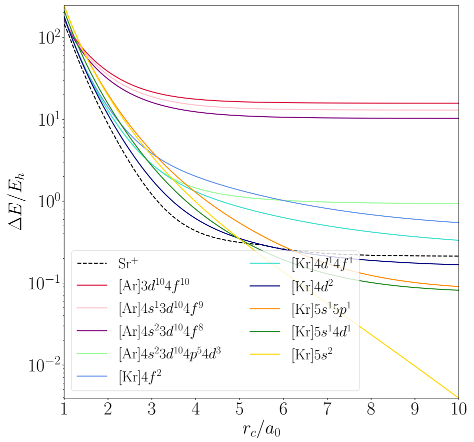

(b) PBE

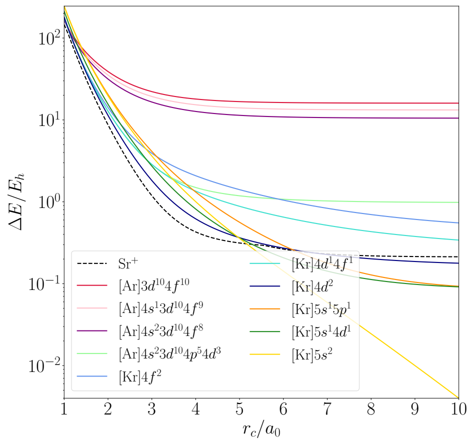

(c) r^{2}SCAN

Figure S81: Energies of various low lying configurations of hard-wall confined spin-restricted Sr shown as the energy difference from unconfined Sr as a function of the confinement radius $r_\infty = 1.0, 1.1, \ldots, 10.0a_0$. Note semilogarithmic scale.

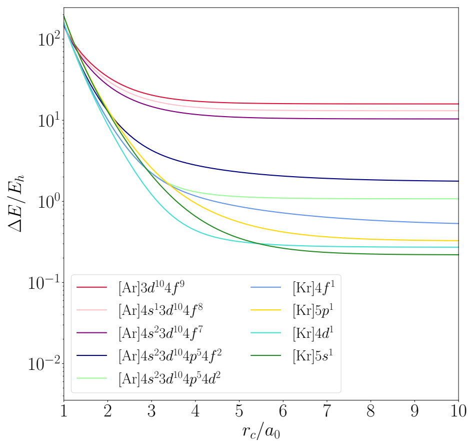

(a) PW92

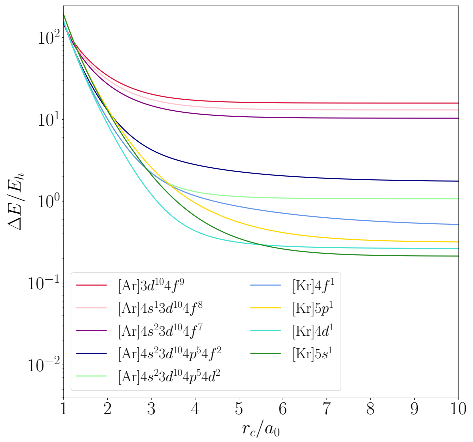

(b) PBE

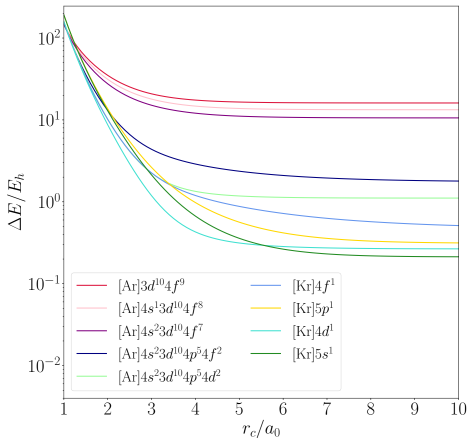

(c) r^{2}SCAN

Figure S82: Energies of various low lying configurations of the hard-wall confined spin-restricted monocation of Sr shown as the energy difference from unconfined Sr as a function of the confinement radius $r_\infty = 1.0, 1.1, \ldots, 10.0a_0$. Note semilogarithmic scale.

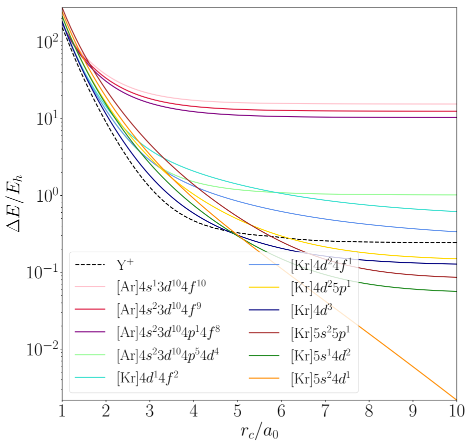

(a) PW92

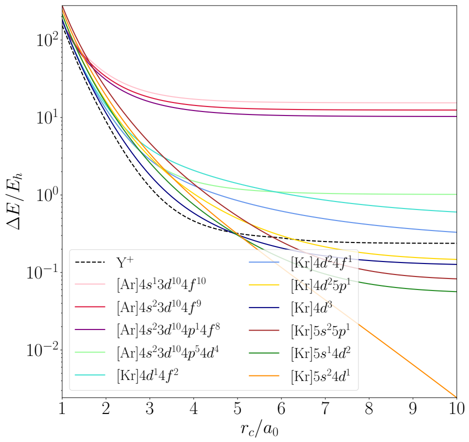

(b) PBE

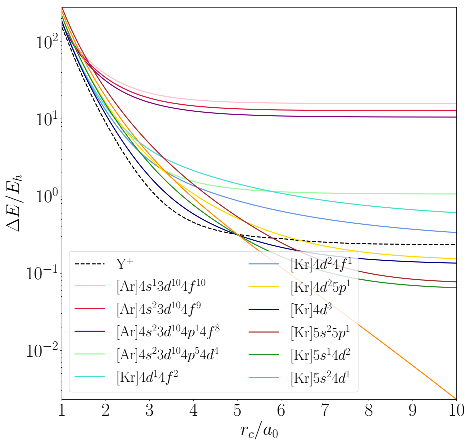

(c) r^{2}SCAN

Figure S83: Energies of various low lying configurations of hard-wall confined spin-restricted Y shown as the energy difference from unconfined Y as a function of the confinement radius $r_\infty = 1.0, 1.1, \ldots, 10.0 a_0$. Note semilogarithmic scale.

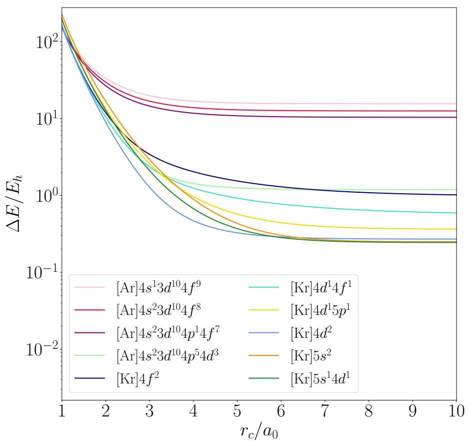

(a) PW92

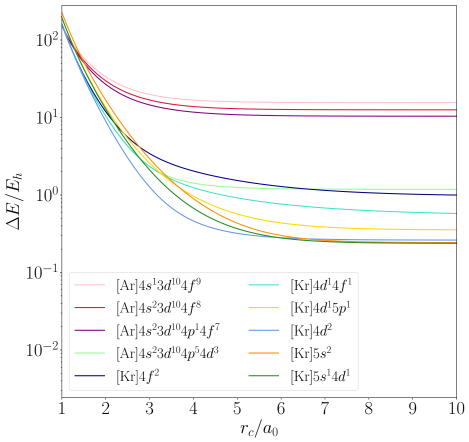

(b) PBE

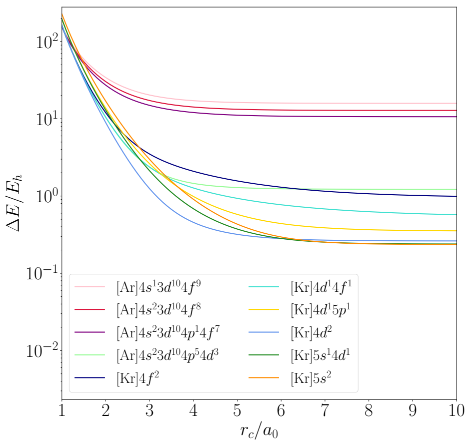

(c) r^{2}SCAN

Figure S84: Energies of various low lying configurations of the hard-wall confined spin-restricted monocation of Y shown as the energy difference from unconfined Y as a function of the confinement radius $r_\infty = 1.0, 1.1, \ldots, 10.0 a_0$. Note semilogarithmic scale.

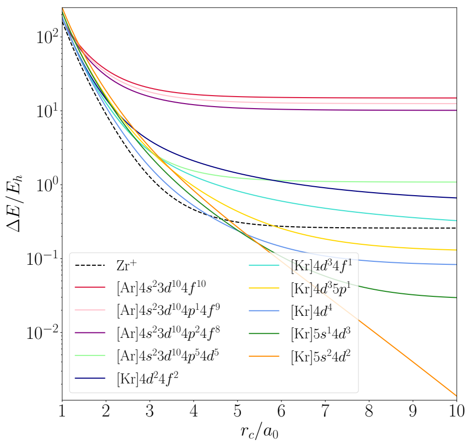

(a) PW92

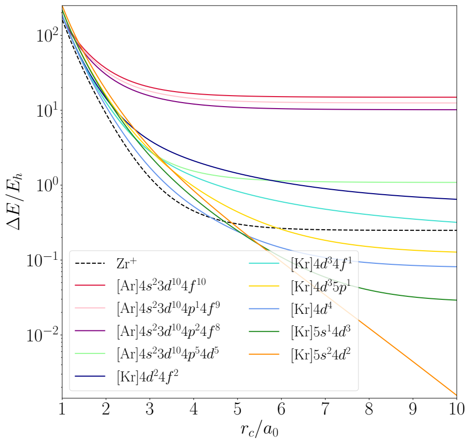

(b) PBE

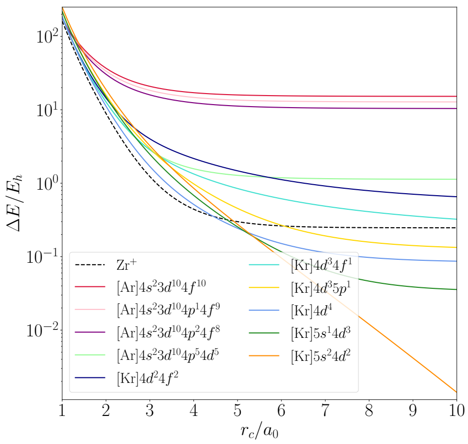

(c) r^{2}SCAN

Figure S85: Energies of various low lying configurations of hard-wall confined spin-restricted Zr shown as the energy difference from unconfined Zr as a function of the confinement radius $r_\infty = 1.0, 1.1, \ldots, 10.0 a_0$. Note semilogarithmic scale.

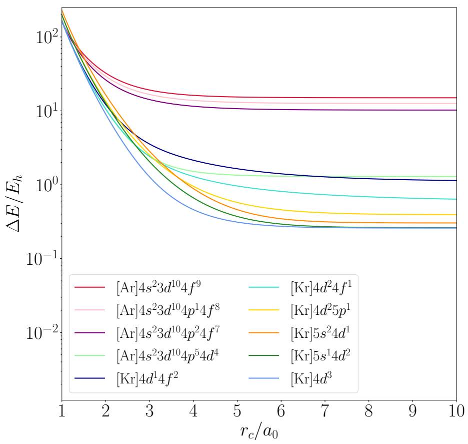

(a) PW92

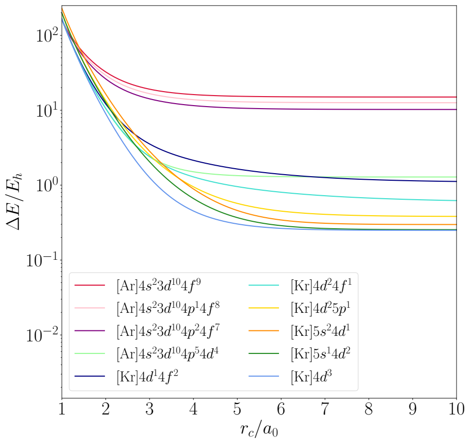

(b) PBE

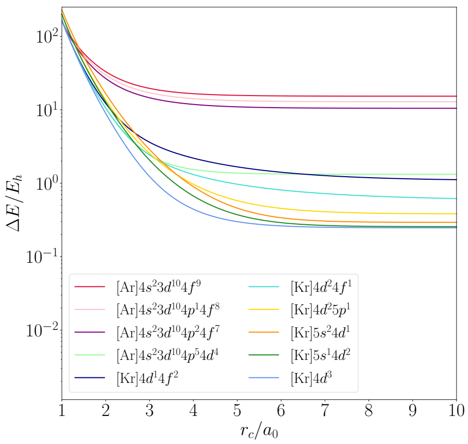

(c) r^{2}SCAN

Figure S86: Energies of various low lying configurations of the hard-wall confined spin-restricted monocation of Zr shown as the energy difference from unconfined Zr as a function of the confinement radius $r_\infty = 1.0, 1.1, \ldots, 10.0 a_0$. Note semilogarithmic scale.

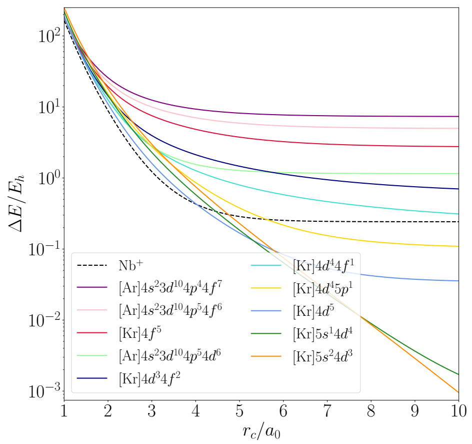

(a) PW92

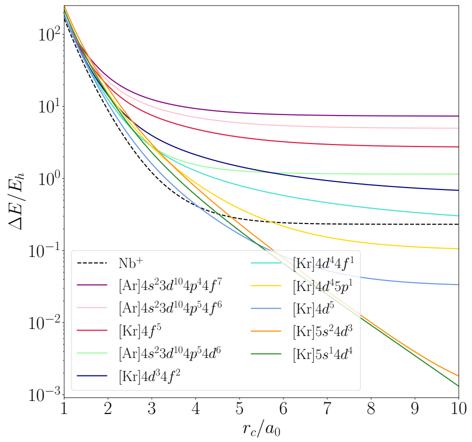

(b) PBE

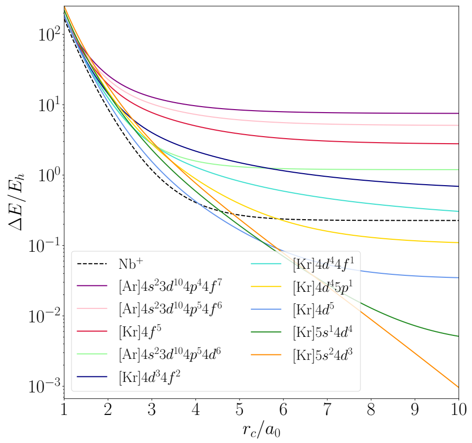

(c) r^{2}SCAN

Figure S87: Energies of various low lying configurations of hard-wall confined spin-restricted Nb shown as the energy difference from unconfined Nb as a function of the confinement radius $r_\infty = 1.0, 1.1, \ldots, 10.0a_0$. Note semilogarithmic scale.

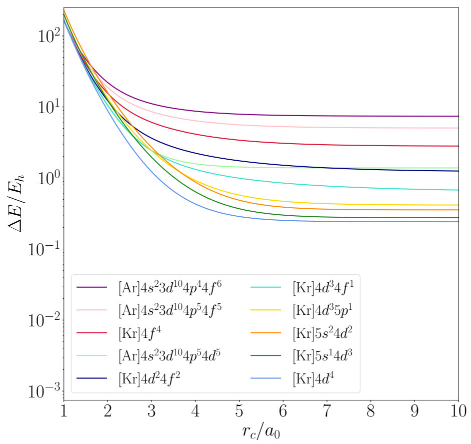

(a) PW92

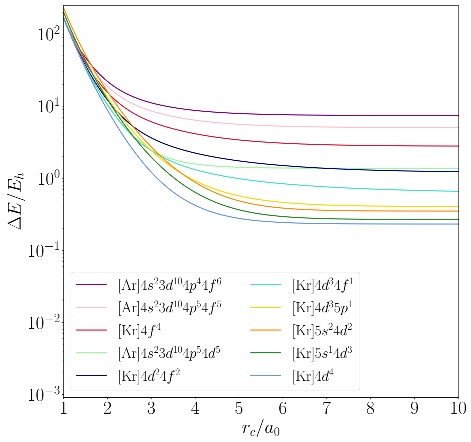

(b) PBE

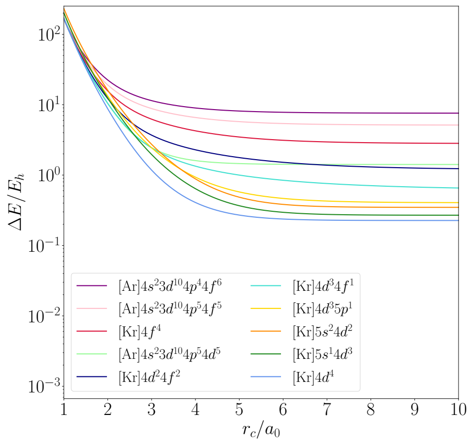

(c) r^{2}SCAN

Figure S88: Energies of various low lying configurations of the hard-wall confined spin-restricted monocation of Nb shown as the energy difference from unconfined Nb as a function of the confinement radius $r_\infty = 1.0, 1.1, \ldots, 10.0a_0$. Note semilogarithmic scale.

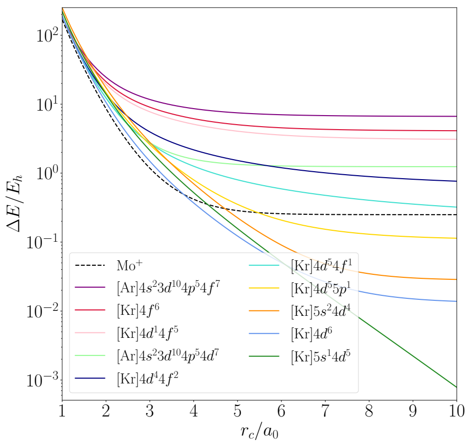

(a) PW92

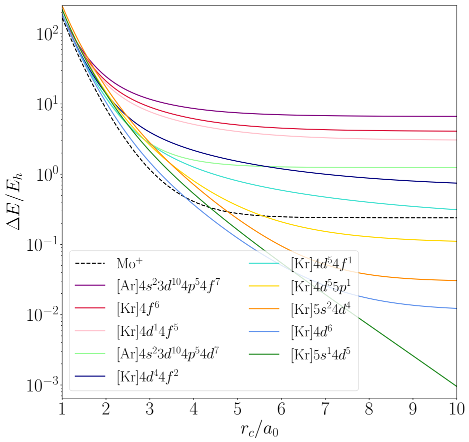

(b) PBE

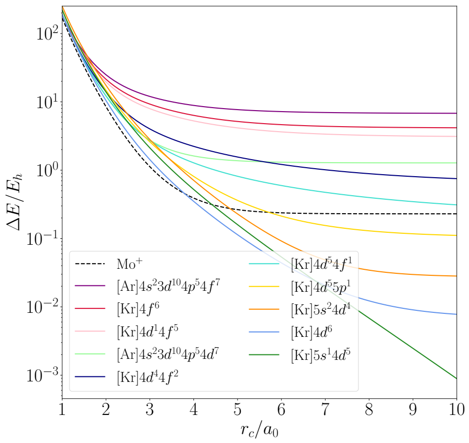

(c) r^{2}SCAN

Figure S89: Energies of various low lying configurations of hard-wall confined spin-restricted Mo shown as the energy difference from unconfined Mo as a function of the confinement radius $r_\infty = 1.0, 1.1, \ldots, 10.0a_0$. Note semilogarithmic scale.

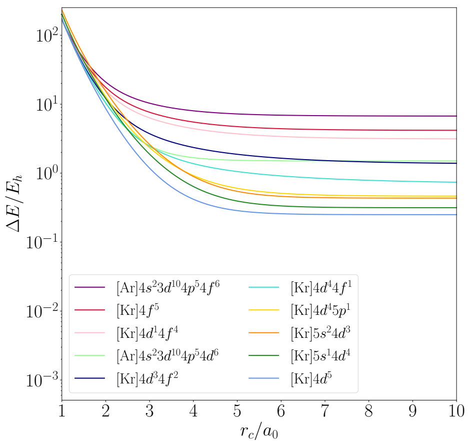

(a) PW92

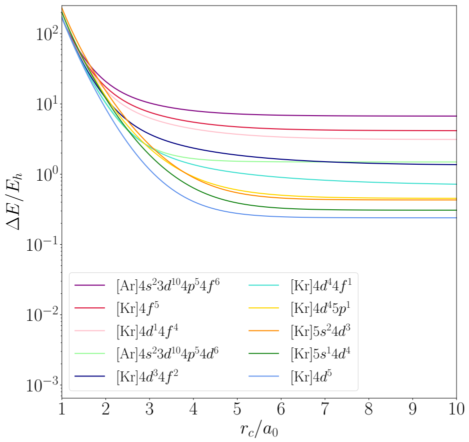

(b) PBE

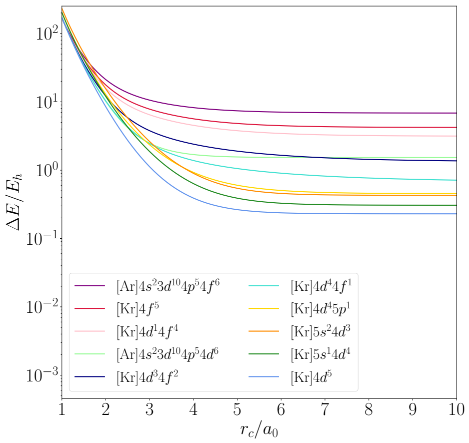

(c) r^{2}SCAN

Figure S90: Energies of various low lying configurations of the hard-wall confined spin-restricted monocation of Mo shown as the energy difference from unconfined Mo as a function of the confinement radius $r_\infty = 1.0, 1.1, \ldots, 10.0a_0$. Note semilogarithmic scale.

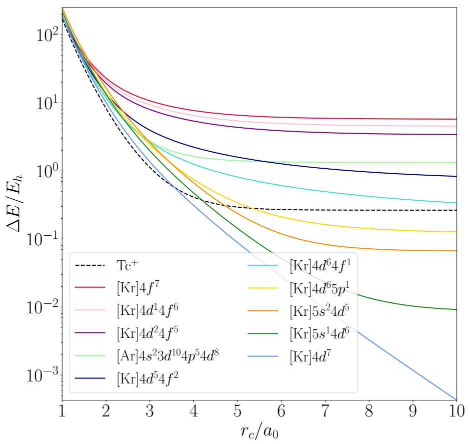

(a) PW92

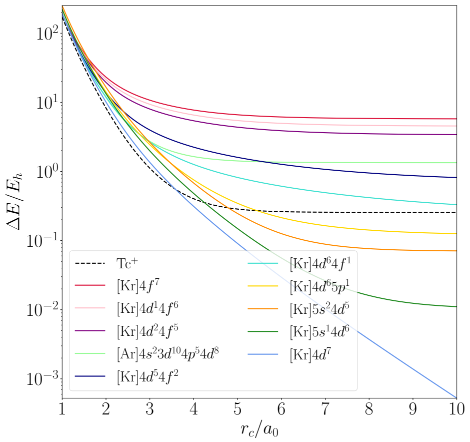

(b) PBE

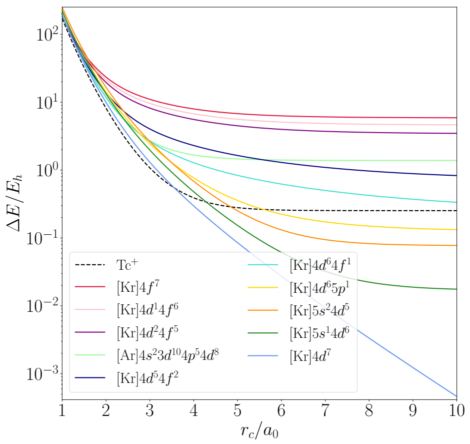

(c) r^{2}SCAN

Figure S91: Energies of various low lying configurations of hard-wall confined spin-restricted Tc shown as the energy difference from unconfined Tc as a function of the confinement radius $r_\infty = 1.0, 1.1, \ldots, 10.0a_0$. Note semilogarithmic scale.

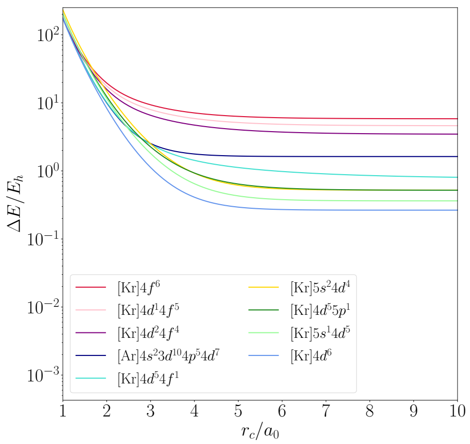

(a) PW92

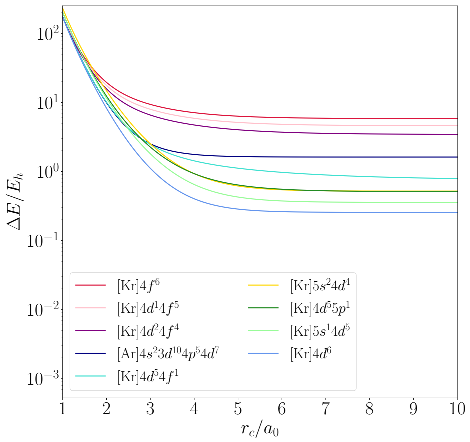

(b) PBE

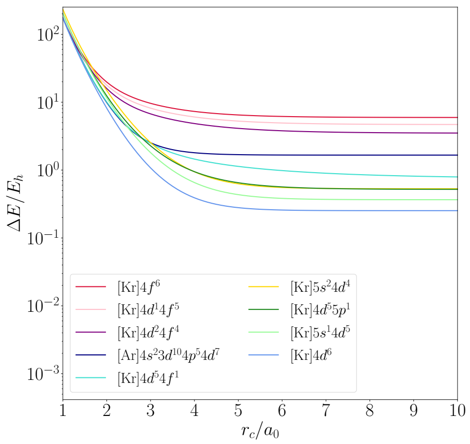

(c) r^{2}SCAN

Figure S92: Energies of various low lying configurations of the hard-wall confined spin-restricted monocation of Tc shown as the energy difference from unconfined Tc as a function of the confinement radius $r_\infty = 1.0, 1.1, \ldots, 10.0a_0$. Note semilogarithmic scale.

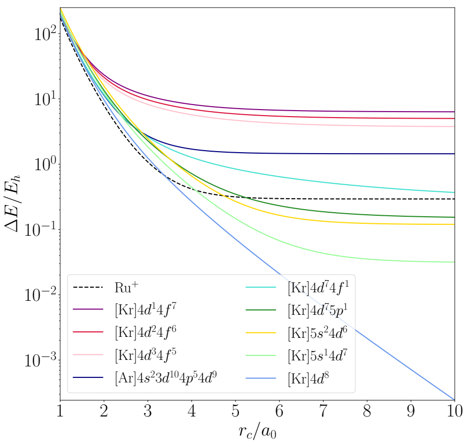

(a) PW92

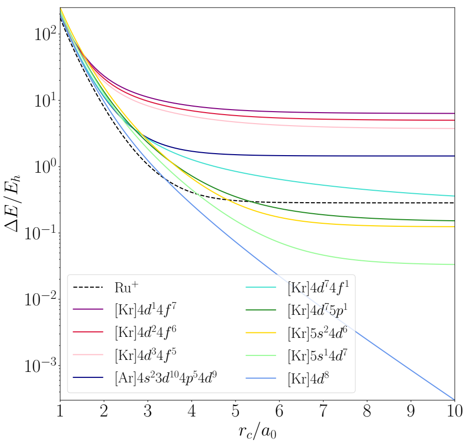

(b) PBE

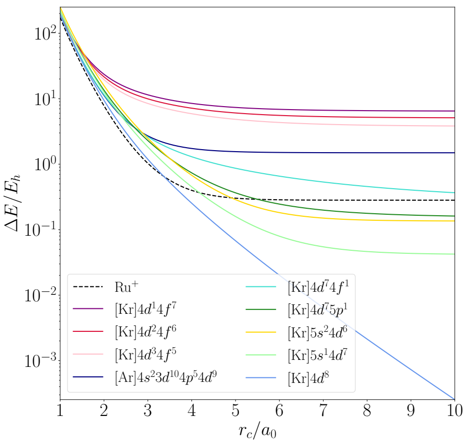

(c) r^{2}SCAN

Figure S93: Energies of various low lying configurations of hard-wall confined spin-restricted Ru shown as the energy difference from unconfined Ru as a function of the confinement radius $r_\infty = 1.0, 1.1, \ldots, 10.0 a_0$. Note semilogarithmic scale.

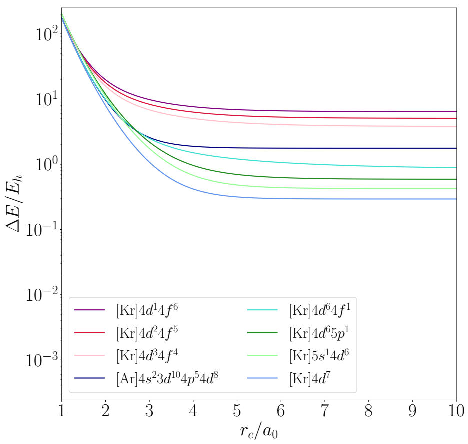

(a) PW92

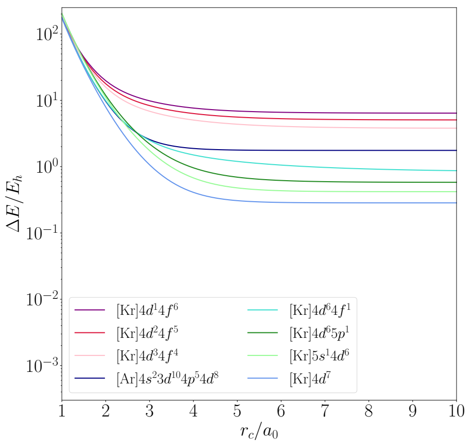

(b) PBE

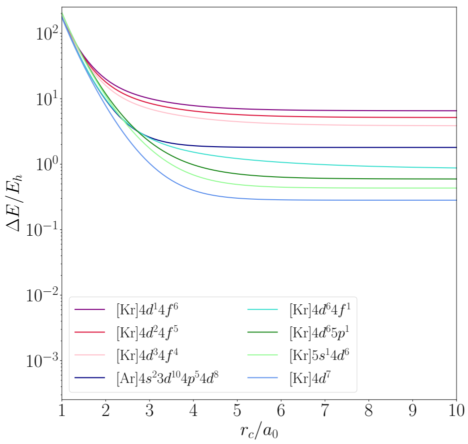

(c) r^{2}SCAN

Figure S94: Energies of various low lying configurations of the hard-wall confined spin-restricted monocation of Ru shown as the energy difference from unconfined Ru as a function of the confinement radius $r_\infty = 1.0, 1.1, \ldots, 10.0 a_0$. Note semilogarithmic scale.

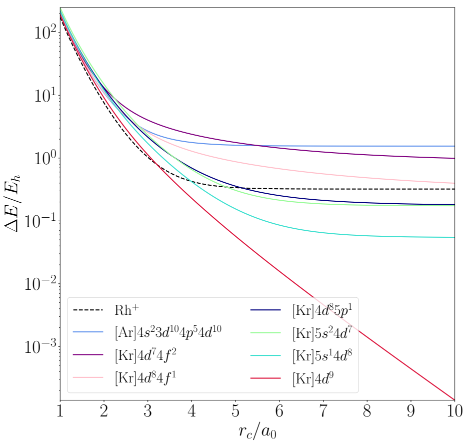

(a) PW92

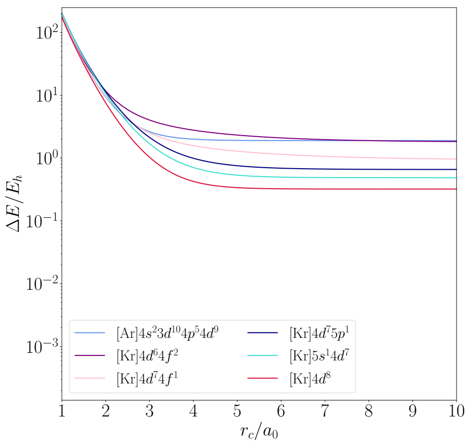

(a) PW92

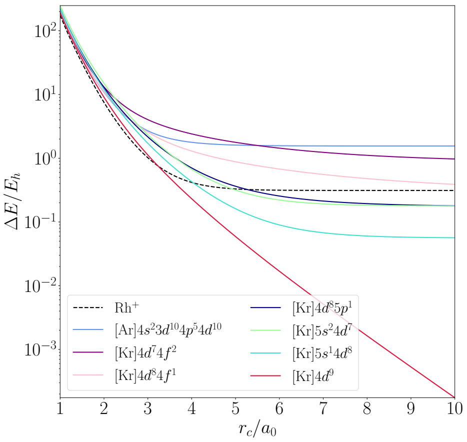

(b) PBE

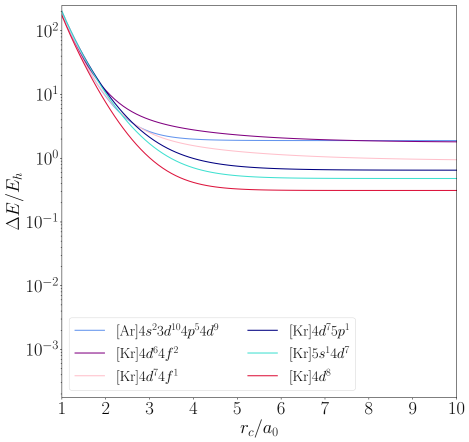

(b) PBE

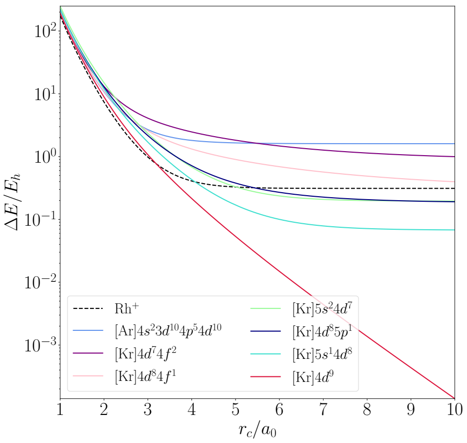

(c) r^{2}SCAN

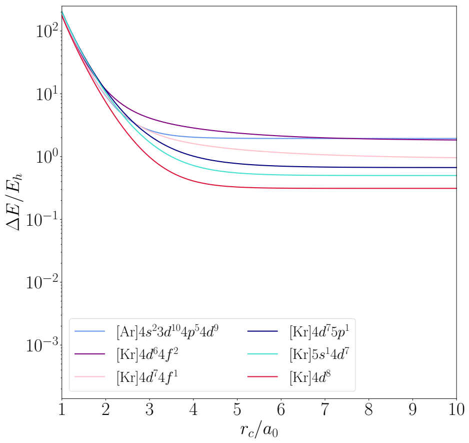

(c) r^{2}SCAN

Figure S95: Energies of various low lying configurations of hard-wall confined spin-restricted Rh shown as the energy difference from unconfined Rh as a function of the confinement radius $r_\infty = 1.0, 1.1, \ldots, 10.0a_0$. Note semilogarithmic scale.

Figure S96: Energies of various low lying configurations of the hard-wall confined spin-restricted monocation of Rh shown as the energy difference from unconfined Rh as a function of the confinement radius $r_\infty = 1.0, 1.1, \ldots, 10.0a_0$. Note semilogarithmic scale.

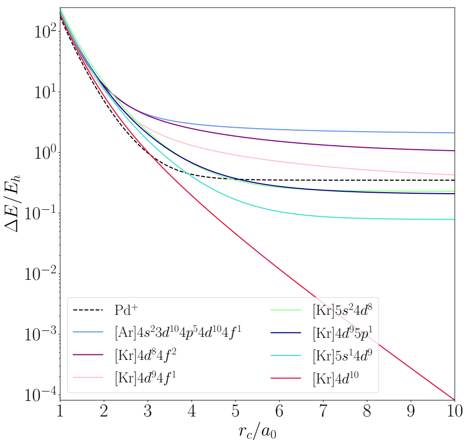

(a) PW92

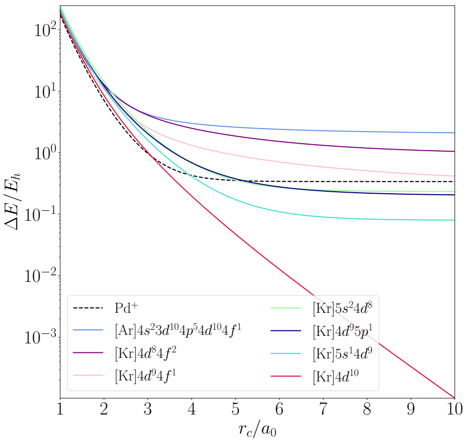

(b) PBE

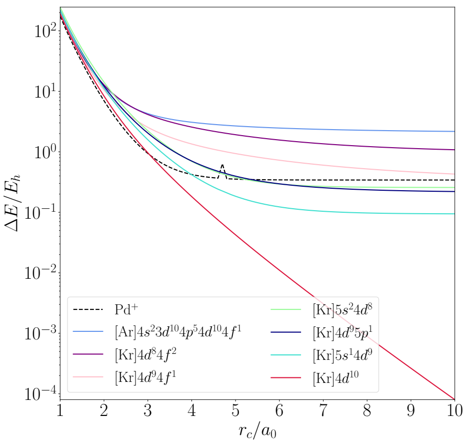

(c) r^{2}SCAN

Figure S97: Energies of various low lying configurations of hard-wall confined spin-restricted Pd shown as the energy difference from unconfined Pd as a function of the confinement radius $r_\infty = 1.0, 1.1, \ldots, 10.0a_0$. Note semilogarithmic scale.

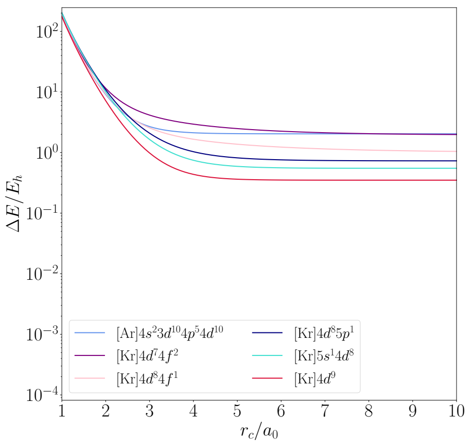

(a) PW92

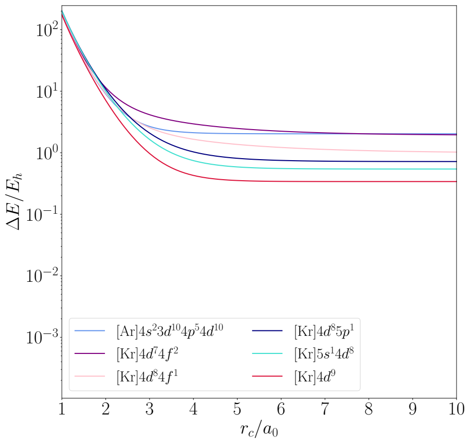

(b) PBE

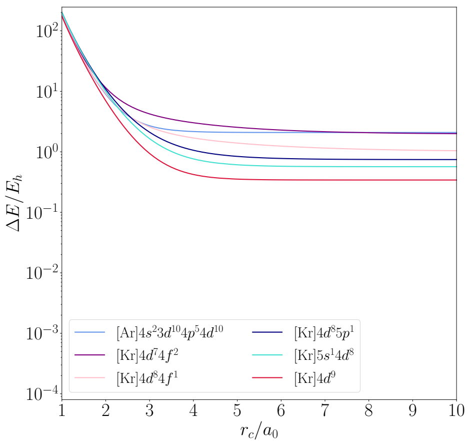

(c) r^{2}SCAN

Figure S98: Energies of various low lying configurations of the hard-wall confined spin-restricted monocation of Pd shown as the energy difference from unconfined Pd as a function of the confinement radius $r_\infty = 1.0, 1.1, \ldots, 10.0a_0$. Note semilogarithmic scale.

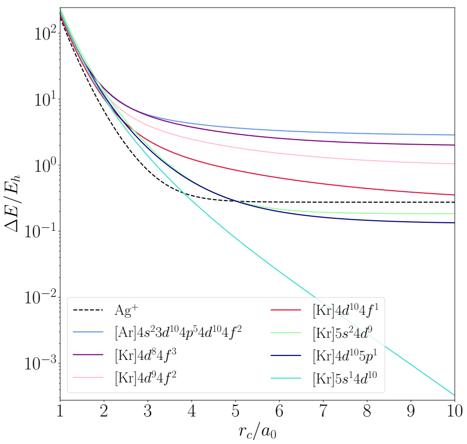

(a) PW92

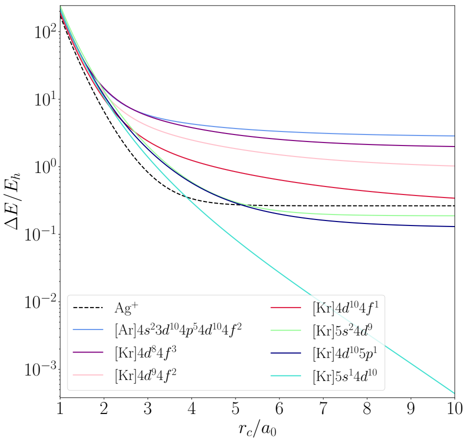

(b) PBE

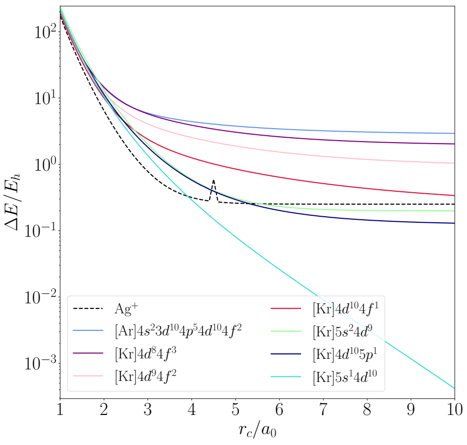

(c) r^{2}SCAN

Figure S99: Energies of various low lying configurations of hard-wall confined spin-restricted Ag shown as the energy difference from unconfined Ag as a function of the confinement radius $r_\infty = 1.0, 1.1, \ldots, 10.0a_0$. Note semilogarithmic scale.

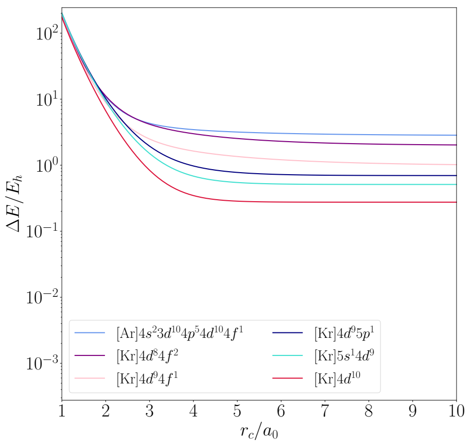

(a) PW92

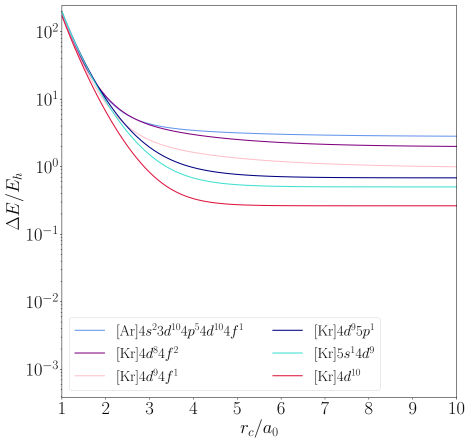

(b) PBE

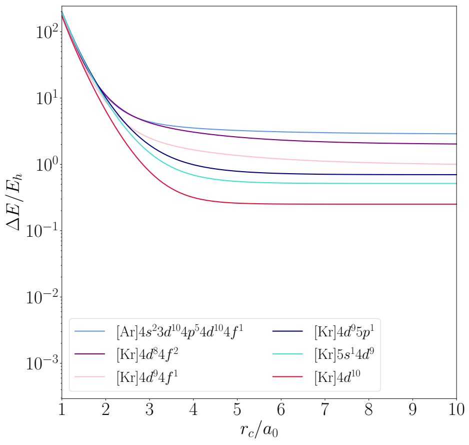

(c) r^{2}SCAN

Figure S100: Energies of various low lying configurations of the hard-wall confined spin-restricted monocation of Ag shown as the energy difference from unconfined Ag as a function of the confinement radius $r_\infty = 1.0, 1.1, \ldots, 10.0a_0$. Note semilogarithmic scale.

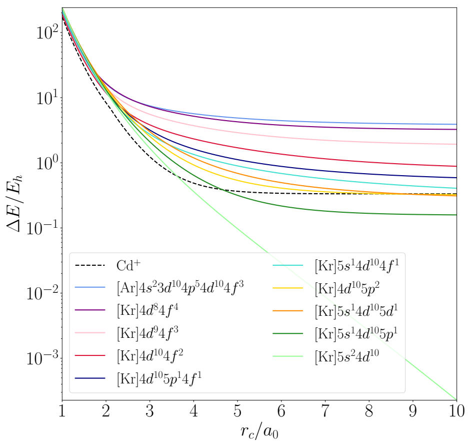

(a) PW92

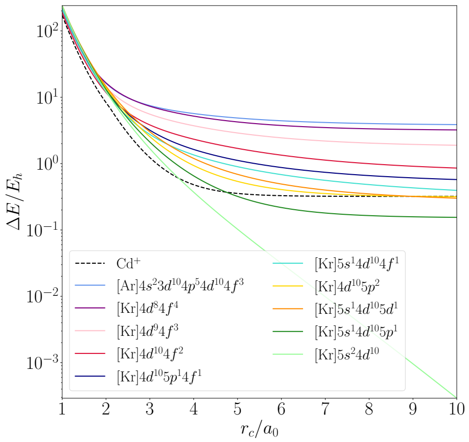

(b) PBE

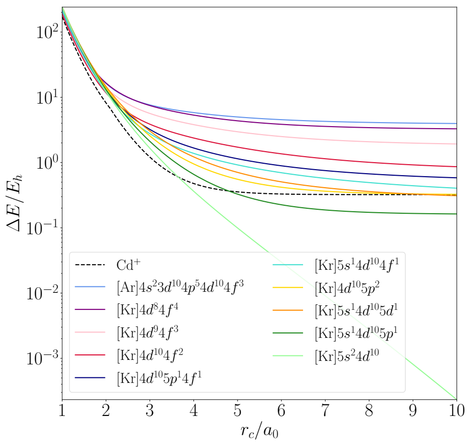

(c) r^{2}SCAN

Figure S101: Energies of various low lying configurations of hard-wall confined spin-restricted Cd shown as the energy difference from unconfined Cd as a function of the confinement radius $r_\infty = 1.0, 1.1, \ldots, 10.0 a_0$. Note semilogarithmic scale.

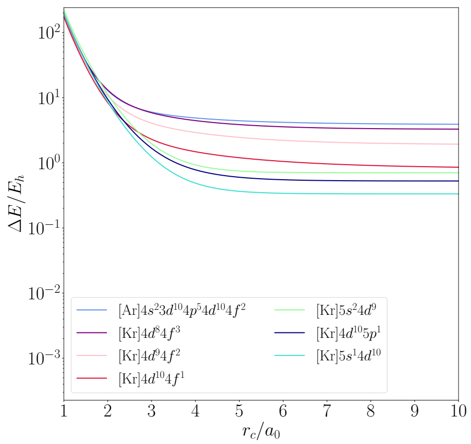

(a) PW92

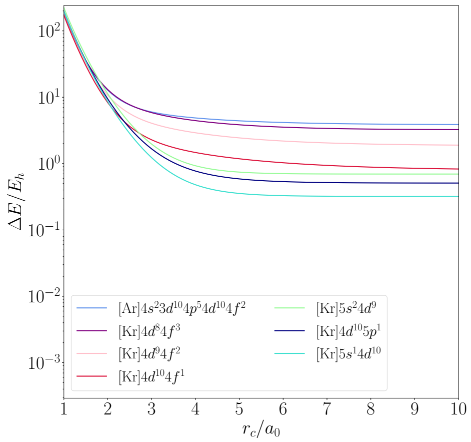

(b) PBE

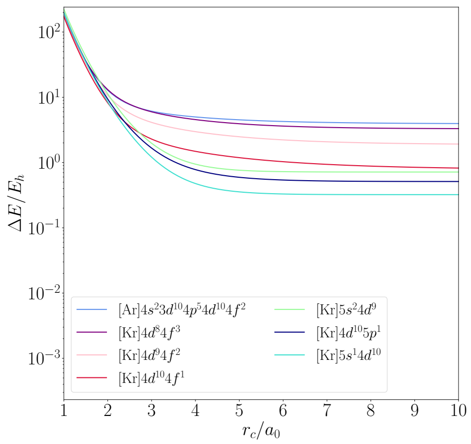

(c) r^{2}SCAN

Figure S102: Energies of various low lying configurations of the hard-wall confined spin-restricted monocation of Cd shown as the energy difference from unconfined Cd as a function of the confinement radius $r_\infty = 1.0, 1.1, \ldots, 10.0 a_0$. Note semilogarithmic scale.

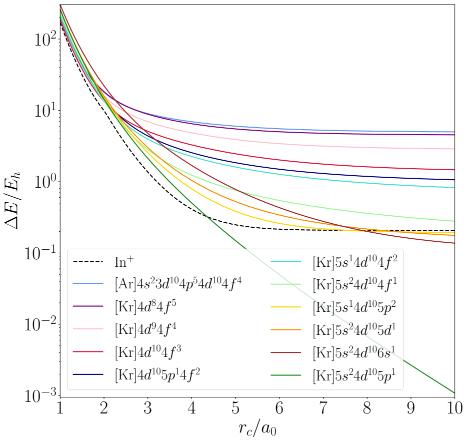

(a) PW92

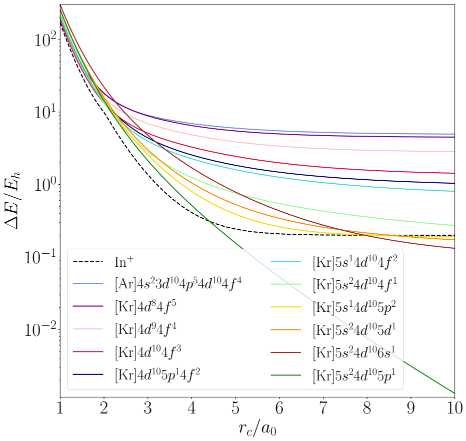

(b) PBE

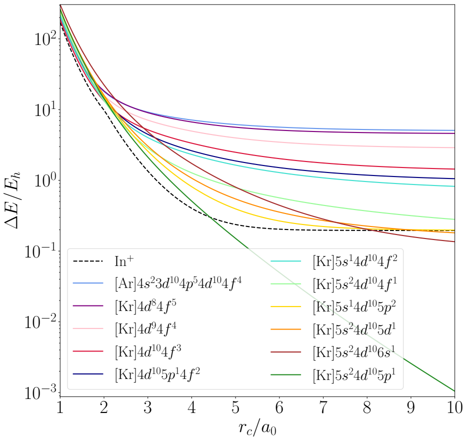

(c) r^{2}SCAN

Figure S103: Energies of various low lying configurations of hard-wall confined spin-restricted In shown as the energy difference from unconfined In as a function of the confinement radius $r_\infty = 1.0, 1.1, \ldots, 10.0 a_0$. Note semilogarithmic scale.

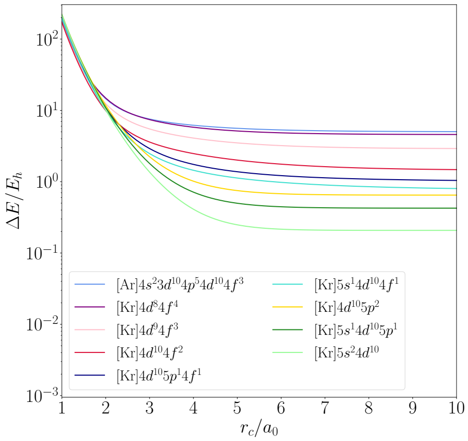

(a) PW92

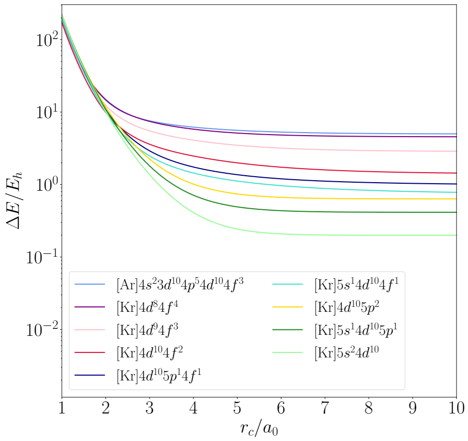

(b) PBE

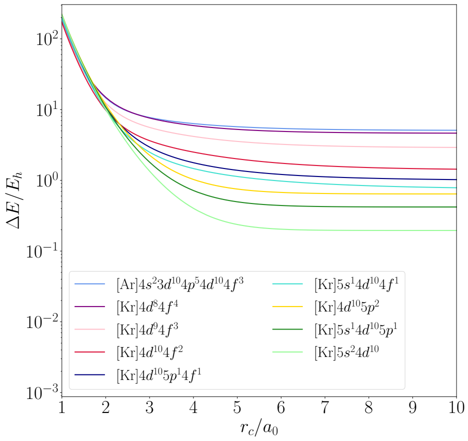

(c) r^{2}SCAN

Figure S104: Energies of various low lying configurations of the hard-wall confined spin-restricted monocation of In shown as the energy difference from unconfined In as a function of the confinement radius $r_\infty = 1.0, 1.1, \ldots, 10.0 a_0$. Note semilogarithmic scale.

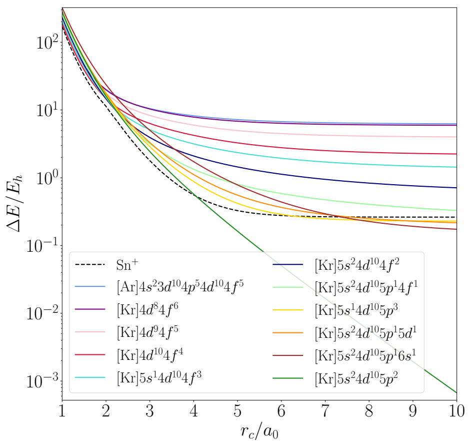

(a) PW92

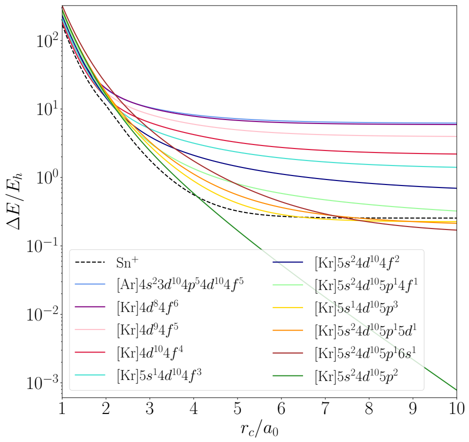

(b) PBE

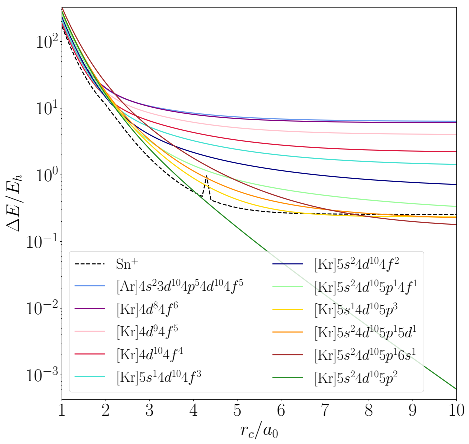

(c) r^{2}SCAN

Figure S105: Energies of various low lying configurations of hard-wall confined spin-restricted Sn shown as the energy difference from unconfined Sn as a function of the confinement radius $r_\infty = 1.0, 1.1, \ldots, 10.0 a_0$. Note semilogarithmic scale.

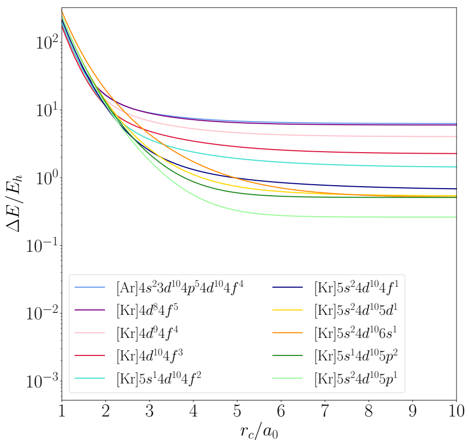

(a) PW92

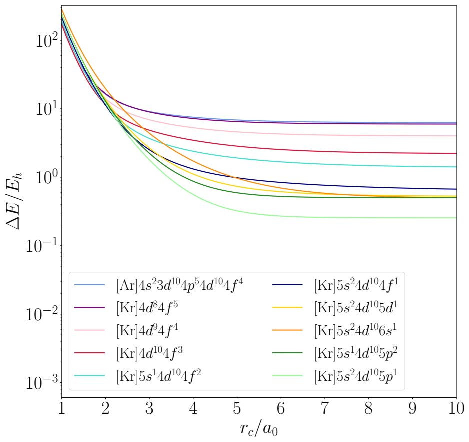

(b) PBE

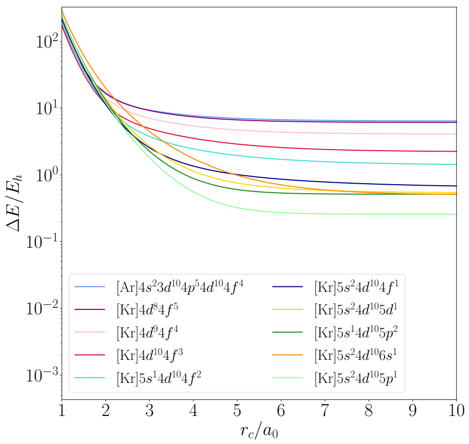

(c) r^{2}SCAN

Figure S106: Energies of various low lying configurations of the hard-wall confined spin-restricted monocation of Sn shown as the energy difference from unconfined Sn as a function of the confinement radius $r_\infty = 1.0, 1.1, \ldots, 10.0 a_0$. Note semilogarithmic scale.

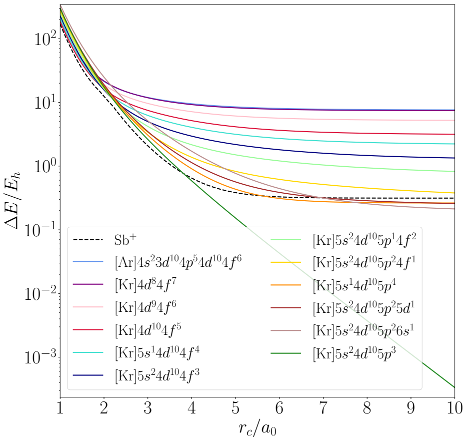

(a) PW92

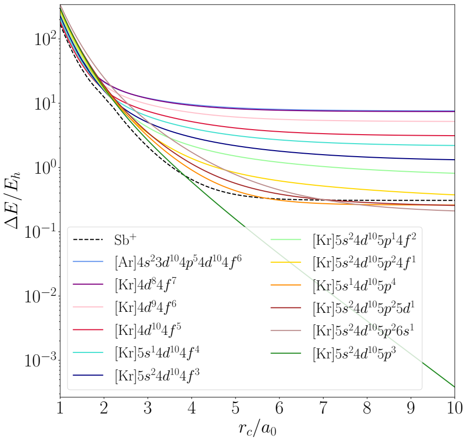

(b) PBE

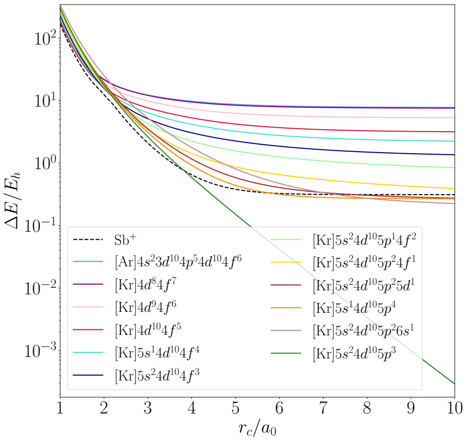

(c) r^2SCAN

Figure S107: Energies of various low lying configurations of hard-wall confined spin-restricted Sb shown as the energy difference from unconfined Sb as a function of the confinement radius $r_\infty = 1.0, 1.1, \ldots, 10.0a_0$. Note semilogarithmic scale.

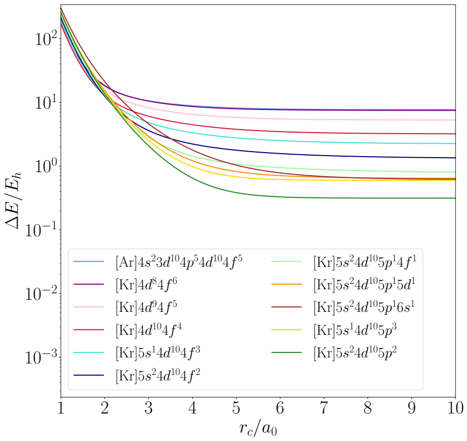

(a) PW92

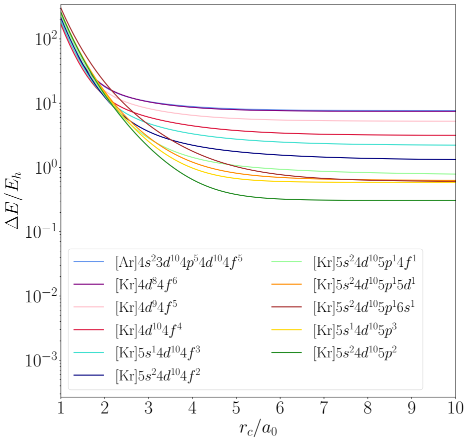

(b) PBE

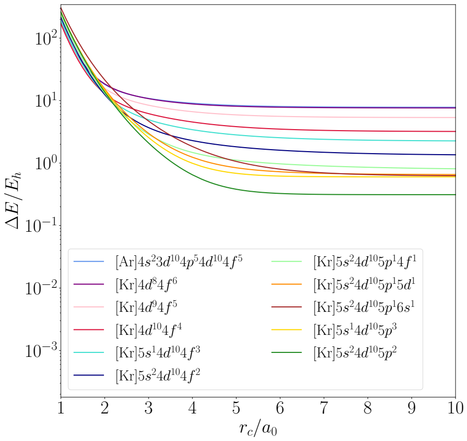

(c) r^2SCAN

Figure S108: Energies of various low lying configurations of the hard-wall confined spin-restricted monocation of Sb shown as the energy difference from unconfined Sb as a function of the confinement radius $r_\infty = 1.0, 1.1, \ldots, 10.0a_0$. Note semilogarithmic scale.

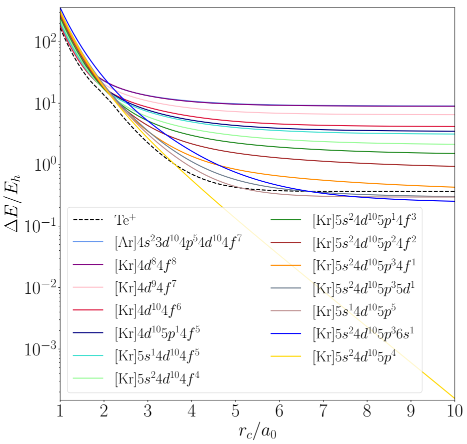

(a) PW92

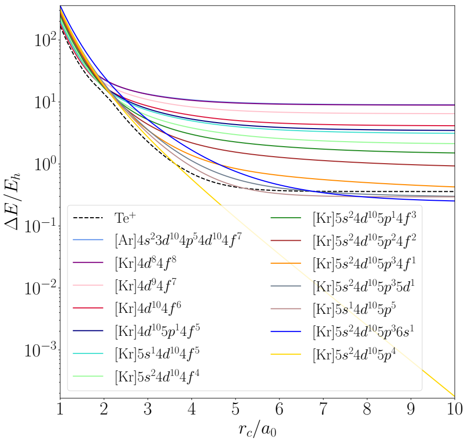

(b) PBE

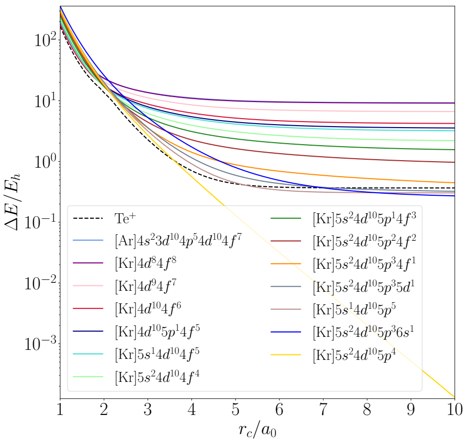

(c) r^{2}SCAN

Figure S109: Energies of various low lying configurations of hard-wall confined spin-restricted Te shown as the energy difference from unconfined Te as a function of the confinement radius $r_\infty = 1.0, 1.1, \ldots, 10.0 a_0$. Note semilogarithmic scale.

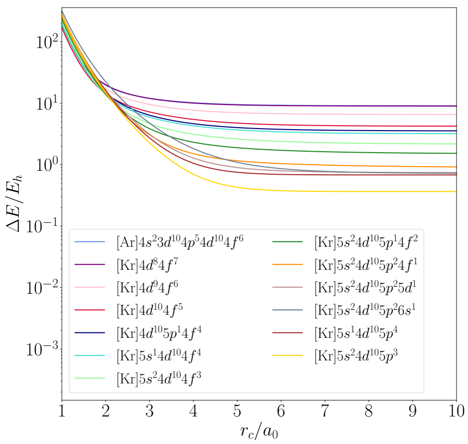

(a) PW92

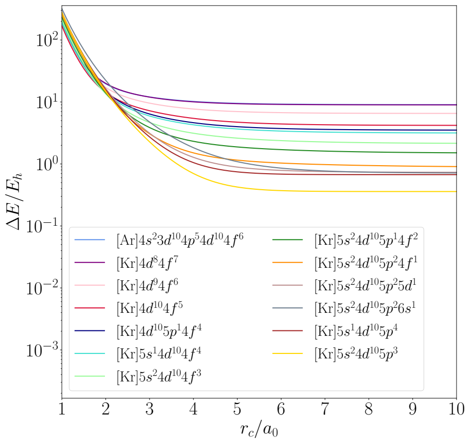

(b) PBE

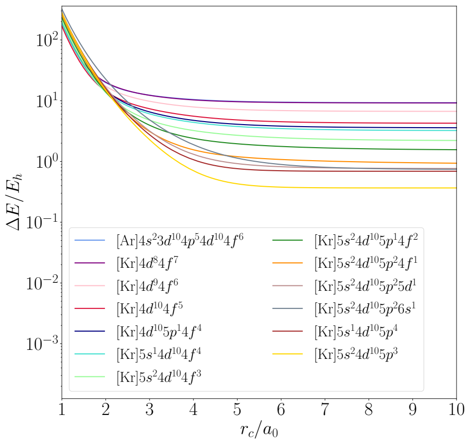

(c) r^{2}SCAN

Figure S110: Energies of various low lying configurations of the hard-wall confined spin-restricted monocation of Te shown as the energy difference from unconfined Te as a function of the confinement radius $r_\infty = 1.0, 1.1, \ldots, 10.0 a_0$. Note semilogarithmic scale.

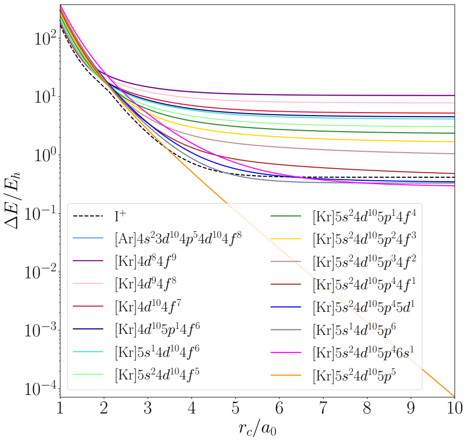

(a) PW92

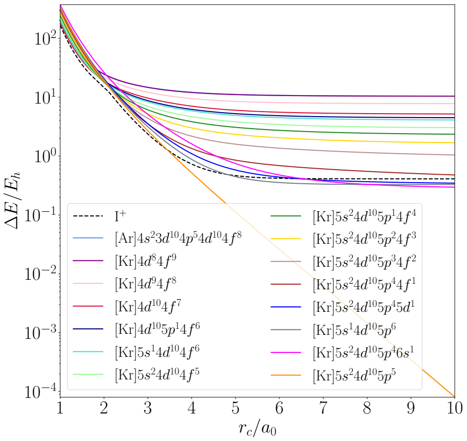

(b) PBE

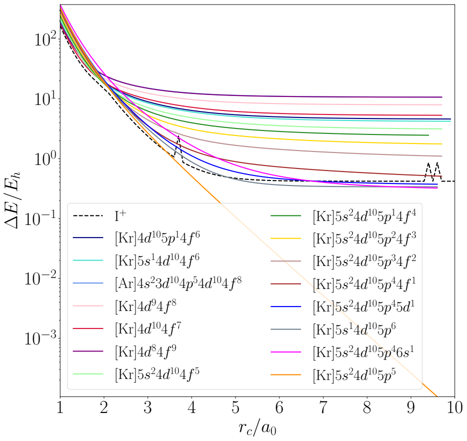

(c) r^{2}SCAN

Figure S111: Energies of various low lying configurations of hard-wall confined spin-restricted I shown as the energy difference from unconfined I as a function of the confinement radius $r_\infty = 1.0, 1.1, \ldots, 10.0 a_0$. Note semilogarithmic scale.

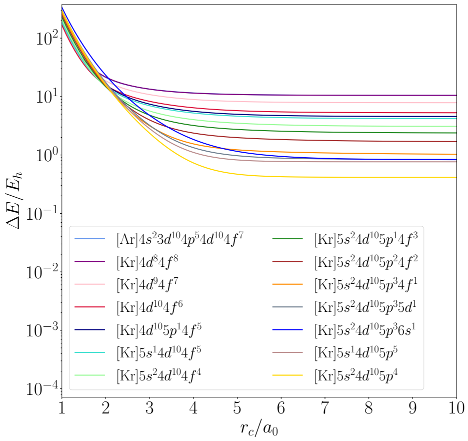

(a) PW92

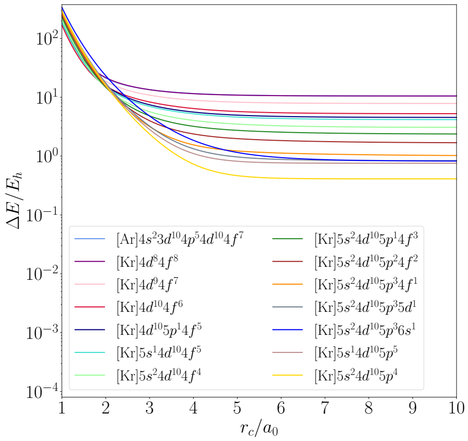

(b) PBE

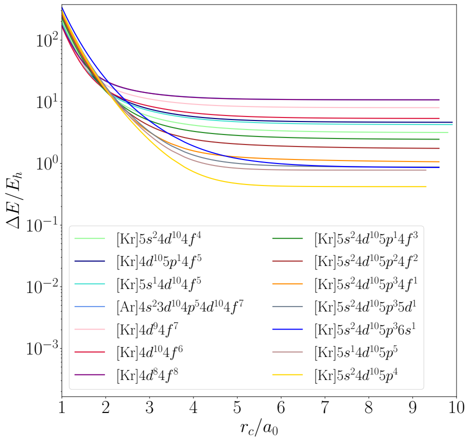

(c) r^{2}SCAN

Figure S112: Energies of various low lying configurations of the hard-wall confined spin-restricted monocation of I shown as the energy difference from unconfined I as a function of the confinement radius $r_\infty = 1.0, 1.1, \ldots, 10.0 a_0$. Note semilogarithmic scale.

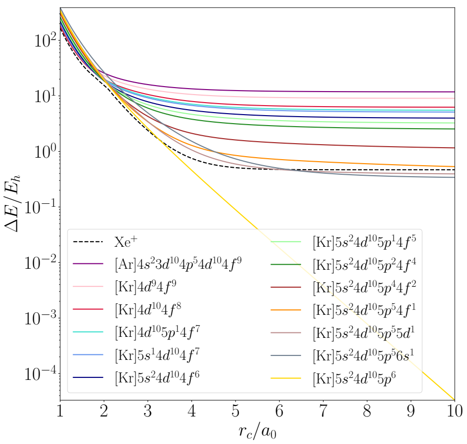

(a) PW92

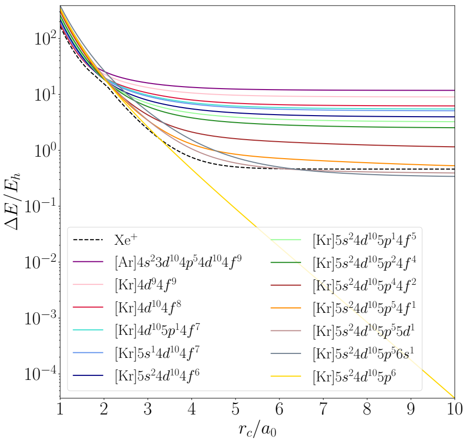

(b) PBE

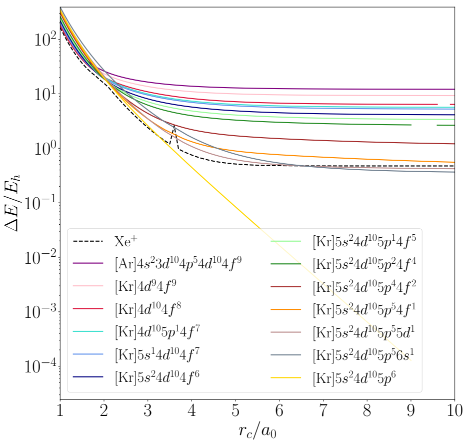

(c) r^{2}SCAN

Figure S113: Energies of various low lying configurations of hard-wall confined spin-restricted Xe shown as the energy difference from unconfined Xe as a function of the confinement radius $r_\infty = 1.0, 1.1, \ldots, 10.0a_0$. Note semilogarithmic scale.

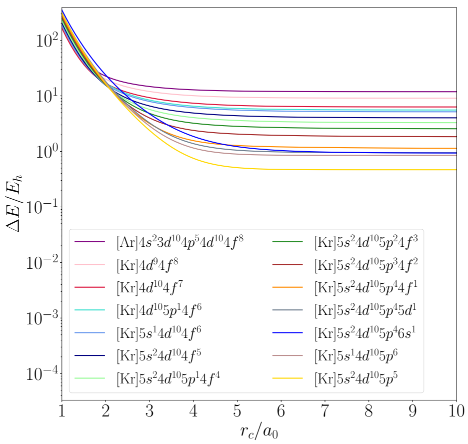

(a) PW92

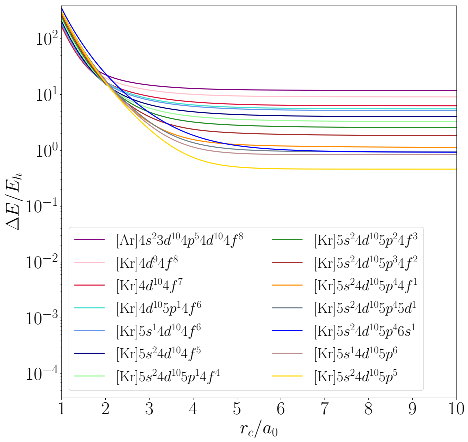

(b) PBE

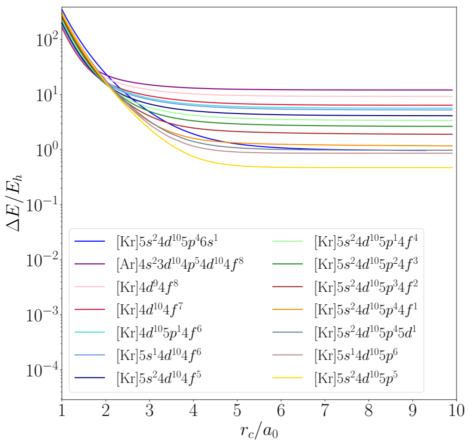

(c) r^{2}SCAN

Figure S114: Energies of various low lying configurations of the hard-wall confined spin-restricted monocation of Xe shown as the energy difference from unconfined Xe as a function of the confinement radius $r_\infty = 1.0, 1.1, \ldots, 10.0a_0$. Note semilogarithmic scale.

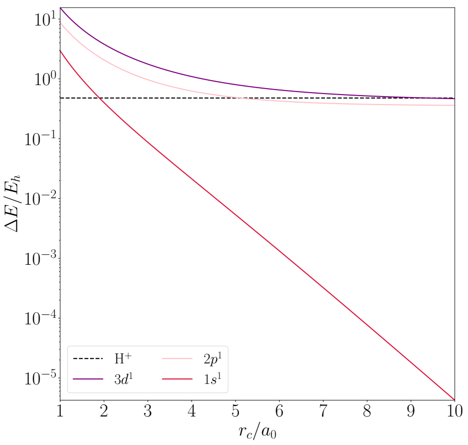

(a) PW92

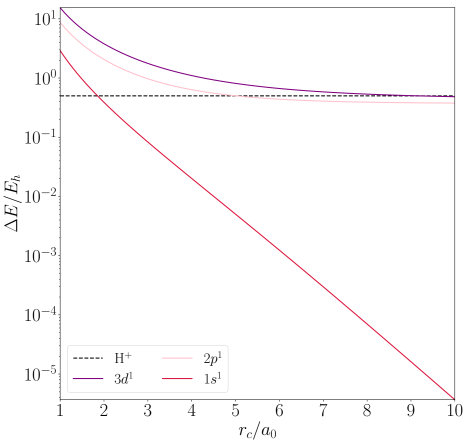

(b) PBE

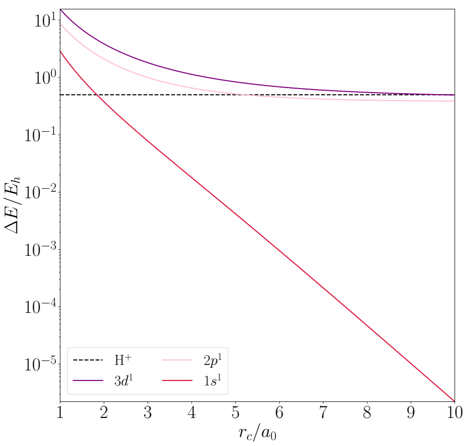

(c) r^{2}SCAN

Figure S115: Energies of various low lying configurations of hard-wall confined spin-polarized H shown as the energy difference from unconfined H as a function of the confinement radius $r_\infty = 1.0, 1.1, \ldots, 10.0a_0$. Note semilogarithmic scale.

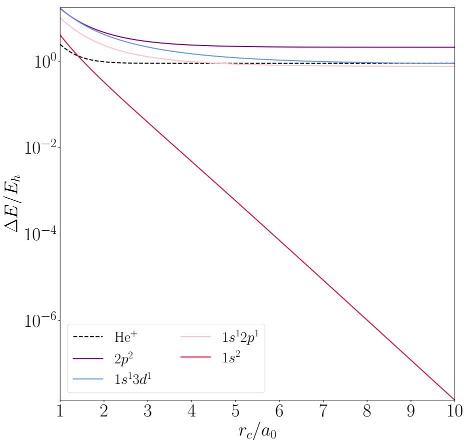

(a) PW92

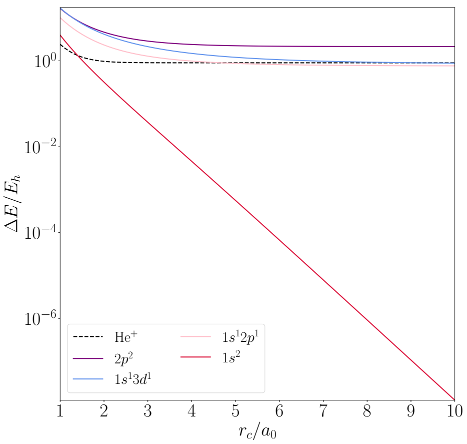

(b) PBE

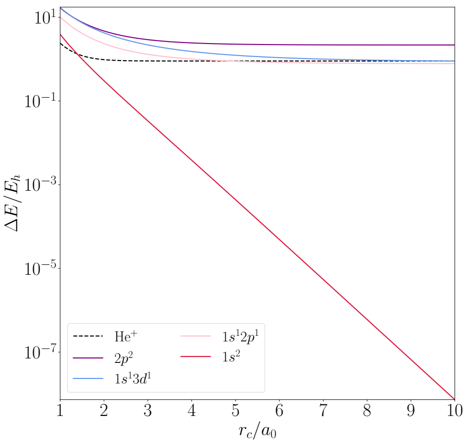

(c) r^{2}SCAN

Figure S116: Energies of various low lying configurations of hard-wall confined spin-polarized He shown as the energy difference from unconfined He as a function of the confinement radius $r_\infty = 1.0, 1.1, \ldots, 10.0 a_0$. Note semilogarithmic scale.

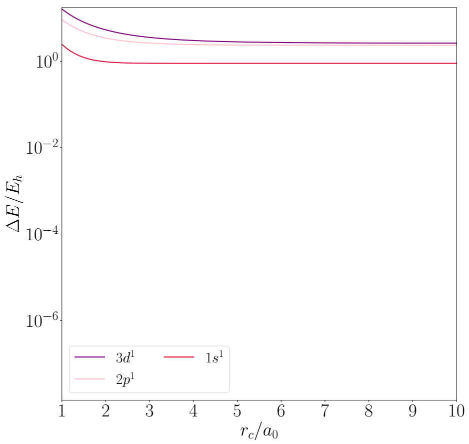

(a) PW92

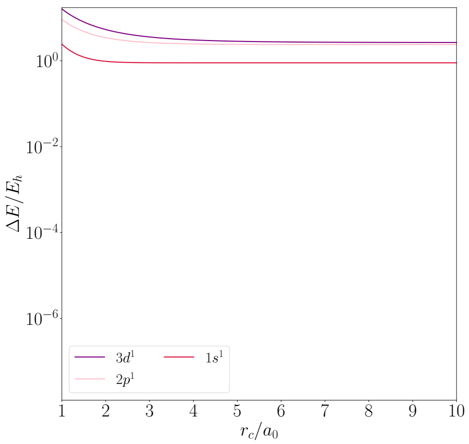

(b) PBE

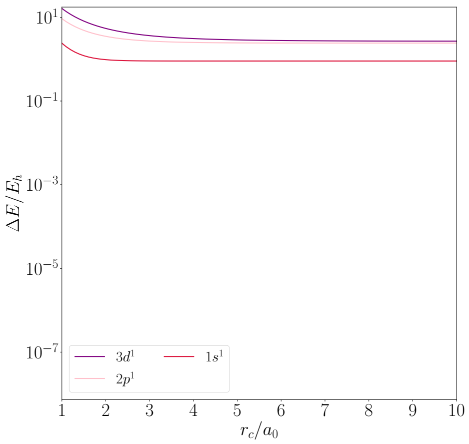

(c) r^{2}SCAN

Figure S117: Energies of various low lying configurations of the hard-wall confined spin-polarized monocation of He shown as the energy difference from unconfined He as a function of the confinement radius $r_\infty = 1.0, 1.1, \ldots, 10.0 a_0$. Note semilogarithmic scale.

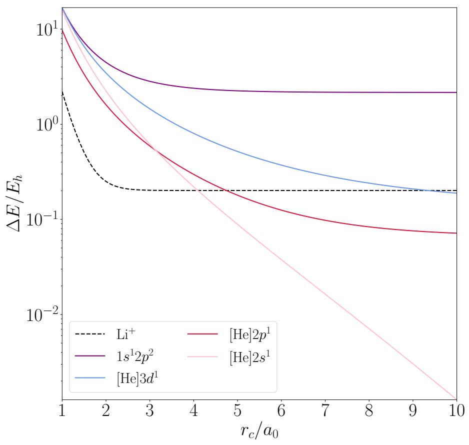

(a) PW92

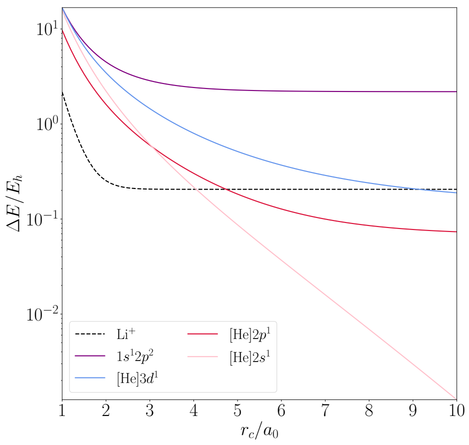

(b) PBE

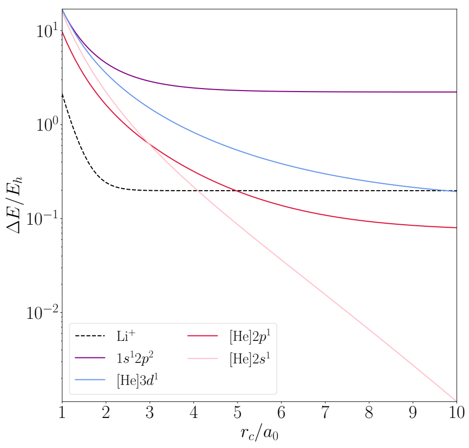

(c) r^{2}SCAN

Figure S118: Energies of various low lying configurations of hard-wall confined spin-polarized Li shown as the energy difference from unconfined Li as a function of the confinement radius $r_\infty = 1.0, 1.1, \ldots, 10.0a_0$. Note semilogarithmic scale.

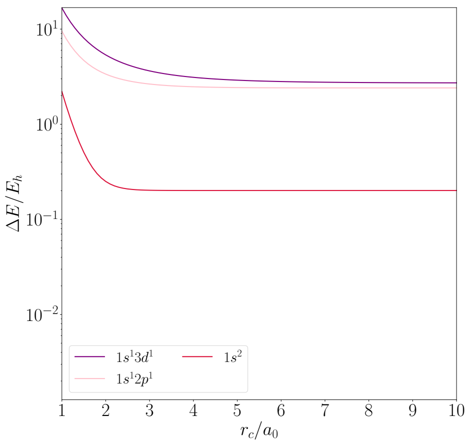

(a) PW92

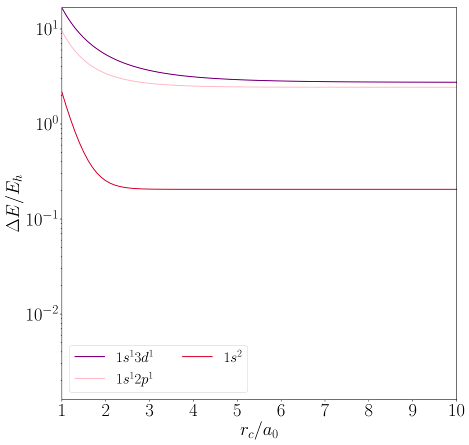

(b) PBE

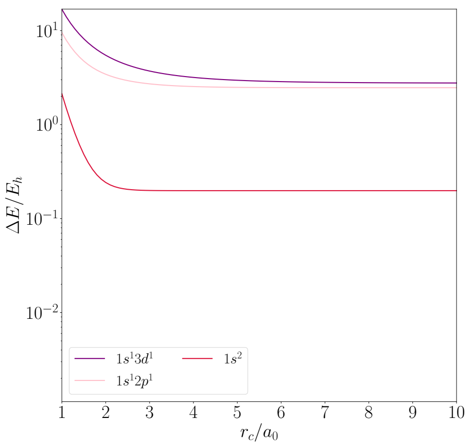

(c) r^{2}SCAN

Figure S119: Energies of various low lying configurations of the hard-wall confined spin-polarized monocation of Li shown as the energy difference from unconfined Li as a function of the confinement radius $r_\infty = 1.0, 1.1, \ldots, 10.0a_0$. Note semilogarithmic scale.

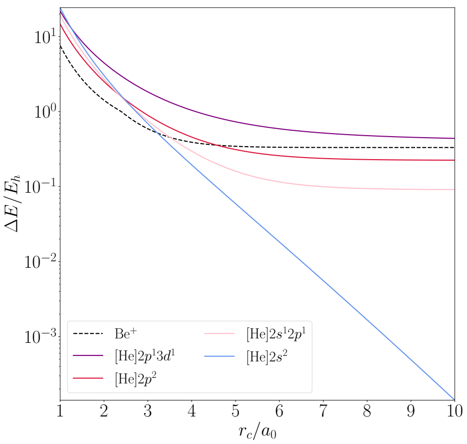

(a) PW92

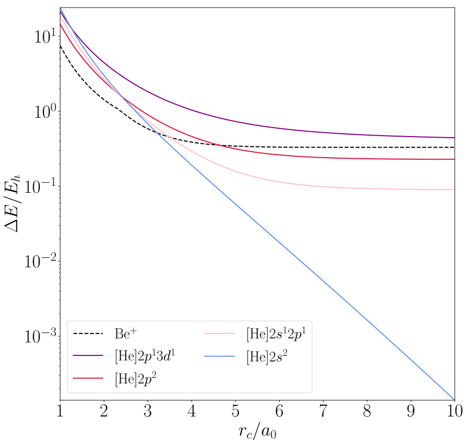

(b) PBE

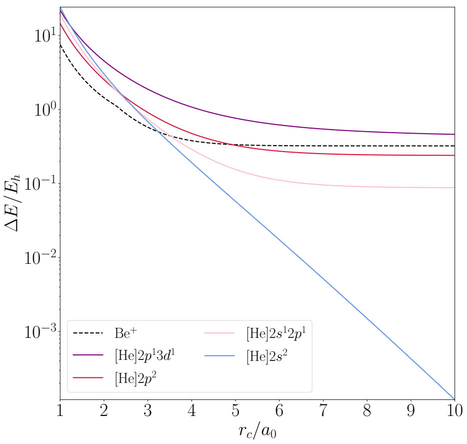

(c) r^{2}SCAN

Figure S120: Energies of various low lying configurations of hard-wall confined spin-polarized Be shown as the energy difference from unconfined Be as a function of the confinement radius $r_\infty = 1.0, 1.1, \ldots, 10.0a_0$. Note semilogarithmic scale.

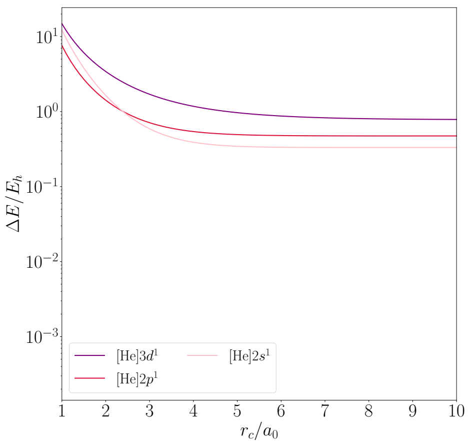

(a) PW92

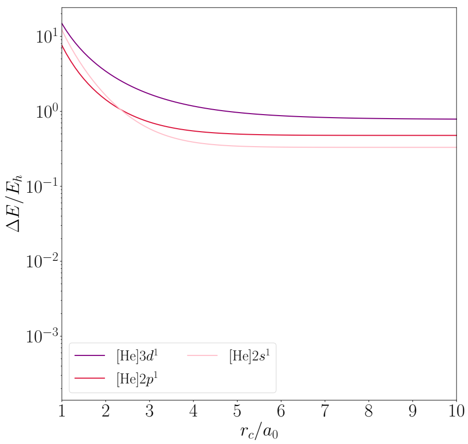

(b) PBE

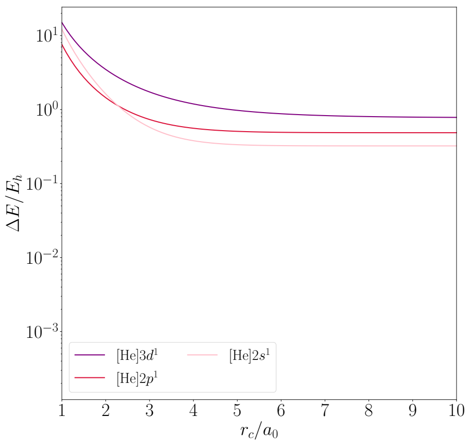

(c) r^{2}SCAN

Figure S121: Energies of various low lying configurations of the hard-wall confined spin-polarized monocation of Be shown as the energy difference from unconfined Be as a function of the confinement radius $r_\infty = 1.0, 1.1, \ldots, 10.0a_0$. Note semilogarithmic scale.

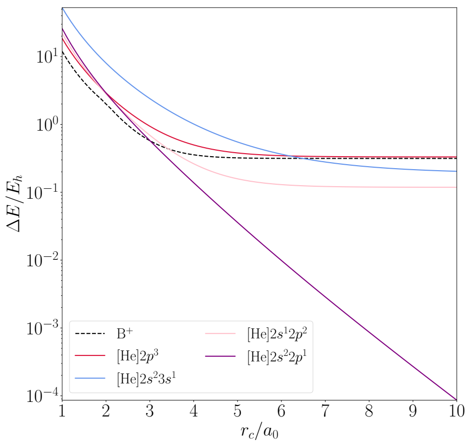

(a) PW92

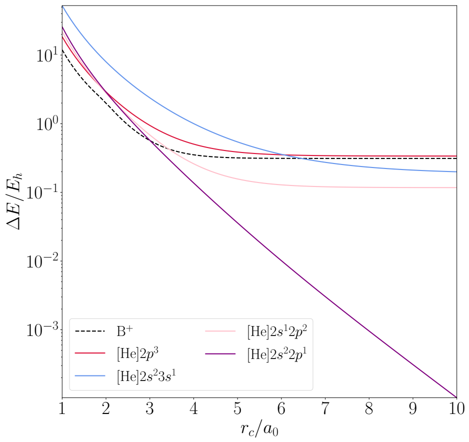

(b) PBE

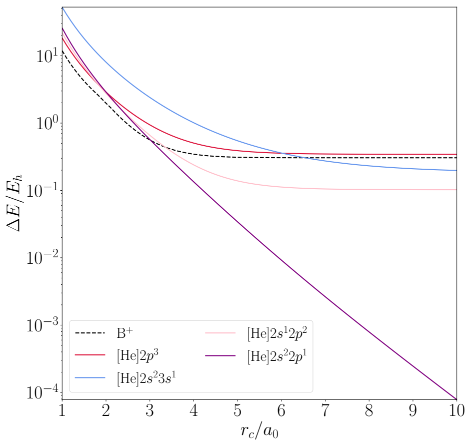

(c) r^{2}SCAN

Figure S122: Energies of various low lying configurations of hard-wall confined spin-polarized B shown as the energy difference from unconfined B as a function of the confinement radius $r_\infty = 1.0, 1.1, \ldots, 10.0 a_0$. Note semilogarithmic scale.

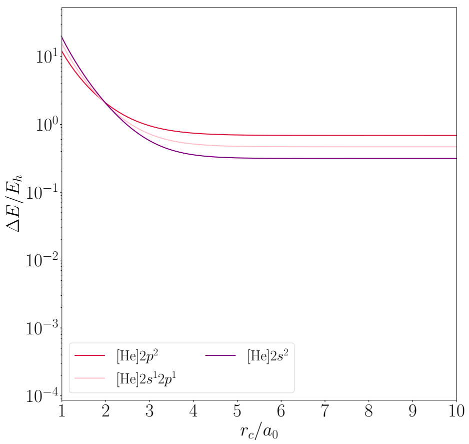

(a) PW92

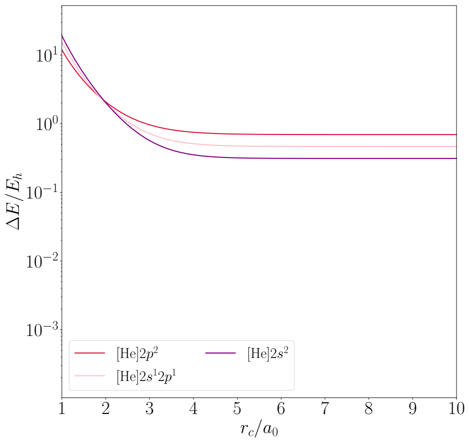

(b) PBE

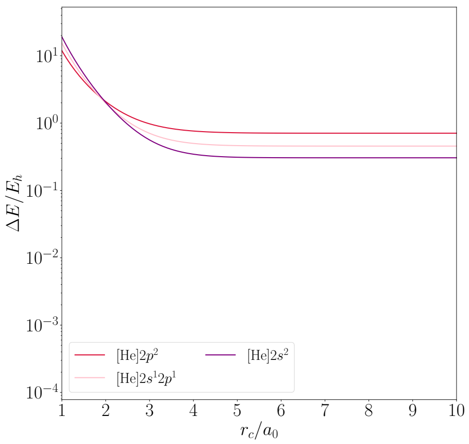

(c) r^{2}SCAN

Figure S123: Energies of various low lying configurations of the hard-wall confined spin-polarized monocation of B shown as the energy difference from unconfined B as a function of the confinement radius $r_\infty = 1.0, 1.1, \ldots, 10.0 a_0$. Note semilogarithmic scale.

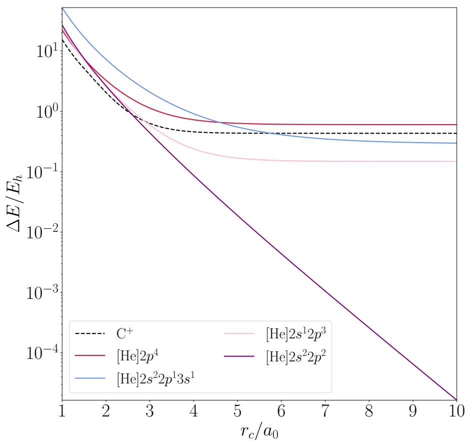

(a) PW92

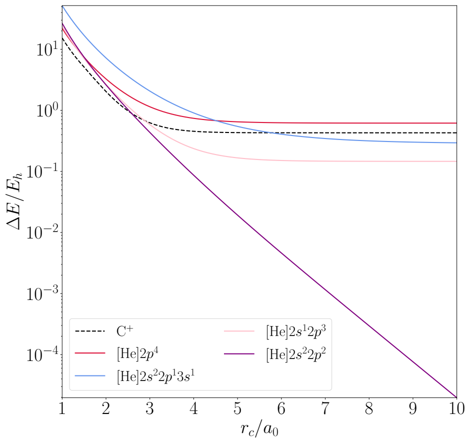

(b) PBE

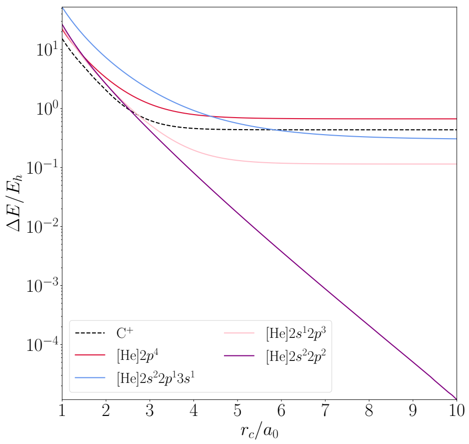

(c) r^{2}SCAN

Figure S124: Energies of various low lying configurations of hard-wall confined spin-polarized C shown as the energy difference from unconfined C as a function of the confinement radius $r_\infty = 1.0, 1.1, \ldots, 10.0 a_0$. Note semilogarithmic scale.

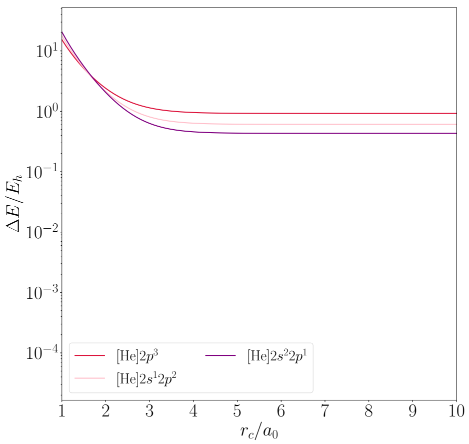

(a) PW92

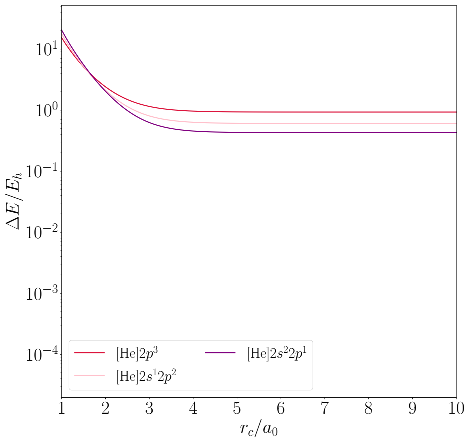

(b) PBE

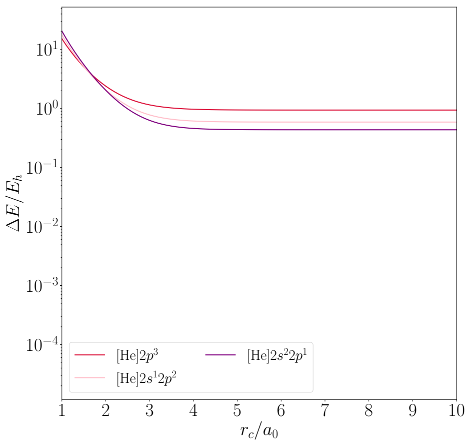

(c) r^{2}SCAN

Figure S125: Energies of various low lying configurations of the hard-wall confined spin-polarized monocation of C shown as the energy difference from unconfined C as a function of the confinement radius $r_\infty = 1.0, 1.1, \ldots, 10.0 a_0$. Note semilogarithmic scale.

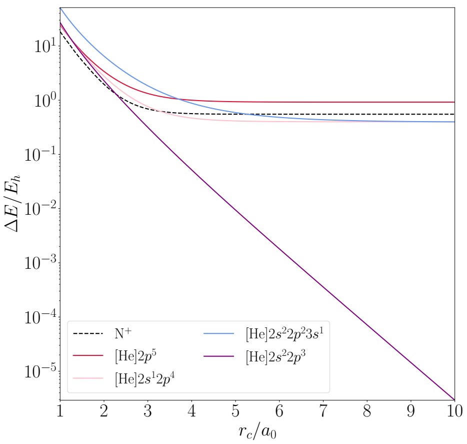

(a) PW92

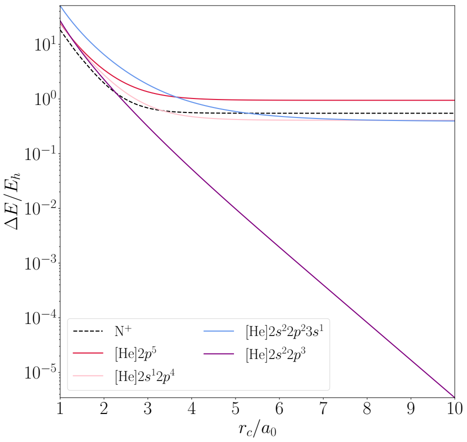

(b) PBE

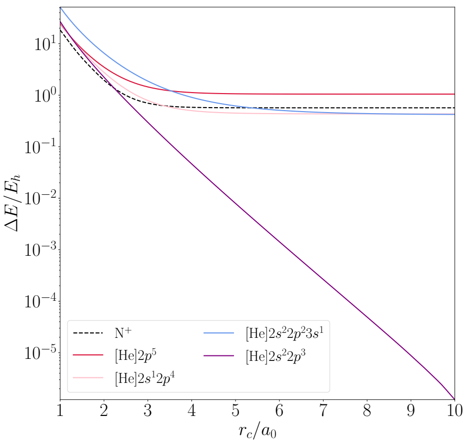

(c) r^{2}SCAN

Figure S126: Energies of various low lying configurations of hard-wall confined spin-polarized N shown as the energy difference from unconfined N as a function of the confinement radius $r_\infty = 1.0, 1.1, \ldots, 10.0a_0$. Note semilogarithmic scale.

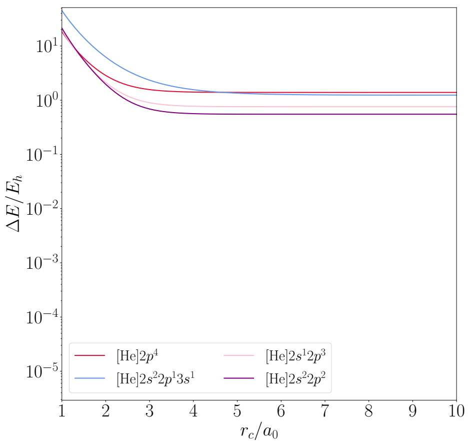

(a) PW92

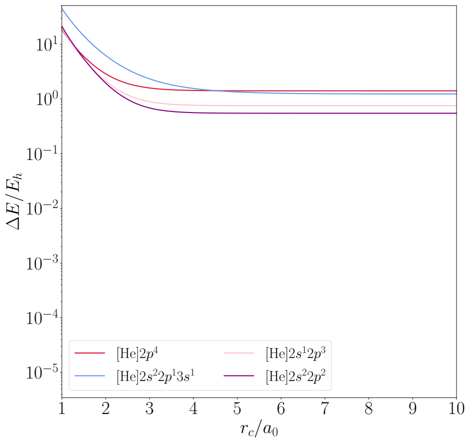

(b) PBE

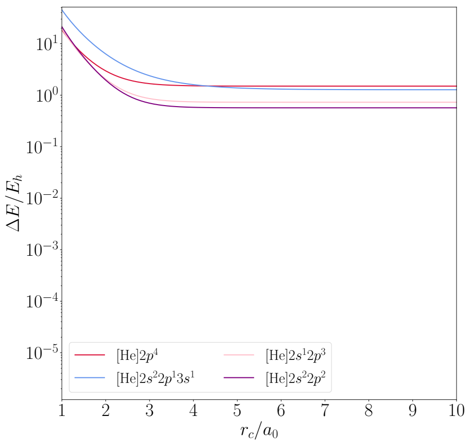

(c) r^{2}SCAN

Figure S127: Energies of various low lying configurations of the hard-wall confined spin-polarized monocation of N shown as the energy difference from unconfined N as a function of the confinement radius $r_\infty = 1.0, 1.1, \ldots, 10.0a_0$. Note semilogarithmic scale.

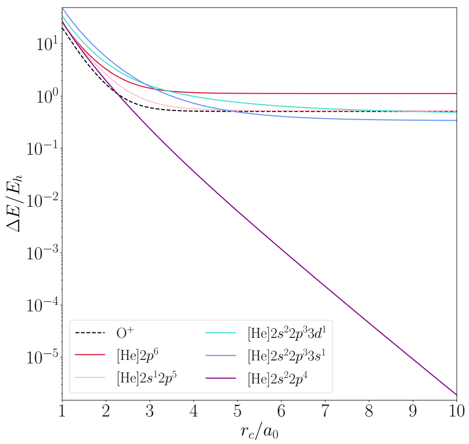

(a) PW92

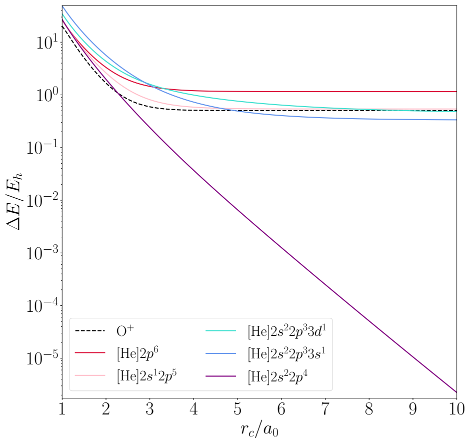

(b) PBE

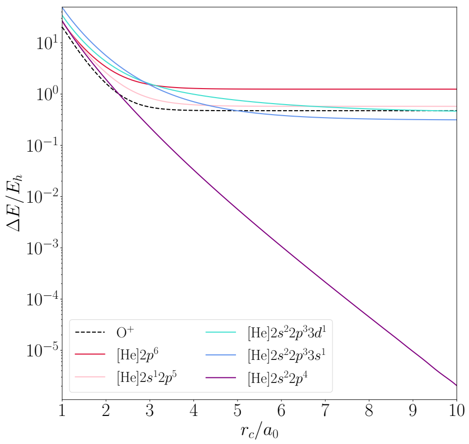

(c) r^{2}SCAN

Figure S128: Energies of various low lying configurations of hard-wall confined spin-polarized O shown as the energy difference from unconfined O as a function of the confinement radius $r_\infty = 1.0, 1.1, \ldots, 10.0a_0$. Note semilogarithmic scale.

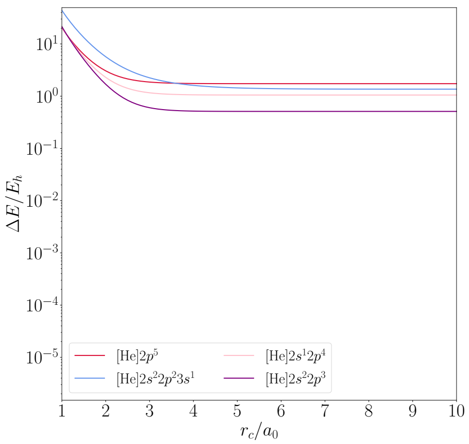

(a) PW92

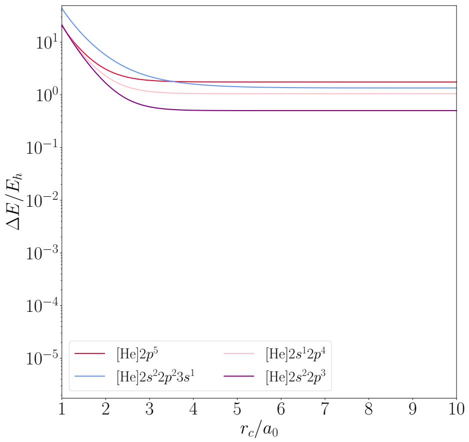

(b) PBE

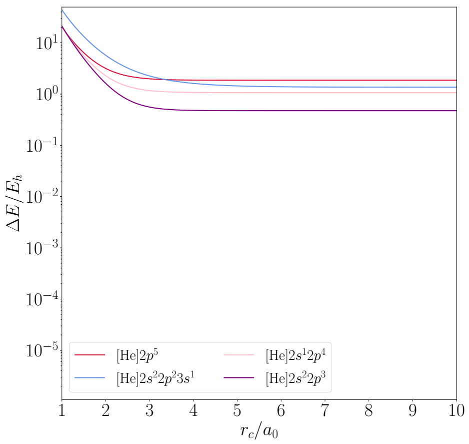

(c) r^{2}SCAN

Figure S129: Energies of various low lying configurations of the hard-wall confined spin-polarized monocation of O shown as the energy difference from unconfined O as a function of the confinement radius $r_\infty = 1.0, 1.1, \ldots, 10.0a_0$. Note semilogarithmic scale.

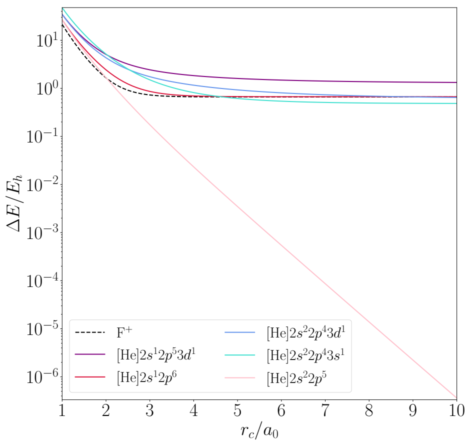

(a) PW92

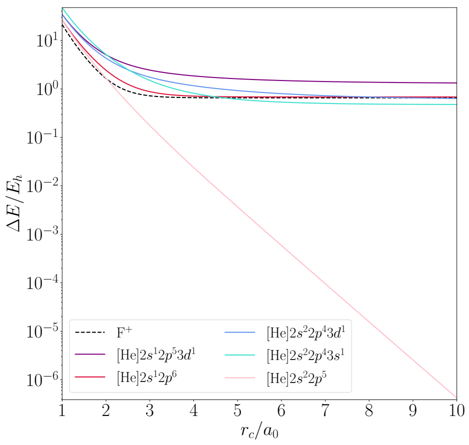

(b) PBE

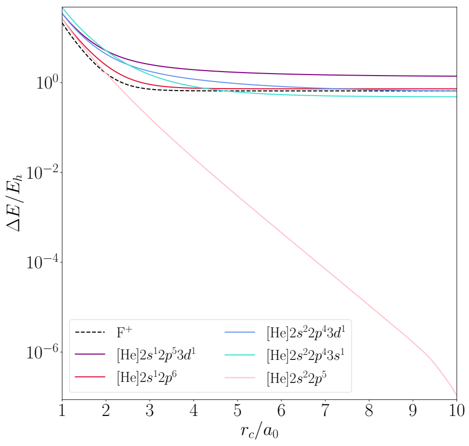

(c) r^{2}SCAN

Figure S130: Energies of various low lying configurations of hard-wall confined spin-polarized F shown as the energy difference from unconfined F as a function of the confinement radius $r_\infty = 1.0, 1.1, \ldots, 10.0 a_0$. Note semilogarithmic scale.

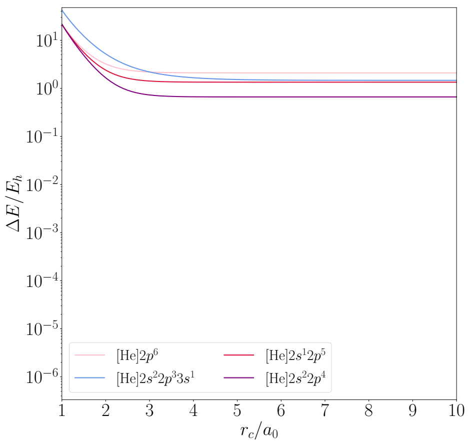

(a) PW92

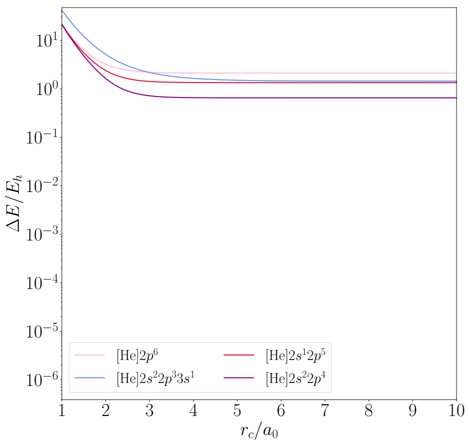

(b) PBE

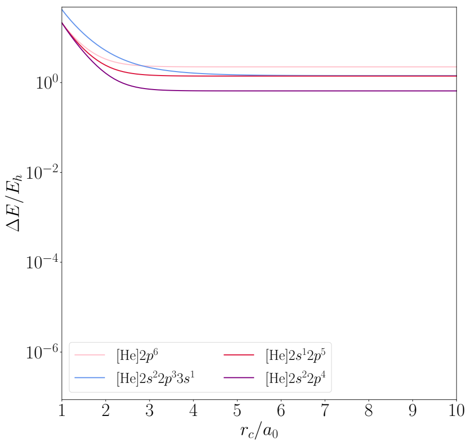

(c) r^{2}SCAN

Figure S131: Energies of various low lying configurations of the hard-wall confined spin-polarized monocation of F shown as the energy difference from unconfined F as a function of the confinement radius $r_\infty = 1.0, 1.1, \ldots, 10.0 a_0$. Note semilogarithmic scale.

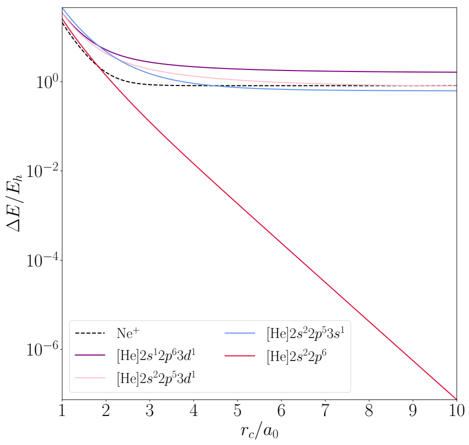

(a) PW92

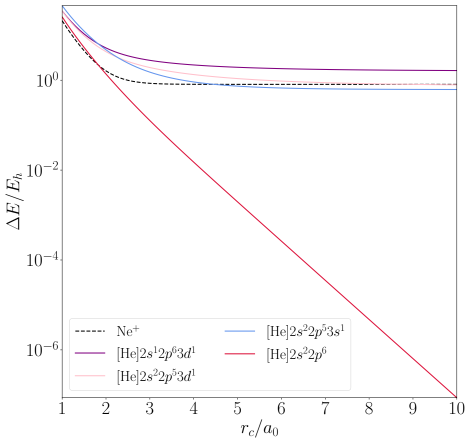

(b) PBE

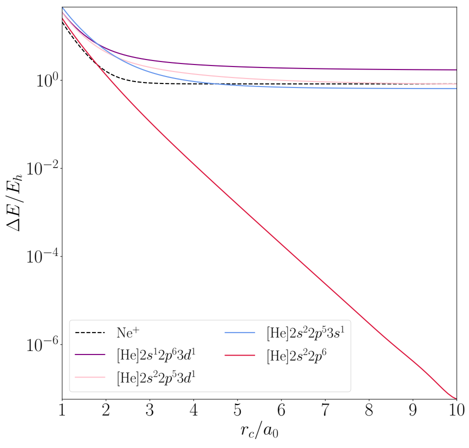

(c) r^{2}SCAN

Figure S132: Energies of various low lying configurations of hard-wall confined spin-polarized Ne shown as the energy difference from unconfined Ne as a function of the confinement radius $r_\infty = 1.0, 1.1, \ldots, 10.0 a_0$. Note semilogarithmic scale.

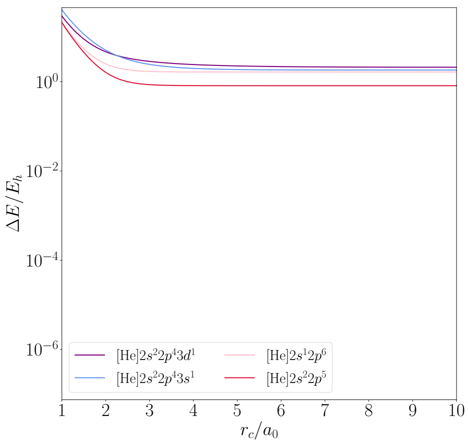

(a) PW92

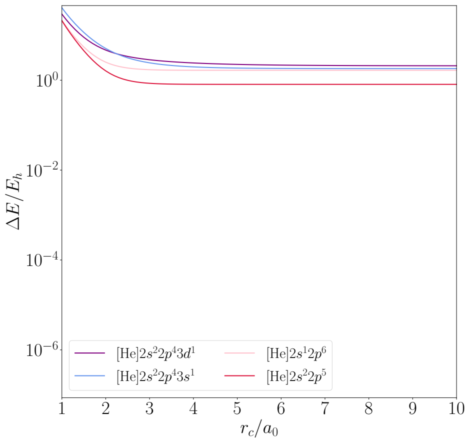

(b) PBE

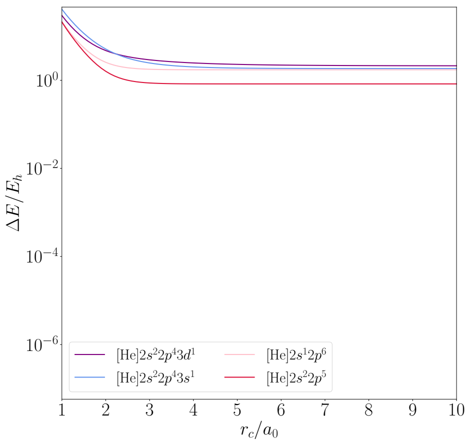

(c) r^{2}SCAN

Figure S133: Energies of various low lying configurations of the hard-wall confined spin-polarized monocation of Ne shown as the energy difference from unconfined Ne as a function of the confinement radius $r_\infty = 1.0, 1.1, \ldots, 10.0 a_0$. Note semilogarithmic scale.

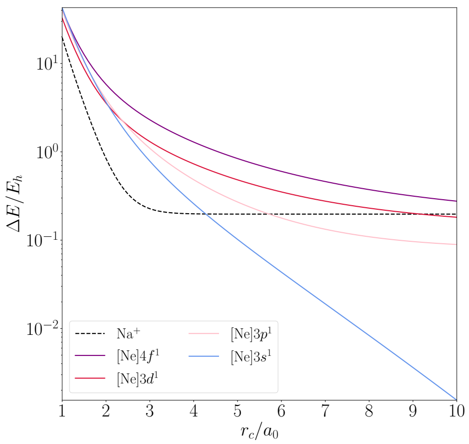

(a) PW92

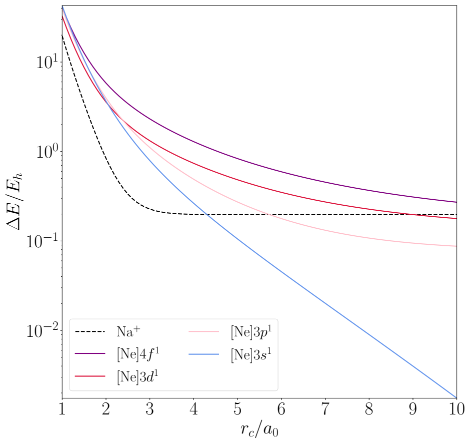

(b) PBE

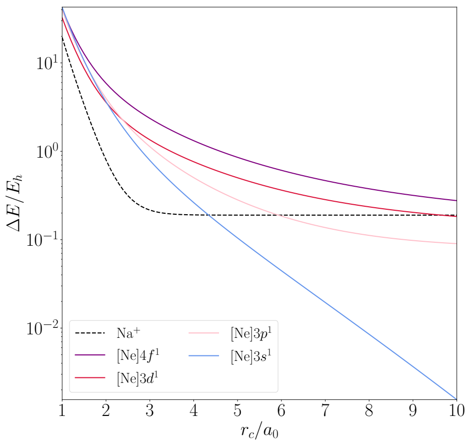

(c) r^{2}SCAN

Figure S134: Energies of various low lying configurations of hard-wall confined spin-polarized Na shown as the energy difference from unconfined Na as a function of the confinement radius $r_\infty = 1.0, 1.1, \ldots, 10.0a_0$. Note semilogarithmic scale.

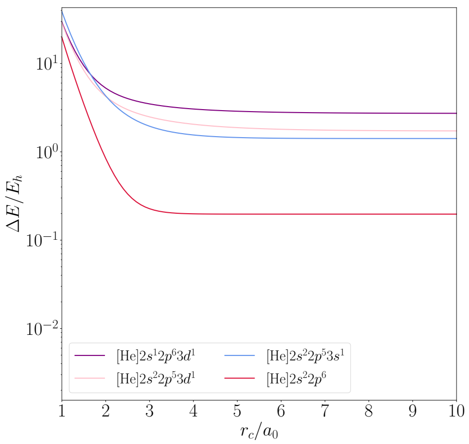

(a) PW92

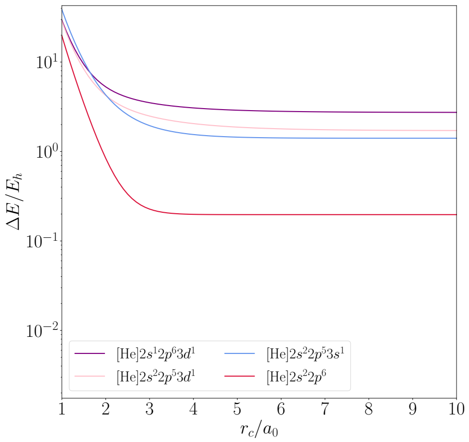

(b) PBE

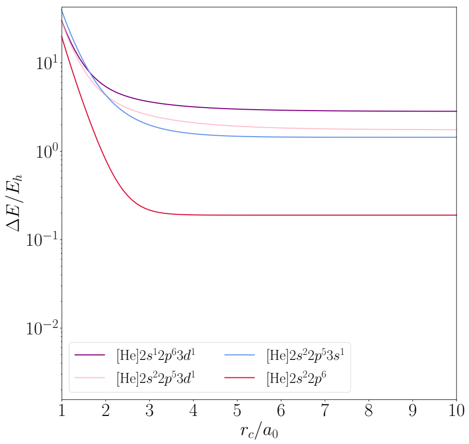

(c) r^{2}SCAN

Figure S135: Energies of various low lying configurations of the hard-wall confined spin-polarized monocation of Na shown as the energy difference from unconfined Na as a function of the confinement radius $r_\infty = 1.0, 1.1, \ldots, 10.0a_0$. Note semilogarithmic scale.

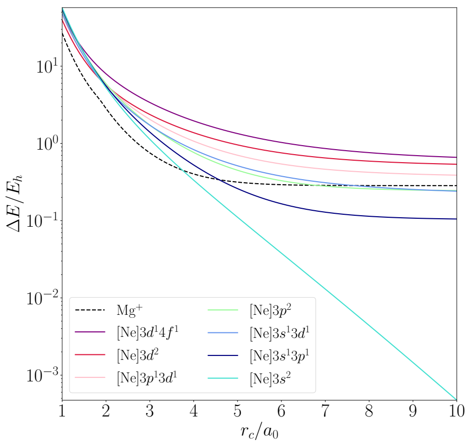

(a) PW92

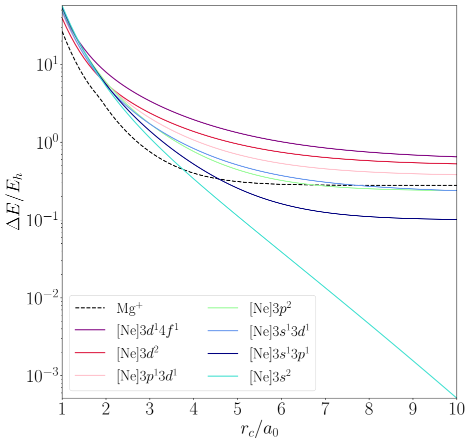

(b) PBE

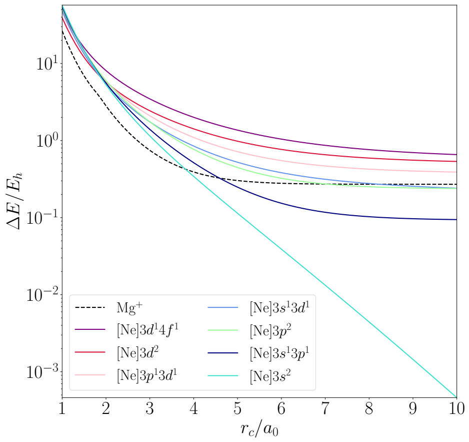

(c) r^{2}SCAN

Figure S136: Energies of various low lying configurations of hard-wall confined spin-polarized Mg shown as the energy difference from unconfined Mg as a function of the confinement radius $r_\infty = 1.0, 1.1, \ldots, 10.0a_0$. Note semilogarithmic scale.

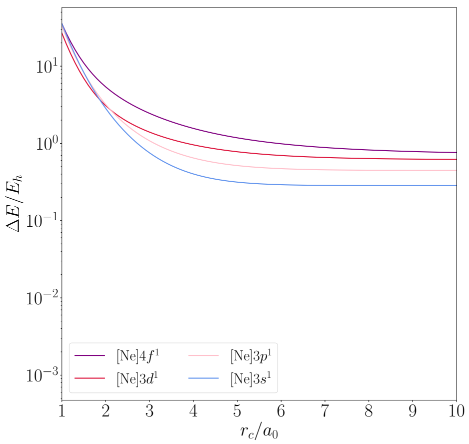

(a) PW92

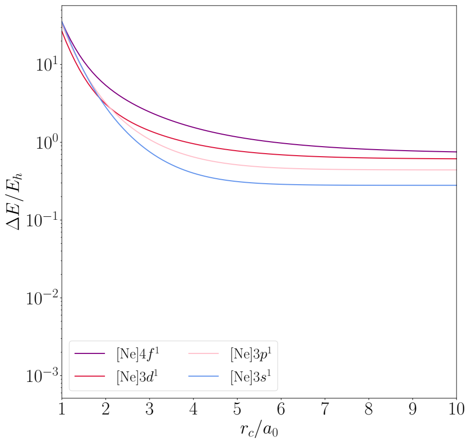

(b) PBE

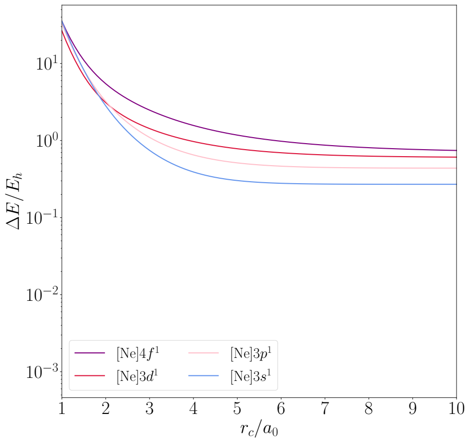

(c) r^{2}SCAN

Figure S137: Energies of various low lying configurations of the hard-wall confined spin-polarized monocation of Mg shown as the energy difference from unconfined Mg as a function of the confinement radius $r_\infty = 1.0, 1.1, \ldots, 10.0a_0$. Note semilogarithmic scale.

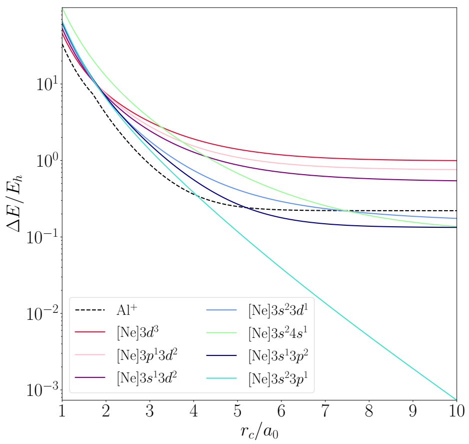

(a) PW92

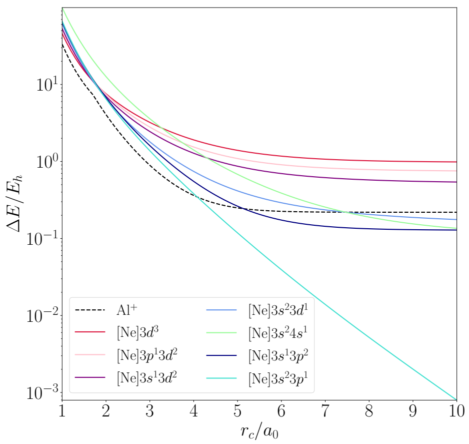

(b) PBE

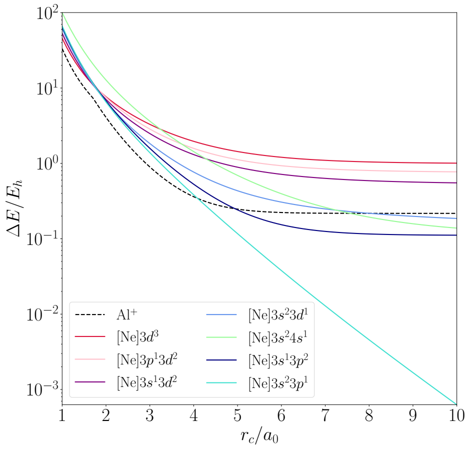

(c) r^{2}SCAN

Figure S138: Energies of various low lying configurations of hard-wall confined spin-polarized Al shown as the energy difference from unconfined Al as a function of the confinement radius $r_\infty = 1.0, 1.1, \ldots, 10.0a_0$. Note semilogarithmic scale.

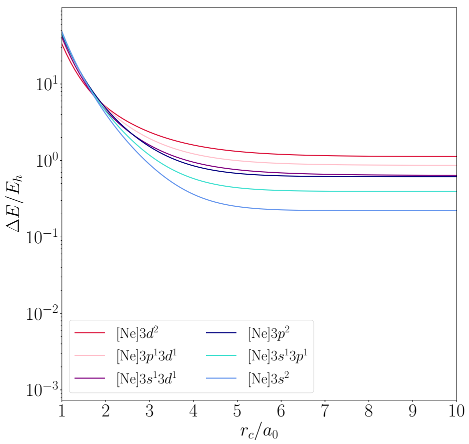

(a) PW92

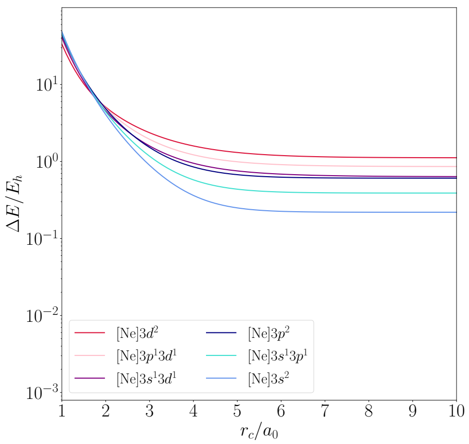

(b) PBE

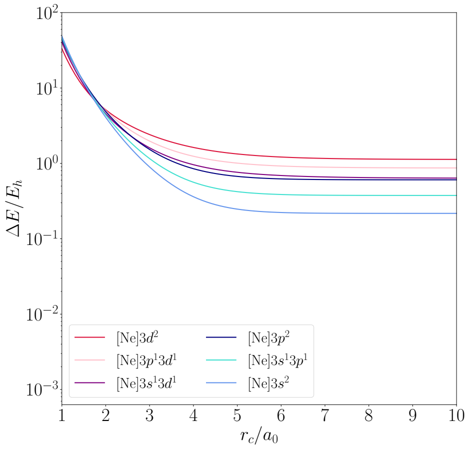

(c) r^{2}SCAN

Figure S139: Energies of various low lying configurations of the hard-wall confined spin-polarized monocation of Al shown as the energy difference from unconfined Al as a function of the confinement radius $r_\infty = 1.0, 1.1, \ldots, 10.0a_0$. Note semilogarithmic scale.

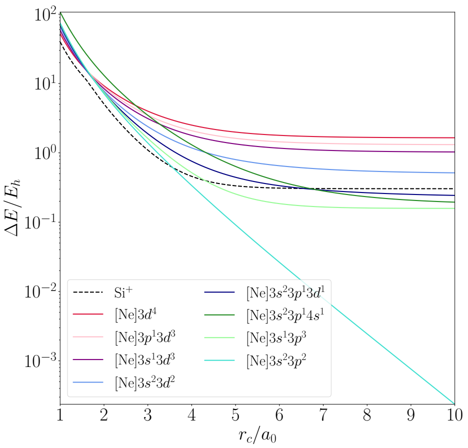

(a) PW92

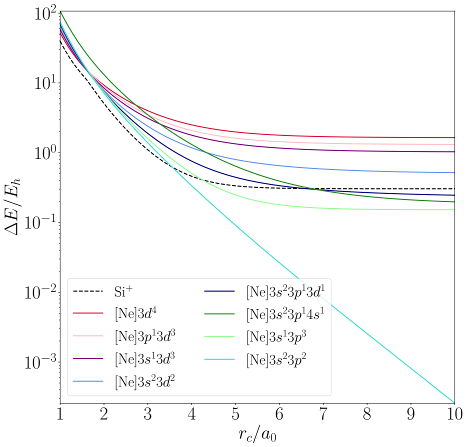

(b) PBE

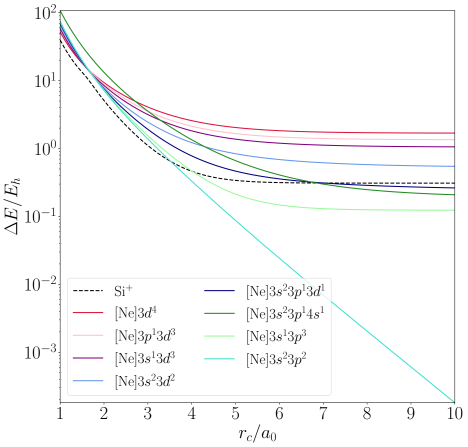

(c) r^{2}SCAN

Figure S140: Energies of various low lying configurations of hard-wall confined spin-polarized Si shown as the energy difference from unconfined Si as a function of the confinement radius $r_\infty = 1.0, 1.1, \ldots, 10.0 a_0$. Note semilogarithmic scale.

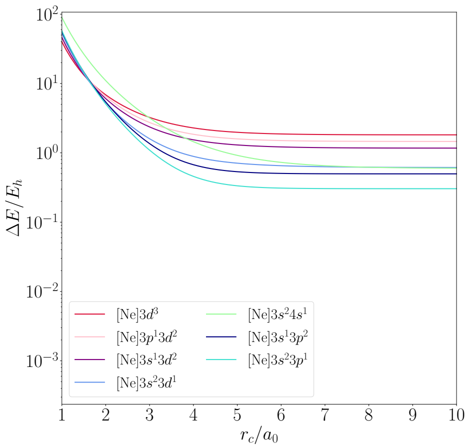

(a) PW92

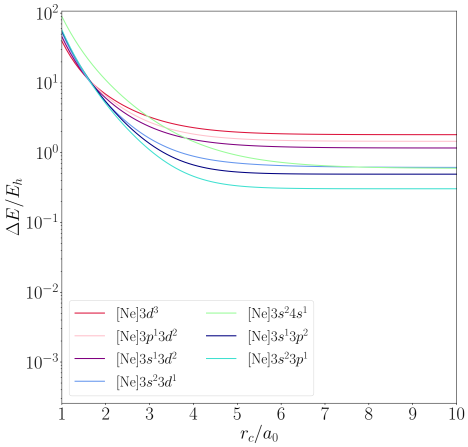

(b) PBE

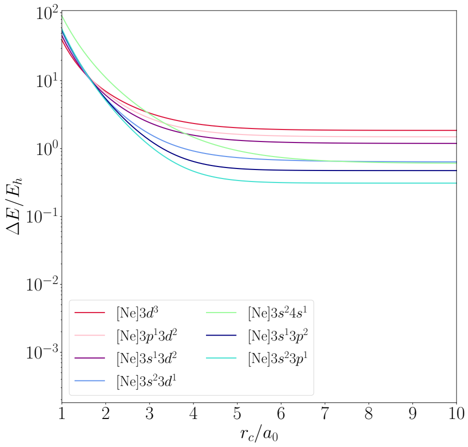

(c) r^{2}SCAN

Figure S141: Energies of various low lying configurations of the hard-wall confined spin-polarized monocation of Si shown as the energy difference from unconfined Si as a function of the confinement radius $r_\infty = 1.0, 1.1, \ldots, 10.0 a_0$. Note semilogarithmic scale.

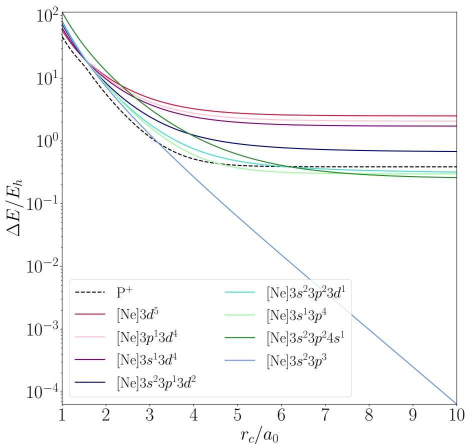

(a) PW92

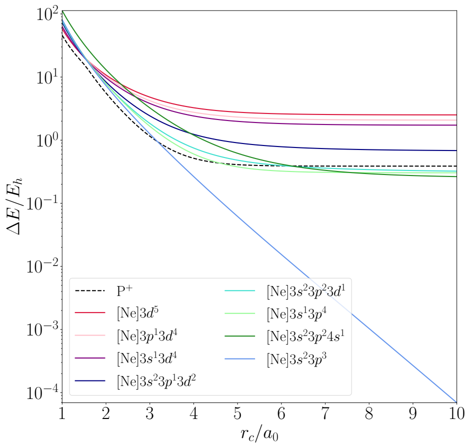

(b) PBE

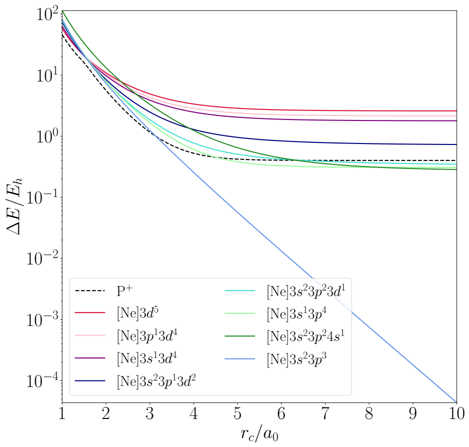

(c) r^{2}SCAN

Figure S142: Energies of various low lying configurations of hard-wall confined spin-polarized P shown as the energy difference from unconfined P as a function of the confinement radius $r_\infty = 1.0, 1.1, \ldots, 10.0 a_0$. Note semilogarithmic scale.

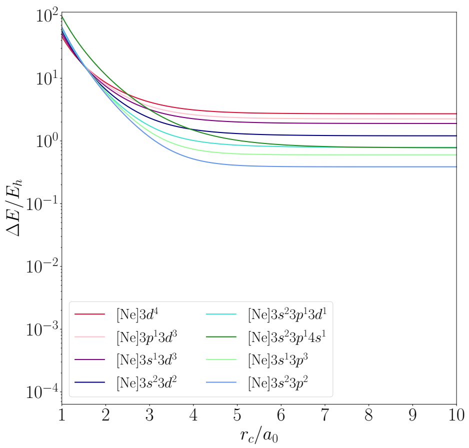

(a) PW92

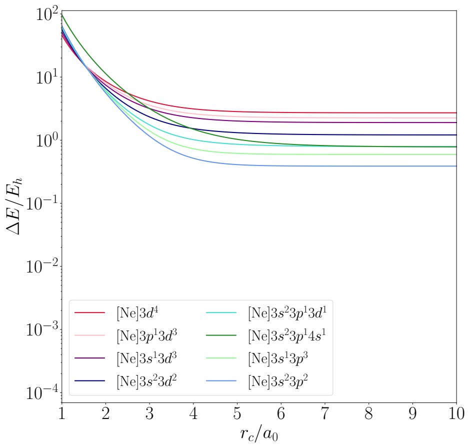

(b) PBE

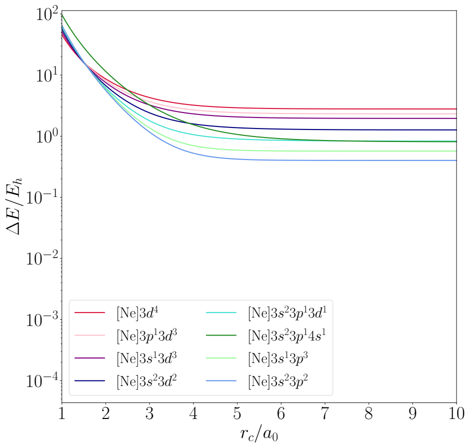

(c) r^{2}SCAN

Figure S143: Energies of various low lying configurations of the hard-wall confined spin-polarized monocation of P shown as the energy difference from unconfined P as a function of the confinement radius $r_\infty = 1.0, 1.1, \ldots, 10.0 a_0$. Note semilogarithmic scale.

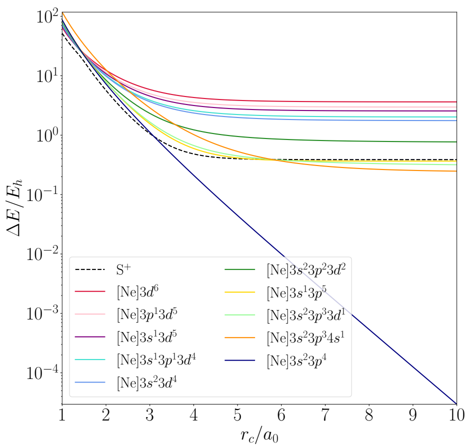

(a) PW92

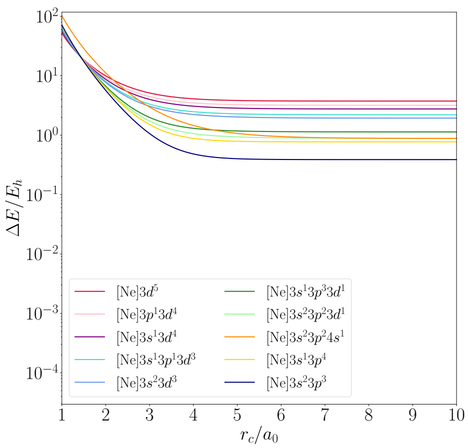

(a) PW92

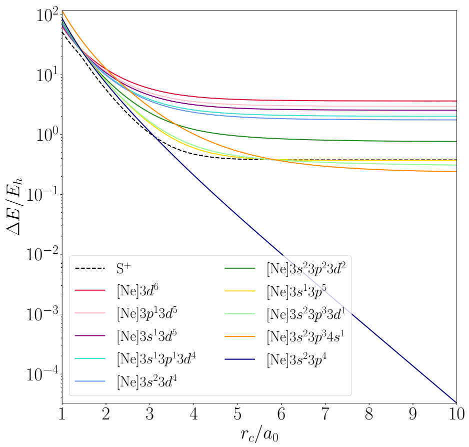

(b) PBE

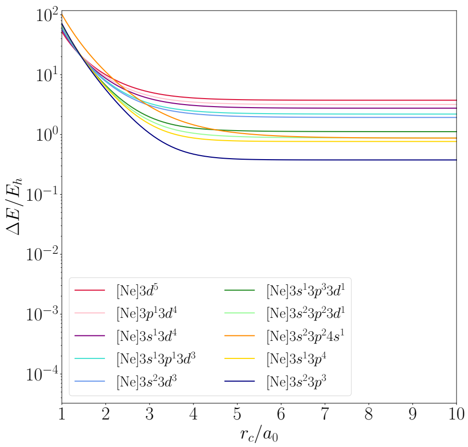

(b) PBE

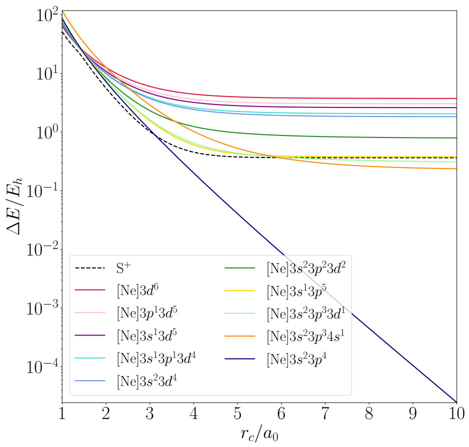

(c) r^{2}SCAN

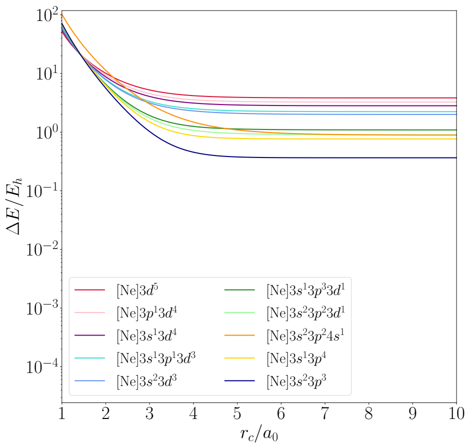

(c) r^{2}SCAN

Figure S144: Energies of various low lying configurations of hard-wall confined spin-polarized S shown as the energy difference from unconfined S as a function of the confinement radius $r_\infty = 1.0, 1.1, \ldots, 10.0a_0$. Note semilogarithmic scale.

Figure S145: Energies of various low lying configurations of the hard-wall confined spin-polarized monocation of S shown as the energy difference from unconfined S as a function of the confinement radius $r_\infty = 1.0, 1.1, \ldots, 10.0a_0$. Note semilogarithmic scale.

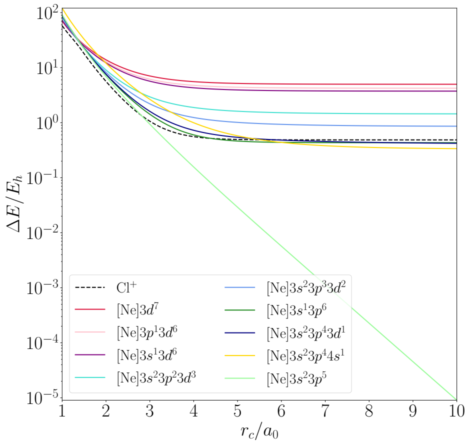

(a) PW92

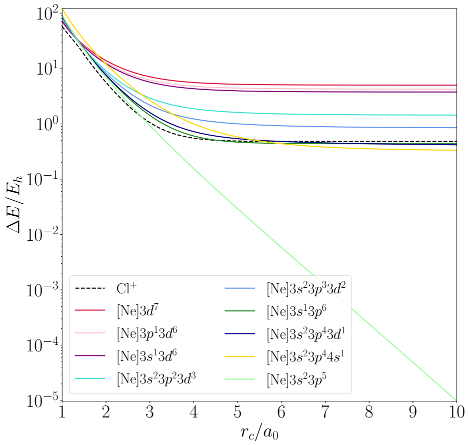

(b) PBE

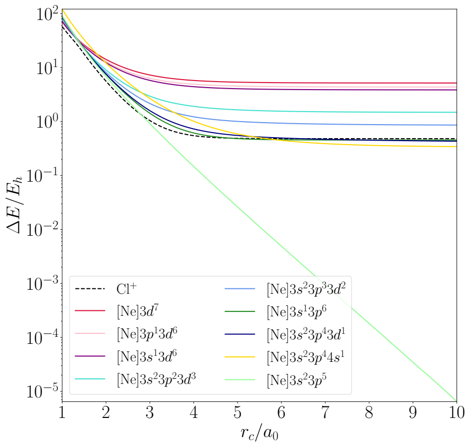

(c) r^{2}SCAN

Figure S146: Energies of various low lying configurations of hard-wall confined spin-polarized Cl shown as the energy difference from unconfined Cl as a function of the confinement radius $r_\infty = 1.0, 1.1, \ldots, 10.0 a_0$. Note semilogarithmic scale.

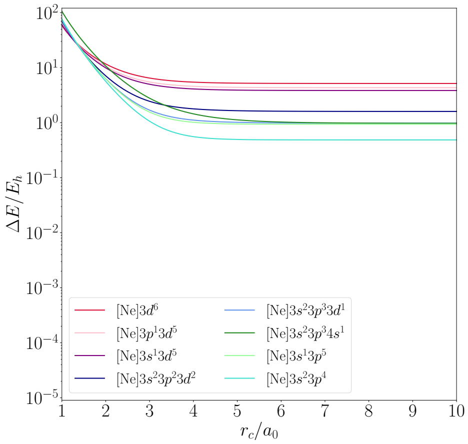

(a) PW92

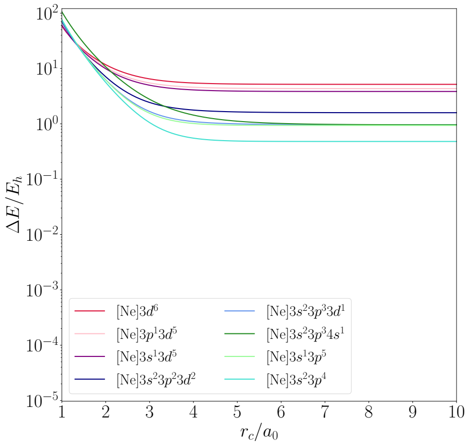

(b) PBE

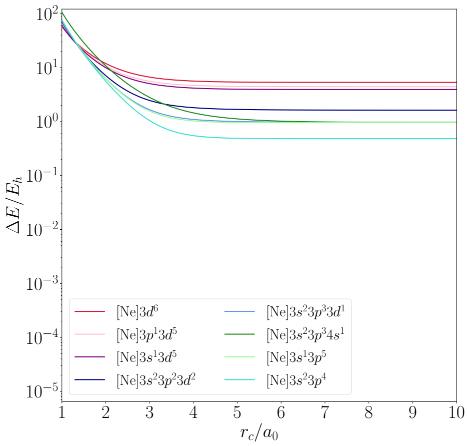

(c) r^{2}SCAN

Figure S147: Energies of various low lying configurations of the hard-wall confined spin-polarized monocation of Cl shown as the energy difference from unconfined Cl as a function of the confinement radius $r_\infty = 1.0, 1.1, \ldots, 10.0 a_0$. Note semilogarithmic scale.

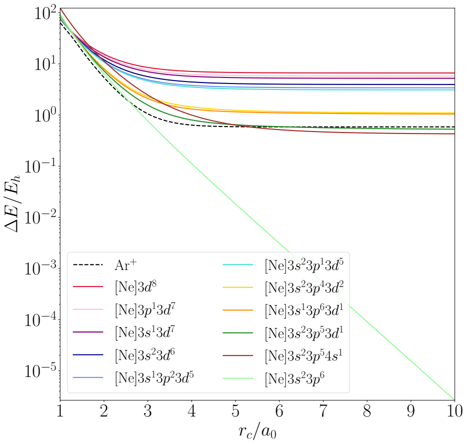

(a) PW92

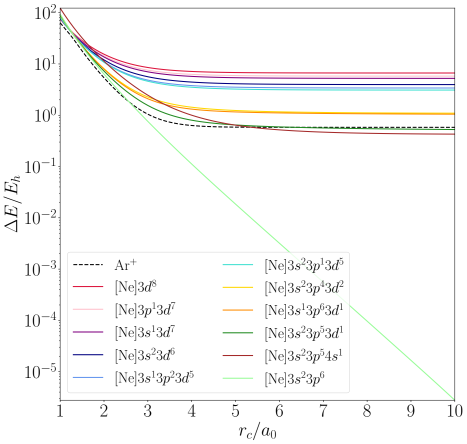

(b) PBE

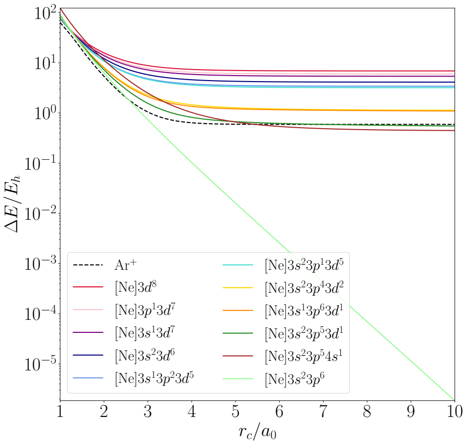

(c) r^{2}SCAN

Figure S148: Energies of various low lying configurations of hard-wall confined spin-polarized Ar shown as the energy difference from unconfined Ar as a function of the confinement radius $r_\infty = 1.0, 1.1, \ldots, 10.0 a_0$. Note semilogarithmic scale.

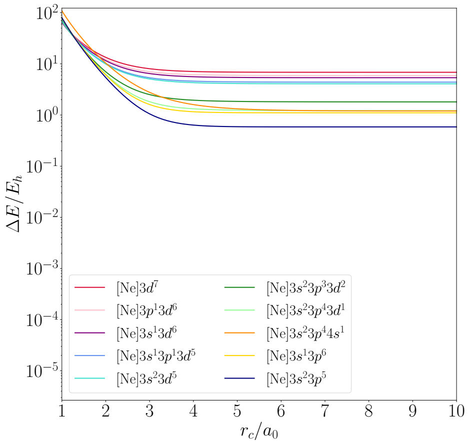

(a) PW92

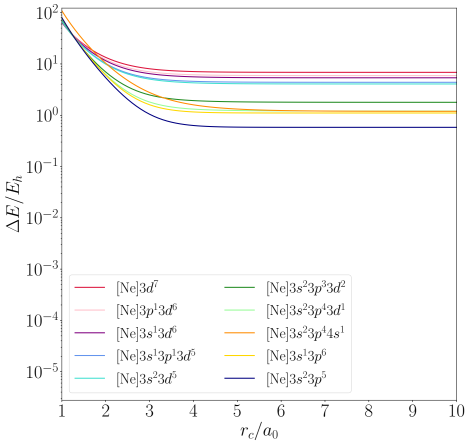

(b) PBE

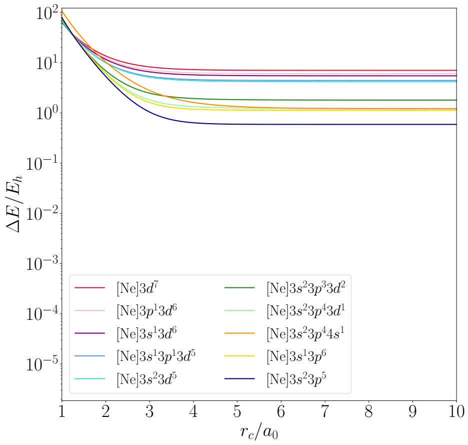

(c) r^{2}SCAN

Figure S149: Energies of various low lying configurations of the hard-wall confined spin-polarized monocation of Ar shown as the energy difference from unconfined Ar as a function of the confinement radius $r_\infty = 1.0, 1.1, \ldots, 10.0 a_0$. Note semilogarithmic scale.

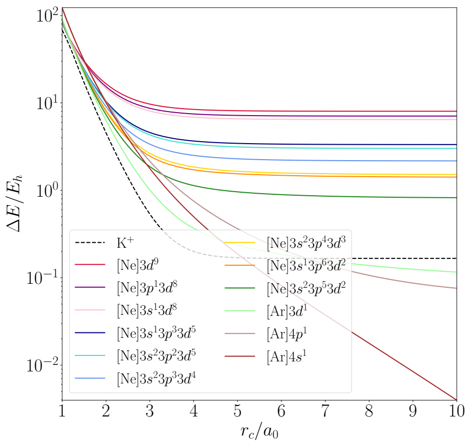

(a) PW92

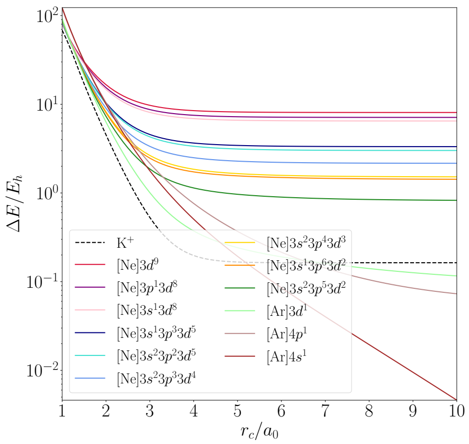

(b) PBE

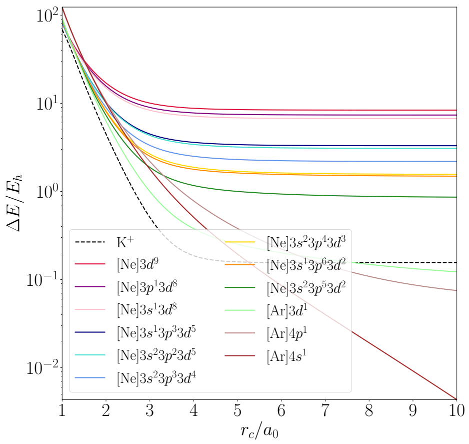

(c) r^{2}SCAN

Figure S150: Energies of various low lying configurations of hard-wall confined spin-polarized K shown as the energy difference from unconfined K as a function of the confinement radius $r_\infty = 1.0, 1.1, \ldots, 10.0 a_0$. Note semilogarithmic scale.

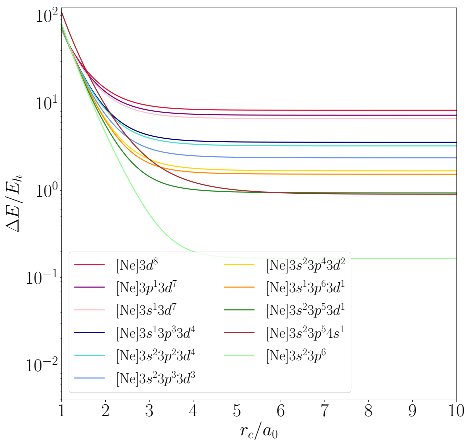

(a) PW92

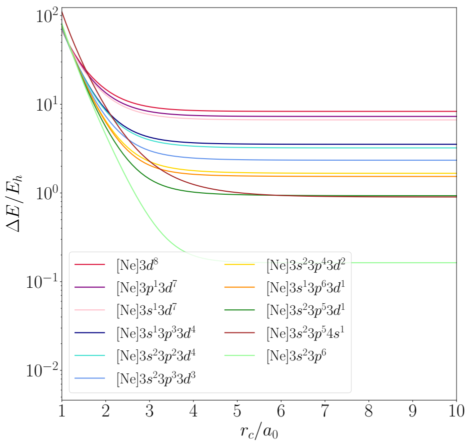

(b) PBE

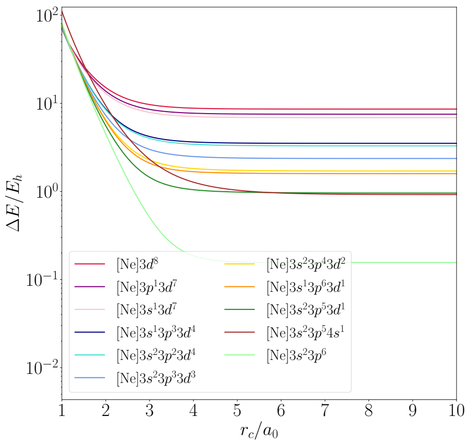

(c) r^{2}SCAN

Figure S151: Energies of various low lying configurations of the hard-wall confined spin-polarized monocation of K shown as the energy difference from unconfined K as a function of the confinement radius $r_\infty = 1.0, 1.1, \ldots, 10.0 a_0$. Note semilogarithmic scale.

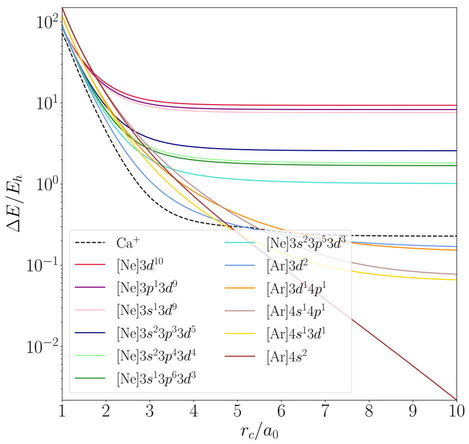

(a) PW92

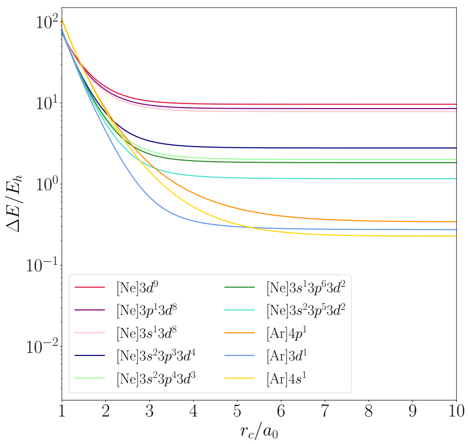

(a) PW92

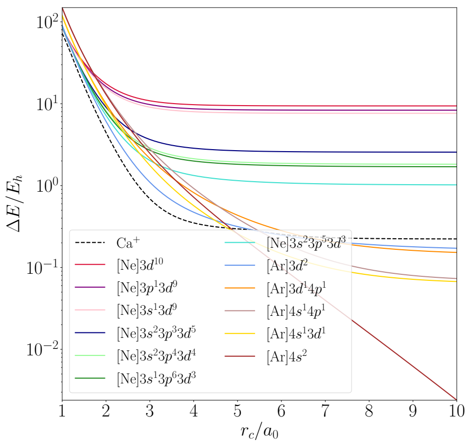

(b) PBE

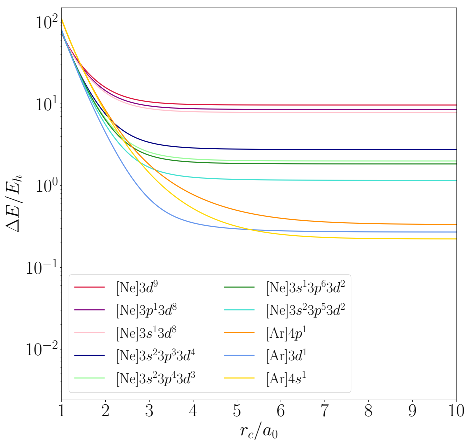

(b) PBE

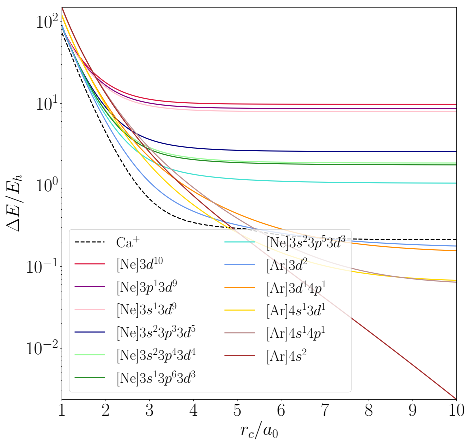

(c) r^{2}SCAN

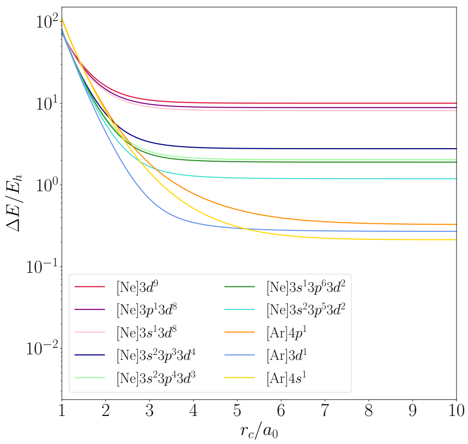

(c) r^{2}SCAN

Figure S152: Energies of various low lying configurations of hard-wall confined spin-polarized Ca shown as the energy difference from unconfined Ca as a function of the confinement radius $r_\infty = 1.0, 1.1, \ldots, 10.0a_0$. Note semilogarithmic scale.

Figure S153: Energies of various low lying configurations of the hard-wall confined spin-polarized monocation of Ca shown as the energy difference from unconfined Ca as a function of the confinement radius $r_\infty = 1.0, 1.1, \ldots, 10.0a_0$. Note semilogarithmic scale.

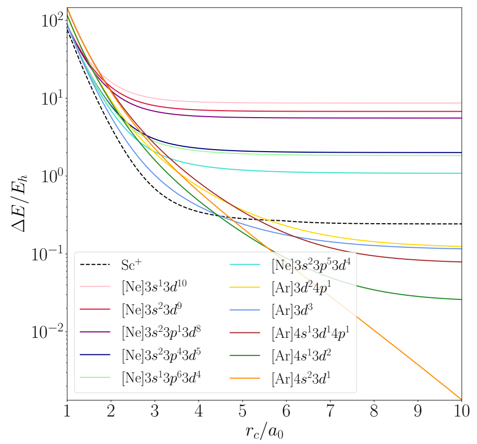

(a) PW92

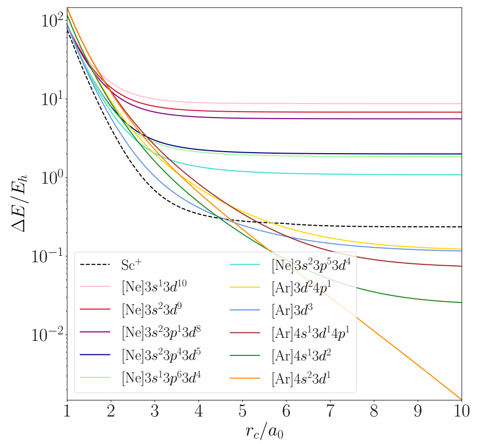

(b) PBE

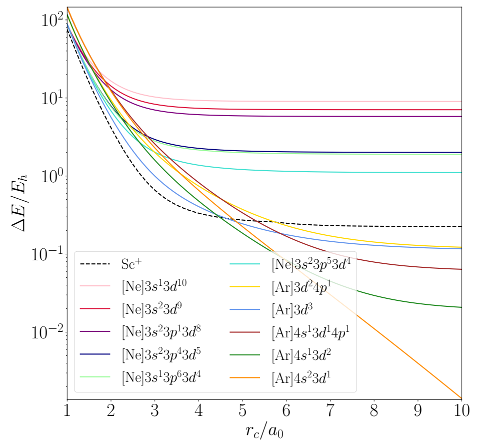

(c) r^{2}SCAN

Figure S154: Energies of various low lying configurations of hard-wall confined spin-polarized Sc shown as the energy difference from unconfined Sc as a function of the confinement radius $r_\infty = 1.0, 1.1, \ldots, 10.0a_0$. Note semilogarithmic scale.

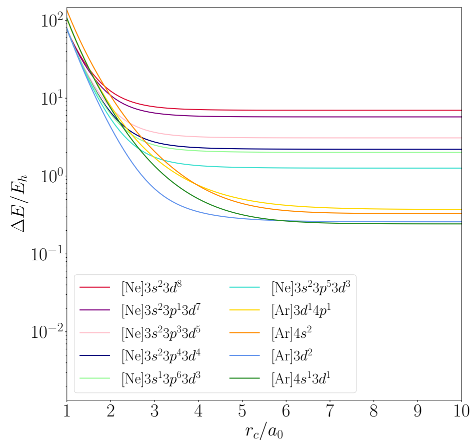

(a) PW92

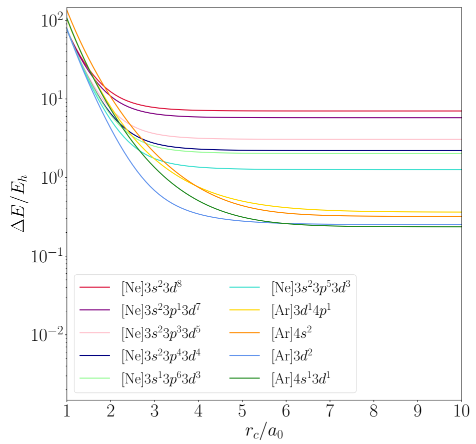

(b) PBE

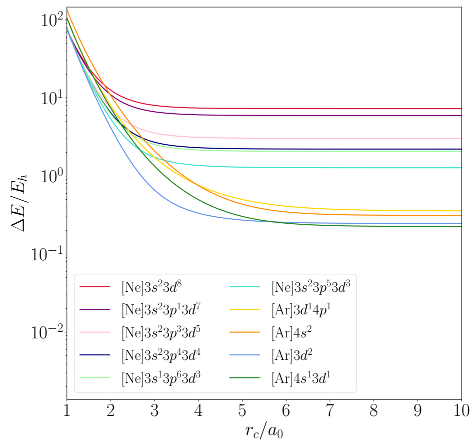

(c) r^{2}SCAN

Figure S155: Energies of various low lying configurations of the hard-wall confined spin-polarized monocation of Sc shown as the energy difference from unconfined Sc as a function of the confinement radius $r_\infty = 1.0, 1.1, \ldots, 10.0a_0$. Note semilogarithmic scale.

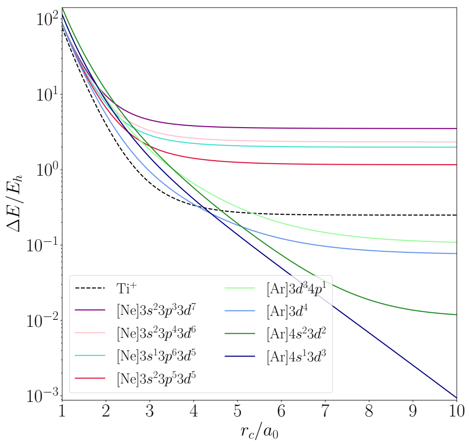

(a) PW92

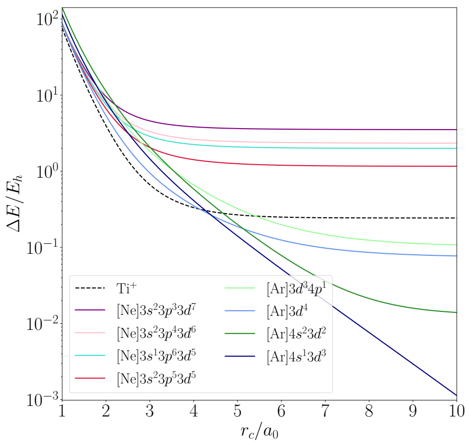

(b) PBE

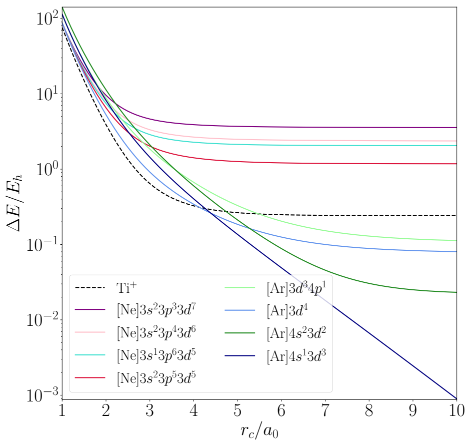

(c) r^{2}SCAN

Figure S156: Energies of various low lying configurations of hard-wall confined spin-polarized Ti shown as the energy difference from unconfined Ti as a function of the confinement radius $r_\infty = 1.0, 1.1, \ldots, 10.0a_0$. Note semilogarithmic scale.

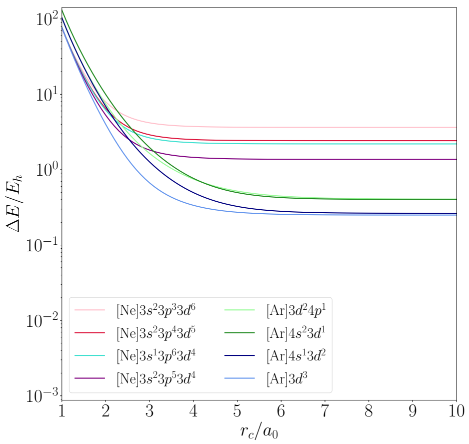

(a) PW92

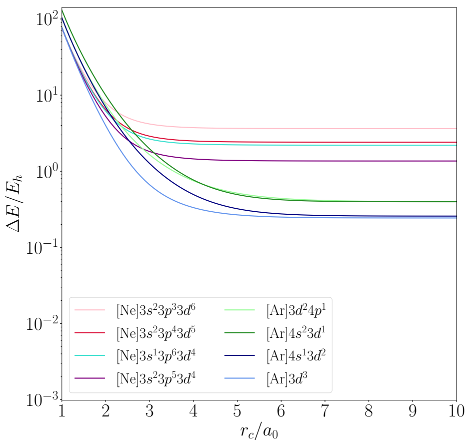

(b) PBE

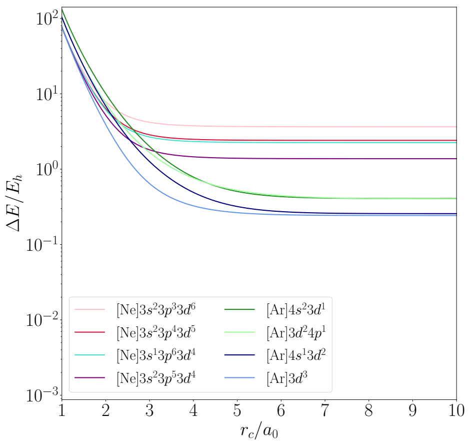

(c) r^{2}SCAN

Figure S157: Energies of various low lying configurations of the hard-wall confined spin-polarized monocation of Ti shown as the energy difference from unconfined Ti as a function of the confinement radius $r_\infty = 1.0, 1.1, \ldots, 10.0a_0$. Note semilogarithmic scale.

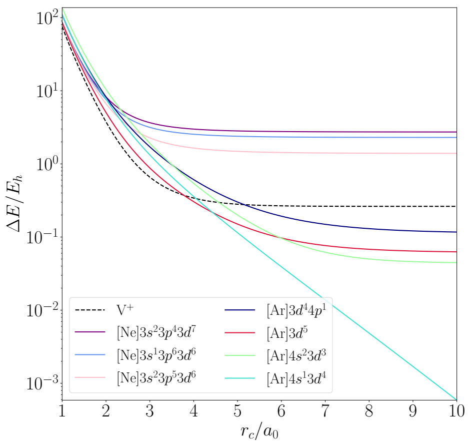

(a) PW92

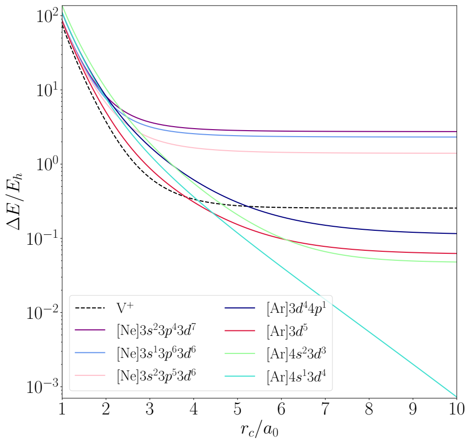

(b) PBE

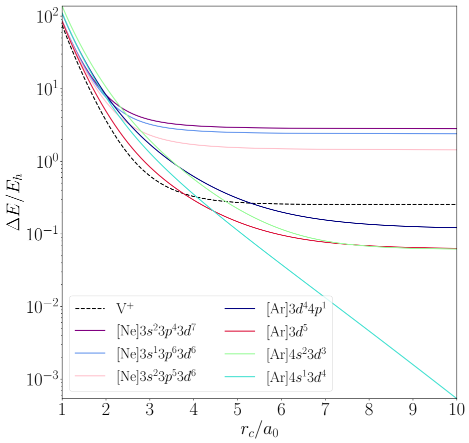

(c) r^{2}SCAN

Figure S158: Energies of various low lying configurations of hard-wall confined spin-polarized V shown as the energy difference from unconfined V as a function of the confinement radius $r_\infty = 1.0, 1.1, \ldots, 10.0a_0$. Note semilogarithmic scale.

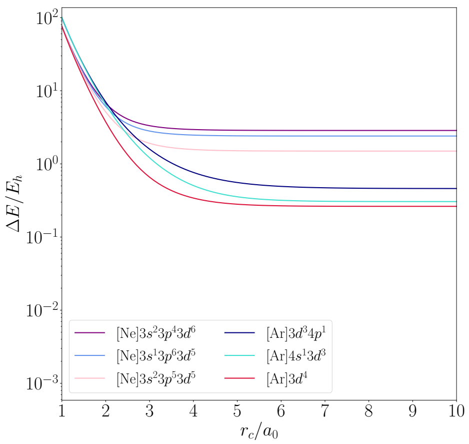

(a) PW92

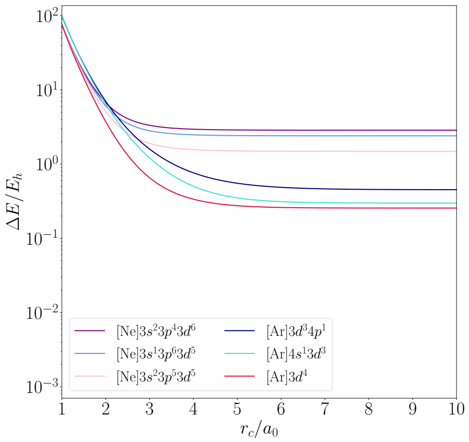

(b) PBE

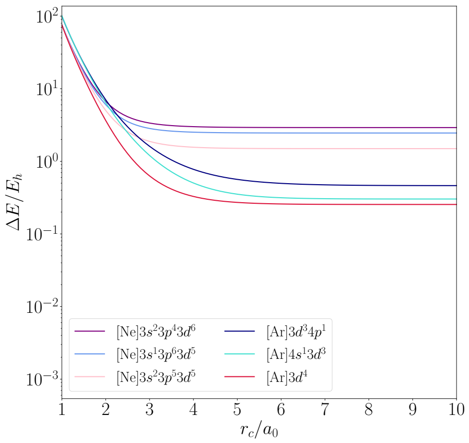

(c) r^{2}SCAN

Figure S159: Energies of various low lying configurations of the hard-wall confined spin-polarized monocation of V shown as the energy difference from unconfined V as a function of the confinement radius $r_\infty = 1.0, 1.1, \ldots, 10.0a_0$. Note semilogarithmic scale.

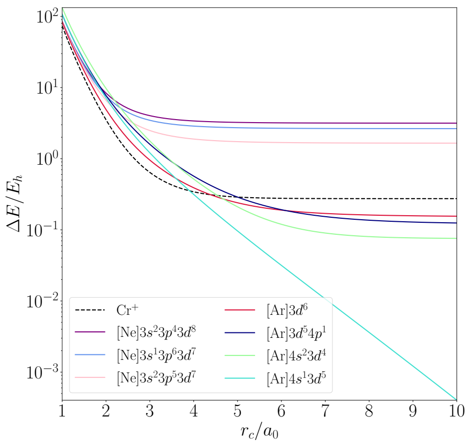

(a) PW92

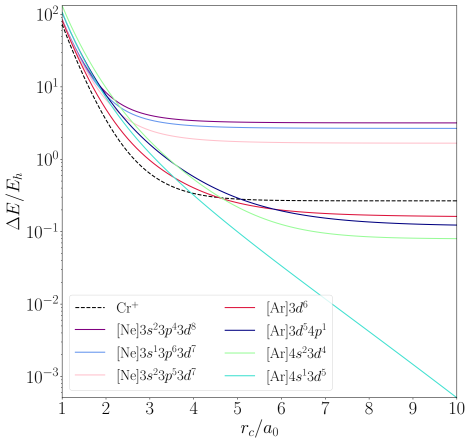

(b) PBE

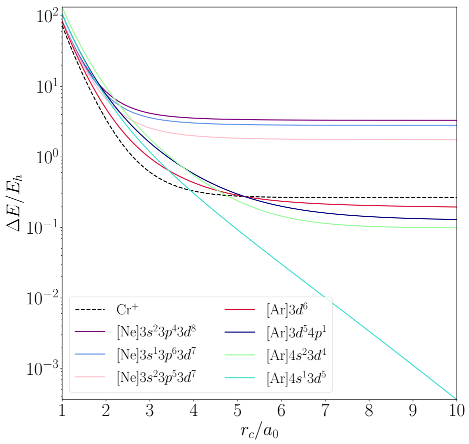

(c) r^{2}SCAN

Figure S160: Energies of various low lying configurations of hard-wall confined spin-polarized Cr shown as the energy difference from unconfined Cr as a function of the confinement radius $r_\infty = 1.0, 1.1, \ldots, 10.0 a_0$. Note semilogarithmic scale.

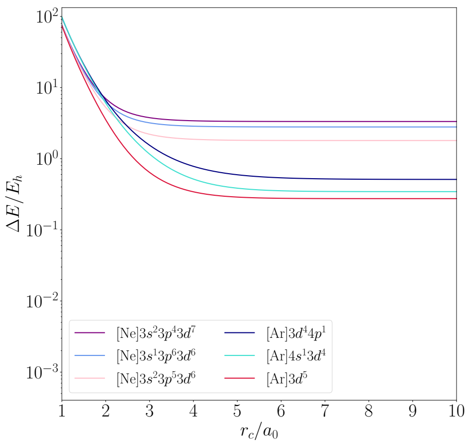

(a) PW92

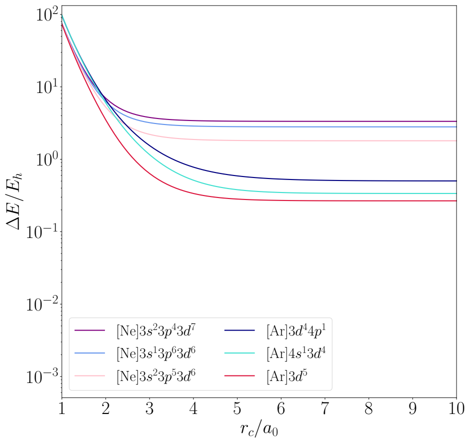

(b) PBE

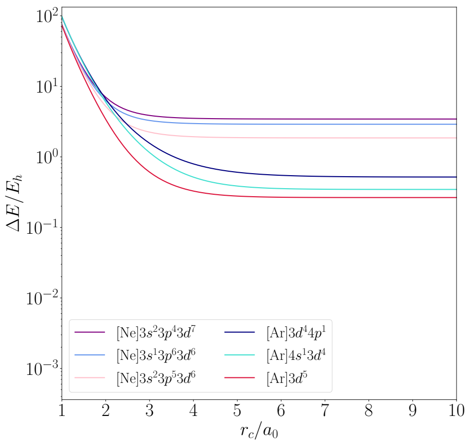

(c) r^{2}SCAN

Figure S161: Energies of various low lying configurations of the hard-wall confined spin-polarized monocation of Cr shown as the energy difference from unconfined Cr as a function of the confinement radius $r_\infty = 1.0, 1.1, \ldots, 10.0 a_0$. Note semilogarithmic scale.

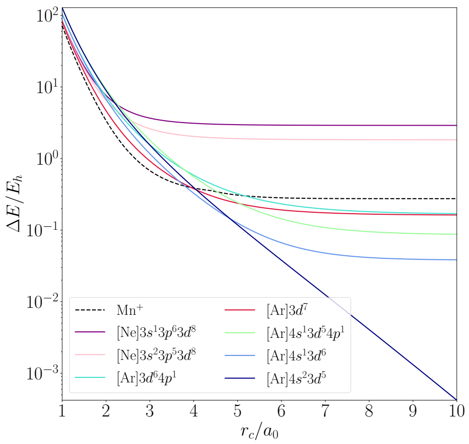

(a) PW92

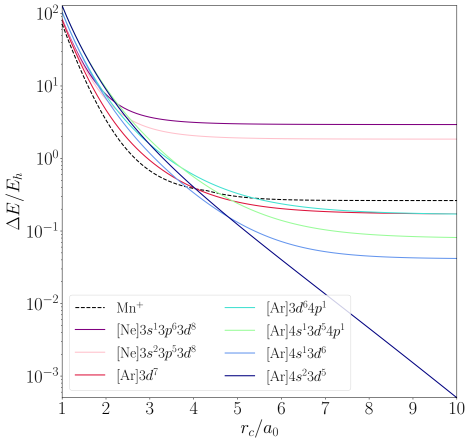

(b) PBE

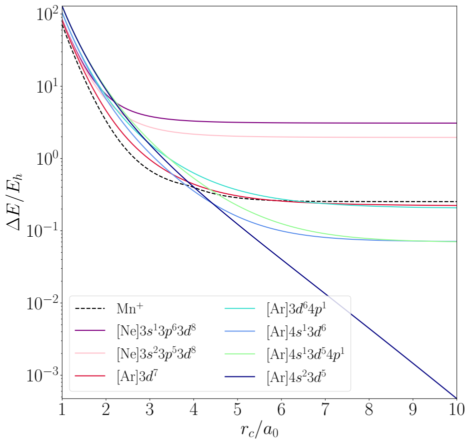

(c) r^{2}SCAN

Figure S162: Energies of various low lying configurations of hard-wall confined spin-polarized Mn shown as the energy difference from unconfined Mn as a function of the confinement radius $r_\infty = 1.0, 1.1, \ldots, 10.0a_0$. Note semilogarithmic scale.

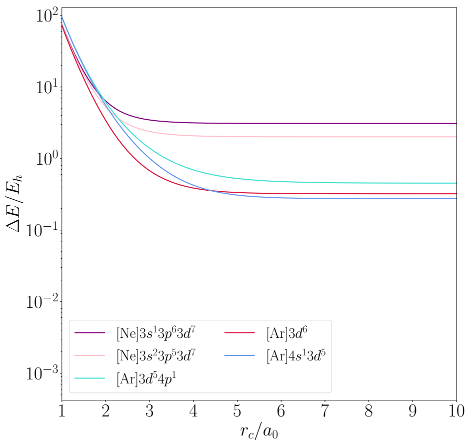

(a) PW92

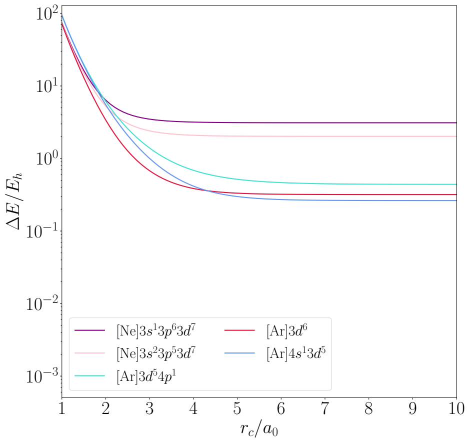

(b) PBE

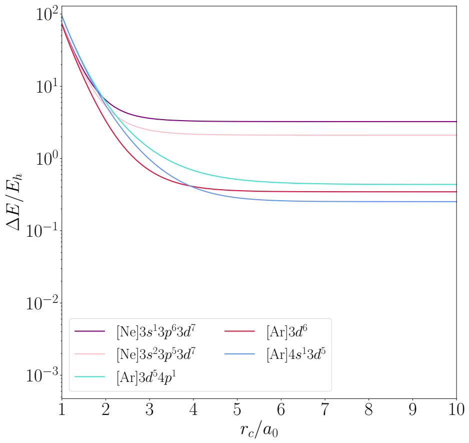

(c) r^{2}SCAN

Figure S163: Energies of various low lying configurations of the hard-wall confined spin-polarized monocation of Mn shown as the energy difference from unconfined Mn as a function of the confinement radius $r_\infty = 1.0, 1.1, \ldots, 10.0a_0$. Note semilogarithmic scale.

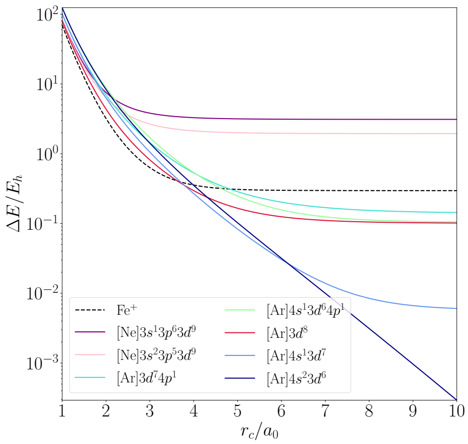

(a) PW92

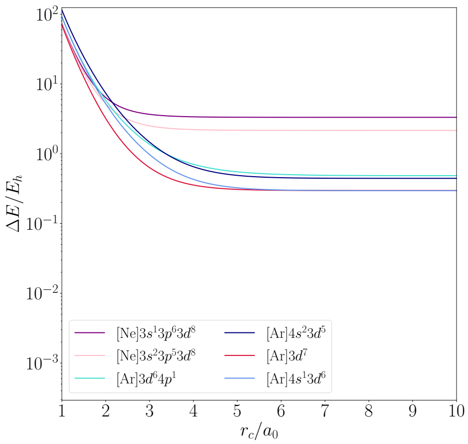

(a) PW92

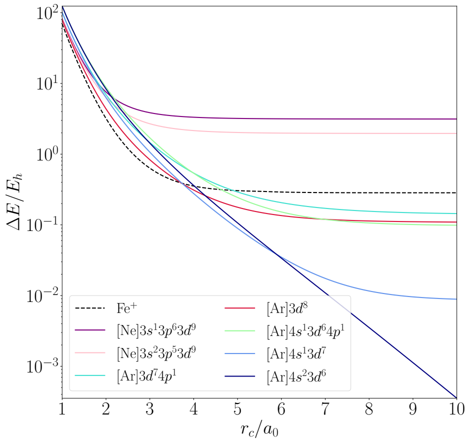

(b) PBE

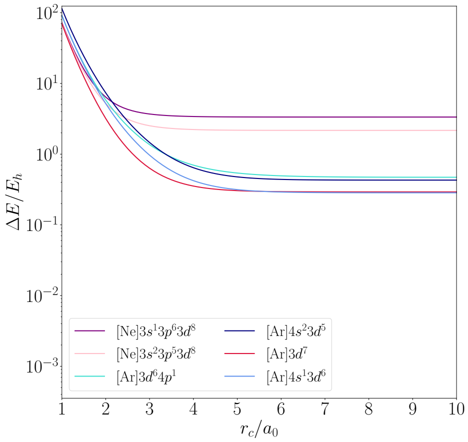

(b) PBE

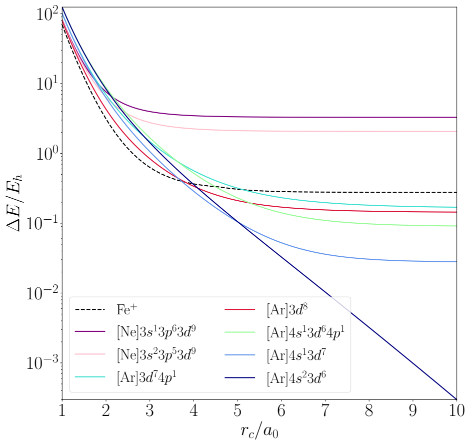

(c) r^{2}SCAN

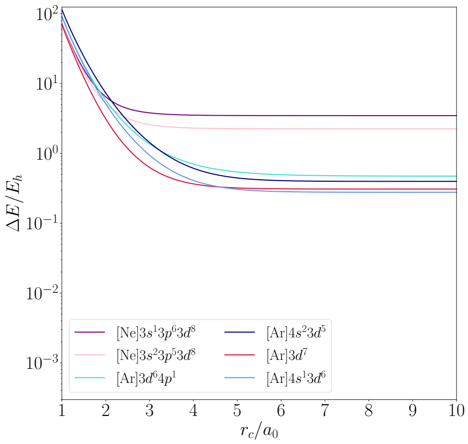

(c) r^{2}SCAN

Figure S164: Energies of various low lying configurations of hard-wall confined spin-polarized Fe shown as the energy difference from unconfined Fe as a function of the confinement radius $r_\infty = 1.0, 1.1, \ldots, 10.0a_0$. Note semilogarithmic scale.

Figure S165: Energies of various low lying configurations of the hard-wall confined spin-polarized monocation of Fe shown as the energy difference from unconfined Fe as a function of the confinement radius $r_\infty = 1.0, 1.1, \ldots, 10.0a_0$. Note semilogarithmic scale.

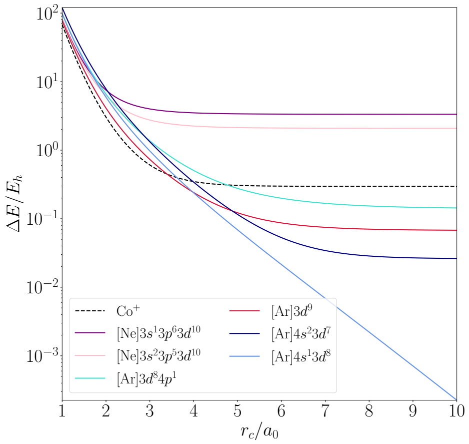

(a) PW92

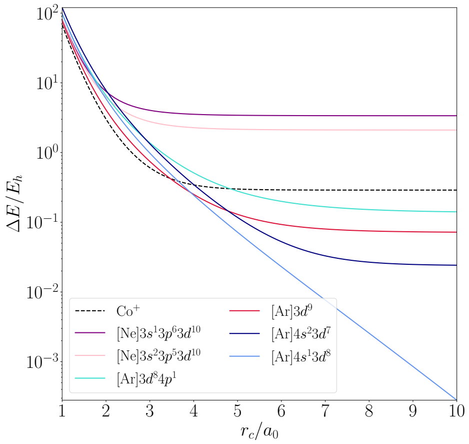

(b) PBE

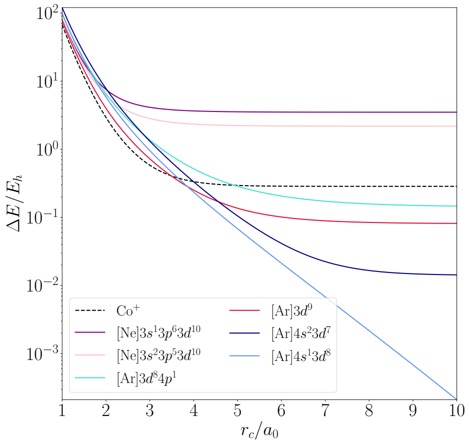

(c) r^{2}SCAN

Figure S166: Energies of various low lying configurations of hard-wall confined spin-polarized Co shown as the energy difference from unconfined Co as a function of the confinement radius $r_\infty = 1.0, 1.1, \ldots, 10.0a_0$. Note semilogarithmic scale.

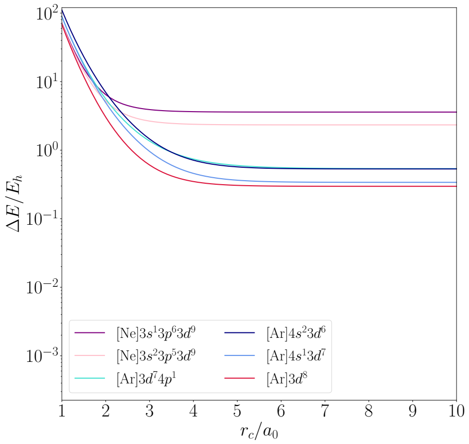

(a) PW92

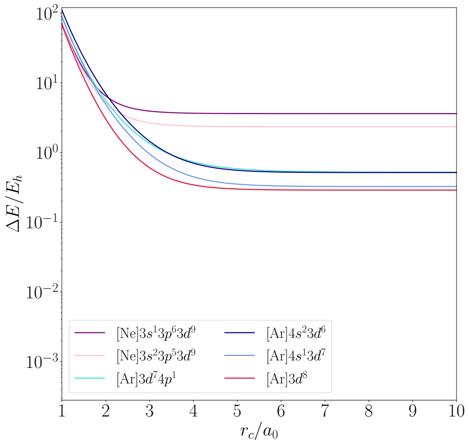

(b) PBE

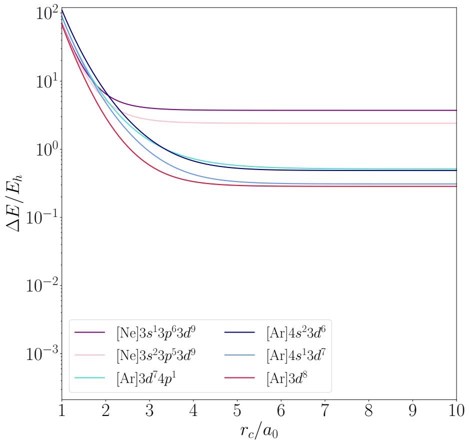

(c) r^{2}SCAN

Figure S167: Energies of various low lying configurations of the hard-wall confined spin-polarized monocation of Co shown as the energy difference from unconfined Co as a function of the confinement radius $r_\infty = 1.0, 1.1, \ldots, 10.0a_0$. Note semilogarithmic scale.

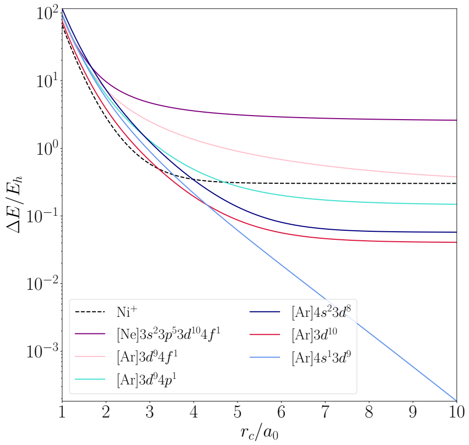

(a) PW92

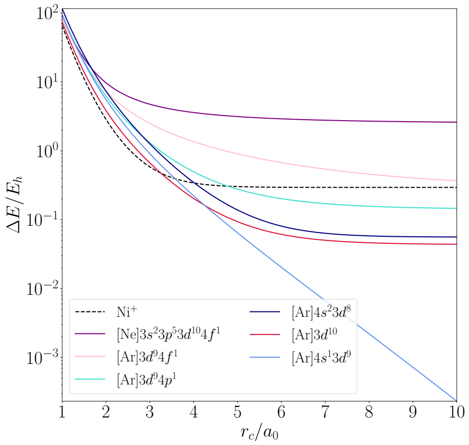

(b) PBE

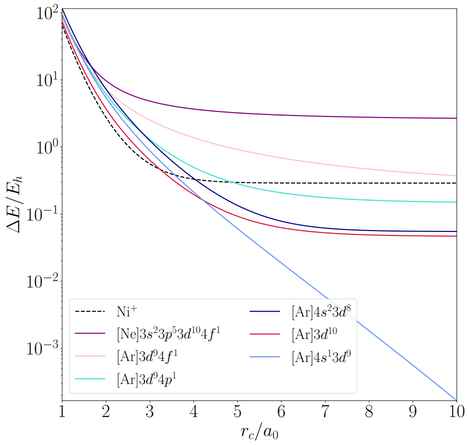

(c) r^{2}SCAN

Figure S168: Energies of various low lying configurations of hard-wall confined spin-polarized Ni shown as the energy difference from unconfined Ni as a function of the confinement radius $r_\infty = 1.0, 1.1, \ldots, 10.0a_0$. Note semilogarithmic scale.

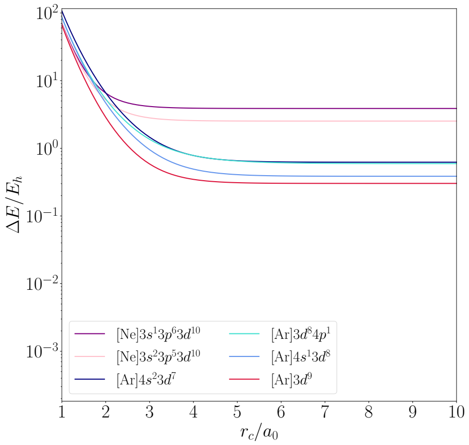

(a) PW92

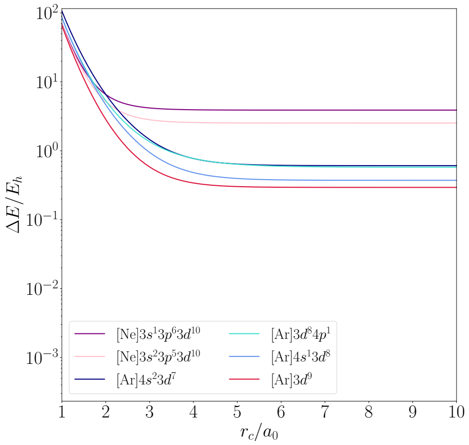

(b) PBE

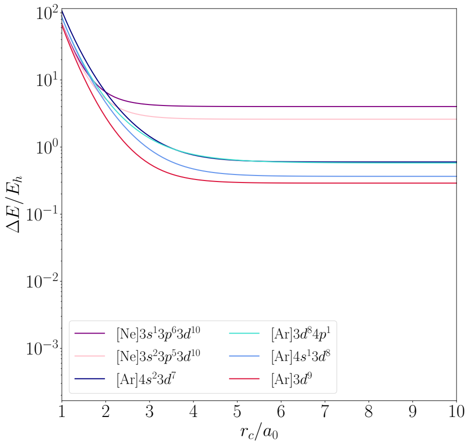

(c) r^{2}SCAN

Figure S169: Energies of various low lying configurations of the hard-wall confined spin-polarized monocation of Ni shown as the energy difference from unconfined Ni as a function of the confinement radius $r_\infty = 1.0, 1.1, \ldots, 10.0a_0$. Note semilogarithmic scale.

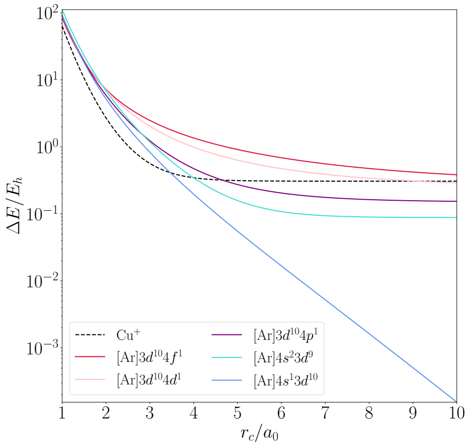

(a) PW92

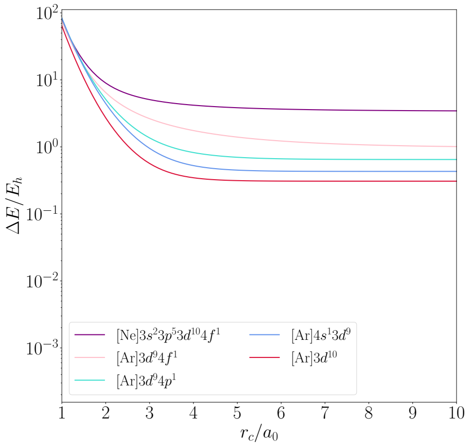

(a) PW92

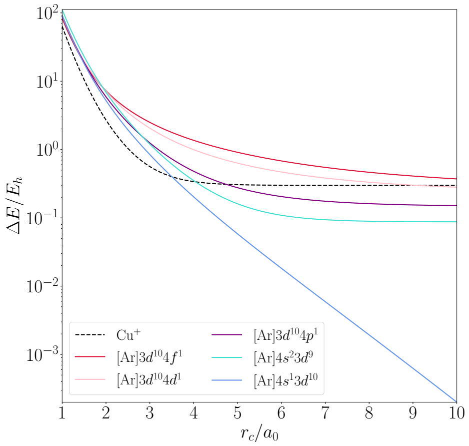

(b) PBE

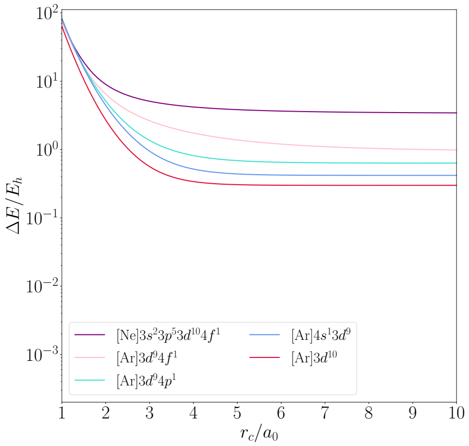

(b) PBE

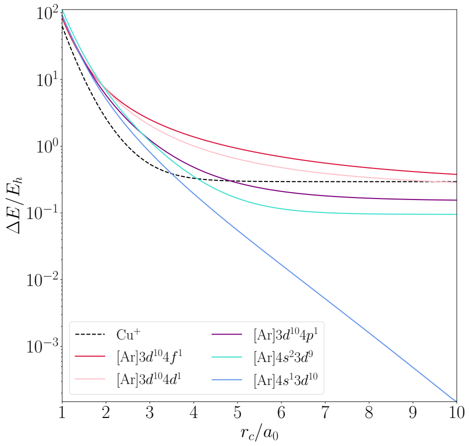

(c) r^{2}SCAN

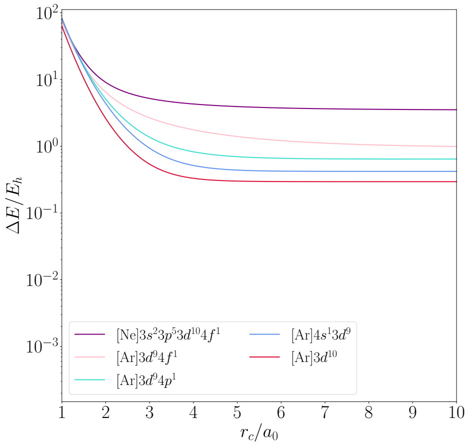

(c) r^{2}SCAN

Figure S170: Energies of various low lying configurations of hard-wall confined spin-polarized Cu shown as the energy difference from unconfined Cu as a function of the confinement radius $r_\infty = 1.0, 1.1, \ldots, 10.0a_0$. Note semilogarithmic scale.

Figure S171: Energies of various low lying configurations of the hard-wall confined spin-polarized monocation of Cu shown as the energy difference from unconfined Cu as a function of the confinement radius $r_\infty = 1.0, 1.1, \ldots, 10.0a_0$. Note semilogarithmic scale.

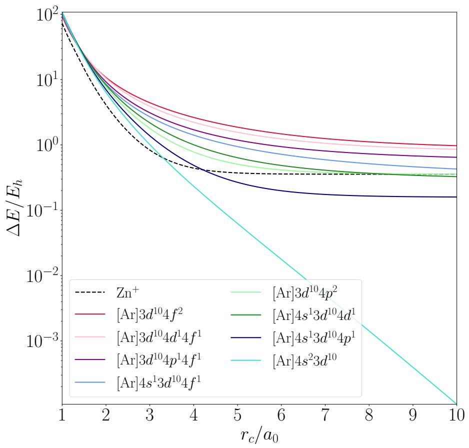

(a) PW92

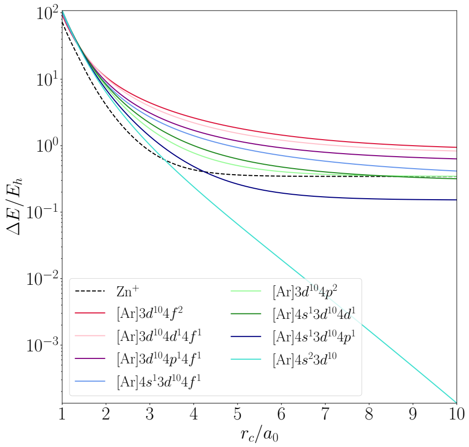

(b) PBE

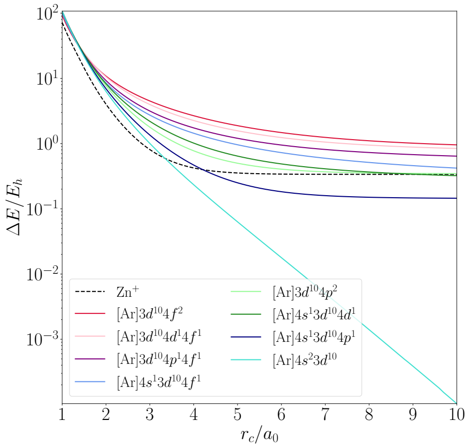

(c) r^{2}SCAN

Figure S172: Energies of various low lying configurations of hard-wall confined spin-polarized Zn shown as the energy difference from unconfined Zn as a function of the confinement radius $r_\infty = 1.0, 1.1, \ldots, 10.0a_0$. Note semilogarithmic scale.

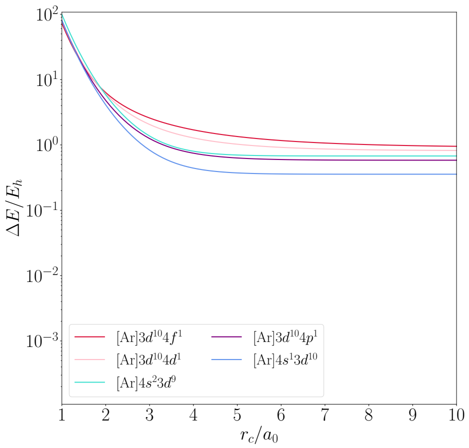

(a) PW92

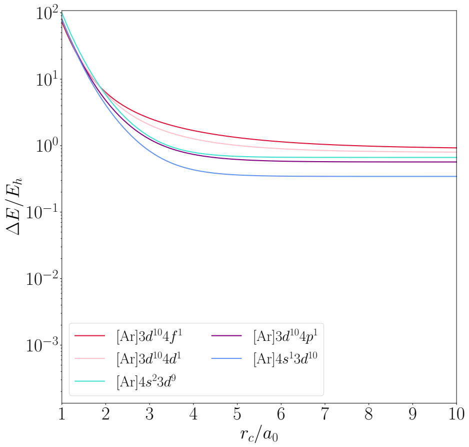

(b) PBE

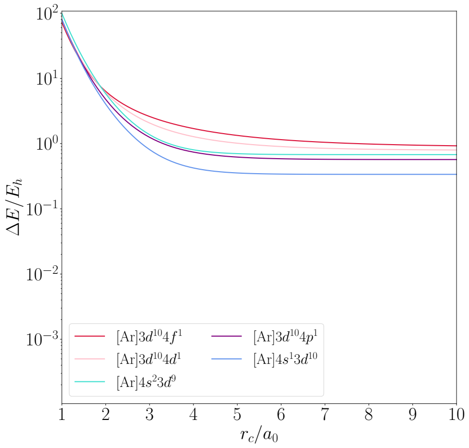

(c) r^{2}SCAN

Figure S173: Energies of various low lying configurations of the hard-wall confined spin-polarized monocation of Zn shown as the energy difference from unconfined Zn as a function of the confinement radius $r_\infty = 1.0, 1.1, \ldots, 10.0a_0$. Note semilogarithmic scale.

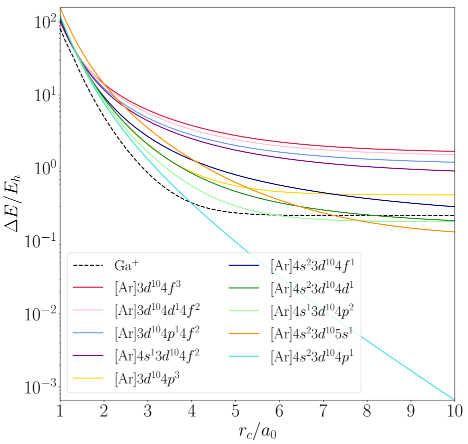

(a) PW92

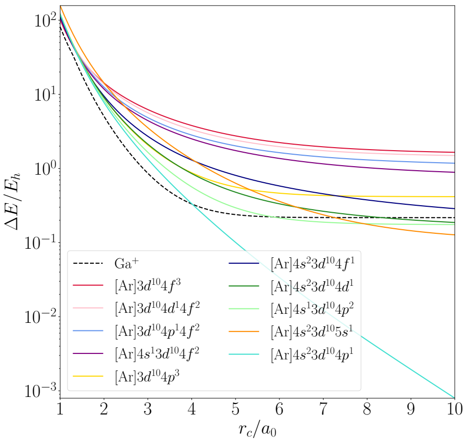

(b) PBE

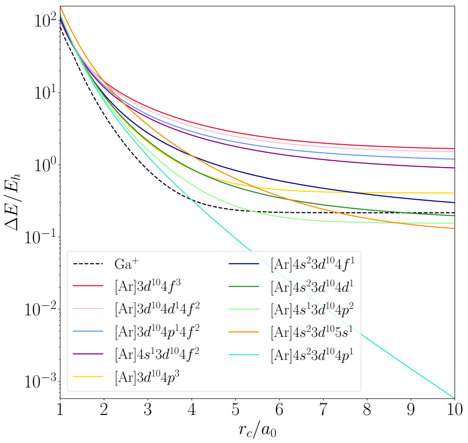

(c) r^{2}SCAN

Figure S174: Energies of various low lying configurations of hard-wall confined spin-polarized Ga shown as the energy difference from unconfined Ga as a function of the confinement radius $r_\infty = 1.0, 1.1, \ldots, 10.0a_0$. Note semilogarithmic scale.

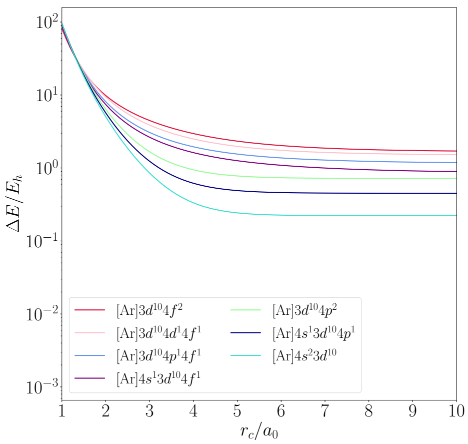

(a) PW92

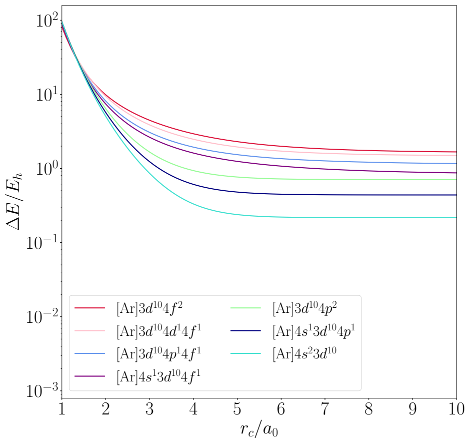

(b) PBE

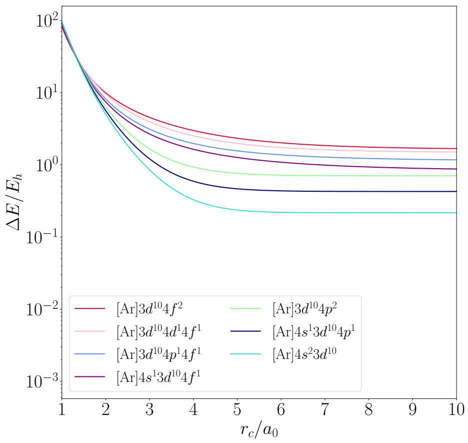

(c) r^{2}SCAN

Figure S175: Energies of various low lying configurations of the hard-wall confined spin-polarized monocation of Ga shown as the energy difference from unconfined Ga as a function of the confinement radius $r_\infty = 1.0, 1.1, \ldots, 10.0a_0$. Note semilogarithmic scale.

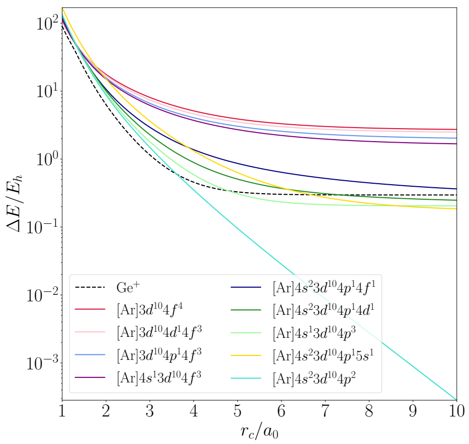

(a) PW92

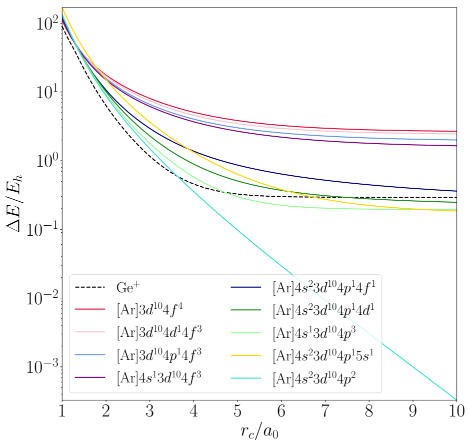

(b) PBE

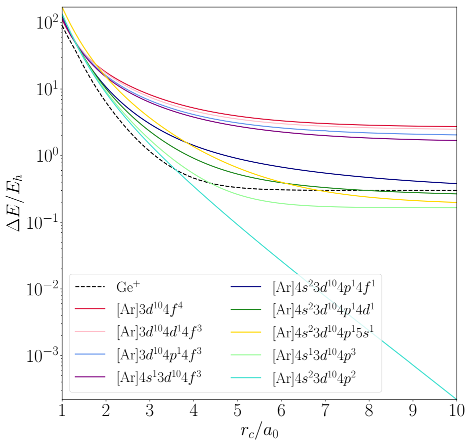

(c) r^{2}SCAN

Figure S176: Energies of various low lying configurations of hard-wall confined spin-polarized Ge shown as the energy difference from unconfined Ge as a function of the confinement radius $r_\infty = 1.0, 1.1, \ldots, 10.0a_0$. Note semilogarithmic scale.

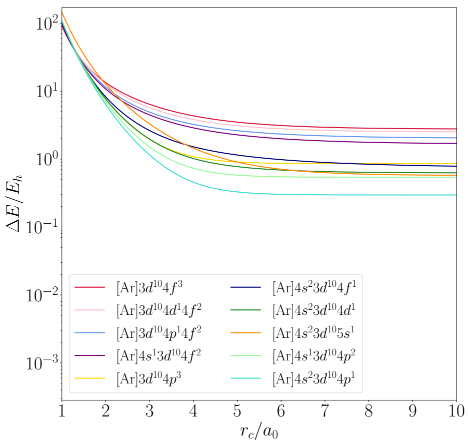

(a) PW92

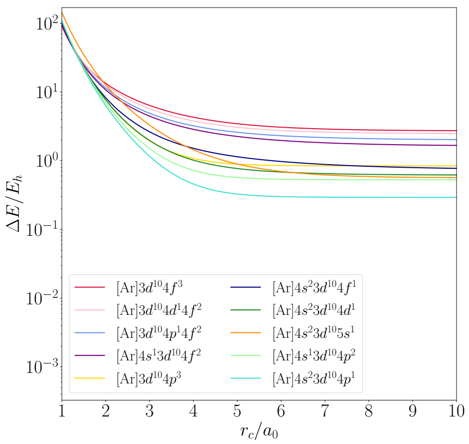

(b) PBE

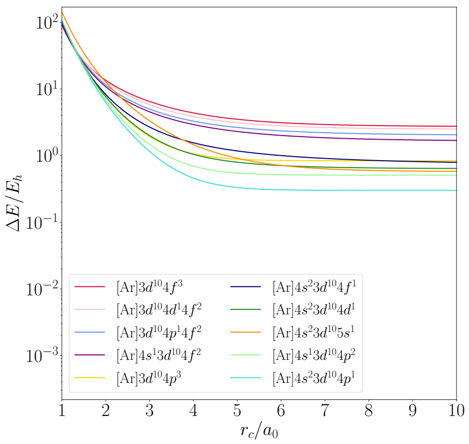

(c) r^{2}SCAN

Figure S177: Energies of various low lying configurations of the hard-wall confined spin-polarized monocation of Ge shown as the energy difference from unconfined Ge as a function of the confinement radius $r_\infty = 1.0, 1.1, \ldots, 10.0a_0$. Note semilogarithmic scale.

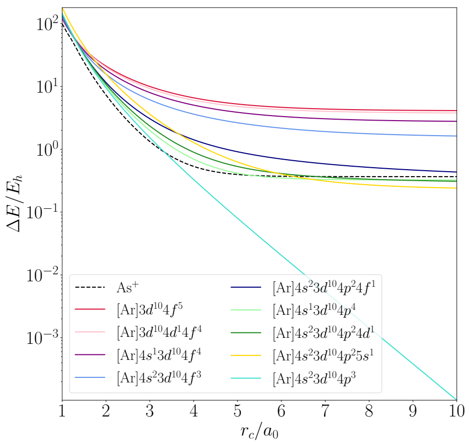

(a) PW92

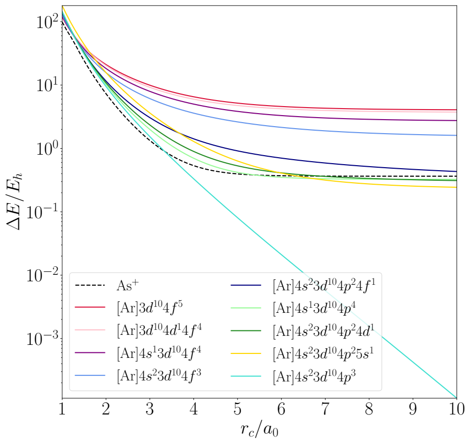

(b) PBE

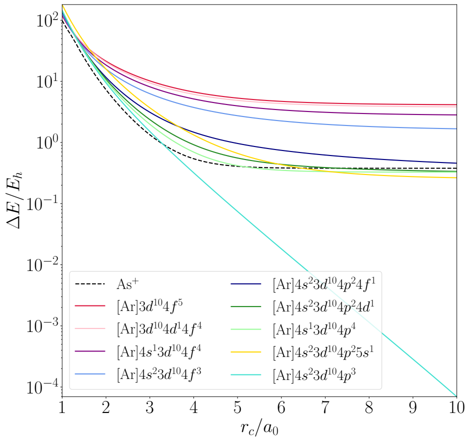

(c) r^{2}SCAN

Figure S178: Energies of various low lying configurations of hard-wall confined spin-polarized As shown as the energy difference from unconfined As as a function of the confinement radius $r_\infty = 1.0, 1.1, \ldots, 10.0a_0$. Note semilogarithmic scale.

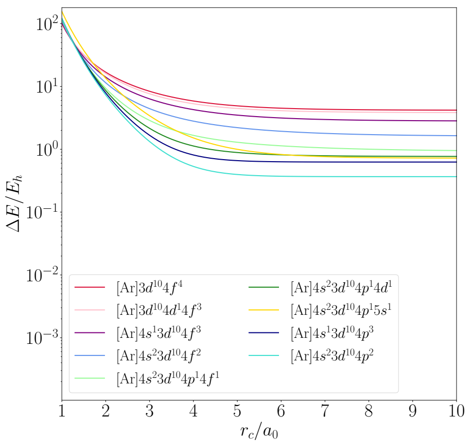

(a) PW92

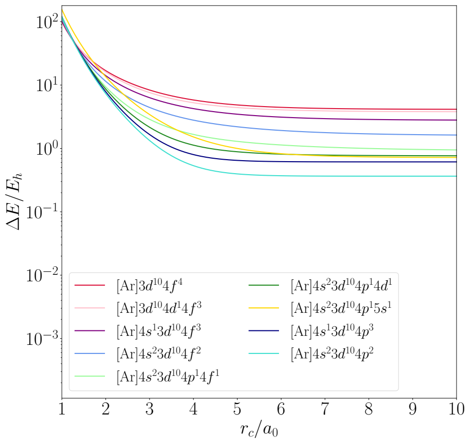

(b) PBE

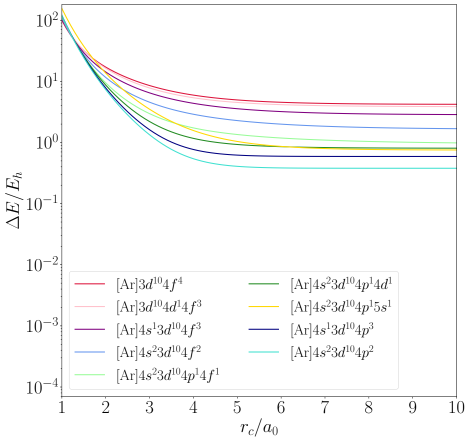

(c) r^{2}SCAN

Figure S179: Energies of various low lying configurations of the hard-wall confined spin-polarized monocation of As shown as the energy difference from unconfined As as a function of the confinement radius $r_\infty = 1.0, 1.1, \ldots, 10.0a_0$. Note semilogarithmic scale.

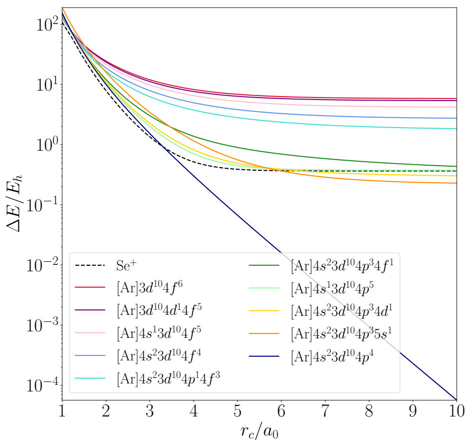

(a) PW92

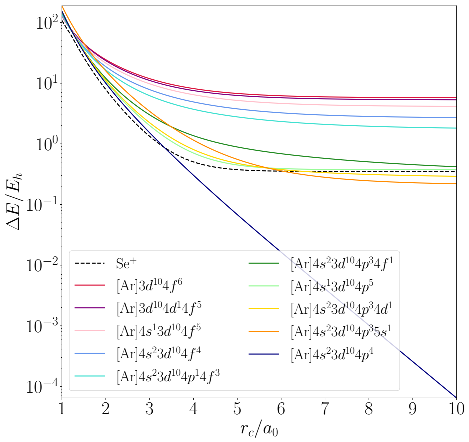

(b) PBE

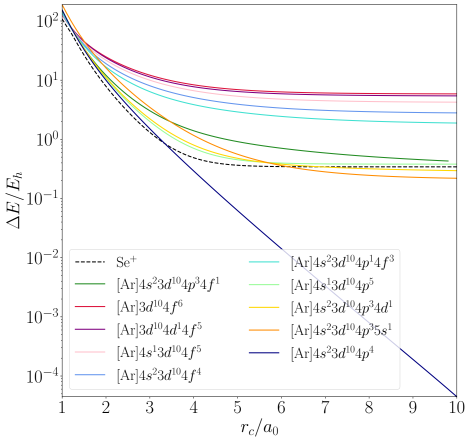

(c) r^{2}SCAN

Figure S180: Energies of various low lying configurations of hard-wall confined spin-polarized Se shown as the energy difference from unconfined Se as a function of the confinement radius $r_\infty = 1.0, 1.1, \ldots, 10.0a_0$. Note semilogarithmic scale.

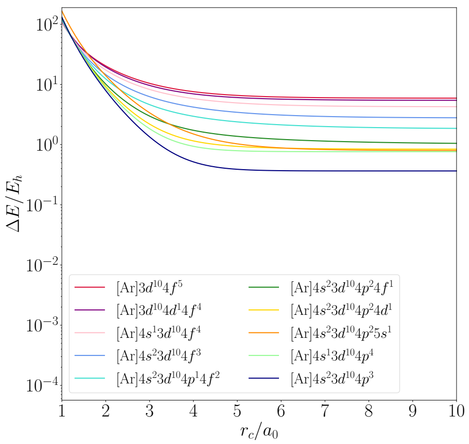

(a) PW92

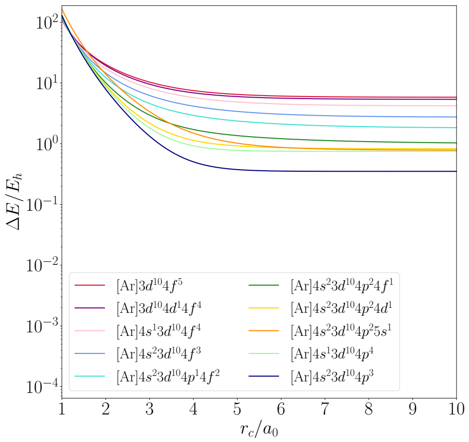

(b) PBE

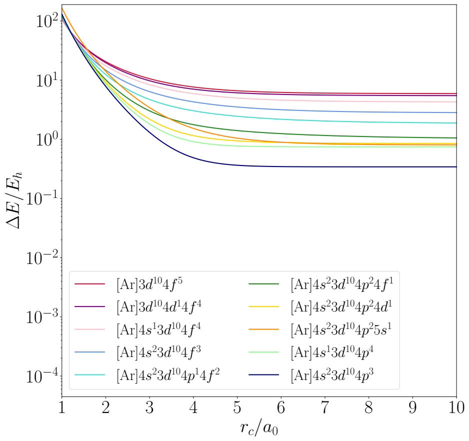

(c) r^{2}SCAN

Figure S181: Energies of various low lying configurations of the hard-wall confined spin-polarized monocation of Se shown as the energy difference from unconfined Se as a function of the confinement radius $r_\infty = 1.0, 1.1, \ldots, 10.0a_0$. Note semilogarithmic scale.

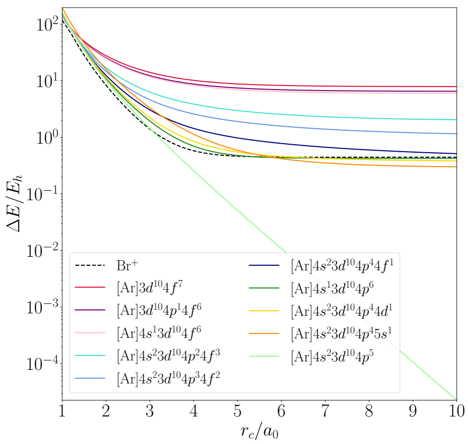

(a) PW92

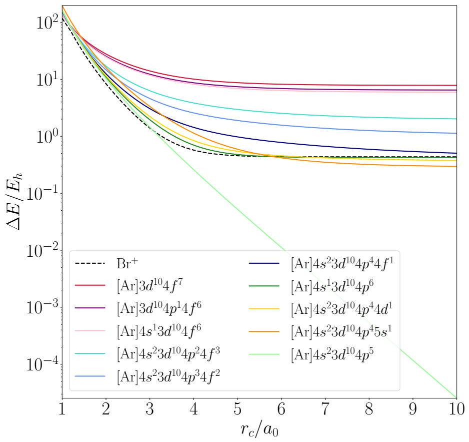

(b) PBE

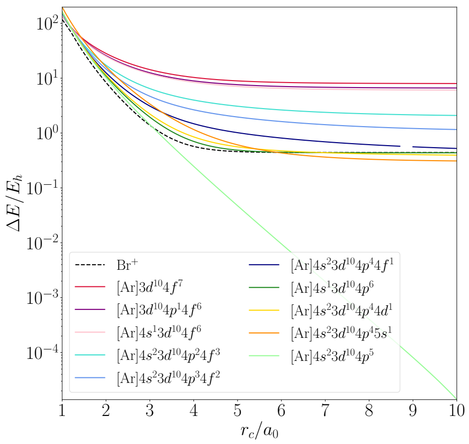

(c) r^{2}SCAN

Figure S182: Energies of various low lying configurations of hard-wall confined spin-polarized Br shown as the energy difference from unconfined Br as a function of the confinement radius $r_\infty = 1.0, 1.1, \ldots, 10.0a_0$. Note semilogarithmic scale.

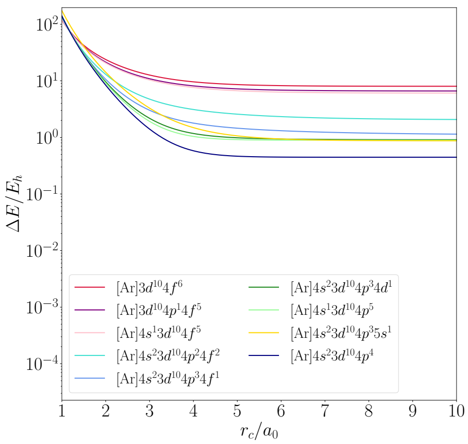

(a) PW92

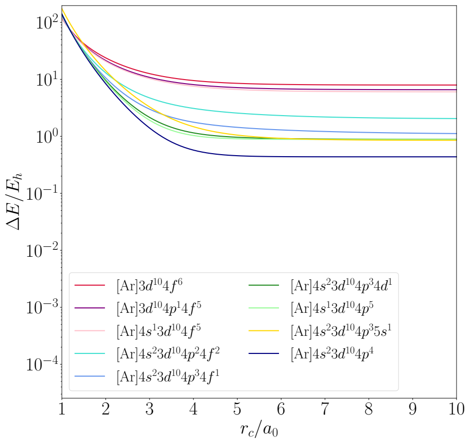

(b) PBE

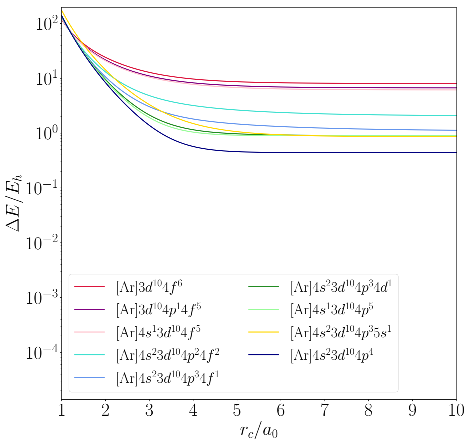

(c) r^{2}SCAN

Figure S183: Energies of various low lying configurations of the hard-wall confined spin-polarized monocation of Br shown as the energy difference from unconfined Br as a function of the confinement radius $r_\infty = 1.0, 1.1, \ldots, 10.0a_0$. Note semilogarithmic scale.

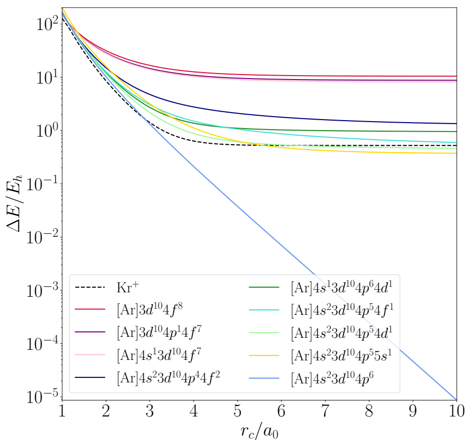

(a) PW92

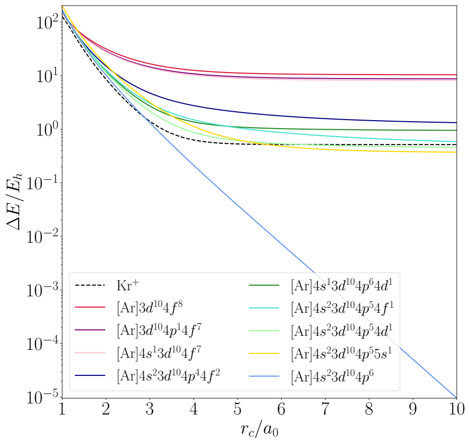

(b) PBE

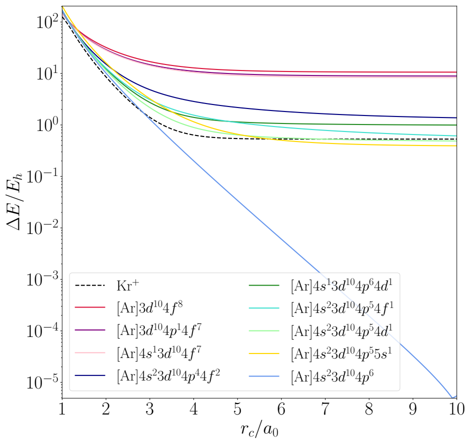

(c) r^{2}SCAN

Figure S184: Energies of various low lying configurations of hard-wall confined spin-polarized Kr shown as the energy difference from unconfined Kr as a function of the confinement radius $r_\infty = 1.0, 1.1, \ldots, 10.0 a_0$. Note semilogarithmic scale.

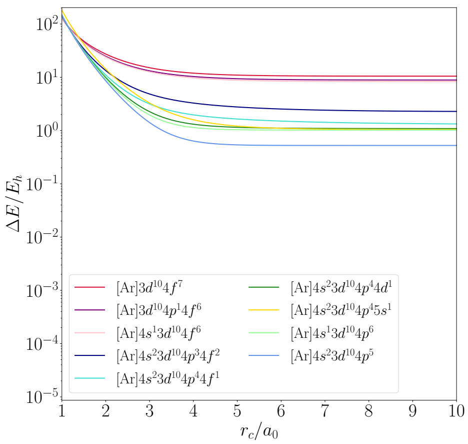

(a) PW92

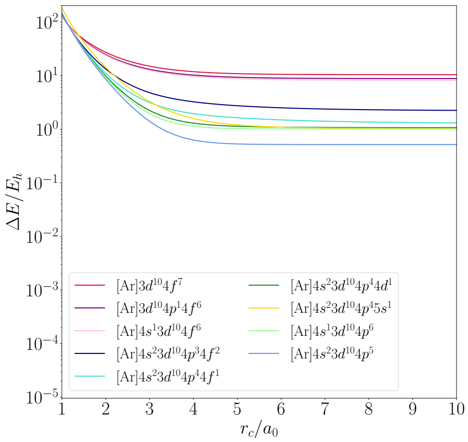

(b) PBE

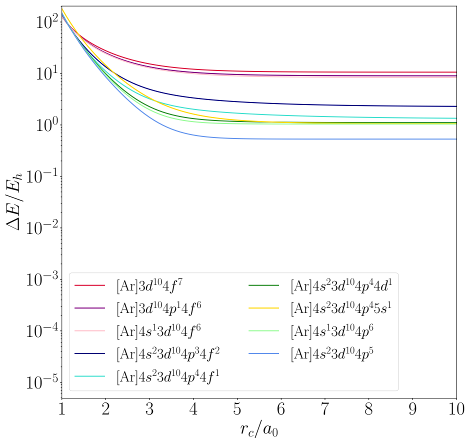

(c) r^{2}SCAN

Figure S185: Energies of various low lying configurations of the hard-wall confined spin-polarized monocation of Kr shown as the energy difference from unconfined Kr as a function of the confinement radius $r_\infty = 1.0, 1.1, \ldots, 10.0 a_0$. Note semilogarithmic scale.

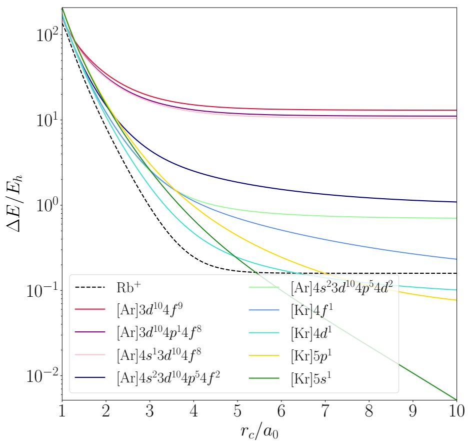

(a) PW92

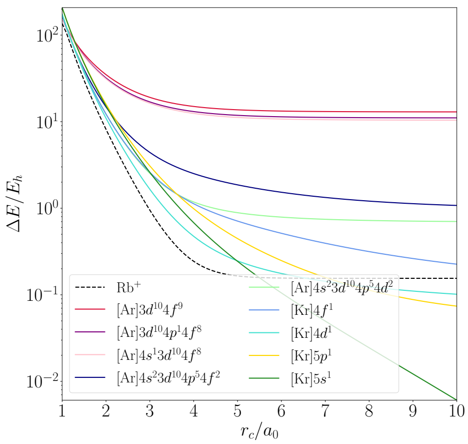

(b) PBE

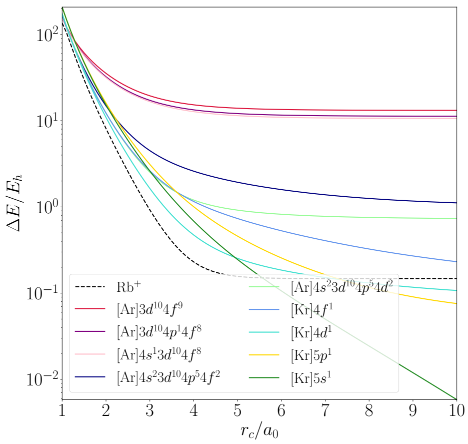

(c) r^{2}SCAN

Figure S186: Energies of various low lying configurations of hard-wall confined spin-polarized Rb shown as the energy difference from unconfined Rb as a function of the confinement radius $r_\infty = 1.0, 1.1, \ldots, 10.0a_0$. Note semilogarithmic scale.

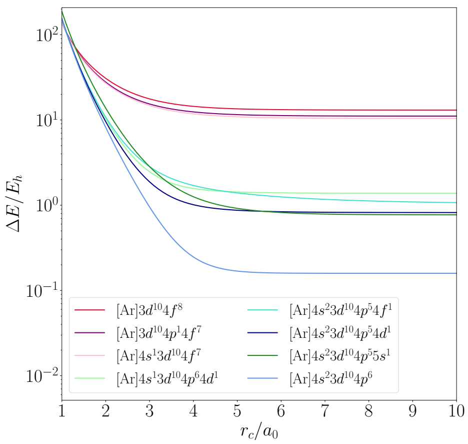

(a) PW92

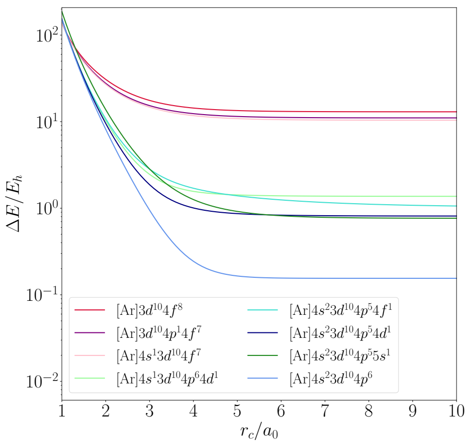

(b) PBE

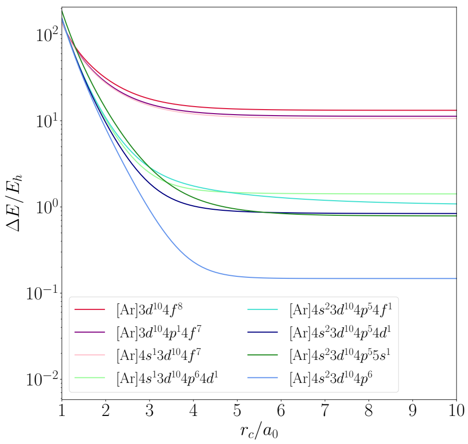

(c) r^{2}SCAN

Figure S187: Energies of various low lying configurations of the hard-wall confined spin-polarized monocation of Rb shown as the energy difference from unconfined Rb as a function of the confinement radius $r_\infty = 1.0, 1.1, \ldots, 10.0a_0$. Note semilogarithmic scale.

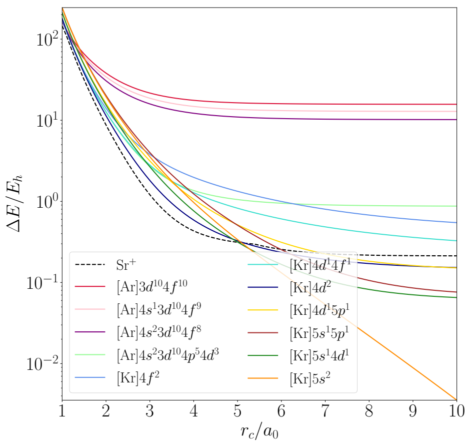

(a) PW92

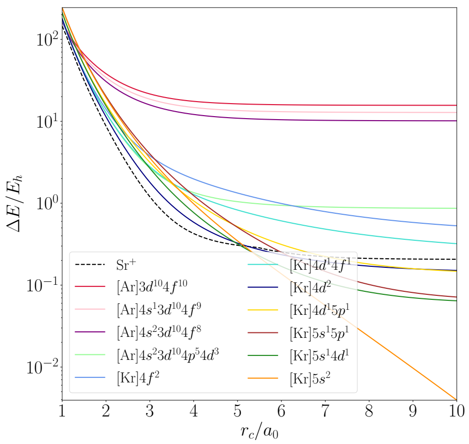

(b) PBE

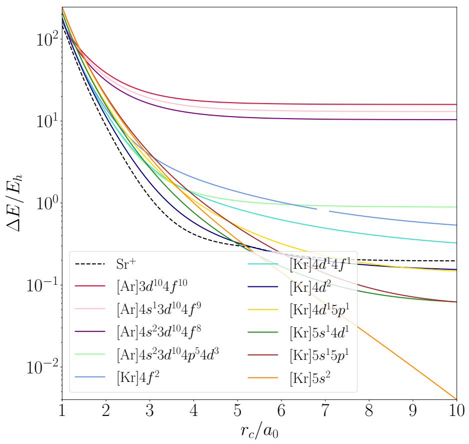

(c) r^{2}SCAN

Figure S188: Energies of various low lying configurations of hard-wall confined spin-polarized Sr shown as the energy difference from unconfined Sr as a function of the confinement radius $r_\infty = 1.0, 1.1, \ldots, 10.0a_0$. Note semilogarithmic scale.

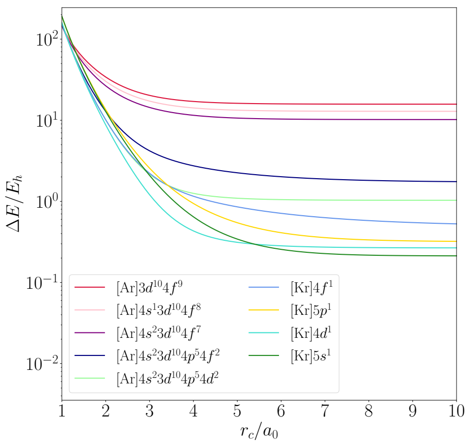

(a) PW92

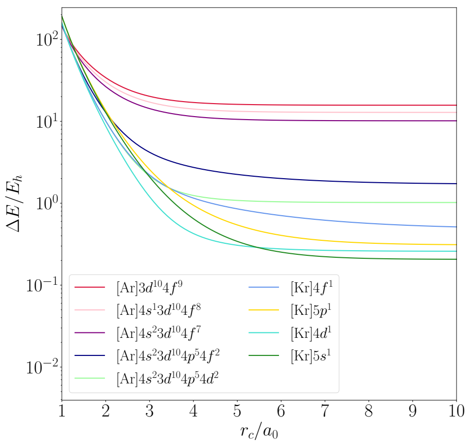

(b) PBE

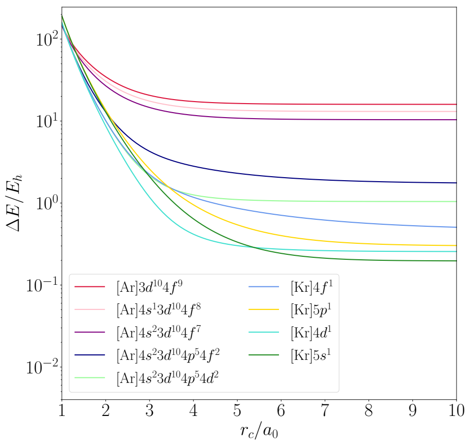

(c) r^{2}SCAN

Figure S189: Energies of various low lying configurations of the hard-wall confined spin-polarized monocation of Sr shown as the energy difference from unconfined Sr as a function of the confinement radius $r_\infty = 1.0, 1.1, \ldots, 10.0a_0$. Note semilogarithmic scale.

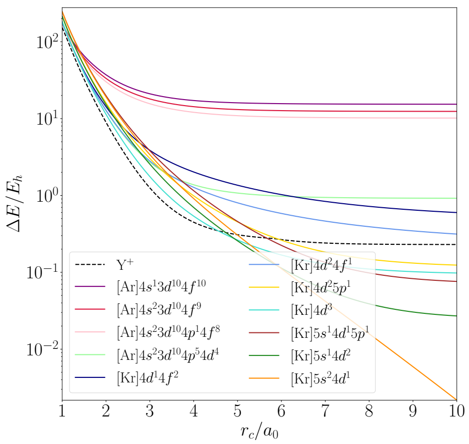

(a) PW92

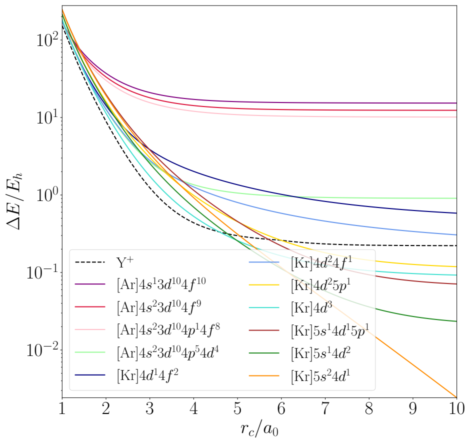

(b) PBE

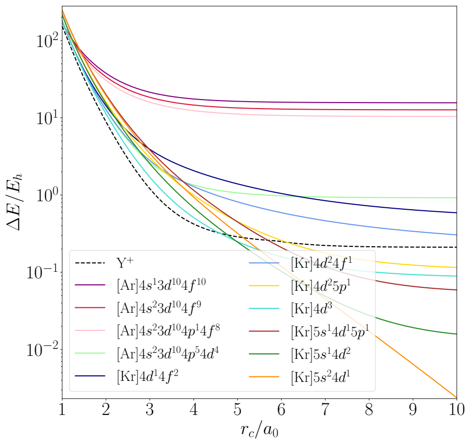

(c) r^{2}SCAN

Figure S190: Energies of various low lying configurations of hard-wall confined spin-polarized Y shown as the energy difference from unconfined Y as a function of the confinement radius $r_\infty = 1.0, 1.1, \ldots, 10.0 a_0$. Note semilogarithmic scale.

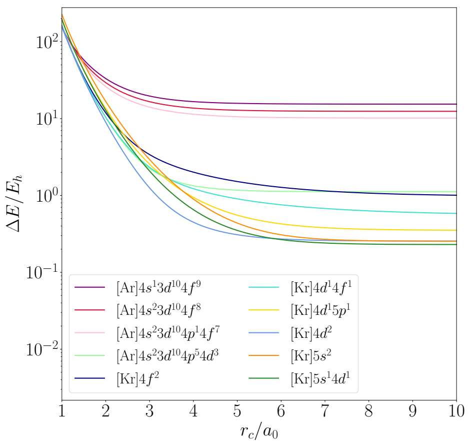

(a) PW92

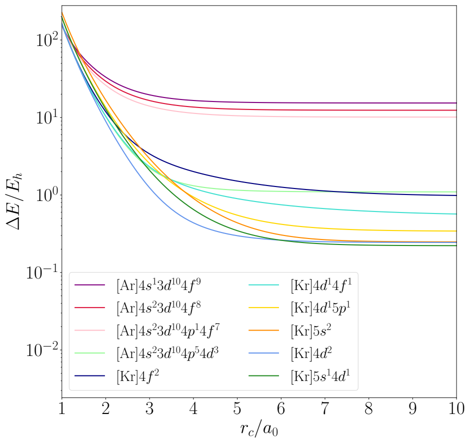

(b) PBE

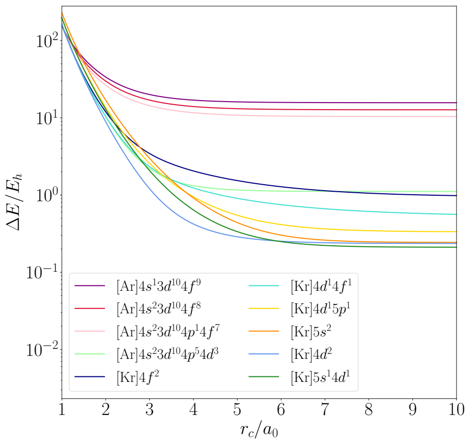

(c) r^{2}SCAN

Figure S191: Energies of various low lying configurations of the hard-wall confined spin-polarized monocation of Y shown as the energy difference from unconfined Y as a function of the confinement radius $r_\infty = 1.0, 1.1, \ldots, 10.0 a_0$. Note semilogarithmic scale.

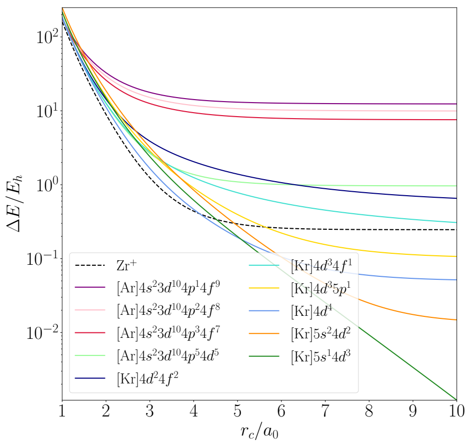

(a) PW92

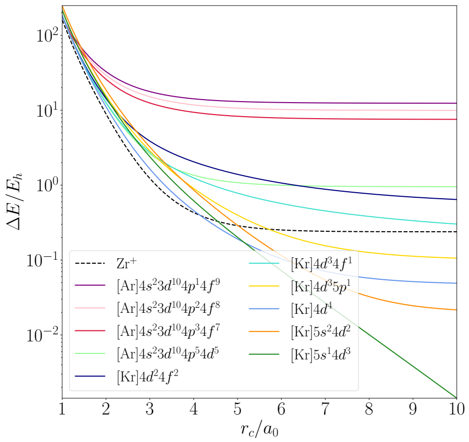

(b) PBE

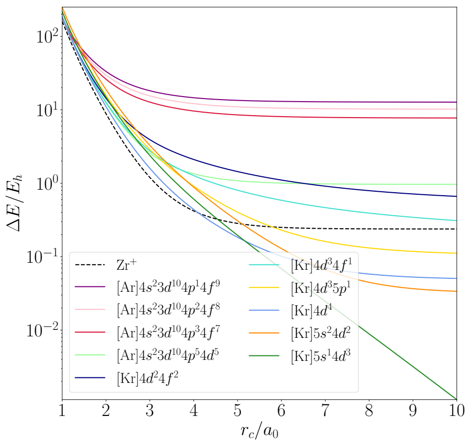

(c) r^{2}SCAN

Figure S192: Energies of various low lying configurations of hard-wall confined spin-polarized Zr shown as the energy difference from unconfined Zr as a function of the confinement radius $r_\infty = 1.0, 1.1, \ldots, 10.0a_0$. Note semilogarithmic scale.

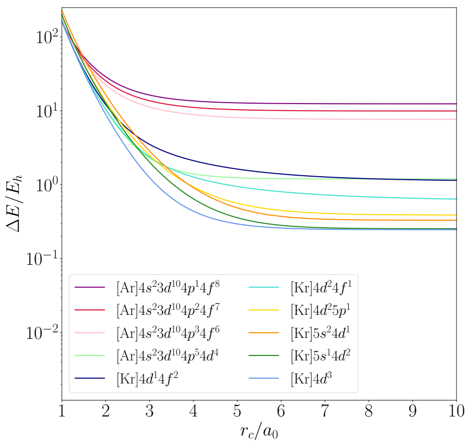

(a) PW92

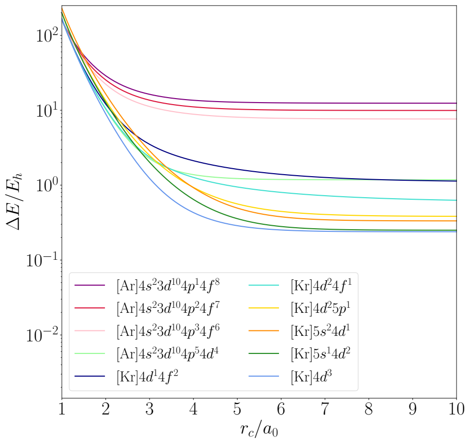

(b) PBE

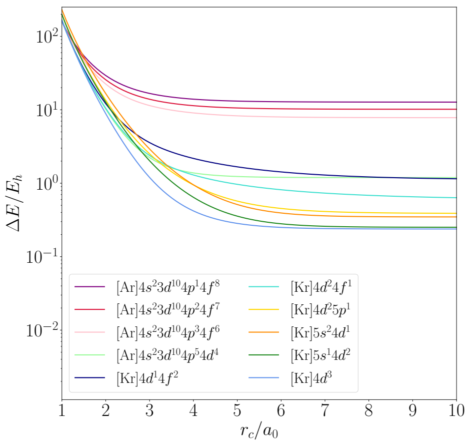

(c) r^{2}SCAN

Figure S193: Energies of various low lying configurations of the hard-wall confined spin-polarized monocation of Zr shown as the energy difference from unconfined Zr as a function of the confinement radius $r_\infty = 1.0, 1.1, \ldots, 10.0a_0$. Note semilogarithmic scale.

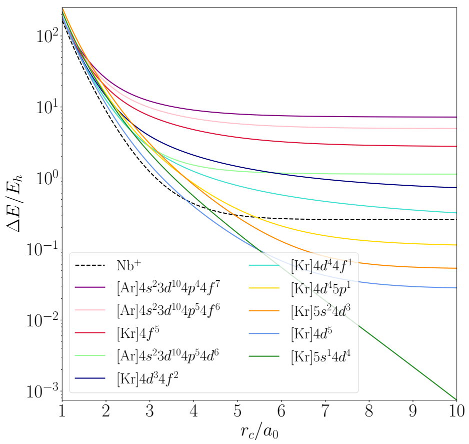

(a) PW92

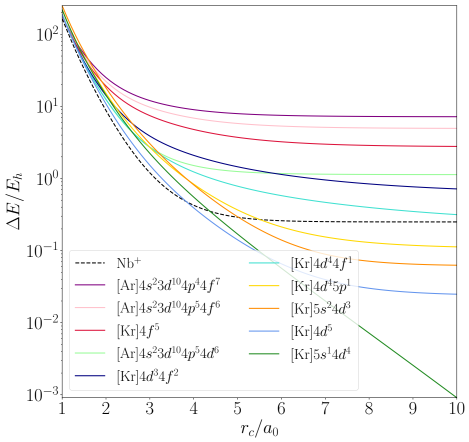

(b) PBE

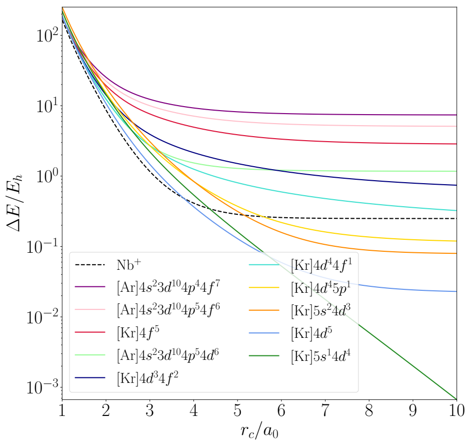

(c) r^{2}SCAN

Figure S194: Energies of various low lying configurations of hard-wall confined spin-polarized Nb shown as the energy difference from unconfined Nb as a function of the confinement radius $r_\infty = 1.0, 1.1, \ldots, 10.0a_0$. Note semilogarithmic scale.

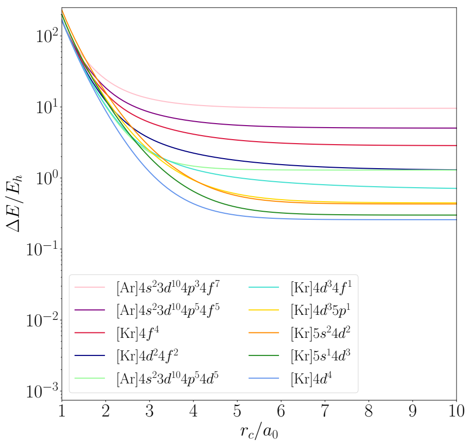

(a) PW92

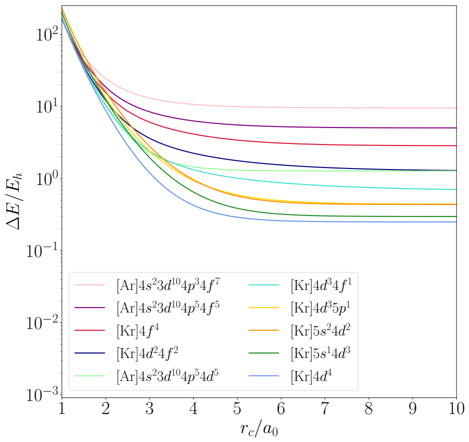

(b) PBE

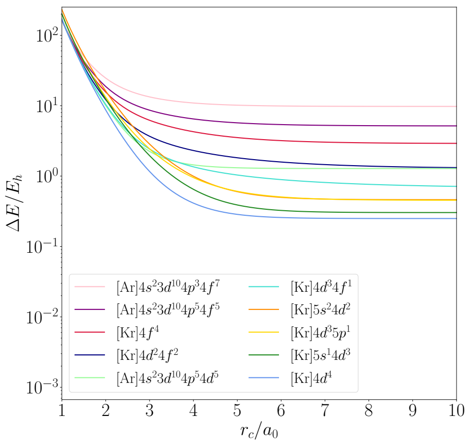

(c) r^{2}SCAN

Figure S195: Energies of various low lying configurations of the hard-wall confined spin-polarized monocation of Nb shown as the energy difference from unconfined Nb as a function of the confinement radius $r_\infty = 1.0, 1.1, \ldots, 10.0a_0$. Note semilogarithmic scale.

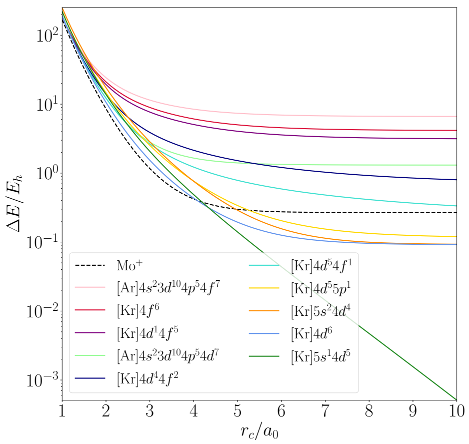

(a) PW92

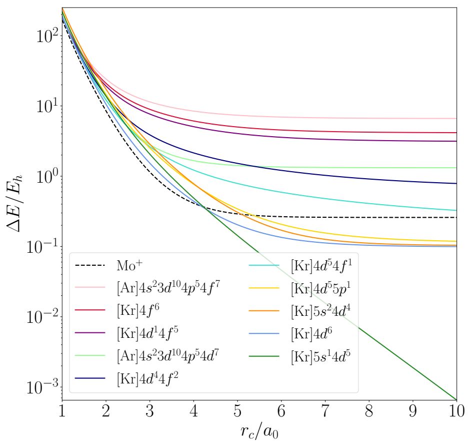

(b) PBE

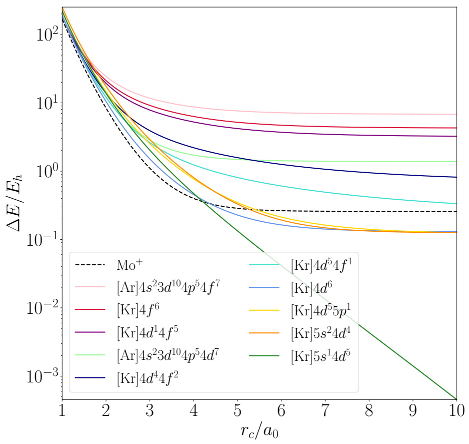

(c) r^{2}SCAN

Figure S196: Energies of various low lying configurations of hard-wall confined spin-polarized Mo shown as the energy difference from unconfined Mo as a function of the confinement radius $r_\infty = 1.0, 1.1, \ldots, 10.0a_0$. Note semilogarithmic scale.

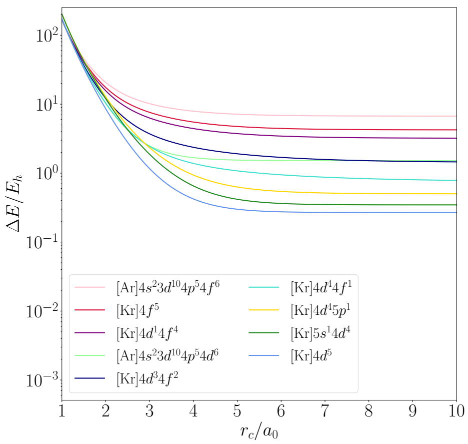

(a) PW92

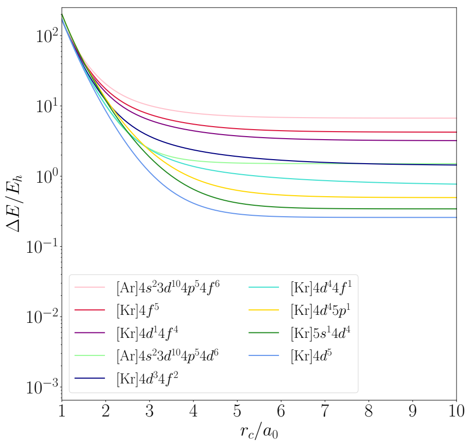

(b) PBE

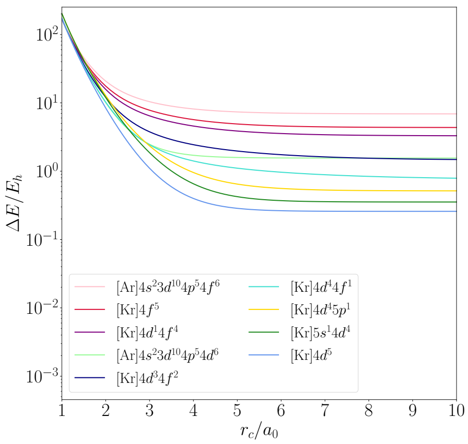

(c) r^{2}SCAN

Figure S197: Energies of various low lying configurations of the hard-wall confined spin-polarized monocation of Mo shown as the energy difference from unconfined Mo as a function of the confinement radius $r_\infty = 1.0, 1.1, \ldots, 10.0a_0$. Note semilogarithmic scale.

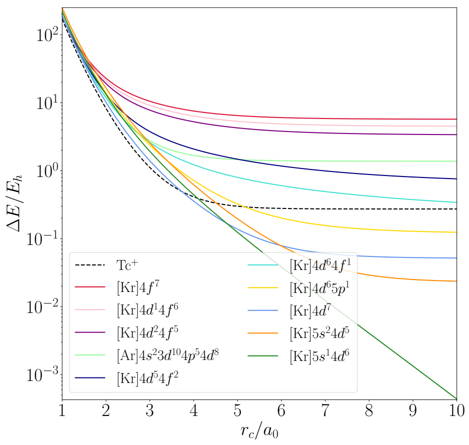

(a) PW92

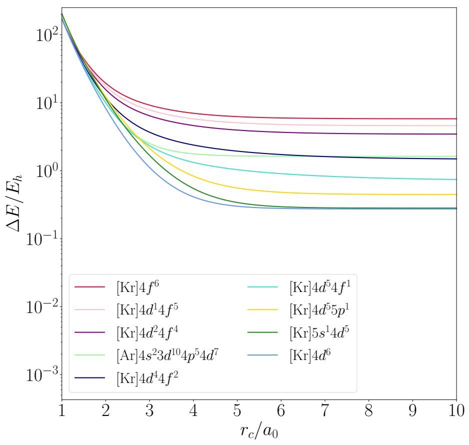

(a) PW92

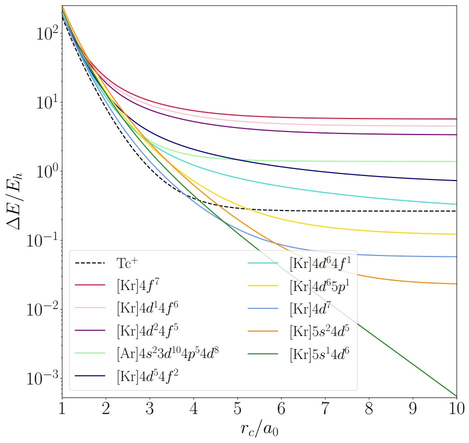

(b) PBE

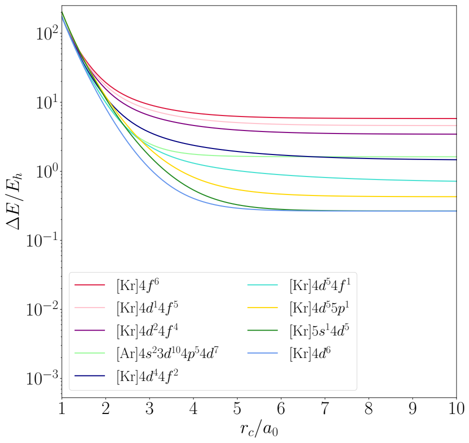

(b) PBE

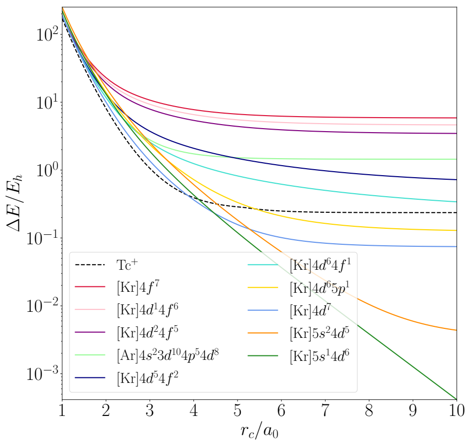

(c) r^{2}SCAN

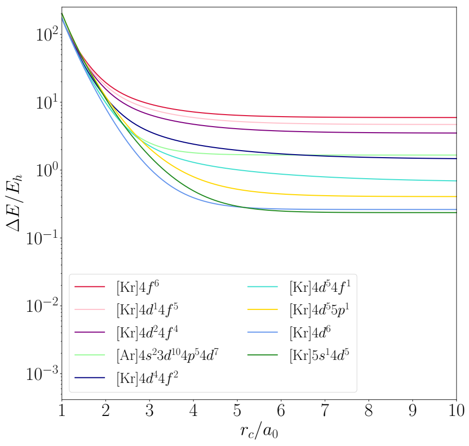

(c) r^{2}SCAN

Figure S198: Energies of various low lying configurations of hard-wall confined spin-polarized Tc shown as the energy difference from unconfined Tc as a function of the confinement radius $r_\infty = 1.0, 1.1, \dots, 10.0a_0$. Note semilogarithmic scale.

Figure S199: Energies of various low lying configurations of the hard-wall confined spin-polarized monocation of Tc shown as the energy difference from unconfined Tc as a function of the confinement radius $r_\infty = 1.0, 1.1, \dots, 10.0a_0$. Note semilogarithmic scale.

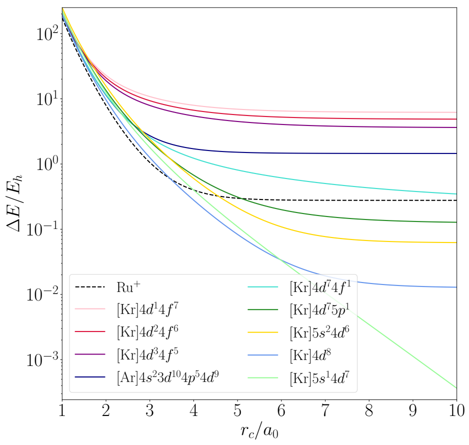

(a) PW92

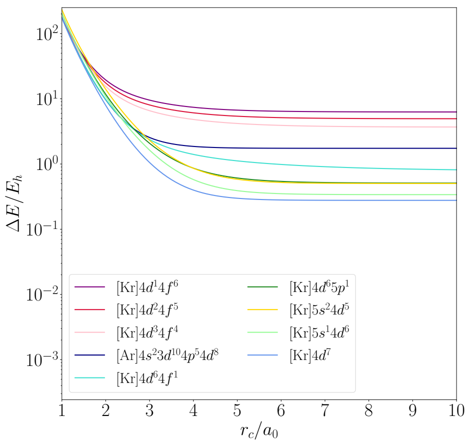

(a) PW92

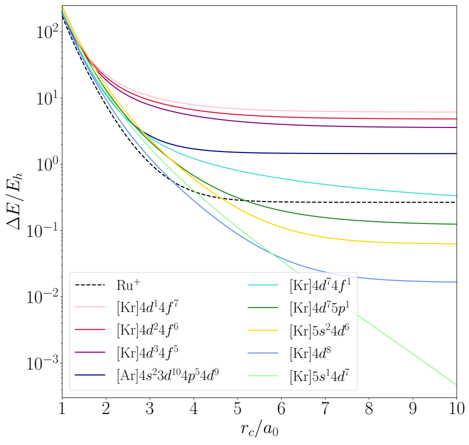

(b) PBE

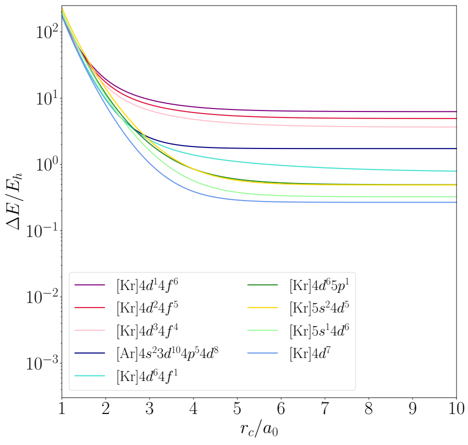

(b) PBE

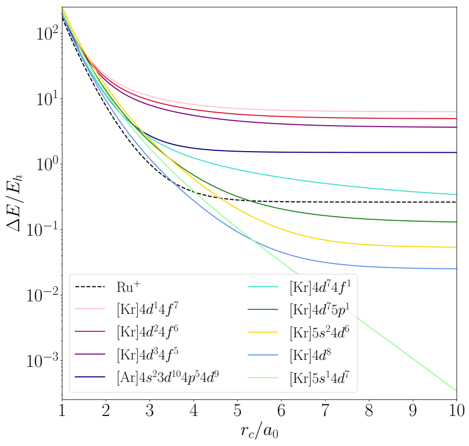

(c) r^{2}SCAN

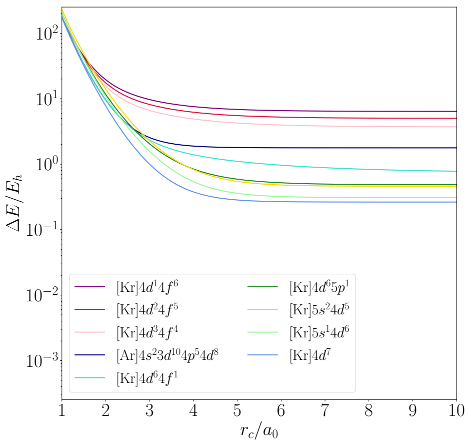

(c) r^{2}SCAN

Figure S200: Energies of various low lying configurations of hard-wall confined spin-polarized Ru shown as the energy difference from unconfined Ru as a function of the confinement radius $r_\infty = 1.0, 1.1, \ldots, 10.0 a_0$. Note semilogarithmic scale.

Figure S201: Energies of various low lying configurations of the hard-wall confined spin-polarized monocation of Ru shown as the energy difference from unconfined Ru as a function of the confinement radius $r_\infty = 1.0, 1.1, \ldots, 10.0 a_0$. Note semilogarithmic scale.

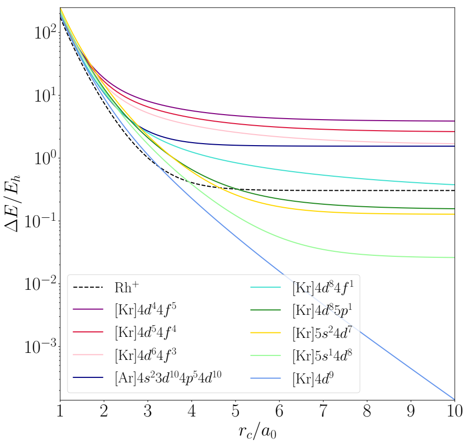

(a) PW92

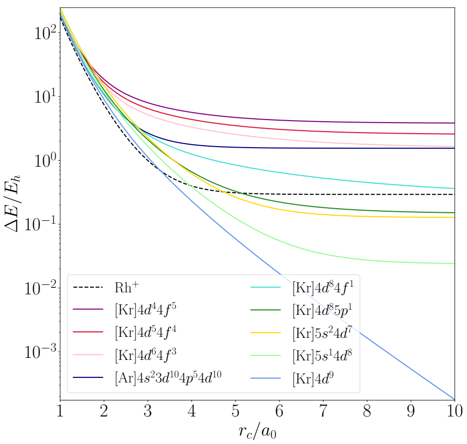

(b) PBE

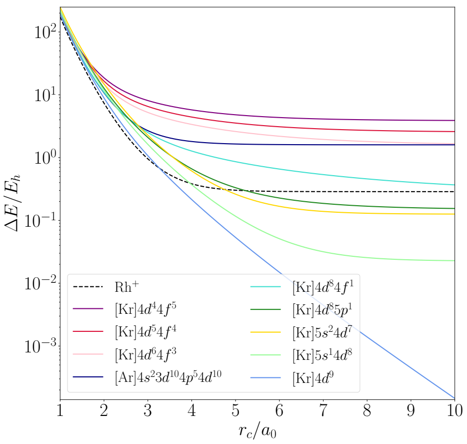

(c) r^{2}SCAN

Figure S202: Energies of various low lying configurations of hard-wall confined spin-polarized Rh shown as the energy difference from unconfined Rh as a function of the confinement radius $r_\infty = 1.0, 1.1, \ldots, 10.0a_0$. Note semilogarithmic scale.

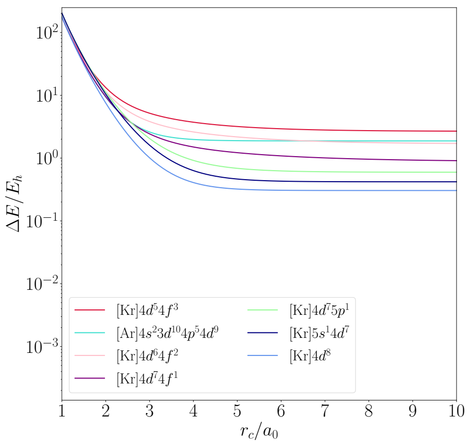

(a) PW92

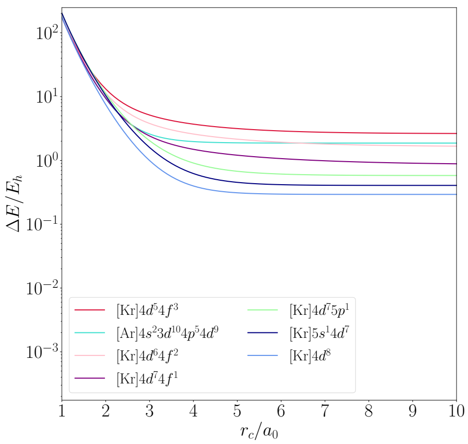

(b) PBE

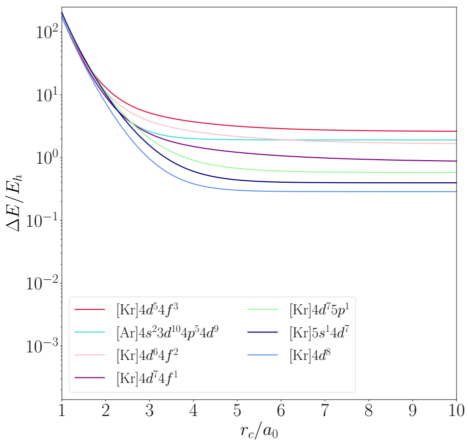

(c) r^{2}SCAN

Figure S203: Energies of various low lying configurations of the hard-wall confined spin-polarized monocation of Rh shown as the energy difference from unconfined Rh as a function of the confinement radius $r_\infty = 1.0, 1.1, \ldots, 10.0a_0$. Note semilogarithmic scale.

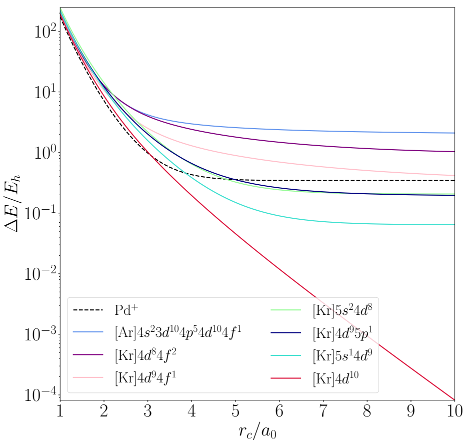

(a) PW92

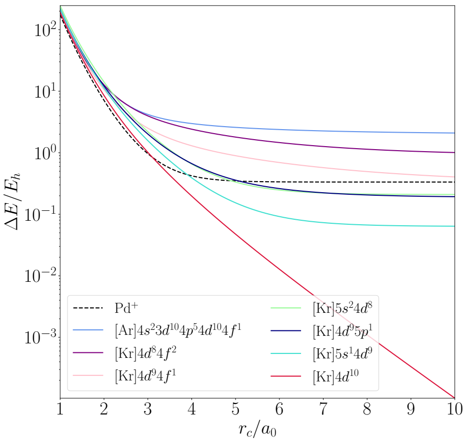

(b) PBE

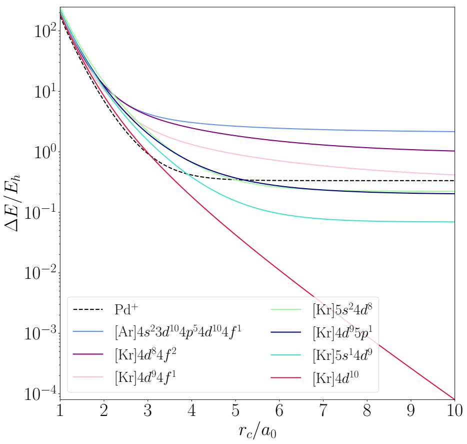

(c) r^{2}SCAN

Figure S204: Energies of various low lying configurations of hard-wall confined spin-polarized Pd shown as the energy difference from unconfined Pd as a function of the confinement radius $r_\infty = 1.0, 1.1, \dots, 10.0 a_0$. Note semilogarithmic scale.

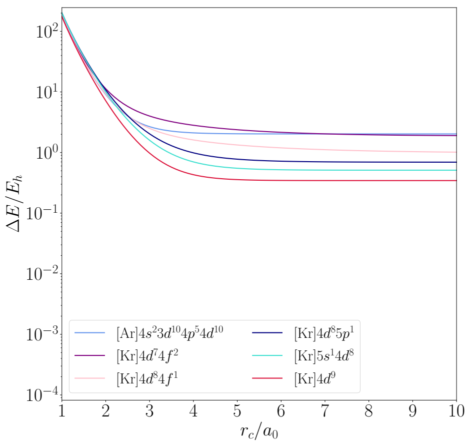

(a) PW92

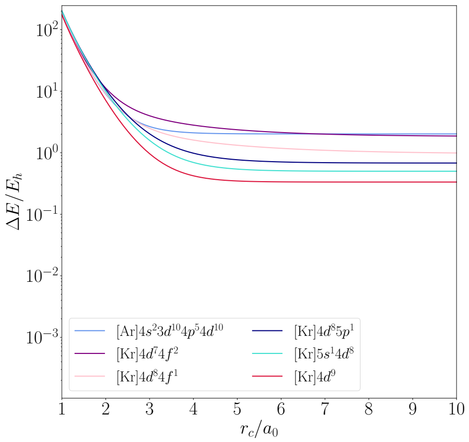

(b) PBE

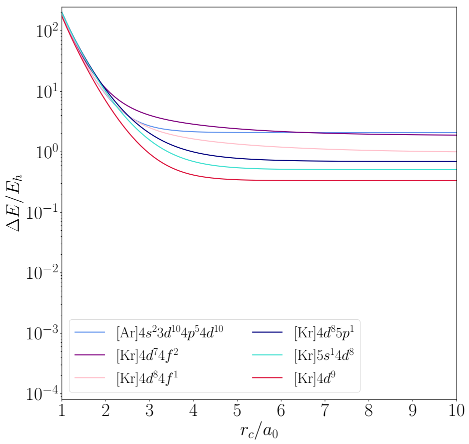

(c) r^{2}SCAN

Figure S205: Energies of various low lying configurations of the hard-wall confined spin-polarized monocation of Pd shown as the energy difference from unconfined Pd as a function of the confinement radius $r_\infty = 1.0, 1.1, \dots, 10.0 a_0$. Note semilogarithmic scale.

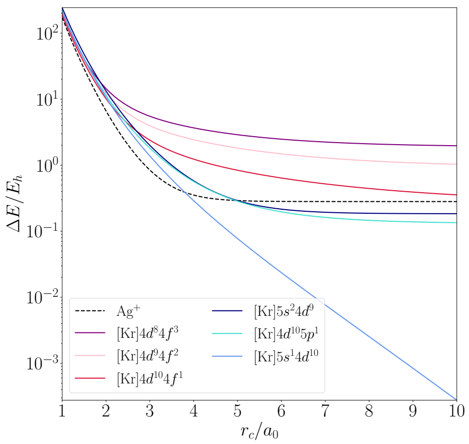

(a) PW92

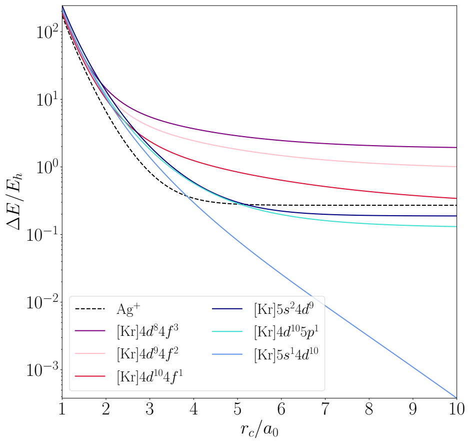

(b) PBE

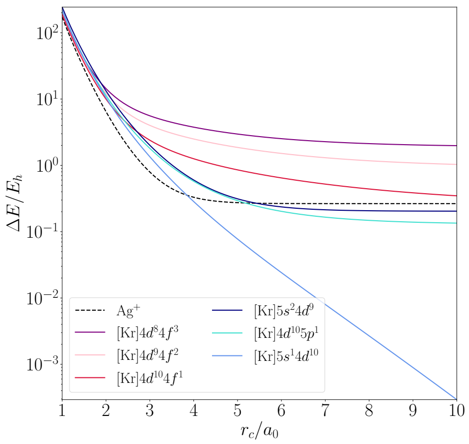

(c) r^{2}SCAN

Figure S206: Energies of various low lying configurations of hard-wall confined spin-polarized Ag shown as the energy difference from unconfined Ag as a function of the confinement radius $r_\infty = 1.0, 1.1, \ldots, 10.0a_0$. Note semilogarithmic scale.

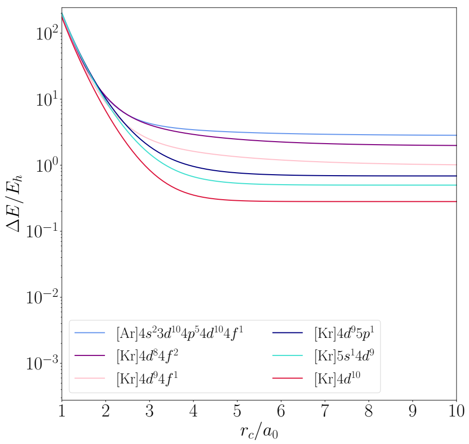

(a) PW92

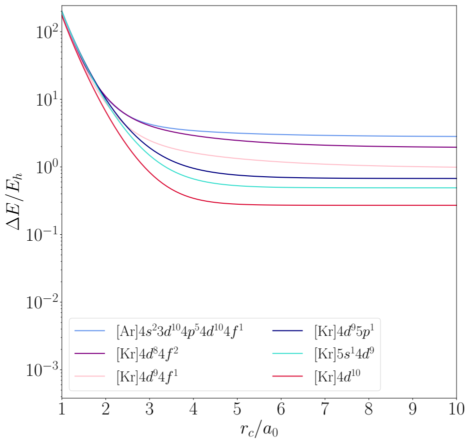

(b) PBE

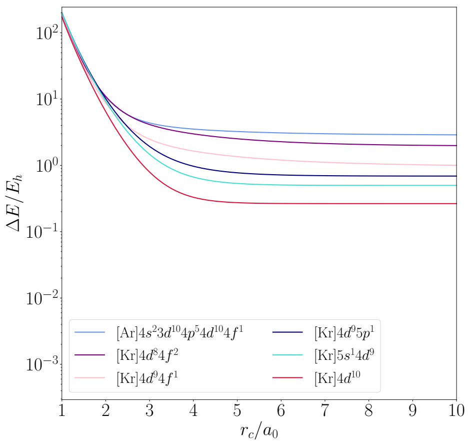

(c) r^{2}SCAN

Figure S207: Energies of various low lying configurations of the hard-wall confined spin-polarized monocation of Ag shown as the energy difference from unconfined Ag as a function of the confinement radius $r_\infty = 1.0, 1.1, \ldots, 10.0a_0$. Note semilogarithmic scale.

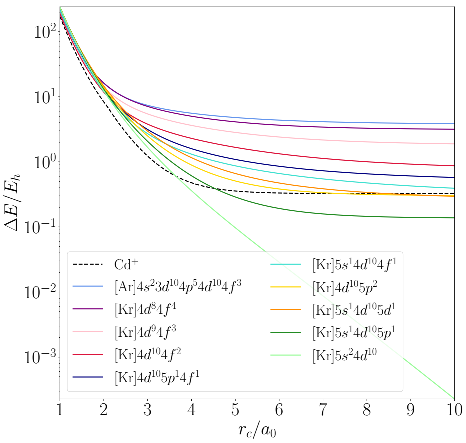

(a) PW92

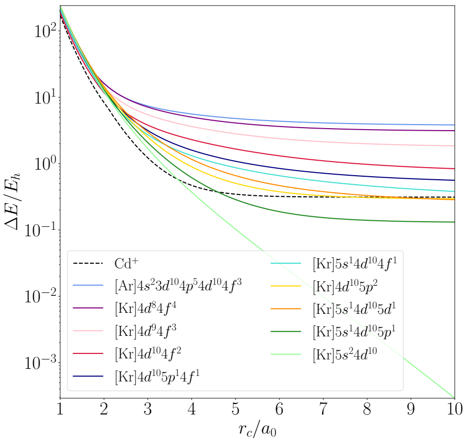

(b) PBE

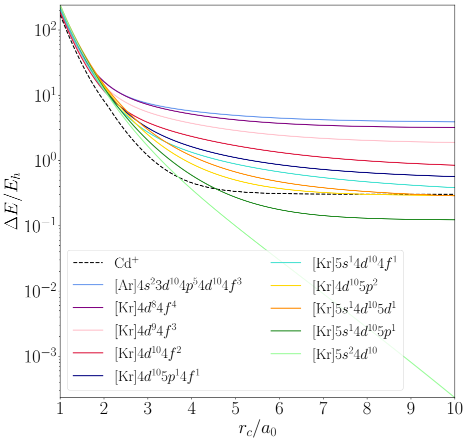

(c) r^{2}SCAN

Figure S208: Energies of various low lying configurations of hard-wall confined spin-polarized Cd shown as the energy difference from unconfined Cd as a function of the confinement radius $r_\infty = 1.0, 1.1, \ldots, 10.0a_0$. Note semilogarithmic scale.

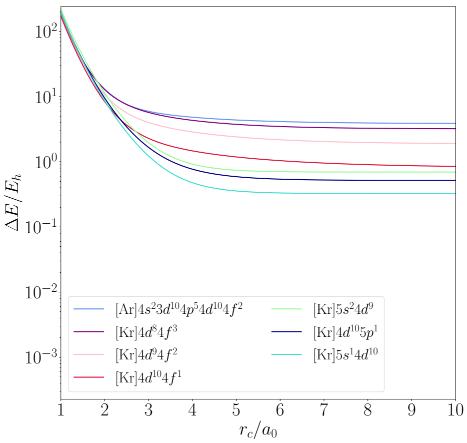

(a) PW92

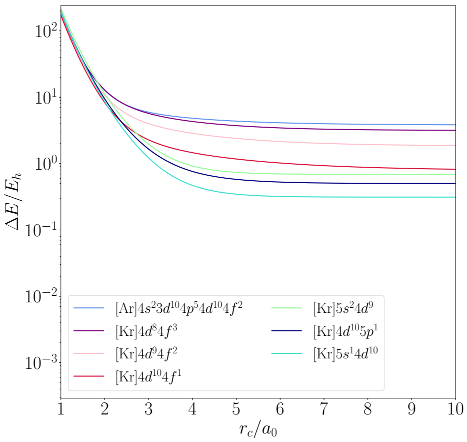

(b) PBE

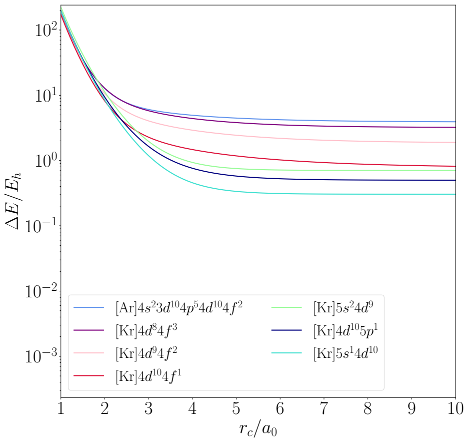

(c) r^{2}SCAN

Figure S209: Energies of various low lying configurations of the hard-wall confined spin-polarized monocation of Cd shown as the energy difference from unconfined Cd as a function of the confinement radius $r_\infty = 1.0, 1.1, \ldots, 10.0a_0$. Note semilogarithmic scale.

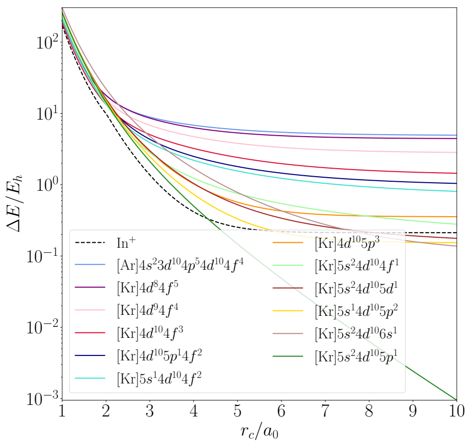

(a) PW92

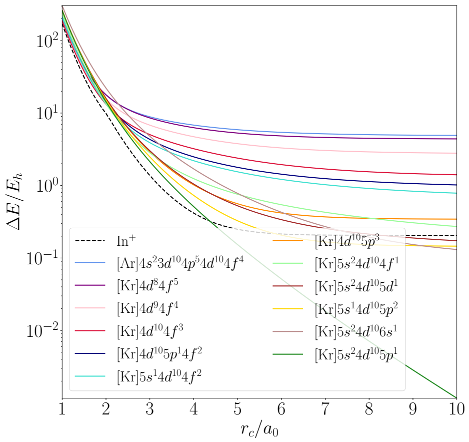

(b) PBE

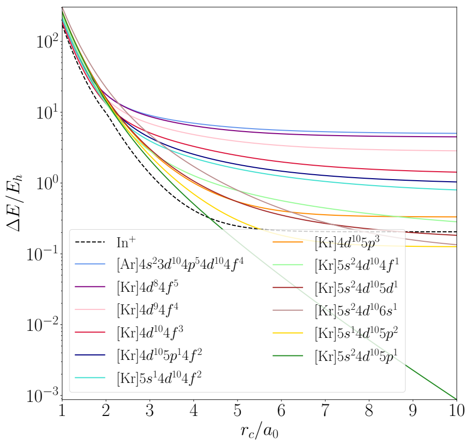

(c) r^{2}SCAN

Figure S210: Energies of various low lying configurations of hard-wall confined spin-polarized In shown as the energy difference from unconfined In as a function of the confinement radius $r_\infty = 1.0, 1.1, \ldots, 10.0a_0$. Note semilogarithmic scale.

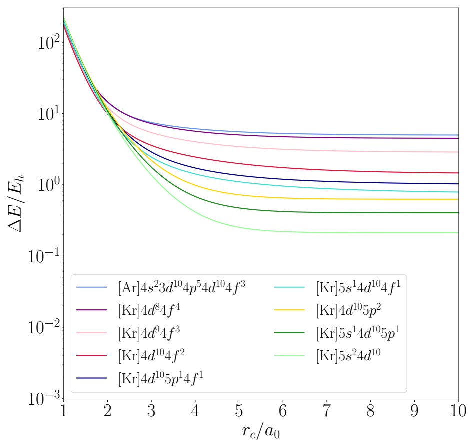

(a) PW92

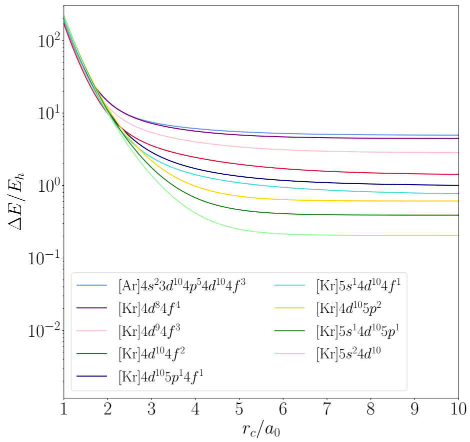

(b) PBE

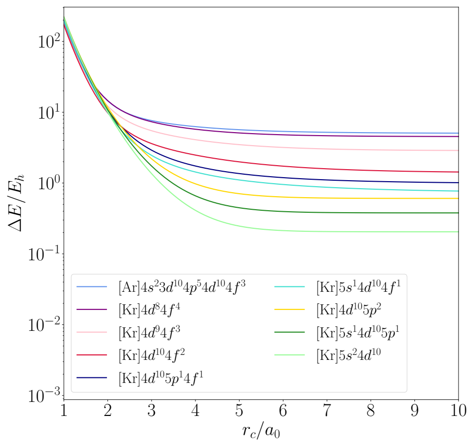

(c) r^{2}SCAN

Figure S211: Energies of various low lying configurations of the hard-wall confined spin-polarized monocation of In shown as the energy difference from unconfined In as a function of the confinement radius $r_\infty = 1.0, 1.1, \ldots, 10.0a_0$. Note semilogarithmic scale.

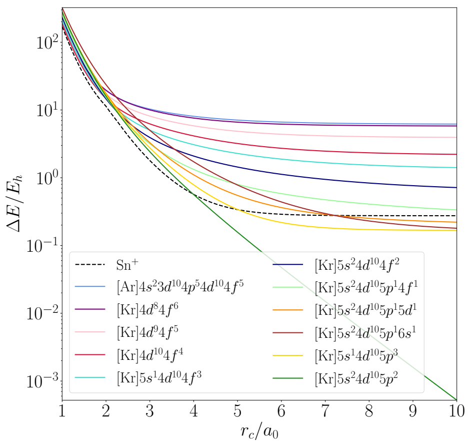

(a) PW92

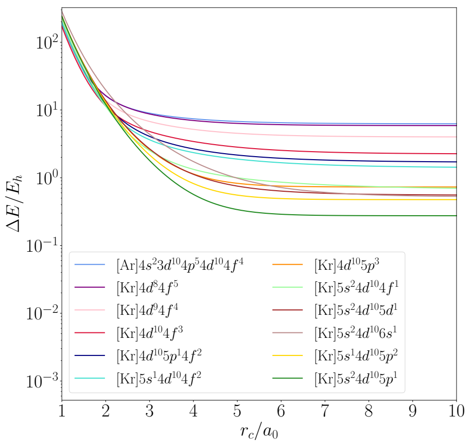

(a) PW92

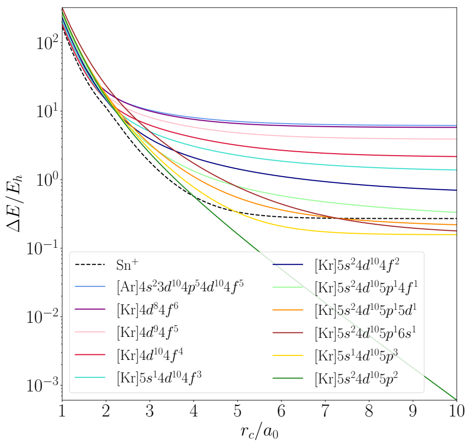

(b) PBE

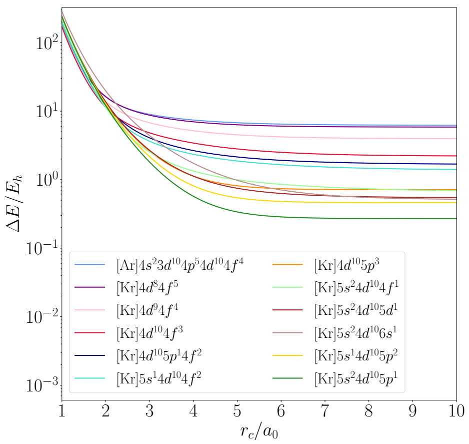

(b) PBE

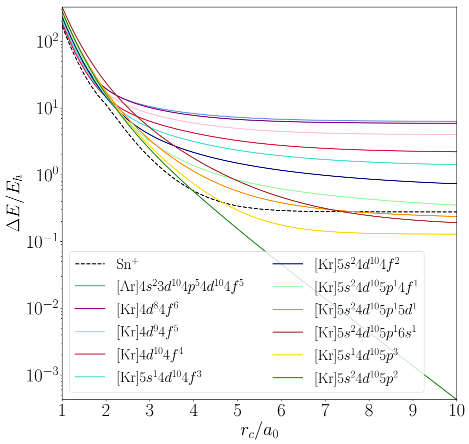

(c) r^{2}SCAN

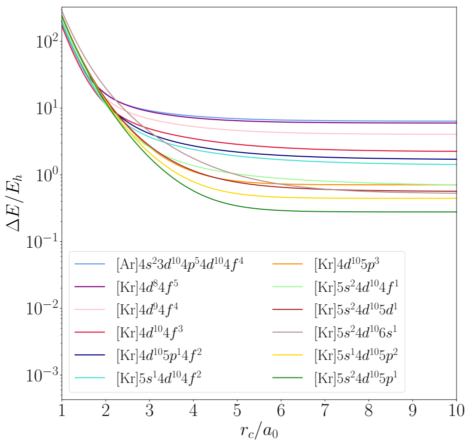

(c) r^{2}SCAN

Figure S212: Energies of various low lying configurations of hard-wall confined spin-polarized Sn shown as the energy difference from unconfined Sn as a function of the confinement radius $r_\infty = 1.0, 1.1, \ldots, 10.0 a_0$. Note semilogarithmic scale.

Figure S213: Energies of various low lying configurations of the hard-wall confined spin-polarized monocation of Sn shown as the energy difference from unconfined Sn as a function of the confinement radius $r_\infty = 1.0, 1.1, \ldots, 10.0 a_0$. Note semilogarithmic scale.

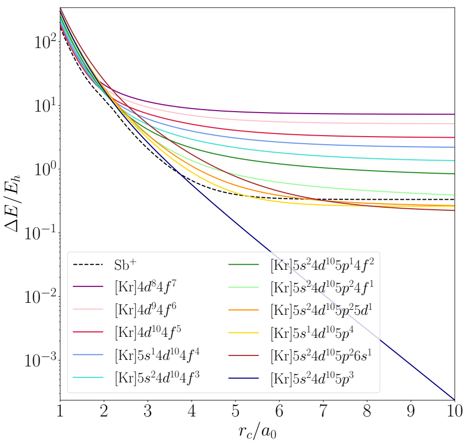

(a) PW92

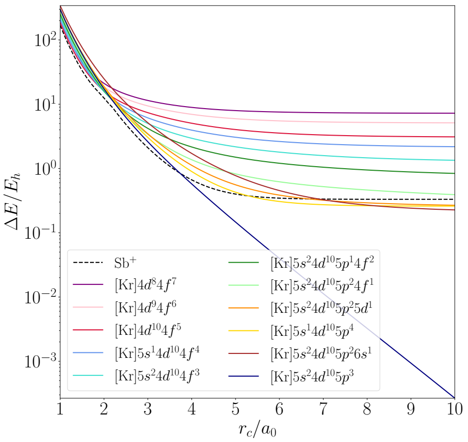

(b) PBE

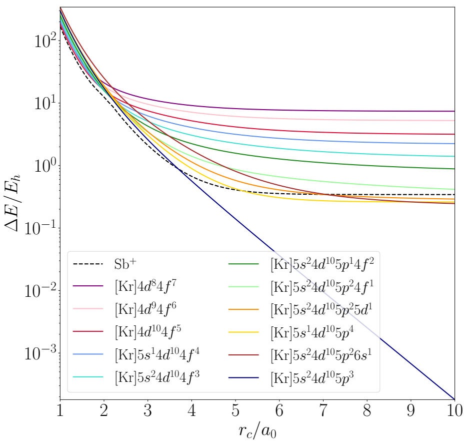

(c) r^{2}SCAN

Figure S214: Energies of various low lying configurations of hard-wall confined spin-polarized Sb shown as the energy difference from unconfined Sb as a function of the confinement radius $r_\infty = 1.0, 1.1, \ldots, 10.0 a_0$. Note semilogarithmic scale.

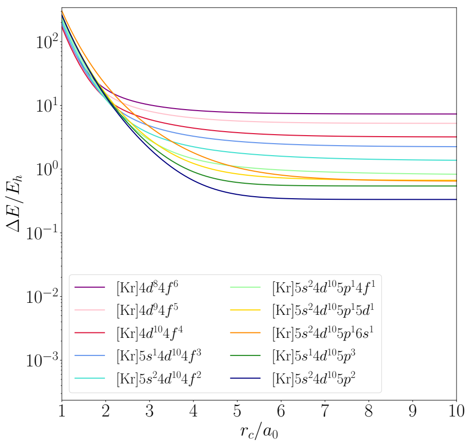

(a) PW92

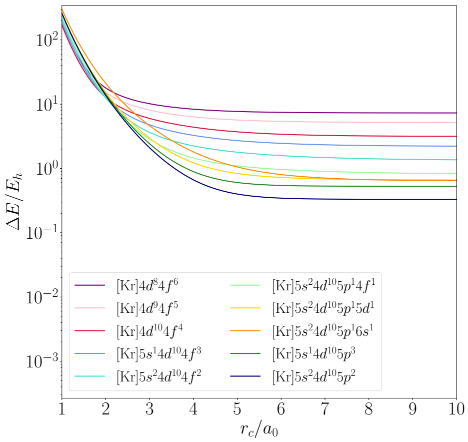

(b) PBE

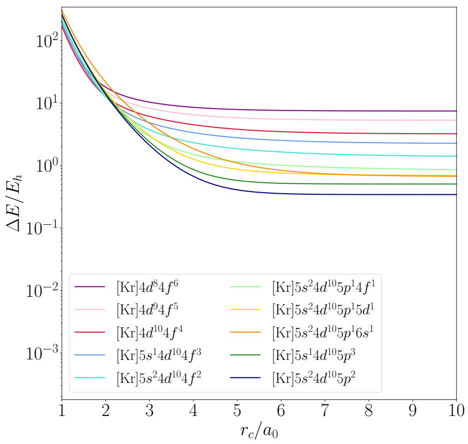

(c) r^{2}SCAN

Figure S215: Energies of various low lying configurations of the hard-wall confined spin-polarized monocation of Sb shown as the energy difference from unconfined Sb as a function of the confinement radius $r_\infty = 1.0, 1.1, \ldots, 10.0 a_0$. Note semilogarithmic scale.

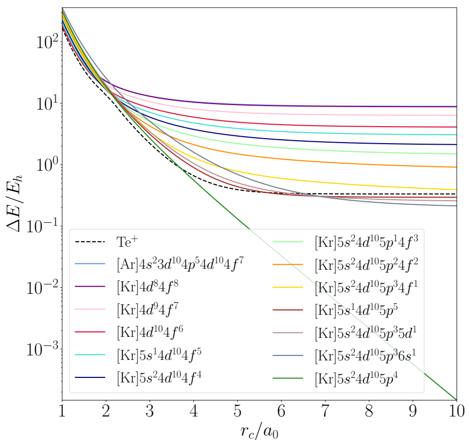

(a) PW92

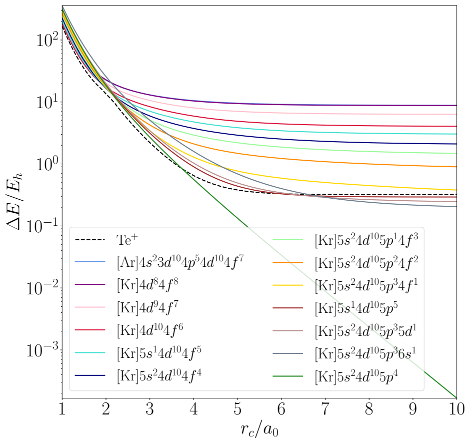

(b) PBE

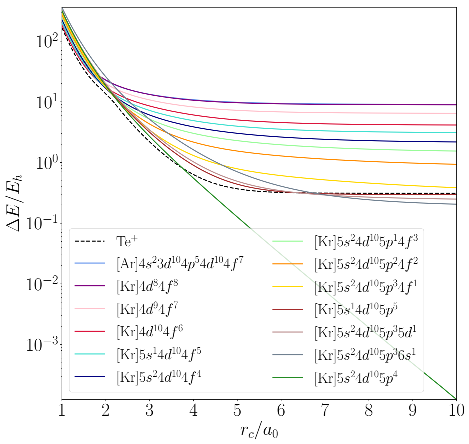

(c) r^{2}SCAN

Figure S216: Energies of various low lying configurations of hard-wall confined spin-polarized Te shown as the energy difference from unconfined Te as a function of the confinement radius $r_\infty = 1.0, 1.1, \ldots, 10.0 a_0$. Note semilogarithmic scale.

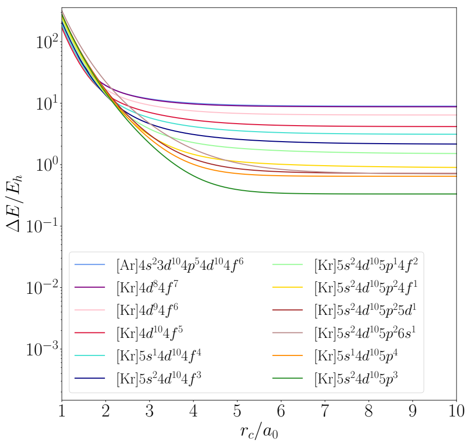

(a) PW92

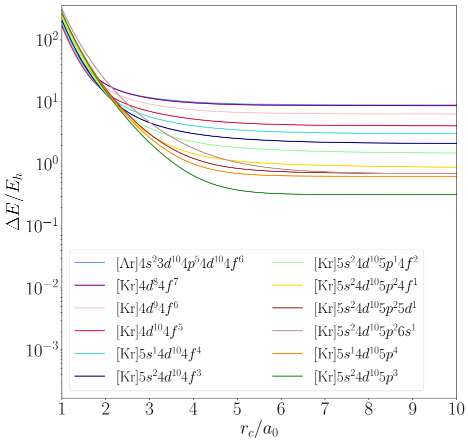

(b) PBE

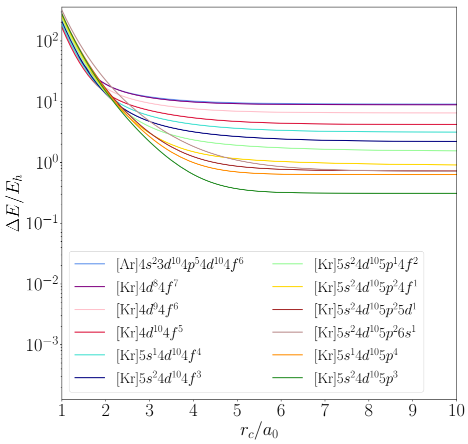

(c) r^{2}SCAN

Figure S217: Energies of various low lying configurations of the hard-wall confined spin-polarized monocation of Te shown as the energy difference from unconfined Te as a function of the confinement radius $r_\infty = 1.0, 1.1, \ldots, 10.0 a_0$. Note semilogarithmic scale.

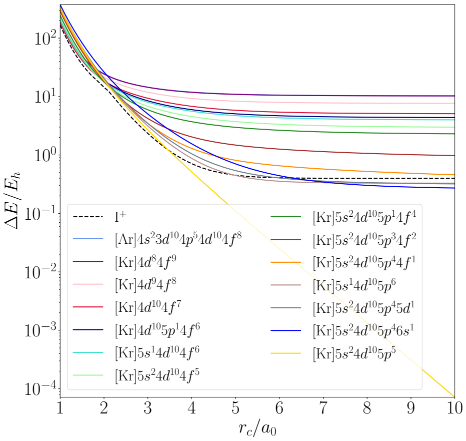

(a) PW92

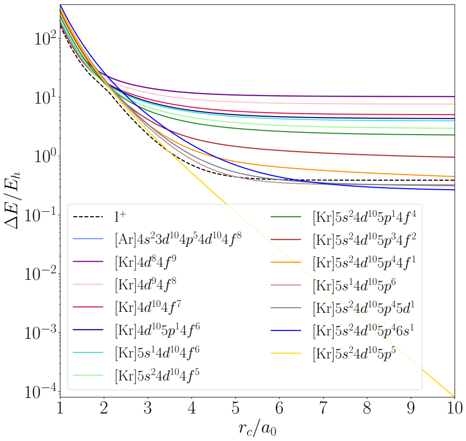

(b) PBE

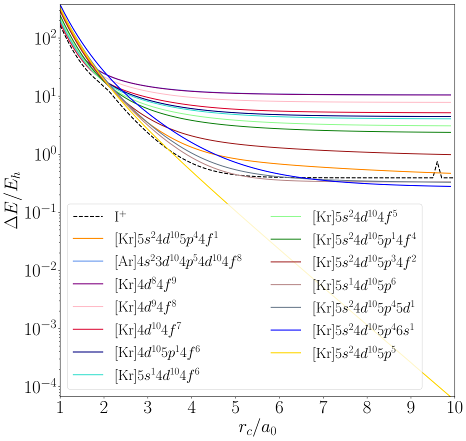

(c) r^{2}SCAN

Figure S218: Energies of various low lying configurations of hard-wall confined spin-polarized I shown as the energy difference from unconfined I as a function of the confinement radius $r_\infty = 1.0, 1.1, \ldots, 10.0a_0$. Note semilogarithmic scale.

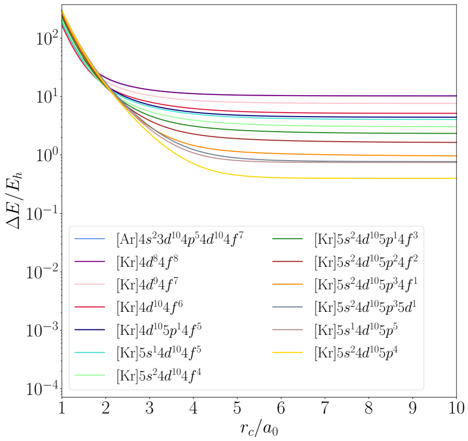

(a) PW92

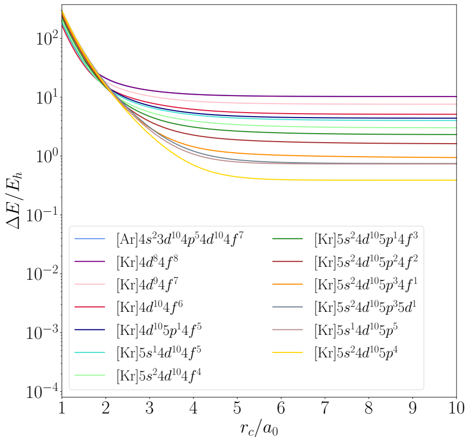

(b) PBE

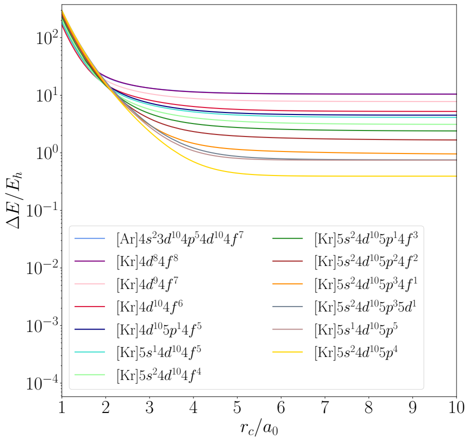

(c) r^{2}SCAN

Figure S219: Energies of various low lying configurations of the hard-wall confined spin-polarized monocation of I shown as the energy difference from unconfined I as a function of the confinement radius $r_\infty = 1.0, 1.1, \ldots, 10.0a_0$. Note semilogarithmic scale.

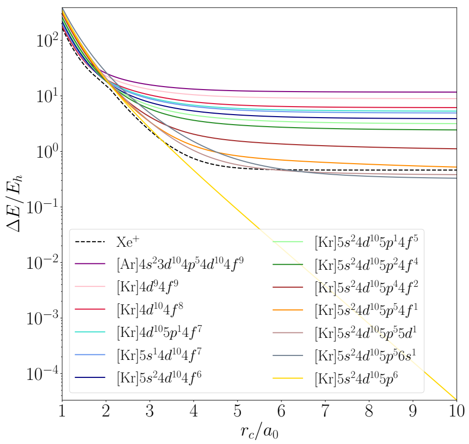

(a) PW92

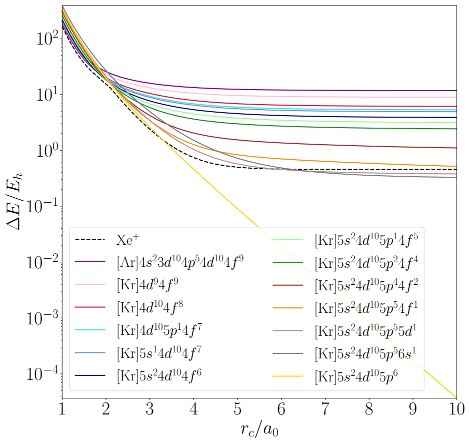

(b) PBE

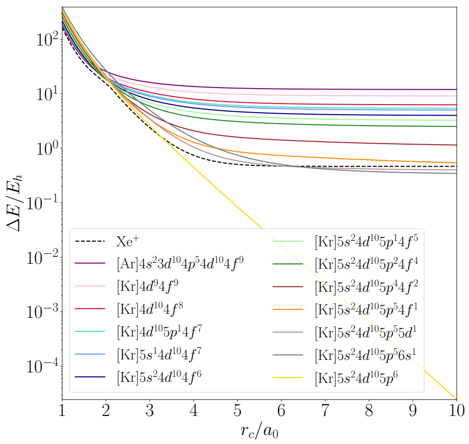

(c) r^{2}SCAN

Figure S220: Energies of various low lying configurations of hard-wall confined spin-polarized Xe shown as the energy difference from unconfined Xe as a function of the confinement radius $r_\infty = 1.0, 1.1, \ldots, 10.0a_0$. Note semilogarithmic scale.

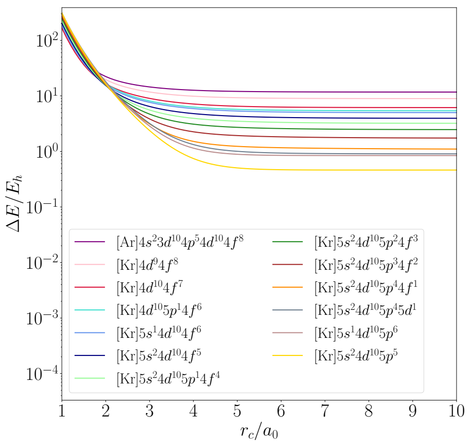

(a) PW92

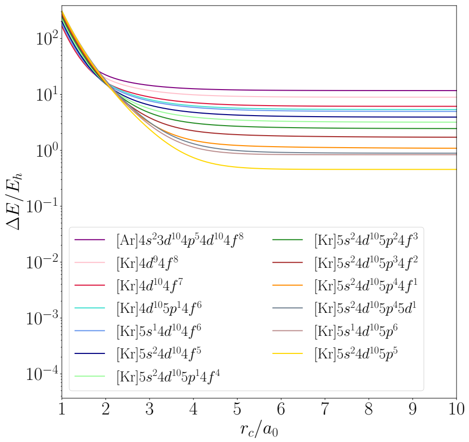

(b) PBE

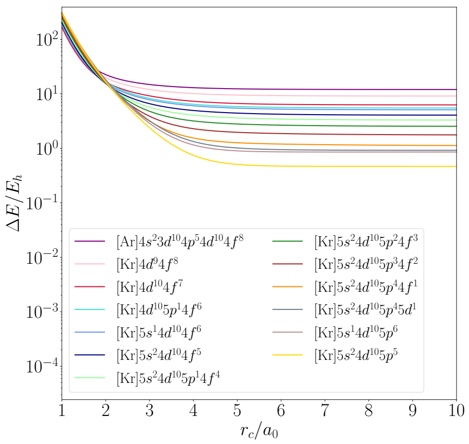

(c) r^{2}SCAN

Figure S221: Energies of various low lying configurations of the hard-wall confined spin-polarized monocation of Xe shown as the energy difference from unconfined Xe as a function of the confinement radius $r_\infty = 1.0, 1.1, \ldots, 10.0a_0$. Note semilogarithmic scale.
